# Iridium-Catalyzed Asymmetric Difunctionalization of C–C σ-Bonds Enabled by Ring-Strained Boronate Complexes

**DOI:** 10.1021/jacs.3c03248

**Published:** 2023-07-20

**Authors:** Hong-Cheng Shen, Mihai V. Popescu, Ze-Shu Wang, Louis de Lescure, Adam Noble, Robert S. Paton, Varinder K. Aggarwal

**Affiliations:** † School of Chemistry, University of Bristol, Cantock’s Close, Bristol BS8 1TS, U.K.; ‡ Department of Chemistry, Colorado State University, Ft. Collins, Colorado 80523-1872, United States

## Abstract

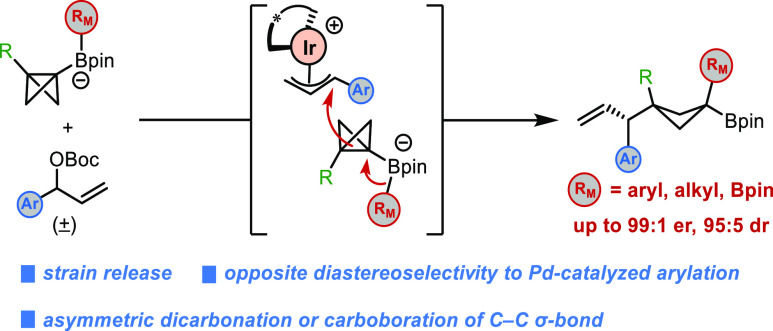

Enantioenriched organoboron intermediates are important building blocks in organic synthesis and drug discovery. Recently, transition metal-catalyzed enantioselective 1,2-metalate rearrangements of alkenylboronates have emerged as an attractive protocol to access these valuable reagents by installing two different carbon fragments across C=C π-bonds. Herein, we report the development of an iridium-catalyzed asymmetric allylation-induced 1,2-metalate rearrangement of bicyclo[1.1.0]butyl (BCB) boronate complexes enabled by strain release, which allows asymmetric difunctionalization of C–C σ-bonds, including dicarbonation and carboboration. This protocol provides a variety of enantioenriched three-dimensional 1,1,3-trisubstituted cyclobutane products bearing a boronic ester that can be readily derivatized. Notably, the reaction gives *trans* diastereoisomers that result from an *anti*-addition across the C–C σ-bond, which is in contrast to the *syn*-additions observed for reactions promoted by Pd^II^–aryl complexes and other electrophiles in our previous works. The diastereoselectivity has been rationalized based on a combination of experimental data and density functional theory calculations, which suggest that the BCB boronate complexes are highly nucleophilic and react via early transition states with low activation barriers.

## Introduction

**Scheme 1:**
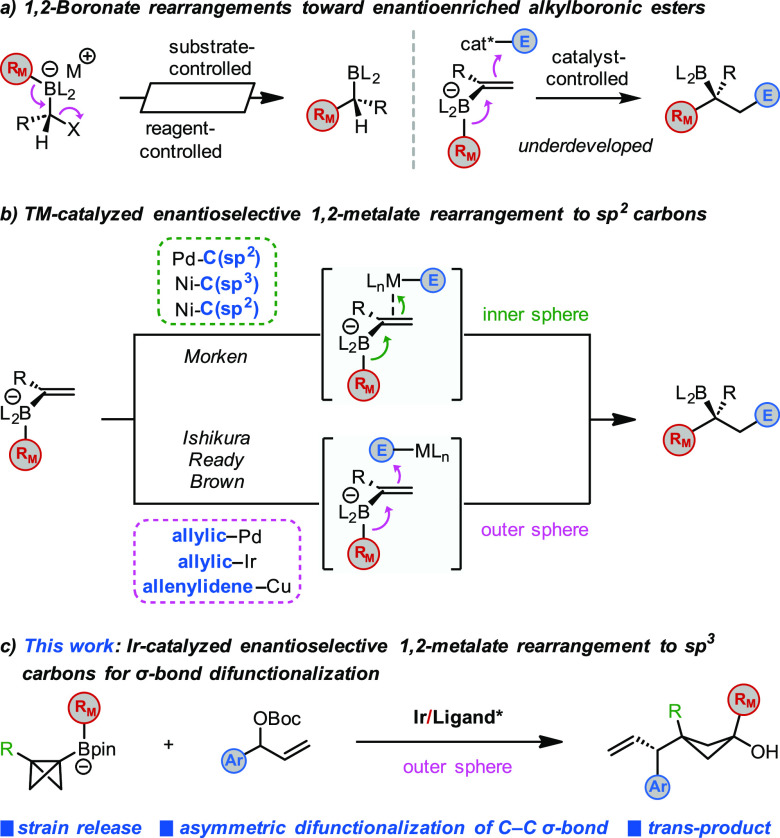
Transition Metal-Catalyzed Enantioselective 1,2-Metalate Rearrangements

Enantioenriched organoboron compounds are highly valuable reagents for the stereocontrolled assembly of complex molecules, with applications spanning organic synthesis and drug discovery. Their broad utility has led to the development of numerous methods for their construction,^1^ including stereospecific 1,2-metalate rearrangements of boronate complexes.^2^ These reactions typically proceed via homologation of boronic esters with metal carbenoids through either substrate-controlled homologation^3^ or the more versatile reagent-controlled lithiation–borylation (Scheme 1a, left).^2a,2d^ In contrast, catalytic enantioselective 1,2-metalate rearrangements of alkenylboronate complexes to access enantioenriched alkylboron reagents remain relatively underdeveloped (Scheme 1a, right). Recently, significant breakthroughs have been reported for reactions of alkenylboronate complexes with chiral transition metal-based complexes, which trigger a 1,2-migration of an alkyl or aryl group on boron to the neighboring sp^2^ carbon.^2b,2c^

Transition metal-promoted 1,2-metalate rearrangements of alkenylboronates can occur either through inner sphere (the π-bond directly interacts with the metal) or outer sphere (the boronate attacks the metal-bound electrophile) pathways.^2b,2c^ Morken showed that 1,2-metalate rearrangements of alkenylboronates could be conducted catalytically and enantioselectively with π-acidic late transition metal complexes, such as Pd^II^ and Ni^II^. Mechanistically, such reactions are believed to proceed by way of an inner sphere metal-induced migration pathway.^4,5^ Related reactions of alkenylboronates with electrophilic metal complexes, including π-allyl–Pd,^6,7^ π-allyl–Ir,^8^ and allenylidene–copper,^9^ were reported by Ishikura, Ready, and Brown and are believed to occur via an outer sphere pathway (Scheme 1b).

Reactions of metal complexes with C=C π-bonds leading to the installation of two different carbon fragments across the bonds are well known. However, related reactions with C–C σ-bonds are much less common since σ-bonds are typically strong, inert, and geometrically inaccessible. We recently showed that cationic Pd^II^ complexes could activate σ-bonds of bicyclo[1.1.0]butyl (BCB) boronate complexes to promote 1,2-boronate migrations to sp^3^ carbons and achieve a σ-bond dicarbonation.^10^ The reaction was driven by the high strain energy [63.9 kcal/mol (experimental)/66.3 kcal/mol (calculated)] of bicyclo[1.1.0]butanes^11^ and occurred with high diastereoselectivity for the formation of 1,1,3-trisubstituted cyclobutanes with a cis relationship between the two carbon substituents. Further studies suggested that the reaction occurred through a concerted pathway with the antiperiplanar alignment of the migrating group with the breaking σ-bond.^12^ The reactions of highly strained boronate complexes were subsequently expanded to the construction of boronic ester-substituted azetidines^13^ and other cyclobutanes.^14^ Despite this progress, its extension to the synthesis of enantioenriched boron reagents remains unexplored. We questioned whether chiral π-allyl metal complexes could induce 1,2-metalate rearrangements of strained BCB boronate complexes to give diastereo- and enantioenriched cyclobutanes. We were particularly attracted to the π-allyl iridium complexes developed by Carreira^15,16^ as they had been shown to be effective with a very broad range of nucleophiles, including very weak nucleophiles like alkenes.^15c^ Indeed, and relevant to this work, Ready showed that Carreira’s iridium(phosphoramidite) complexes were highly effective in the enantio- and diastereoselective allylation of alkenylboronates.^8^ Herein, we report success in our endeavor and the development of an iridium-catalyzed asymmetric allylation-induced 1,2-metalate rearrangement of BCB boronate complexes (Scheme 1c).^17,18^ This protocol allows asymmetric difunctionalization of C–C σ-bonds, providing a variety of enantioenriched 1,1,3-trisubstituted cyclobutane products bearing a boronic ester that can be readily derivatized. Notably, this 1,2-metalate rearrangement triggered by the π-allyl-iridium complex resulted in *anti*-addition across the C–C σ-bond, in contrast to reactions promoted by Pd^II^–aryl complexes and other electrophiles, which lead predominantly to *cis* diastereoisomers via a *syn*-addition.

## Results and Discussion

### Optimization Studies

We first investigated the iridium-catalyzed allylation of BCB boronate complex **3a**, derived from 2-naphthyl-Bpin **1** and bicyclo[1.1.0]butyl sulfoxide **2**, with racemic Boc-protected allylic alcohol **4a** in a series of solvents. Typically, the boronic ester product was oxidized to the corresponding cyclobutanol, although isolation of the cyclobutyl boronic ester was also possible. Full optimization of the process is provided in the Supporting Information, but some details are discussed below (Table 1). We were pleased to find that the reaction was successful in the presence of 2.5 mol % [Ir(cod)Cl]_2_ and 10 mol % phosphoramidite ligand **L1**(15,18b,18d) in a mixture of DCM and toluene to give 1,1,3-trisubstituted cyclobutane **5** in 75% NMR yield with 95:5 dr and 99:1 er (entry 1). Without the iridium catalyst and ligand, no product was obtained (entry 2). Product **5** was observed with slightly lower enantioselectivity when MeOH was not added before solvent exchange to quench any excess alkyllithium (entry 3, see the Supporting Information for additional details). We subsequently investigated different phosphoramidite ligands and found that **L2** led to similar efficiency but lower enantioselectivity (entry 4). Evaluation of different activating groups on the allylic alcohol showed that acetyl (**4b**) was less effective than Boc (entry 5). Finally, increasing the temperature resulted in a marginal increase in yield while still maintaining excellent diastereo- and enantioselectivity. The steric effects of the achiral boron ligand were also explored (see Table S2 in the Supporting Information for details),^4h^ which revealed that less hindered ligands (e.g., ethylene glycol) resulted in lower *anti*/*syn* selectivity compared to more hindered ligands (e.g., pinacol).

**Table 1:**
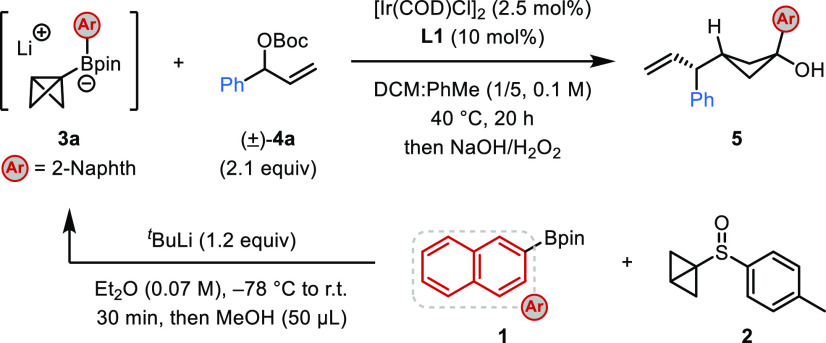
Optimization Studies^a^

entry	alteration to conditions	yield (%)	dr	er
1	none	75 (73^b^)	95:5	99:1
2	without [Ir(COD)Cl]_2_ and **L1**	0		
3	without MeOH	73	94:6	97:3
4	**L2**	64	94:6	21:79
5	**4b**	27	94:6	97:3
6	60 °C	77 (78^b^)	95:5	99:1

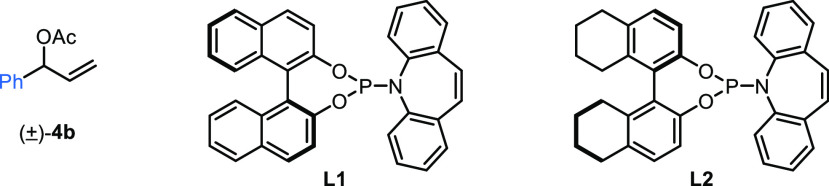

a **3a** was preformed using **1** (0.2 mmol), **2** (1.2 equiv), ^*t*^BuLi (1.2 equiv), Et_2_O, −78 °C, 5 min then r.t., 30 min. MeOH (50 μL) was added before concentration and addition of **4a** (2.1 equiv), [Ir(COD)Cl]_2_ (2.5 mol %), and 10 mol % of the ligand in DCM/toluene. The yields were determined by ^1^H NMR spectroscopy using 1,3,5-trimethoxybenzene as an internal standard; the dr and er were determined by HPLC analysis.

b Yield of the isolated product.

### Substrate Scope

Under the optimized reaction conditions (Table 1, entry 6), the generality of the reaction was then evaluated. As depicted in Table 2, this asymmetric difunctionalization of C–C σ-bonds catalyzed by iridium proceeded efficiently with a wide range of Boc-activated allylic alcohols. Various substituents on the phenyl group were tolerated, including both electron-donating and electron-withdrawing groups at the *para*-, *meta*-, and *ortho*- positions, leading to the formation of the corresponding 1,1,3-trisubstituted cyclobutane products **6–16** in 57–78% yields, 90:10–94:6 dr, and up to >99:1 er. In addition, substrates derived from other aromatic rings, such as naphthalene (**17**), pyridine (**18**), and indole (**19**), also furnished the products in good yields and stereoselectivities. Of note, attempts to extend the reaction to alkyl-substituted Boc-protected allylic alcohols only gave a trace of products.

**Table 2:**
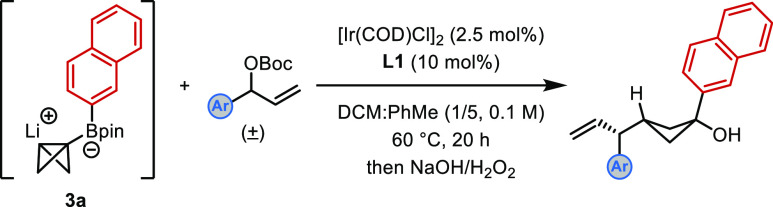

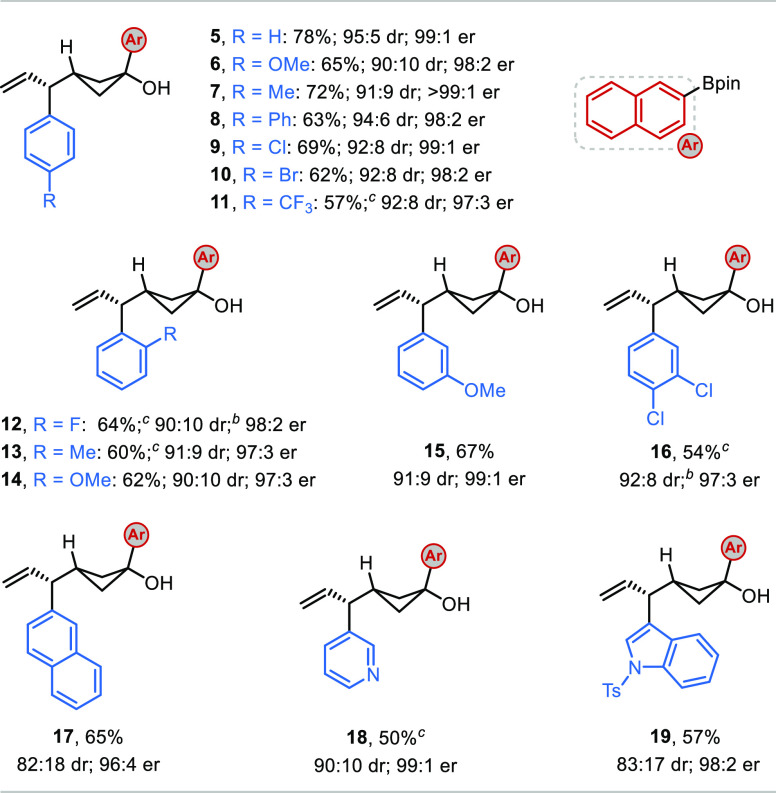
Substrate Scope of Allylic Carbonates^a^

a Reaction conditions: **3a** was preformed using **1** (0.2 mmol), **2** (1.2 equiv), ^*t*^BuLi (1.2 equiv), Et_2_O, −78 °C, 5 min, then r.t., 30 min. MeOH (50 μL) was added before concentration and addition of **4a** (2.1 equiv), [Ir(COD)Cl]_2_ (2.5 mol %), and 10 mol % **L1** in DCM/toluene = 1/5. Yields are for isolated products. The dr and er were determined by HPLC analysis.

b Determined by ^1^H NMR analysis.

c ^*t*^BuOH (50 μL) was added before concentration, and a 2:1 DCM/toluene mixture was used as the solvent.

The scope of the boronic ester was then explored using Boc-protected allylic alcohol **4a** as the standard substrate (Table 3). We first evaluated a range of aryl boronic esters (**20–26**) bearing both electron-withdrawing (**21–24**) and electron-donating groups (**25, 26**). They all gave moderate to good yields and excellent enantioselectivity. While sterically demanding substituents such as *o*-Cl gave moderate diastereoselectivity, *para* and *meta* substituents allowed for product formation with good diastereoselectivity. Dichlorophenyl and 1-naphthyl migrated efficiently to deliver the products in good yields and selectivity (**27**, **28**). Boronates derived from heterocycles, including indole, furan, and thiophene, were also investigated, affording the corresponding products (**29–31**) in good efficiencies. Additionally, primary alkyl boronic esters worked equally well, with 2-phenethyl (**32**) and methyl (**33**) boronic esters giving the cyclobutane products in good yields, good diastereoselectivity, and very high enantioselectivity. Sterically demanding secondary and tertiary boronic esters (**34–36**) could also be employed, giving good to excellent enantioselectivity, albeit with reduced diastereoselectivity. The boronate derived from the prop-1-en-2-yl boronic ester furnished the desired product (**37**) in good yield with high selectivity. This example is particularly noteworthy in that the boronate complex can react with the π-allyl complex on either the π-bond (forming **37′**) or the σ-bond, but it reacted exclusively on the σ-bond.^8^ In the reaction of bis(pinacolato)diboron-derived ate complexes, carboboration occurred at the C–C σ-bond to give **38** in a 61% yield and 97:3 er.

**Table 3:**
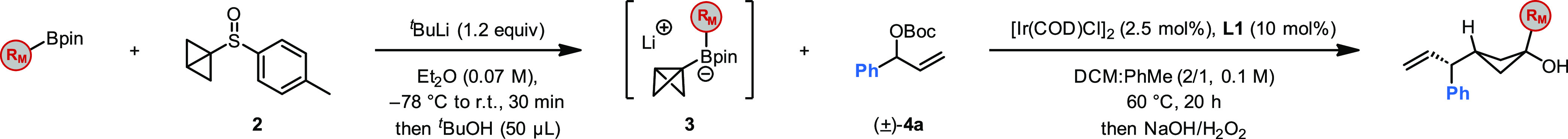

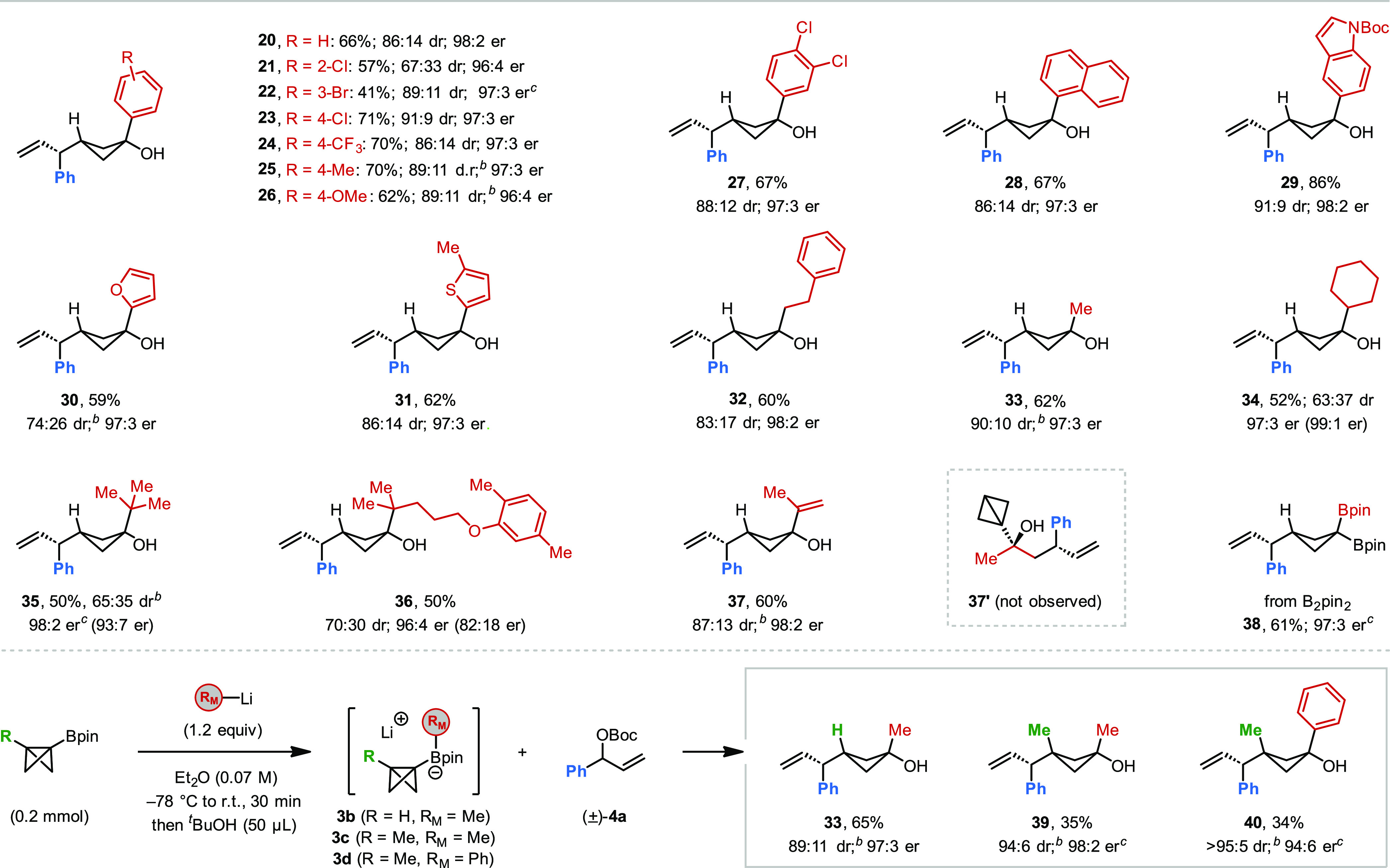
Substrate Scope of Bicyclo[1.1.0]butyl Boronates^a^

a Reaction conditions: **3** was preformed using boronic esters (0.2 mmol), **2** (1.2 equiv), ^*t*^BuLi (1.2 equiv), Et_2_O, −78 °C, 5 min then r.t., 30 min. ^*t*^BuOH (50 μL) was added before concentration and addition of **4a** (2.1 equiv), [Ir(COD)Cl]_2_ (2.5 mol %), and 10 mol % **L1** in DCM/toluene = 2/1. The yields are of isolated products. The dr and er were determined by HPLC analysis. The er of the minor diastereoisomer is shown in parentheses.

b Determined by ^1^H NMR analysis.

c Determined by HPLC analysis after the derivatization.

BCB boronate complexes can be accessed either through the reaction of BCB lithium reagents with boronic esters or through the addition of organolithium reagents to BCB–Bpin substrates. We adopted the latter approach to further investigate the substitution effects at the BCB ring (Table 3, bottom). The boronate derived from methyl lithium and BCB–Bpin furnished product **33** with comparable yield and stereoselectivity compared to that obtained through the reaction of BCB lithium with methyl boronic ester. Finally, this asymmetric difunctionalization of C–C σ-bonds was also achieved for more hindered β-methyl-substituted BCB boronate complexes. Both methyl and phenyl migrating groups provided products **39** and **40**, respectively, in moderate yields but with excellent diastereo- and enantioselectivity. Notably, the C–C σ-bond at the β-methyl-substituted BCB boronate complex was unreactive toward cationic Pd^II^ complexes in our previous work, which suggests that the π-allyl-iridium complex is a considerably more reactive electrophile than Pd^II^–aryl complexes.^10,12^

### Gram-Scale Reactions and Product Elaborations

**Scheme 2:**
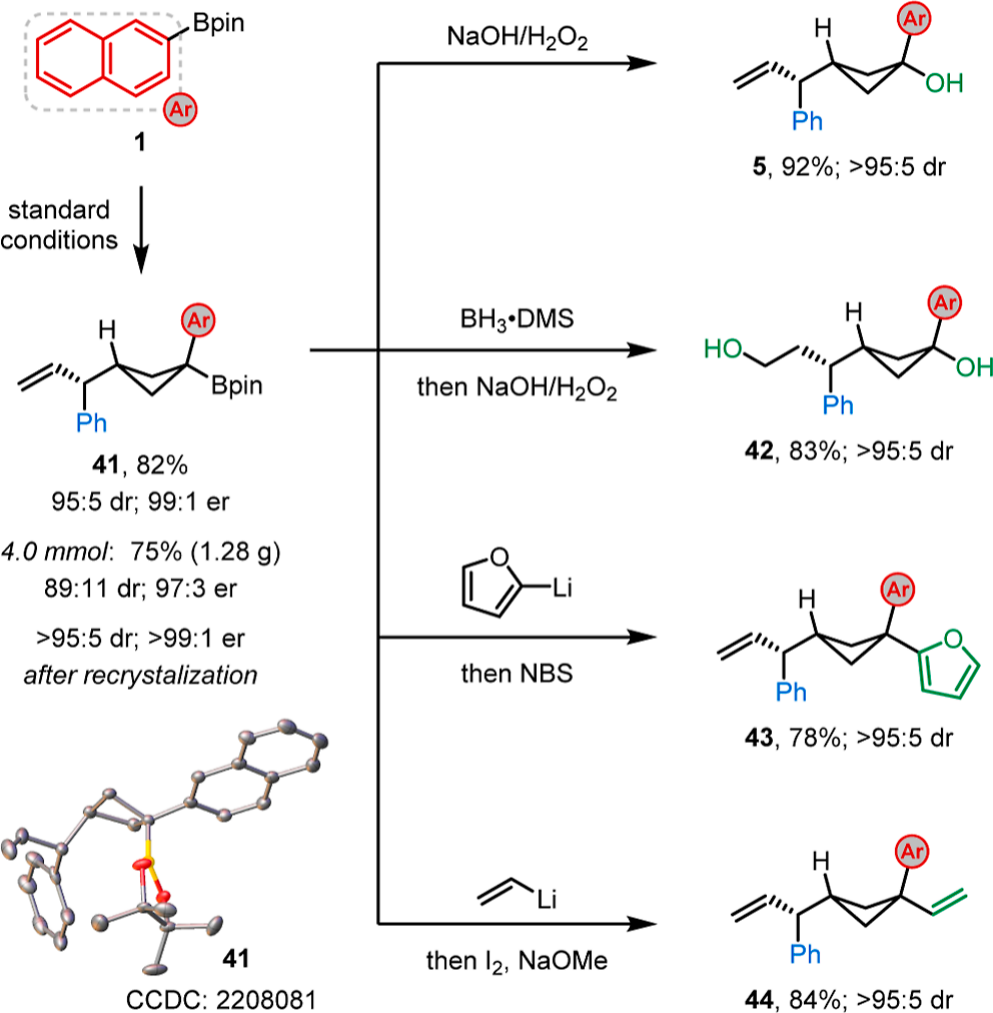
Synthesis of the Enantioenriched Cyclobutyl Boronic Ester and Further Applications See the Supporting Information for reaction conditions.

To demonstrate the synthetic utility of this methodology, the standard reaction conditions were conducted on a gram-scale to afford the desired enantioenriched cyclobutyl boronic ester **41** with only slightly decreased stereoselectivity compared to the small-scale reaction (Scheme 2). The relative stereochemistry of boronic ester **41** was determined by X-ray diffraction experiments (see Figure S2 and Table S4 in the Supporting Information for details). Further functionalizations were performed on boronic ester **41**, which could be obtained in >95:5 dr and >99:1 er after recrystallization.^19^ Oxidation of **41** gave alcohol **5**, and hydroboration/oxidation gave diol **42**, both in excellent yields. Furan **43** and diene **44** were obtained in high yields and with complete stereospecificity by employing our transition metal-free cross-coupling and Zweifel olefination, respectively.^20^

### Mechanistic Studies

**Scheme 3:**
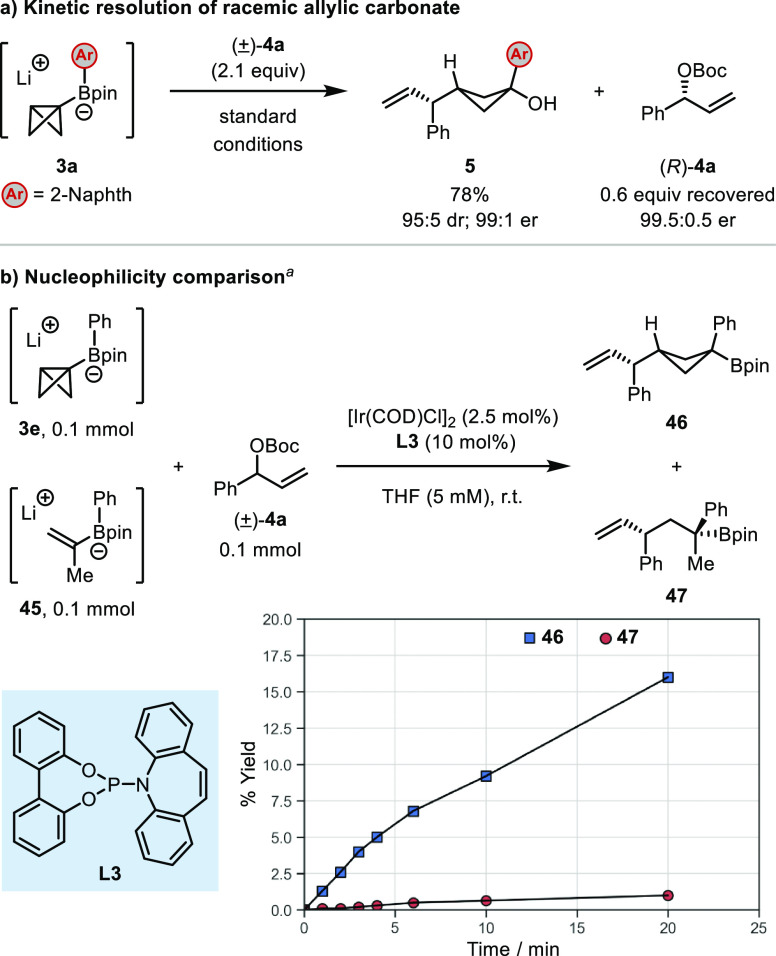
Mechanistic Studies The yields were determined by GC analysis using hexadecane as an internal standard.

We subsequently performed a series of experiments to probe the mechanism of this asymmetric allylation of BCB boronate complexes (Scheme 3). To determine if the racemic carbonate **4a** was undergoing a kinetic resolution, we conducted the allylation using boronate complex **3a** and isolated both the allylation product **5** and the unreacted **4a** (Scheme 3a). Recovered carbonate (*R*)-**4a** was isolated in 99.5:0.5 er, indicating that an efficient kinetic resolution had occurred.^8a,21^ The high kinetic resolution is a result of one enantiomer of the substrate **4a** undergoing oxidative addition much more rapidly than the other and is in keeping with Carreira’s and Ready’s mechanistic studies of such systems.^8,16,18b^ Next, we studied the relative nucleophilicity of BCB boronate complex **3e** by performing a competition reaction with alkenylboronate complex **45** (Scheme 3b). Recently, Ready et al. investigated the nucleophilicity of **45** and determined that it had a nucleophilicity parameter of *N* = 11 on the Mayr scale,^8b^ which is similar to allylboronate complexes.^22^ Using alkenylboronate complex **45** as a benchmark, we compared the nucleophilicity of our BCB boronate complex **3e**. Thus, equal amounts of **3e** and **45** were allowed to react with **4a** using the achiral ligand **L3**,^23^ and the reaction was monitored using GC analysis. The results showed that **3e** is almost an order of magnitude more reactive than **45**. This increased reactivity of the BCB boronate over the alkenylboronate is consistent with the chemoselectivity observed in the formation of product **37** (Table 3). Furthermore, it is consistent with the chemoselectivity observed in related palladium-catalyzed arylations,^10^ where a boronate derived from vinyl boronic acid pinacol ester and BCB-lithium reacted exclusively at the σ-bond. In both cases, the high reactivity of the C–C σ-bond presumably results from the substantial π-character of this bond and the additional driving force provided by the release of ring strain.

**Scheme 4:**
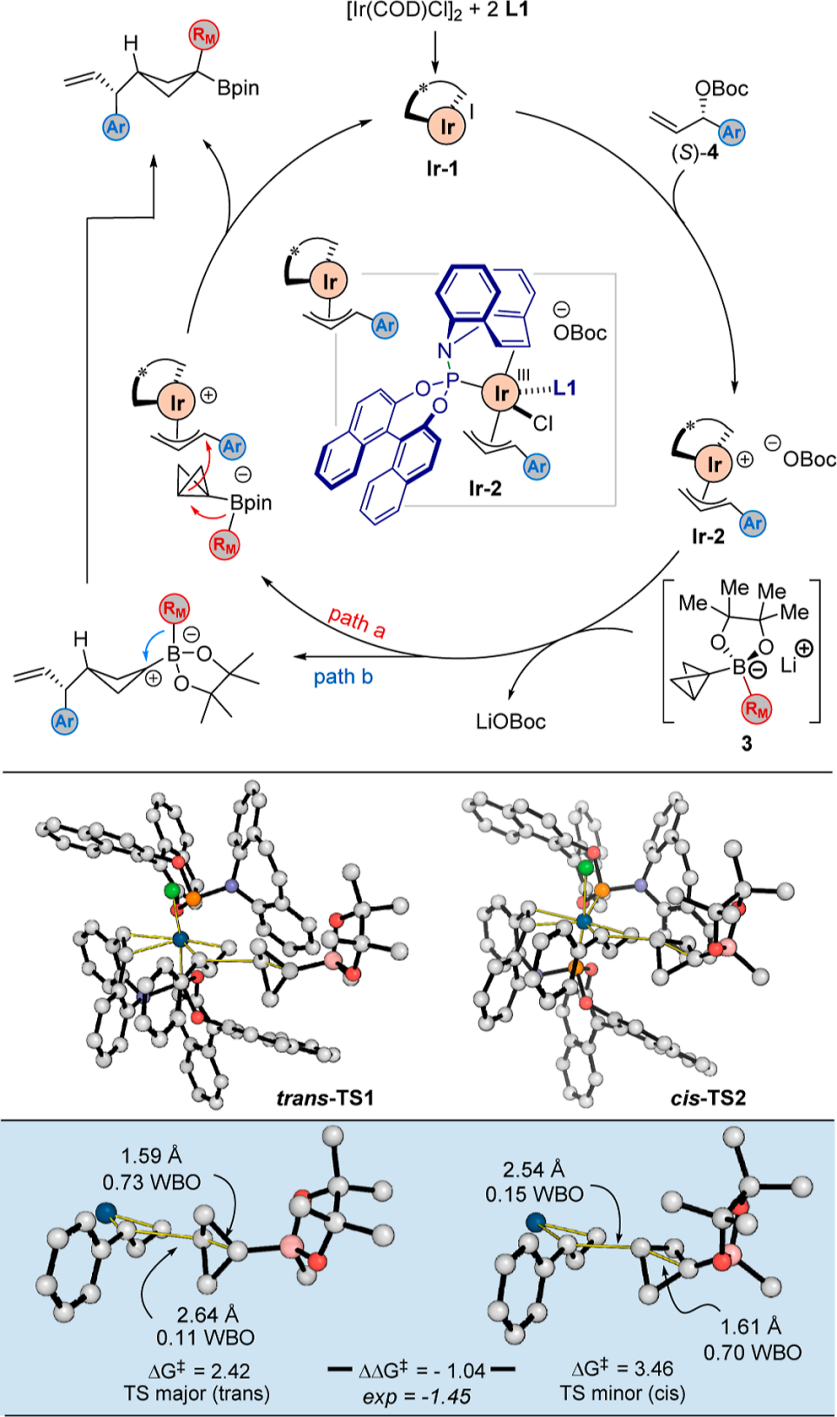
Proposed Catalytic Cycle and Computed TSs

A proposed catalytic cycle for this reaction is illustrated in Scheme 4.^8b,16,18b^ The active catalytic species **Ir-1**, generated from an Ir^I^ precursor and 2 equiv of phosphoramidite/olefin ligand **L1**, undergoes oxidative addition with high enantioselectivity for the (*S*)-enantiomer of the allylic electrophile **4** to furnish the π-allyl-iridium intermediate **Ir-2**.^8^ This highly electrophilic species can then react with BCB boronate complex **3**, which induces a 1,2-boronate migration either in a concerted process (path a) or in a stepwise manner (path b), depending on the initial conformation of the boronate complex.

We subsequently performed density functional theory (DFT) calculations to investigate the nature of the reaction of **Ir-2** with **3** and determine the origins of diastereoselectivity.^8b,15f,24^ We first computed the potential energy surface (PES) of the reaction of BCB boronate **3b** (R_M_ = Me) with the tropylium ion as a simplified model cationic electrophile (see Supporting Information). This confirmed that C–C bond formation occurs in an exergonic and irreversible fashion. We were able to characterize both concerted and stepwise pathways on the PES; however, for path b, the computed barrier for the 1,2-shift is extremely small at around 0.3 kcal/mol. Based on these data, we propose that C–C bond-forming transition structures (TSs) are diastereoselectivity-determining since rotation about the exocyclic C–B bond cannot occur during either pathway. Therefore, to determine the origins of diastereoselectivity for the allylation reaction, we focused our attention on the C–C bond-forming TSs leading to *trans*- and *cis*-diastereomeric products for the reaction of **3b** with π-allyl-iridium complex **Ir-2**. Calculations were performed at the M06/6-311+G(d,p)-SDD(Ir)//B3LYP/6-31G(d)-LANL2DZ(Ir) level of theory, and an SMD model for dichloromethane was used during all stages.^25,26^ Note that since enantioselectivity and regioselectivity were not the focus of this study, we only computed the TSs involving the major enantiomer and major regioisomer. Following conformational analysis, we located TSs for the C–C bond-forming step between **Ir-2** and **3b** (R_M_ = Me), and the two lowest energy competing TSs are shown in Scheme 4, while additional higher-energy TS conformations are depicted in the Supporting Information Figure S3. Consistent with the experimentally observed selectivity, ***trans*-TS1** is lower in energy than ***cis*-TS2** by 1.04 kcal/mol (c.f. experimental value of 1.45 kcal/mol). Structurally, ***trans*-TS1** and ***cis*-TS2** are early transition states, with long forming C–C bonds of 2.64 and 2.54 Å, and short internal breaking C–C bonds of the BCB moiety of 1.59 and 1.61 Å, respectively, compared to their equilibrium lengths of 1.50–1.51 Å. These results were further supported by Wiberg bond order calculations,^27^ which revealed bond orders of 0.11 and 0.15 for the forming C–C bonds in ***trans*-TS1** and ***cis*-TS2**, respectively. Likewise, the internal breaking C–C bonds of the BCB group show a marginal decrease from 0.89 in the ground state to 0.73 in ***trans*-TS1**, with a similar change of 0.89 to 0.70 for ***cis*-TS2**. These distances are consistent with very small activation barriers, measured relative to the most stable ion–pair complex, of 2.4–3.5 kcal/mol. These values are lower than those obtained for the addition of alkenylboronates to a π-allyl-iridium electrophile (4.3 kcal/mol),^8b^ consistent with the greater nucleophilicity of the BCB boronate (Scheme 3b). For both diastereomeric pathways, the boronate approaches from an open region of space relatively unhindered by either phosphoramidite ligand, and we could not identify any appreciable noncovalent (e.g., dispersive) interactions favoring the *trans*-pathway.^28^ Indeed, other *trans*-TS conformers formed from rotation about the incipient C–C bond are also lower in energy than all *cis*-conformers (see Supporting Information, Table S6), suggesting that the difference in energy is not due to stabilizing intermolecular interactions.

Selective formation of the *trans*-product is intriguing since this behavior contrasts with our previously reported *cis*-selective reactions of BCB boronate complexes with other electrophiles. We have previously hypothesized that the preferential formation of the *cis* product is favored due to a stabilizing antiperiplanar orientation between the internal C–C bond of the BCB group and the B–C bond of the migrating group, which is only present in the TS leading to the *cis* product.^12^ However, there was no evidence of any degree of migration of the substituent on boron in the computed TSs, implying that such stabilizing effects do not influence the diastereoselectivity of reactions with π-allyl-iridium complexes.

**Scheme 5:**
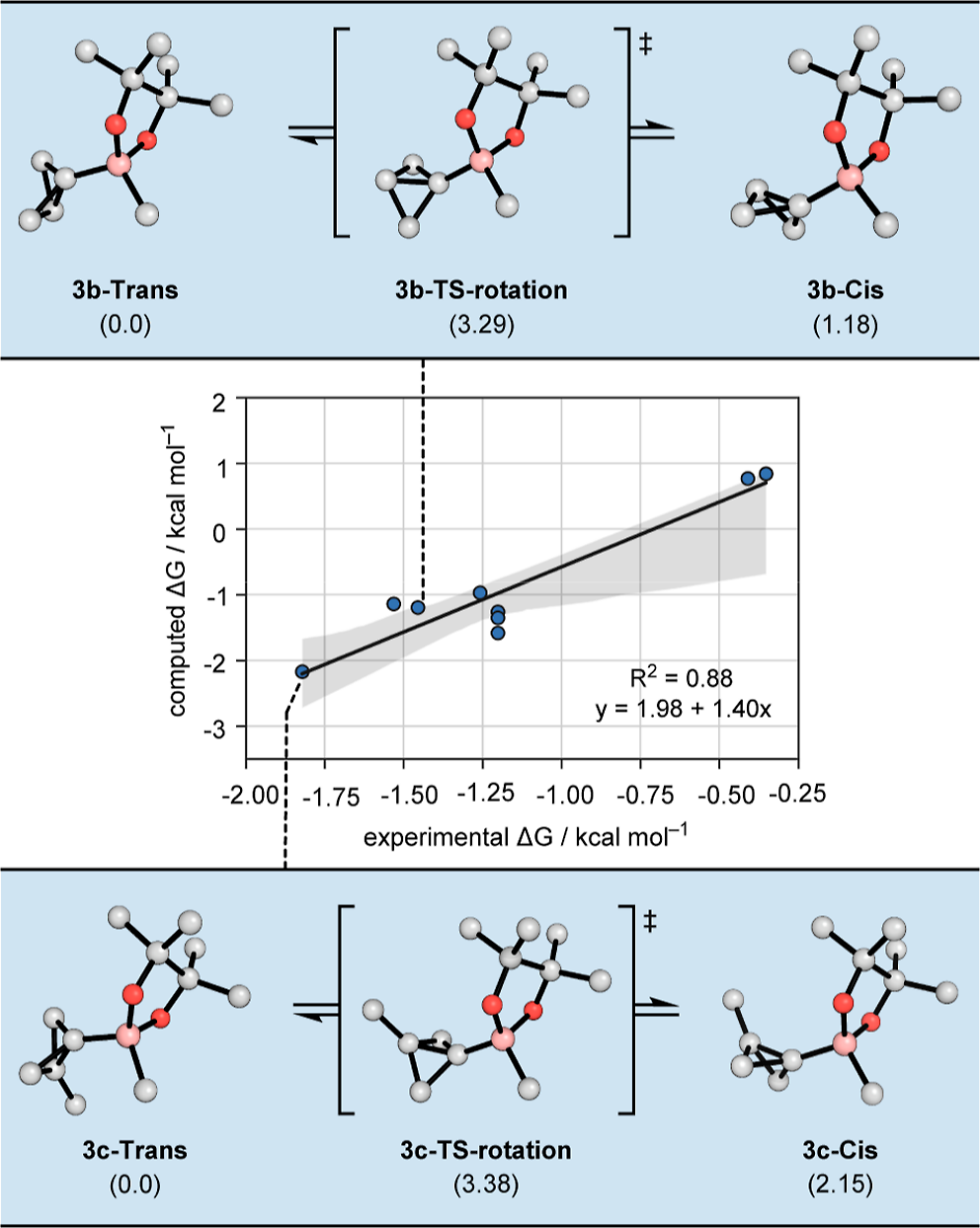
Rotational Profile of BCB Boronate Complexes **3b** (Top) and **3c** (Bottom) and Correlation of BCB Conformational Preferences and Experimentally Observed Product Diastereoselectivities with Various Alkyl, Alkenyl, and Aryl Substituents (Middle)

Another possibility is that the diastereoselectivity is controlled by the ground-state conformation of the boronate complex. To further probe this hypothesis, we computed the equilibrium conformer populations of a wide range of boronate complexes and compared the results with the experimentally observed selectivity values (Scheme 5). A good Pearson correlation coefficient of *R*^2^ = 0.88 was obtained, indicating a correlation between the equilibrium preference of the boronate complexes and the experimental outcome. In general, except for highly sterically hindered cyclohexyl and *tert*-butyl migrating groups, the *trans*-conformation of the boronate complex was found to be highly preferred. We ascribe the success of these computed ground-state energy differences in accounting for diastereoselectivity in terms of the Hammond postulate: highly nucleophilic BCB boronates react via extremely early and low-barrier C–C bond-forming TSs, in which the BCB structure closely resembles that of the ground-state. Conformational preferences about the C–B bond are therefore shared in the TSs, and as stated above, C–B bond rotation does not occur before the 1,2-shift. Another consequence of such early TSs with little C–C breaking is that the importance of hyperconjugative effects, which tend to favor the *cis* product,^10,12^ are diminished.

The contrasting behavior of the BCB boronates with Ir and Pd complexes can now be understood. In the case of the highly electrophilic π-allyl–Ir complexes, the reactions occur through a very early TS, and the diastereoselectivity is governed by the ground-state conformation of the BCB boronate complex, which favors the formation of the *trans* diastereoisomer. In the case of the less reactive Pd^II^–aryl complexes^10^ (and other less reactive electrophiles),^12^ the later TS benefits from the antiperiplanar alignment of the migrating group on boron with the breaking C–C σ-bond, which leads predominantly to the *cis* diastereoisomer.

An intriguing alternative hypothesis is that the low barriers for C–C bond formation mean these additions may not operate under Curtin–Hammett control, such that ground-state conformational populations and rates of interconversion directly influence the diastereoselectivity. To examine this possibility, we computed rotational barriers for equilibration between the trans and *cis* forms of BCB boronate **3b** and β-methyl-substituted BCB **3c** (TS-rotation, Scheme 5). We also studied a range of different boron substituents (Table S2). Depending on the direction of rotation, computed barriers range between 1.2 and 3.4 kcal/mol. With smaller alkyl and aryl substituents, the *trans*-bicyclobutane conformer is favored since this avoids unfavorable eclipsing steric interactions between Bpin and the C_1_–C_3_ bond present in the *cis*-conformer. For larger substituents, this preference is inverted due to similar steric interactions involving the substituent (Figure S5). As the magnitudes of the rotational barriers are comparable to those of **TS1** and **TS2**, it is difficult to disprove the presence or absence of Curtin–Hammett behavior unequivocally. However, based on previous mechanistic insight from Ready et al.,^8b^ the energy barrier for the formation of the π-allyl complex **Ir-2** from **Ir-1** and **4** is significantly higher than our computed rotational barriers, which provides ample time for thermal equilibration of the boronate complexes before each catalytic turnover of the iridium complex. Therefore, Curtin–Hammett control is likely for the present reaction.

## Conclusions

In summary, we have described an Ir-catalyzed asymmetric allylation-induced 1,2-boronate migration, leading to the difunctionalization of C–C σ-bonds of strained boronate complexes. The methodology enables the modular synthesis of 1,1,3-trisubstituted cyclobutyl boronic esters with very high enantioselectivity and moderate to excellent diastereoselectivity. The versatile reactivity of organoboron compounds makes these products valuable building blocks for accessing enantioenriched three-dimensional cyclobutanes, which are present in a broad range of natural products and drugs.^29^ More importantly, this process unveils a new strategy for asymmetric difunctionalization of C–C σ-bonds, including dicarbonation and carboboration. Computational studies of competing allylation TSs are consistent with the experimentally observed selectivity for the *trans*-diastereoisomer. The BCB boronate complexes are highly nucleophilic and react via early transition states with low activation barriers, with the diastereoselectivity reflecting the ground-state conformational preference of the BCB boronate substrates, in accordance with Hammond’s postulate.

## Supporting Information Available

Experimental procedures, characterization data, HPLC spectra, and NMR spectra of compounds (PDF)
