# Supplementary material for: Iridium-Catalyzed Asymmetric Difunctionalization of C–C σ-Bonds Enabled by Ring-Strained Boronate Complexes
Source: J Am Chem Soc. 2023 Jul 20;145(30):16508–16. doi: 10.1021/jacs.3c03248 (PMC10401714; doi:10.1021/jacs.3c03248)
Supplement: Supplementary file 1 — ja3c03248_si_001.pdf [file ja3c03248_si_001.pdf]

SUPPORTING INFORMATION

Iridium-Catalyzed Asymmetric Difunctionalization of C–C σ-Bonds Enabled by Ring Strained Boronate Complexes

Hong-Cheng Shen[a], Mihai V. Popescu[b], Ze-Shu Wang[a], Louis de Lescure[b], Adam Noble[a], Robert S. Paton*[b] and Varinder K. Aggarwal*[a]

[a]*School of Chemistry, University of Bristol, Cantock's Close, Bristol BS8 1TS, UK*
*e-mail: v.aggarwal@bristol.ac.uk
[b]*Department of Chemistry, Colorado State University, Ft. Col-lins, Colorado 80523-1872, US*
*e-mail: robert.paton@colostate.edu

TABLE OF CONTENTS

1. Materials and General Methods .... - 4 -
1.1. Glassware, Solvents, and Reagents .... - 4 -
1.2. Chromatography and Instrumentation........ - 4 -
1.3. Naming of Compounds .... - 4 -
2. Experimental Data........ - 6 -
2.1. Reaction Optimization........ - 6 -
2.2. General Procedures .... - 7 -
2.2.1. General Procedure A: Preparation of Bicyclo[1.1.0]butyl Boronate Complexes from BCB-sulfoxide .... - 7 -
2.2.2. General Procedure B: For the formation of product 5-10, 14-15, 17, 19 .... - 7 -
2.2.3. General Procedure C: For the formation of product 11-13, 16, 18, 20-38 .... - 8 -
2.2.4. General Procedure D: For the formation of product 39, 40 .... - 8 -
2.2.5. Procedure E: For gram-scale reaction with the formation of product 41 .... - 9 -
2.3. Procedures for Transformation of Products .... - 9 -
2.3.1. Procedure F: For the formation of product 5 from product 41 .... - 9 -
2.3.2. Procedure G: For the formation of product 42 from product 41........ - 10 -
2.3.3. Procedure H: For the formation of product 43 from product 41........ - 10 -
2.3.4. Procedure I: For the formation of product 44 from product 41 .... - 10 -
2.3.5. Procedure J: For hydroboration-oxidation of products .... - 11 -
2.4 Unsuccessful Examples........ - 11 -
2.5. Procedures for Mechanistic Studies........ - 12 -
2.5.1. Kinetic resolution of racemic carbonate........ - 12 -
2.5.2. Nucleophilicity comparison between alkenylboronate complex 45 and BCB boronate complex 3e .... - 12 -
2.5.3. Table S2. Substituent effects for diastereoselectivity........ - 14 -
2.6. Synthesis of Starting Materials .... - 15 -
2.6.1. Synthesis of racemic allylic carbonates .... - 15 -
2.6.2. Synthesis of bicyclo[1.1.0]butyl sulfoxide .... - 15 -
2.6.3. Table S3. Synthesis of noncommercially available boronic esters used in this project. .... - 15 -
2.7. Crystallography........ - 16 -
2.8. Characterization Data .... - 18 -

(*1s*,*3s*)-1-(Naphthalen-2-yl)-3-((*R*)-1-phenylallyl)cyclobutan-1-ol (5)............................. - 18 -
(*1s*,*3s*)-3-((*R*)-1-(4-Methoxyphenyl)allyl)-1-(naphthalen-2-yl)cyclobutan-1-ol (6) .... - 18 -
(*1s*,*3s*)-1-(Naphthalen-2-yl)-3-((*R*)-1-(p-tolyl)allyl)cyclobutan-1-ol (7) .... - 18 -
(*1s*,*3s*)-3-((*R*)-1-([1,1'-Biphenyl]-4-yl)allyl)-1-(naphthalen-2-yl)cyclobutan-1-ol (8) .... - 19 -
(*1s*,*3s*)-3-((*R*)-1-(4-Chlorophenyl)allyl)-1-(naphthalen-2-yl)cyclobutan-1-ol (9) .... - 19 -
(*1s*,*3s*)-3-((*R*)-1-(4-Bromophenyl)allyl)-1-(naphthalen-2-yl)cyclobutan-1-ol (10) .... - 19 -
(*1s*,*3s*)-1-(Naphthalen-2-yl)-3-((*R*)-1-(4-(trifluoromethyl)phenyl)allyl)cyclobutan-1-ol (11) - 19 -
(*1s*,*3s*)-3-((*R*)-1-(2-Fluorophenyl)allyl)-1-(naphthalen-2-yl)cyclobutan-1-ol (12)............. - 20 -
(*1s*,*3s*)-1-(Naphthalen-2-yl)-3-((*R*)-1-(o-tolyl)allyl)cyclobutan-1-ol (13) .... - 20 -
(*1s*,*3s*)-3-((*R*)-1-(2-Methoxyphenyl)allyl)-1-(naphthalen-2-yl)cyclobutan-1-ol (14) .... - 20 -
(*1s*,*3s*)-3-((*R*)-1-(3-Methoxyphenyl)allyl)-1-(naphthalen-2-yl)cyclobutan-1-ol (15) .... - 21 -
(*1s*,*3s*)-3-((*R*)-1-(3,4-Dichlorophenyl)allyl)-1-(naphthalen-2-yl)cyclobutan-1-ol (16)....... - 21 -
(*1s*,*3s*)-1-(Naphthalen-2-yl)-3-((*R*)-1-(naphthalen-2-yl)allyl)cyclobutan-1-ol (17) .... - 21 -
(*1s*,*3s*)-1-(Naphthalen-2-yl)-3-((*R*)-1-(pyridin-3-yl)allyl)cyclobutan-1-ol (18) .... - 22 -
(*1s*,*3s*)-1-(Naphthalen-2-yl)-3-((*R*)-1-(1-tosyl-1H-indol-3-yl)allyl)cyclobutan-1-ol (19) .... - 22 -
(*1s*,*3s*)-1-Phenyl-3-((*R*)-1-phenylallyl)cyclobutan-1-ol (20) .... - 22 -
(*1s*,*3s*)-1-(2- Chlorophenyl)-3-((*R*)-1-phenylallyl)cyclobutan-1-ol (21)........................... - 22 -
(*1s*,*3s*)-1-(3-Bromophenyl)-3-((*R*)-1-phenylallyl)cyclobutan-1-ol (22)............................ - 23 -
(*1s*,*3s*)-1-(4-Chlorophenyl)-3-((*R*)-1-phenylallyl)cyclobutan-1-ol (23)............................ - 23 -
(*1s*,*3s*)-3-((*R*)-1-Phenylallyl)-1-(4-(trifluoromethyl)phenyl)cyclobutan-1-ol (24) .... - 23 -
(*1s*,*3s*)-3-((*R*)-1-Phenylallyl)-1-(p-tolyl)cyclobutan-1-ol (25).. - 23 -
(*1s*,*3s*)-1-(4-Methoxyphenyl)-3-((*R*)-1-phenylallyl)cyclobutan-1-ol (26)......................... - 24 -
(*1s*,*3s*)-1-(3,4-Dichlorophenyl)-3-((*R*)-1-phenylallyl)cyclobutan-1-ol (27) .... - 24 -
(*1s*,*3s*)-1-(Naphthalen-1-yl)-3-((*R*)-1-phenylallyl)cyclobutan-1-ol (28)........................... - 24 -
tert-Butyl 5-((*1s*,*3s*)-1-hydroxy-3-((*R*)-1-phenylallyl)cyclobutyl)-1H-indole-1-carboxylate (29)- 25 -
(*1s*,*3s*)-1-(Furan-2-yl)-3-((*R*)-1-phenylallyl)cyclobutan-1-ol (30) .... - 25 -
(*1s*,*3s*)-1-(5-Methylthiophen-2-yl)-3-((*R*)-1-phenylallyl)cyclobutan-1-ol (31).................. - 25 -
(*1s*,*3r*)-1-Phenethyl-3-((*R*)-1-phenylallyl)cyclobutan-1-ol (32)....................................... - 25 -
(*1s*,*3s*)-1-Methyl-3-((*R*)-1-phenylallyl)cyclobutan-1-ol (33) .... - 26 -
(*1s*,*3s*)-1-Cyclohexyl-3-((*R*)-1-phenylallyl)cyclobutan-1-ol (34)...................................... - 26 -
(*1s*,*3s*)-1-(tert-Butyl)-3-((*R*)-1-phenylallyl)cyclobutan-1-ol (35) .... - 26 -
(*1s*,*3s*)-1-(5-(2,5-Dimethylphenoxy)-2-methylpentan-2-yl)-3-((*R*)-1-phenylallyl)cyclobutan-1-ol (36) .... - 26 -

(*1s*,*3s*)-3-((*R*)-1-Phenylallyl)-1-(prop-1-en-2-yl)cyclobutan-1-ol (37) .... - 27 -
(*R*)-2,2'-(3-(1-Phenylallyl)cyclobutane-1,1-diyl)bis(4,4,5,5-tetramethyl-1,3,2-dioxaborolane) (38) .... - 27 -
(*1s*,*3s*)-1,3-Dimethyl-3-((*R*)-1-phenylallyl)cyclobutan-1-ol (39) .... - 27 -
(*1s*,*3s*)-3-Methyl-1-phenyl-3-((*R*)-1-phenylallyl)cyclobutan-1-ol (40)............................. - 27 -
4,4,5,5-tetra-Methyl-2-((*1R*,*3r*)-1-(naphthalen-2-yl)-3-((*R*)-1-phenylallyl)cyclobutyl)-1,3,2-dioxaborolane (41) .... - 28 -
(*1s*,*3s*)-3-((*R*)-3-Hydroxy-1-phenylpropyl)-1-(naphthalen-2-yl)cyclobutan-1-ol (42) .... - 28 -
2-((*1s*,*3s*)-1-(Naphthalen-2-yl)-3-((*R*)-1-phenylallyl)cyclobutyl)furan (43) .... - 28 -
2-((*1R*,*3r*)-3-((*R*)-1-Phenylallyl)-1-vinylcyclobutyl)naphthalene (44).............................. - 29 -
(*1s*,*3s*)-3-((*R*)-3-Hydroxy-1-phenylpropyl)-1,3-dimethylcyclobutan-1-ol (S3)................. - 29 -
(*1s*,*3s*)-1-(tert-Butyl)-3-((*R*)-3-hydroxy-1-phenylpropyl)cyclobutan-1-ol (S4)................. - 29 -
(*1s*,*3s*)-1-(3-Bromophenyl)-3-((*R*)-3-hydroxy-1-phenylpropyl)cyclobutan-1-ol (S5) .... - 29 -
3. Computational Details .... - 30 -
3.1. General Information .... - 30 -
3.2. Transition Structures for the Allylation Reaction.. - 31 -
3.3. Rotational Barriers for BCB-Boronate Complexes .... - 34 -
3.4. Reaction of BCB-Boronate Complexes with the Tropylium Ion .... - 36 -
3.5. Thermochemical Data...38
3.6. XYZ coordinates...42
4. Reference 101
5. Spectra 103
5.1 NMR Spectra...103
5.2 HPLC Spectra 146

1. MATERIALS AND GENERAL METHODS

1.1. Glassware, Solvents, and Reagents

All manipulations were performed with oven-dried (130 °C for a minimum of 12 h) or flame-dried glassware using standard Schlenk techniques under an atmosphere of nitrogen, unless otherwise stated.

All anhydrous solvents were commercially supplied or dried using an Anhydrous Engineering alumina column drying system (dichloromethane, toluene, diethyl ether, and tetrahydrofuran). Reagents were purchased from commercial sources and used as received. All organolithium reagents were titrated against *N*-benzylbenzamide.[1]

1.2. Chromatography and Instrumentation

Thin layer chromatography (TLC) was performed using Merck Kieselgel 60 F254 fluorescent treated silica, which was visualised under UV light, or by staining with aqueous basic potassium permanganate followed by heating, or Hanessian's stain (CAM stain) followed by heating, or *p*-anisaldehyde solution followed by heating, as stated.

Flash column chromatography (FCC) was carried out using Sigma-Aldrich silica gel (60 Å, 230-400 mesh, 40-63 μm) or boric acid impregnated silica gel.[2] In cases where automated column chromatography was employed the solvent gradient and flow rate are indicated.

NMR spectra were recorded at various field strengths, as indicated, using Bruker 400 MHz, Varian VNMR 400 MHz for ^{1}H, ^{11}B, and ^{13}C acquisitions. All NMR spectra were recorder at 25 °C unless otherwise stated. Chemical shifts (δ) are reported in parts per million (ppm) and referenced to $CDCl_3$ (^{1}H: 7.26 ppm; ^{13}C: 77.16 ppm). Coupling constants (J) are given in Hertz (Hz) and refer to apparent multiplicities (s = singlet, d = doublet, t = triplet, q = quartet, quin = quintet, hex = hextet, h = heptet, m = multiplet, brs = broad signal, dd = doublet of doublets, etc.). The ^{1}H NMR spectra are reported as follows: chemical shift (multiplicity, coupling constants, number of protons).

HPLC analyses were performed on Agilent 1100 system with Daicel Chiralpak columns.

High resolution mass spectra (HRMS) were recorded on a Bruker Daltonics MicrOTOF II by Electrospray Ionisation (ESI) or a Bruker UltrafleXtreme by Matrix-assisted Laser Desorption/Ionisation (MALDI).

IR spectra were recorded neat as a thin film on a Perkin Elmer Spectrum One FT-IR. Selected absorption maxima (ν_{max}) are reported in wavenumbers (cm^{-1}).

Gas chromatography–mass spectrometry (**GC-MS**) was recorded on an Agilent 6890 Series GC and 5973 detectors using a HP-5MS UI column (15 m x 0.25 mm x 0.25 μm).

1.3. Naming of Compounds

Compound names are those generated by ChemDraw Professional 20.0 software (PerkinElmer), following

the IUPAC nomenclature.

2. EXPERIMENTAL DATA

2.1. Reaction Optimization

Table S1: Reaction Optimization[a]

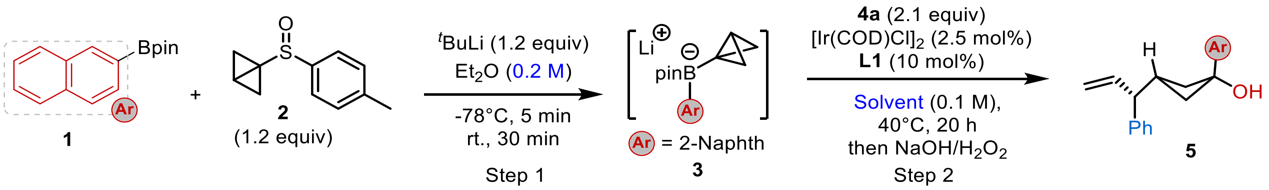

Entry	Solvent	NMR Yield	dr	er
1	toluene	56%	93:7	93.5:6.5
2	*m*-xylene	50%	93:7	95:5
3	THF	55%	83:17	84:16
4	EtOAc	46%	90:10	97:3
5	$HCCl_3$	43%	95:5	90.5:9.5
6	DCM	49%	93:7	96.5:3.5
7[b]	DCM	57%	92:8	96:4
8[b]	DCM/Tol = 1/1	67%	94:6	94.5:4.5
9[b]	DCM/Tol = 5/1	60%	94:6	97:3
10[b]	DCM/Tol = 1/5	66%	93:7	97:3
11[b, c]	DCM/Tol = 1/5	62%	94:6	>99:1
12[b, c, d]	DCM/Tol = 1/5	75%	95:5	99:1

[a] **3** was preformed using **1** (0.2 mmol), **2** (1.2 equiv), ^{t}BuLi (1.2 equiv), Et_2O (0.2 M), -78 °C, 5 min then r.t., 30 min. **4** (2.1 equiv), $[Ir(COD)Cl]_2$ (2.5 mol%) + 10 mol% of the ligand in solvent (0.1 M). The NMR yield was measured by ^{1}H NMR spectroscopy using 1,3,5-trimethoxybenzene as an internal standard; drs and ers were determined by HPLC analysis. [b] Et_2O (0.1 M) in step 1. [c] MeOH (50 μL) was added to quench RLi before solvent change. [d] Et_2O (*c* = 0.067 M) in step 1.

Figure S1: Stoichiometry of ^{t}BuLi

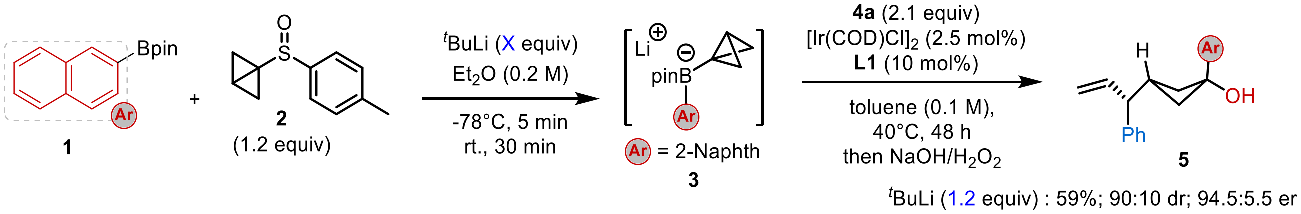

^{t}BuLi (1.2 equiv) : 59%; 90:10 dr; 94.5:5.5 er
^{t}BuLi (1.4 equiv) : 62%; 92:8 dr; 88:12 er

2.2. General Procedures

2.2.1. General Procedure A: Preparation of Bicyclo[1.1.0]butyl Boronate Complexes from BCB-sulfoxide

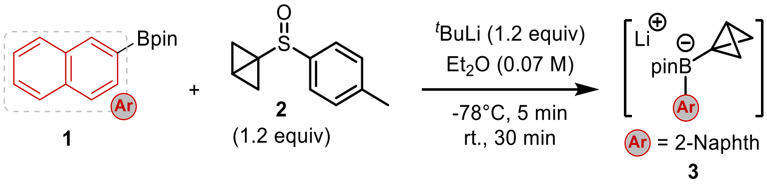

tert-Butyl lithium (in pentane, 0.24 mmol, 1.20 equiv) was added dropwise to a solution of BCB-sulfoxide **2** (47 mg, 0.24 mmol, 1.20 equiv) and the boronic ester **1** (0.20 mmol, 1.0 equiv) in anhydrous diethyl ether (3.0 mL, *c* = 0.067 M) at –78 °C (dry ice/acetone). The reaction mixture was stirred at –78 °C for 5 min. Subsequently, the reaction was removed from the cooling bath and warmed to ambient temperature. After stirring for 30 min, the reaction was analysed by ^{11}B NMR spectrometry to determine whether the bicyclo[1.1.0]butyl boronate complexe was formed.

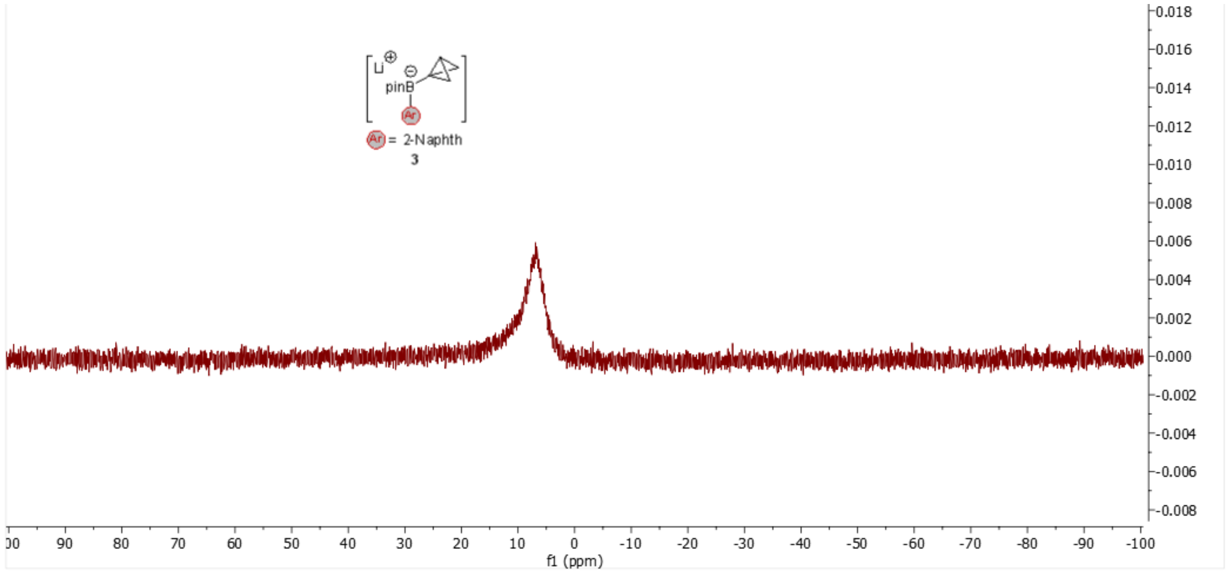

2.2.2. General Procedure B: For the formation of product 5-10, 14-15, 17, 19

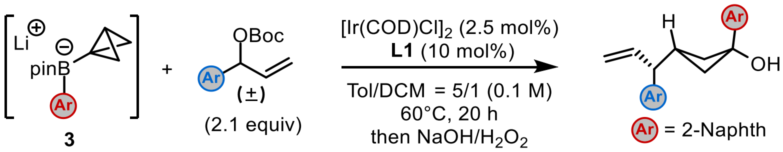

tert-Butyl lithium (in pentane, 0.24 mmol, 1.2 equiv) was added dropwise to a solution of BCB-sulfoxide **5** (47 mg, 0.24 mmol, 1.20 equiv) and the boronic ester **1** (0.20 mmol, 1.0 equiv) in anhydrous ethyl ether (3.0 mL) at –78 °C (dry ice/acetone). The reaction mixture was stirred at –78 °C for 5 min. Subsequently, the reaction was removed from the cooling bath and warmed to ambient temperature. After stirring for 30 min, MeOH (50 μL) was added before the solvent was evaporated carefully in vacuo. A solution of $[Ir(COD)Cl]_2$ (3.4 mg, 2.5 mol%) and **L1** (10.1 mg, 10 mol%) in anhydrous toluene/DCM(5:1, v/v, 1.0 mL) (pre-mixed under N_2 for 30 min) was then added followed by the corresponding allyl carbonate (0.42 mmol, 2.1 equiv). The reaction flask was sealed and heated at 60 °C for 20 h. After cooling to ambient temperature,

the resulting solution was filtered through a silica plug with Et_2O, concentrated in vacuo, and diluted with THF (2.0 mL). The reaction solution was treated with NaOH (3 M aqueous solution, 1 mL) followed by the dropwise addition of H_2O_2 (30% aqueous solution, 1 mL) at 0 °C. It was allowed to stir at room temperature for 3 h, and then quenched with saturated aqueous $Na_2S_2O_3$ (1 mL) at 0 °C. After stirring for 10 min at ambient temperature, the mixture was poured into a separating funnel, and the aqueous phase was extracted with Et_2O (3 x 10 mL). The combined organic layers were washed with brine, dried using Na_2SO_4, filtered, and concentrated in vacuo. The residue was purified by flash column chromatography on silica gel to afford the desired product.

2.2.3. General Procedure C: For the formation of product 11-13, 16, 18, 20-38

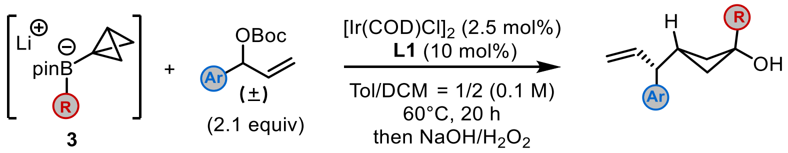

tert-Butyl lithium (in pentane, 0.24 mmol, 1.20 equiv) was added dropwise to a solution of BCB-sulfoxide **5** (47 mg, 0.24 mmol, 1.20 equiv) and the boronic ester (0.20 mmol, 1.0 equiv) in anhydrous ethyl ether (3.0 mL) at –78 °C (dry ice/acetone) and allowed to stir for 5 min. The cooling bath was removed, and the reaction was allowed to warm to ambient temperature and stirred for an additional 30 minutes. Then ^{t}BuOH (50 μL) was added before the solvent was carefully removed under vacuum. A separate 7 mL vial was brought into the glove box and $[Ir(COD)Cl]_2$ (3.4 mg, 2.5 mol%) and **L1** (10.1 mg, 10 mol%) were added. The vial was capped and removed from the glove box. To the vial was added anhydrous Tol/DCM (v/v = 1/2, 1.0 mL). After stirring for 30 minutes at room temperature, the catalyst was then transferred to the reaction vial and the vial was rinsed with anhydrous Tol/DCM (v/v = 1/2, 1.0 mL). The corresponding allyl carbonate (0.42 mmol, 2.1 equiv) was subsequently added to the reaction vial and the reaction was heated to 60 °C for 20 hours. The resulting mixture was cooled to room temperature, filtered through a silica plug with diethyl ether, and concentrated under reduced pressure. The crude oil was diluted with THF (2.0 mL), cooled to 0 °C, and 3M NaOH (1.0 mL), followed by 38% H_2O_2 (1.0 mL) was added, dropwise. The reaction mixture was allowed to warm to room temperature and stirred for an additional 3 hours. The resulting mixture was again cooled to 0 °C and saturated aqueous $Na_2S_2O_3$ (1 mL) was added dropwise. The reaction mixture was allowed to warm to room temperature and the aqueous layer was extracted with diethyl ether (3 x 10 mL). The combined organic layers were washed with brine, dried with Na_2SO_4, filtered, and concentrated in vacuo. The crude product was purified by flash column chromatography on silica gel to afford the desired product.

2.2.4. General Procedure D: For the formation of product 39, 40

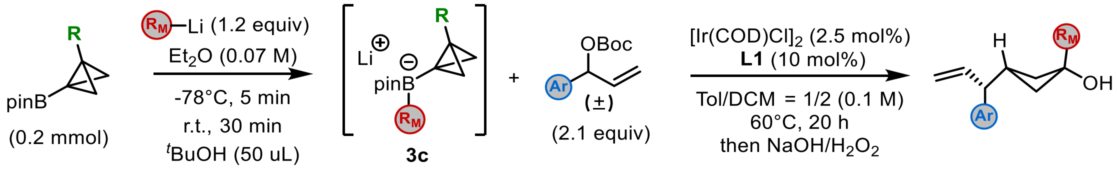

R_M lithium (0.24 mmol, 1.20 equiv) was added dropwise to a solution of BCB boronic ester (0.20 mmol, 1.0 equiv) in anhydrous diethyl ether (3.0 mL) at –78°C (dry ice/acetone) and allowed to stir for 5 min. The cooling bath was removed, and the reaction was allowed to warm to ambient temperature and stirred for an additional 30 minutes. Then ^{t}BuOH (50 μL) was added before the solvent was carefully removed under vacuum. A separate 7 mL vial was brought into the glove box and $[Ir(COD)Cl]_2$ (3.4 mg, 2.5 mol%) and **L1** (10.1 mg, 10 mol%) were added. The vial was capped and removed from the glove box. To the vial was added anhydrous Tol/DCM (v/v = 1/2, 1.0 mL). After stirring for 30 minutes at room temperature, the catalyst was then transferred to the reaction vial and the vial was rinsed with anhydrous Tol/DCM (v/v = 1/2, 1.0 mL). The corresponding allyl carbonate (0.42 mmol, 2.1 equiv) was subsequently added to the reaction vial and the reaction was heated to 60℃ for 20 hours. The resulting mixture was cooled to room temperature, filtered through a silica plug with diethyl ether, and concentrated under reduced pressure. The crude oil was diluted with THF (2.0 mL), cooled to 0℃, and 3M NaOH (1.0 mL), followed by 38% H_2O_2 (1.0 mL) was

added, dropwise. The reaction mixture was allowed to warm to room temperature and stirred for an additional 3 hours. The resulting mixture was again cooled to 0℃ and saturated aqueous $Na_2S_2O_3$ (2 mL) was added dropwise. The reaction mixture was allowed to warm to room temperature and the aqueous layer was extracted with diethyl ether (3 x 10 mL). The combined organic layers were washed with brine, dried using Na_2SO_4, filtered, and concentrated in vacuo. The crude product was purified by flash column chromatography on silica gel to afford the desired product.

2.2.5. Procedure E: For gram-scale reaction with the formation of product 41

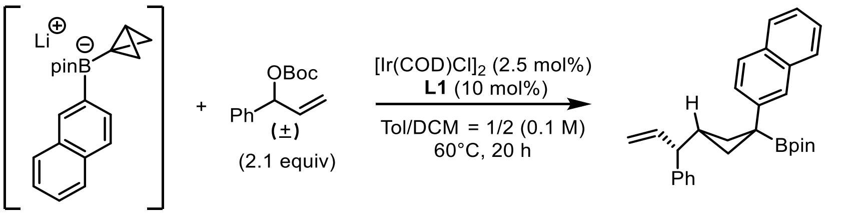

tert-Butyl lithium (in pentane, 4.8 mmol, 1.20 equiv) was added dropwise to a solution of BCB-sulfoxide **5** (0.92 g, 4.8 mmol, 1.20 equiv) and the boronic ester (4.00 mmol, 1.02 g, 1.00 equiv) in anhydrous ethyl ether (60 mL) at –78°C (dry ice/acetone) and allowed to stir for 5 min. The cooling bath was removed, and the reaction was allowed to warm to ambient temperature and stirred for an additional 30 minutes. Then ^{t}BuOH (1.0 mL) was added before the solvent was carefully removed under vacuum. A separate 25 mL vial was brought into the glove box and $[Ir(COD)Cl]_2$ (0.10 mmol, 67.2 mg, 2.5 mol%) and **L1** (0.40 mmol, 202.9 mg, 10 mol%) were added. The vial was capped and removed from the glove box. To the vial was added anhydrous Tol/DCM (v/v = 1/2, 20 mL). After stirring for 30 minutes at room temperature, the catalyst was then transferred to the reaction vial and the vial was rinsed with anhydrous Tol/DCM (v/v = 1/2, 20 mL). The corresponding allyl carbonate (8.4 mmol, 1.97g, 2.10 equiv) was subsequently added to the reaction vial and the reaction was heated to 60℃ for 20 hours. The resulting mixture was cooled to room temperature, filtered through a silica plug with diethyl ether, and concentrated under reduced pressure. The crude product was purified by flash column chromatography (hexane: diethyl ether = 40:1) on silica gel to afford the desired product (1.28 g, 75% yield).

2.3. Procedures for transformation of products

2.3.1. Procedure F: For the formation of product 5 from product 41

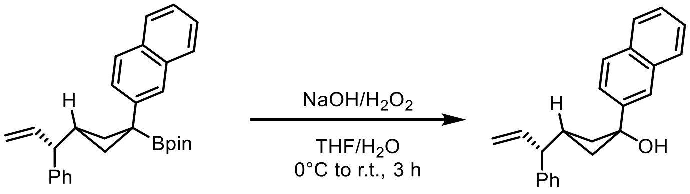

Boronic ester **41** (35 mg, 0.082 mmol, 1.00 equiv) was dissolved in THF (2 mL) and cooled to 0°C (ice/water). NaOH (3 M aqueous solution, 0.5 mL) was added dropwise followed by H_2O_2 (30% aqueous solution, 0.5 mL) dropwise addition to the vigorously stirring reaction mixture, which was subsequently warmed to room temperature and allowed to stir for an additional 3 hours. The resulting mixture was again cooled to 0℃ and saturated aqueous $Na_2S_2O_3$ (2 mL) was added dropwise. The reaction mixture was again allowed to warm to room temperature and the aqueous layer was extracted with ethyl acetate (3 x 10 mL). The combined organic layers were washed with brine, dried with Na_2SO_4, filtered, and concentrated in vacuo. The crude product was purified by flash column chromatography on silica gel (hexane: EtOAc, 95:5) to afford **5** as a colorless oil (28.9 mg, 92% yield).

2.3.2. Procedure G: For the formation of product 42 from product 41

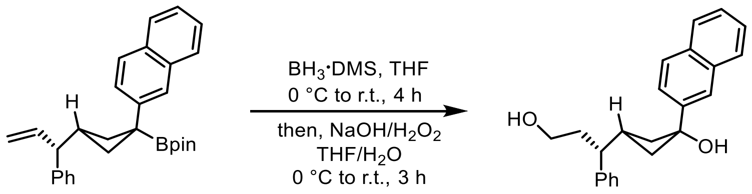

To a solution of boronic ester 41 (29 mg, 0.067 mmol, 1.00 equiv) in THF (0.7 mL) was added a 2M solution of BH_3·DMS in diethyl ether (67 mL, 0.14 mmol, 2.0 equiv) dropwise at 0℃ over 5 minutes. The reaction mixture was subsequently warmed to room temperature and stirred for an additional 4 hours. The reaction mixture was again cooled to 0℃ and a 3M solution of NaOH (0.5 mL) was added followed by 30% H_2O_2 (0.5 mL) dropwise, then the mixture was warmed to room temperature to stir for an additional 3 hours. After completion, the reaction was again cooled to 0℃ and a sat. aqueous solution of $Na_2S_2O_3$ (1.0 mL) was added dropwise before warming the mixture to room temperature. The biphasic solution was diluted with EtOAc (5.0 mL) and then the aqueous layer was extracted with EtOAc (3 x 10 mL). The combined organic layers were washed with brine, dried with Na_2SO_4, filtered, and concentrated under reduced pressure. The crude product was purified via flash column chromatography on silica gel (hexanes: EtOAc, 1:1) to afford 42 as a a colorless oil (18.4 mg, 83% yield).

2.3.3. Procedure H: For the formation of product 43 from product 41

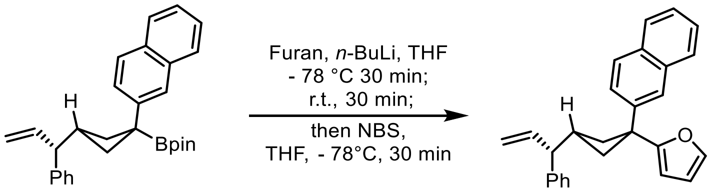

n-BuLi (175 µL, 0.26 mmol, 1.49 M in Hexanes) was added dropwise to a solution of furan (22 µL, 0.26 mmol, 1.30 equiv) in anhydrous THF (1 mL) at –78 °C and stirred for 30 min before warming to room temperature and stirring for an additional 30 min. The reaction was again cooled to –78°C and boronic ester **41** (85 mg, 0.2 mmol, 1.0 equiv) in THF (1.0 mL) was added slowly dropwise. The reaction was allowed to stir at this temperature for 5 min before warming to ambient temperature for 15 min to allow for complete ate complex formation. Following this, the reaction was re-cooled to −78 ° C once more and N-bromosuccinimide (46 mg, 0.26 mmol, 1.3 equiv) in THF (1.0 mL) was added dropwise slowly to the reaction mixture. The reaction was then left to stir for 1 hour at this temperature before saturated aqueous $Na_2S_2O_3$ (1 mL) was added, and the reaction was warmed to room temperature before extracting with Et_2O (3 × 10 mL). The combined organic phases were then dried with Na_2SO_4, filtered and concentrated under vacuo. The crude product was purified by flash column chromatography on silica gel (hexanes: EtOAc, 98:2) to afford the product **43** as a colorless oil (56.9 mg, 78% yield).

2.3.4. Procedure I: For the formation of product 44 from product 41

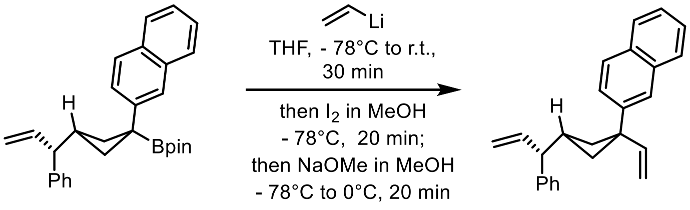

n-BuLi (1.5 M in hexanes, 0.27 mL, 0.40 mmol, 2.0 equiv) was added dropwise to neat tetravinyltin (37 μL, 0.20 mmol, 1.0 equiv) at ambient temperature. The reaction mixture was stirred at this temperature for 30 min, during which time a white precipitate of vinyl lithium formed. Anhydrous THF (1.0 mL) was added and a homogeneous solution formed which was then added dropwise to a solution of boronic ester **41** (85 mg, 0.20 mmol, 1.0 equiv) in 1.0 mL anhydrous THF at –78°C. The resulting solution was then stirred at –78°C for 15 min then warmed to 0°C and stirred for a further 15 min. The reaction was again cooled to –78°C and a suspension of NaOMe (0.80 mmol, 4.0 equiv) in MeOH (0.5 mL x 2) was added in one portion followed by dropwise addition of a solution of iodine (61 mg, 0.24 mmol, 1.2 equiv) in MeOH (1 mL) before stirring at –78°C for 20 min. The reaction was slowly warmed to 0°C and stirred for an additional 30 min. Saturated aqueous $Na_2S_2O_3$ solution (2 mL) was then added, followed by DCM (5 mL) and the resulting biphasic mixture was filtered through a short pad of Celite to facilitate extraction. The organic phase was separated, and aqueous phase extracted with DCM (3× 10 mL), the combined organic phases were dried ($MgSO_4$), filtered and concentrated. The crude product was purified by flash column chromatography on silica gel (hexane: Et_2O = 40:1) to afford **44** as a colorless oil (54.1 mg, 84% yield).

2.3.5. Procedure J: For hydroboration-oxidation of products

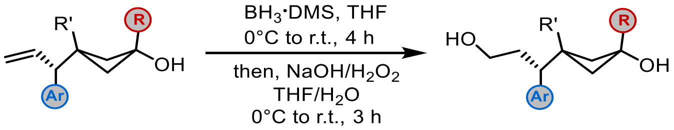

To a solution of corresponding alcohol (1.0 equiv) in THF (0.1 M) was added a 2M solution of BH_3·DMS in diethyl ether (1.5 equiv) dropwise at 0℃ over 5 minutes. The reaction mixture was subsequently warmed to room temperature and stirred for an additional 4 hours. The reaction mixture was again cooled to 0℃ and a 3M solution of NaOH (0.5 mL/0.05 mmol) was added followed by 30% H_2O_2 (0.5 mL/0.05 mmol) dropwise, then the mixture was warmed to room temperature to stir for an additional 3 hours. After completion, the reaction was again cooled to 0℃ and saturated aqueous $Na_2S_2O_3$ soulution (1.0 mL/0.01 mmol) was added dropwise before warming the mixture to room temperature. The biphasic solution was diluted with EtOAc (5.0 mL/0.05 mmol) and then the aqueous layer was extracted with EtOAc (3 x 10 mL/0.1 mmol). The combined organic layers were washed with brine, dried with Na_2SO_4, filtered, and concentrated *in vacuum*. The crude product was purified via flash column chromatography on silica gel (hexanes: EtOAc, 1:1) to afford the corresponding product.

2.4 Unsuccessful Examples

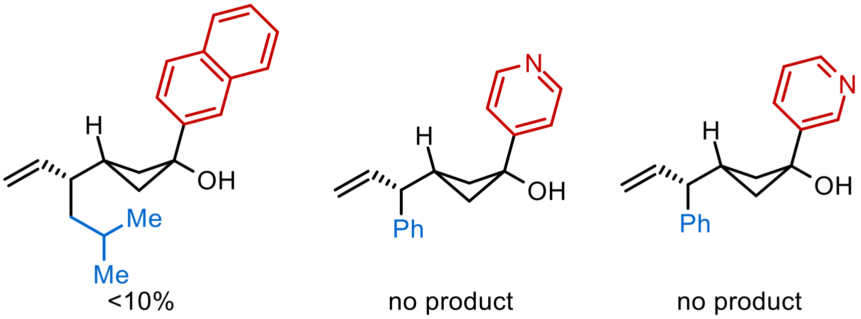

2.5. Procedures for mechanistic studies

2.5.1. Kinetic resolution of racemic carbonate

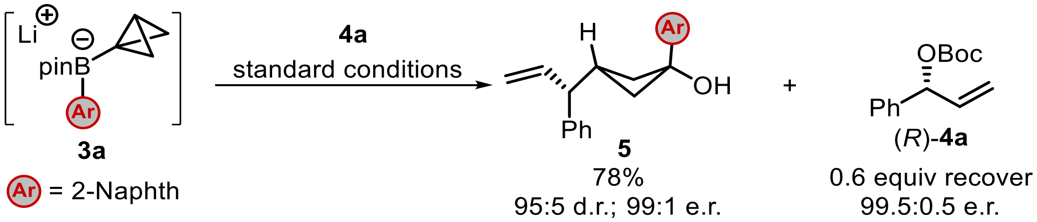

Reaction was performed according to **General Procedure B** using **4a** and the crude product was subjected to flash column chromatography on silica gel (hexanes: EtOAc, 95:5) for recovery of (*R*)-**4a** as a colorless oil (~28% yield; ~0.59 equiv) and er was analyzed compared to *rac*-**4a**.

rac-**4a**

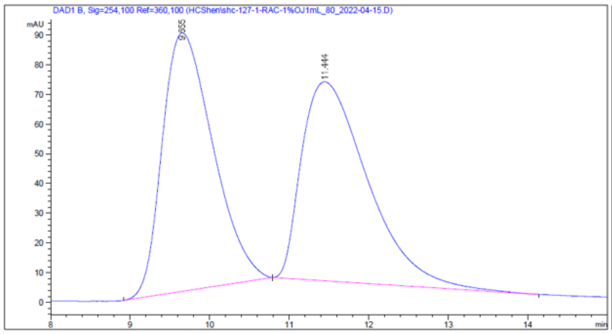

Peak #	RetTime [min]	Type	Width [min]	Area [mAU*s]	Height [mAU]	Area %
1	9.655	BB	0.6067	3847.40869	86.96015	49.8545
2	11.444	BB	0.6846	3869.86670	66.97660	50.1455

(*R*)-**4a**

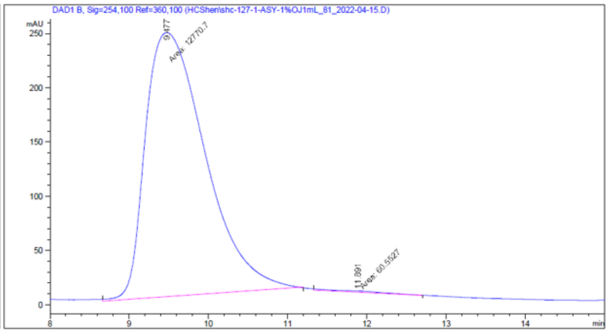

Peak #	RetTime [min]	Type	Width [min]	Area [mAU*s]	Height [mAU]	Area %
1	9.477	MM	0.6140	1.27707e4	243.37236	99.5281
2	11.891	MM	0.7884	60.55275	1.28015	0.4719

2.5.2. Nucleophilicity comparison between alkenylboronate complex 45 and BCB boronate complex 3e

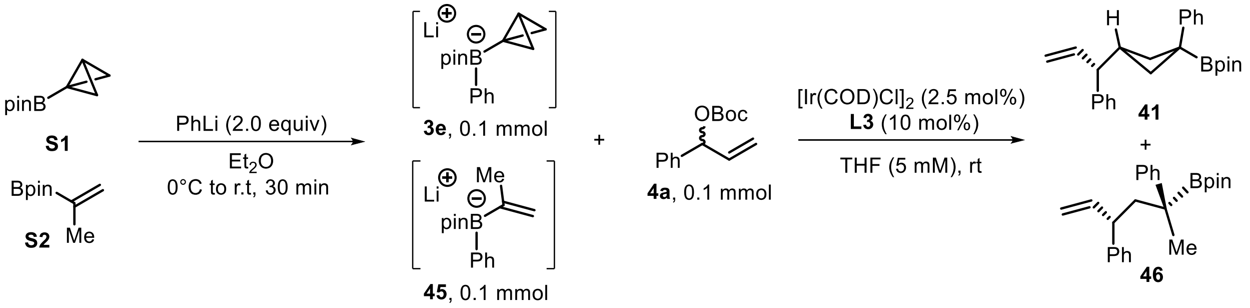

Phenyl lithium (0.200 mmol, 108 μL, 2.00 equiv) was added dropwise to a solution of BCB boronic ester **S1** (0.10 mmol, 19.3 μL, 1.0 equiv) and alkenyl boronic ester (0.10 mmol, 18.7 μL, 1.0 equiv) **S2** in anhydrous ethyl ether (3.0 mL) at –78°C (dry ice/acetone) and allowed to stir for 5 min. The cooling bath was removed, and the reaction was allowed to warm to ambient temperature and stirred for an additional 30 minutes. Then the solvent was carefully removed under vacuum and redissolved in anhydrous THF (18 mL). A separate 7 mL vial was brought into the glove box and $[Ir(COD)Cl]_2$ (1.7 mg; 2.5 mol%) and **L3** (4.1 mg, 10 mol%) were added. The vial was capped and removed from the glove box. To the vial was added anhydrous THF (1.0 mL). After stirring for 30 minutes at room temperature, the catalyst was then transferred to the

reaction vial and the vial was rinsed with anhydrous THF (1.0 mL). The corresponding allyl carbonate (0.10 mmol, 1.0 equiv) and internal standard hexadecane (20 μL; 0.065 mmol) were subsequently added to the reaction vial, and the reaction was stirred at ambient temperature and monitored by GC analysis. The GC yields of products 41 and 46 were shown in the next table.

Entry	Time	GC yield of 41	GC yield of 46
1	1 min	1.3%	0.1%
2	2 min	2.6%	0.1%
3	3 min	4.0%	0.2%
4	4 min	5.0%	0.3%
5	6 min	6.8%	0.5%
6	10 min	9.2%	0.64%
7[b]	20 min	16%	1%
8[b]	14 h	35%	6%

2.5.3. Table S2. Substituent effects for diastereoselectivity

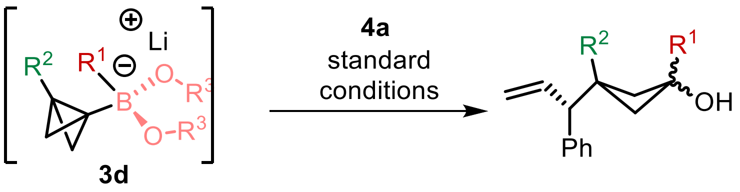

Entry	R^1	R^2	$B(OR^3)_2$	d.r.
1	Me	H	Bpin	90:10
2	Ph	H	Bpin	86:14
3	Cy	H	Bpin	63:37
4	Me	Me	Bpin	94:6
5	Ph	Me	Bpin	>95:5
6	Ph	H	Bgly	44:56
7	Cy	H	Bgly	50:50
8	Ph	H	Bneo	78:22
9	Ph	H	Bmac	62:38

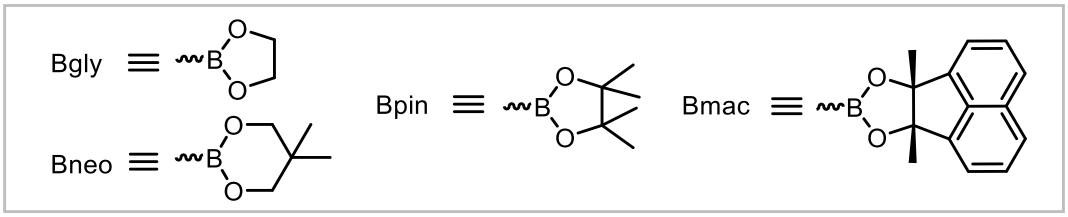

Reactions were performed according to **General Procedure B** (entry 2)**, General Procedure C** (entries 1, 3, 6-9) and **General Procedure D** (entries 4-5). The d.r. was determined by HPLC analysis.

2.6. Synthesis of Starting Materials

2.6.1. Synthesis of racemic allylic carbonates

All racemic allylic carbonates were synthesized according to relevant literatures.[2-3]

2.6.2. Synthesis of bicyclo[1.1.0]butyl sulfoxide

The bicyclo[1.1.0]butyl sulfoxide was synthesized according to our previous work.[4]

2.6.3. Table S3. Synthesis of noncommercially available boronic esters used in this project.

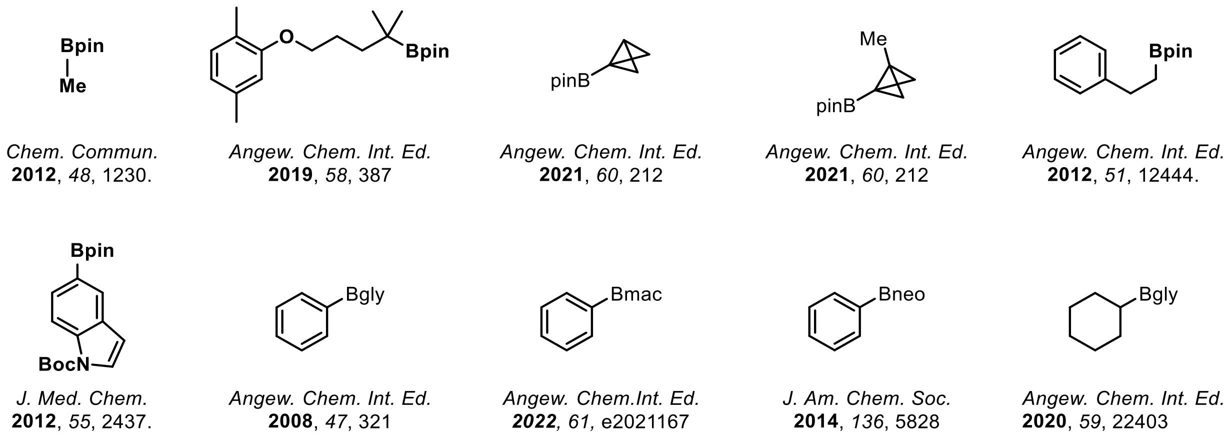

Scheme S1. Previously Reported Boronic Ester Substrates

2.7. Crystallography

X-ray diffraction experiments on **41** were carried out at 100(2) K on a Bruker D8 Venture diffractometer using Cu-K_{α} radiation (λ = 1.54178 Å). Data collection was performed using a Bruker CPAD detector. Intensities were integrated in SAINT[5] and absorption corrections based on equivalent reflections were applied using SADABS.[6] The structure was solved using ShelXT[7] and refined by full matrix least squares against F^2 in ShelXL[8, 9] using Olex2.[10] All of the non-hydrogen atoms were refined anisotropically. While all the hydrogen atoms were located geometrically and refined using a riding model. The molecule displayed disorder and was partially modelled in two positions with refined occupancies, SADI and SIMU were used to maintain sensible geometries and thermal parameters. Absolute structure was not determined, crystal quality was poor and the error on the Flack parameter was too large, hence has been removed from the cif. The crystal structure and refinement data are given in Table S4. Crystallographic data for compound **41** has been deposited with the Cambridge Crystallographic Data Centre as supplementary publication CCDC 2208081. Copies of the data can be obtained free of charge on application to CCDC, 12 Union Road, Cambridge CB2 1EZ, UK [fax(+44) 1223 336033, e-mail: deposit@ccdc.cam.ac.uk].

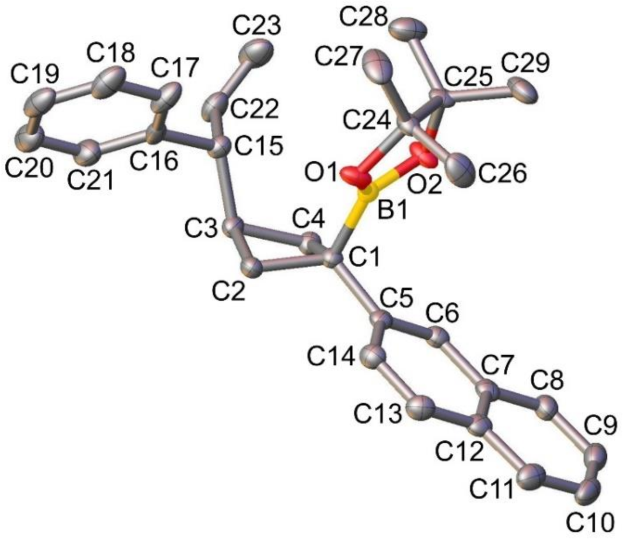

Figure S2. Crystal structure of **41** with the anisotropic displacement parameters depicted at the 50% probability level. Disorder and hydrogens omitted for clarity.

Table S4. Crystal data and structure refinement for **41**.

CCDC Number	2208081
Empirical formula	$C_{29}H_{33}BO_2$
Formula weight	424.36
Temperature/K	100.00
Crystal system	orthorhombic
Space group	$C222_1$
a/Å	12.3042(5)
b/Å	13.0643(5)
c/Å	30.6975(13)
α/°	90
β/°	90
γ/°	90
Volume/Å^3	4934.5(3)
Z	8
ρ_{calc}g/cm^3	1.142
μ/mm^{-1}	0.531
F(000)	1824.0
Crystal size/mm^3	0.329 × 0.32 × 0.12
Radiation	CuKα (λ = 1.54178)
2Θ range for data collection/°	5.758 to 144.176
Index ranges	-15 ≤ h ≤ 15, -16 ≤ k ≤ 16, -37 ≤ l ≤ 37
Reflections collected	56843
Independent reflections	4854 [R_{int} = 0.0307, R_{sigma} = 0.0155]
Data/restraints/parameters	4854/449/401
Goodness-of-fit on F^2	1.078
Final R indexes [I>=2σ (I)]	R_1 = 0.0359, wR_2 = 0.0943
Final R indexes [all data]	R_1 = 0.0359, wR_2 = 0.0943
Largest diff. peak/hole / e Å^{-3}	0.28/-0.23

2.8. Characterization Data

(*1s*,*3s*)-1-(Naphthalen-2-yl)-3-((*R*)-1-phenylallyl)cyclobutan-1-ol (5)

Prepared following **General Procedure B.** Purification by flash column chromatography (5:1:1 hexane/EtOAc/DCM) gave the title compound (49.0 mg, 78%, 95:5 dr, 99:1 er) as a colorless oil.

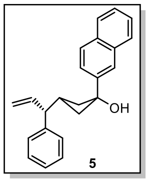

5

^{1}H NMR (400 MHz, $CDCl_3$): δ_H 7.91 (d, *J* = 1.9 Hz, 1H), 7.90 – 7.79 (m, 3H), 7.64 (dd, *J* = 8.6, 1.9 Hz, 1H), 7.54 – 7.44 (m, 2H), 7.33-7.28 (m, 2H), 7.26 – 7.16 (m, 3H), 6.02 – 5.88 (m, 1H), 5.14 – 5.06 (m, 1H), 5.07 (d, *J* = 1.1 Hz, 1H), 3.39 – 3.35 (m, 1H), 3.00 – 2.86 (m, 1H), 2.63 (m, 1H), 2.36 – 2.22 (m, 2H), 2.20 – 2.12 (m, 1H), 2.10 (s, 1H) ppm. **^{13}C NMR** (101 MHz, $CDCl_3$): δ_C 143.1, 142.5, 139.9, 133.2, 132.8, 128.8, 128.6, 128.3, 128.0, 127.7, 126.6, 126.4, 126.2, 124.4, 123.4, 115.1, 72.8, 57.2, 42.3, 41.8, 29.9 ppm. **Specific rotation** $[\alpha]_D^{22}$ = +59 (c = 0.68, CH_2Cl_2). **HRMS** (ESI) m/z calculated for $C_{23}H_{21}$ $[M\text{-}H_2O\text{+}H]^+$, 297.1638; found, 297.1647**. IR (thin film)** v_{max}/cm^{-1}: 3344, 2973, 1600, 1492, 1144, 914, 857, 700. **HPLC conditions**: Chiral column IC, hexane: isopropanol = 97:3, flow rate = 0.8 mL/min, wavelength = 254 nm, t_R = 21.4 min for major isomer, t_R = 19.2 min for minor isomer.

(*1s*,*3s*)-3-((*R*)-1-(4-Methoxyphenyl)allyl)-1-(naphthalen-2-yl)cyclobutan-1-ol (6)

Prepared following **General Procedure B.** Purification by flash column chromatography (5:1:1 hexane/EtOAc/DCM) gave the title compound (44.8 mg, 65%, 90:10 dr, >99:1 er) as a colorless oil.

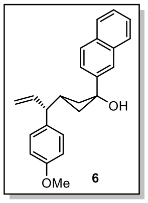

6

^{1}H NMR (400 MHz, $CDCl_3$): δ_H 7.91 (d, *J* = 1.9 Hz, 1H), 7.90 – 7.80 (m, 3H), 7.65 (dd, *J* = 8.6, 1.9 Hz, 1H), 7.53 – 7.46 (m, 2H), 7.15 – 7.07 (m, 2H), 6.88 – 6.83 (m, 2H), 5.99 – 5.85 (m, 1H), 5.09 – 5.07 (m, 1H), 5.06– 5.04 (m, 1H), 3.79 (s, 3H), 3.32 (dd, *J* = 9.1, 7.5 Hz, 1H), 2.99 – 2.85 (m, 1H), 2.6-2.61 (m, 1H), 2.33 – 2.18 (m, 2H), 2.19 – 2.09 (m, 2H) ppm. **^{13}C NMR** (101 MHz, $CDCl_3$): δ_C 158.3, 143.1, 140.1, 134.6, 133.1, 132.8, 128.8, 128.8, 128.3, 127.7, 126.4, 126.2, 124.4, 123.4, 114.8, 114.0, 72.8, 56.3, 55.4, 42.3, 41.9, 30.0 ppm. **HRMS** (ESI) m/z calculated for $C_{24}H_{22}O$ $[M\text{-}H_2O\text{+}H]^+$, 327.1743; found, 327.1744. **IR (thin film)** v_{max}/cm^{-1}: 3364, 2971, 2930, 1609, 1510, 1243, 1035, 749. **HPLC conditions**: Chiral column IA, hexane: isopropanol = 95:5, flow rate = 1 mL/min, wavelength = 254 nm, t_R = 38.1 min for major isomer.

(*1s*,*3s*)-1-(Naphthalen-2-yl)-3-((*R*)-1-(p-tolyl)allyl)cyclobutan-1-ol (7)

Prepared following **General Procedure B.** Purification by flash column chromatography (5:1:1 hexane/EtOAc/DCM) gave the title compound (47.3 mg, 72%, 91:9 dr, >99:1 er) as a colorless oil.

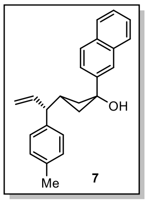

7

^{1}H NMR (400 MHz, $CDCl_3$): δ_H 7.91 (d, *J* = 1.9 Hz, 1H), 7.90 – 7.80 (m, 3H), 7.65 (dd, *J* = 8.6, 1.9 Hz, 1H), 7.53 – 7.45 (m, 2H), 7.14 – 7.06 (m, 4H), 5.93 (ddd, *J* = 17.0, 10.4, 7.6 Hz, 1H), 5.12 – 5.05 (m, 1H), 5.08 – 5.02 (m, 1H), 3.37 – 3.29 (m, 1H), 2.99 – 2.86 (m, 1H), 2.67-2.62 (m, 1H), 2.33 (s, 3H), 2.33 – 2.21 (m, 2H), 2.20 – 2.10 (m, 1H), 1.98 (s, 2H) ppm. **^{13}C NMR** (101 MHz, $CDCl_3$): δ_C 143.1, 140.1, 139.5, 136.1, 133.2, 132.8, 129.3, 128.8, 128.3, 127.8, 127.7, 126.4, 126.2, 124.4, 123.4, 114.9, 72.8, 56.8, 42.3, 41.9, 29.9, 21.2 ppm. **HRMS** (ESI) m/z calculated for $C_{24}H_{23}$ $[M\text{-}H_2O\text{+}H]^+$, 311.1794; found, 311.1796. **IR (thin film)** v_{max}/cm^{-1}: 3349, 2974, 2925, 1635, 1512, 1137, 815, 749. **HPLC conditions**: Chiral column IA, hexane: isopropanol = 95:5, flow rate = 1 mL/min, wavelength = 254 nm, t_R = 25.6 min for major isomer.

(*1s*,*3s*)-3-((*R*)-1-([1,1'-Biphenyl]-4-yl)allyl)-1-(naphthalen-2-yl)cyclobutan-1-ol (8)

Prepared following **General Procedure B.** Purification by flash column chromatography (5:1:1 hexane/EtOAc/DCM) gave the title compound (49.2 mg, 63%, 94:6 dr, 98.5:1.5 er) as a colorless oil.

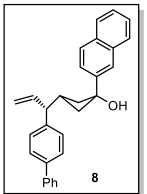
8

^{1}H NMR (400 MHz, $CDCl_3$): δ_H 7.83 (d, *J* = 1.9 Hz, 1H), 7.81 – 7.71 (m, 3H), 7.56 (dd, J = 8.6, 1.9 Hz, 1H), 7.53 – 7.44 (m, 2H), 7.46 – 7.42 (m, 2H), 7.43 – 7.36 (m, 2H), 7.38 – 7.29 (m, 2H), 7.28 – 7.19 (m, 1H), 7.20 – 7.13 (m, 2H), 5.88 (ddd, J = 17.1, 10.3, 7.7 Hz, 1H), 5.08 – 5.00 (m, 1H), 5.04 – 4.97 (m, 1H), 3.36 – 3.26 (m, 1H), 2.92 – 2.79 (m, 1H), 2.59 (ddd, J = 11.6, 6.7, 4.8 Hz, 1H), 2.28 – 2.16 (m, 2H), 2.14 – 2.05 (m, 1H), 2.06 (s, 1H) ppm. **^{13}C NMR** (101 MHz, $CDCl_3$): δ_C 143.1, 141.6, 141.1, 139.8, 139.5, 133.2, 132.8, 128.9, 128.8, 128.3, 128.3, 127.7, 127.3, 127.2, 127.1, 126.4, 126.2, 124.4, 123.4, 115.3, 72.8, 56.9, 42.3, 41.9, 29.9 ppm. **HRMS** (ESI) m/z calculated for $C_{29}H_{25}$ [M-H_2O+H]$^+$, 373.1951; found, 373.1969. **IR (thin film)** ν_{max}/cm^{-1}: 3347, 3055, 2973, 2928, 1634, 1600, 1485, 765. **HPLC conditions**: Chiral column IC, hexane: isopropanol = 97:3, flow rate = 1 mL/min, wavelength = 254 nm, t_R = 27.7 min for major isomer, t_R = 22.1 min for minor isomer.

(*1s*,*3s*)-3-((*R*)-1-(4-Chlorophenyl)allyl)-1-(naphthalen-2-yl)cyclobutan-1-ol (9)

Prepared following **General Procedure B.** Purification by flash column chromatography (5:1:1 hexane/EtOAc/DCM) gave the title compound (48.1 mg, 69%, 92:8 dr, 98.5:1.5 er) as a colorless oil.

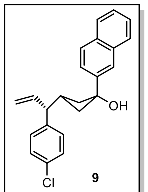
9

^{1}H NMR (400 MHz, $CDCl_3$): δ_H 7.84 – 7.72 (m, 4H), 7.55 (dd, *J* = 8.6, 1.9 Hz, 1H), 7.45 – 7.38 (m, 2H), 7.19 (m, 2H), 7.07 – 7.00 (m, 2H), 5.82 (m, 1H), 5.06-5.02 (m, 1H), 5.02 – 4.94 (m, 1H), 3.31 – 3.20 (m, 1H), 2.90 – 2.78 (m, 1H), 2.54 (m, 1H), 2.25 – 2.07 (m, 2H), 2.08 – 1.98 (m, 1H), 1.84 (s, 1H) ppm. **^{13}C NMR** (101 MHz, $CDCl_3$): δ_C 142.9, 141.0, 139.3, 133.1, 132.9, 132.3, 129.3, 128.9, 128.8, 128.3, 127.7, 126.5, 126.3, 124.3, 123.4, 115.5, 72.8, 56.5, 42.2, 41.7, 29.9 ppm. **Specific rotation** $[\alpha]_D^{22}$ = +83 (c = 0.41, CH_2Cl_2). **HRMS** (ESI) m/z calculated for $C_{23}H_{20}Cl$ [M-H_2O+H]$^+$, 331.1248; found, 331.1255. **IR (thin film)** ν_{max}/cm^{-1}: 3338, 2974, 2928, 1634, 1490, 1241, 819, 749. **HPLC conditions**: Chiral column IC, hexane: isopropanol = 97:3, flow rate = 0.8 mL/min, wavelength = 254 nm, t_R = 21.1 min for major isomer, t_R = 19.2 min for minor isomer.

(*1s*,*3s*)-3-((*R*)-1-(4-Bromophenyl)allyl)-1-(naphthalen-2-yl)cyclobutan-1-ol (10)

Prepared following **General Procedure B.** Purification by flash column chromatography (5:1:1 hexane/EtOAc/DCM) gave the title compound (48.6 mg, 62%, 92:8 dr, 98.5:1.5 er) as a colorless oil.

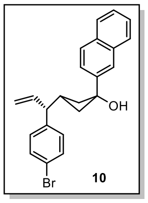
10

^{1}H NMR (400 MHz, $CDCl_3$): δ_H 7.92 – 7.80 (m, 4H), 7.63 (dd, *J* = 8.6, 1.9 Hz, 1H), 7.53 – 7.46 (m, 2H), 7.44 – 7.40 (m, 2H), 7.10 – 7.02 (m, 2H), 5.89 (ddd, *J* = 17.0, 10.4, 7.5 Hz, 1H), 5.13 – 5.06 (m, 1H), 5.10 – 5.02 (m, 1H), 3.40 – 3.26 (m, 1H), 3.00 – 2.85 (m, 1H), 2.62 (ddd, *J* = 11.8, 7.1, 5.1 Hz, 1H), 2.32 – 2.16 (m, 2H), 2.16 – 2.08 (m, 1H) ppm. **^{13}C NMR** (101 MHz, $CDCl_3$): δ_C 143.0, 141.5, 139.2, 133.1, 132.9, 131.7, 129.7, 128.9, 128.3, 127.7, 126.5, 126.3, 124.3, 123.4, 120.3, 115.6, 72.8, 56.5, 42.1, 41.6, 29.9 ppm. **Specific rotation** $[\alpha]_D^{22}$ = +79 (c = 0.79, CH_2Cl_2). **HRMS** (ESI) m/z calculated for $C_{23}H_{20}Br$ [M-H_2O+H]$^+$, 375.0743; found, 375.0750. **IR (thin film)** ν_{max}/cm^{-1}: 3339, 2974, 2928, 1634, 1487, 1010, 817, 748. **HPLC conditions**: Chiral column IC, hexane: isopropanol = 95:5, flow rate = 1 mL/min, wavelength = 254 nm, t_R = 13.4 min for major isomer, t_R = 12.1 min for minor isomer.

(*1s*,*3s*)-1-(Naphthalen-2-yl)-3-((*R*)-1-(4-(trifluoromethyl)phenyl)allyl)cyclobutan-1-ol (11)

Prepared following **General Procedure B.** Purification by flash column chromatography (5:1:1 hexane/EtOAc/DCM) gave the title compound (43.6 mg, 57%, 92:8 dr, 97:3 er) as a colorless oil.

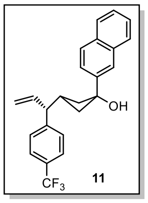
11

^{1}H NMR (400 MHz, $CDCl_3$): δ_H 7.90 (d, *J* = 1.9 Hz, 1H), 7.91 – 7.80 (m, 3H), 7.63 (dd, *J* = 8.6, 1.9 Hz, 1H), 7.59 – 7.52 (m, 2H), 7.53 – 7.46 (m, 2H), 7.30 (d, *J* = 8.0 Hz, 2H), 5.92 (ddd, *J* = 17.0, 10.4, 7.6 Hz, 1H), 5.14 – 5.12 (m, 1H), 5.11 – 5.08 (m, 1H), 3.47 – 3.42 (m, 1H), 3.03 – 2.87 (m, 1H), 2.65 – 2.59 (m, 1H), 2.35 – 2.24 (m, 2H), 2.18 – 2.08 (m, 1H), 1.93 (s, 1H) ppm. **^{13}C NMR** (101 MHz, $CDCl_3$): δ_C 146.7, 142.8, 138.9, 133.1, 132.9, 129.0 (q, *J* = 16.0 Hz), 128.3, 128.3, 127.7, 126.5, 126.3, 125.8 (q, *J* = 270 Hz), 125.6 (q, *J* = 3.8 Hz), 124.3, 123.4, 116.0, 72.8, 57.0, 42.1, 41.6, 29.9 ppm. **Specific rotation** $[\alpha]_D^{22}$ = +58 (c = 0.76, CH_2Cl_2). **HRMS** (ESI) m/z calculated for $C_{24}H_{20}F_3$ $[M-H_2O+H]^+$, 365.1512; found, 365.1526. **IR (thin film)** v_{max}/cm^{-1}: 3335, 2976, 2931, 1617, 1325, 1121, 748. **HPLC conditions**: Chiral column IA, hexane: isopropanol = 90:10, flow rate = 1 mL/min, wavelength = 254 nm, t_R = 12.3 min for major isomer, t_R = 13.4 min for minor isomer.

(*1s*,*3s*)-3-((*R*)-1-(2-Fluorophenyl)allyl)-1-(naphthalen-2-yl)cyclobutan-1-ol (12)

Prepared following **General Procedure C.** Purification by flash column chromatography (5:1:1 hexane/EtOAc/DCM) gave the title compound (42.3 mg, 64%, 90:10 dr, 98:2 er) as a colorless oil.

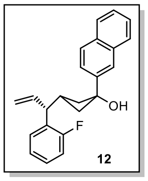
12

^{1}H NMR (400 MHz, $CDCl_3$): δ_H 7.92 (d, *J* = 1.9 Hz, 1H), 7.90 – 7.80 (m, 3H), 7.65 (dd, *J* = 8.6, 1.9 Hz, 1H), 7.54 – 7.44 (m, 2H), 7.24 – 7.13 (m, 2H), 7.11 – 6.99 (m, 2H), 5.97 (m, 1H), 5.16 – 5.09 (m, 1H), 5.09 (d, *J* = 1.1 Hz, 1H), 3.77 – 3.72 (m, 1H), 2.97 – 2.90 (m, 1H), 2.66 – 2.60 (m, 1H), 2.40 – 2.25 (m, 2H), 2.21 – 2.12 (m, 1H), 2.13 (s, 1H) ppm. **^{13}C NMR** (101 MHz, $CDCl_3$): δ_C 162.1, 159.7, 143.0, 138.4, 133.0 (d, *J* = 32.2 Hz), 129.3 (d, *J* = 14.9 Hz), 129.2 (d, *J* = 5.0 Hz), 128.8, 128.3, 128.0 (d, *J* = 8.3 Hz), 127.7, 126.4, 126.2, 124.3 (d, *J* = 3.6 Hz), 123.9 (d, *J* = 93.0 Hz), 115.8, 115.7 (d, *J* = 23.0 Hz), 72.7, 50.1, 42.4, 41.5, 29.3 ppm. **Specific rotation** $[\alpha]_D^{22}$ = +53 (c = 0.86, CH_2Cl_2). **HRMS** (ESI) m/z calculated for $C_{23}H_{20}F$ $[M-H_2O+H]^+$, 315.1544; found, 315.1542. **IR (thin film)** v_{max}/cm^{-1}: 3350, 3058, 2975, 2930, 1636, 1488, 1227, 819, 753. **HPLC conditions**: Chiral column IC, hexane: isopropanol = 95:5, flow rate = 1 mL/min, wavelength = 254 nm, t_R = 13.0 min for major isomer, t_R = 11.2 min for minor isomer.

(*1s*,*3s*)-1-(Naphthalen-2-yl)-3-((*R*)-1-(o-tolyl)allyl)cyclobutan-1-ol (13)

Prepared following **General Procedure C.** Purification by flash column chromatography (5:1:1 hexane/EtOAc/DCM) gave the title compound (39.4 mg, 60%, 91:9 dr, 97:3 er) as a colorless oil.

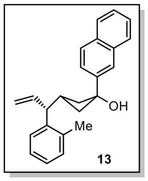
13

^{1}H NMR (400 MHz, $CDCl_3$): δ_H 7.94 (d, *J* = 1.9 Hz, 1H), 7.92 – 7.81 (m, 3H), 7.66 (dd, *J* = 8.6, 1.9 Hz, 1H), 7.54 – 7.47 (m, 2H), 7.20 – 7.09 (m, 4H), 5.85 (ddd, J = 17.1, 10.3, 7.5 Hz, 1H), 5.08 – 4.96 (m, 2H), 3.69 – 3.64 (m, 1H), 3.03 – 2.93 (m, 1H), 2.75 – 2.64 (m, 1H), 2.44 – 2.38 (m, 1H), 2.40 (s, 3H), 2.38 – 2.28 (m, 1H), 2.14 (s, 1H), 2.10 – 2.00 (m, 1H) ppm. **^{13}C NMR** (101 MHz, $CDCl_3$): δ_C 143.1, 140.4, 139.5, 136.5, 133.2, 132.8, 130.6, 128.8, 128.3, 127.7, 126.7, 126.4, 126.3, 126.2, 124.4, 123.4, 115.0, 72.9, 52.5, 42.3, 41.7, 29.4, 19.9 ppm. **HRMS** (ESI) m/z calculated for $C_{24}H_{23}$ $[M-H_2O+H]^+$, 311.1794; found, 311.1795. **IR (thin film)** v_{max}/cm^{-1}: 3350, 3056, 2973, 2930, 1633, 1488, 748. **HPLC conditions**: Chiral column IC, hexane: isopropanol = 97:3, flow rate = 1 mL/min, wavelength = 254 nm, t_R = 20.9 min for major isomer, t_R = 19.3 min for minor isomer.

(*1s*,*3s*)-3-((*R*)-1-(2-Methoxyphenyl)allyl)-1-(naphthalen-2-yl)cyclobutan-1-ol (14)

Prepared following **General Procedure B.** Purification by flash column chromatography (5:1:1 hexane/EtOAc/DCM) gave the title compound (42.7 mg, 62%, 90:10 dr, 97:3 er) as a colorless oil.

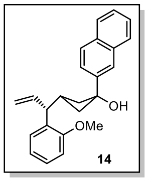
14

^{1}H NMR (400 MHz, $CDCl_3$): δ_H 7.93 (d, *J* = 1.9 Hz, 1H), 7.89 – 7.81 (m, 3H), 7.66 (dd, *J* = 8.6, 1.9 Hz, 1H), 7.51 – 7.45 (m, 2H), 7.22 – 7.18 (m, 1H), 7.12 (dd, *J* = 7.5, 1.7 Hz, 1H), 6.94 – 6.86 (m, 2H), 5.99 (ddd, *J* = 17.1, 10.3, 7.4 Hz, 1H), 5.10 – 5.06 (m, 1H), 5.05 – 5.03 (m, 1H), 3.94 – 3.89 (m, 1H), 3.85 (s, 3H), 2.99 – 2.88 (m, 1H), 2.69 – 2.55 (m, 1H), 2.41 – 2.25 (m, 2H), 2.21 – 2.11 (m, 2H) ppm. **^{13}C NMR** (101 MHz, $CDCl_3$): δ_C 157.3, 143.3, 139.5, 133.2, 132.8, 130.9, 128.7, 128.3, 128.3, 127.7, 127.4, 126.3, 126.1, 124.4, 123.4, 120.8, 114.8, 110.9, 72.8, 55.6, 49.3, 42.6, 41.6, 29.24 ppm. **Specific rotation** $[\alpha]_D^{22}$ = +52 (c = 1.22, CH_2Cl_2).

HRMS (ESI) m/z calculated for $C_{24}H_{23}$ [M-H_2O+H]$^+$, 321.1743; found, 327.1741. **IR (thin film)** ν_{max}/cm^{-1}: 3356, 3057, 2972, 2932, 2835, 1634, 1598, 1490, 1241, 1028, 750. **HPLC conditions**: Chiral column IA, hexane: isopropanol = 90:10, flow rate = 1 mL/min, wavelength = 254 nm, t_R = 12.0 min for major isomer, t_R = 14.8 min for minor isomer.

(*1s*,*3s*)-3-((*R*)-1-(3-Methoxyphenyl)allyl)-1-(naphthalen-2-yl)cyclobutan-1-ol (15)

Prepared following **General Procedure B.** Purification by flash column chromatography (5:1:1 hexane/EtOAc/DCM) gave the title compound (46.1 mg, 67%, 91:9 dr, 98.5:1.5 er) as a colorless oil.

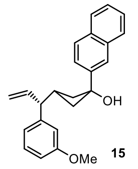

15

^{1}H NMR (400 MHz, $CDCl_3$): δ_H 7.91 (d, *J* = 1.9 Hz, 1H), 7.90 – 7.80 (m, 3H), 7.64 (dd, *J* = 8.6, 1.9 Hz, 1H), 7.53 – 7.46 (m, 2H), 7.28 – 7.18 (m, 1H), 6.84 – 6.73 (m, 3H), 5.94 (ddd, *J* = 17.0, 10.3, 7.6 Hz, 1H), 5.15 – 5.07 (m, 1H), 5.11 – 5.05 (m, 1H), 3.80 (s, 3H), 3.38 – 3.30 (m, 1H), 2.99 – 2.85 (m, 1H), 2.65 (ddd, *J* = 11.4, 6.5, 4.7 Hz, 1H), 2.33 – 2.21 (m, 2H), 2.21 – 2.11 (m, 2H) ppm. **^{13}C NMR** (101 MHz, $CDCl_3$): δ_C 159.8, 144.2, 143.1, 139.7, 133.1, 132.8, 129.5, 128.8, 128.3, 127.7, 126.4, 126.2, 124.3, 123.4, 120.4, 115.2, 113.9, 111.5, 72.8, 57.2, 55.3, 42.3, 41.8, 29.9 ppm. **HRMS** (ESI) m/z calculated for $C_{24}H_{23}O$ [M-H_2O+H]$^+$, 327.1743; found, 327.1756. **IR (thin film)** ν_{max}/cm $^{-1}$: 3369, 2972, 2931, 1599, 1489, 1262, 1046, 749. **HPLC conditions**: Chiral column IA, hexane: isopropanol = 95:5, flow rate = 1 mL/min, wavelength = 254 nm, t_R = 36.6 min for major isomer, t_R = 39.4 min for minor isomer.

(*1s*,*3s*)-3-((*R*)-1-(3,4-Dichlorophenyl)allyl)-1-(naphthalen-2-yl)cyclobutan-1-ol (16)

Prepared following **General Procedure C.** Purification by flash column chromatography (5:1:1 hexane/EtOAc/DCM) gave the title compound (41.3 mg, 54%, 92:8 dr, 97.5:2.5 er) as a colorless oil.

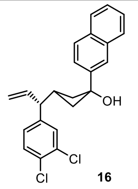

16

^{1}H NMR (400 MHz, $CDCl_3$): δ_H 7.92 – 7.80 (m, 4H), 7.63 (dd, *J* = 8.6, 1.9 Hz, 1H), 7.55 – 7.44 (m, 2H), 7.36 (d, *J* = 8.2 Hz, 1H), 7.27 (d, *J* = 2.1 Hz, 1H), 7.02 (dd, *J* = 8.3, 2.1 Hz, 1H), 5.87 (ddd, *J* = 17.1, 10.3, 7.5 Hz, 1H), 5.16 – 5.04 (m, 2H), 3.40 – 3.27 (m, 1H), 2.93 (ddd, *J* = 12.5, 6.7, 4.8 Hz, 1H), 2.67 – 2.57 (m, 1H), 2.31 – 2.17 (m, 2H), 2.16 – 2.08 (m, 2H) ppm. **^{13}C NMR** (101 MHz, $CDCl_3$): δ_C 142.9, 142.8, 138.7, 133.1, 132.9, 132.6, 130.5, 130.5, 129.9, 128.9, 128.3, 127.7, 127.4, 126.5, 126.3, 124.2, 123.4, 116.1, 72.8, 56.2, 42.0, 41.6, 29.8 ppm. **Specific rotation** $[\alpha]_D^{22}$ = +75 (c = 0.59, CH_2Cl_2). **HRMS** (ESI) m/z calculated for $C_{23}H_{19}Cl_2$ [M-H_2O+H]$^+$, 365.0858; found, 365.0871. **IR (thin film)** ν_{max}/cm^{-1}: 3338, 2975, 1635, 1471, 1029, 819, 748. **HPLC conditions**: Chiral column IC, hexane: isopropanol = 95:5, flow rate = 0.8 mL/min, wavelength = 254 nm, t_R = 15.5 min for major isomer, t_R = 13.8 min for minor isomer.

(*1s*,*3s*)-1-(Naphthalen-2-yl)-3-((*R*)-1-(naphthalen-2-yl)allyl)cyclobutan-1-ol (17)

Prepared following **General Procedure B.** Purification by flash column chromatography (5:1:1 hexane/EtOAc/DCM) gave the title compound (47.4 mg, 65%, 82:18 dr, 96.5:3.5 er) as a colorless oil.

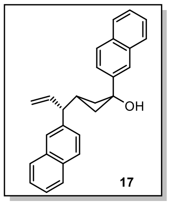

17

^{1}H NMR (400 MHz, $CDCl_3$): δ_H 7.92 (d, *J* = 1.9 Hz, 1H), 7.90 – 7.77 (m, 6H), 7.68 – 7.60 (m, 2H), 7.54 – 7.41 (m, 4H), 7.34 (dd, *J* = 8.5, 1.8 Hz, 1H), 6.04 (ddd, *J* = 17.0, 10.4, 7.5 Hz, 1H), 5.19 – 5.12 (m, 1H), 5.12 (d, *J* = 1.2 Hz, 1H), 3.61 – 3.49 (m, 1H), 3.04 – 2.91 (m, 1H), 2.64 (ddd, *J* = 12.0, 7.2, 5.0 Hz, 1H), 2.46 – 2.30 (m, 2H), 2.25 – 2.15 (m, 1H), 2.16 (s, 1H) ppm. **^{13}C NMR** (101 MHz, $CDCl_3$): δ_C 143.1, 140.0, 139.7, 133.7, 133.1, 132.8, 132.5, 128.8, 128.3, 128.2, 127.8, 127.8, 127.7, 126.5, 126.4, 126.3, 126.2, 126.1, 125.5, 124.4, 123.4, 115.4, 72.9, 57.3, 42.3, 41.89, 29.9 ppm. **HRMS** (ESI) m/z calculated for $C_{27}H_{23}$ [M-H_2O+H]$^+$, 347.1794; found, 347.1807. **IR (thin film)** ν_{max}/cm^{-1}: 3349, 3054, 29741634, ,1600, 1506, 1488, 1241, 817, 747. **HPLC conditions**: Chiral column IA, hexane: isopropanol = 93:7, flow rate = 1 mL/min, wavelength = 254 nm, t_R = 24.4 min for major isomer, t_R = 27.5 min for minor isomer.

(*1s*,*3s*)-1-(Naphthalen-2-yl)-3-((*R*)-1-(pyridin-3-yl)allyl)cyclobutan-1-ol (18)

Prepared following **General Procedure C.** Purification by flash column chromatography (3:1:1 hexane/EtOAc/DCM) gave the title compound (31.5 mg, 50%, 90:10 dr, 98.5:1.5 er) as a yellow oil.

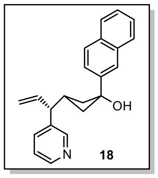
18

^{1}H NMR (400 MHz, $CDCl_3$): δ_H 8.43 – 8.37 (m, 2H), 7.91 – 7.86 (m, 1H), 7.89 – 7.79 (m, 3H), 7.63 (dd, *J* = 8.6, 1.9 Hz, 1H), 7.53 – 7.42 (m, 3H), 7.20 (ddd, *J* = 7.8, 4.8, 0.9 Hz, 1H), 5.89 (ddd, *J* = 17.1, 10.3, 7.4 Hz, 1H), 5.15 – 5.03 (m, 2H), 3.44 – 3.32 (m, 1H), 2.99 – 2.85 (m, 2H), 2.60 (ddd, *J* = 11.9, 7.1, 4.9 Hz, 1H), 2.36 – 2.16 (m, 2H), 2.16 – 2.06 (m, 1H) ppm. **^{13}C NMR** (101 MHz, $CDCl_3$): δ_C 149.6, 148.0, 143.1, 138.7, 137.9, 135.3, 133.1, 132.8, 128.8, 128.3, 127.7, 126.4, 126.2, 124.3, 123.6, 123.34, 116.1, 72.6, 54.4, 42.2, 41.6, 29.9 ppm. **Specific rotation** $[\alpha]_D^{22}$ = +50 (c = 0.72, CH_2Cl_2). **HRMS** (ESI) m/z calculated for $C_{22}H_{20}N$ $[M-H_2O+H]^+$, 316.1696; found, 316.1694. **IR (thin film)** ν_{max}/cm^{-1}: 3335, 2973, 2928, 1634, 1425, 1238, 1028, 750. **HPLC conditions**: Chiral column IA, hexane: isopropanol = 90:10, flow rate = 1 mL/min, wavelength = 254 nm, t_R = 54.5 min for major isomer, t_R = 52.2 min for minor isomer.

(*1s*,*3s*)-1-(Naphthalen-2-yl)-3-((*R*)-1-(1-tosyl-1H-indol-3-yl)allyl)cyclobutan-1-ol (19)

Prepared following **General Procedure B.** Purification by flash column chromatography (3:1:1 hexane/EtOAc/DCM) gave the title compound (57.8 mg, 57%, 83:17 dr, 97.5:2.5 er) as a thick oil.

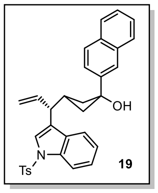
19

^{1}H NMR (400 MHz, $CDCl_3$): δ_H 7.88 (m, 1H), 7.84 (d, *J* = 1.9 Hz, 1H), 7.84 – 7.72 (m, 3H), 7.66 – 7.58 (m, 2H), 7.56 (dd, *J* = 8.6, 1.9 Hz, 1H), 7.45 – 7.38 (m, 3H), 7.25 – 7.18 (m, 2H), 7.14 – 7.06 (m, 3H), 5.86 – 5.71 (m, 1H), 5.05 – 5.03 (m, 1H), 5.01 (dd, *J* = 2.8, 1.0 Hz, 1H), 3.54 – 3.50 (m, 1H), 2.89 – 2.83 (m, 1H), 2.67 – 2.61 (m, 1H), 2.36 – 2.22 (m, 2H), 2.23 (s, 3H), 2.12 – 1.97 (m, 2H) ppm. **^{13}C NMR** (101 MHz, $CDCl_3$): δ_C 144.9, 143.0, 138.1, 135.6, 135.3, 133.2, 132.9, 130.7, 129.9, 128.9, 128.3, 127.7, 126.8, 126.5, 126.3, 124.8, 124.2, 123.5, 123.4, 123.12, 122.89, 120.4, 116.1, 114.0, 72.89, 48.0, 42.0, 42.0, 29.0, 21.7 ppm. **HRMS** (MALDI) m/z calculated for $C_{32}H_{29}NO_3SNa$ $[M-H_2O+H]^+$, 530.1760; found, 530.1768. **IR (thin film)** ν_{max}/cm^{-1}: 3398, 2975, 1598, 1447, 1367, 1173, 748. **HPLC conditions**: Chiral column IC, hexane: isopropanol = 92:8, flow rate = 1 mL/min, wavelength = 254 nm, t_R = 51.0 min for major isomer, t_R = 80.7 min for minor isomer.

(*1s*,*3s*)-1-Phenyl-3-((*R*)-1-phenylallyl)cyclobutan-1-ol (20)

Prepared following **General Procedure C.** Purification by flash column chromatography (5:1:1 hexane/EtOAc/DCM) gave the title compound (34.9 mg, 66%, 86:14 dr, 98:2 er) as colorless oil.

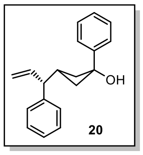
20

^{1}H NMR (400 MHz, $CDCl_3$): δ_H 7.55 – 7.50 (m, 2H), 7.42 – 7.36 (m, 2H), 7.33 – 7.27 (m, 3H), 7.24 – 7.16 (m, 3H), 5.94 (ddd, *J* = 17.5, 9.8, 7.6 Hz, 1H), 5.13 – 5.06 (m, 1H), 5.09 – 5.03 (m, 1H), 3.38 – 3.29 (m, 1H), 2.89 – 2.76 (m, 1H), 2.59 – 2.48 (m, 1H), 2.23 (m, 2H), 2.14 – 2.04 (m, 2H) ppm. **^{13}C NMR** (101 MHz, $CDCl_3$): δ_C 146.0, 142.5, 139.8, 128.7, 128.6, 127.9, 127.5, 126.5, 125.4, 115.1, 72.6, 57.2, 42.4, 41.9, 29.8 ppm. **HRMS** (ESI) m/z calculated for $C_{19}H_{19}$ $[M-H_2O+H]^+$, 247.1481; found, 247.1487. **IR (thin film)** ν_{max}/cm^{-1}: 3343, 2973, 2929, 1636, 1600, 1493, 912, 698. **HPLC conditions**: Chiral column IA, hexane: isopropanol = 95:5, flow rate = 0.8 mL/min, wavelength = 254 nm, t_R = 16.3 min for major isomer, t_R = 15.4 min for minor isomer.

(*1s*,*3s*)-1-(2- Chlorophenyl)-3-((*R*)-1-phenylallyl)cyclobutan-1-ol (21)

Prepared following **General Procedure C.** Purification by flash column chromatography (5:1:1 hexane/EtOAc/DCM) gave the title compound (34.0 mg, 57%, 67:33 dr, 96.5:3.5 er) as a colorless oil.

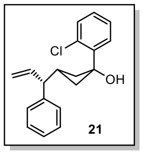
21

^{1}H NMR (400 MHz, $CDCl_3$): δ_H 7.40 – 7.35 (m, 1H), 7.32 – 7.28 (m, 1H), 7.25 – 7.23 (m, 1H), 7.22 – 7.08 (m, 6H), 5.84 (ddd, *J* = 16.8, 10.7, 7.7 Hz, 1H), 5.04 – 4.99 (m, 1H), 4.97 (d, *J* = 1.1 Hz, 1H), 3.38 – 3.30 (m, 1H), 2.93 (s, 1H), 2.92 – 2.86 (m, 1H), 2.62 – 2.55 (m, 1H), 2.24 – 2.16 (m, 1H), 2.11 – 2.00 (m, 2H) ppm. **^{13}C NMR** (101 MHz, $CDCl_3$): 142.6, 141.5, 139.9, 133.5, 131.2, 129.2, 128.6, 128.0, 127.0, 126.9, 126.5, 115.1, 73.6, 56.7, 40.4, 40.0, 30.6 ppm. **HRMS** (ESI) m/z calculated for $C_{19}H_{18}Cl$ $[M-H_2O+H]^+$, 281.1092; found, 281.1102. **IR (thin film)**

ν_{max}/cm^{-1}: 3410, 2980, 1636, 1600, 1492, 1040, 756. **HPLC conditions**: Chiral column IA, hexane: isopropanol = 95:5, flow rate = 1 mL/min, wavelength = 254 nm, t_R = 12.7 min for major isomer, t_R = 11.4 min for minor isomer.

(*1s*,*3s*)-1-(3-Bromophenyl)-3-((*R*)-1-phenylallyl)cyclobutan-1-ol (22)

Prepared following **General Procedure C.** Purification by flash column chromatography (5:1:1 hexane/EtOAc/DCM) gave the title compound (28.1 mg, 41%, 89:11 dr, 96.5:3.5 er) as a colorless oil.

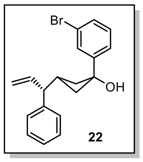

^{1}H NMR (400 MHz, $CDCl_3$): δ_H 7.57 (t, *J* = 1.9 Hz, 1H), 7.36 – 7.32 (m, 2H), 7.23 – 7.19 (m, 2H), 7.18 – 7.07 (m, 4H), 5.84 (ddd, *J* = 17.5, 9.7, 7.6 Hz, 1H), 5.01 – 5.00 (m, 1H), 4.98 – 4.96 (m, 1H), 3.24 (dd, *J* = 9.3, 7.6 Hz, 1H), 2.74 – 2.62 (m, 1H), 2.39 (ddd, *J* = 12.2, 7.2, 4.9 Hz, 1H), 2.22 – 2.09 (m, 2H), 2.04 – 1.96 (m, 1H), 1.96 (s, 1H) ppm. **^{13}C NMR** (101 MHz, $CDCl_3$): δ_C 148.5, 142.3, 139.6, 130.6, 130.3, 128.7, 128.6, 127.9, 126.6, 124.0, 122.9, 115.23, 72.3, 57.1, 42.5, 42.01, 29.7 ppm. **HRMS** (ESI) m/z calculated for $C_{23}H_{22}O$ [M-H_2O+H]$^+$, 326.0670; found, 326.0558. **IR (thin film)** ν_{max}/cm^{-1}: 3341, 2976, 1595, 1565, 1413, 1072, 751, 700.

(*1s*,*3s*)-1-(4-Chlorophenyl)-3-((*R*)-1-phenylallyl)cyclobutan-1-ol (23)

Prepared following **General Procedure C.** Purification by flash column chromatography (5:1:1 hexane/EtOAc/DCM) gave the title compound (42.3 mg, 71%, 91:9 dr, 97:3 er) as a colorless oil.

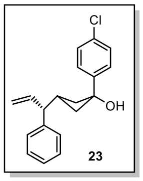

^{1}H NMR (400 MHz, $CDCl_3$): δ_H 7.47 – 7.41 (m, 2H), 7.37 – 7.26 (m, 4H), 7.26 – 7.13 (m, 3H), 5.92 (ddd, *J* = 17.5, 9.7, 7.7 Hz, 1H), 5.09 – 5.08 (m, 1H), 5.08 – 5.02 (m, 1H), 3.32 (dd, *J* = 9.2, 7.5 Hz, 1H), 2.82 – 2.68 (m, 1H), 2.47 (ddd, *J* = 11.9, 6.8, 4.9 Hz, 1H), 2.26 – 2.17 (m, 2H), 2.12 – 2.04 (m, 1H), 2.05 (s, 1H) ppm. **^{13}C NMR** (101 MHz, $CDCl_3$): δ_C 144.6, 142.3, 139.7, 133.3, 128.7, 128.6, 127.9, 126.9, 126.6, 115.2, 72.3, 57.1, 42.6, 42.1, 29.7 ppm. **HRMS** (ESI) m/z calculated for $C_{19}H_{18}Cl$ [M-H_2O+H]$^+$, 281.1092; found, 281.1102. **IR (thin film)** ν_{max}/cm^{-1}: 3337, 2974, 2930, 1636, 1599, 1491, 1013, 831, 700. **HPLC conditions**: Chiral column IA, hexane: isopropanol = 97:3, flow rate = 0.8 mL/min, wavelength = 254 nm, t_R = 29.4 min for major isomer, t_R = 28.0 min for minor isomer.

(*1s*,*3s*)-3-((*R*)-1-Phenylallyl)-1-(4-(trifluoromethyl)phenyl)cyclobutan-1-ol (24)

Prepared following **General Procedure C.** Purification by flash column chromatography (5:1:1 hexane/EtOAc/DCM) gave the title compound (46.5 mg, 70%, 86:14 dr, 97:3 er) as a colorless oil.

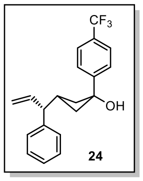

^{1}H NMR (400 MHz, $CDCl_3$): δ_H 7.63 (m, 4H), 7.36 – 7.27 (m, 2H), 7.26 – 7.15 (m, 3H), 5.94 (ddd, *J* = 17.5, 9.7, 7.6 Hz, 1H), 5.10 – 5.10 (m, 1H), 5.08 – 5.06 (m, 1H), 3.34 (dd, *J* = 9.3, 7.6 Hz, 1H), 2.85 – 2.72 (m, 1H), 2.56 – 2.44 (m, 1H), 2.33 – 2.22 (m, 2H), 2.19 – 2.06 (m, 2H) ppm. **^{13}C NMR** (101 MHz, $CDCl_3$): δ_C 150.1, 139.6, 129.7 (q, 22.0 Hz), 128.7, 127.9, 126.7, 125.7, 125.6 (q, 4.0 Hz), 124.3 (q, 270 Hz), 115.3, 72.4, 57.1, 42.7, 42.2, 29.8 ppm. **HRMS** (ESI) m/z calculated for $C_{20}H_{18}F_3$ [M-H_2O+H]$^+$, 315.1355 found, 315.1364. **IR (thin film)** ν_{max}/cm^{-1}: 3342, 2976, 2932, 1619, 1408, 1326, 1123, 701. **HPLC conditions**: Chiral column IB, hexane: isopropanol = 98:2, flow rate = 0.5 mL/min, wavelength = 254 nm, t_R = 26.8 min for major isomer, t_R = 30.4 min for minor isomer.

(*1s*,*3s*)-3-((*R*)-1-Phenylallyl)-1-(p-tolyl)cyclobutan-1-ol (25)

Prepared following **General Procedure C.** Purification by flash column chromatography (5:1:1 hexane/EtOAc/DCM) gave the title compound (39.0 mg, 70%, 89:11 dr, 97.5:2.5 er) as a colorless oil.

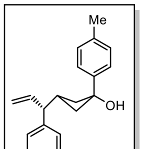
25

^{1}H NMR (400 MHz, $CDCl_3$): δ_H 7.43 – 7.38 (m, 2H), 7.34 – 7.25 (m, 2H), 7.21 – 7.17 (m, 5H), 5.93 (ddd, *J* = 17.5, 9.8, 7.6 Hz, 1H), 5.11 – 5.05 (m, 1H), 5.05 (d, *J* = 1.1 Hz, 1H), 3.35 – 3.30 (m, 1H), 2.87 – 2.73 (m, 1H), 2.51 (ddd, *J* = 11.5, 6.5, 4.8 Hz, 1H), 2.36 (s, 3H), 2.26 – 2.14 (m, 2H), 2.12 – 2.02 (m, 1H), 1.90 (s, 1H) ppm. **^{13}C NMR** (101 MHz, $CDCl_3$): δ_C 143.1, 142.6, 139.9, 137.3, 129.3, 128.6, 127.9, 126.5, 125.4, 115.0, 72.5, 57.2, 42.4, 41.9, 29.8, 21.2 ppm. **HRMS** (ESI) m/z calculated for $C_{20}H_{21}$ [M-H_2O+H]$^+$, 261.1638 found, 261.1637. **IR (thin film)** v_{max}/cm^{-1}: 3344, 3026, 2974, ,2926, 1635, 1600, 1451, 913, 700. **HPLC conditions**: Chiral column IC, hexane: isopropanol = 97:3, flow rate = 0.5 mL/min, wavelength = 254 nm, t_R = 22.7 min for major isomer, t_R = 21.6 min for minor isomer.

(*1s*,*3s*)-1-(4-Methoxyphenyl)-3-((*R*)-1-phenylallyl)cyclobutan-1-ol (26)

Prepared following **General Procedure C.** Purification by flash column chromatography (5:1:1 hexane/EtOAc/DCM) gave the title compound (36.5 mg, 62%, 89:11 dr, 96:4 er) as a colorless oil.

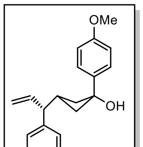
26

^{1}H NMR (400 MHz, $CDCl_3$): δ_H 7.47 – 7.40 (m, 2H), 7.33 – 7.27 (m, 2H), 7.23 – 7.14 (m, 3H), 6.94 – 6.86 (m, 2H), 5.92 (ddd, *J* = 17.5, 9.8, 7.6 Hz, 1H), 5.11 – 5.04 (m, 1H), 5.05 – 5.04 (m, 1H), 3.81 (s, 3H), 3.31 (dd, *J* = 9.1, 7.5 Hz, 1H), 2.87 – 2.72 (m, 1H), 2.50 (ddd, *J* = 11.4, 6.7, 5.0 Hz, 1H), 2.24 – 2.11 (m, 2H), 2.10 – 2.00 (m, 1H), 2.02 (s, 1H) ppm. **^{13}C NMR** (101 MHz, $CDCl_3$): δ_C 159.0, 142.6, 139.9, 138.2, 128.6, 127.9, 126.8, 126.5, 115.0, 113.9, 72.3, 57.1, 55.4, 42.4, 41.9, 29.8 ppm. **HRMS** (ESI) m/z calculated for $C_{20}H_{21}O$ [M-H_2O+H]$^+$, 277.1587 found, 277.1582. **IR (thin film)** v_{max}/cm^{-1}: 3364, 2973, 2931, 1610, 1513, 1249, 1034, 915, 832, 701. **HPLC conditions**: Chiral column IC, hexane: isopropanol = 98:2, flow rate = 0.8 mL/min, wavelength = 254 nm, t_R = 53.7 min for major isomer, t_R = 48.3 min for minor isomer.

(*1s*,*3s*)-1-(3,4-Dichlorophenyl)-3-((*R*)-1-phenylallyl)cyclobutan-1-ol (27)

Prepared following **General Procedure C.** Purification by flash column chromatography (5:1:1 hexane/EtOAc/DCM) gave the title compound (44.5 mg, 67%, 88:12 dr, 97:3 er) as a colorless oil.

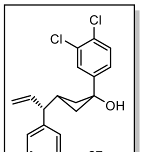
27

^{1}H NMR (400 MHz, $CDCl_3$): δ_H 7.59 (d, *J* = 2.1 Hz, 1H), 7.46 – 7.39 (m, 1H), 7.35 – 7.28 (m, 3H), 7.25 – 7.15 (m, 3H), 5.99 – 5.85 (m, 1H), 5.09 (d, *J* = 1.1 Hz, 1H), 5.09 – 5.02 (m, 1H), 3.33 – 3.29 (m, 1H), 2.79 – 2.67 (m, 1H), 2.44 (ddd, *J* = 12.3, 7.2, 5.0 Hz, 1H), 2.29 – 2.15 (m, 2H), 2.10 (s, 1H), 2.10 – 1.98 (m, 1H) ppm. **^{13}C NMR** (101 MHz, $CDCl_3$): δ_C 146.6, 142.1, 139.5, 132.7, 130.6, 128.7, 127.9, 127.7, 126.7, 124.8, 115.4, 72.0, 57.1, 42.7, 42.2, 29.7 ppm**. HRMS** (ESI) m/z calculated for $C_{19}H_{17}Cl_2$ [M-H_2O+H]$^+$, 315.0702 found, 315.0711. **IR (thin film)** v_{max}/cm^{-1}: 3668, 3338, 2977, 1469, 1378, 1066, 915, 701. **HPLC conditions**: Chiral column IA, hexane: isopropanol = 97:3, flow rate = 0.8 mL/min, wavelength = 254 nm, t_R = 26.1 min for major isomer, t_R = 23.4 min for minor isomer.

(*1s*,*3s*)-1-(Naphthalen-1-yl)-3-((*R*)-1-phenylallyl)cyclobutan-1-ol (28)

Prepared following **General Procedure C.** Purification by flash column chromatography (5:1:1 hexane/EtOAc/DCM) gave the title compound (42.1 mg, 67%, 86:14 dr, 97:3 er) as a colorless oil.

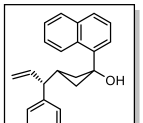
28

^{1}H NMR (400 MHz, $CDCl_3$): δ_H 8.32 – 8.27 (m, 1H), 7.91 – 7.84 (m, 1H), 7.82 – 7.80 (m, 1H), 7.57 (dd, *J* = 7.2, 1.2 Hz, 1H), 7.53 – 7.44 (m, 2H), 7.43 (dd, *J* = 8.1, 7.1 Hz, 1H), 7.33 – 7.24 (m, 2H), 7.21 – 7.16 (m, 3H), 5.93 (ddd, *J* = 17.0, 10.3, 7.6 Hz, 1H), 5.14 – 5.05 (m, 1H), 5.09 – 5.03 (m, 1H), 3.49 – 3.37 (m, 1H), 3.20 – 3.09 (m, 1H), 2.86 – 2.79 (m, 1H), 2.48 – 2.37 (m, 1H), 2.35 – 2.25 (m, 2H), 2.25 – 2.12 (m, 1H) ppm. **^{13}C NMR** (101 MHz, $CDCl_3$): δ_C 142.6, 140.0, 139.7, 135.0, 130.9, 129.2, 129.1, 128.6, 127.9, 126.5, 126.5, 125.9, 125.8, 124.9, 123.0, 115.0, 73.9, 57.1, 42.2, 41.8, 31.2 ppm. **HRMS** (ESI) m/z calculated for $C_{23}H_{21}$ [M-H_2O+H]$^+$, 297.1638 found, 297.1638. **IR (thin film)** v_{max}/cm^{-1}: 3667, 3344, 2976, 2925, 1637, 1599, 1492, 779, 700. **HPLC conditions**: Chiral column IA, hexane: isopropanol = 95:5, flow rate = 1 mL/min, wavelength = 254 nm, t_R = 17.0 min for major isomer, t_R = 15.3 min for minor isomer.

tert-Butyl 5-((*1s*,*3s*)-1-hydroxy-3-((*R*)-1-phenylallyl)cyclobutyl)-1H-indole-1-carboxylate (29)

Prepared following **General Procedure C.** Purification by flash column chromatography (3:1:1 hexane/EtOAc/DCM) gave the title compound (69.4 mg, 86%, 91:9 dr, 98.5:1.5 er) as a thick oil.

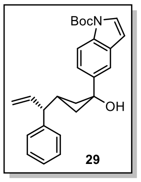

^{1}H NMR (400 MHz, $CDCl_3$): δ_H 8.13 (d, J = 8.6 Hz, 1H), 7.69 (dd, *J* = 1.9, 0.7 Hz, 1H), 7.61 (d, J = 3.7 Hz, 1H), 7.48 (dd, J = 8.7, 1.9 Hz, 1H), 7.34 – 7.25 (m, 2H), 7.23 – 7.14 (m, 3H), 6.56 (dd, J = 3.8, 0.8 Hz, 1H), 5.12 – 5.05 (m, 1H), 5.05 (d, J = 1.1 Hz, 1H), 3.36 – 3.32 (m, 1H), 2.95 – 2.81 (m, 1H), 2.59 (ddd, J = 10.7, 6.7, 5.0 Hz, 1H), 2.30 – 2.18 (m, 2H), 2.17 – 2.07 (m, 1H), 2.06 (s, 1H), 1.68 (s, 9H) ppm. **^{13}C NMR** (101 MHz, $CDCl_3$): δ_C 149.8, 142.6, 140.5, 139.9, 130.7, 128.6, 127.9, 126.6, 126.5, 122.2, 117.6, 115.5, 115.0, 107.6, 83.9, 72.9, 57.2, 42.6, 42.1, 29.9, 28.3 ppm. **HRMS** (ESI) m/z calculated for $C_{26}H_{28}NO_2$ $[M-H_2O+H]^+$, 386.2115 found, 386.2126. **IR (thin film)** v_{max}/cm^{-1}: 3668, 3358, 2977, 1733, 1472, 1370, 1084, 912, 701. **HPLC conditions**: Chiral column IC, hexane: isopropanol = 90:10, flow rate = 0.8 mL/min, wavelength = 254 nm, t_R = 11.7 min for major isomer, t_R = 10.7 min for minor isomer.

(*1s*,*3s*)-1-(Furan-2-yl)-3-((*R*)-1-phenylallyl)cyclobutan-1-ol (30)

Prepared following **General Procedure C.** Purification by flash column chromatography (5:1:1 hexane/EtOAc/DCM) gave the title compound (30.0 mg, 59%, 74:26 dr, 97:3 er) as a colorless oil.

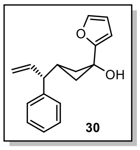

^{1}H NMR (400 MHz, $CDCl_3$): δ_H 7.38 (dd, J = 1.8, 0.8 Hz, 1H), 7.32 – 7.25 (m, 2H), 7.23 – 7.14 (m, 3H), 6.33 (dd, J = 3.2, 1.8 Hz, 1H), 6.28 (dd, J = 3.2, 0.9 Hz, 1H), 5.91 (ddd, J = 17.4, 9.7, 7.6 Hz, 1H), 5.07 (p, J = 1.7 Hz, 1H), 5.07 – 5.00 (m, 1H), 3.32 – 3.22 (m, 1H), 2.75 (m, 1H), 2.48 – 2.42 (m, 1H), 2.32 – 2.21 (m, 2H), 2.22 – 2.09 (m, 1H), 2.02 (dd, J = 11.6, 9.1 Hz, 1H) ppm. **^{13}C NMR** (101 MHz, $CDCl_3$): δ_C 149.8, 142.6, 140.5, 139.9, 130.7, 128.6, 127.9, 126.6, 126.5, 122.2, 117.6, 115.5, 115.0, 107.6, 83.9, 72.9, 57.2, 42.6, 42.1, 29.9, 28.3 ppm. **HRMS** (ESI) m/z calculated for $C_{17}H_{17}$ $[M-H_2O+H]^+$, 237.1274 found, 237.1271. **IR (thin film)** v_{max}/cm^{-1}: 3360, 2979, 1452, 1394, 1240, 1064, 916, 701. **HPLC conditions**: Chiral column IC, hexane: isopropanol = 97:3, flow rate = 0.8 mL/min, wavelength = 254 nm, t_R = 20.2 min for major isomer, t_R = 16.5 min for minor isomer.

(*1s*,*3s*)-1-(5-Methylthiophen-2-yl)-3-((*R*)-1-phenylallyl)cyclobutan-1-ol (31)

Prepared following **General Procedure C.** Purification by flash column chromatography (5:1:1 hexane/EtOAc/DCM) gave the title compound (35.2 mg, 62%, 86:14 dr, 96.5:3.5 er) as a white soild.

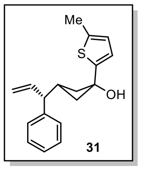

^{1}H NMR (400 MHz, $CDCl_3$): δ_H δ 7.33 – 7.27 (m, 2H), 7.24 – 7.14 (m, 3H), 6.83 (d, J = 3.4 Hz, 1H), 6.60 (dq, J = 3.5, 1.2 Hz, 1H), 5.91 (ddd, J = 17.5, 9.9, 7.6 Hz, 1H), 5.10 – 5.04 (m, 1H), 5.07 – 5.01 (m, 1H), 3.27 (dd, J = 9.7, 7.6 Hz, 1H), 2.80 – 2.69 (m, 1H), 2.50 – 2.39 (m, 5H), 2.38 – 2.23 (m, 1H), 2.27 – 2.16 (m, 2H), 2.16 – 2.04 (m, 1H) ppm. **^{13}C NMR** (101 MHz, $CDCl_3$): δ_C 149.1, 142.4, 139.8, 139.7, 128.6, 127.9, 126.5, 124.7, 123.3, 115.1, 70.5, 57.0, 43.7, 43.2, 30.1, 15.5 ppm. **HRMS** (ESI) m/z calculated for $C_{18}H_{19}S$ $[M-H_2O+H]^+$, 267.1202 found, 267.1198. **IR (thin film)** v_{max}/cm^{-1}: 3342, 2974, 2929, 1636, 1492, 1236, 1057, 915, 700. **HPLC conditions**: Chiral column AD-H, hexane: isopropanol = 97:3, flow rate = 0.8 mL/min, wavelength = 254 nm, t_R = 32.6 min for major isomer, t_R = 29.9 min for minor isomer.

(*1s*,*3r*)-1-Phenethyl-3-((*R*)-1-phenylallyl)cyclobutan-1-ol (32)

Prepared following **General Procedure C.** Purification by flash column chromatography (5:1:1 hexane/EtOAc/DCM) gave the title compound (35.1 mg, 60%, 83:17 dr, 97:3 er) as a colorless oil.

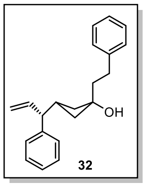

^{1}H NMR (400 MHz, $CDCl_3$): δ_H 7.34 – 7.27 (m, 4H), 7.25 – 7.15 (m, 6H), 5.93 (ddd, J = 16.8, 10.5, 7.5 Hz, 1H), 5.10 – 5.04 (m, 1H), 5.08 – 5.00 (m, 1H), 3.27 – 3.18 (m, 1H), 2.76 – 2.67 (m, 2H), 2.42 – 2.35 (m, 1H), 2.24 – 2.11 (m, 1H), 2.14 – 2.05 (m, 1H), 1.97 – 1.88 (m, 2H), 1.85 (dd, J = 11.8, 8.7 Hz, 1H), 1.72 (dd, J = 11.6, 8.6 Hz, 1H), 1.64 (s, 1H) ppm. **^{13}C NMR** (101 MHz, $CDCl_3$): δ_C 142.6, 142.5, 139.9, 128.6, 128.6, 128.5, 127.9, 126.5, 126.0, 115.0, 71.2, 57.4, 42.1, 41.6, 41.1, 30.2, 30.1 ppm. **HRMS** (ESI) m/z calculated for $C_{21}H_{23}$ $[M-H_2O+H]^+$, 275.1794 found, 275.1799. **IR (thin film)** v_{max}/cm^{-1}: 3356, 2970, 2925, 1602, 1494,

1453, 1249, 106, 913, 699. **HPLC conditions**: Chiral column IC, hexane: isopropanol = 95:5, flow rate = 1.0 mL/min, wavelength = 254 nm, t_R = 10.3 min for major isomer, t_R = 8.0 min for minor isomer.

(*1s*,*3s*)-1-Methyl-3-((*R*)-1-phenylallyl)cyclobutan-1-ol (33)

Prepared following **General Procedure C.** Purification by flash column chromatography (5:1:1 hexane/EtOAc/DCM) gave the title compound (25.1 mg, 62%, 90:10 dr, 97.5:2.5 er) as a colorless oil.

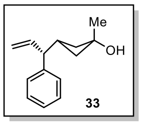

^{1}H NMR (400 MHz, $CDCl_3$): δ_H 7.32 – 7.27 (m, 2H), 7.23 – 7.12 (m, 3H), 5.90 (ddd, *J* = 16.9, 10.5, 7.5 Hz, 1H), 5.08 – 5.01 (m, 1H), 5.05 – 4.97 (m, 1H), 3.21 – 3.12 (m, 1H), 2.34 – 2.21 (m, 1H), 2.21 – 2.06 (m, 1H), 2.00 – 1.94 (m, 1H), 1.89 – 1.79 (m, 1H), 1.77 – 1.67 (m, 1H), 1.63 (s, 1H), 1.39 – 1.34 (s, 3H) ppm. **^{13}C NMR** (101 MHz, $CDCl_3$): δ_C 142.7, 139.9, 128.5, 127.9, 126.5, 114.9, 69.0, 57.3, 43.3, 42.8, 29.8, 27.7 ppm. **HRMS** (ESI) m/z calculated for $C_{14}H_{17}$ $[M-H_2O+H]^+$, 185.1325 found, 185.1322. **IR (thin film)** v_{max}/cm^{-1}: 3337, 2965, 2925, 1637, 1601, 1260, 913, 699. **HPLC conditions**: Chiral column OJ, hexane: isopropanol = 95:5, flow rate = 1.0 mL/min, wavelength = 254 nm, t_R = 17.1 min for major isomer, t_R = 13.4 min for minor isomer.

(*1s*,*3s*)-1-Cyclohexyl-3-((*R*)-1-phenylallyl)cyclobutan-1-ol (34)

Prepared following **General Procedure C.** Purification by flash column chromatography (5:1:1 hexane/EtOAc/DCM) gave the title compound (28.1 mg, 52%, 63:3 dr, 97.5: 2.5 er) as a colorless oil.

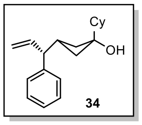

^{1}H NMR (400 MHz, $CDCl_3$): δ_H 7.31 – 7.27 (m, 2H), 7.24 – 7.12 (m, 3H), 5.98 – 5.79 (m, 1H), 5.04 – 4.96 (m, 2H), 3.31 – 3.15 (m, 0.7H), 3.08 (dd, *J* = 10.5, 7.5 Hz, 0.3H), 2.88 – 2.77 (m, 0.3H), 2.50 – 2.36 (m, 0.7H), 2.19 – 2.01 (m, 2H), 1.86 – 1.70 (m, 4H), 1.73 – 1.58 (m, 3H), 1.39 – 1.30 (m, 1H), 1.26 – 1.06 (m, 4H), 1.09 – 0.91 (m, 2H) ppm. **^{13}C NMR** (101 MHz, $CDCl_3$): δ_C 142.8, 140.1, 128.5, 127.9, 126.4, 114.9, 73.6, 57.6, 45.5, 39.5, 39.0, 29.7, 26.7, 26.6, 26.6, 25.9, 25.9 ppm. **HRMS** (ESI) m/z calculated for $C_{19}H_{25}$ $[M-H_2O+H]^+$, 253.1951 found, 253.1950. **IR (thin film)** v_{max}/cm^{-1}: 3389, 2925, 2852, 1636, 1601, 1493, 1451, 1256, 1074, 912, 699. **HPLC conditions**: Chiral column IC, hexane: isopropanol = 95:5, flow rate = 1.0 mL/min, wavelength = 254 nm, t_R = 6.2 min for major isomer, t_R = 6.8 min for minor isomer.

(*1s*,*3s*)-1-(tert-Butyl)-3-((*R*)-1-phenylallyl)cyclobutan-1-ol (35)

Prepared following **General Procedure C.** Purification by flash column chromatography (5:1:1 hexane/EtOAc/DCM) gave the title compound (24.4 mg, 50%, 65:35 dr, 97.5:2.5 er) as a colorless oil.

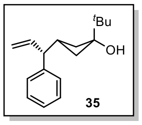

^{1}H NMR (400 MHz, $CDCl_3$): δ_H 7.34 – 7.25 (m, 2H), 7.24 – 7.13 (m, 3H), 5.97 – 5.83 (m, 1H), 5.08 – 5.04 (m, 1H), 5.03 – 5.00 (m, 1H), 3.27 (dd, *J* = 10.3, 7.6 Hz, 0.4H), 3.09 (dd, *J* = 10.4, 7.4 Hz, 0.6H), 2.88 – 2.77 (m, 0.6H), 2.54 (dddd, *J* = 13.0, 8.6, 3.5, 1.1 Hz, 0.6H), 2.26 (dddd, *J* = 12.7, 8.7, 3.5, 1.1 Hz, 0.4H), 2.18 – 2.03 (m, 0.4H), 2.04 – 1.94 (m, 0.4H), 1.93 – 1.88 (m, 0.6H), 1.89 – 1.79 (m, 0.6H), 1.73 (dd, *J* = 12.9, 7.7 Hz, 0.4H), 1.67 – 1.58 (m, 0.4H), 1.58 – 1.53 (m, 0.6H), 0.92 (s, 3.6H), 0.87 (s, 5.4H) ppm. **^{13}C NMR** (101 MHz, $CDCl_3$): δ_C 142.6, 142.5, 140.2, 140.1, 128.4, 127.9, 126.3, 114.7, 114.5, 79.4, 76.5, 57.7, 56.2, 37.3, 36.8, 35.6, 35.5, 35.1, 34.6, 31.1, 29.2, 24.3, 23.8 ppm. **HRMS** (ESI) m/z calculated for $C_{17}H_{23}$ $[M-H_2O+H]^+$, 227.1794 found, 227.1792. **IR (thin film)** v_{max}/cm^{-1}: 3398, 2963, 1636, 1600, 1471, 1364, 1051, 909, 700.

(*1s*,*3s*)-1-(5-(2,5-Dimethylphenoxy)-2-methylpentan-2-yl)-3-((*R*)-1-phenylallyl)cyclobutan-1-ol (36)

Prepared following **General Procedure C.** Purification by flash column chromatography (5:1:1 hexane/EtOAc/DCM) gave the title compound (39.2 mg, 50%, 70:30 dr, 96:4 er) as a thick oil.

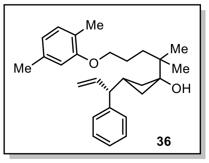

^{1}H NMR (400 MHz, $CDCl_3$): δ_H 7.32-7.27 (m, 2H), 7.25 – 7.14 (m, 3H), 7.01 (dd, J = 7.5, 0.9 Hz, 1H), 6.70 – 6.63 (m, 1H), 6.62 (d, J = 1.6 Hz, 1H), 5.97 – 5.82 (m, 1H), 5.05 (ddd, J = 4.6, 1.7, 1.0 Hz, 1H), 5.06 – 4.99 (m, 1H), 3.91 (t, J = 6.2 Hz, 2H), 3.30 (dd, J = 10.3, 7.6 Hz, 0.3H), 3.10 (dd, J = 10.3, 7.4 Hz, 0.7H), 2.91 – 2.80 (m, 0.7 H), 2.59 (dddd, J = 13.1, 8.7, 3.2, 1.1 Hz, 0.3H), 2.31 (s, 3H), 2.18 (s, 3H), 2.02 (dd, J = 12.3, 8.7 Hz, 0.7H), 1.95 – 1.83 (m, 1.5H), 1.83 – 1.68 (m, 2.1H), 1.70 – 1.59 (m, 0.7H), 1.54

– 1.36 (m, 2H), 0.92 (d, J = 5.3 Hz, 1.8H), 0.87 (d, J = 6.7 Hz, 4.2H) ppm. **^{13}C NMR** (101 MHz, $CDCl_3$): δ_C 157.2, 142.6, 140.2, 136.6, 130.4, 128.5, 128.0, 126.5, 123.7, 120.8, 114.7, 112.3, 79.9, 68.8, 56.3, 38.1, 36.0, 35.4, 32.9, 32.0, 24.8, 21.8, 21.8, 21.5, 16.0 ppm. **HRMS** (ESI) m/z calculated for $C_{27}H_{35}O$ $[M-H_2O+H]^+$, 375.2682 found, 375.2696. **IR (thin film)** ν_{max}/cm^{-1}: 3362, 2957, 1615, 1585, 1508, 1263, 1129, 1043, 908, 700. **HPLC conditions**: Chiral column IC, hexane: isopropanol = 98:2, flow rate = 0.5 mL/min, wavelength = 254 nm, t_R = 16.2 min for major isomer, t_R = 19.5 min for minor isomer.

(*1s*,*3s*)-3-((*R*)-1-Phenylallyl)-1-(prop-1-en-2-yl)cyclobutan-1-ol (37)

Prepared following **General Procedure C.** Purification by flash column chromatography (5:1:1 hexane/EtOAc/DCM) gave the title compound (27.4 mg, 60%, 87:13 dr, 98: er) as a colorless oil.

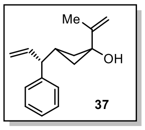

^{1}H NMR (400 MHz, $CDCl_3$): δ_H 7.32 – 7.24 (m, 2H), 7.24 – 7.12 (m, 3H), 5.97 – 5.82 (m, 1H), 5.09 – 4.98 (m, 1H), 5.05 – 4.99 (m, 1H), 5.03 – 4.98 (m, 1H), 4.89 – 4.87 (m, 1H), 3.34 – 3.13 (m, 1H), 2.60 – 2.55 (m, 1H), 2.31 – 2.25 (m, 1H), 2.19 – 2.08 (m, 1H), 1.90 (dd, J = 11.9, 8.8 Hz, 1H), 1.80 (dd, J = 1.4, 0.7 Hz, 3H), 1.81 – 1.71 (m, 1H), 1.71 – 1.69 (m, 1H) ppm. **^{13}C NMR** (101 MHz, $CDCl_3$): δ_C 147.7, 142.6, 140.0, 128.6, 127.9, 126.5, 114.9, 110.1, 73.5, 57.3, 40.0, 39.5, 30.1, 17.7 ppm. **Specific rotation** $[\alpha]_D^{22}$ = +41 (c = 0.0.54, CH_2Cl_2). **HRMS** (ESI) m/z calculated for $C_{16}H_{19}$ $[M-H_2O+H]^+$, 211.1481 found, 211.1479. **IR (thin film)** ν_{max}/cm^{-1}: 3352, 3083, 2973, 2926, 1634, 1601, 1492, 1451, 1238, 1071, 904, 700. **HPLC conditions**: Chiral column IA, hexane: isopropanol = 98:2, flow rate = 0.5 mL/min, wavelength = 254 nm, t_R = 27.2 min for major isomer, t_R = 23.5 min for minor isomer.

(*R*)-2,2'-(3-(1-Phenylallyl)cyclobutane-1,1-diyl)bis(4,4,5,5-tetramethyl-1,3,2-dioxaborolane) (38)

Prepared following **General Procedure C.** Purification by flash column chromatography (20:1 hexane/Et_2O) gave the title compound (51.8 mg, 61%, 97.5:2.5 er) as a colorless oil.

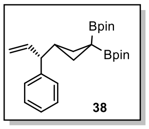

^{1}H NMR (400 MHz, $CDCl_3$): δ_H 7.23 – 7.14 (m, 2H), 7.13 – 7.02 (m, 3H), 5.78 (ddd, J = 16.9, 10.5, 7.6 Hz, 1H), 4.95 – 4.89 (m, 1H), 4.90 (ddd, J = 10.6, 1.9, 1.1 Hz, 1H), 3.18 – 3.05 (m, 1H), 2.62 – 2.51 (m, 1H), 2.31 (ddd, J = 10.7, 8.2, 2.3 Hz, 1H), 2.05 (ddd, J = 10.6, 8.4, 2.3 Hz, 1H), 1.98 (dd, J = 10.8, 7.8 Hz, 1H), 1.81 (dd, J = 10.8, 7.8 Hz,1H), 1.17 (s, 12H), 1.16 (s, 12H) ppm. **^{13}C NMR** (101 MHz, $CDCl_3$): δ_C 142.8, 140.1, 128.4, 128.1, 126.2, 114.8, 83.2, 83.2, 56.5, 38.9, 31.4, 30.9, 24.8 ppm. **Specific rotation** $[\alpha]_D^{22}$ = +9 (c = 1.11, CH_2Cl_2). **HRMS** (ESI) m/z calculated for $C_{25}H_{39}B_2O_4$ $[M +H]^+$, 425.3029 found, 425.3040. **IR (thin film)** ν_{max}/cm^{-1}: 3393, 2978, 1781, 1453, 1371, 1336, 1145, 982, 850, 702. **HPLC conditions**: Enantiomeric excess was determined after oxidation according to **procedure F**. Chiral column IB, hexane: isopropanol = 95:5, flow rate = 1.0 mL/min, wavelength = 254 nm, t_R = 29.7 min for major isomer, t_R = 31.7 min for minor isomer.

(*1s*,*3s*)-1,3-Dimethyl-3-((*R*)-1-phenylallyl)cyclobutan-1-ol (39)

Prepared following **General Procedure D.** Purification by flash column chromatography (5:1:1 hexane/EtOAc/DCM) gave the title compound (15.1 mg, 35%, 94:6 dr, 98:2 er) as a colorless oil.

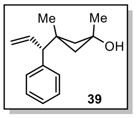

^{1}H NMR (400 MHz, $CDCl_3$): δ_H 7.31 – 7.29 (m, 2H), 7.24 – 7.17 (m, 3H), 6.17 (ddd, *J* = 17.0, 10.3, 8.9 Hz, 1H), 5.18 (ddd, *J* = 10.2, 1.9, 0.7 Hz, 1H), 5.11 (ddd, *J* = 17.0, 1.9, 1.0 Hz, 1H), 3.34 (d, *J* = 8.9 Hz, 1H), 2.28 (dd, *J* = 17.0, 12.7 Hz, 2H), 1.94 (ddd, *J* = 12.6, 3.0, 1.1 Hz, 1H), 1.79 (ddd, *J* = 12.6, 3.0, 1.2 Hz, 1H), 1.52 (s, 1H), 1.40 (s, 3H), 1.09 (s, 3H) ppm. **^{13}C NMR** (101 MHz, $CDCl_3$): δ_C 142.0, 138.3, 129.0, 128.2, 126.4, 117.3, 68.1, 60.4, 47.8, 47.7, 31.6, 30.6, 24.9 ppm. **Specific rotation** $[\alpha]_D^{22}$ = +39 (c = 0.56, CH_2Cl_2). **HRMS** (ESI) m/z calculated for $C_{15}H_{20}ONa$ $[M +Na]^+$, 239.1406 found, 239.1404. **IR (thin film)** ν_{max}/cm^{-1}: 3354, 2968, 2924, 1379, 1221, 1066, 916, 702.

(*1s*,*3s*)-3-Methyl-1-phenyl-3-((*R*)-1-phenylallyl)cyclobutan-1-ol (40)

Prepared following **General Procedure D.** Purification by flash column chromatography (5:1:1 hexane/EtOAc/DCM) gave the title compound (18.9 mg, 34%, >95:5 dr, 94.5:5.5 er) as a colorless oil.

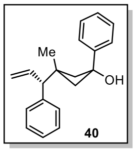

^{1}H NMR (400 MHz, $CDCl_3$): δ_H 7.49 – 7.42 (m, 2H), 7.39 – 7.20 (m, 8H), 6.24 (ddd, *J* = 17.0, 10.3, 8.9 Hz, 1H), 5.26 – 5.13 (m, 2H), 3.58 (d, *J* = 8.9 Hz, 1H), 2.64 – 2.46 (m, 3H), 2.39 – 2.30 (m, 1H), 1.86 (s, 1H), 0.95 (s, 3H) ppm. **^{13}C NMR** (101 MHz, $CDCl_3$): δ_C 147.1, 141.8, 138.0, 129.1, 128.7, 128.3, 127.5, 126.5, 125.6, 117.3, 72.0, 60.0, 46.8, 46.7, 33.1, 23.9 ppm. **Specific rotation** $[\alpha]_D^{22}$ = +34 (c = 0.71, CH_2Cl_2). **HRMS** (ESI) m/z calculated for $C_{20}H_{21}$ [M-H_2O+H]$^+$, 261.1638 found, 261.1639. **IR (thin film)** ν_{max}/cm^{-1}: 3383, 2971, 1494, 11378, 1055, 915, 700. **HPLC conditions**: Chiral column IC, hexane: isopropanol = 97:3, flow rate = 0.8 mL/min, wavelength = 254 nm, t_R = 11.3 min for major isomer, t_R = 10.4 min for minor isomer.

4,4,5,5-tetra-Methyl-2-((*1R*,*3r*)-1-(naphthalen-2-yl)-3-((*R*)-1-phenylallyl)cyclobutyl)-1,3,2-dioxaborolane (41)

Prepared following **Procedure E.** Purification by flash column chromatography (40:1 hexane/EtOAc) gave the title compound (1.28 g, 75%, 89:11 dr, 97:3 er) as a white soild.

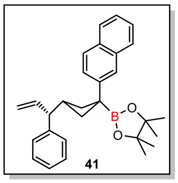

^{1}H NMR (400 MHz, $CDCl_3$): δ_H 7.81 – 7.73 (m, 3H), 7.63 (d, *J* = 1.8 Hz, 1H), 7.41 (dddd, *J* = 20.8, 8.1, 6.8, 1.4 Hz, 2H), 7.36 – 7.28 (m, 3H), 7.25 – 7.10 (m, 3H), 5.91 (ddd, *J* = 17.1, 10.3, 7.8 Hz, 1H), 5.13 – 5.06 (m, 1H), 5.09 – 5.04 (m, 1H), 3.45 – 3.37 (m, 1H), 2.69 – 2.62 (m, 2H), 2.59 – 2.53 (m, 1H), 2.44 – 2.29 (m, 2H), 1.19 (d, *J* = 2.3 Hz, 12H) ppm. **^{13}C NMR** (101 MHz, $CDCl_3$): δ_C 145.5, 143.0, 140.4, 133.7, 131.5, 128.5, 128.1, 127.7, 127.7, 127.6, 126.4, 126.1, 125.8, 124.9, 123.6, 115.0, 83.7, 55.9, 35.6, 35.5, 35.0, 24.7, 24.6 ppm. **^{11}B NMR** (128 MHz, $CDCl_3$): δ_B 33.7 ppm. **Specific rotation** $[\alpha]_D^{22}$ = +21 (c = 0.66, CH_2Cl_2). **HRMS** (EI) m/z calculated for $C_{29}H_{33}BO_2$ [M]$^+$, 431.1982 found, 424.2568. **IR (thin film)** ν_{max}/cm^{-1}: 2976, 1631, 1599, 1454, 1350, 1130, 857, 700.

(*1s*,*3s*)-3-((*R*)-3-Hydroxy-1-phenylpropyl)-1-(naphthalen-2-yl)cyclobutan-1-ol (42)

Prepared following **Procedure G.** Purification by flash column chromatography (3:1 hexane/EtOAc) gave the title compound (18.4 mg, 83% yield, >95:5 dr) as a colorless oil.

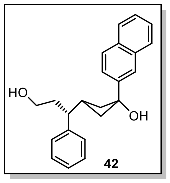

^{1}H NMR (400 MHz, $CDCl_3$) δ_H 7.89 (d, *J* = 1.9 Hz, 1H), 7.89 – 7.78 (m, 3H), 7.63 (dd, *J* = 8.6, 1.9 Hz, 1H), 7.54 – 7.43 (m, 2H), 7.34 – 7.25 (m, 2H), 7.25 – 7.12 (m, 3H), 3.53 (ddd, *J* = 10.5, 7.2, 5.0 Hz, 1H), 3.45 (ddd, *J* = 10.6, 7.9, 6.5 Hz, 1H), 2.97 (ddd, *J* = 12.0, 7.1, 5.2 Hz, 1H), 2.77 (td, *J* = 10.3, 4.0 Hz, 1H), 2.59 – 2.45 (m, 1H), 2.34 – 2.20 (m, 1H), 2.17 – 1.93 (m, 3H), 1.73 (dddd, *J* = 13.6, 11.2, 6.5, 5.0 Hz, 1H), 1.24 (s, 1H) ppm. **^{13}C NMR** (101 MHz, $CDCl_3$) δ_C 142.99, 142.96, 133.1, 132.8, 128.8, 128.7, 128.3, 127.9, 127.7, 126.6, 126.4, 126.2, 124.4, 123.4, 72.5, 61.2, 49.9, 42.7, 41.9, 36.6, 31.6 pm. **Specific rotation** $[\alpha]_D^{22}$ = +78 (c = 0.75, CH_2Cl_2). **HRMS** (ESI) m/z calculated for $C_{23}H_{324}O_2$ [M]$^+$, 315.1743 found, 315.1749. **IR (thin film)** ν_{max}/cm^{-1}: 3337, 3056, 2970, 2928, 1600, 1452, 1023, 747, 702.

2-((*1s*,*3s*)-1-(Naphthalen-2-yl)-3-((*R*)-1-phenylallyl)cyclobutyl)furan (43)

Prepared following **Procedure H.** Purification by flash column chromatography (20:1 hexane/EtOAc) gave the title compound (56.9 mg, 78%, >95:5 dr) as a colorless oil.

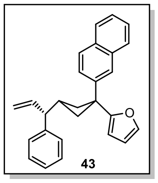

^{1}H NMR (400 MHz, $CDCl_3$) δ_H 7.80 – 7.75 (m, 1H), 7.76 – 7.67 (m, 3H), 7.42 – 7.31 (m, 3H), 7.23 (dd, *J* = 1.9, 0.9 Hz, 1H), 7.23 – 7.14 (m, 2H), 7.15 – 7.07 (m, 1H), 7.11 – 7.03 (m, 2H), 6.11 (dd, *J* = 3.2, 1.8 Hz, 1H), 5.81 (ddd, *J* = 17.1, 10.3, 7.6 Hz, 1H), 5.57 (dd, *J* = 3.2, 0.9 Hz, 1H), 5.04 – 4.92 (m, 2H), 3.29 (dd, *J* = 9.3, 7.7 Hz, 1H), 2.90 (ddd, *J* = 11.6, 7.6, 4.7 Hz, 1H), 2.65 – 2.54 (m, 2H), 2.55 – 2.39 (m, 2H) ppm. **^{13}C NMR** (101 MHz, $CDCl_3$) δ_C 161.7, 142.5, 141.5, 139.9, 133.3, 132.2, 128.6, 128.1, 128.1, 128.0, 127.7, 126.5, 126.2, 125.9, 125.2, 115.0, 110.2, 105.2, 56.8, 42.7, 38.9, 38.4, 33.7 ppm. **Specific rotation** $[\alpha]_D^{22}$ = +25 (c = 1.89, CH_2Cl_2). **HRMS** (EI) m/z calculated for $C_{27}H_{24}O$ [M]$^+$, 364.1822 found, 364.1821. **IR (thin film)** ν_{max}/cm^{-1}: 3046, 2976, 2937, 1634, 1600, 1502, 1452, 816, 737, 700.

2-((*1R*,*3r*)-3-((*R*)-1-Phenylallyl)-1-vinylcyclobutyl)naphthalene (44)

Prepared following **Procedure I.** Purification by flash column chromatography (20:1 hexane/EtOAc) gave the title compound (54.1 mg, 84%, >95:5 dr) as a colorless oil.

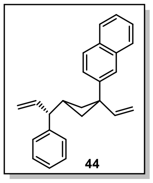

44

^{1}H NMR (400 MHz, $CDCl_3$) δ_H 7.86 – 7.76 (m, 4H), 7.53 – 7.38 (m, 3H), 7.36 – 7.25 (m, 2H), 7.26 – 7.13 (m, 3H), 6.12 (dd, *J* = 17.2, 10.3 Hz, 1H), 5.98 – 5.84 (m, 1H), 5.09 (ddd, *J* = 7.3, 1.8, 1.1 Hz, 1H), 5.05 (d, *J* = 1.1 Hz, 1H), 4.98 (dd, *J* = 10.3, 1.2 Hz, 1H), 4.86 (dd, *J* = 17.1, 1.3 Hz, 1H), 3.37 – 3.24 (m, 1H), 2.90 – 2.79 (m, 1H), 2.64 – 2.47 (m, 2H), 2.36 – 2.26 (m, 1H), 2.21 – 2.12 (m, 1H) ppm. **^{13}C NMR** (101 MHz, $CDCl_3$) δ_C 147.9, 144.0, 142.6, 140.1, 133.4, 132.0, 128.6, 128.1, 128.0, 127.9, 127.6, 126.5, 126.3, 126.1, 125.6, 124.5, 114.9, 111.3, 57.1, 45.8, 38.5, 37.9, 33.8 ppm. **Specific rotation** $[\alpha]_D^{22}$ = +50 (c = 1.05, CH_2Cl_2). **HRMS** (ESI) m/z calculated for $C_{25}H_{24}$ $[M]^+$, 324.1873 found, 324.1871. **IR (thin film)** ν_{max}/cm^{-1}: 3066, 2971, 2928, 1632, 1600, 1451, 992, 911, 746, 700.

(*1s*,*3s*)-3-((*R*)-3-Hydroxy-1-phenylpropyl)-1,3-dimethylcyclobutan-1-ol (S3)

Prepared following **Procedure J** with alcohol **39** (0.053 mmol). Purification by flash column chromatography (1:1 hexane/EtOAc) gave the title compound (8.6 mg, 70%) as a colorless oil.

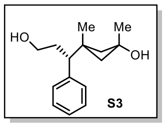

S3

^{1}H NMR (400 MHz, $CDCl_3$): δ_H 7.31 – 7.27 (m, 2H), 7.24 – 7.18 (m, 3H), 3.53 – 3.53 (m, 1H), 3.43 – 3.41 (m, 1H), 2.84 (dd, *J* = 11.5, 3.4 Hz, 1H), 2.16 (t, *J* = 13.3 Hz, 2H), 2.06 – 1.85 (m, 3H), 1.70 (s, 1H), 1.64 (s, 1H), 1.61 (ddd, *J* = 12.6, 2.9, 1.1 Hz, 1H), 1.39 (s, 3H), 1.06 (s, 3H) ppm. **^{13}C NMR** (101 MHz, $CDCl_3$): δ_C 141.1, 129.4, 128.2, 126.6, 67.9, 61.9, 53.1, 49.1, 47.8, 32.2, 31.9, 30.7, 23.1 ppm. **Specific rotation** $[\alpha]_D^{22}$ = +22 (c = 0.47, CH_2Cl_2). **HRMS** (ESI) m/z calculated for $C_{15}H_{22}O_2Na$ $[M +Na]^+$, 257.1512 found, 257.1505. **IR (thin film)** ν_{max}/cm^{-1}: 3330, 2958, 2924, 1452, 1378, 1621, 1043, 964, 726, 703. **HPLC conditions**: Chiral column IC, hexane: isopropanol = 90:0, flow rate = 1.0 mL/min, wavelength = 254 nm, t_R = 14.6 min for major isomer, t_R = 9.6 min for minor isomer.

(*1s*,*3s*)-1-(tert-Butyl)-3-((*R*)-3-hydroxy-1-phenylpropyl)cyclobutan-1-ol (S4)

Prepared following **Procedure J** with alcohol **35** (0.053 mmol). Purification by flash column chromatography (1:1 hexane/EtOAc) gave the title compound (15.7 mg, 60%) as a colorless oil.

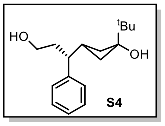

S4

^{1}H NMR (400 MHz, $CDCl_3$): δ_H 7.33 – 7.24 (m, 2H), 7.24 – 7.17 (m, 1H), 7.18 – 7.14 (m, 2H), 3.54 – 3.49 (m, 1H), 3.46 – 3.40 (m, 1H), 2.72 – 2.53 (m, 2H), 2.20 – 2.08 (m, 1H), 2.03 – 1.86 (m, 2H), 1.77 – 1.63 (m, 2H), 1.52 – 1.44 (m, 1H), 0.89 (s, 9H) ppm. **^{13}C NMR** (101 MHz, $CDCl_3$): δ_C 143.3, 128.6, 128.0, 126.5, 76.5, 61.3, 50.7, 37.9, 37.01, 36.7, 35.6, 31.2, 23.9 ppm. **Specific rotation** $[\alpha]_D^{22}$ = -7.3 (c = 1.37, CH_2Cl_2). **HRMS** (ESI) m/z calculated for $C_{17}H_{25}O$ $[M-H_2O+H]^+$, 245.1900 found, 245.1900. **IR (thin film)** ν_{max}/cm^{-1}: 3371, 2960, 1453, 1393, 1246, 1053, 896, 701. **HPLC conditions**: Chiral column IA, hexane: isopropanol = 90:10, flow rate = 0.8 mL/min, wavelength = 254 nm, t_R = 16.4 min for major isomer, t_R = 20.2 min for minor isomer.

(*1s*,*3s*)-1-(3-Bromophenyl)-3-((*R*)-3-hydroxy-1-phenylpropyl)cyclobutan-1-ol (S5)

Prepared following **Procedure J** with alcohol **22** (0.050 mmol). Purification by flash column chromatography (1:1 hexane/EtOAc) gave the title compound (14.6 mg, 81%) as a colorless oil.

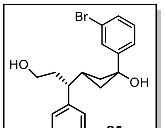

S5

^{1}H NMR (400 MHz, $CDCl_3$): δ_H 7.64 – 7.63 (m, 1H), 7.45 – 7.37 (m, 2H), 7.34 – 7.25 (m, 2H), 7.27 – 7.12 (m, 4H), 3.59 – 3.48 (m, 1H), 3.50 – 3.36 (m, 1H), 2.85 – 2.79 (m, 1H), 2.74 – 2.71 (m, 1H), 2.41 – 2.30 (m, 1H), 2.23 – 2.13 (m, 1H), 2.14 – 2.02 (m, 2H), 2.04 – 1.91 (m, 2H), 1.77 – 1.68 (m, 1H), 1.58 (s, 1H) ppm. **^{13}C NMR** (101 MHz, $CDCl_3$): δ_C 148.5, 142.7, 130.6, 130.3, 128.8, 128.7, 127.9, 126.7, 124.0, 122.9, 72.1, 61.1, 49.9, 42.9, 42.2, 36.5, 31.4 ppm. **Specific rotation** $[\alpha]_D^{22}$ = +40 (c = 0.45, CH_2Cl_2). **HRMS** (ESI) m/z calculated for $C_{19}H_{21}O_2BrNa$ $[M+Na]^+$, 383.0617 found, 383.0609. **IR (thin film)** ν_{max}/cm^{-1}: 3336, 2928, 1566, 1600, 1410, 1237, 1050, 784, 700. **HPLC conditions**: Chiral column IA, hexane: isopropanol = 90:10, flow rate = 1.0 mL/min, wavelength = 254 nm, t_R = 17.9 min for major isomer, t_R = 13.1 min for minor isomer.

3. COMPUTATIONAL DETAILS

3.1. General Information

The hybrid density functional B3LYP,[1] in conjunction with Los Alamos LANL2DZ basis set[2] for iridium and Pople's double-ζ 6-31+G(d) basis set[3] for the remaining elements, was used to optimize all stationary point structures. Single-point energy corrections were applied to these optimized structures using the hybrid meta-GGA M06 functional[4] in conjunction with the SDD basis set[5] for iridium and Pople's triple-ζ 6-311+G(d,p) basis set for the remaining elements. For both optimization and single point calculations, the integral equation formalism variant of the polarizable continuum model (IEF-PCM), with the SMD solvation model, was applied to account for solvent effects (solvent=dichloromethane).[6] This level of theory was chosen based on a previous study by Ready and Liu[7] in the study of the allylation of vinyl boronate esters, which proved reliable when compared to experimental observations. Further benchmarking was conducted on the BCB boronate, and the results, presented in **Table S5**, revealed minimal structural differences across six distinct levels of theory. Notably, the key internal C-C bond exhibited a narrow range of 1.49-1.51 Å, while all other bond lengths and dihedrals displayed very little variation across all levels of theory. Additional studies on the reaction on the reaction with tropylium ion were performed at the ωB97X-D[8]/def2-TZVPP[9]//ωB97x-D/6-31+G(d) level of theory in conjunction with the SMD solvation model for THF. For the model system, IRC calculations were performed on the transition structures (TSs) to confirm the identity of the local minima they connect.[10] *Gaussian 16*[11] revision C.01 was employed for all density functional theory (DFT) calculations, using an "ultrafine" pruned (99,590) grid for numerical integration of the exchange-correlation functional and its derivatives. Conformational analyses of all stationary points were performed manually: low energy conformations of ground and transition state structures were located through systematic dihedral rotations about rotatable single bonds and forming bonds. Input files for single-point energy corrections were generated automatically using the *qprep* function in AQME[12]. Molecular graphics were generated using *PyMol.*[13] Vibrational frequency calculations were performed to verify that stationary points were either minima or first-order saddle points on the potential energy surface (PES) and to calculate thermal corrections to Gibbs free energies (G). The computed thermochemistry data were corrected following Grimme's quasi-harmonic (QHA) model for entropy[14] with a frequency cut-off value of 100.0 cm^{-1} using the *GoodVibes*[15] program at 333.15 K (60˚C) unless otherwise stated. Also, 1 M standard state concentration corrections were applied to all individual calculations to account for reactions in solution (i.e., a correction from 1 atm to 1 M)[16]. NCIPLOT was used to visualize noncovalent interactions from promolecular densities.[17]

Entry	Functional	BCB-*trans* d(C–C)	BCB-*cis* d(C–C)	Overlay-*trans*
1	B3LYP	1.51	1.50	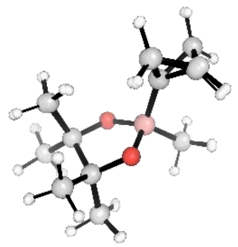
2	M06	1.49	1.49	
3	*wB97XD*	1.50	1.49	
4	TPSS	1.52	1.51	
5	PBE	1.52	1.51	
6	M06L	1.51	1.49	

Table S5: Structural benchmarking of the BCB-boronate with different functionals. All calculations were performed using the 6-31+G(d) functional in conjuctions with the SMD=dichloromethane solvation model

3.2. Transition Structures for the Allylation Reaction

The absolute sense of enantiocontrol in Ir(phosphoramidite)-catalyzed allylations with racemic allyl carbonates has been investigated computationally: oxidative addition occurs preferentially to one enantiomer (i.e., a kinetic resolution) to form a cationic π-allyl-Ir(**III**) complex in high enantioselectivity.[17] In this work, our computational studies focused on the origins of reactivity and diastereoselectivity in the allylation of bicyclobutyl boronates. For the reaction of the prototypical bicyclobutyl boronate substrate with a methyl group on the boron (**3b**), we were able to identify 8 TS conformations for the major diastereomer of the allyl iridium complex **Ir-2** that led to the *trans*-product and 4 TS conformations that led to the *cis*-product (**Figure S3**). Equilibration of the boronate ester was studied via rotation about the C–B bond connecting the boronate group and the bicyclobutane motif of substrate **3b**. The rotational barrier was found to be 3.3 kcal/mol, similar to the activation energy barrier for the reaction with the allyl iridium complex. While rapid equilibration is challenging to assess based on the small energy differences between the two processes, it is important to consider the rate of formation of the allyl-iridium complex. Based on computational work from Ready and Liu,[17] the activation energy barrier for the formation of allyl-iridium (**Ir-2**) electrophile is estimated to be 17.6 kcal/mol (in THF). As such, due to the large energetic requirement to generate the reactive catalytic species **Ir-2**, the equilibration of rotational conformers of substrate **3b** can be easily established as the two events (formation of allyl iridium and bond rotation) are independent of one another.

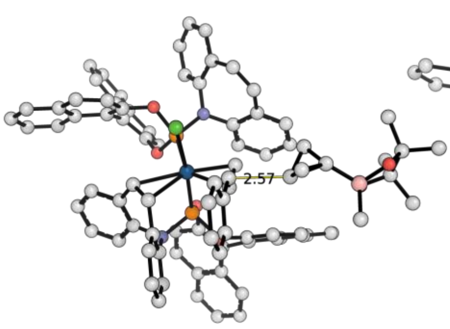

TS-trans_a
1.15

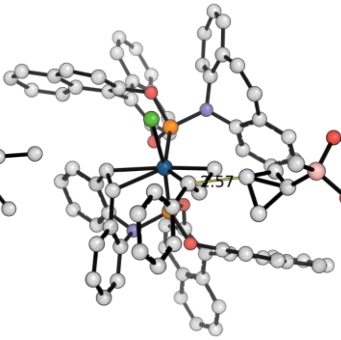

TS-trans_b
0.04

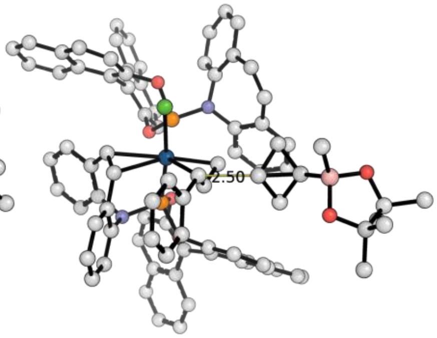

TS-trans_c
1.64

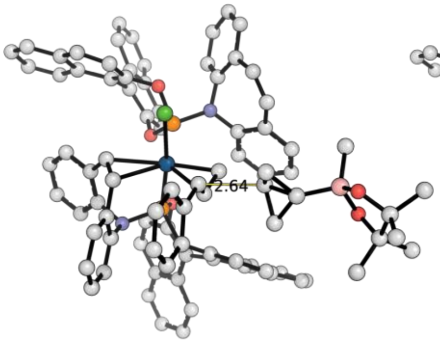

TS-trans_d
1.38

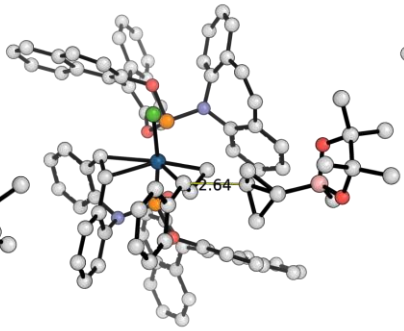

TS-trans_e
0.00

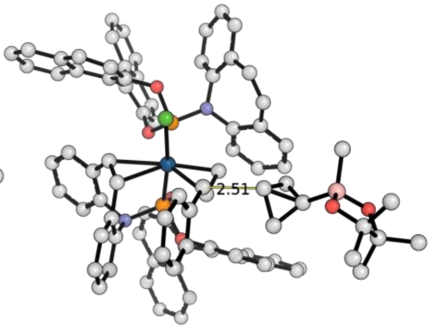

TS-trans_f
0.27

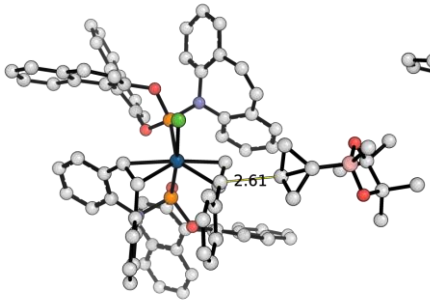

TS-cis_a
2.19

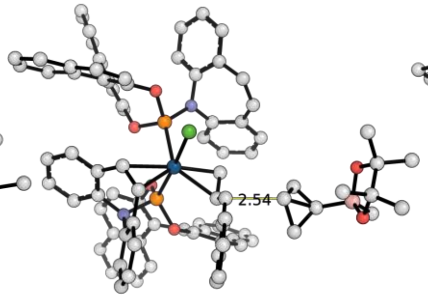

TS-cis_b
1.12

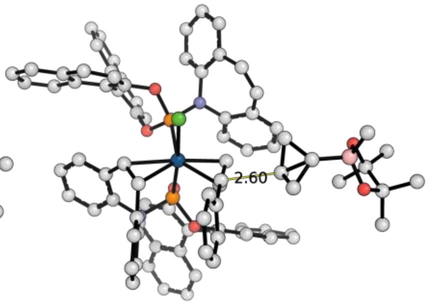

TS-cis_c
2.30

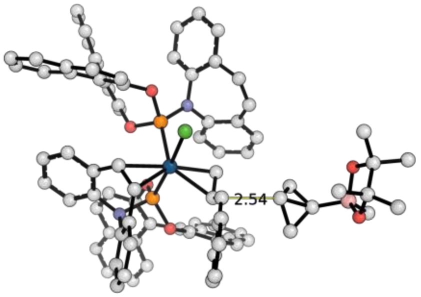

TS-cis_d
1.11

Figure S3: DFT optimized competing allylation TSs. M06/6-311+G(d,p)–SDD(Ir)//B3LYP/6-31+G(d)–LANL2DZ(Ir) relative Gibbs energies (298K) shown in kcal/mol. Forming C–C distance shown in Å.

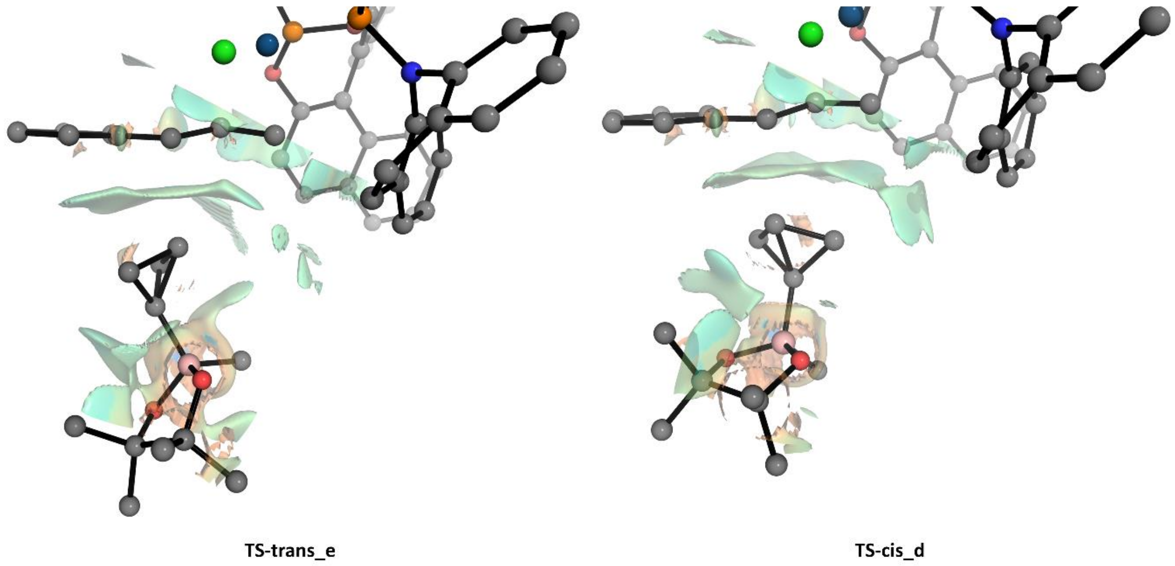

Figure S4: Noncovalent interaction plot (from promolecular densities) of the most energetically favorable allylation TSs (H atoms not shown). Intermolecular interactions between nucleophile and electrophile are shown, with weak dispersive interactions in green. Based on a visual comparison of the competing diastereomeric TSs, the greater stability of the *trans*-TS does cannot be attributed to more favorable dispersion interactions.

3.3. Rotational Barriers for BCB-Boronate Complexes

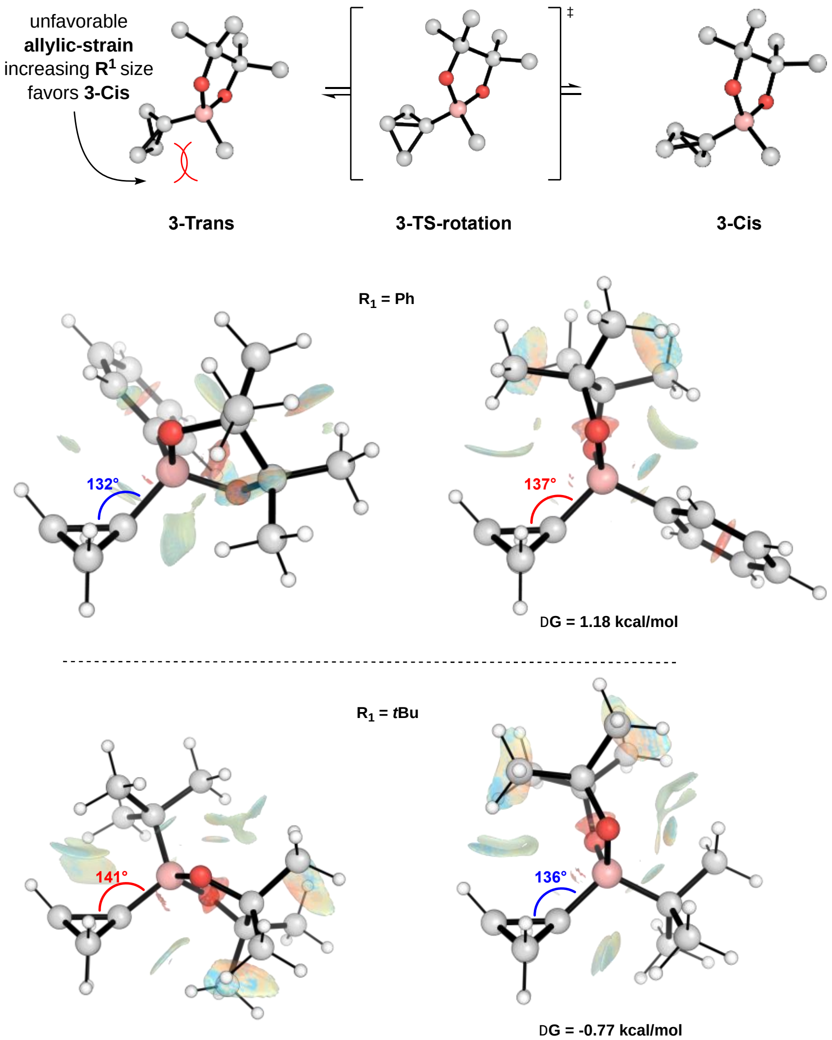

Figure S5: Equilibration of boronate ester conformers via C–B bond rotation. Smaller alkyl and aryl B-substituents result in the *trans*-conformation being preferred (LHS) while larger alkyl substituents result in the *cis*-conformation being favored (RHS).

Entry	R^1	R^2	dr (*expt.*)	ΔG(DFT) / kcal·mol^{-1}	boronate structure
1	Me	H	90:10	1.18	
2	Ph	H	86:4	1.26	
3	*t*Bu	H	65:35	-0.77	
4	cyclohexyl	H	63:37	-0.84	
5	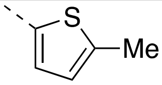	H	86:14	1.58	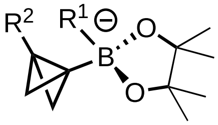
6	Me	Me	94:6	2.17	
7	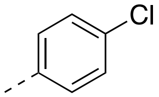	H	91:9	1.14	
8	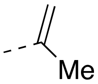	H	87:13	0.97	

9	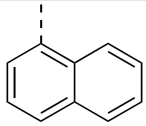	H	86:14	1.35	

Table S6: Computed equilibrium conformational distribution for a series of boronate esters

We have computed the ΔG values for systems with varying boronate groups where a B-phenyl substituent is present (below), which also reflect the experimental trend in diastereoselectivity obtained**.**

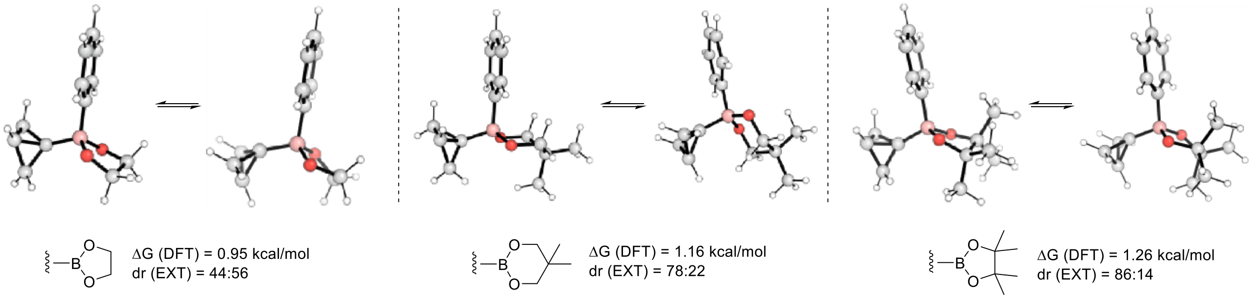

Figure S6: Calculated energy differences of ground state conformations of the B-Ph boronate complexes and relationship to experimentally observed diastereoselectivity.

3.4. Reaction of BCB-Boronate Complexes with the Tropylium Ion

Due to the considerable computational expense associated with computing the potential energy surface for the reaction between allyl-iridium complex **Ir-2** and the boronates, we opted to study the full potential energy surface (PES) for a model electrophile. To this extent, we chose the reaction with the tropylium ion as a model substrate. We modeled the reaction between the tropylium ion and bicyclobutyl boronate ester (**SM-tropylium**) at the ωB97x-D/def2-TZVPP//ωB97x-D/6-31+G(d) level of theory in conjunction with the SMD solvation model for THF. Gibbs free energies were obtained at 298.15 K (25˚C) (**Figure S6**). The TS leading to the formation of the *cis*-product was found to require an activation energy barrier of 6.0 kcal/mol, while the *trans*-pathway was preferred, only requiring an activation energy barrier of 5.2 kcal/mol. Compared to the reaction with an allyl-iridium electrophile, these TS geometries are later (i.e., a shorter forming C–C bond; 2.30-2.37 Å for the forming C–C bond) with larger activation energy barriers. IRC calculations performed from the *cis* TS were found to lead directly to the experimentally observed product in a concerted asynchronous process. There is no stable minimum on the PES corresponding to a zwitterionic intermediate, although the IRC shows a relatively flat surface after the TS (**Figures S7** and **S8**). On the other hand, the *trans*-TS was found to lead to a stable local minimum corresponding to zwitterion (**INT-tropylium-*trans***). However, 1,2-migration of the methyl group was found to proceed very rapidly with a subsequent barrier of just 0.3 kcal/mol leading to the *trans*-product. Therefore, we predict that C–C bond formation between the BCB and the electrophile is diastereoselectivity-determining for both products.

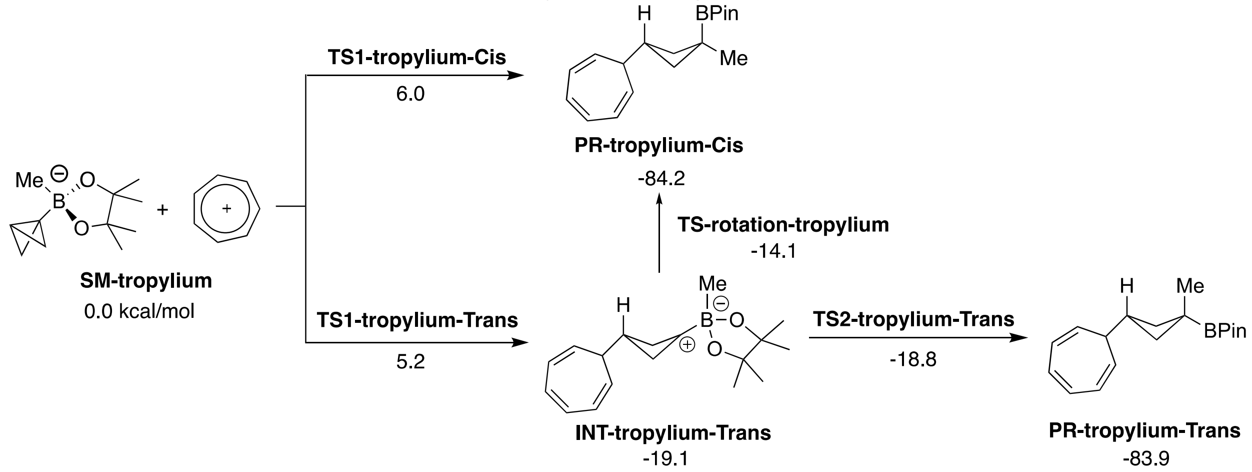

Figure S7: Proposed reaction mechanism for the reaction with tropylium ion

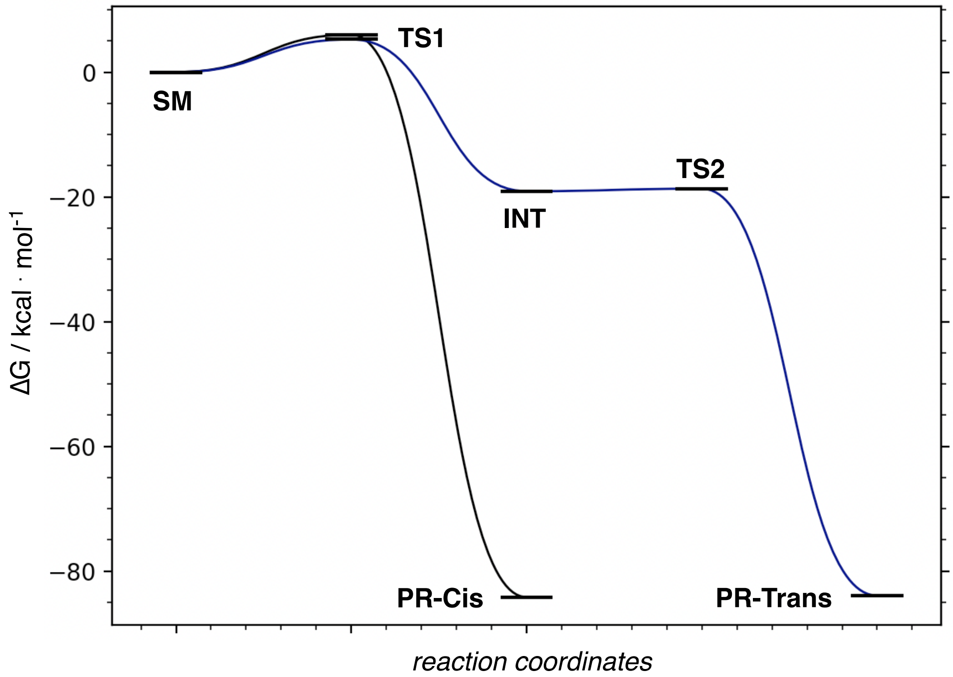

Figure S8: ωB97x-D/def2-TZVPP//ωB97x-D/6-31+G(d) Gibbs free energy surface (298K, 1M) for the reaction with the tropylium cation.

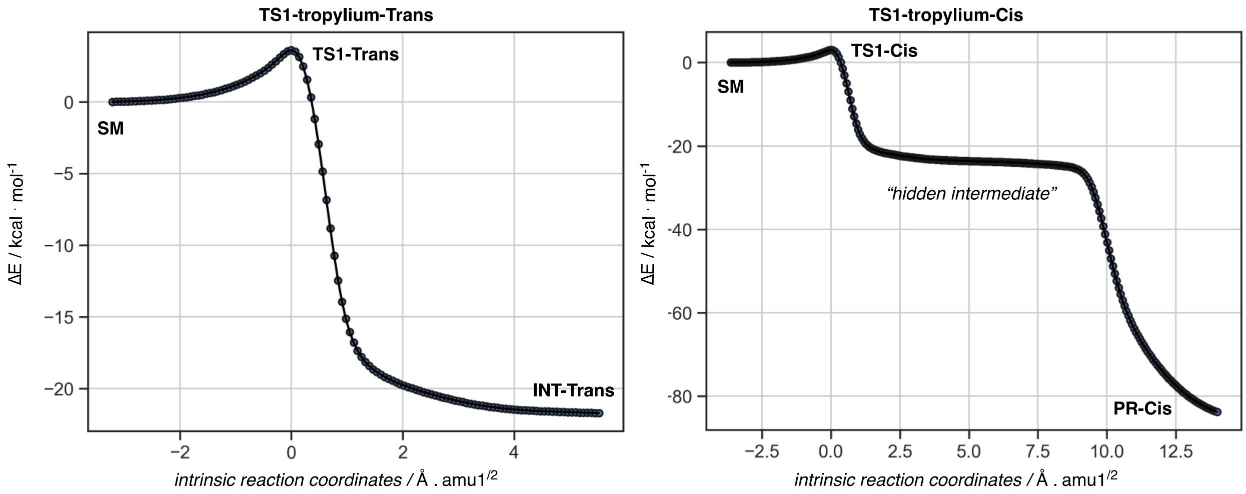

Figure S9: ωB97x-D/6-31+G(d) intrinsic reaction coordinates computed for **TS1-tropylium-*trans*** (LHS) and **TS1-tropylium-*cis*** (RHS).

3.5. Thermochemical Data

Legend:
E_{SPC} = energy obtained in the single-point energy corrections
E = energy obtained in the geometry optimizations
ZPE = zero-point energy
H_{SPC} = enthalpy corrected with E_{SPC}
T·S = temperature times entropy with no correction
T·qh-S = temperature times entropy with quasi-harmonic S correction
G(T) = Gibbs free energy corrected only with E_{SPC}
qh-G(T) = Gibbs free energy with E_{SPC} and quasi-harmonic S correction
ν_{imag} = imaginary frequencies

Name	E_{SPC}	E	ZPE	H_{SPC}	T·S	T·qh-S	G(T)	qh-G(T)	ν_{imag} / cm^{-1}
**SM-CIS_a**	-5228.635116	-5230.801841	1.385484	-5227.141492	0.261789	0.233158	-5227.403281	-5227.374650	
**SM-CIS_b**	-5228.640253	-5230.802748	1.385413	-5227.146781	0.259490	0.232069	-5227.406270	-5227.378849	
**SM-CIS_c**	-5228.641149	-5230.804732	1.386071	-5227.147209	0.256897	0.230677	-5227.404107	-5227.377886	
**SM-TRANS_a**	-5228.639878	-5230.803057	1.385428	-5227.146329	0.258067	0.231540	-5227.404396	-5227.377869	
**SM-TRANS_b**	-5228.639211	-5230.801733	1.385347	-5227.145639	0.260997	0.232793	-5227.406636	-5227.378431	
**TS-CIS_a**	-5228.634115	-5230.794836	1.384418	-5227.142410	0.254318	0.229130	-5227.396727	-5227.371540	-143.4
**TS-CIS_b**	-5228.635969	-5230.795347	1.384677	-5227.144059	0.254490	0.229191	-5227.398549	-5227.373249	-168.21
**TS-CIS_c**	-5228.634129	-5230.794803	1.384489	-5227.142428	0.254024	0.228939	-5227.396452	-5227.371367	-148.45
**TS-CIS_d**	-5228.635971	-5230.795347	1.384675	-5227.144061	0.254502	0.229197	-5227.398562	-5227.373258	-168.16
**TS-TRANS_a**	-5228.635988	-5230.794503	1.384460	-5227.144271	0.253947	0.228930	-5227.398218	-5227.373201	-154.36
**TS-TRANS_b**	-5228.636642	-5230.794832	1.384314	-5227.144967	0.256270	0.230007	-5227.401237	-5227.374974	-142.28
**TS-TRANS_c**	-5228.635929	-5230.795976	1.384995	-5227.143865	0.253204	0.228551	-5227.397069	-5227.372415	-177.61
**TS-TRANS_d**	-5228.635837	-5230.796916	1.384676	-5227.144113	0.253589	0.228717	-5227.397703	-5227.372830	-104.64
**TS-TRANS_e**	-5228.637284	-5230.797904	1.384299	-5227.145650	0.254731	0.229381	-5227.400381	-5227.375031	-98.83
**TS-TRANS_f**	-5228.636680	-5230.796153	1.384392	-5227.144990	0.255192	0.229613	-5227.400182	-5227.374603	-170.69
**3b-Cis_c**	-606.393170	-606.632346	0.286870	-606.085814	0.064490	0.063326	-606.150304	-606.149140	
**3b-TS-rotation_a**	-606.389763	-606.628374	0.286561	-606.083542	0.062362	0.061615	-606.145904	-606.145157	-52.33
**3b-TS-rotation_b**	-606.390682	-606.629006	0.286548	-606.084485	0.062310	0.061554	-606.146795	-606.146040	-60.96
**3b-Trans_a**	-606.394041	-606.632253	0.286501	-606.086857	0.065525	0.063876	-606.152382	-606.150734	
**3b-Trans_b**	-606.395280	-606.633548	0.286930	-606.087839	0.064651	0.063369	-606.152490	-606.151208	
**3c-Cis_d**	-645.685405	-645.948498	0.313743	-645.348945	0.070754	0.068159	-645.419699	-645.417103	

**3c-TS-rotation_a**	-645.684986	-645.946844	0.313988	-645.349481	0.066007	0.065271	-645.415488	-645.414751	-35.24
**3c-TS-rotation_b**	-645.685456	-645.947056	0.313907	-645.349992	0.066303	0.065371	-645.416294	-645.415363	-27.85
**3c-Trans_a**	-645.689004	-645.950516	0.314324	-645.352258	0.068919	0.067223	-645.421178	-645.419482	
**3c-Trans_b**	-645.690253	-645.951632	0.314210	-645.353587	0.068926	0.067231	-645.422513	-645.420818	
**INT-tropylium-trans_a**	-877.496803	-877.201674	0.419831	-877.054609	0.068921	0.065676	-877.123530	-877.120285	
**INT-tropylium-trans_b**	-877.494467	-877.199320	0.419726	-877.052325	0.069429	0.065943	-877.121755	-877.118268	
**INT-tropylium-trans_c**	-877.496942	-877.201756	0.419056	-877.055414	0.069495	0.066049	-877.124909	-877.121463	
**PR-tropylium-Cis_a**	-877.604245	-877.308731	0.424248	-877.158222	0.069049	0.065143	-877.227271	-877.223364	
**PR-tropylium-Cis_b**	-877.604743	-877.309091	0.423939	-877.159118	0.068257	0.064724	-877.227375	-877.223841	
**PR-tropylium-Cis_c**	-877.601294	-877.305661	0.424282	-877.155177	0.069691	0.065511	-877.224868	-877.220688	
**PR-tropylium-Cis_d**	-877.605684	-877.310189	0.423656	-877.160237	0.069141	0.065197	-877.229378	-877.225434	
**PR-tropylium-Cis_e**	-877.604358	-877.308663	0.423931	-877.158739	0.068369	0.064802	-877.227108	-877.223541	
**PR-tropylium-Trans_a**	-877.602710	-877.307163	0.424053	-877.156895	0.068897	0.065077	-877.225793	-877.221972	
**PR-tropylium-Trans_b**	-877.599854	-877.303989	0.424296	-877.153692	0.069625	0.065472	-877.223317	-877.219164	
**PR-tropylium-Trans_c**	-877.600322	-877.304511	0.424312	-877.154130	0.070329	0.065778	-877.224459	-877.219908	
**PR-tropylium-Trans_d**	-877.604851	-877.309321	0.423568	-877.159447	0.069543	0.065511	-877.228990	-877.224958	
**PR-tropylium-Trans_e**	-877.604480	-877.308896	0.423891	-877.158883	0.068703	0.064875	-877.227586	-877.223758	
**PR-tropylium-Trans_f**	-877.604175	-877.308485	0.424138	-877.158439	0.067622	0.064334	-877.226061	-877.222773	
**SM-tropylium_cis_b**	-877.460665	-877.163486	0.416811	-877.020063	0.074894	0.069480	-877.094957	-877.089544	
**SM-tropylium_cis_c**	-877.460681	-877.163483	0.416758	-877.020124	0.074304	0.069343	-877.094428	-877.089467	
**SM-tropylium_trans_b**	-877.462669	-877.165538	0.416878	-877.022041	0.074060	0.069077	-877.096101	-877.091118	
**TS1-tropylium-Cis_a**	-877.450739	-877.154730	0.416696	-877.011334	0.070553	0.066742	-877.081888	-877.078076	-428.28
**TS1-tropylium-Cis_b**	-877.453542	-877.157378	0.416547	-877.014342	0.069820	0.066434	-877.084162	-877.080777	-356.22
**TS1-tropylium-Cis_c**	-877.453782	-877.157638	0.416520	-877.014551	0.070565	0.066842	-877.085115	-877.081393	-342.85
**TS1-tropylium-Trans_a**	-877.451696	-877.155804	0.416815	-877.012289	0.069606	0.066202	-877.081894	-877.078490	-459.89
**TS1-tropylium-Trans_b**	-877.454850	-877.158831	0.416555	-877.015600	0.070592	0.066754	-877.086192	-877.082354	-380.1
**TS1-tropylium-Trans_c**	-877.454850	-877.158831	0.416554	-877.015601	0.070604	0.066760	-877.086204	-877.082360	-380.13
**TS2-tropylium-Trans_a**	-877.496001	-877.201173	0.418430	-877.055738	0.067675	0.064549	-877.123412	-877.120287	-128.2
**TS2-tropylium-Trans_b**	-877.494694	-877.199752	0.418671	-877.054239	0.067700	0.064582	-877.121939	-877.118821	-90.65
**TS2-tropylium-Trans_c**	-877.494681	-877.199738	0.418563	-877.054315	0.067762	0.064641	-877.122077	-877.118956	-88.22
**TS2-tropylium-Trans_d**	-877.496513	-877.201633	0.418078	-877.056652	0.067390	0.064449	-877.124042	-877.121101	-105.54
**TS-rotation-tropylium_a**	-877.48934	-877.19371	0.420052	-877.04791	0.065697	0.06318	-877.11361	-877.11109	-56.22
**TS-rotation-tropylium_b**	-877.48707	-877.19146	0.41991	-877.04573	0.066019	0.063487	-877.11175	-877.10922	-57.23
**TS-rotation-tropylium_c**	-877.48671	-877.19111	0.420029	-877.04531	0.065599	0.063327	-877.11091	-877.10863	-41.24

**TS-rotation-tropylium_d**	-877.48919	-877.19351	0.419294	-877.04846	0.065832	0.063387	-877.11429	-877.11185	-46.09
**TS-rotation-tropylium_e**	-877.48712	-877.19108	0.419998	-877.04595	0.064787	0.062796	-877.11073	-877.10874	-31.35
**TS-rotation-tropylium_f**	-877.48694	-877.19093	0.419944	-877.04579	0.064959	0.062916	-877.11075	-877.10871	-38.97
**CIS_2MeThiofuranBoryl_a**	-1158.135815	-1158.463769	0.334212	-1157.775316	0.079422	0.076004	-1157.854738	-1157.851320	
**CIS_2MeThiofuranBoryl_b**	-1158.135937	-1158.463866	0.334248	-1157.775434	0.079220	0.075947	-1157.854655	-1157.851381	
**CIS_4ClPhBoryl_a**	-1257.635658	-1257.984267	0.330295	-1257.279254	0.079637	0.075922	-1257.358891	-1257.355176	
**CIS_CyBoryl_a**	-801.648009	-801.992730	0.407568	-801.214398	0.077504	0.074731	-801.291902	-801.289130	
**CIS_CyBoryl_d**	-801.651530	-801.995880	0.407302	-801.218077	0.077792	0.074934	-801.295869	-801.293010	
**CIS_CyBoryl_e**	-801.649100	-801.992831	0.407299	-801.215611	0.078278	0.075113	-801.293889	-801.290724	
**CIS_EneBoryl_a**	-683.751573	-684.033487	0.319958	-683.408306	0.071540	0.069311	-683.479846	-683.477617	
**CIS_EneBoryl_b**	-683.751839	-684.033905	0.319992	-683.408523	0.071422	0.069305	-683.479945	-683.477828	
**CIS_Me2Boryl_d**	-645.685404	-645.948498	0.313742	-645.348945	0.070745	0.068155	-645.419691	-645.417100	
**CIS_MeBoryl_c**	-606.393170	-606.632346	0.286871	-606.085814	0.064486	0.063322	-606.150300	-606.149136	
**CIS_NapBoryl_a**	-951.599588	-952.030329	0.386057	-951.185802	0.081203	0.078240	-951.267005	-951.264041	
**CIS_NapBoryl_b**	-951.599128	-952.029910	0.385651	-951.185507	0.082738	0.078925	-951.268245	-951.264432	
**CIS_Ph2ClBoryl_a**	-1257.635209	-1257.981025	0.330181	-1257.278940	0.079125	0.075640	-1257.358065	-1257.354580	
**CIS_Ph2ClBoryl_b**	-1257.636148	-1257.981681	0.330300	-1257.279818	0.078426	0.075361	-1257.358244	-1257.355180	
**CIS_PhBoryl_a**	-798.037016	-798.383019	0.339713	-797.672749	0.075211	0.072148	-797.747960	-797.744897	
**CIS_tBuBoryl_a**	-724.265468	-724.565231	0.369573	-723.870240	0.076112	0.073499	-723.946351	-723.943738	
**TRANS_2MeThiofuranBoryl_b**	-1158.138341	-1158.465338	0.333946	-1157.778012	0.079801	0.076214	-1157.857812	-1157.854225	
**TRANS_2MeThiofuranBoryl_c**	-1158.138154	-1158.465204	0.334130	-1157.777693	0.079535	0.076034	-1157.857229	-1157.853728	
**TRANS_2MeThiofuranBoryl_d**	-1158.137723	-1158.464085	0.334312	-1157.777204	0.078988	0.075777	-1157.856192	-1157.852981	
**TRANS_4ClPhBoryl_a**	-1257.637590	-1257.985007	0.330501	-1257.281083	0.079252	0.075628	-1257.360335	-1257.356711	
**TRANS_4ClPhBoryl_b**	-1257.637806	-1257.985617	0.330280	-1257.281441	0.079209	0.075724	-1257.360650	-1257.357165	
**TRANS_CyBoryl_a**	-801.647209	-801.990868	0.407169	-801.213773	0.078556	0.075259	-801.292329	-801.289032	
**TRANS_CyBoryl_c**	-801.648510	-801.992977	0.407279	-801.215068	0.078040	0.074995	-801.293108	-801.290063	
**TRANS_CyBoryl_d**	-801.650160	-801.993930	0.407208	-801.216732	0.078015	0.075055	-801.294747	-801.291787	
**TRANS_EneBoryl_a**	-683.752807	-684.033110	0.320205	-683.409391	0.070975	0.069015	-683.480367	-683.478407	
**TRANS_EneBoryl_b**	-683.752327	-684.033890	0.320115	-683.408986	0.070773	0.069077	-683.479759	-683.478063	
**TRANS_EneBoryl_c**	-683.752985	-684.034416	0.319764	-683.409771	0.072003	0.069664	-683.481773	-683.479434	
**TRANS_EneBoryl_d**	-683.753711	-684.034476	0.320037	-683.410334	0.071557	0.069342	-683.481890	-683.479676	
**TRANS_FuranBoryl_a**	-795.855550	-796.162320	0.310038	-795.521972	0.072954	0.070189	-795.594926	-795.592161	
**TRANS_FuranBoryl_b**	-795.855543	-796.161916	0.309840	-795.522126	0.073027	0.070284	-795.595153	-795.592411	

**TRANS_FuranBoryl_d**	-795.856403	-796.163426	0.309990	-795.522840	0.073130	0.070304	-795.595970	-795.593144
**TRANS_Me2Boryl_a**	-645.688996	-645.950516	0.314325	-645.352252	0.068912	0.067218	-645.421164	-645.419471
**TRANS_Me2Boryl_b**	-645.690257	-645.951632	0.314199	-645.353595	0.068957	0.067248	-645.422552	-645.420844
**TRANS_MeBoryl_a**	-606.394041	-606.632253	0.286498	-606.086858	0.065526	0.063878	-606.152384	-606.150736
**TRANS_MeBoryl_b**	-606.395286	-606.633548	0.286931	-606.087844	0.064652	0.063369	-606.152496	-606.151213
**TRANS_NapBoryl_a**	-951.601220	-952.030885	0.386072	-951.187469	0.081039	0.078067	-951.268507	-951.265536
**TRANS_NapBoryl_b**	-951.600700	-952.030922	0.385982	-951.186980	0.081232	0.078202	-951.268212	-951.265182
**TRANS_NapBoryl_c**	-951.601475	-952.031561	0.385690	-951.187831	0.083326	0.079196	-951.271157	-951.267028
**TRANS_NapBoryl_d**	-951.600890	-952.031596	0.385624	-951.187341	0.081912	0.078658	-951.269253	-951.265999
**TRANS_PhBoryl_a**	-798.038966	-798.383887	0.339637	-797.674743	0.075437	0.072204	-797.750181	-797.746947
**TRANS_PhBoryl_b**	-798.039547	-798.384657	0.340020	-797.675118	0.074275	0.071725	-797.749393	-797.746843
**TRANS_tBuBoryl_c**	-724.264790	-724.562814	0.369861	-723.869429	0.075286	0.073078	-723.944715	-723.942508

Table S6: Compiled thermochemical data in atomic units for structures

3.6. XYZ coordinates

174
SM-CIS_a
Eopt -5230.801841

Ir	0.345856	-0.297846	1.374885
C	-1.613432	-0.465811	0.589818
P	0.738347	1.816659	0.610619
C	-2.004432	0.268508	1.786508
H	-1.892765	-1.515892	0.559694
H	-1.788849	0.052161	-0.347925
P	1.544953	-1.453414	-0.242812
N	1.797552	2.662375	1.670526
O	1.353956	1.962445	-0.885320
O	-0.488155	2.916542	0.631456
C	-2.302282	-0.347876	2.987197
H	-2.101318	1.343586	1.718059
N	0.757530	-2.111939	-1.595632
O	2.301248	-2.845000	0.168910
O	2.839449	-0.515747	-0.661023
C	1.238491	2.906460	2.986634
C	3.175325	2.223490	1.752693
C	1.714744	3.223619	-1.398006
C	-1.270571	3.259070	-0.479155
C	-2.799133	0.262615	4.202334
H	-2.196042	-1.427792	3.024922
C	1.044832	-3.442791	-2.106697
C	-0.327151	-1.461708	-2.286019
C	3.526831	-2.940329	0.818983
C	3.821389	-0.966737	-1.546303
C	0.916657	4.211936	3.358336
C	1.022590	1.823569	3.869229
C	4.185629	3.117690	1.391531
C	3.489041	0.938732	2.237634
C	0.742540	4.165979	-1.682502
C	3.086457	3.419675	-1.661017
C	-0.705572	3.823993	-1.613490
C	-2.666287	3.093799	-0.328785
C	-3.113981	1.636422	4.321760
C	-3.010136	-0.577885	5.320113
C	2.181434	-3.677133	-2.883732
C	0.151929	-4.496137	-1.813986
C	-0.077133	-0.327488	-3.065026
C	-1.631693	-2.003443	-2.203335
C	4.702559	-2.517875	0.211661
C	3.501248	-3.598379	2.070171
C	4.705039	-1.969882	-1.174567
C	3.881031	-0.310519	-2.795732
C	0.385981	4.464447	4.623602
H	1.086360	5.015823	2.649251
C	0.462902	2.099663	5.131735
C	1.379158	0.436191	3.534811
C	5.523112	2.733434	1.480691
H	3.909806	4.107627	1.045853
C	4.842613	0.560694	2.295560
C	2.468504	0.030097	2.773549
C	1.195941	5.481439	-2.070440
C	3.510906	4.632452	-2.145743
H	3.777988	2.606524	-1.475627
C	-1.575325	4.053983	-2.745988
C	-3.509285	3.414300	-1.362270
H	-3.061532	2.730777	0.611503
C	-3.621185	2.140371	5.513484
H	-2.973586	2.305788	3.479277
C	-3.512403	-0.067174	6.513023
H	-2.779645	-1.636568	5.234579
C	2.497880	-4.969464	-3.300677
H	2.822061	-2.845873	-3.148794
C	0.509735	-5.799016	-2.214786
C	-1.167476	-4.281068	-1.225098
C	-1.116184	0.304777	-3.746463
H	0.936663	0.049741	-3.136839
C	-2.666373	-1.333951	-2.885822
C	-1.958570	-3.204136	-1.434064
C	5.919035	-2.616248	0.987549
C	4.670472	-3.791008	2.761575
H	2.545192	-3.939227	2.449197
C	5.598969	-2.471163	-2.193700
C	4.809842	-0.705155	-3.726732
H	3.194240	0.506072	-2.988186
C	0.161258	3.405694	5.509723
H	0.140814	5.482204	4.912986
H	0.285012	1.278285	5.820289
H	0.909405	-0.324750	4.151264
C	5.849306	1.447955	1.924952
H	6.306123	3.432470	1.201330
H	5.097296	-0.426815	2.665812
H	2.760353	-1.010716	2.867909
C	0.316266	6.594848	-2.180157
C	2.592308	5.701947	-2.319815
H	4.561976	4.796801	-2.367062
C	-1.085006	4.422491	-4.031611
C	-2.990273	3.863597	-2.604248
H	-4.583298	3.299223	-1.245723
C	-3.820758	1.292987	6.611393
H	-3.867500	3.195435	5.590682
H	-3.670808	-0.726591	7.361338
C	1.671015	-6.038795	-2.942061
H	3.395364	-5.139516	-3.888461
H	-0.159381	-6.619947	-1.969773
H	-1.602261	-5.134130	-0.708261
C	-2.417441	-0.198907	-3.650952
H	-0.911893	1.188143	-4.343882
H	-3.677134	-1.726731	-2.811041
H	-2.984662	-3.257835	-1.074818
C	7.149732	-2.033722	0.569060
C	5.898698	-3.284061	2.258501
H	4.664632	-4.312174	3.715105
C	6.389748	-3.642238	-2.016807
C	5.663641	-1.810300	-3.467374
H	4.877427	-0.198040	-4.685366

H	-0.257451	3.597176	6.493406
H	6.888391	1.139313	1.991042
C	0.783841	7.839334	-2.545548
H	-0.735567	6.465965	-1.953308
C	3.038252	6.992284	-2.717414
C	-1.942476	4.624534	-5.093041
H	-0.018120	4.528580	-4.186556
C	-3.849673	4.101157	-3.711544
H	-4.221000	1.693608	7.538639
H	1.921917	-7.051583	-3.245013
H	-3.233342	0.293462	-4.172252
C	8.292366	-2.145716	1.333445
H	7.186349	-1.476299	-0.359016
C	7.098525	-3.400168	3.012910
C	7.224946	-4.098669	-3.014387
H	6.319002	-4.197006	-1.089100
C	6.554817	-2.291252	-4.465895
C	2.154764	8.041484	-2.832873
H	0.091998	8.674863	-2.608459
H	4.098018	7.134963	-2.913848
C	-3.340826	4.479148	-4.933298
H	-1.537129	4.893844	-6.064627
H	-4.918910	3.961332	-3.573677
C	8.276341	-2.849865	2.560655
H	9.213810	-1.683169	0.990028
H	7.060133	-3.923534	3.965051
C	7.326577	-3.409376	-4.246246
H	7.806647	-5.002320	-2.853817
H	6.598019	-1.764391	-5.415933
H	2.506731	9.025653	-3.129376
H	-4.004341	4.650991	-5.776142
H	9.186376	-2.938990	3.147371
H	7.997610	-3.774841	-5.018586
Cl	0.094780	-2.524340	2.480354
C	-7.477526	-1.509612	1.348656
C	-6.506905	-0.388558	1.125431
C	-7.630355	-0.202260	2.095546
H	-7.396463	-0.125555	3.167447
H	-8.468561	0.424808	1.786678
C	-6.008645	-1.699544	1.652446
H	-5.639554	-1.746954	2.687402
H	-5.462399	-2.349040	0.965441
H	-6.257797	0.149863	0.217510
O	-7.892297	-3.085220	-0.699056
O	-9.539701	-1.478418	-0.174737
C	-8.315954	-2.492408	-1.913918
C	-9.703581	-1.830938	-1.536600
C	-8.397230	-3.587766	-2.989281
H	-8.788616	-3.202679	-3.940040
H	-7.394733	-3.991288	-3.182217
H	-9.029575	-4.419797	-2.666182
C	-7.272992	-1.449398	-2.373196
H	-6.285729	-1.928020	-2.392898
H	-7.479515	-1.058805	-3.378284
H	-7.221277	-0.605292	-1.680534
C	-10.049650	-0.563056	-2.332781
H	-10.119012	-0.760485	-3.410493
H	-11.021731	-0.174015	-2.003442
H	-9.308535	0.225464	-2.171747
C	-10.883752	-2.819556	-1.674505
H	-10.668426	-3.772246	-1.182426
H	-11.765008	-2.383658	-1.188049
H	-11.142198	-3.025438	-2.721379
B	-8.540747	-2.395387	0.464434
C	-9.228935	-3.510421	1.466876
H	-9.761514	-3.040191	2.310974
H	-9.958856	-4.160356	0.958155
H	-8.477003	-4.181115	1.916905

174

SM-CIS_b

Eopt -5230.802748

Ir	0.543299	-0.743747	1.262356
C	-1.332536	-1.181904	0.383756
P	0.487335	1.505224	0.888573
C	-1.834328	-0.719417	1.668473
H	-1.388893	-2.250169	0.191569
H	-1.615365	-0.572764	-0.468507
P	1.965114	-1.336770	-0.493997
N	1.360134	2.346105	2.107386
O	1.061965	2.016892	-0.541298
O	-0.931600	2.325424	1.049240
C	-1.942686	-1.522848	2.789849
H	-2.172281	0.304566	1.742830
N	1.329800	-1.902320	-1.964166
O	2.992609	-2.596351	-0.299354
O	3.040600	-0.099700	-0.701884
C	0.787656	2.221406	3.434893
C	2.799841	2.192176	2.139354
C	1.151536	3.390806	-0.838223
C	-1.774723	2.669613	-0.017778
C	-2.527821	-1.171321	4.066947
H	-1.604906	-2.550798	2.700473
C	1.890052	-3.035653	-2.683445
C	0.134812	-1.378144	-2.575421
C	4.208179	-2.557406	0.373482
C	4.100355	-0.182547	-1.608036
C	0.218810	3.343475	4.037592
C	0.819176	0.973902	4.099070
C	3.598074	3.326554	1.976431
C	3.377731	0.931883	2.385947
C	0.003902	4.144377	-1.008443
C	2.452395	3.904931	-1.019505
C	-1.340771	3.504316	-1.037508
C	-3.106532	2.204368	0.066539
C	-3.150589	0.071493	4.325262
C	-2.510005	-2.144564	5.092234
C	3.054195	-2.894410	-3.442083
C	1.234930	-4.283942	-2.611246
C	0.144926	-0.099634	-3.141751
C	-1.029075	-2.180581	-2.633764
C	5.275137	-1.807449	-0.104653
C	4.312018	-3.417385	1.491227

C	5.170586	-1.035130	-1.375543
C	4.031007	0.680209	-2.724559
C	-0.314197	3.247847	5.323303
H	0.203841	4.280879	3.490874
C	0.256360	0.896761	5.387225
C	1.437050	-0.228437	3.515545
C	4.987021	3.216430	2.032030
H	3.118698	4.284844	1.810418
C	4.780464	0.837344	2.414663
C	2.573578	-0.252964	2.713519
C	0.172364	5.569268	-1.176661
C	2.614386	5.239828	-1.298110
H	3.298283	3.233020	-0.937325
C	-2.241134	3.720492	-2.148502
C	-3.998858	2.494507	-0.934379
H	-3.420432	1.636618	0.933416
C	-3.735466	0.321888	5.560743
H	-3.184221	0.834636	3.554504
C	-3.089370	-1.886118	6.330893
H	-2.040797	-3.105674	4.898637
C	3.634834	-4.001780	-4.059024
H	3.510432	-1.917464	-3.535200
C	1.857654	-5.395869	-3.212966
C	-0.101555	-4.450570	-2.046686
C	-1.002133	0.415213	-3.744218
H	1.058749	0.482873	-3.110815
C	-2.179934	-1.628573	-3.230075
C	-1.099078	-3.538542	-2.094394
C	6.482731	-1.795612	0.691355
C	5.492597	-3.490368	2.186138
H	3.443729	-4.003836	1.766961
C	6.154129	-1.159557	-2.427575
C	5.026148	0.650159	-3.670066
H	3.192642	1.363313	-2.802939
C	-0.291011	2.022880	5.997671
H	-0.751710	4.123849	5.793269
H	0.269737	-0.054156	5.912369
H	1.151722	-1.167957	3.979745
C	5.577163	1.966271	2.243832
H	5.604733	4.101293	1.908263
H	5.239054	-0.127066	2.604864
H	3.081104	-1.207694	2.626428
C	-0.921056	6.480036	-1.148560
C	1.492152	6.109812	-1.340562
H	3.608318	5.650338	-1.454136
C	-1.840276	4.374610	-3.348625
C	-3.584673	3.221812	-2.080364
H	-5.024444	2.142291	-0.870165
C	-3.705888	-0.653304	6.566370
H	-4.220146	1.276415	5.744187
H	-3.069268	-2.644374	7.108345
C	3.046841	-5.263168	-3.922170
H	4.551090	-3.879314	-4.629397
H	1.372531	-6.365813	-3.138754
H	-0.349407	-5.452422	-1.701702
C	-2.171148	-0.351565	-3.782382
H	-0.985643	1.411609	-4.175098
H	-3.087639	-2.225539	-3.260219
H	-2.091615	-3.863035	-1.787234
C	7.569926	-0.913075	0.430301
C	6.593586	-2.667250	1.827141
H	5.588772	-4.161238	3.035622
C	7.168862	-2.158815	-2.418897
C	6.087743	-0.287993	-3.567318
H	4.993493	1.320485	-4.524718
H	-0.707819	1.942561	6.997354
H	6.658241	1.871364	2.283963
C	-0.723921	7.835454	-1.306302
H	-1.923426	6.103415	-0.981902
C	1.658573	7.510156	-1.523044
C	-2.722070	4.552718	-4.394375
H	-0.819840	4.723274	-3.452196
C	-4.475756	3.439712	-3.166557
H	-4.168206	-0.452302	7.528908
H	3.504513	-6.134472	-4.382422
H	-3.071560	0.047401	-4.240342
C	8.707248	-0.925939	1.210601
H	7.496106	-0.204511	-0.385653
C	7.787879	-2.671117	2.599083
C	8.085832	-2.256108	-3.443887
H	7.209301	-2.869158	-1.602087
C	7.064180	-0.397916	-4.595378
C	0.574910	8.358789	-1.511007
H	-1.575624	8.509130	-1.268301
H	2.665409	7.897622	-1.657628
C	-4.058157	4.096077	-4.302106
H	-2.383416	5.045630	-5.301737
H	-5.491202	3.060730	-3.084540
C	8.829960	-1.824151	2.296986
H	9.515862	-0.234256	0.990076
H	7.852404	-3.350684	3.445385
C	8.049400	-1.357041	-4.536141
H	8.841049	-3.036897	-3.414414
H	7.003645	0.283256	-5.440436
H	0.713473	9.428154	-1.643862
H	-4.743453	4.251125	-5.130652
H	9.735763	-1.830319	2.896847
H	8.785577	-1.438911	-5.330959
Cl	0.758383	-3.158753	1.908291
C	-6.953543	-1.047544	-0.145708
C	-6.364060	-2.090271	0.757846
C	-5.668978	-1.753534	-0.522805
H	-4.699175	-1.235651	-0.481617
H	-5.745498	-2.469521	-1.343088
C	-6.536648	-0.692938	1.264231
H	-5.638478	-0.088628	1.463098
H	-7.349831	-0.503695	1.967142
H	-6.779909	-3.027150	1.111730
O	-9.552518	-0.738495	-0.104041
O	-8.605026	-2.005183	-1.856311
C	-10.349852	-1.897843	-0.274141
C	-9.957842	-2.404313	-1.721862

C	-11.826192	-1.502595	-0.111230
H	-12.501133	-2.347087	-0.302497
H	-12.006146	-1.160273	0.916064
H	-12.097712	-0.683130	-0.783134
C	-10.006694	-2.927740	0.824553
H	-10.093603	-2.440414	1.803627
H	-10.681841	-3.793444	0.817883
H	-8.980966	-3.291870	0.722648
C	-10.042477	-3.926504	-1.914505
H	-11.060596	-4.304623	-1.754271
H	-9.749693	-4.186390	-2.940003
H	-9.366393	-4.454538	-1.235560
C	-10.802986	-1.733999	-2.828989
H	-10.835938	-0.647537	-2.706421
H	-10.341016	-1.946951	-3.800859
H	-11.835138	-2.106770	-2.856451
B	-8.344744	-0.812241	-0.987346
C	-8.189570	0.591207	-1.843126
H	-7.325714	0.565742	-2.529264
H	-9.072449	0.825067	-2.459509
H	-8.034842	1.461508	-1.181682

174

SM-CIS_c

Eopt -5230.804732

Ir	-0.035068	0.092748	1.337214
C	-1.882095	0.467412	0.350292
P	1.092547	1.895569	0.492332
C	-2.083685	1.399897	1.445057
H	-2.478261	-0.441252	0.364155
H	-1.804460	0.921482	-0.631700
P	0.807795	-1.510852	-0.131735
N	2.323950	2.404187	1.581301
O	1.792600	1.730162	-0.964336
O	0.315013	3.345205	0.384740
C	-2.604040	1.033926	2.673801
H	-1.830989	2.437970	1.280121
N	-0.052908	-1.958189	-1.526079
O	1.026298	-3.043651	0.396647
O	2.367373	-1.078280	-0.467548
C	1.812367	2.939598	2.829075
C	3.441778	1.511623	1.811660
C	2.597331	2.758869	-1.491990
C	-0.234698	3.877039	-0.787562
C	-2.918960	1.894037	3.795582
H	-2.863868	-0.011324	2.807424
C	-0.166911	-3.336596	-1.978165
C	-0.812902	-1.035911	-2.331717
C	2.104526	-3.498502	1.147724
C	3.205319	-1.876901	-1.248012
C	1.974053	4.297185	3.105377
C	1.157368	2.082727	3.742226
C	4.727679	1.953708	1.492908
C	3.238389	0.247159	2.397986
C	2.030167	3.960869	-1.875886
C	3.964955	2.457340	-1.658635
C	0.552029	4.143936	-1.898727
C	-1.604660	4.222007	-0.728759
C	-2.732591	3.296042	3.790005
C	-3.474073	1.291397	4.948179
C	0.890782	-3.956612	-2.647270
C	-1.370577	-4.036470	-1.746193
C	-0.149176	-0.074912	-3.101421
C	-2.225191	-1.133629	-2.363039
C	3.392493	-3.527736	0.626766
C	1.774844	-4.035374	2.413481
C	3.676476	-3.091558	-0.770412
C	3.568682	-1.349378	-2.506941
C	1.498602	4.824015	4.306501
H	2.472490	4.925218	2.373857
C	0.664664	2.641381	4.937213
C	1.000083	0.638221	3.511052
C	5.828037	1.130745	1.730681
H	4.850697	2.941989	1.064245
C	4.358072	-0.577116	2.608539
C	1.922668	-0.194746	2.878614
C	2.932746	5.017521	-2.270016
C	4.810841	3.422648	-2.147281
H	4.319532	1.467277	-1.398037
C	-0.119307	4.596459	-3.096847
C	-2.225019	4.753975	-1.830349
H	-2.150674	4.082382	0.196139
C	-3.096182	4.058370	4.893792
H	-2.307453	3.787031	2.920643
C	-3.831616	2.058523	6.052814
H	-3.628772	0.215803	4.958210
C	0.810101	-5.301689	-3.005368
H	1.779845	-3.384146	-2.876277
C	-1.415918	-5.404771	-2.082325
C	-2.598483	-3.385616	-1.298770
C	-0.870180	0.825557	-3.884564
H	0.934277	-0.039950	-3.082547
C	-2.929004	-0.197051	-3.146584
C	-2.990110	-2.128365	-1.611083
C	4.448757	-3.982801	1.504181
C	2.760360	-4.568628	3.204731
H	0.736508	-4.013819	2.722321
C	4.423775	-3.915748	-1.693675
C	4.379384	-2.078304	-3.341416
H	3.201947	-0.367199	-2.783040
C	0.845153	3.992743	5.222141
H	1.630640	5.880173	4.523011
H	0.156235	1.998086	5.649904
H	0.257035	0.147826	4.133147
C	5.639292	-0.140243	2.282849
H	6.826967	1.479193	1.484357
H	4.214768	-1.553420	3.059793
H	1.823101	-1.257618	3.073465
C	2.501173	6.357745	-2.477919
C	4.331340	4.731527	-2.421229
H	5.864917	3.203936	-2.295010
C	0.538741	4.698538	-4.355949
C	-1.516657	4.918049	-3.049045

H	-3.274666	5.030533	-1.784964
C	-3.645510	3.443943	6.026987
H	-2.953614	5.135012	4.875620
H	-4.260099	1.580485	6.928883
C	-0.339218	-6.036022	-2.696110
H	1.648367	-5.774690	-3.508834
H	-2.330036	-5.957579	-1.881126
H	-3.329756	-4.030639	-0.815739
C	-2.267476	0.766990	-3.900795
H	-0.344740	1.571997	-4.472685
H	-4.014672	-0.241726	-3.152630
H	-4.019031	-1.854109	-1.375189
C	5.829598	-3.869686	1.172764
C	4.118211	-4.533780	2.788238
H	2.514096	-5.002088	4.170309
C	4.762749	-5.270756	-1.415974
C	4.796817	-3.385913	-2.975751
H	4.678977	-1.677353	-4.305956
H	0.469179	4.399443	6.156415
H	6.490741	-0.788672	2.466742
C	3.391677	7.344617	-2.843912
H	1.458226	6.610282	-2.326586
C	5.219337	5.767073	-2.822442
C	-0.134543	5.124365	-5.481981
H	1.582392	4.419568	-4.437111
C	-2.176669	5.375423	-4.222131
H	-3.929358	4.046256	6.885645
H	-0.402076	-7.090158	-2.951997
H	-2.836615	1.472240	-4.499920
C	6.812359	-4.318145	2.030479
H	6.116348	-3.406781	0.236461
C	5.153692	-5.005971	3.640927
C	5.466088	-6.033007	-2.324496
H	4.443465	-5.718197	-0.482566
C	5.545161	-4.189656	-3.879389
C	4.762416	7.048206	-3.033787
H	3.037088	8.362357	-2.982402
H	6.272681	5.526582	-2.943273
C	-1.501848	5.482453	-5.417003
H	0.391848	5.180795	-6.430908
H	-3.232794	5.624646	-4.156273
C	6.475987	-4.909600	3.271074
H	7.856978	-4.210982	1.750620
H	4.875457	-5.433333	4.601152
C	5.880536	-5.486136	-3.562261
H	5.698294	-7.068082	-2.089053
H	5.829314	-3.760968	-4.837311
H	5.450365	7.834584	-3.331502
H	-2.016285	5.825252	-6.310346
H	7.259972	-5.268779	3.931938
H	6.443673	-6.095669	-4.263495
Cl	-1.072310	-1.832027	2.529736
C	-7.090873	-0.104212	0.863902
C	-7.763931	1.159645	0.418808
C	-8.113581	0.574871	1.749978
H	-7.812407	1.113133	2.660666
H	-9.074106	0.065145	1.842188
C	-6.275718	1.137604	0.579970
H	-5.824876	1.724155	1.394107
H	-5.667002	1.112045	-0.325881
H	-8.379257	1.359793	-0.451434
O	-6.381714	-1.778187	-1.027322
O	-8.319122	-2.264123	0.231919
C	-7.390703	-2.207066	-1.931395
C	-8.417485	-2.963151	-0.995533
C	-6.750208	-3.086675	-3.015690
H	-7.505620	-3.521986	-3.682800
H	-6.071822	-2.486067	-3.635010
H	-6.164388	-3.900439	-2.578669
C	-8.024750	-0.979325	-2.619624
H	-7.228170	-0.367421	-3.060666
H	-8.717283	-1.260097	-3.423748
H	-8.566445	-0.358751	-1.901270
C	-9.877901	-2.913491	-1.470719
H	-10.004731	-3.375901	-2.458240
H	-10.513720	-3.461248	-0.763365
H	-10.249207	-1.885599	-1.518566
C	-8.034051	-4.445418	-0.781276
H	-6.979614	-4.553404	-0.511526
H	-8.633708	-4.847563	0.044399
H	-8.225281	-5.061986	-1.669095
B	-6.955136	-1.661321	0.362863
C	-5.996071	-2.464785	1.436243
H	-6.415185	-2.449243	2.456683
H	-5.846769	-3.524863	1.175921
H	-4.989937	-2.018994	1.513537

174
SM-TRANS_a
Eopt -5230.803057

Ir	0.510547	-0.222387	1.498267
C	-1.480653	-0.667800	0.910831
P	0.623875	1.826468	0.497740
C	-1.852259	0.160578	2.048589
H	-1.644496	-1.737062	1.016079
H	-1.780959	-0.278738	-0.056890
P	1.666007	-1.438128	-0.116545
N	1.691379	2.869606	1.351939
O	1.084042	1.857464	-1.059626
O	-0.692060	2.815848	0.532454
C	-1.959850	-0.315039	3.341920
H	-2.078439	1.201607	1.863206
N	0.827377	-2.306864	-1.311016
O	2.590189	-2.704491	0.353192
O	2.819324	-0.440673	-0.752868
C	1.232598	3.202577	2.687387
C	3.105276	2.555726	1.341738
C	1.277540	3.080871	-1.729899
C	-1.602062	2.970531	-0.521621
C	-2.405185	0.408705	4.514223
H	-1.727159	-1.363040	3.505220
C	1.207275	-3.648917	-1.724311
C	-0.377685	-1.838035	-1.946966

C	3.870214	-2.617467	0.889573	C	5.844202	2.028840	1.339546
C	3.761374	-0.895928	-1.678712	H	6.052979	3.956867	0.385771
C	0.836447	4.510447	2.966937	H	5.334317	0.185907	2.325319
C	1.189058	2.199061	3.681080	H	3.090640	-0.568213	2.796675
C	3.994169	3.487948	0.802042	C	-0.495497	6.217137	-2.707434
C	3.575684	1.358108	1.914863	C	1.831765	5.506666	-2.982562
C	0.199480	3.901748	-2.009203	H	3.865477	4.769946	-3.132947
C	2.595589	3.360968	-2.146389	C	-1.851383	3.744342	-4.174398
C	-1.197963	3.450384	-1.759187	C	-3.558121	3.194542	-2.511343
C	-2.957165	2.712877	-0.212860	H	-4.961696	2.663160	-0.947043
C	-2.862721	1.746612	4.483754	C	-3.299398	1.669646	6.863257
C	-2.407929	-0.277826	5.750298	H	-3.664292	3.389563	5.609261
C	2.284176	-3.849591	-2.590460	H	-2.844677	-0.194257	7.854558
C	0.464071	-4.749122	-1.245452	C	2.030036	-6.243294	-2.374488
C	-0.322332	-0.763341	-2.840052	H	3.549220	-5.281029	-3.576262
C	-1.603534	-2.496574	-1.689694	H	0.370952	-6.897870	-1.169377
C	4.941658	-2.160762	0.132668	H	-1.106824	-5.438146	0.082784
C	4.017295	-3.133392	2.197740	C	-2.705579	-0.926789	-3.202935
C	4.769468	-1.769145	-1.295314	H	-1.427789	0.533387	-4.154037
C	3.646490	-0.378561	-2.988116	H	-3.713176	-2.484597	-2.114883
C	0.402101	4.845923	4.249506	H	-2.710299	-3.756039	-0.324910
H	0.873747	5.250600	2.173963	C	7.351883	-1.427851	0.201869
C	0.724484	2.555277	4.961753	C	6.380040	-2.591648	2.120970
C	1.632885	0.816654	3.439704	H	5.382204	-3.556458	3.787127
C	5.364201	3.228094	0.803337	C	6.531711	-3.366803	-2.123652
H	3.599821	4.409556	0.388870	C	5.502069	-1.779614	-3.675459
C	4.958569	1.103490	1.885067	H	4.455757	-0.388747	-4.968328
C	2.691083	0.425742	2.624265	H	0.005225	4.121479	6.244397
C	0.490513	5.199573	-2.573009	H	6.909415	1.817561	1.339804
C	2.858703	4.543881	-2.792391	C	-0.181247	7.447711	-3.243980
H	3.374226	2.633778	-1.949843	H	-1.505782	6.028072	-2.364014
C	-2.185722	3.481758	-2.814953	C	2.117356	6.777217	-3.553529
C	-3.917585	2.847739	-1.183046	C	-2.817706	3.756460	-5.158597
H	-3.229367	2.428797	0.796268	H	-0.817379	3.920043	-4.445717
C	-3.306811	2.364615	5.646560	C	-4.533942	3.237237	-3.544297
H	-2.881301	2.296344	3.548341	H	-3.650782	2.158314	7.767857
C	-2.847126	0.347567	6.913254	H	2.359649	-7.252394	-2.605919
H	-2.066255	-1.309026	5.781316	H	-3.613299	-0.570507	-3.681336
C	2.698348	-5.140643	-2.915847	C	8.562485	-1.356639	0.859057
H	2.802439	-2.991387	-2.998064	H	7.253242	-0.973036	-0.776230
C	0.922241	-6.044893	-1.556926	C	7.646777	-2.520410	2.763326
C	-0.812959	-4.604668	-0.552004	C	7.317195	-3.854515	-3.146440
C	-1.480557	-0.306061	-3.467362	H	6.598767	-3.818662	-1.141449
H	0.633327	-0.292085	-3.040516	C	6.344326	-2.287720	-4.702583
C	-2.761318	-2.003036	-2.322778	C	1.132873	7.729895	-3.687034
C	-1.730870	-3.638994	-0.784911	H	-0.950952	8.210645	-3.322795
C	6.224693	-2.067037	0.793374	H	3.136963	6.984297	-3.868876
C	5.255089	-3.145231	2.789278	C	-4.176156	3.516550	-4.843722
H	3.135354	-3.513030	2.699727	H	-2.530112	3.948027	-6.188864
C	5.615837	-2.298132	-2.340828	H	-5.568714	3.027608	-3.285517
C	4.523929	-0.790008	-3.960800	C	8.721931	-1.922248	2.146211
H	2.867538	0.346713	-3.194857	H	9.401346	-0.855694	0.383350
C	0.348162	3.865798	5.246077	H	7.742336	-2.937662	3.762755
H	0.098857	5.865838	4.467418	C	7.240076	-3.300698	-4.446536
H	0.680096	1.795531	5.737143	H	7.998346	-4.678565	-2.952234
H	1.297815	0.090176	4.174610	H	6.250167	-1.868918	-5.701456

H	1.362791	8.700657	-4.117293
H	-4.927267	3.538675	-5.628332
H	9.683911	-1.867670	2.648116
H	7.873047	-3.688501	-5.239825
Cl	0.574002	-2.344363	2.816066
C	-6.761190	-1.830178	0.970196
C	-6.300892	-0.845442	2.014253
C	-5.756213	-2.235808	2.029787
H	-4.699002	-2.390933	1.770613
H	-6.112212	-2.914112	2.806643
C	-6.059245	-0.549114	0.569456
H	-5.025892	-0.560067	0.193215
H	-6.691075	0.202544	0.095463
H	-6.894005	-0.314471	2.750953
O	-8.216088	-3.384579	-0.562818
O	-9.075183	-1.277930	0.060087
C	-8.723769	-2.768422	-1.730457
C	-9.687950	-1.650670	-1.161644
C	-9.419155	-3.838254	-2.587582
H	-9.897006	-3.406583	-3.476864
H	-8.680558	-4.573629	-2.931853
H	-10.178328	-4.378751	-2.014742
C	-7.555802	-2.182384	-2.556519
H	-6.820584	-2.977764	-2.731694
H	-7.877393	-1.799838	-3.534137
H	-7.050308	-1.373869	-2.020859
C	-9.819862	-0.403067	-2.048683
H	-10.236540	-0.643691	-3.035540
H	-10.493220	0.320984	-1.571939
H	-8.854112	0.090593	-2.192853
C	-11.108315	-2.189146	-0.875710
H	-11.075235	-3.118617	-0.299705
H	-11.652467	-1.445882	-0.280041
H	-11.683570	-2.374430	-1.792171
B	-8.247273	-2.414533	0.585233
C	-8.910188	-3.159748	1.897414
H	-8.990351	-2.500009	2.777739
H	-9.928341	-3.524822	1.689714
H	-8.331038	-4.040222	2.221437

174

SM-TRANS_b

Eopt -5230.801733

Ir	0.569639	-0.214981	1.514606
C	-1.393303	-0.834528	0.987772
P	0.514900	1.782197	0.412285
C	-1.801492	0.022549	2.092574
H	-1.477008	-1.905854	1.151653
H	-1.739417	-0.520643	0.008238
P	1.800520	-1.420711	-0.057488
N	1.503301	2.938646	1.214164
O	0.964015	1.780950	-1.148713
O	-0.876369	2.662188	0.413753
C	-1.852128	-0.401560	3.407944
H	-2.107806	1.034853	1.866299
N	1.026515	-2.395259	-1.212927
O	2.813907	-2.598651	0.456817
O	2.876004	-0.367551	-0.737879
C	1.033882	3.279491	2.544510
C	2.937273	2.733727	1.198669
C	1.057707	2.988536	-1.869000
C	-1.798391	2.693187	-0.642074
C	-2.336098	0.336667	4.554484
H	-1.538033	-1.420513	3.611762
C	1.503359	-3.720339	-1.576956
C	-0.215017	-2.045800	-1.855256
C	4.088225	-2.403645	0.978144
C	3.846311	-0.786354	-1.651007
C	0.543540	4.562876	2.785450
C	1.080141	2.310907	3.572327
C	3.746680	3.712857	0.618438
C	3.504237	1.596204	1.806224
C	-0.082792	3.710666	-2.171884
C	2.347672	3.355597	-2.305083
C	-1.438332	3.157739	-1.898573
C	-3.124433	2.325788	-0.319820
C	-2.895031	1.633499	4.473291
C	-2.274137	-0.294724	5.818280
C	2.586707	-3.874254	-2.444764
C	0.847206	-4.853139	-1.049099
C	-0.245591	-1.016639	-2.801618
C	-1.386308	-2.779490	-1.552365
C	5.120333	-1.895832	0.199039
C	4.278998	-2.865769	2.300757
C	4.916723	-1.568325	-1.241110
C	3.690689	-0.325221	-2.977044
C	0.103872	4.910748	4.062907
H	0.513750	5.274934	1.966823
C	0.610785	2.678044	4.847995
C	1.621744	0.955584	3.371642
C	5.132697	3.559546	0.611857
H	3.278470	4.587225	0.180438
C	4.902309	1.447814	1.767291
C	2.703238	0.622218	2.557825
C	0.102329	5.005512	-2.784324
C	2.514069	4.531399	-2.994941
H	3.181876	2.699746	-2.086763
C	-2.425918	3.064529	-2.950868
C	-4.093787	2.338082	-1.290572
H	-3.369009	2.051250	0.698630
C	-3.374023	2.265076	5.614626
H	-2.963648	2.139515	3.515860
C	-2.748752	0.344690	6.959282
H	-1.854964	-1.295079	5.887726
C	3.092211	-5.142946	-2.725899
H	3.038322	-2.996434	-2.888406
C	1.396457	-6.122828	-1.317601
C	-0.429171	-4.775573	-0.343547
C	-1.439192	-0.677571	-3.437366
H	0.672438	-0.490498	-3.038615
C	-2.582172	-2.404395	-2.195566
C	-1.417898	-3.888346	-0.598410
C	6.395246	-1.685040	0.848831

C	5.516454	-2.766672	2.884476
H	3.429488	-3.292784	2.820202
C	5.798953	-2.068392	-2.271243
C	4.595690	-0.702231	-3.938220
H	2.859004	0.332017	-3.204935
C	0.141185	3.966226	5.093787
H	-0.273028	5.911975	4.250423
H	0.636202	1.945747	5.650240
H	1.362594	0.238465	4.144812
C	5.708873	2.419145	1.180749
H	5.758878	4.324897	0.162394
H	5.351255	0.576098	2.231580
H	3.187680	-0.325759	2.767512
C	-0.962548	5.935324	-2.949038
C	1.413660	5.403106	-3.212249
H	3.498397	4.823751	-3.350480
C	-2.117020	3.304654	-4.320426
C	-3.767574	2.669223	-2.631382
H	-5.116045	2.064927	-1.044689
C	-3.301716	1.625147	6.859132
H	-3.809218	3.257306	5.538994
H	-2.695932	-0.154153	7.922581
C	2.509606	-6.270466	-2.138638
H	3.946850	-5.245423	-3.388430
H	0.912392	-6.998559	-0.892840
H	-0.652539	-5.602114	0.328035
C	-2.613159	-1.371836	-3.127695
H	-1.453889	0.127442	-4.165843
H	-3.493629	-2.946094	-1.957087
H	-2.379561	-4.056352	-0.117048
C	7.469479	-0.983476	0.230020
C	6.593982	-2.151777	2.192111
H	5.677959	-3.135160	3.893968
C	6.792058	-3.058050	-2.021562
C	5.645497	-1.605389	-3.622311
H	4.497048	-0.341350	-4.958413
H	-0.204089	4.231083	6.088922
H	6.787074	2.289615	1.174659
C	-0.748881	7.167653	-3.529192
H	-1.953676	5.677550	-2.594395
C	1.595277	6.671775	-3.828206
C	-3.081636	3.195476	-5.300368
H	-1.102528	3.559222	-4.602556
C	-4.744303	2.587247	-3.661151
H	-3.681197	2.123703	7.746814
H	2.910623	-7.260746	-2.336137
H	-3.548315	-1.107527	-3.612860
C	8.673170	-0.799243	0.877863
H	7.334343	-0.570353	-0.762137
C	7.853666	-1.964060	2.824703
C	7.610312	-3.520728	-3.030241
H	6.893780	-3.470099	-1.024938
C	6.522515	-2.083599	-4.634532
C	0.537645	7.538167	-3.988046
H	-1.577069	7.863800	-3.629977
H	2.594179	6.949318	-4.155571
C	-4.413475	2.848934	-4.971372
H	-2.812844	3.373402	-6.338150
H	-5.756182	2.296632	-3.390415
C	8.878790	-1.307692	2.182196
H	9.470532	-0.252982	0.381143
H	7.982969	-2.339721	3.836737
C	7.491106	-3.018315	-4.347884
H	8.350714	-4.285232	-2.811064
H	6.397129	-1.706253	-5.646392
H	0.688432	8.509049	-4.451764
H	-5.164280	2.775778	-5.753148
H	9.835533	-1.164140	2.676554
H	8.150417	-3.384557	-5.129889
Cl	0.806716	-2.258775	2.949028
C	-7.063470	-1.104669	1.078474
C	-6.129738	-1.412667	2.220824
C	-5.650818	-1.585540	0.816141
H	-4.879781	-0.902199	0.431046
H	-5.559839	-2.603343	0.437047
C	-6.677936	-0.032282	2.079462
H	-5.992629	0.780479	1.797335
H	-7.452106	0.284077	2.779963
H	-6.249340	-2.139011	3.017294
O	-8.410362	-3.122624	0.147041
O	-9.063431	-0.963738	-0.538726
C	-8.667163	-3.169979	-1.246627
C	-9.545038	-1.879508	-1.503454
C	-9.366371	-4.499859	-1.570338
H	-9.650772	-4.567867	-2.628605
H	-8.688982	-5.336219	-1.354367
H	-10.264350	-4.638995	-0.961138
C	-7.333168	-3.128548	-2.025056
H	-6.683476	-3.933050	-1.658637
H	-7.471361	-3.274511	-3.104265
H	-6.813577	-2.178136	-1.870668
C	-9.378157	-1.252611	-2.897110
H	-9.663411	-1.946860	-3.698334
H	-10.020559	-0.366490	-2.981864
H	-8.347383	-0.928915	-3.069465
C	-11.052888	-2.136950	-1.276489
H	-11.236455	-2.656447	-0.331876
H	-11.570892	-1.170953	-1.232946
H	-11.505262	-2.727253	-2.084095
B	-8.528216	-1.713589	0.649462
C	-9.548749	-1.642457	1.944510
H	-9.733712	-0.609454	2.282753
H	-10.537735	-2.072429	1.721970
H	-9.166854	-2.190986	2.822212

174
TS-CIS_a
Eopt -5230.794836

Ir	0.073752	-0.567565	1.125067
C	-1.752786	-0.686044	0.010935
P	0.323126	1.664592	0.909443
C	-2.247440	-0.335317	1.321663
H	-1.918712	-1.705241	-0.325733

H	-1.858737	0.060507	-0.769777
P	1.601752	-1.257204	-0.574538
N	1.183643	2.326694	2.242238
O	1.072113	2.203140	-0.433213
O	-1.002424	2.641465	1.015468
C	-2.760130	-1.290912	2.230505
H	-2.440513	0.706638	1.539919
N	1.063382	-1.632969	-2.148620
O	2.436276	-2.662246	-0.392912
O	2.858273	-0.172470	-0.602306
C	0.464010	2.223340	3.499197
C	2.565116	1.914654	2.405933
C	1.355230	3.572009	-0.589306
C	-1.692031	3.161268	-0.084531
C	-3.318997	-1.017549	3.536409
H	-2.543255	-2.330198	2.022040
C	1.542640	-2.773113	-2.910365
C	0.016781	-0.903679	-2.815156
C	3.562720	-2.826334	0.402181
C	3.984226	-0.352250	-1.403585
C	0.058801	3.388719	4.149576
C	0.199511	0.949078	4.046699
C	3.556301	2.898456	2.421253
C	2.886301	0.558864	2.610772
C	0.329019	4.481132	-0.779803
C	2.719817	3.929428	-0.605300
C	-1.074139	4.022010	-0.980351
C	-3.066562	2.839974	-0.164867
C	-3.758619	0.263258	3.945041
C	-3.449847	-2.095664	4.443052
C	2.785572	-2.736413	-3.546512
C	0.729477	-3.923304	-3.008417
C	0.246847	0.405714	-3.250209
C	-1.227361	-1.532007	-3.059620
C	4.766296	-2.208326	0.085864
C	3.430722	-3.747828	1.466875
C	4.901470	-1.358370	-1.131730
C	4.150549	0.573122	-2.458852
C	-0.602959	3.306561	5.375167
H	0.272013	4.347735	3.687822
C	-0.480971	0.890498	5.276625
C	0.632249	-0.305787	3.399366
C	4.890010	2.543828	2.621851
H	3.267636	3.934231	2.279984
C	4.238199	0.220965	2.796305
C	1.856642	-0.488834	2.723749
C	0.683178	5.881243	-0.792757
C	3.067764	5.251463	-0.735159
H	3.466726	3.149716	-0.513940
C	-1.835454	4.439094	-2.136614
C	-3.822068	3.320610	-1.204008
H	-3.513949	2.226348	0.607700
C	-4.299331	0.451522	5.211295
H	-3.692910	1.104994	3.262968
C	-3.975313	-1.897354	5.718186
H	-3.119027	-3.084389	4.137519
C	3.279389	-3.863531	-4.202492
H	3.368280	-1.825045	-3.514317
C	1.261841	-5.061921	-3.645531
C	-0.668866	-3.945757	-2.586425
C	-0.753770	1.119253	-3.908678
H	1.215982	0.857536	-3.070562
C	-2.225681	-0.784144	-3.714061
C	-1.529202	-2.904304	-2.651041
C	5.872877	-2.409186	0.994877
C	4.509879	-4.023931	2.267687
H	2.465764	-4.216909	1.618327
C	5.965040	-1.558976	-2.089586
C	5.226901	0.458738	-3.303465
H	3.420790	1.367269	-2.572715
C	-0.867438	2.054381	5.938633
H	-0.912856	4.214243	5.885096
H	-0.691117	-0.080156	5.717145
H	0.175636	-1.202654	3.806886
C	5.229198	1.199658	2.804640
H	5.658307	3.311726	2.636336
H	4.501471	-0.818502	2.962309
H	2.230076	-1.506488	2.686476
C	-0.289684	6.919914	-0.767057
C	2.068064	6.259283	-0.788901
H	4.114238	5.542778	-0.762414
C	-1.253532	5.145215	-3.228305
C	-3.225901	4.097888	-2.231234
H	-4.881172	3.085639	-1.263918
C	-4.403578	-0.624291	6.103809
H	-4.641597	1.438418	5.509318
H	-4.058338	-2.734174	6.405689
C	2.524258	-5.040092	-4.229618
H	4.256641	-3.824550	-4.675202
H	0.651491	-5.959838	-3.700135
H	-1.079380	-4.925941	-2.352124
C	-1.996700	0.521050	-4.138680
H	-0.566027	2.137541	-4.235701
H	-3.191099	-1.250519	-3.892297
H	-2.581874	-3.111750	-2.467278
C	7.091333	-1.677115	0.901286
C	5.744737	-3.348608	2.073291
H	4.422997	-4.744404	3.076682
C	6.830020	-2.689958	-2.054642
C	6.137480	-0.622781	-3.165262
H	5.372826	1.173998	-4.108497
H	-1.382332	1.983360	6.892502
H	6.265239	0.913221	2.960180
C	0.083813	8.247116	-0.771626
H	-1.341500	6.662609	-0.722905
C	2.419143	7.636853	-0.816859
C	-2.007051	5.519287	-4.321410
H	-0.195637	5.377836	-3.210043
C	-3.980388	4.517214	-3.360910
H	-4.823871	-0.468656	7.093608
H	2.910869	-5.929071	-4.720355
H	-2.783095	1.072594	-4.646080

C	8.129024	-1.895176	1.783531
H	7.200135	-0.921190	0.133060
C	6.840398	-3.565284	2.953512
C	7.828364	-2.856974	-2.990816
H	6.687737	-3.444227	-1.290250
C	7.193565	-0.809891	-4.099268
C	1.449969	8.614284	-0.812293
H	-0.680026	9.019290	-0.738206
H	3.473823	7.900750	-0.826392
C	-3.388135	5.218530	-4.386662
H	-1.531799	6.047105	-5.143828
H	-5.035243	4.257330	-3.402090
C	8.014284	-2.860633	2.811647
H	9.042926	-1.314645	1.689763
H	6.723049	-4.292659	3.753152
C	8.028745	-1.900518	-4.014566
H	8.464175	-3.737028	-2.944873
H	7.314046	-0.079241	-4.895486
H	1.728631	9.664299	-0.825220
H	-3.970796	5.527868	-5.249862
H	8.843209	-3.029460	3.493424
H	8.826786	-2.039565	-4.738614
Cl	-0.087052	-3.024351	1.630523
C	-6.282956	-2.873280	0.951620
C	-4.991663	-1.941513	1.043088
C	-6.126414	-1.869066	2.035195
H	-5.865502	-2.178564	3.057585
H	-6.802036	-1.014354	1.997748
C	-4.916947	-3.453589	0.984063
H	-4.534519	-3.937673	1.893922
H	-4.561053	-3.922666	0.065522
H	-4.786450	-1.256567	0.232726
O	-6.987731	-3.133183	-1.571055
O	-8.328241	-1.806046	-0.159518
C	-7.397050	-1.993240	-2.313975
C	-8.635359	-1.442700	-1.496561
C	-7.719489	-2.437501	-3.748809
H	-8.107712	-1.609917	-4.356469
H	-6.807517	-2.807330	-4.234848
H	-8.451699	-3.249986	-3.761315
C	-6.238456	-0.975838	-2.374559
H	-5.336664	-1.491586	-2.725405
H	-6.443004	-0.144666	-3.061720
H	-6.022943	-0.556919	-1.387639
C	-8.821941	0.080701	-1.557642
H	-8.989578	0.431120	-2.584128
H	-9.696777	0.369270	-0.961349
H	-7.955832	0.608837	-1.148034
C	-9.965933	-2.103974	-1.918956
H	-9.881813	-3.193476	-1.958933
H	-10.733604	-1.855195	-1.176166
H	-10.315638	-1.751769	-2.897754
B	-7.478659	-3.029608	-0.166972
C	-8.287639	-4.407496	0.263396
H	-8.700119	-4.356154	1.284292
H	-9.132743	-4.618180	-0.409796
H	-7.635518	-5.295885	0.237373

174
TS-CIS_b
Eopt -5230.795347

Ir	-0.144020	-0.405781	0.919538
C	-1.777113	-0.087407	-0.434507
P	0.628422	1.716859	0.853557
C	-2.397375	0.361166	0.790175
H	-2.105755	-1.041722	-0.834367
H	-1.587091	0.666210	-1.191515
P	1.408918	-1.387850	-0.587284
N	1.416780	2.138333	2.323143
O	1.663115	2.097543	-0.345860
O	-0.443045	2.972740	0.831865
C	-3.211865	-0.466021	1.593278
H	-2.380194	1.418094	1.019426
N	1.033388	-1.611284	-2.236149
O	1.889762	-2.944067	-0.362996
O	2.858482	-0.604165	-0.391612
C	0.526776	2.176326	3.469569
C	2.639938	1.428753	2.644808
C	2.262676	3.368315	-0.399193
C	-0.839296	3.654626	-0.323041
C	-3.860774	-0.076405	2.834448
H	-3.173475	-1.529430	1.396911
C	1.377412	-2.808646	-2.983791
C	0.246210	-0.675639	-2.995180
C	2.839879	-3.366978	0.556471
C	4.020185	-1.002351	-1.049121
C	0.292978	3.393592	4.109214
C	-0.077042	0.983079	3.924076
C	3.809464	2.168086	2.835477
C	2.628977	0.031968	2.823979
C	1.502064	4.488188	-0.686582
C	3.661112	3.407924	-0.217214
C	0.074368	4.361462	-1.091728
C	-2.226859	3.666034	-0.594334
C	-4.045115	1.268411	3.224132
C	-4.349811	-1.096276	3.681787
C	2.674675	-3.005976	-3.462440
C	0.376076	-3.772184	-3.233858
C	0.782529	0.569447	-3.339015
C	-1.055485	-1.037507	-3.416550
C	4.179338	-3.020888	0.425338
C	2.372214	-4.260402	1.548169
C	4.656239	-2.191237	-0.718319
C	4.522842	-0.106526	-2.019920
C	-0.535137	3.442310	5.231066
H	0.767703	4.289152	3.720726
C	-0.920352	1.059256	5.047962
C	0.162709	-0.327262	3.289726
C	4.994630	1.522905	3.187370
H	3.774441	3.244594	2.709146
C	3.837416	-0.600987	3.163371
C	1.390215	-0.761983	2.759894
C	2.156656	5.772053	-0.591692

C	4.309024	4.618494	-0.241364
H	4.195853	2.478492	-0.061734
C	-0.405856	4.956427	-2.318793
C	-2.703804	4.322078	-1.700590
H	-2.904103	3.165776	0.086826
C	-4.704000	1.575172	4.411838
H	-3.684071	2.074241	2.593698
C	-4.995096	-0.785468	4.874384
H	-4.218925	-2.134630	3.388144
C	3.019601	-4.194020	-4.106190
H	3.414811	-2.229773	-3.319250
C	0.753906	-4.980088	-3.853353
C	-1.042171	-3.524239	-2.989648
C	0.031094	1.483948	-4.076188
H	1.791937	0.812153	-3.025208
C	-1.799516	-0.089141	-4.145646
C	-1.667430	-2.331247	-3.113122
C	5.085526	-3.481208	1.454198
C	3.250141	-4.785384	2.462305
H	1.317518	-4.509402	1.552993
C	5.773091	-2.593484	-1.542805
C	5.656073	-0.429938	-2.724440
H	4.000827	0.830373	-2.180868
C	-1.137630	2.270996	5.700759
H	-0.712115	4.389555	5.732447
H	-1.394451	0.152788	5.414061
H	-0.535514	-1.106673	3.579189
C	5.006588	0.133599	3.345629
H	5.901977	2.100991	3.337738
H	3.843506	-1.675736	3.312030
H	1.531711	-1.836108	2.706359
C	1.445143	7.003304	-0.653006
C	3.576204	5.827065	-0.385093
H	5.387289	4.665744	-0.115044
C	0.466310	5.519088	-3.294301
C	-1.811061	4.949342	-2.608454
H	-3.770536	4.342984	-1.905742
C	-5.179559	0.552821	5.240193
H	-4.847435	2.614376	4.694032
H	-5.362045	-1.582227	5.515248
C	2.060429	-5.196654	-4.279014
H	4.037912	-4.338943	-4.455524
H	-0.007591	-5.737117	-4.023342
H	-1.663142	-4.407514	-2.853925
C	-1.267480	1.153342	-4.476898
H	0.454678	2.450559	-4.331735
H	-2.808566	-0.347548	-4.456567
H	-2.755163	-2.329935	-3.072896
C	6.433976	-3.032419	1.552486
C	4.616170	-4.394585	2.457901
H	2.903545	-5.487439	3.215983
C	6.363768	-3.886959	-1.462044
C	6.286750	-1.688371	-2.532910
H	6.060760	0.258056	-3.461865
H	-1.784557	2.302104	6.572840
H	5.925007	-0.378175	3.617975
C	2.100799	8.211798	-0.549954
H	0.367138	6.989684	-0.763069
C	4.224653	7.090042	-0.305890
C	-0.024576	6.069973	-4.459740
H	1.536618	5.498926	-3.128094
C	-2.287536	5.544967	-3.808144
H	-5.693900	0.797928	6.165462
H	2.326367	-6.133532	-4.760818
H	-1.860978	1.863909	-5.045194
C	7.273924	-3.495248	2.543936
H	6.803971	-2.300079	0.845314
C	5.512679	-4.868657	3.454732
C	7.422139	-4.242433	-2.271067
H	5.960507	-4.612876	-0.766364
C	7.396221	-2.076248	-3.333751
C	3.506023	8.261072	-0.389732
H	1.530628	9.136076	-0.586599
H	5.301969	7.109626	-0.160928
C	-1.415571	6.099957	-4.717282
H	0.666896	6.481446	-5.190101
H	-3.357933	5.538109	-3.998120
C	6.818973	-4.436848	3.497137
H	8.295300	-3.127589	2.595357
H	5.136169	-5.571326	4.194061
C	7.960037	-3.325164	-3.205039
H	7.843068	-5.241301	-2.194149
H	7.777705	-1.367872	-4.065071
H	4.010084	9.220906	-0.319071
H	-1.788219	6.546022	-5.635106
H	7.494476	-4.801079	4.266214
H	8.801651	-3.615395	-3.827851
Cl	-0.918671	-2.768658	1.281772
C	-6.484078	-0.131755	-0.755810
C	-5.357413	-0.670850	0.253947
C	-5.092252	-0.377988	-1.205834
H	-4.401051	0.457736	-1.386201
H	-4.944356	-1.216779	-1.886797
C	-6.192474	0.563709	0.520749
H	-5.626868	1.506871	0.532069
H	-6.967042	0.516998	1.286095
H	-5.522197	-1.650552	0.680334
O	-8.877209	-1.028747	-0.202459
O	-7.603735	-2.186838	-1.810477
C	-9.109661	-2.416143	-0.004111
C	-8.613861	-3.075968	-1.358008
C	-10.602800	-2.621228	0.298506
H	-10.857382	-3.683880	0.401987
H	-10.858305	-2.124822	1.243371
H	-11.234706	-2.188183	-0.482139
C	-8.307867	-2.898564	1.223299
H	-8.545874	-2.251516	2.076244
H	-8.551121	-3.931212	1.504433
H	-7.230002	-2.839666	1.049548
C	-7.996956	-4.473970	-1.201101
H	-8.715400	-5.194386	-0.789647
H	-7.676343	-4.847544	-2.181877

H	-7.116331	-4.453776	-0.552430
C	-9.730519	-3.162825	-2.422206
H	-10.248245	-2.207462	-2.546179
H	-9.276348	-3.423793	-3.385808
H	-10.479171	-3.928679	-2.182880
B	-7.879236	-0.820874	-1.287244
C	-8.470894	0.224048	-2.426572
H	-7.777361	0.366008	-3.271525
H	-9.422877	-0.128179	-2.852899
H	-8.665575	1.227528	-2.013205

174

TS-CIS_c

Eopt -5230.794803

Ir	0.088845	-0.593738	1.123807
C	-1.736259	-0.754362	0.016670
P	0.285589	1.645547	0.919399
C	-2.236624	-0.413286	1.328334
H	-1.880331	-1.776772	-0.319899
H	-1.863635	-0.010054	-0.763017
P	1.626890	-1.239970	-0.579754
N	1.131586	2.322299	2.254243
O	1.020607	2.208881	-0.420826
O	-1.062893	2.590066	1.031048
C	-2.728542	-1.373641	2.242741
H	-2.453349	0.624722	1.541733
N	1.092691	-1.621210	-2.154098
O	2.492418	-2.626814	-0.405671
O	2.858815	-0.127228	-0.605748
C	0.416825	2.196400	3.511704
C	2.523439	1.945125	2.413496
C	1.269599	3.585025	-0.571266
C	-1.766239	3.096645	-0.066570
C	-3.306634	-1.097597	3.540850
H	-2.483815	-2.409494	2.048129
C	1.595847	-2.747738	-2.920981
C	0.031306	-0.910377	-2.817597
C	3.621944	-2.769403	0.389175
C	3.988390	-0.280014	-1.407777
C	-0.019026	3.348423	4.166086
C	0.186286	0.913860	4.055052
C	3.489085	2.953973	2.431694
C	2.879903	0.597063	2.610997
C	0.220950	4.469228	-0.756795
C	2.624852	3.976477	-0.587779
C	-1.170439	3.976328	-0.958853
C	-3.132349	2.741484	-0.148068
C	-3.780667	0.177376	3.928747
C	-3.416861	-2.166193	4.461446
C	2.838575	-2.682769	-3.555353
C	0.806653	-3.913991	-3.025468
C	0.234223	0.405884	-3.245454
C	-1.199158	-1.563108	-3.067589
C	4.811444	-2.123634	0.075415
C	3.510211	-3.697859	1.450127
C	4.927539	-1.266702	-1.139189
C	4.134578	0.652005	-2.460191
C	-0.678429	3.244477	5.391272
H	0.168388	4.314355	3.707567
C	-0.492811	0.832854	5.284506
C	0.651740	-0.326499	3.403270
C	4.831942	2.632723	2.627197
H	3.173619	3.982708	2.296534
C	4.240605	0.292963	2.791314
C	1.877161	-0.476415	2.723344
C	0.539985	5.877757	-0.763553
C	2.939501	5.307389	-0.712086
H	3.391306	3.215396	-0.501252
C	-1.942295	4.379445	-2.113066
C	-3.899823	3.207024	-1.185306
H	-3.564330	2.113773	0.621906
C	-4.337972	0.368638	5.187213
H	-3.729477	1.011944	3.236706
C	-3.959571	-1.964435	5.728589
H	-3.057304	-3.150033	4.173031
C	3.356455	-3.796511	-4.215581
H	3.402804	-1.760033	-3.518186
C	1.363301	-5.038507	-3.666980
C	-0.591990	-3.967518	-2.607447
C	-0.780057	1.101649	-3.902018
H	1.193214	0.877165	-3.061689
C	-2.212043	-0.833091	-3.720156
C	-1.473436	-2.943696	-2.668867
C	5.922158	-2.303400	0.983770
C	4.595094	-3.952830	2.250222
H	2.555896	-4.188954	1.599398
C	5.994955	-1.441262	-2.097835
C	5.213067	0.563663	-3.305180
H	3.388127	1.430769	-2.571981
C	-0.910120	1.983779	5.950305
H	-1.012411	4.141894	5.904146
H	-0.677798	-0.144588	5.721283
H	0.217267	-1.236563	3.805797
C	5.206099	1.296785	2.802022
H	5.580250	3.420061	2.643822
H	4.531065	-0.740044	2.951866
H	2.274344	-1.484710	2.678466
C	-0.458713	6.891428	-0.731786
C	1.914960	6.290331	-0.759552
H	3.978300	5.624936	-0.739703
C	-1.378615	5.105135	-3.201493
C	-3.323630	4.003501	-2.209221
H	-4.952530	2.945067	-1.246570
C	-4.424814	-0.697989	6.092537
H	-4.707577	1.350438	5.468972
H	-4.027701	-2.793804	6.426701
C	2.625798	-4.988248	-4.249024
H	4.333350	-3.735172	-4.686652
H	0.771368	-5.948336	-3.726749
H	-0.983072	-4.957473	-2.381192
C	-2.009766	0.478769	-4.137749
H	-0.613039	2.125260	-4.223584
H	-3.166298	-1.320080	-3.904425

H -2.522773 -3.173544 -2.493069
C 7.124103 -1.544199 0.892999
C 5.814647 -3.249379 2.058730
H 4.523939 -4.678145 3.056387
C 6.883731 -2.553767 -2.066841
C 6.147066 -0.498094 -3.170433
H 5.343318 1.284450 -4.107965
H -1.423427 1.895839 6.903609
H 6.249567 1.036315 2.953544
C -0.118745 8.227589 -0.730264
H -1.503637 6.607434 -0.687766
C 2.231252 7.676468 -0.781141
C -2.141450 5.464737 -4.292991
H -0.326993 5.364543 -3.182131
C -4.088501 4.408196 -3.337252
H -4.859388 -0.540250 7.075833
H 3.031280 -5.866925 -4.743075
H -2.806364 1.016226 -4.644274
C 8.166148 -1.742197 1.774845
H 7.216123 -0.783360 0.127423
C 6.914575 -3.444520 2.938664
C 7.885269 -2.696230 -3.003661
H 6.757307 -3.313742 -1.305283
C 7.206875 -0.659307 -4.105060
C 1.237700 8.629098 -0.770609
H -0.901694 8.980158 -0.692231
H 3.278793 7.967370 -0.790567
C -3.514365 5.128953 -4.359821
H -1.679807 6.008172 -5.112987
H -5.136221 4.121195 -3.379951
C 8.072494 -2.713371 2.799714
H 9.066945 -1.141163 1.683486
H 6.813225 -4.177015 3.735834
C 8.065179 -1.732113 -4.024048
H 8.539643 -3.562736 -2.960843
H 7.311486 0.076483 -4.898821
H 1.489826 9.685862 -0.778652
H -4.104675 5.426931 -5.221818
H 8.904658 -2.865893 3.481389
H 8.865954 -1.851708 -4.748563
Cl -0.023732 -3.056292 1.614273
C -6.093280 -3.129260 0.764414
C -4.912885 -2.089989 1.021078
C -6.011093 -2.344757 2.023789
H -5.681606 -2.814043 2.962427
H -6.777432 -1.582559 2.165819
C -4.670562 -3.542076 0.663605
H -4.203397 -4.148166 1.452342
H -4.300384 -3.778332 -0.334909
H -4.821921 -1.243597 0.355731
O -6.814347 -2.931777 -1.756051
O -8.282485 -2.116068 -0.103900
C -7.378017 -1.720829 -2.240554
C -8.662144 -1.524299 -1.336906
C -7.665157 -1.883098 -3.741082
H -8.166916 -1.001056 -4.159549
H -6.721627 -2.018058 -4.285529
H -8.286900 -2.761163 -3.937891
C -6.362731 -0.570667 -2.068222
H -5.405653 -0.873150 -2.509868
H -6.687561 0.353137 -2.564444
H -6.189358 -0.347040 -1.012011
C -9.051102 -0.061425 -1.076440
H -9.283234 0.471698 -2.007294
H -9.945401 -0.025214 -0.441425
H -8.255858 0.480039 -0.555682
C -9.900964 -2.254297 -1.902260
H -9.672463 -3.287957 -2.175708
H -10.678815 -2.276576 -1.129429
H -10.315351 -1.750752 -2.784950
B -7.287696 -3.193733 -0.365052
C -7.909405 -4.722213 -0.243406
H -8.294404 -4.943142 0.765597
H -8.741938 -4.891304 -0.943551
H -7.154001 -5.494870 -0.462018
174
TS-CIS_d
Eopt -5230.795347
Ir -0.143911 -0.405611 0.919928
C -1.777098 -0.087273 -0.434080
P 0.628536 1.717000 0.853633
C -2.397330 0.361323 0.790594
H -2.105747 -1.041597 -0.833917
H -1.587073 0.666324 -1.191106
P 1.408725 -1.387826 -0.587185
N 1.417173 2.138591 2.323031
O 1.663023 2.097533 -0.346003
O -0.442932 2.972880 0.832019
C -3.211759 -0.465901 1.593714
H -2.380121 1.418248 1.019864
N 1.032971 -1.611298 -2.235996
O 1.889501 -2.944061 -0.362905
O 2.858349 -0.604206 -0.391719
C 0.527362 2.176680 3.469607
C 2.640348 1.428957 2.644521
C 2.262594 3.368289 -0.399577
C -0.839387 3.654693 -0.322859
C -3.860523 -0.076423 2.834989
H -3.173425 -1.529291 1.397232
C 1.376809 -2.808717 -2.983634
C 0.245839 -0.675587 -2.994994
C 2.839716 -3.367014 0.556442
C 4.019965 -1.002466 -1.049336
C 0.293742 3.393986 4.109241
C -0.076458 0.983486 3.924249
C 3.809942 2.168236 2.834976
C 2.629342 0.032177 2.823734
C 1.501937 4.488129 -0.686964
C 3.661059 3.407909 -0.217825
C 0.074162 4.361374 -1.091828
C -2.227019 3.666242 -0.593818
C -4.044763 1.268349 3.224880

C	-4.349468	-1.096393	3.682266
C	2.674011	-3.006205	-3.462381
C	0.375344	-3.772145	-3.233613
C	0.782269	0.569425	-3.338918
C	-1.055936	-1.037320	-3.416236
C	4.179177	-3.020996	0.425136
C	2.372134	-4.260420	1.548195
C	4.655987	-2.191385	-0.718589
C	4.522582	-0.106667	-2.020178
C	-0.534202	3.442799	5.231216
H	0.768465	4.289499	3.720647
C	-0.919595	1.059761	5.048258
C	0.163113	-0.326896	3.289906
C	4.995132	1.523006	3.186702
H	3.774954	3.244742	2.708611
C	3.837802	-0.600827	3.162956
C	1.390524	-0.761713	2.759868
C	2.156548	5.772004	-0.592323
C	4.308970	4.618474	-0.242211
H	4.195821	2.478492	-0.062326
C	-0.406274	4.956211	-2.318870
C	-2.704142	4.322198	-1.700052
H	-2.904222	3.166302	0.087620
C	-4.703448	1.574972	4.412734
H	-3.683785	2.074247	2.594496
C	-4.994552	-0.785724	4.875005
H	-4.218658	-2.134711	3.388463
C	3.018750	-4.194295	-4.106146
H	3.414253	-2.230091	-3.319257
C	0.752986	-4.980099	-3.853128
C	-1.042855	-3.524044	-2.989284
C	0.030873	1.483990	-4.076051
H	1.791733	0.812025	-3.025207
C	-1.799923	-0.088893	-4.145295
C	-1.667995	-2.330985	-3.112716
C	5.085471	-3.481362	1.453883
C	3.250151	-4.785452	2.462215
H	1.317427	-4.509366	1.553153
C	5.772729	-2.593699	-1.543195
C	5.655723	-0.430142	-2.724811
H	4.000610	0.830268	-2.181060
C	-1.136702	2.271542	5.701039
H	-0.711044	4.390074	5.732588
H	-1.393694	0.153338	5.414468
H	-0.535042	-1.106275	3.579621
C	5.007042	0.133705	3.345004
H	5.902532	2.101050	3.336905
H	3.843862	-1.675573	3.311644
H	1.531979	-1.835851	2.706479
C	1.445031	7.003252	-0.653653
C	3.576130	5.827031	-0.385957
H	5.387255	4.665736	-0.116066
C	0.465733	5.518678	-3.294632
C	-1.811541	4.949216	-2.608226
H	-3.770924	4.343230	-1.904934
C	-5.178908	0.552524	5.241024
H	-4.846801	2.614142	4.695093
H	-5.361426	-1.582556	5.515820
C	2.059449	-5.196819	-4.278890
H	4.037017	-4.339338	-4.455558
H	-0.008614	-5.737039	-4.023052
H	-1.663910	-4.407248	-2.853491
C	-1.267774	1.153519	-4.476636
H	0.454539	2.450551	-4.331657
H	-2.809032	-0.347194	-4.456114
H	-2.755725	-2.329552	-3.072386
C	6.433956	-3.032642	1.552003
C	4.616198	-4.394720	2.457641
H	2.903616	-5.487499	3.215930
C	6.363332	-3.887211	-1.462500
C	6.286341	-1.688615	-2.533350
H	6.060383	0.257833	-3.462269
H	-1.783498	2.302722	6.573215
H	5.925477	-0.378109	3.617220
C	2.100710	8.211753	-0.550834
H	0.367008	6.989626	-0.763540
C	4.224597	7.090014	-0.306991
C	-0.025355	6.069456	-4.460037
H	1.536075	5.498452	-3.128642
C	-2.288222	5.544724	-3.807891
H	-5.693088	0.797522	6.166411
H	2.325241	-6.133731	-4.760710
H	-1.861244	1.864138	-5.044897
C	7.274009	-3.495521	2.543342
H	6.803900	-2.300314	0.844790
C	5.512811	-4.868847	3.454353
C	7.421594	-4.242750	-2.271637
H	5.960098	-4.613105	-0.766781
C	7.395703	-2.076559	-3.334308
C	3.505959	8.261039	-0.390841
H	1.530536	9.136030	-0.587484
H	5.301936	7.109608	-0.162202
C	-1.416402	6.099520	-4.717287
H	0.665995	6.480784	-5.190595
H	-3.358660	5.537937	-3.997637
C	6.819133	-4.437107	3.496592
H	8.295410	-3.127915	2.594634
H	5.136358	-5.571503	4.193724
C	7.959454	-3.325511	-3.205661
H	7.842466	-5.241646	-2.194769
H	7.777154	-1.368205	-4.065667
H	4.010036	9.220878	-0.320362
H	-1.789208	6.545500	-5.635089
H	7.494718	-4.801382	4.265577
H	8.800983	-3.615794	-3.828564
Cl	-0.918527	-2.768451	1.282547
C	-6.484408	-0.131277	-0.754934
C	-5.357410	-0.670631	0.254311
C	-5.092478	-0.376432	-1.205239
H	-4.401701	0.459779	-1.384978
H	-4.944195	-1.214594	-1.886894
C	-6.193101	0.563269	0.522189

H	-5.627994	1.506724	0.534320
H	-6.967617	0.515500	1.287524
H	-5.521659	-1.650781	0.679879
O	-8.877320	-1.029382	-0.202379
O	-7.603149	-2.186346	-1.810658
C	-9.109104	-2.416957	-0.004507
C	-8.612979	-3.076040	-1.358632
C	-10.602143	-2.622870	0.298032
H	-10.856231	-3.685688	0.401013
H	-10.857871	-2.127029	1.243135
H	-11.234255	-2.189746	-0.482401
C	-8.307080	-2.899441	1.222730
H	-8.545302	-2.252739	2.075876
H	-8.549944	-3.932264	1.503564
H	-7.229243	-2.840096	1.048960
C	-7.995580	-4.473884	-1.202272
H	-8.713801	-5.194735	-0.791190
H	-7.674747	-4.846922	-2.183180
H	-7.115019	-4.453653	-0.553515
C	-9.729550	-3.162841	-2.422924
H	-10.247608	-2.207606	-2.546506
H	-9.275242	-3.423237	-3.386616
H	-10.477942	-3.929063	-2.183963
B	-7.879227	-0.820695	-1.286900
C	-8.471068	0.224486	-2.425898
H	-7.777418	0.367074	-3.270650
H	-9.422826	-0.127925	-2.852573
H	-8.666220	1.227715	-2.012146

174

TS-TRANS_a

Eopt -5230.794503

Ir	0.274973	-0.796258	1.100516
C	-1.574031	-1.050297	0.060252
P	0.177340	1.458080	1.065250
C	-2.014278	-0.928550	1.433757
H	-1.637474	-2.039986	-0.382075
H	-1.827486	-0.248567	-0.626225
P	1.825822	-1.115664	-0.683459
N	0.996979	2.138914	2.412602
O	0.751898	2.214212	-0.258437
O	-1.274556	2.201337	1.314448
C	-2.436393	-2.061485	2.171590
H	-2.300899	0.042502	1.815394
N	1.300613	-1.445229	-2.271356
O	2.865391	-2.388719	-0.635024
O	2.904462	0.145593	-0.647310
C	0.364930	1.830427	3.683270
C	2.430444	1.928540	2.488243
C	0.778624	3.619857	-0.318488
C	-2.112266	2.642641	0.283109
C	-2.907816	-2.093332	3.539705
H	-2.218117	-3.029891	1.735854
C	1.926864	-2.429531	-3.136241
C	0.115157	-0.861079	-2.840773
C	4.036484	-2.442304	0.107773
C	4.012749	0.206810	-1.489051
C	-0.168444	2.868433	4.446992
C	0.315205	0.491849	4.129331
C	3.265389	3.047006	2.538109
C	2.957726	0.625291	2.577033
C	-0.400275	4.340823	-0.406670
C	2.056448	4.216826	-0.350887
C	-1.709772	3.650722	-0.580273
C	-3.403492	2.070106	0.236171
C	-3.341846	-0.946600	4.243072
C	-2.958008	-3.347431	4.192327
C	3.112014	-2.135190	-3.813995
C	1.314858	-3.692176	-3.293080
C	0.104250	0.493634	-3.188188
C	-1.017935	-1.673504	-3.081351
C	5.121613	-1.627185	-0.190402
C	4.084736	-3.454261	1.094596
C	5.078957	-0.668703	-1.332420
C	3.997715	1.233327	-2.460428
C	-0.745239	2.593390	5.687332
H	-0.119824	3.881768	4.060870
C	-0.282568	0.237098	5.377123
C	0.893081	-0.630568	3.361400
C	4.645045	2.884819	2.661379
H	2.820668	4.034789	2.485238
C	4.351784	0.484095	2.689541
C	2.102695	-0.573010	2.639430
C	-0.299063	5.779609	-0.331093
C	2.159536	5.585415	-0.393791
H	2.932740	3.579354	-0.341761
C	-2.597466	3.986173	-1.670910
C	-4.286513	2.448785	-0.742744
H	-3.685515	1.335468	0.979987
C	-3.817967	-1.059414	5.544411
H	-3.328449	0.026045	3.762742
C	-3.417649	-3.451501	5.501400
H	-2.626884	-4.233451	3.657654
C	3.753725	-3.111457	-4.575598
H	3.534253	-1.142135	-3.731857
C	1.997322	-4.674509	-4.037871
C	-0.036167	-3.982899	-2.820364
C	-1.033282	1.069614	-3.752223
H	0.995218	1.087464	-3.014878
C	-2.159326	-1.063371	-3.637563
C	-1.061769	-3.102468	-2.769979
C	6.282391	-1.732444	0.666135
C	5.224672	-3.628892	1.837353
H	3.204943	-4.071716	1.233958
C	6.120929	-0.624258	-2.332965
C	5.045031	1.355662	-3.339771
H	3.153116	1.913643	-2.482140
C	-0.795760	1.275642	6.152238
H	-1.155001	3.402467	6.285139
H	-0.326707	-0.785390	5.741462
H	0.594261	-1.614679	3.709537
C	5.186855	1.597911	2.733894
H	5.291439	3.756946	2.702404

H	4.773088	-0.512539	2.768596
H	2.621401	-1.516332	2.507048
C	-1.437943	6.623478	-0.201028
C	0.996118	6.398897	-0.344170
H	3.135816	6.060851	-0.432002
C	-2.218271	4.857803	-2.731717
C	-3.901321	3.388960	-1.734335
H	-5.273619	1.996558	-0.788078
C	-3.855080	-2.308270	6.178021
H	-4.162330	-0.173641	6.070241
H	-3.444504	-4.420464	5.991364
C	3.206388	-4.394918	-4.666744
H	4.684975	-2.872122	-5.081098
H	1.544245	-5.657427	-4.140222
H	-0.263798	-5.033245	-2.649242
C	-2.170879	0.286742	-3.974112
H	-1.034172	2.123954	-4.011757
H	-3.042195	-1.672762	-3.812717
H	-2.053709	-3.500968	-2.565560
C	7.375095	-0.820749	0.601235
C	6.337926	-2.763202	1.664509
H	5.277880	-4.415862	2.585028
C	7.144061	-1.610852	-2.423551
C	6.110478	0.416140	-3.323410
H	5.051799	2.151214	-4.080001
H	-1.242169	1.054941	7.117744
H	6.259845	1.460506	2.830786
C	-1.305469	7.993657	-0.123564
H	-2.424827	6.179687	-0.142788
C	1.096809	7.816010	-0.284763
C	-3.085089	5.146577	-3.765106
H	-1.224753	5.289693	-2.737865
C	-4.778848	3.723760	-2.801735
H	-4.227812	-2.388502	7.195472
H	3.710910	-5.167147	-5.240937
H	-3.063233	0.730254	-4.406353
C	8.468194	-0.950008	1.432658
H	7.340142	0.002130	-0.102263
C	7.488206	-2.882644	2.492018
C	8.119652	-1.543323	-3.395488
H	7.144225	-2.441354	-1.727915
C	7.145784	0.471978	-4.296845
C	-0.028706	8.601550	-0.180490
H	-2.191626	8.612559	-0.012196
H	2.086662	8.265033	-0.307749
C	-4.384954	4.588554	-3.797687
H	-2.762827	5.807226	-4.565504
H	-5.766332	3.269521	-2.818842
C	8.538745	-2.000583	2.377872
H	9.281780	-0.232841	1.363006
H	7.511210	-3.680362	3.230455
C	8.136558	-0.482690	-4.332355
H	8.880242	-2.317860	-3.445842
H	7.125880	1.278405	-5.025876
H	0.059378	9.683127	-0.127379
H	-5.060238	4.832888	-4.612907

H	9.410232	-2.096411	3.019548
H	8.918288	-0.436216	-5.085511
Cl	0.462730	-3.281054	1.412144
C	-6.087192	-2.785280	0.423211
C	-4.786927	-2.180864	1.131182
C	-5.649231	-3.238690	1.775453
H	-5.166038	-4.217309	1.897397
H	-6.272562	-2.959967	2.624134
C	-4.755420	-2.806970	-0.245229
H	-4.187599	-3.743841	-0.316638
H	-4.641433	-2.151601	-1.106445
H	-4.832005	-1.120840	1.338869
O	-8.433013	-2.756769	-0.683931
O	-7.000076	-0.922144	-1.062364
C	-8.409279	-2.511251	-2.081098
C	-7.879223	-1.025196	-2.174356
C	-9.825549	-2.723123	-2.636698
H	-9.887152	-2.471337	-3.703313
H	-10.112259	-3.776692	-2.526822
H	-10.562399	-2.122761	-2.095302
C	-7.465570	-3.522556	-2.767083
H	-7.767641	-4.536063	-2.476305
H	-7.504715	-3.459581	-3.862101
H	-6.428399	-3.380168	-2.450962
C	-7.099921	-0.702171	-3.456879
H	-7.722713	-0.823438	-4.352491
H	-6.755806	0.339737	-3.430893
H	-6.216823	-1.339053	-3.563031
C	-9.012340	0.012907	-2.020835
H	-9.643024	-0.208396	-1.154652
H	-8.565238	1.002558	-1.865537
H	-9.655588	0.068764	-2.908203
B	-7.415581	-1.895888	-0.004078
C	-8.082569	-1.191360	1.321727
H	-7.385475	-0.537938	1.871752
H	-8.944886	-0.563542	1.050016
H	-8.458050	-1.928916	2.049421

174
TS-TRANS_b
Eopt -5230.794832

Ir	0.118360	-0.477239	1.170742
C	-1.731391	-0.589402	0.092957
P	0.477842	1.720767	0.786033
C	-2.208835	-0.131101	1.377796
H	-1.942734	-1.620646	-0.172403
H	-1.807075	0.104626	-0.737422
P	1.585435	-1.353169	-0.481697
N	1.391723	2.434626	2.056325
O	1.229615	2.128020	-0.601027
O	-0.795393	2.768817	0.841695
C	-2.748048	-1.001341	2.350314
H	-2.348388	0.931505	1.528924
N	1.017994	-1.806973	-2.024379
O	2.359503	-2.778297	-0.211777
O	2.887132	-0.329370	-0.586499
C	0.685353	2.472635	3.323928

C	2.752323	1.966515	2.239385	C	5.907997	-1.937070	-2.023986
C	1.575918	3.467597	-0.855288	C	5.236420	0.031835	-3.352244
C	-1.477849	3.245137	-0.281977	H	3.477633	1.057060	-2.657660
C	-3.266081	-0.617354	3.650254	C	-0.626002	2.572534	5.778352
H	-2.577950	-2.060531	2.206829	H	-0.562114	4.720173	5.547335
C	1.454247	-3.003289	-2.723703	H	-0.561979	0.421078	5.732264
C	-0.023440	-1.094485	-2.717037	H	0.224367	-0.897884	3.907069
C	3.491861	-2.939792	0.575562	C	5.382840	1.146394	2.662276
C	3.991722	-0.605659	-1.389212	H	5.916532	3.213042	2.320436
C	0.346749	3.706614	3.878890	H	4.554458	-0.812116	2.990024
C	0.363442	1.262957	3.977085	H	2.241776	-1.398725	2.793348
C	3.792985	2.894984	2.164328	C	0.091253	6.871909	-1.247717
C	3.007453	0.616856	2.550101	C	2.414080	6.096953	-1.250424
C	0.592020	4.410773	-1.096598	H	4.423649	5.284309	-1.198059
C	2.955616	3.756137	-0.912653	C	-1.000176	4.981027	-3.563723
C	-0.834756	4.009015	-1.245234	C	-3.003154	4.107300	-2.465731
C	-2.867793	2.990261	-0.318707	H	-4.688591	3.251433	-1.403692
C	-3.599945	0.712746	3.989389	C	-4.313517	-0.005017	6.188502
C	-3.465921	-1.630665	4.615750	H	-4.386909	2.035517	5.488486
C	2.682885	-3.036578	-3.387296	H	-4.120544	-2.116245	6.604973
C	0.610830	-4.135690	-2.737399	C	2.348692	-5.368562	-3.928597
C	0.232237	0.175999	-3.242934	H	4.099655	-4.226367	-4.482165
C	-1.289437	-1.702244	-2.891625	H	0.465832	-6.206178	-3.306768
C	4.716087	-2.397474	0.203696	H	-1.206998	-5.051081	-1.987123
C	3.337473	-3.785974	1.698217	C	-2.031628	0.300312	-4.078083
C	4.869202	-1.631861	-1.066682	H	-0.558782	1.862638	-4.320706
C	4.178598	0.243944	-2.503019	H	-3.269037	-1.415102	-3.686000
C	-0.304204	3.759060	5.111833	H	-2.666872	-3.209734	-2.177623
H	0.602139	4.612434	3.337930	C	7.074992	-1.920309	0.952790
C	-0.306900	1.339997	5.211841	C	5.676337	-3.453724	2.246002
C	0.727409	-0.059998	3.433925	H	4.312310	-4.724315	3.353356
C	5.109727	2.487772	2.376817	C	6.724693	-3.100131	-1.930174
H	3.556042	3.930117	1.944425	C	6.102913	-1.077066	-3.157961
C	4.342947	0.223630	2.745103	H	5.399659	0.687924	-4.203069
C	1.927324	-0.361035	2.762357	H	-1.133562	2.606706	6.738136
C	1.012761	5.787671	-1.211744	H	6.405334	0.819418	2.826992
C	3.364814	5.047393	-1.138398	C	0.527756	8.175814	-1.349349
H	3.666224	2.949741	-0.776064	H	-0.970911	6.669886	-1.173990
C	-1.595793	4.382680	-2.416494	C	2.830272	7.450597	-1.378150
C	-3.618103	3.435128	-1.377305	C	-1.754698	5.316371	-4.668706
H	-3.329757	2.455362	0.501910	H	0.068013	5.160885	-3.578113
C	-4.124825	1.010065	5.244117	C	-3.757031	4.485126	-3.610303
H	-3.458999	1.510713	3.268063	H	-4.723902	0.232515	7.166169
C	-3.976291	-1.326571	5.873114	H	2.701576	-6.294191	-4.374936
H	-3.218222	-2.657890	4.361438	H	-2.816126	0.841993	-4.598853
C	3.133163	-4.212564	-3.986521	C	8.116524	-2.128782	1.832849
H	3.288277	-2.140448	-3.423228	H	7.203881	-1.219009	0.137356
C	1.099919	-5.323184	-3.317335	C	6.775867	-3.663921	3.122948
C	-0.777766	-4.097073	-2.286964	C	7.700592	-3.367053	-2.866853
C	-0.765819	0.874309	-3.921491	H	6.562717	-3.799330	-1.118740
H	1.218706	0.608606	-3.115921	C	7.135279	-1.366807	-4.092317
C	-2.285645	-0.967863	-3.564600	C	1.909206	8.472147	-1.430853
C	-1.613907	-3.039359	-2.394370	H	-0.197599	8.984836	-1.361478
C	5.827555	-2.590294	1.108691	H	3.896097	7.661655	-1.417604
C	4.416558	-4.059713	2.499821	C	-3.149969	5.082504	-4.691700
H	2.355592	-4.201467	1.891829	H	-1.269477	5.761653	-5.533082

H	-4.824050	4.276947	-3.617002
C	7.976877	-3.022417	2.921005
H	9.053158	-1.596465	1.689968
H	6.640226	-4.334210	3.968213
C	7.925086	-2.485007	-3.950645
H	8.299322	-4.269172	-2.774531
H	7.274075	-0.692199	-4.933605
H	2.237841	9.503872	-1.519889
H	-3.732642	5.360779	-5.565398
H	8.808797	-3.185321	3.600562
H	8.705413	-2.702420	-4.674666
Cl	-0.150980	-2.875077	1.873874
C	-6.373145	-1.269207	0.510923
C	-5.039973	-1.600278	1.353735
C	-5.035905	-1.342660	-0.134284
H	-4.508011	-0.427012	-0.437405
H	-4.874304	-2.179017	-0.813379
C	-5.986049	-0.479739	1.711623
H	-5.567543	0.530351	1.595926
H	-6.609340	-0.580016	2.600069
H	-5.005107	-2.571502	1.829777
O	-7.503934	-3.154910	-0.873979
O	-8.810905	-1.326189	-0.160347
C	-8.236983	-2.684044	-1.996105
C	-9.407239	-1.850848	-1.336436
C	-8.694777	-3.895731	-2.821020
H	-9.331412	-3.599221	-3.664578
H	-7.819832	-4.415697	-3.231644
H	-9.245752	-4.613522	-2.206408
C	-7.328267	-1.808867	-2.886792
H	-6.432157	-2.382990	-3.152817
H	-7.822341	-1.508391	-3.819469
H	-7.006086	-0.903771	-2.363323
C	-9.925632	-0.682035	-2.187016
H	-10.333116	-1.023743	-3.147251
H	-10.730264	-0.162816	-1.650870
H	-9.136692	0.049362	-2.385720
C	-10.610694	-2.737165	-0.945822
H	-10.291218	-3.626219	-0.394202
H	-11.276456	-2.159062	-0.293577
H	-11.191700	-3.065061	-1.817370
B	-7.698495	-2.230874	0.279216
C	-8.046632	-3.075555	1.644973
H	-8.175103	-2.434962	2.532612
H	-8.981610	-3.647422	1.541017
H	-7.263780	-3.808470	1.900078

174

TS-TRANS_c

Eopt -5230.795976

Ir	0.117527	-0.690575	0.977808
C	-1.635868	-0.785150	-0.260017
P	0.350537	1.553588	0.940916
C	-2.259134	-0.510614	1.012648
H	-1.729075	-1.790065	-0.658707
H	-1.693932	-0.004337	-1.011087
P	1.750049	-1.235638	-0.658569
N	1.133950	2.127565	2.361441
O	1.161329	2.197825	-0.317352
O	-0.986287	2.515901	1.055137
C	-2.808079	-1.503771	1.853717
H	-2.475563	0.520165	1.258447
N	1.333478	-1.487674	-2.294254
O	2.586745	-2.645713	-0.530686
O	2.991114	-0.143262	-0.510133
C	0.348449	1.946935	3.568592
C	2.508171	1.717451	2.576385
C	1.437482	3.575524	-0.361283
C	-1.627665	3.114317	-0.033107
C	-3.417712	-1.269170	3.156412
H	-2.546289	-2.531381	1.637895
C	1.892719	-2.551028	-3.111088
C	0.314173	-0.728927	-2.970813
C	3.662197	-2.866837	0.319093
C	4.171670	-0.245451	-1.242379
C	-0.108706	3.069226	4.259263
C	0.068513	0.641159	4.027610
C	3.487467	2.704913	2.705816
C	2.831837	0.354417	2.717324
C	0.413020	4.488512	-0.541016
C	2.797419	3.942998	-0.283902
C	-0.974547	4.035776	-0.839281
C	-2.998016	2.804962	-0.192516
C	-3.909202	-0.012300	3.569976
C	-3.544684	-2.364977	4.038541
C	3.172999	-2.436349	-3.657779
C	1.118418	-3.705905	-3.357429
C	0.538861	0.614599	-3.288193
C	-0.897197	-1.359802	-3.341955
C	4.876210	-2.216046	0.135284
C	3.473618	-3.877155	1.290690
C	5.081172	-1.264656	-0.994518
C	4.398178	0.769384	-2.199648
C	-0.839771	2.910884	5.436943
H	0.117977	4.055177	3.865785
C	-0.683525	0.505846	5.209202
C	0.554819	-0.569081	3.339180
C	4.811429	2.347050	2.958507
H	3.197303	3.745679	2.612646
C	4.174273	0.013634	2.957257
C	1.806166	-0.702226	2.712188
C	0.752906	5.888806	-0.441618
C	3.137981	5.273090	-0.306893
H	3.546935	3.164196	-0.208167
C	-1.685104	4.528593	-1.998095
C	-3.708778	3.353710	-1.229448
H	-3.477652	2.146019	0.520984
C	-4.506569	0.136494	4.818074
H	-3.845184	0.844156	2.906323
C	-4.130160	-2.209183	5.292084
H	-3.180177	-3.339805	3.725039
C	3.737181	-3.494296	-4.369997
H	3.729258	-1.519601	-3.512632

C	1.720252	-4.776329	-4.048577	C	6.776666	-3.798792	3.008278
C	-0.303382	-3.792623	-3.035584	C	8.144683	-2.578338	-2.771540
C	-0.439636	1.359949	-3.945078	H	6.897283	-3.318973	-1.211520
H	1.486022	1.068503	-3.018510	C	7.562674	-0.446059	-3.739427
C	-1.876051	-0.579894	-3.988690	C	1.491652	8.622306	-0.240570
C	-1.185653	-2.768101	-3.071195	H	-0.643454	9.006535	-0.244828
C	5.923883	-2.486197	1.095194	H	3.520967	7.925573	-0.201737
C	4.501774	-4.213241	2.134301	C	-3.137370	5.454645	-4.259543
H	2.506832	-4.364037	1.338360	H	-1.251974	6.332982	-4.875337
C	6.209282	-1.374762	-1.891398	H	-4.823850	4.429772	-3.416113
C	5.531328	0.736513	-2.974202	C	7.948384	-3.076487	3.004469
H	3.667996	1.565733	-2.293516	H	9.028063	-1.432949	2.087798
C	-1.122490	1.626103	5.911734	H	6.616757	-4.592682	3.733793
H	-1.190416	3.785197	5.977849	C	8.402963	-1.535057	-3.692342
H	-0.908604	-0.490396	5.579336	H	8.786100	-3.455436	-2.758456
H	0.074175	-1.492295	3.648224	H	7.728005	0.351985	-4.459226
C	5.153538	0.996532	3.079120	H	1.759859	9.672749	-0.169577
H	5.570212	3.117579	3.061853	H	-3.681629	5.820098	-5.125863
H	4.437586	-1.032369	3.075152	H	8.733580	-3.297253	3.722197
H	2.183965	-1.714391	2.615693	H	9.249948	-1.605741	-4.369152
C	-0.230635	6.916601	-0.394523	Cl	-0.050531	-3.173496	1.337201
C	2.131964	6.275382	-0.343701	C	-5.956602	-2.235862	-0.535449
H	4.181484	5.572808	-0.261832	C	-4.953290	-2.123232	0.723403
C	-1.057140	5.306246	-3.012927	C	-4.666495	-2.974060	-0.496637
C	-3.068757	4.194132	-2.177608	H	-3.822020	-2.622946	-1.101656
H	-4.764692	3.127226	-1.348782	H	-4.708887	-4.058965	-0.403991
C	-4.617290	-0.957952	5.683800	C	-5.466806	-0.922586	-0.042207
H	-4.890888	1.107337	5.117915	H	-4.687169	-0.378160	-0.593961
H	-4.215833	-3.062274	5.959233	H	-6.208993	-0.279395	0.427945
C	3.016639	-4.679888	-4.543722	H	-5.353146	-2.488215	1.658995
H	4.741591	-3.395673	-4.771787	O	-8.081655	-2.850079	-1.869134
H	1.137699	-5.678468	-4.217761	O	-8.352253	-1.854026	0.250449
H	-0.702458	-4.797734	-2.914273	C	-8.999851	-1.777138	-2.012353
C	-1.654466	0.760093	-4.291661	C	-9.492757	-1.528250	-0.531539
H	-0.257152	2.404340	-4.179616	C	-10.100493	-2.208211	-2.992941
H	-2.818931	-1.048545	-4.258950	H	-10.883019	-1.444532	-3.089449
H	-2.241985	-3.013784	-2.979630	H	-9.667133	-2.368043	-3.988431
C	7.136599	-1.740383	1.144901	H	-10.568647	-3.146751	-2.682574
C	5.737938	-3.515065	2.079442	C	-8.273311	-0.552658	-2.609474
H	4.371906	-5.001319	2.871378	H	-7.769418	-0.860050	-3.533947
C	7.082808	-2.499729	-1.895603	H	-8.960975	0.266417	-2.856018
C	6.443404	-0.347365	-2.867593	H	-7.511023	-0.167292	-1.926224
H	5.723322	1.520258	-3.702071	C	-9.906341	-0.081747	-0.225255
H	-1.692232	1.496079	6.827412	H	-10.752337	0.239743	-0.846313
H	6.182205	0.708246	3.274807	H	-10.215009	0.000677	0.824705
C	0.128999	8.243711	-0.292048	H	-9.078826	0.616467	-0.382184
H	-1.280645	6.649670	-0.419934	C	-10.659292	-2.459846	-0.135715
C	2.469887	7.654143	-0.262468	H	-10.443836	-3.501493	-0.392020
C	-1.761809	5.751409	-4.111980	H	-10.800813	-2.404333	0.950504
H	-0.001992	5.537909	-2.931323	H	-11.605350	-2.177385	-0.614898
C	-3.772843	4.686784	-3.310132	B	-7.500419	-2.830549	-0.489491
H	-5.086884	-0.836386	6.656094	C	-7.513051	-4.346211	0.142076
H	3.457274	-5.516458	-5.079019	H	-7.096870	-4.400391	1.161277
H	-2.424229	1.337064	-4.796312	H	-8.537177	-4.744785	0.204576
C	8.117453	-2.025896	2.071828	H	-6.942242	-5.065367	-0.467472
H	7.285650	-0.920725	0.452696	174			

TS-TRANS_d

Eopt -5230.796916

Ir	0.155422	-0.645763	1.009638
C	-1.603940	-0.714758	-0.208386
P	0.423366	1.597003	0.926770
C	-2.202975	-0.405350	1.067662
H	-1.743007	-1.722318	-0.589487
H	-1.657650	0.055864	-0.970529
P	1.756610	-1.268724	-0.626879
N	1.218444	2.187176	2.332885
O	1.245838	2.189593	-0.348441
O	-0.895049	2.585666	1.014159
C	-2.760356	-1.381755	1.918620
H	-2.399362	0.631371	1.304084
N	1.293457	-1.577891	-2.239924
O	2.572900	-2.686602	-0.462397
O	3.017960	-0.192994	-0.547999
C	0.435772	2.034059	3.545546
C	2.590495	1.767612	2.543530
C	1.544888	3.560205	-0.437562
C	-1.528100	3.159294	-0.093500
C	-3.411362	-1.148507	3.188002
H	-2.541088	-2.416838	1.687656
C	1.808874	-2.688137	-3.022477
C	0.278921	-0.821314	-2.926377
C	3.659057	-2.892015	0.377691
C	4.180330	-0.348320	-1.301146
C	-0.012510	3.172080	4.215798
C	0.145535	0.738856	4.028091
C	3.580378	2.747713	2.644683
C	2.902729	0.403935	2.703217
C	0.535385	4.484014	-0.644789
C	2.911389	3.905837	-0.376867
C	-0.860121	4.044920	-0.927437
C	-2.902625	2.864759	-0.244754
C	-3.864183	0.123372	3.612370
C	-3.621686	-2.256681	4.043073
C	3.080763	-2.628775	-3.596708
C	1.001806	-3.832303	-3.204882
C	0.526702	0.505310	-3.294386
C	-0.952575	-1.437308	-3.254463
C	4.879787	-2.270528	0.145182
C	3.471004	-3.856216	1.395156
C	5.077503	-1.372003	-1.028209
C	4.401813	0.618247	-2.308089
C	-0.745897	3.040686	5.395458
H	0.222093	4.148924	3.804678
C	-0.610237	0.630686	5.210310
C	0.622324	-0.487654	3.362555
C	4.903978	2.381276	2.886825
H	3.298901	3.789429	2.536440
C	4.245119	0.054263	2.930904
C	1.867698	-0.643189	2.730011
C	0.900107	5.880741	-0.593502
C	3.274860	5.228223	-0.445147
H	3.647409	3.116617	-0.278756
C	-1.562848	4.512032	-2.101716
C	-3.604457	3.392584	-1.298502
H	-3.393418	2.233375	0.485988
C	-4.485814	0.275157	4.847774
H	-3.733879	0.990045	2.972609
C	-4.232160	-2.096175	5.283865
H	-3.283970	-3.238849	3.724337
C	3.605536	-3.729888	-4.272552
H	3.661612	-1.720824	-3.500778
C	1.564363	-4.945935	-3.860056
C	-0.415713	-3.869554	-2.854663
C	-0.445591	1.248007	-3.963222
H	1.486621	0.948931	-3.054277
C	-1.923595	-0.659886	-3.915571
C	-1.272543	-2.825211	-2.920094
C	5.940663	-2.518105	1.096216
C	4.509020	-4.173801	2.234027
H	2.497278	-4.323835	1.480564
C	6.183197	-1.542294	-1.943261
C	5.515590	0.529394	-3.106047
H	3.683381	1.422615	-2.421940
C	-1.040686	1.767048	5.892188
H	-1.089516	3.927346	5.920503
H	-0.843999	-0.356994	5.597963
H	0.136070	-1.401069	3.691611
C	5.234978	1.029585	3.024278
H	5.671292	3.145991	2.967812
H	4.500223	-0.992130	3.062317
H	2.235935	-1.660186	2.648647
C	-0.064822	6.926993	-0.579872
C	2.286122	6.246014	-0.512457
H	4.323597	5.510879	-0.413854
C	-0.922642	5.246266	-3.141014
C	-2.951646	4.193994	-2.271197
H	-4.663273	3.177216	-1.412397
C	-4.666794	-0.829921	5.688356
H	-4.831880	1.256681	5.158811
H	-4.375354	-2.955868	5.932103
C	2.853126	-4.903359	-4.381936
H	4.604250	-3.673761	-4.696101
H	0.957166	-5.839697	-3.979556
H	-0.837086	-4.858122	-2.683170
C	-1.677837	0.662725	-4.271113
H	-0.244574	2.279351	-4.237029
H	-2.881353	-1.116236	-4.152570
H	-2.333355	-3.038868	-2.800247
C	7.166685	-1.792787	1.091300
C	5.755629	-3.500604	2.126973
H	4.379724	-4.927020	3.006786
C	7.037506	-2.681540	-1.915064
C	6.411493	-0.564731	-2.970659
H	5.703329	1.275398	-3.873602
H	-1.612992	1.658357	6.809041
H	6.263460	0.734613	3.210823
C	0.318557	8.250279	-0.524797
H	-1.119495	6.678249	-0.593625

C	2.648564	7.620496	-0.479706
C	-1.620208	5.666775	-4.254208
H	0.136107	5.463379	-3.066971
C	-3.648034	4.661252	-3.419194
H	-5.151480	-0.703936	6.652609
H	3.262944	-5.772982	-4.888268
H	-2.442792	1.237828	-4.785041
C	8.159758	-2.055799	2.011799
H	7.316666	-1.006435	0.361630
C	6.806635	-3.762571	3.048367
C	8.076566	-2.819451	-2.810771
H	6.854574	-3.465049	-1.189705
C	7.507766	-0.723583	-3.862608
C	1.687817	8.606165	-0.489768
H	-0.440158	9.027898	-0.502893
H	3.704486	7.874944	-0.431329
C	-3.000413	5.387644	-4.392657
H	-1.101067	6.214679	-5.035980
H	-4.702867	4.417257	-3.517508
C	7.990207	-3.062115	2.992031
H	9.080521	-1.479128	1.985916
H	6.646964	-4.521640	3.810288
C	8.330189	-1.824388	-3.784683
H	8.703133	-3.706460	-2.772085
H	7.669569	0.037251	-4.622341
H	1.974875	9.653470	-0.456484
H	-3.538786	5.733286	-5.270707
H	8.784832	-3.265797	3.704414
H	9.159069	-1.941553	-4.477264
Cl	-0.048728	-3.116752	1.415288
C	-6.534973	-2.146525	0.444215
C	-4.958374	-1.924248	0.566379
C	-5.959869	-0.843182	0.881539
H	-6.000064	-0.539134	1.937976
H	-6.071888	-0.011980	0.185701
C	-5.679556	-3.039918	1.276733
H	-5.698862	-2.956339	2.372916
H	-5.538585	-4.063761	0.928391
H	-4.358716	-2.009483	-0.329876
O	-7.826977	-1.260565	-1.627938
O	-8.726875	-3.077740	-0.422887
C	-9.196109	-0.977538	-1.375184
C	-9.802464	-2.401132	-1.053695
C	-9.788770	-0.290587	-2.614294
H	-10.867064	-0.115261	-2.507614
H	-9.307288	0.684415	-2.763673
H	-9.621944	-0.882318	-3.519062
C	-9.320764	-0.007368	-0.180099
H	-8.714125	0.884168	-0.380595
H	-10.354768	0.321492	-0.015643
H	-8.955342	-0.461871	0.745503
C	-11.003467	-2.389730	-0.096575
H	-11.844402	-1.811673	-0.501148
H	-11.354997	-3.416311	0.068008
H	-10.734061	-1.973993	0.878805
C	-10.208627	-3.167303	-2.332342
H	-9.411908	-3.146098	-3.081801
H	-10.394271	-4.215741	-2.069397
H	-11.121852	-2.766578	-2.790832
B	-7.427688	-2.500564	-0.900246
C	-6.597748	-3.511801	-1.895648
H	-6.247175	-4.427319	-1.391742
H	-7.213273	-3.845886	-2.745165
H	-5.703657	-3.034593	-2.330626

174

TS-TRANS_e

Eopt -5230.797904

Ir	0.012133	-0.352712	1.091179
C	-1.766872	-0.178259	-0.076334
P	0.685354	1.782931	0.775807
C	-2.244784	0.320633	1.195261
H	-2.113585	-1.162444	-0.377690
H	-1.711661	0.535851	-0.892165
P	1.432786	-1.411926	-0.491136
N	1.598185	2.348230	2.119519
O	1.576790	2.095214	-0.550961
O	-0.434578	2.993177	0.767807
C	-2.982069	-0.480462	2.093387
H	-2.243194	1.389355	1.366843
N	0.887131	-1.777136	-2.064694
O	1.979719	-2.933949	-0.196447
O	2.870148	-0.582830	-0.517196
C	0.819749	2.443116	3.341403
C	2.876851	1.710269	2.366481
C	2.115207	3.376924	-0.764211
C	-0.972323	3.562782	-0.391028
C	-3.587319	-0.055921	3.333707
H	-2.964449	-1.549367	1.921888
C	1.170864	-3.030694	-2.741332
C	0.004788	-0.914653	-2.806161
C	3.026777	-3.258546	0.655328
C	3.970280	-1.015619	-1.254732
C	0.590985	3.697677	3.906411
C	0.312193	1.268000	3.938181
C	4.026500	2.503330	2.377671
C	2.945081	0.332243	2.648769
C	1.284931	4.446684	-1.051417
C	3.522253	3.473716	-0.735320
C	-0.170638	4.243023	-1.296448
C	-2.377739	3.489699	-0.524090
C	-3.783806	1.302124	3.680071
C	-4.021706	-1.052636	4.240402
C	2.417850	-3.261202	-3.327252
C	0.163389	-4.018017	-2.805844
C	0.479816	0.301321	-3.308044
C	-1.329030	-1.319375	-3.050688
C	4.335072	-2.893440	0.363296
C	2.686706	-4.077228	1.757294
C	4.670173	-2.157655	-0.889593
C	4.346743	-0.200967	-2.346579
C	-0.136858	3.804813	5.091909
H	0.989684	4.576510	3.409406

C	-0.432237	1.402128	5.124796	C	5.010909	-4.075351	2.449998
C	0.551268	-0.079255	3.384261	H	3.416730	-5.143492	3.460054
C	5.268477	1.930419	2.649888	C	6.341726	-3.879537	-1.653233
H	3.932056	3.564680	2.175785	C	6.092531	-1.789279	-2.900577
C	4.208483	-0.228732	2.904799	H	5.717874	0.060618	-3.967930
C	1.743185	-0.509165	2.772823	H	-1.214828	2.729499	6.622565
C	1.896016	5.753576	-1.121486	H	6.319978	0.102889	3.118817
C	4.116486	4.697905	-0.918628	C	1.750921	8.186347	-1.254713
H	4.105340	2.575754	-0.568805	H	0.052844	6.898919	-1.190979
C	-0.795985	4.724237	-2.508109	C	3.930453	7.152713	-1.156249
C	-2.990280	4.039946	-1.621189	C	-0.679832	5.688657	-4.750074
H	-2.958656	3.006190	0.252081	H	1.029619	5.282192	-3.546940
C	-4.377390	1.641936	4.891036	C	-2.840206	5.121971	-3.838062
H	-3.478669	2.087406	2.996301	H	-5.246002	0.914115	6.728264
C	-4.603380	-0.705957	5.456370	H	1.986248	-6.489800	-4.315974
H	-3.880809	-2.098253	3.981388	H	-2.354238	1.429607	-4.814309
C	2.714169	-4.498174	-3.898864	C	7.634078	-3.107977	2.195764
H	3.159133	-2.472623	-3.321548	H	6.963584	-2.079446	0.455438
C	0.495128	-5.271744	-3.357172	C	6.014292	-4.437610	3.390368
C	-1.225779	-3.762699	-2.433577	C	7.322677	-4.285885	-2.532749
C	-0.361726	1.145290	-4.031617	H	6.032393	-4.546698	-0.857730
H	1.513324	0.576173	-3.128306	C	7.125444	-2.226552	-3.774963
C	-2.163647	-0.440326	-3.768549	C	3.162288	8.291106	-1.249441
C	-1.882455	-2.589567	-2.581184	H	1.144884	9.087189	-1.298733
C	5.351293	-3.241697	1.331357	H	5.015551	7.215675	-1.129227
C	3.665622	-4.499714	2.620592	C	-2.089114	5.642779	-4.867319
H	1.646410	-4.351502	1.886842	H	-0.083505	6.071692	-5.573819
C	5.708547	-2.610758	-1.786661	H	-3.922347	5.056047	-3.918438
C	5.410153	-0.565602	-3.134832	C	7.303677	-3.971924	3.266571
H	3.786709	0.708966	-2.532895	H	8.643415	-2.713516	2.114029
C	-0.645475	2.653134	5.700590	H	5.734324	-5.081255	4.220773
H	-0.309974	4.781614	5.534416	C	7.735679	-3.447052	-3.595210
H	-0.831628	0.510643	5.600498	H	7.778664	-5.264793	-2.411545
H	-0.074633	-0.859197	3.807211	H	7.409391	-1.580586	-4.602151
C	5.357068	0.558961	2.907985	H	3.633238	9.268594	-1.304775
H	6.160302	2.550606	2.660235	H	-2.570685	6.003009	-5.772074
H	4.277000	-1.287989	3.129677	H	8.061474	-4.250128	3.993717
H	1.927542	-1.577786	2.791899	H	8.517614	-3.776311	-4.273861
C	1.135123	6.954040	-1.197650	Cl	-0.637197	-2.689184	1.731644
C	3.326206	5.868275	-1.072184	C	-6.788067	-0.697648	0.504424
H	5.199259	4.787947	-0.912595	C	-5.210731	-0.705852	0.696924
C	-0.051565	5.246904	-3.604325	C	-6.045686	0.523833	0.937886
C	-2.220898	4.637798	-2.653462	H	-6.093358	0.857841	1.983812
H	-4.071047	3.996095	-1.723298	H	-5.983722	1.347086	0.226008
C	-4.783586	0.641869	5.783473	C	-6.117073	-1.675017	1.409997
H	-4.526958	2.687925	5.142663	H	-6.166114	-1.550090	2.501236
H	-4.921980	-1.482690	6.145446	H	-6.123000	-2.714085	1.083820
C	1.756132	-5.516621	-3.891036	H	-4.592533	-0.912548	-0.166014
H	3.695476	-4.667590	-4.332892	O	-9.080944	-0.918540	-0.692049
H	-0.268379	-6.045175	-3.385475	O	-7.361713	-2.417475	-1.285503
H	-1.812871	-4.638355	-2.163751	C	-9.627868	-2.227417	-0.672403
C	-1.690791	0.773304	-4.258297	C	-8.602615	-3.057915	-1.543263
H	0.015894	2.090035	-4.410973	C	-11.053375	-2.167590	-1.242228
H	-3.196779	-0.730185	-3.942901	H	-11.503406	-3.165570	-1.322526
H	-2.959612	-2.595176	-2.424044	H	-11.691284	-1.567556	-0.580779
C	6.687841	-2.754587	1.256368	H	-11.070551	-1.699118	-2.230560

C	-9.712909	-2.729714	0.785220
H	-10.259120	-1.987485	1.380095
H	-10.242637	-3.687190	0.870763
H	-8.719819	-2.846214	1.228329
C	-8.483010	-4.539644	-1.158932
H	-9.434797	-5.071605	-1.285485
H	-7.740562	-5.030038	-1.801489
H	-8.154964	-4.662089	-0.122454
C	-8.910954	-2.973297	-3.054627
H	-9.072873	-1.938923	-3.372262
H	-8.050722	-3.364482	-3.611580
H	-9.793220	-3.561013	-3.338736
B	-7.600510	-0.993267	-0.903617
C	-7.152795	0.089296	-2.057191
H	-6.070136	0.084167	-2.267291
H	-7.654061	-0.114773	-3.015674
H	-7.413716	1.126569	-1.790935

174

TS-TRANS_f

Eopt -5230.796153

Ir	0.104092	-0.678403	0.926140
C	-1.604770	-0.664885	-0.368427
P	0.403940	1.559031	0.936497
C	-2.270980	-0.428787	0.890956
H	-1.734059	-1.645263	-0.817201
H	-1.603694	0.149564	-1.085413
P	1.767974	-1.252105	-0.667541
N	1.155595	2.081879	2.394299
O	1.276281	2.199796	-0.281614
O	-0.907038	2.557656	1.023215
C	-2.901004	-1.450425	1.635614
H	-2.461273	0.594386	1.187265
N	1.389457	-1.462135	-2.318031
O	2.548153	-2.694130	-0.540959
O	3.042749	-0.208034	-0.464012
C	0.326791	1.892408	3.570920
C	2.512751	1.634839	2.641389
C	1.598146	3.568366	-0.288135
C	-1.489743	3.199145	-0.073502
C	-3.567873	-1.283407	2.917294
H	-2.666879	-2.470736	1.360521
C	1.931966	-2.531811	-3.137493
C	0.422944	-0.650291	-3.009898
C	3.584833	-2.970753	0.340122
C	4.242947	-0.344170	-1.158331
C	-0.131774	3.009540	4.269010
C	0.002961	0.583656	3.991537
C	3.510143	2.595853	2.822101
C	2.801653	0.261801	2.759313
C	0.610000	4.518873	-0.477422
C	2.966580	3.888066	-0.163397
C	-0.780700	4.119244	-0.832507
C	-2.860857	2.931850	-0.290706
C	-3.986477	-0.030832	3.417899
C	-3.822812	-2.437486	3.691697
C	3.233350	-2.459247	-3.639873
C	1.120466	-3.649199	-3.432089
C	0.710555	0.689507	-3.289262
C	-0.799502	-1.224396	-3.433030
C	4.826667	-2.361503	0.209218
C	3.327438	-3.991142	1.284999
C	5.104258	-1.401310	-0.897234
C	4.540614	0.675651	-2.090746
C	-0.910750	2.842720	5.414454
H	0.130467	3.998401	3.906137
C	-0.798723	0.440206	5.139295
C	0.488160	-0.622887	3.295898
C	4.818164	2.201588	3.102199
H	3.246462	3.644948	2.745340
C	4.129123	-0.115639	3.026745
C	1.753649	-0.770648	2.703628
C	0.992588	5.904283	-0.333808
C	3.351214	5.206093	-0.144429
H	3.687531	3.082999	-0.084987
C	-1.434585	4.661592	-2.002680
C	-3.517396	3.526634	-1.337862
H	-3.383432	2.267006	0.386376
C	-4.642686	0.055862	4.643174
H	-3.809503	0.873906	2.845578
C	-4.467217	-2.344682	4.921077
H	-3.509977	-3.406792	3.312146
C	3.777686	-3.525311	-4.355305
H	3.820883	-1.568992	-3.457790
C	1.701131	-4.729588	-4.125927
C	-0.313356	-3.684583	-3.157187
C	-0.215282	1.487090	-3.960884
H	1.664890	1.098312	-2.976544
C	-1.724407	-0.392719	-4.094432
C	-1.152577	-2.624776	-3.200067
C	5.830500	-2.685319	1.198810
C	4.313311	-4.378010	2.156854
H	2.343337	-4.444245	1.290371
C	6.256099	-1.542428	-1.758724
C	5.697026	0.610040	-2.828069
H	3.845465	1.501481	-2.195868
C	-1.240772	1.554906	5.848798
H	-1.262672	3.713054	5.960906
H	-1.061361	-0.558635	5.476934
H	-0.020913	-1.541498	3.571307
C	5.126570	0.841012	3.198510
H	5.590944	2.951685	3.244541
H	4.365491	-1.169945	3.125853
H	2.111543	-1.788892	2.595205
C	0.042376	6.963175	-0.291112
C	2.379981	6.242006	-0.185057
H	4.402282	5.469351	-0.062210
C	-0.748821	5.445242	-2.974590
C	-2.819997	4.372683	-2.239449
H	-4.573796	3.332860	-1.501972
C	-4.884546	-1.096935	5.398132
H	-4.967137	1.024910	5.011742
H	-4.652514	-3.242169	5.504483

C	3.015880	-4.676622	-4.577212
H	4.797926	-3.460043	-4.722506
H	1.088657	-5.603664	-4.332535
H	-0.755601	-4.674965	-3.068811
C	-1.440131	0.943272	-4.360688
H	0.015461	2.528176	-4.165736
H	-2.675534	-0.817109	-4.405748
H	-2.220197	-2.830106	-3.147182
C	7.066734	-1.985034	1.301920
C	5.574192	-3.722979	2.157717
H	4.130497	-5.173870	2.873968
C	7.084637	-2.700881	-1.752471
C	6.562256	-0.510503	-2.709808
H	5.943682	1.396881	-3.535853
H	-1.849467	1.418496	6.738143
H	6.142828	0.524405	3.414433
C	0.442085	8.274574	-0.143996
H	-1.014552	6.732372	-0.355700
C	2.760272	7.606235	-0.058433
C	-1.400908	5.938645	-4.085435
H	0.308945	5.643620	-2.850632
C	-3.468951	4.914245	-3.382583
H	-5.397797	-1.023374	6.353025
H	3.439692	-5.519844	-5.115646
H	-2.169363	1.560837	-4.877334
C	8.004343	-2.320991	2.256281
H	7.268516	-1.160543	0.628963
C	6.569661	-4.059079	3.116072
C	8.171098	-2.808466	-2.594484
H	6.844207	-3.522338	-1.088410
C	7.705399	-0.640367	-3.545837
C	1.814168	8.605872	-0.041325
H	-0.305958	9.061591	-0.101075
H	3.817390	7.840418	0.040692
C	-2.778291	5.686795	-4.288668
H	-0.847607	6.523892	-4.814988
H	-4.522292	4.690647	-3.531984
C	7.765798	-3.379649	3.164322
H	8.934617	-1.762081	2.313146
H	6.356882	-4.858595	3.821580
C	8.500604	-1.762305	-3.488880
H	8.776791	-3.710473	-2.574972
H	7.926297	0.161156	-4.246509
H	2.114409	9.644629	0.064677
H	-3.280562	6.090185	-5.163462
H	8.517442	-3.640465	3.904188
H	9.366314	-1.856135	-4.138619
Cl	-0.149552	-3.156843	1.242020
C	-6.218035	-1.450266	-0.636650
C	-4.990054	-1.899730	0.319625
C	-4.806253	-1.395690	-1.094521
H	-4.283388	-0.431934	-1.161245
H	-4.504902	-2.098385	-1.871146
C	-6.027916	-0.873349	0.718843
H	-5.633119	0.145235	0.841743
H	-6.771895	-1.144244	1.465969
H	-4.996326	-2.939773	0.619834
O	-8.327588	-1.601705	-2.106219
O	-8.349380	-2.714305	-0.027956
C	-9.524230	-1.236005	-1.435205
C	-9.690431	-2.377147	-0.354839
C	-10.657069	-1.159987	-2.469804
H	-11.627316	-0.958030	-1.997957
H	-10.456759	-0.346879	-3.179135
H	-10.737337	-2.086948	-3.044917
C	-9.355082	0.161689	-0.801486
H	-9.023599	0.861661	-1.578300
H	-10.290924	0.547050	-0.377000
H	-8.597870	0.155285	-0.012320
C	-10.419035	-1.949640	0.927068
H	-11.446499	-1.622582	0.721637
H	-10.472966	-2.797085	1.622260
H	-9.893839	-1.136792	1.437234
C	-10.399779	-3.625220	-0.924219
H	-9.959872	-3.933884	-1.877209
H	-10.278152	-4.452862	-0.214898
H	-11.474710	-3.465978	-1.077813
B	-7.459279	-2.397915	-1.181868
C	-6.877526	-3.719504	-1.962697
H	-6.227812	-4.349777	-1.333558
H	-7.694089	-4.369161	-2.312818
H	-6.288920	-3.459778	-2.857539

34

3b-Cis_c

Eopt -606.632346

C	-2.103125	0.146973	-0.030093
C	-2.678278	-1.091847	0.587511
C	-2.988848	0.239961	1.193798
H	-4.017159	0.624933	1.128120
H	-2.485210	0.501976	2.125990
C	-3.023609	-0.748698	-0.828203
H	-4.055289	-0.447234	-1.062533
H	-2.550464	-1.330403	-1.621408
H	-2.184376	-1.962210	1.005391
O	0.189408	-0.103190	-1.263549
O	0.184630	0.815215	0.913121
C	1.258284	-0.693771	-0.543454
C	1.481676	0.301675	0.666255
C	2.463419	-0.837894	-1.486375
H	2.218482	-1.538587	-2.295056
H	2.728462	0.117654	-1.948411
H	3.347589	-1.228654	-0.965854
C	0.847385	-2.106155	-0.074117
H	0.491705	-2.675985	-0.941269
H	1.681112	-2.661745	0.374941
H	0.033120	-2.062072	0.653806
C	2.001407	-0.356851	1.953506
H	1.299652	-1.106481	2.331024
H	2.976975	-0.837695	1.804387
H	2.122842	0.404135	2.735192
C	2.434394	1.463852	0.304582
H	3.482492	1.144938	0.234080

H	2.155663	1.931263	-0.644154
H	2.366249	2.229686	1.086978
B	-0.620405	0.771576	-0.352017
C	-0.830517	2.256150	-1.040489
H	-1.377536	2.950432	-0.380480
H	0.118242	2.752282	-1.300637
H	-1.415962	2.199326	-1.974119

34
3b-TS-rotation_a
Eopt -606.628374

C	-2.123447	-0.092185	-0.218548
C	-2.924487	-0.631122	0.937279
C	-3.501562	0.461281	0.100201
H	-4.367031	0.231918	-0.539207
H	-3.537012	1.465583	0.525120
C	-2.544924	-1.548509	-0.176347
H	-3.337743	-1.919801	-0.842290
H	-1.762855	-2.284160	0.008928
H	-2.651646	-0.648433	1.986394
O	0.273804	-0.018560	-1.296101
O	0.080512	0.582313	0.971703
C	1.394078	-0.554732	-0.615725
C	1.440395	0.267265	0.736799
C	2.633940	-0.391425	-1.510119
H	2.774156	0.650273	-1.813484
H	3.550422	-0.734770	-1.012346
H	2.510238	-0.986882	-2.423978
C	1.176476	-2.064873	-0.375755
H	0.914743	-2.539463	-1.329210
H	2.072117	-2.564373	0.016457
H	0.355127	-2.242683	0.323949
C	1.976050	-0.514656	1.947228
H	3.008627	-0.854273	1.793454
H	1.968397	0.129916	2.835661
H	1.354230	-1.386772	2.169306
C	2.267234	1.567701	0.614056
H	1.988109	2.145122	-0.271266
H	2.071257	2.191614	1.494688
H	3.347266	1.376057	0.569456
B	-0.656552	0.666109	-0.334791
C	-0.927413	2.216794	-0.829500
H	0.000314	2.788984	-0.985687
H	-1.478403	2.253245	-1.784857
H	-1.530117	2.795086	-0.109372

34
3b-TS-rotation_b
Eopt -606.629006

C	-2.104093	0.045158	0.293503
C	-2.976777	-0.944773	-0.430944
C	-2.485988	-1.271923	0.940155
H	-3.214017	-1.299816	1.764072
H	-1.699586	-2.019954	1.039883
C	-3.518469	0.411114	-0.122779
H	-4.322751	0.498048	0.622688
H	-3.617775	1.121123	-0.945147
H	-2.788969	-1.433030	-1.380668
O	0.096421	0.081785	-1.175296
O	0.269079	0.577811	1.119954
C	1.271195	-0.543593	-0.693453
C	1.558996	0.205203	0.672417
C	2.376426	-0.393105	-1.751777
H	2.529865	0.655062	-2.024623
H	3.334344	-0.806815	-1.410031
H	2.089512	-0.932175	-2.663959
C	1.005290	-2.052618	-0.498066
H	0.598540	-2.462094	-1.430849
H	1.914528	-2.615884	-0.250122
H	0.270073	-2.229795	0.291913
C	2.215847	-0.665069	1.755612
H	3.194954	-1.047963	1.439437
H	2.370108	-0.068986	2.664250
H	1.581364	-1.514679	2.024053
C	2.432135	1.467630	0.486219
H	2.056965	2.105604	-0.318616
H	2.405090	2.051691	1.414348
H	3.480913	1.226075	0.269502
B	-0.649602	0.751791	-0.052299
C	-0.920818	2.330972	-0.444026
H	0.007862	2.882330	-0.658177
H	-1.551804	2.437094	-1.342898
H	-1.437665	2.884472	0.358005

34
3b-Trans_a
Eopt -606.632253

C	2.036378	-0.091187	0.294601
C	3.327366	-0.437841	-0.398851
C	3.353674	0.499876	0.761903
H	3.817203	0.164036	1.700494
H	3.513575	1.559729	0.558305
C	2.524689	-1.524576	0.239808
H	2.929164	-2.022892	1.132915
H	1.966257	-2.192805	-0.415878
H	3.604780	-0.283929	-1.436100
O	-0.275251	0.853815	0.931804
O	-0.130451	-0.284612	-1.128982
C	-1.579386	0.421627	0.592126
C	-1.318923	-0.709675	-0.483018
C	-2.390982	1.606158	0.017890
H	-3.450071	1.358223	-0.131288
H	-2.336992	2.444336	0.723576
H	-1.984654	1.948953	-0.937671
C	-2.284237	-0.056179	1.871705
H	-2.414893	0.790147	2.558450
H	-3.279191	-0.471155	1.663497
H	-1.694671	-0.815120	2.394208
C	-1.092542	-2.092129	0.167578
H	-2.015096	-2.516613	0.584683
H	-0.720181	-2.786081	-0.595916
H	-0.347495	-2.040520	0.966967
C	-2.422398	-0.857332	-1.542581
H	-3.390095	-1.121020	-1.095968
H	-2.547260	0.062745	-2.121053

H	-2.153885	-1.655117	-2.247160
B	0.653902	0.625368	-0.228518
C	1.030429	2.029078	-1.011543
H	1.540167	2.757445	-0.359804
H	1.696478	1.869487	-1.876524
H	0.138551	2.542762	-1.403408

34
3b-Trans_b
Eopt -606.633548

C	-2.021266	-0.223493	-0.170935
C	-3.228408	-0.263328	0.730120
C	-3.409544	0.044082	-0.719570
H	-3.913981	-0.696779	-1.356036
H	-3.635558	1.074496	-0.997994
C	-2.391077	-1.490639	0.573322
H	-2.818842	-2.355435	0.045882
H	-1.733319	-1.763171	1.398912
H	-3.436252	0.338508	1.608283
O	0.293261	0.366662	-1.264390
O	0.121972	0.341022	1.089674
C	1.392432	-0.370371	-0.763108
C	1.473611	0.084833	0.749582
C	2.634597	-0.028781	-1.601391
H	2.810577	1.050491	-1.634815
H	3.539072	-0.516443	-1.214602
H	2.489169	-0.370985	-2.634094
C	1.116036	-1.883460	-0.914910
H	0.854841	-2.088100	-1.960582
H	1.986944	-2.500756	-0.658012
H	0.274500	-2.203981	-0.294598
C	2.032403	-0.974702	1.711405
H	3.062497	-1.257051	1.457282
H	2.040249	-0.578641	2.735127
H	1.417200	-1.879334	1.713313
C	2.296734	1.380328	0.930262
H	1.989540	2.152011	0.218379
H	2.121555	1.768936	1.941000
H	3.376018	1.216987	0.813373
B	-0.658138	0.693022	-0.144852
C	-1.059353	2.289360	-0.236226
H	-0.175256	2.941674	-0.158733
H	-1.545500	2.547809	-1.191501
H	-1.751602	2.610201	0.560087

37
3c-Cis_d
Eopt -645.948498

C	-1.859790	0.617430	-0.071114
C	-2.809681	-0.517849	0.133655
C	-2.848381	0.639407	1.082212
H	-3.714699	1.318547	1.077165
H	-2.425767	0.484417	2.077701
C	-2.902048	0.288543	-1.125701
H	-3.772371	0.942369	-1.290192
H	-2.528033	-0.171573	-2.043550
O	0.464886	0.322988	-1.257258
O	0.439678	0.582969	1.088540
C	1.433640	-0.560169	-0.719187
C	1.683541	0.001043	0.741376
C	2.669781	-0.544860	-1.633056
H	3.484001	-1.165203	-1.235868
H	2.402079	-0.941527	-2.620916
H	3.047956	0.471234	-1.778819
C	0.865274	-1.994585	-0.707630
H	0.503342	-2.238198	-1.714153
H	1.614891	-2.746278	-0.427324
H	0.021708	-2.079801	-0.018888
C	2.029706	-1.067847	1.790298
H	2.176954	-0.590602	2.767653
H	1.223055	-1.798571	1.900179
H	2.953908	-1.605359	1.540971
C	2.792629	1.077766	0.781109
H	2.646546	1.840669	0.011455
H	2.761441	1.578149	1.756781
H	3.796168	0.651647	0.652552
B	-0.271446	1.025958	-0.155511
C	-0.204604	2.660468	-0.394691
H	0.824591	3.049016	-0.455799
H	-0.704464	2.961735	-1.331087
H	-0.704024	3.224429	0.411320
C	-2.737563	-1.996460	0.376226
H	-2.273699	-2.519294	-0.469294
H	-2.151153	-2.225254	1.276015
H	-3.736741	-2.435323	0.520414

37
3c-TS-rotation_a
Eopt -645.946844

C	-1.867591	0.455148	-0.470386
C	-2.839256	-0.563131	0.049038
C	-3.230430	0.878735	0.059405
H	-4.020524	1.230456	-0.621638
H	-3.242179	1.386499	1.026521
C	-2.423164	-0.626248	-1.383869
H	-3.150923	-0.374331	-2.170253
H	-1.736561	-1.421944	-1.676208
O	0.651717	0.725907	-1.180288
O	0.206342	0.132789	1.056287
C	1.616716	-0.234540	-0.788964
C	1.555902	-0.202639	0.792117
C	2.976026	0.162446	-1.386980
H	3.253714	1.183312	-1.108678
H	3.779598	-0.514520	-1.068256
H	2.923793	0.122265	-2.482673
C	1.229379	-1.615550	-1.362386
H	1.064872	-1.515078	-2.442070
H	2.009857	-2.372288	-1.208526
H	0.302726	-1.987366	-0.915936
C	1.871822	-1.542640	1.475867
H	2.890328	-1.886710	1.253167
H	1.791279	-1.428923	2.564598
H	1.170016	-2.325718	1.174619
C	2.492947	0.866610	1.400717
H	2.378468	1.835647	0.908065

H 2.234506 0.997348 2.458736
H 3.550901 0.578735 1.345013
B -0.341222 0.958663 -0.076534
C -0.414246 2.568029 0.278721
H 0.569655 3.008179 0.504878
H -0.830953 3.155332 -0.557689
H -1.058624 2.781139 1.148129
C -2.768633 -1.575702 1.152352
H -1.965803 -2.301550 0.972438
H -2.564015 -1.092771 2.116325
H -3.705322 -2.143705 1.260322
37
3c-TS-rotation_b
Eopt -645.947056
C -1.824473 0.633342 0.247881
C -2.846773 -0.461544 0.189332
C -2.424049 0.062783 1.523614
H -3.133509 0.651128 2.125795
H -1.764892 -0.563955 2.127630
C -3.167684 0.861183 -0.428384
H -3.934980 1.502955 0.031549
H -3.161106 0.916987 -1.519912
O 0.316516 -0.011331 -1.198036
O 0.626888 0.882745 0.961573
C 1.341158 -0.754773 -0.562089
C 1.814322 0.199562 0.605589
C 2.423055 -1.087148 -1.601134
H 2.789652 -0.184975 -2.099742
H 3.279069 -1.607877 -1.152029
H 2.004060 -1.743950 -2.374275
C 0.764149 -2.082667 -0.025854
H 0.263885 -2.609288 -0.847164
H 1.538360 -2.747660 0.378703
H 0.024158 -1.901781 0.759017
C 2.353742 -0.520287 1.851307
H 3.234233 -1.134897 1.622714
H 2.652335 0.219725 2.605013
H 1.591930 -1.161395 2.304568
C 2.884624 1.213583 0.141330
H 2.597400 1.705499 -0.791875
H 2.989890 1.988787 0.910166
H 3.868173 0.748611 -0.006172
B -0.279227 0.977426 -0.229559
C -0.295079 2.475449 -0.920090
H 0.704572 2.824606 -1.223681
H -0.922144 2.507920 -1.827243
H -0.703562 3.244082 -0.242048
C -2.906331 -1.848260 -0.378854
H -2.648122 -1.850493 -1.446213
H -2.209472 -2.521901 0.133590
H -3.910987 -2.287905 -0.284767
37
3c-Trans_a
Eopt -645.950516
C -1.693168 -0.014705 -0.610013
C -3.086986 -0.346672 -0.162594
C -2.900250 0.611991 -1.292804
H -3.185323 0.312653 -2.312072
H -3.084831 1.668945 -1.089495
C -2.217501 -1.437884 -0.699561
H -2.459582 -1.895160 -1.670688
H -1.803852 -2.144956 0.021098
O 0.686544 0.914903 -0.929314
O 0.295976 -0.317698 1.044845
C 1.935086 0.430202 -0.472135
C 1.532140 -0.749286 0.501078
C 2.699768 1.559440 0.256695
H 3.731515 1.277874 0.505083
H 2.738516 2.437088 -0.400174
H 2.196343 1.856847 1.180956
C 2.769103 0.003581 -1.690281
H 2.999805 0.882227 -2.306505
H 3.722600 -0.455054 -1.397034
H 2.222877 -0.704704 -2.319849
C 1.334776 -2.084216 -0.251097
H 2.282332 -2.514503 -0.600530
H 0.868541 -2.808536 0.428101
H 0.674006 -1.960970 -1.114260
C 2.514478 -0.994340 1.657071
H 3.513703 -1.271443 1.296204
H 2.610310 -0.111509 2.296127
H 2.148569 -1.817136 2.284823
B -0.371780 0.647851 0.107581
C -0.812765 2.013635 0.922960
H -1.316791 2.756220 0.282571
H -1.506965 1.799480 1.752907
H 0.049277 2.532889 1.370453
C -3.808508 -0.213166 1.146033
H -3.884145 0.838389 1.452000
H -4.833184 -0.612732 1.102242
H -3.282022 -0.754284 1.943777
37
3c-Trans_b
Eopt -645.951632
C -1.693209 -0.539788 -0.338173
C -3.008199 -0.305148 0.349101
C -3.005244 -0.729399 -1.083519
H -3.357163 -1.736069 -1.352807
H -3.259092 0.026643 -1.829548
C -2.099729 -1.374152 0.864483
H -2.387317 -2.429998 0.751983
H -1.562441 -1.164901 1.790724
O 0.730956 -0.317277 -1.331892
O 0.232607 0.762946 0.706731
C 1.769936 -0.622652 -0.421101
C 1.624757 0.500354 0.683475
C 3.107378 -0.604667 -1.178571
H 3.255809 0.341211 -1.707836
H 3.962059 -0.764985 -0.508166
H 3.118927 -1.406978 -1.927523
C 1.562361 -2.046153 0.144264
H 1.470412 -2.744993 -0.696405

H 2.400271 -2.380266 0.770195
H 0.643450 -2.115171 0.732927
C 2.071454 0.078694 2.091754
H 3.134915 -0.192789 2.121078
H 1.922270 0.910460 2.792401
H 1.488481 -0.770668 2.460223
C 2.379175 1.795845 0.307614
H 2.149798 2.109631 -0.715091
H 2.057284 2.597435 0.983486
H 3.468525 1.694414 0.398566
B -0.379990 0.405719 -0.618671
C -0.817006 1.721842 -1.510910
H 0.038600 2.387984 -1.705077
H -1.221240 1.449379 -2.500064
H -1.587742 2.340442 -1.021664
C -3.602883 0.892665 1.029062
H -3.738141 1.722527 0.322939
H -4.588017 0.679469 1.471290
H -2.950952 1.248633 1.838129
41
CIS_2MeThiofuranBoryl_a
Eopt -1158.463769
C -0.475503 2.117431 0.032851
C -1.583389 3.108772 -0.160772
C -0.753108 3.167052 1.083547
H -0.074343 4.019681 1.227909
H -1.205016 2.808614 2.009982
C -0.457962 3.059829 -1.147570
H 0.245172 3.903715 -1.191172
H -0.658528 2.608388 -2.120564
H -2.647506 2.948313 -0.293071
O -0.850413 -0.189308 -1.106974
O -1.221836 -0.023122 1.217003
C -1.989319 -0.980921 -0.790640
C -1.853323 -1.215670 0.768060
C -1.949211 -2.259859 -1.639184
H -2.776240 -2.938731 -1.394053
H -2.036420 -2.000435 -2.701895
H -1.006959 -2.799640 -1.507962
C -3.263986 -0.192853 -1.157540
H -3.188298 0.132794 -2.202054
H -4.176059 -0.794898 -1.056121
H -3.370243 0.700136 -0.535008
C -3.185477 -1.366592 1.514909
H -3.758359 -2.231100 1.155796
H -2.993588 -1.516147 2.584921
H -3.806719 -0.472109 1.412971
C -0.965233 -2.432144 1.107756
H -0.014790 -2.397665 0.568465
H -0.739473 -2.414438 2.180815
H -1.459166 -3.385528 0.881318
B -0.358955 0.493178 0.123637
C 1.235481 0.115062 0.358054
C 2.012573 0.141008 1.492542
C 3.397891 -0.176676 1.297931
H 1.596723 0.385755 2.467784
C 3.719714 -0.452606 -0.004575
H 4.132062 -0.202081 2.100800
S 2.275059 -0.325894 -0.996268
C 5.054668 -0.822470 -0.581445
H 5.399398 -0.096297 -1.330378
H 5.808796 -0.861884 0.212881
H 5.038466 -1.804769 -1.073047
41
CIS_2MeThiofuranBoryl_b
Eopt -1158.463866
C 0.376337 2.088511 0.004677
C 1.442905 3.133974 -0.133741
C 0.369517 3.036865 -1.172212
H -0.371518 3.845452 -1.245264
H 0.637140 2.600880 -2.136101
C 0.554192 3.142336 1.071346
H -0.170389 3.960635 1.188742
H 0.980457 2.799409 2.015606
H 2.518056 3.021553 -0.216874
O 1.185847 -0.082654 1.192916
O 0.929043 -0.120255 -1.152376
C 2.252635 -0.850593 0.648294
C 1.729362 -1.239052 -0.792759
C 2.522061 -2.041910 1.578337
H 3.295836 -2.708220 1.175458
H 2.871612 -1.678520 2.553079
H 1.614987 -2.628173 1.750508
C 3.522297 0.023567 0.593186
H 3.705086 0.445390 1.588902
H 4.413624 -0.544955 0.298301
H 3.397688 0.855856 -0.105492
C 2.826930 -1.410302 -1.851407
H 3.525134 -2.215190 -1.588181
H 2.371758 -1.667295 -2.816213
H 3.398151 -0.488052 -1.991213
C 0.860299 -2.514739 -0.781668
H 0.080750 -2.457985 -0.017159
H 0.366473 -2.612905 -1.755976
H 1.452490 -3.422519 -0.609749
B 0.347335 0.459476 0.086685
C -1.214927 -0.020769 0.346945
C -1.834980 -0.428993 1.503984
C -3.237645 -0.707685 1.386634
H -1.284302 -0.534496 2.435414
C -3.732274 -0.516311 0.124276
H -3.859676 -1.044500 2.213503
C -5.132526 -0.718652 -0.375154
H -5.185555 -1.475483 -1.169685
H -5.777986 -1.053375 0.444869
H -5.566005 0.204329 -0.783665
S -2.430919 0.016661 -0.930687
41
CIS_4ClPhBoryl_a
Eopt -1257.984267
C -0.930095 2.104205 0.035815
C -2.108192 3.031829 0.004263

C	-1.172466	3.072352	1.171427
H	-0.535188	3.957612	1.308278
H	-1.515741	2.632475	2.109035
C	-1.077567	3.112550	-1.078898
H	-0.432820	4.001392	-1.130973
H	-1.341164	2.707680	-2.057440
H	-3.167586	2.806818	-0.044946
O	-1.357734	-0.163222	-1.170934
O	-1.389646	-0.131173	1.190203
C	-2.404583	-1.022138	-0.736257
C	-2.025569	-1.330431	0.768347
C	-2.442414	-2.252865	-1.653604
H	-3.192134	-2.984387	-1.325316
H	-2.703613	-1.945538	-2.674208
H	-1.469391	-2.750440	-1.697624
C	-3.751259	-0.279774	-0.862385
H	-3.847713	0.104664	-1.884986
H	-4.611908	-0.931422	-0.664131
H	-3.799900	0.572048	-0.178264
C	-3.222037	-1.595713	1.691987
H	-3.800625	-2.470681	1.369102
H	-2.865728	-1.790652	2.711416
H	-3.893382	-0.733222	1.736403
C	-1.038955	-2.511393	0.896922
H	-0.183692	-2.395592	0.225259
H	-0.654583	-2.539907	1.923663
H	-1.514233	-3.478008	0.687441
B	-0.717468	0.484466	0.011836
C	0.908192	0.188755	-0.008145
C	1.607035	-0.086888	-1.197546
C	1.684662	0.245680	1.164707
C	2.991018	-0.289198	-1.234989
H	1.046783	-0.158823	-2.127915
C	3.068749	0.047279	1.168887
H	1.191093	0.442526	2.115288
C	3.706121	-0.216645	-0.041958
H	3.501888	-0.501906	-2.170079
H	3.639967	0.093714	2.091952
Cl	5.468247	-0.471243	-0.062748

47

CIS_CyBoryl_a

Eopt -801.992730

C	-0.324826	2.065430	-0.143570
C	-1.450768	3.052382	-0.057535
C	-0.367946	3.093433	0.970436
H	0.319387	3.952275	0.982204
H	-0.596580	2.706775	1.965176
C	-0.571751	3.048467	-1.268733
H	0.096630	3.905572	-1.439011
H	-0.977189	2.625630	-2.189904
H	-2.520779	2.908349	0.044978
O	-0.872103	-0.267981	-1.201546
O	-0.635626	-0.145436	1.137960
C	-2.008165	-0.913161	-0.653702
C	-1.636636	-1.116149	0.882966
C	-2.238915	-2.220657	-1.431505
H	-3.086822	-2.793118	-1.033728
H	-2.460135	-1.985626	-2.480290
H	-1.353404	-2.862089	-1.418504
C	-3.257258	-0.028924	-0.864996
H	-3.341678	0.209109	-1.932473
H	-4.184048	-0.530720	-0.558096
H	-3.183550	0.914373	-0.320078
C	-2.805174	-0.877067	1.856483
H	-3.634002	-1.577764	1.691373
H	-2.453857	-1.015611	2.886925
H	-3.193499	0.142368	1.776720
C	-1.063474	-2.517667	1.187788
H	-0.249905	-2.782077	0.507646
H	-0.656570	-2.514689	2.206392
H	-1.825428	-3.305930	1.131569
B	-0.092228	0.429229	-0.133982
C	1.537632	0.170580	-0.359275
C	2.405887	0.671816	0.814730
C	1.948040	-1.276805	-0.696175
H	1.822341	0.780071	-1.241346
C	3.918322	0.524602	0.566365
H	2.136280	0.104410	1.720435
H	2.177682	1.722205	1.038682
C	3.452649	-1.430206	-0.980868
H	1.693658	-1.932073	0.151796
H	1.363085	-1.639337	-1.551581
C	4.297776	-0.916794	0.194904
H	4.491578	0.849266	1.447990
H	4.212411	1.194145	-0.257402
H	3.705402	-2.479774	-1.195285
H	3.710927	-0.857888	-1.885945
H	5.369780	-0.984236	-0.040323
H	4.128911	-1.568092	1.066799

47

CIS_CyBoryl_d

Eopt -801.995880

C	-0.117605	1.915796	-0.112876
C	0.754604	3.132206	-0.230794
C	-0.238141	2.838668	-1.308544
H	-1.102310	3.508348	-1.429124
H	0.141096	2.444197	-2.252792
C	-0.174060	3.006341	0.935291
H	-1.032153	3.691078	1.004326
H	0.260448	2.756062	1.904828
H	1.835217	3.215788	-0.269792
O	0.906062	-0.107480	1.185880
O	0.923823	-0.169364	-1.175025
C	2.184802	-0.568128	0.784001
C	1.969037	-1.012644	-0.722867
C	2.610963	-1.701562	1.731253
H	3.570823	-2.145986	1.437269
H	2.726129	-1.306192	2.748645
H	1.859189	-2.495192	1.770372
C	3.212361	0.576279	0.924348
H	3.152203	0.979175	1.942871
H	4.243847	0.240714	0.754755

H	3.000553	1.393089	0.229709
C	3.190024	-0.804883	-1.633536
H	4.057945	-1.388114	-1.299210
H	2.946140	-1.127710	-2.653823
H	3.479077	0.248887	-1.682650
C	1.531382	-2.489739	-0.851380
H	0.692557	-2.722112	-0.189331
H	1.201912	-2.669841	-1.882043
H	2.346309	-3.191369	-0.630516
B	0.093379	0.288813	-0.011509
C	-1.373357	-0.487616	0.052979
C	-2.221752	-0.126589	1.289915
C	-2.230922	-0.334295	-1.219656
H	-1.143971	-1.566792	0.144496
C	-3.542889	-0.912340	1.371113
H	-2.458892	0.949476	1.271321
H	-1.631945	-0.291553	2.202883
C	-3.552098	-1.121833	-1.159789
H	-2.469446	0.730577	-1.376641
H	-1.647412	-0.647116	-2.097044
C	-4.371304	-0.750890	0.086711
H	-4.134933	-0.594498	2.242727
H	-3.319847	-1.980604	1.520569
H	-4.151315	-0.952147	-2.067175
H	-3.329192	-2.200208	-1.132493
H	-5.286822	-1.357458	0.140279
H	-4.697461	0.297564	0.001271

47

CIS_CyBoryl_e

Eopt -801.992831

C	0.359253	2.046221	-0.209673
C	1.543306	2.962781	-0.312263
C	0.520661	2.968394	-1.401634
H	-0.113880	3.858164	-1.527244
H	0.782861	2.484401	-2.344157
C	0.603186	3.108399	0.842043
H	-0.024233	4.010257	0.901536
H	0.932048	2.747104	1.818026
H	2.604800	2.740733	-0.342102
O	0.515937	-0.225754	1.104020
O	0.899146	-0.253417	-1.218001
C	1.755139	-0.886354	0.923741
C	1.833524	-1.155316	-0.649423
C	1.745256	-2.160575	1.786574
H	2.669837	-2.741977	1.677737
H	1.650516	-1.881462	2.843545
H	0.899130	-2.808564	1.541289
C	2.895197	0.014052	1.450109
H	2.688138	0.263784	2.498125
H	3.875304	-0.478414	1.410188
H	2.959851	0.951124	0.892707
C	3.218282	-0.884413	-1.268356
H	3.994239	-1.544099	-0.858224
H	3.171836	-1.060001	-2.350649
H	3.532684	0.152206	-1.117764
C	1.430516	-2.593684	-1.042798
H	0.456790	-2.870870	-0.630761
H	1.354722	-2.649185	-2.135881
H	2.166172	-3.341346	-0.719139
B	0.070451	0.418164	-0.166247
C	-1.560134	0.189312	-0.386633
C	-1.979736	-1.274973	-0.620531
C	-2.394132	0.774895	0.773717
H	-1.850606	0.747772	-1.299406
C	-3.495419	-1.459754	-0.814509
H	-1.659965	-1.880145	0.243498
H	-1.445786	-1.677210	-1.491079
C	-3.913204	0.601800	0.595998
H	-2.088152	0.281374	1.710363
H	-2.163942	1.841061	0.905512
C	-4.290530	-0.867748	0.358482
H	-3.745160	-2.524269	-0.940276
H	-3.804810	-0.957470	-1.744872
H	-4.454591	0.993822	1.470327
H	-4.245659	1.199360	-0.267668
H	-5.371331	-0.965168	0.181171
H	-4.070721	-1.446653	1.269215

38

CIS_EneBoryl_a

Eopt -684.033487

C	1.874026	-0.705649	-0.160352
C	2.076220	-2.186560	-0.293638
C	2.580608	-1.276412	-1.368730
H	3.666387	-1.163364	-1.500739
H	2.021592	-1.242045	-2.305259
C	2.673594	-1.465517	0.873990
H	3.767524	-1.370172	0.933082
H	2.194461	-1.591981	1.846336
H	1.348528	-2.989478	-0.331273
O	-0.242304	0.042534	1.173685
O	-0.329765	0.097223	-1.185556
C	-1.506262	-0.466341	0.776467
C	-1.666330	0.063045	-0.706385
C	-2.567220	0.047165	1.761474
H	-3.580734	-0.258180	1.470548
H	-2.371264	-0.362294	2.760729
H	-2.543197	1.137766	1.842142
C	-1.484366	-2.008065	0.850860
H	-1.151030	-2.308258	1.851789
H	-2.472515	-2.453148	0.676627
H	-0.783863	-2.429688	0.125014
C	-2.489119	-0.847974	-1.628967
H	-3.520266	-0.967941	-1.272190
H	-2.534189	-0.410252	-2.634273
H	-2.037337	-1.839870	-1.721764
C	-2.275964	1.481153	-0.761694
H	-1.739215	2.174635	-0.108383
H	-2.194214	1.861505	-1.787127
H	-3.337467	1.491019	-0.483213
B	0.596762	0.314883	-0.038601
C	1.194799	1.848495	0.049972
C	1.194689	2.705516	-0.984142

H 0.751857 2.434155 -1.942670
C 1.818212 2.274272 1.362696
H 2.194289 3.308070 1.352159
H 1.091328 2.181395 2.181677
H 2.657467 1.616826 1.634718
H 1.627278 3.710054 -0.929266
38
CIS_EneBoryl_b
Eopt -684.033905
C -1.900337 -0.700195 0.001468
C -2.106753 -2.177340 0.172856
C -2.678076 -1.235058 1.184015
H -3.770276 -1.123276 1.245383
H -2.178972 -1.168935 2.152133
C -2.631224 -1.497526 -1.053276
H -3.719294 -1.408285 -1.184450
H -2.091073 -1.654727 -1.988505
H -1.374941 -2.969784 0.281435
O 0.328139 -0.099901 -1.198697
O 0.220775 0.165900 1.146028
C 1.565940 -0.535634 -0.655714
C 1.592611 0.118348 0.785330
C 2.697817 -0.078015 -1.588001
H 3.687669 -0.332400 -1.187152
H 2.597006 -0.573491 -2.562161
H 2.661053 1.001204 -1.761645
C 1.579054 -2.078378 -0.602453
H 1.328579 -2.466515 -1.597252
H 2.559271 -2.483045 -0.318822
H 0.834056 -2.457847 0.102330
C 2.347626 -0.697399 1.845031
H 3.405710 -0.827813 1.583578
H 2.305950 -0.176824 2.810288
H 1.901030 -1.686192 1.984785
C 2.170369 1.551010 0.777542
H 1.685466 2.173075 0.020180
H 1.982657 2.009368 1.756480
H 3.253372 1.568062 0.600312
B -0.601498 0.293875 -0.099462
C -1.148453 1.835086 -0.321050
C -0.983935 2.522990 -1.463088
H -0.443117 2.091625 -2.305317
C -1.896946 2.479701 0.827175
H -2.204816 3.515220 0.620035
H -2.800793 1.908711 1.085530
H -1.278088 2.482348 1.735602
H -1.370418 3.536593 -1.613113
37
CIS_Me2Boryl_d
Eopt -645.948498
C -1.859745 0.617477 -0.070664
C -2.809814 -0.517703 0.133677
C -2.848581 0.639429 1.082420
H -3.714795 1.318703 1.077300
H -2.426226 0.484173 2.077983
C -2.901825 0.288901 -1.125579
H -3.772034 0.942856 -1.290138
H -2.527571 -0.171100 -2.043382
O 0.464587 0.322410 -1.257174
O 0.440020 0.583214 1.088565
C 1.433487 -0.560524 -0.719003
C 1.683833 0.001322 0.741238
C 2.669334 -0.545685 -1.633250
H 3.483676 -1.165813 -1.235968
H 2.401365 -0.942876 -2.620833
H 3.047452 0.470346 -1.779646
C 0.865020 -1.994894 -0.706548
H 0.502646 -2.238972 -1.712807
H 1.614678 -2.746526 -0.426184
H 0.021720 -2.079620 -0.017411
C 2.030523 -1.067100 1.790466
H 2.178327 -0.589449 2.767546
H 1.223905 -1.797758 1.901093
H 2.954576 -1.604742 1.540859
C 2.792830 1.078183 0.780116
H 2.646444 1.840716 0.010154
H 2.761930 1.579012 1.755574
H 3.796350 0.652053 0.651433
B -0.271416 1.025850 -0.155464
C -0.204533 2.660284 -0.395206
H 0.824646 3.048799 -0.456809
H -0.704730 2.961273 -1.331519
H -0.703661 3.224506 0.410813
C -2.738047 -1.996379 0.375987
H -2.273483 -2.519090 -0.469229
H -2.152476 -2.225428 1.276261
H -3.737408 -2.435163 0.519179
34
CIS_MeBoryl_c
Eopt -606.632346
C -2.102918 0.146789 -0.030355
C -2.678160 -1.091816 0.587639
C -2.987981 0.240116 1.194048
H -4.016220 0.625399 1.128928
H -2.483721 0.502055 2.125920
C -3.024129 -0.748565 -0.827911
H -4.055838 -0.446743 -1.061710
H -2.551713 -1.330558 -1.621354
H -2.184354 -1.962377 1.005225
O 0.189766 -0.102812 -1.263842
O 0.184663 0.815425 0.912884
C 1.258243 -0.693761 -0.543535
C 1.481593 0.301550 0.666292
C 2.463514 -0.838191 -1.486204
H 2.218473 -1.538737 -2.294992
H 2.728973 0.117319 -1.948099
H 3.347432 -1.229305 -0.965537
C 0.846753 -2.106066 -0.074465
H 0.490685 -2.675599 -0.941664
H 1.680282 -2.662115 0.374380
H 0.032592 -2.061689 0.653563
C 2.000886 -0.357071 1.953662

H 1.298761 -1.106396 2.331107
H 2.976302 -0.838288 1.804775
H 2.122454 0.403951 2.735304
C 2.434621 1.463566 0.304914
H 3.482663 1.144414 0.234721
H 2.156257 1.931028 -0.643922
H 2.366401 2.229422 1.087291
B -0.620288 0.771618 -0.352302
C -0.830495 2.256243 -1.040623
H -1.377632 2.950490 -0.380654
H 0.118370 2.752377 -1.300475
H -1.415718 2.199590 -1.974417
47
CIS_NapBoryl_a
Eopt -952.030329
C 0.607886 2.041074 0.325628
C 1.584058 3.167794 0.155520
C 0.361409 3.116851 -0.706689
H -0.421685 3.874370 -0.560742
H 0.492647 2.822356 -1.748983
C 0.902942 2.976362 1.474790
H 0.166598 3.723728 1.803163
H 1.491186 2.554602 2.291339
H 2.635571 3.143532 -0.107363
O 1.725562 -0.165239 1.119839
O 1.063447 -0.020231 -1.135873
C 2.722351 -0.799139 0.329414
C 1.971988 -1.107756 -1.026257
C 3.229086 -2.038533 1.080737
H 3.960052 -2.604471 0.488828
H 3.722687 -1.730142 2.011118
H 2.407571 -2.708999 1.349075
C 3.905879 0.170849 0.134483
H 4.230912 0.532644 1.117438
H 4.766320 -0.306694 -0.351611
H 3.611798 1.038520 -0.462708
C 2.867124 -1.120355 -2.272103
H 3.644049 -1.892801 -2.207614
H 2.260041 -1.332958 -3.161095
H 3.352474 -0.152548 -2.427738
C 1.185081 -2.435007 -0.975462
H 0.561539 -2.494810 -0.078992
H 0.522290 -2.486154 -1.847669
H 1.843479 -3.312491 -0.998855
B 0.664695 0.408290 0.237491
C -0.817064 -0.164834 0.732394
C -0.963726 -0.624976 2.035377
C -2.012698 -0.128476 -0.073656
C -2.207226 -1.029071 2.581352
H -0.075926 -0.681216 2.661702
C -3.284426 -0.530914 0.470889
C -1.986118 0.293493 -1.437121
C -3.352095 -0.979158 1.818120
H -2.255346 -1.378091 3.611833
C -4.447843 -0.476520 -0.347897
C -3.130861 0.334902 -2.205435
H -1.025871 0.552909 -1.867912
H -4.314555 -1.281578 2.227032
C -4.378503 -0.049937 -1.655696
H -5.400455 -0.782309 0.081702
H -3.078210 0.658786 -3.242625
H -5.276177 -0.013770 -2.268843
47
CIS_NapBoryl_b
Eopt -952.029910
C 0.599029 1.894712 -0.790326
C 1.582942 3.020540 -0.909165
C 0.878794 2.529523 -2.134007
H 0.140118 3.183867 -2.618517
H 1.451750 1.922195 -2.836387
C 0.374901 3.187111 -0.040205
H -0.406320 3.896072 -0.349651
H 0.521290 3.147342 1.040394
H 2.639054 3.054563 -0.665958
O 1.013718 0.132857 1.104075
O 1.722180 -0.377339 -1.083852
C 2.295978 -0.472391 1.198308
C 2.452338 -1.208687 -0.192152
C 2.306499 -1.401374 2.420931
H 3.251525 -1.953083 2.506840
H 2.183885 -0.808757 3.336361
H 1.485716 -2.123152 2.382729
C 3.359646 0.623658 1.413862
H 3.066703 1.231777 2.278311
H 4.356277 0.210103 1.614540
H 3.429348 1.286747 0.547337
C 3.896443 -1.316242 -0.700976
H 4.528982 -1.893464 -0.014453
H 3.908795 -1.826184 -1.672454
H 4.347058 -0.329346 -0.840416
C 1.834338 -2.623504 -0.182189
H 0.808161 -2.612123 0.196537
H 1.806885 -3.005161 -1.209851
H 2.417255 -3.329498 0.422636
B 0.641840 0.322270 -0.331692
C -0.855470 -0.320344 -0.662205
C -1.042302 -1.010149 -1.853783
C -2.027295 -0.124061 0.155594
C -2.300650 -1.504204 -2.277668
H -0.175584 -1.177181 -2.489734
C -3.314740 -0.613215 -0.267787
C -1.963610 0.556944 1.408693
C -3.421906 -1.308013 -1.503221
H -2.379225 -2.038435 -3.223381
C -4.455544 -0.392218 0.554784
C -3.087603 0.757028 2.182717
H -0.992707 0.893908 1.754243
H -4.396013 -1.676772 -1.819608
C -4.350276 0.280758 1.751824
H -5.420276 -0.767209 0.216895
H -3.006868 1.279516 3.133457
H -5.231284 0.443533 2.368640

41
CIS_Ph2ClBoryl_a
Eopt -1257.981025

C	0.128864	1.942859	-0.649034
C	1.124451	3.020041	-0.969112
C	0.136765	2.601094	-2.010885
H	-0.653869	3.306497	-2.303898
H	0.503132	1.989248	-2.836501
C	0.155968	3.221523	0.153697
H	-0.633649	3.978282	0.042479
H	0.536002	3.136033	1.172648
H	2.207983	2.982697	-0.969845
O	0.856632	0.112983	1.077873
O	1.111102	-0.339503	-1.215401
C	2.102805	-0.547494	0.905461
C	1.958534	-1.236922	-0.508925
C	2.302942	-1.518592	2.078236
H	3.220808	-2.110469	1.967788
H	2.382633	-0.952870	3.015071
H	1.457240	-2.204620	2.179273
C	3.240232	0.494727	0.942872
H	3.143024	1.088626	1.859641
H	4.235286	0.031431	0.944807
H	3.182296	1.179342	0.092218
C	3.270257	-1.383940	-1.292023
H	3.994976	-2.011732	-0.757969
H	3.071245	-1.857931	-2.261486
H	3.732473	-0.412258	-1.488421
C	1.288364	-2.625082	-0.419963
H	0.357183	-2.585944	0.152619
H	1.044609	-2.968672	-1.432424
H	1.944603	-3.374643	0.039834
B	0.225639	0.362705	-0.241497
C	-1.326008	-0.263992	-0.317921
C	-2.353308	-0.172634	0.629312
C	-1.710654	-0.916154	-1.511532
C	-3.644257	-0.680220	0.450392
C	-2.986824	-1.433218	-1.737634
H	-0.949786	-1.020180	-2.281106
C	-3.965344	-1.317890	-0.747540
H	-4.385630	-0.575881	1.237240
H	-3.218027	-1.925722	-2.680128
H	-4.966278	-1.714903	-0.898900
Cl	-2.083121	0.642215	2.208374

41
CIS_Ph2ClBoryl_b
Eopt -1257.981681

C	-0.105047	1.991771	-0.466489
C	-1.072436	3.127479	-0.639962
C	-0.041595	3.195031	0.442278
H	0.772041	3.929152	0.354872
H	-0.374553	3.036056	1.468737
C	-0.156479	2.763651	-1.765305
H	0.645246	3.463132	-2.041933
H	-0.588348	2.234433	-2.616120
H	-2.155106	3.127381	-0.584972
O	-1.151226	-0.256203	-1.198851
O	-0.870957	0.099315	1.109535
C	-2.286488	-0.796035	-0.534627
C	-1.780542	-0.980950	0.949614
C	-2.698511	-2.093185	-1.244365
H	-3.534544	-2.589955	-0.735336
H	-3.020759	-1.867523	-2.268964
H	-1.864111	-2.797567	-1.308476
C	-3.454039	0.207609	-0.633161
H	-3.597581	0.480455	-1.685609
H	-4.398538	-0.208593	-0.259835
H	-3.237444	1.122674	-0.074856
C	-2.871166	-0.861658	2.022212
H	-3.648393	-1.626674	1.898660
H	-2.428016	-0.995608	3.017017
H	-3.347945	0.122579	2.003042
C	-1.036826	-2.318074	1.158808
H	-0.275905	-2.478896	0.390035
H	-0.528749	-2.289244	2.130087
H	-1.716828	-3.179413	1.157343
B	-0.241636	0.388093	-0.201546
C	1.280636	-0.284170	-0.391612
C	1.571951	-0.964491	-1.595293
C	2.366776	-0.205484	0.488750
C	2.820463	-1.513556	-1.892551
C	3.631697	-0.745895	0.238621
C	3.862005	-1.406505	-0.968136
H	2.980986	-2.025445	-2.839441
H	4.422570	-0.650635	0.977023
H	4.842123	-1.829123	-1.175991
Cl	2.205724	0.629953	2.071484
H	0.759799	-1.064970	-2.310915

41
CIS_PhBoryl_a
Eopt -798.383019

C	-0.064546	2.056758	-0.076420
C	0.923089	3.185161	-0.043535
C	0.031076	3.024325	-1.233976
H	-0.751895	3.774680	-1.414653
H	0.475141	2.627612	-2.148450
C	-0.136575	3.102474	1.011119
H	-0.933299	3.860077	1.021351
H	0.167698	2.777780	2.007765
H	2.003892	3.163138	0.039439
O	0.762004	-0.076440	1.172570
O	0.770201	-0.087266	-1.189586
C	1.945775	-0.733992	0.741881
C	1.616496	-1.141073	-0.750457
C	2.220314	-1.913912	1.685519
H	3.088245	-2.503363	1.362486
H	2.431393	-1.538451	2.695002
H	1.355463	-2.579955	1.755625
C	3.133750	0.247303	0.830360
H	3.165615	0.673375	1.840438
H	4.098440	-0.239924	0.638518
H	3.017833	1.072196	0.121644

C	2.834188	-1.209164	-1.682218
H	3.563638	-1.957173	-1.345858
H	2.511649	-1.491102	-2.692510
H	3.339100	-0.241621	-1.755097
C	0.861009	-2.484906	-0.840790
H	0.004360	-2.510990	-0.161561
H	0.480046	-2.607442	-1.861903
H	1.506603	-3.343375	-0.615961
B	0.004885	0.424024	-0.015173
C	-1.545997	-0.142064	0.030693
C	-2.198833	-0.444745	1.241332
C	-2.313401	-0.294999	-1.140904
C	-3.531636	-0.867829	1.288747
H	-1.637549	-0.356583	2.170619
C	-3.645881	-0.718902	-1.114448
H	-1.845994	-0.083853	-2.102401
C	-4.265205	-1.005792	0.106418
H	-3.999186	-1.093636	2.246366
H	-4.203028	-0.828658	-2.043994
H	-5.301965	-1.335052	0.135214

43
CIS_tBuBoryl_a
Eopt -724.565231

C	-1.455500	1.245133	-0.036518
C	-1.201279	2.723057	-0.134992
C	-1.997096	2.048369	-1.202836
H	-3.069390	2.278076	-1.290236
H	-1.509593	1.873325	-2.163595
C	-1.949811	2.185897	1.043021
H	-3.017835	2.429540	1.145356
H	-1.425044	2.132630	1.998565
H	-0.271264	3.277931	-0.193436
O	0.365332	-0.182861	1.173853
O	0.359906	-0.174193	-1.178071
C	1.694049	0.123095	0.793847
C	1.722133	-0.101025	-0.792657
C	2.637104	-0.804799	1.582613
H	3.692603	-0.629615	1.337946
H	2.509190	-0.618052	2.656300
H	2.412658	-1.860119	1.404581
C	2.025483	1.570248	1.222312
H	1.851876	1.662991	2.301512
H	3.071668	1.838903	1.027018
H	1.386365	2.300364	0.721834
C	2.392614	1.042935	-1.579250
H	3.454993	1.157122	-1.326951
H	2.327871	0.827222	-2.653213
H	1.893093	2.000584	-1.407057
C	2.419263	-1.412322	-1.216661
H	2.000276	-2.282004	-0.704549
H	2.266675	-1.558006	-2.293272
H	3.500906	-1.392695	-1.031147
B	-0.557442	-0.142578	0.002390
C	-1.577953	-1.474594	0.052169
C	-2.536594	-1.500437	-1.154501
C	-0.775043	-2.787587	0.036850
C	-2.417008	-1.456483	1.345561
H	-3.212191	-0.635573	-1.160383
H	-1.983018	-1.486400	-2.103788
H	-3.171048	-2.404250	-1.161774
H	-0.056869	-2.823775	0.865717
H	-1.428754	-3.672751	0.128424
H	-0.209195	-2.898140	-0.896135
H	-3.068920	-2.343757	1.431654
H	-1.772898	-1.438260	2.234732
H	-3.069024	-0.574901	1.399946

41
TRANS_2MeThiofuranBoryl_b
Eopt -1158.465338

C	-0.538001	2.122254	-0.354283
C	-0.193522	3.358926	0.430298
C	0.430564	3.145197	-0.909386
H	0.134551	3.799715	-1.740342
H	1.481506	2.854693	-0.937606
C	-1.614602	3.113642	0.035536
H	-2.074015	3.772308	-0.714657
H	-2.304889	2.782122	0.811177
H	0.260919	3.432564	1.411920
O	-0.866537	-0.350671	-1.139555
O	-1.287835	0.196505	1.116091
C	-2.053092	-1.022918	-0.742445
C	-1.958303	-1.025096	0.835103
C	-2.070219	-2.416256	-1.386025
H	-1.164575	-2.980006	-1.144777
H	-2.940858	-3.003069	-1.065335
H	-2.118683	-2.319014	-2.477965
C	-3.271107	-0.228830	-1.259029
H	-3.165444	-0.088099	-2.341495
H	-4.222609	-0.744497	-1.076481
H	-3.320580	0.762380	-0.798335
C	-3.310401	-1.015621	1.560186
H	-3.906049	-1.906436	1.322745
H	-3.149031	-1.003433	2.645554
H	-3.896807	-0.128634	1.303489
C	-1.118702	-2.201933	1.375364
H	-0.157629	-2.273986	0.858471
H	-0.912749	-2.027889	2.438531
H	-1.636649	-3.165116	1.284299
B	-0.394681	0.530154	-0.027084
C	1.191568	0.175946	0.271698
C	2.010190	0.481337	1.334391
C	3.364031	0.020778	1.222038
H	1.648733	1.029012	2.202224
C	3.618008	-0.652385	0.056360
H	4.123692	0.182104	1.984252
C	4.905344	-1.272317	-0.402834
H	5.686696	-1.116305	0.349662
H	4.811349	-2.354790	-0.564489
H	5.261964	-0.838395	-1.346896
S	2.151188	-0.723689	-0.905165

41
TRANS_2MeThiofuranBoryl_c

Eopt -1158.465204

C	0.354085	2.088749	0.259413
C	-0.350520	3.145037	-0.547184
C	-0.589802	3.066974	0.925078
H	-0.201490	3.870119	1.566270
H	-1.543985	2.662223	1.265087
C	1.144154	3.123922	-0.513565
H	1.671553	3.936428	0.005652
H	1.664543	2.757738	-1.398755
H	-1.035994	3.014120	-1.376744
O	1.224722	-0.161564	1.251585
O	1.020588	0.039477	-1.089715
C	2.385919	-0.729000	0.663168
C	1.936366	-1.015006	-0.824453
C	2.783363	-1.975936	1.465544
H	1.951659	-2.682002	1.545034
H	3.639523	-2.495770	1.016322
H	3.069070	-1.684658	2.484325
C	3.539602	0.293441	0.744625
H	3.651744	0.612798	1.787756
H	4.499286	-0.123757	0.413366
H	3.325348	1.183175	0.145387
C	3.065756	-0.951331	-1.861310
H	3.842028	-1.702070	-1.664653
H	2.660613	-1.146280	-2.862298
H	3.536549	0.035777	-1.881949
C	1.211864	-2.369939	-0.972080
H	0.413404	-2.475160	-0.232033
H	0.752856	-2.416767	-1.966978
H	1.893094	-3.224612	-0.873626
B	0.379225	0.458659	0.187597
C	-1.153595	-0.130796	0.368118
C	-1.741876	-0.737479	1.452762
C	-3.114398	-1.116761	1.278654
H	-1.185922	-0.919785	2.368759
C	-3.617604	-0.805985	0.043533
H	-3.708686	-1.609870	2.045159
S	-2.364154	-0.029783	-0.911945
C	-4.992945	-1.059385	-0.500202
H	-5.610803	-1.552846	0.258562
H	-5.502655	-0.131022	-0.791652
H	-4.976098	-1.706880	-1.387430

41

TRANS_2MeThiofuranBoryl_d

Eopt -1158.464085

C	-0.422879	2.111013	-0.389791
C	0.051888	3.295805	0.407908
C	0.615061	3.059881	-0.953775
H	0.351585	3.756017	-1.761745
H	1.634323	2.679000	-1.022899
C	-1.395596	3.184420	0.054344
H	-1.818749	3.897057	-0.667512
H	-2.087162	2.900430	0.846996
H	0.540115	3.297822	1.376142
O	-0.968075	-0.268110	-1.208097
O	-1.283366	0.174418	1.085871
C	-1.803194	-1.292931	-0.686544
C	-2.357615	-0.648567	0.645749
C	-0.971854	-2.566178	-0.420935
H	-1.597624	-3.416215	-0.120252
H	-0.446468	-2.843251	-1.342582
H	-0.219756	-2.398578	0.354457
C	-2.872610	-1.625703	-1.735878
H	-2.395244	-2.058720	-2.624067
H	-3.600567	-2.356224	-1.360147
H	-3.413211	-0.730263	-2.055834
C	-3.598822	0.232046	0.392729
H	-4.490859	-0.359110	0.149246
H	-3.816557	0.809477	1.299332
H	-3.420128	0.938983	-0.422762
C	-2.692497	-1.652537	1.757595
H	-3.476903	-2.356378	1.450451
H	-1.811098	-2.225882	2.058766
H	-3.056490	-1.115558	2.642849
B	-0.401508	0.509368	-0.069658
C	1.142989	0.041747	0.293193
C	1.766430	-0.069600	1.514280
C	3.138737	-0.484580	1.470781
H	1.237853	0.132542	2.442925
C	3.606019	-0.703726	0.202161
H	3.758509	-0.621822	2.354574
S	2.316355	-0.393033	-0.949645
C	4.971719	-1.151530	-0.229027
H	5.459737	-0.421734	-0.889249
H	5.615244	-1.287907	0.647516
H	4.944173	-2.105802	-0.772530

41

TRANS_4ClPhBoryl_a

Eopt -1257.985007

C	-0.936806	2.107547	-0.351534
C	-0.370167	3.291448	0.384918
C	-0.082182	3.132078	-1.070651
H	-0.536628	3.837283	-1.779814
H	0.920807	2.813946	-1.358475
C	-1.852035	3.108274	0.323291
H	-2.447527	3.818394	-0.267577
H	-2.355276	2.758918	1.224488
H	0.302225	3.298990	1.235528
O	-1.447020	-0.247923	-1.243872
O	-1.464095	0.026256	1.099210
C	-2.165392	-1.339758	-0.687947
C	-2.562267	-0.805091	0.745086
C	-1.253718	-2.583946	-0.617078
H	-1.796174	-3.482036	-0.295440
H	-0.841445	-2.778439	-1.614551
H	-0.413798	-2.426807	0.065715
C	-3.350834	-1.659293	-1.609051
H	-2.979914	-2.016348	-2.578185
H	-3.994530	-2.443505	-1.190181
H	-3.962166	-0.771929	-1.796764
C	-3.852312	0.040768	0.713242
H	-4.749571	-0.568821	0.546296

H -3.967489 0.549311 1.678034
H -3.805276 0.805600 -0.067629
C -2.719580 -1.891798 1.817647
H -3.513418 -2.605721 1.562491
H -1.788853 -2.447069 1.965208
H -2.984703 -1.429458 2.776982
B -0.770371 0.494383 -0.138337
C 0.838417 0.138345 -0.050770
C 1.603133 -0.140256 -1.198968
C 1.535333 0.127500 1.172453
C 2.975909 -0.406903 -1.150451
H 1.106041 -0.164680 -2.167078
C 2.905945 -0.136218 1.261986
H 0.985060 0.316023 2.092500
C 3.611071 -0.398274 0.089095
H 3.538246 -0.620844 -2.055164
H 3.414708 -0.141976 2.221985
Cl 5.356669 -0.735643 0.177824
41
TRANS_4ClPhBoryl_b
Eopt -1257.985617
C -0.937633 2.104491 -0.205030
C -0.427251 3.243598 0.636930
C -0.078656 3.193408 -0.813930
H -0.520166 3.939107 -1.489096
H 0.941672 2.916028 -1.082737
C -1.901696 3.034583 0.501110
H -2.488786 3.773808 -0.061805
H -2.433946 2.607097 1.351074
H 0.210566 3.202880 1.512756
O -1.398475 -0.255613 -1.234124
O -1.463383 -0.009083 1.112229
C -2.538347 -0.951847 -0.754194
C -2.206645 -1.173171 0.774714
C -2.703723 -2.242064 -1.569660
H -1.788920 -2.841637 -1.559049
H -3.529678 -2.860374 -1.194871
H -2.923553 -1.992792 -2.615531
C -3.793265 -0.077501 -0.964480
H -3.844837 0.215168 -2.020153
H -4.722627 -0.605336 -0.714747
H -3.745732 0.838063 -0.367449
C -3.430990 -1.253651 1.695732
H -4.082668 -2.097591 1.435439
H -3.104520 -1.393517 2.734115
H -4.022486 -0.334298 1.656425
C -1.333281 -2.424189 1.009335
H -0.468434 -2.439088 0.339466
H -0.957480 -2.404544 2.039531
H -1.892467 -3.358253 0.871656
B -0.752550 0.482055 -0.103458
C 0.857449 0.131475 -0.074858
C 1.589276 -0.147370 -1.244069
C 1.587449 0.122712 1.128587
C 2.963384 -0.410997 -1.233233
H 1.065482 -0.173723 -2.197857
C 2.960339 -0.139218 1.180497
H 1.061653 0.311928 2.062765
C 3.632685 -0.400591 -0.011582
H 3.501312 -0.623669 -2.152985
H 3.495839 -0.143753 2.125829
Cl 5.380754 -0.734483 0.028498
47
TRANS_CyBoryl_a
Eopt -801.990868
C 0.385326 2.150845 0.189372
C -0.043486 3.331356 -0.640600
C -0.510132 3.230631 0.771929
H -0.118361 3.945757 1.509264
H -1.552834 2.962591 0.946040
C 1.411271 3.108489 -0.386294
H 1.949138 3.821668 0.255696
H 2.013801 2.727216 -1.209917
H -0.602731 3.346302 -1.570162
O 0.762037 -0.177905 1.218064
O 0.964804 -0.021628 -1.118801
C 1.680989 -1.176510 0.822616
C 2.084104 -0.758601 -0.655058
C 0.999022 -2.561273 0.893754
H 1.695731 -3.386265 0.695572
H 0.592029 -2.701153 1.902661
H 0.168933 -2.640401 0.186740
C 2.848538 -1.186050 1.825432
H 2.473528 -1.463966 2.818636
H 3.626121 -1.910563 1.549916
H 3.310474 -0.198723 1.913939
C 3.347083 0.129547 -0.695053
H 4.258706 -0.429952 -0.448427
H 3.464666 0.531173 -1.709003
H 3.268090 0.975110 -0.006950
C 2.314983 -1.938937 -1.612977
H 3.138670 -2.584158 -1.280863
H 1.416135 -2.552953 -1.718600
H 2.573189 -1.558422 -2.609520
B 0.168285 0.522258 0.025733
C -1.474289 0.232169 -0.172924
C -1.845110 -0.867222 -1.192270
C -2.210709 -0.047543 1.156076
H -1.925547 1.158313 -0.580132
C -3.364688 -1.018542 -1.385699
H -1.444660 -1.837530 -0.859507
H -1.361233 -0.662078 -2.156054
C -3.732807 -0.203378 0.988760
H -1.803774 -0.966740 1.603105
H -2.002238 0.748948 1.884911
C -4.079106 -1.280290 -0.050763
H -3.589530 -1.827746 -2.097126
H -3.766203 -0.094391 -1.831047
H -4.208790 -0.443321 1.951691
H -4.161263 0.757932 0.663168
H -5.167120 -1.336336 -0.199584
H -3.767017 -2.263482 0.334980

47
TRANS_CyBoryl_c
Eopt -801.992977
C 0.434342 2.059740 -0.242007
C -0.180882 3.318297 0.310239
C 1.238742 2.991342 0.643370
H 2.035487 3.626022 0.227767
H 1.456377 2.619518 1.645210
C -0.079482 3.145812 -1.169397
H 0.620761 3.784027 -1.727684
H -0.993977 2.926347 -1.723298
H -1.046076 3.452643 0.950148
O 0.713467 -0.099937 1.112494
O 0.879153 -0.307577 -1.225775
C 1.726593 -1.055803 0.845336
C 2.080331 -0.825682 -0.684857
C 1.164006 -2.466777 1.123275
H 1.929657 -3.250433 1.053216
H 0.754210 -2.486384 2.140593
H 0.353305 -2.718750 0.433926
C 2.898723 -0.821914 1.813589
H 2.558802 -0.977238 2.845590
H 3.729269 -1.516390 1.630443
H 3.283206 0.199526 1.743837
C 3.225086 0.192263 -0.890660
H 4.200939 -0.202847 -0.579865
H 3.286653 0.438312 -1.957984
H 3.040087 1.122017 -0.347636
C 2.446955 -2.104476 -1.454485
H 3.339327 -2.591908 -1.040710
H 1.624987 -2.826134 -1.450193
H 2.661216 -1.854493 -2.501394
B 0.128690 0.436549 -0.163089
C -1.509688 0.165476 -0.311183
C -1.896896 -1.189150 -0.941434
C -2.266782 0.325894 1.026157
H -1.920101 0.936422 -0.993570
C -3.416244 -1.369768 -1.104144
H -1.521045 -2.007546 -0.307202
H -1.393853 -1.306699 -1.909290
C -3.789885 0.146142 0.894557
H -1.877549 -0.415288 1.740670
H -2.054717 1.307244 1.473154
C -4.147903 -1.194366 0.235397
H -3.648687 -2.356387 -1.533060
H -3.795570 -0.623568 -1.820372
H -4.277283 0.223230 1.878275
H -4.198659 0.965251 0.281873
H -5.235472 -1.276851 0.095987
H -3.857797 -2.014464 0.910734
47
TRANS_CyBoryl_d
Eopt -801.993930
C 0.005501 1.958115 -0.256044
C -0.506849 3.086472 0.598323
C 0.940646 3.046587 0.241688
H 1.335285 3.811154 -0.443162
H 1.644284 2.745261 1.017254
C -1.052226 2.914824 -0.780637
H -0.798451 3.665404 -1.543094
H -2.065426 2.526496 -0.883937
H -0.998445 3.055873 1.564205
O 0.987625 0.072262 1.128334
O 0.907580 -0.309591 -1.198355
C 2.041614 -0.818793 0.806344
C 2.208699 -0.653156 -0.760750
C 1.637862 -2.254517 1.212563
H 2.464498 -2.970043 1.112719
H 1.324003 -2.245358 2.263533
H 0.796267 -2.620606 0.617922
C 3.281785 -0.429826 1.626794
H 3.070617 -0.555287 2.696563
H 4.148494 -1.058602 1.384024
H 3.559318 0.616004 1.466158
C 3.196987 0.471844 -1.143757
H 4.242226 0.202082 -0.943431
H 3.100100 0.669091 -2.218592
H 2.975539 1.402184 -0.614945
C 2.647330 -1.931319 -1.494348
H 3.627470 -2.287987 -1.151564
H 1.920492 -2.738495 -1.364403
H 2.725733 -1.729384 -2.570380
B 0.125986 0.324404 -0.078502
C -1.332637 -0.465328 0.028580
C -2.265184 0.004914 1.164549
C -2.129336 -0.568021 -1.291266
H -1.055205 -1.506109 0.282491
C -3.527909 -0.864465 1.305830
H -2.582059 1.044484 0.984832
H -1.713301 0.015660 2.115644
C -3.392618 -1.438504 -1.164139
H -2.433860 0.434787 -1.629955
H -1.476161 -0.967655 -2.078614
C -4.298580 -0.954467 -0.020778
H -4.185497 -0.474304 2.097283
H -3.234264 -1.879026 1.618110
H -3.955467 -1.452099 -2.109734
H -3.094240 -2.480060 -0.965647
H -5.170888 -1.615320 0.085320
H -4.693139 0.042302 -0.273636
38
TRANS_EneBoryl_a
Eopt -684.033110
C -1.816466 -0.843845 -0.411078
C -2.825149 -1.513418 0.484600
C -3.304956 -0.615444 -0.604163
H -3.833951 -1.056398 -1.460386
H -3.687456 0.363836 -0.314428
C -1.900377 -2.355149 -0.331722
H -2.317005 -2.941064 -1.162927
H -1.084956 -2.854683 0.189730
H -2.922941 -1.421940 1.560642

O 0.376755 0.278509 -1.193404
O 0.258939 -0.410290 1.055377
C 1.702812 0.276524 -0.691791
C 1.593539 -0.611708 0.615807
C 2.151141 1.725256 -0.391701
H 3.199655 1.782721 -0.072774
H 2.047680 2.322549 -1.305926
H 1.529803 2.185649 0.381929
C 2.630755 -0.292157 -1.776099
H 2.630284 0.375006 -2.647436
H 3.667048 -0.379059 -1.424633
H 2.296759 -1.276821 -2.115663
C 1.799255 -2.113533 0.326220
H 2.841496 -2.360575 0.086110
H 1.515353 -2.686302 1.217697
H 1.166659 -2.446906 -0.501796
C 2.551252 -0.206115 1.747491
H 3.603948 -0.296203 1.448809
H 2.371095 0.821727 2.075073
H 2.395299 -0.860946 2.614308
B -0.577837 0.163610 -0.043072
C -1.201609 1.635716 0.375570
C -1.364652 2.030817 1.649845
H -1.052591 1.398469 2.481325
H -1.797955 2.997815 1.926120
C -1.616890 2.572265 -0.741253
H -2.050520 3.515899 -0.377943
H -0.755064 2.815590 -1.377214
H -2.350943 2.102578 -1.411407
38
TRANS_EneBoryl_b
Eopt -684.033890
C -1.728997 -0.896266 -0.381202
C -3.049234 -1.240473 0.252543
C -3.020384 -0.774663 -1.165470
H -3.261515 -1.490968 -1.963256
H -3.359529 0.241382 -1.374135
C -1.992838 -2.292367 0.144888
H -2.155110 -3.136088 -0.541217
H -1.458388 -2.567328 1.054631
H -3.529257 -0.812932 1.125704
O 0.422151 0.209116 -1.215475
O 0.293196 -0.192785 1.106234
C 1.743781 0.216798 -0.700790
C 1.593360 -0.558092 0.668085
C 2.219953 1.672108 -0.498756
H 3.273064 1.730155 -0.194924
H 2.111998 2.214478 -1.445803
H 1.613367 2.189484 0.249702
C 2.669106 -0.456720 -1.724421
H 2.705545 0.144655 -2.641776
H 3.696129 -0.551422 -1.348414
H 2.306772 -1.452048 -1.997680
C 1.659928 -2.088673 0.480387
H 2.672352 -2.440246 0.243282
H 1.346068 -2.574526 1.412227
H 0.985114 -2.418278 -0.315164
C 2.604891 -0.157161 1.751441
H 3.638806 -0.355236 1.440091
H 2.517771 0.902824 2.007814
H 2.416992 -0.734832 2.665492
B -0.545601 0.197194 -0.073800
C -1.236681 1.667799 0.204641
C -1.377869 2.601021 -0.751919
H -1.001762 2.436447 -1.761627
H -1.856666 3.570186 -0.577042
C -1.752805 1.960771 1.598553
H -2.184012 2.967941 1.698373
H -2.521989 1.237174 1.904486
H -0.943781 1.858072 2.334289
38
TRANS_EneBoryl_c
Eopt -684.034416
C -1.720778 -0.961788 -0.208383
C -2.924423 -1.305088 0.626288
C -3.105979 -0.981153 -0.821141
H -3.403919 -1.784899 -1.508832
H -3.532414 -0.010450 -1.079489
C -1.828841 -2.310232 0.473550
H -2.026944 -3.223098 -0.105923
H -1.162045 -2.465429 1.322179
H -3.309048 -0.815102 1.513705
O 0.439160 0.038522 -1.248863
O 0.251591 -0.013505 1.104166
C 1.683037 -0.390888 -0.721585
C 1.629901 0.095714 0.780560
C 2.813342 0.228312 -1.556927
H 2.722521 1.317160 -1.608456
H 3.804118 -0.019117 -1.153758
H 2.767876 -0.156238 -2.583915
C 1.778295 -1.927888 -0.839243
H 1.590075 -2.211616 -1.881805
H 2.765915 -2.314950 -0.556734
H 1.024046 -2.420703 -0.218842
C 2.434467 -0.765470 1.763811
H 3.504042 -0.775429 1.516958
H 2.332134 -0.362811 2.779668
H 2.073394 -1.797969 1.780237
C 2.076672 1.565979 0.939313
H 1.549893 2.218025 0.236313
H 1.832392 1.900689 1.955104
H 3.156471 1.697604 0.793370
B -0.557471 0.188876 -0.141208
C -1.250836 1.685674 -0.195476
C -1.223109 2.470801 -1.285772
H -0.712025 2.154621 -2.194769
H -1.693191 3.459029 -1.322721
C -1.959316 2.187450 1.047077
H -2.322213 3.221296 0.949344
H -2.823253 1.556967 1.301985
H -1.291059 2.139367 1.917559
38

TRANS_EneBoryl_d
Eopt -684.034476

C	-1.728437	-0.962633	-0.231151
C	-2.760751	-1.458765	0.747641
C	-3.208317	-0.886606	-0.555096
H	-3.653997	-1.556825	-1.303087
H	-3.660105	0.105904	-0.537826
C	-1.739646	-2.410238	0.217282
H	-2.066057	-3.210036	-0.462336
H	-0.932418	-2.705620	0.887413
H	-2.938542	-1.113498	1.759997
O	0.395047	0.256051	-1.206753
O	0.278818	-0.186313	1.107916
C	1.658714	-0.237434	-0.797608
C	1.645890	-0.013320	0.767897
C	2.753974	0.536949	-1.546110
H	2.646791	1.616730	-1.406882
H	3.760272	0.242911	-1.220275
H	2.680775	0.333737	-2.622201
C	1.772958	-1.728494	-1.183241
H	1.566595	-1.830001	-2.255774
H	2.772318	-2.140290	-0.991721
H	1.040379	-2.337274	-0.645814
C	2.480603	-1.022693	1.568515
H	3.544041	-0.977411	1.299944
H	2.399077	-0.803123	2.640698
H	2.128975	-2.046986	1.413852
C	2.095296	1.410589	1.162157
H	1.548042	2.171992	0.598906
H	1.877887	1.566096	2.225857
H	3.170446	1.570050	1.010302
B	-0.574573	0.188674	-0.059285
C	-1.309007	1.657031	0.079869
C	-1.589327	2.228638	1.262760
H	-1.312914	1.750103	2.202660
H	-2.089991	3.197965	1.355739
C	-1.684647	2.383081	-1.195592
H	-2.172881	3.352405	-1.015344
H	-0.791296	2.555450	-1.810559
H	-2.359273	1.779830	-1.820362

38
TRANS_FuranBoryl_a
Eopt -796.162320

C	0.774358	1.861940	-0.320455
C	1.706161	2.707740	0.506842
C	2.072610	2.379601	-0.902942
H	2.065282	3.184892	-1.650283
H	2.855397	1.639383	-1.069079
C	0.312097	3.184521	0.251902
H	0.164780	4.060032	-0.396018
H	-0.398900	3.125509	1.075763
H	2.196414	2.441722	1.436724
O	-0.650191	-0.061245	-1.249382
O	-0.692801	0.254727	1.087423
C	-1.779657	-0.758466	-0.745340
C	-2.008843	-0.082808	0.664943
C	-1.454681	-2.264026	-0.634025
H	-2.332514	-2.863915	-0.361707
H	-1.092610	-2.617137	-1.607253
H	-0.667131	-2.450202	0.101379
C	-2.936910	-0.585799	-1.738663
H	-2.685689	-1.078124	-2.686678
H	-3.866177	-1.035882	-1.366191
H	-3.124879	0.469818	-1.954942
C	-2.849919	1.206656	0.564465
H	-3.905648	1.005017	0.342080
H	-2.804959	1.734947	1.524464
H	-2.456767	1.875637	-0.206808
C	-2.643610	-0.997206	1.722012
H	-3.642054	-1.339530	1.420377
H	-2.020979	-1.874921	1.917755
H	-2.751526	-0.450714	2.667610
B	0.206655	0.348372	-0.100679
C	1.469174	-0.673162	0.150116
C	1.969905	-1.299369	1.266266
O	2.304219	-1.000246	-0.911034
C	3.142631	-2.037752	0.884196
H	1.535189	-1.242097	2.256331
C	3.295986	-1.819548	-0.450189
H	3.779183	-2.648096	1.513374
H	4.014678	-2.158027	-1.184334

38
TRANS_FuranBoryl_b
Eopt -796.161916

C	0.757321	1.810706	-0.426222
C	1.823658	2.629433	0.252567
C	1.970066	2.292731	-1.194121
H	1.877962	3.097742	-1.936003
H	2.701193	1.530767	-1.468494
C	0.421344	3.145225	0.204269
H	0.204179	4.025465	-0.417184
H	-0.164065	3.104483	1.122954
H	2.435549	2.344420	1.100416
O	-0.708444	-0.145642	-1.238295
O	-0.637880	0.235075	1.089880
C	-1.851764	-0.763023	-0.662395
C	-1.987832	-0.023787	0.726118
C	-1.600868	-2.276900	-0.494547
H	-2.492481	-2.811354	-0.142748
H	-1.317616	-2.700092	-1.465934
H	-0.782046	-2.469232	0.204535
C	-3.037863	-0.569665	-1.617178
H	-2.849262	-1.108705	-2.554244
H	-3.972519	-0.958321	-1.192604
H	-3.182664	0.485296	-1.867320
C	-2.749638	1.312072	0.599558
H	-3.823204	1.168031	0.422417
H	-2.636525	1.875523	1.533393
H	-2.344551	1.921619	-0.213764
C	-2.641584	-0.854524	1.838947
H	-3.668420	-1.145806	1.582400
H	-2.067048	-1.760693	2.051496

H -2.684591 -0.265490 2.763950
B 0.201522 0.301590 -0.142637
C 1.482222 -0.719829 -0.028976
C 2.031398 -1.655212 -0.872771
O 2.286526 -0.669683 1.103187
C 3.200967 -2.204380 -0.241766
H 1.634651 -1.925771 -1.843020
C 3.304539 -1.567804 0.956527
H 3.868167 -2.966647 -0.625609
H 4.002273 -1.637430 1.780307
38
TRANS_FuranBoryl_d
Eopt -796.163426
C 0.734090 1.836487 -0.285123
C 1.653670 2.689895 0.546677
C 1.997083 2.420587 -0.881685
H 1.943784 3.247797 -1.602700
H 2.806552 1.718467 -1.085727
C 0.237681 3.122426 0.340565
H 0.041934 4.011259 -0.275462
H -0.452719 3.006051 1.176178
H 2.177346 2.416140 1.454895
O -0.686673 -0.157355 -1.223210
O -0.620626 0.197788 1.108282
C -2.010214 -0.286562 -0.726864
C -1.794886 -0.547573 0.816541
C -2.706928 -1.428239 -1.480442
H -2.133451 -2.357796 -1.418549
H -3.716995 -1.616849 -1.094082
H -2.800736 -1.167355 -2.542278
C -2.780803 1.022397 -1.003828
H -2.705444 1.253351 -2.073400
H -3.845533 0.949637 -0.747540
H -2.349705 1.862636 -0.451510
C -2.930689 -0.042290 1.716994
H -3.882813 -0.538731 1.489043
H -2.691072 -0.250000 2.767486
H -3.072051 1.037928 1.617957
C -1.545064 -2.037282 1.137316
H -0.763487 -2.457480 0.497837
H -1.204151 -2.119951 2.176626
H -2.449504 -2.649463 1.029392
B 0.217188 0.299072 -0.123251
C 1.506568 -0.714769 -0.133517
C 1.876939 -1.765803 -0.937096
O 2.508798 -0.553586 0.816256
C 3.137172 -2.273398 -0.467609
H 1.303013 -2.135438 -1.777108
C 3.470758 -1.498170 0.600091
H 3.713733 -3.098168 -0.868773
H 4.311096 -1.488407 1.281211
37
TRANS_Me2Boryl_a
Eopt -645.950516
C 1.693147 -0.014641 0.609706
C 3.087043 -0.346581 0.162724
C 2.900014 0.612136 1.292852
H 3.184878 0.312899 2.312217
H 3.084622 1.669109 1.089624
C 2.217440 -1.437768 0.699643
H 2.459288 -1.894996 1.670869
H 1.804062 -2.145056 -0.020982
O -0.686483 0.915277 0.928899
O -0.296115 -0.317696 -1.044991
C -1.935074 0.430200 0.472299
C -1.532224 -0.749266 -0.501187
C -2.700298 1.559336 -0.256109
H -3.731956 1.277385 -0.504412
H -2.739269 2.436794 0.401006
H -2.197064 1.857178 -1.180352
C -2.768457 0.003224 1.690733
H -2.998866 0.881702 2.307304
H -3.722042 -0.455288 1.397601
H -2.222016 -0.705329 2.319826
C -1.334818 -2.084361 0.250722
H -2.282372 -2.514559 0.600275
H -0.868804 -2.808545 -0.428776
H -0.673815 -1.961390 1.113757
C -2.514765 -0.994134 -1.657044
H -3.513832 -1.271385 -1.295870
H -2.610883 -0.111175 -2.295886
H -2.148908 -1.816779 -2.285030
B 0.371656 0.648015 -0.107923
C 0.812740 2.013566 -0.923515
H 1.316698 2.756248 -0.283174
H 1.507021 1.799272 -1.753365
H -0.049319 2.532739 -1.371143
C 3.809148 -0.213373 -1.145601
H 3.883517 0.837976 -1.452632
H 4.834356 -0.611491 -1.100786
H 3.283840 -0.756062 -1.943058
37
TRANS_Me2Boryl_b
Eopt -645.951632
C -1.693068 -0.539960 -0.338215
C -3.008059 -0.305239 0.349241
C -3.005143 -0.729564 -1.083372
H -3.357102 -1.736262 -1.352547
H -3.259093 0.026394 -1.829450
C -2.099466 -1.374136 0.864594
H -2.387056 -2.430005 0.752219
H -1.562074 -1.164806 1.790748
O 0.730966 -0.316960 -1.332000
O 0.232645 0.763019 0.706769
C 1.769847 -0.622602 -0.421188
C 1.624775 0.500310 0.683466
C 3.107310 -0.604785 -1.178593
H 3.255879 0.341088 -1.707822
H 3.961961 -0.765251 -0.508187
H 3.118759 -1.407071 -1.927570
C 1.561976 -2.046079 0.144075
H 1.469735 -2.744844 -0.696625

H	2.399885	-2.380453	0.769868
H	0.643139	-2.114891	0.732861
C	2.071463	0.078515	2.091695
H	3.134902	-0.193049	2.121002
H	1.922341	0.910248	2.792389
H	1.488429	-0.770827	2.460107
C	2.379282	1.795753	0.307658
H	2.149892	2.109580	-0.715027
H	2.057481	2.597351	0.983562
H	3.468626	1.694239	0.398571
B	-0.380013	0.405842	-0.618597
C	-0.817243	1.721981	-1.510680
H	0.038282	2.388250	-1.704723
H	-1.221429	1.449588	-2.499863
H	-1.588060	2.340390	-1.021334
C	-3.602752	0.892751	1.028846
H	-3.736528	1.722969	0.322870
H	-4.588617	0.680069	1.469708
H	-2.951690	1.247936	1.838929

34
TRANS_MeBoryl_a
Eopt -606.632253

C	2.036057	-0.091504	0.293506
C	3.328352	-0.436755	-0.398153
C	3.352416	0.499676	0.763650
H	3.814492	0.163049	1.702666
H	3.512100	1.559820	0.561429
C	2.525421	-1.524610	0.238256
H	2.928694	-2.023447	1.131608
H	1.968450	-2.192620	-0.418912
H	3.608321	-0.282131	-1.434600
O	-0.274927	0.853364	0.931691
O	-0.131410	-0.284074	-1.129815
C	-1.579247	0.421596	0.592150
C	-1.319137	-0.709715	-0.482847
C	-2.390571	1.606214	0.017757
H	-3.449813	1.358595	-0.130842
H	-2.335973	2.444673	0.723047
H	-1.984533	1.948402	-0.938127
C	-2.284104	-0.055881	1.871803
H	-2.414842	0.790578	2.558362
H	-3.279031	-0.470966	1.663712
H	-1.694490	-0.814658	2.394478
C	-1.091564	-2.091792	0.168110
H	-2.013684	-2.516743	0.585691
H	-0.719033	-2.785768	-0.595265
H	-0.346288	-2.039332	0.967205
C	-2.423269	-0.858272	-1.541515
H	-3.390516	-1.122323	-1.094149
H	-2.549040	0.061542	-2.120197
H	-2.154894	-1.656135	-2.246050
B	0.653665	0.625298	-0.229218
C	1.030232	2.028954	-1.012205
H	1.541070	2.756991	-0.360955
H	1.695433	1.868891	-1.877739
H	0.138428	2.543306	-1.403344

34
TRANS_MeBoryl_b
Eopt -606.633548

C	-2.021180	-0.223781	-0.171397
C	-3.227966	-0.263432	0.730263
C	-3.409708	0.044394	-0.719254
H	-3.914662	-0.696090	-1.355722
H	-3.635479	1.074949	-0.997303
C	-2.391100	-1.490971	0.572812
H	-2.819265	-2.355566	0.045406
H	-1.733037	-1.763816	1.398055
H	-3.435186	0.338207	1.608693
O	0.293447	0.367067	-1.264377
O	0.121750	0.340584	1.089649
C	1.392545	-0.370054	-0.763159
C	1.473430	0.084684	0.749672
C	2.634857	-0.028159	-1.601069
H	2.810745	1.051134	-1.634156
H	3.539257	-0.515835	-1.214143
H	2.489739	-0.370111	-2.633891
C	1.116328	-1.883123	-0.915469
H	0.855175	-2.087440	-1.961204
H	1.987378	-2.500303	-0.658786
H	0.274885	-2.204111	-0.295261
C	2.032294	-0.975090	1.711152
H	3.062415	-1.257208	1.456903
H	2.040102	-0.579407	2.735015
H	1.417214	-1.879803	1.712733
C	2.296383	1.380221	0.930866
H	1.989137	2.152120	0.219252
H	2.121054	1.768411	1.941730
H	3.375688	1.217028	0.814011
B	-0.658212	0.692881	-0.144977
C	-1.059628	2.289155	-0.235978
H	-0.175624	2.941523	-0.157809
H	-1.545230	2.547842	-1.191462
H	-1.752349	2.609799	0.560005

47
TRANS_NapBoryl_a
Eopt -952.030885

C	-0.675350	1.991372	-0.721407
C	-0.342918	3.331686	-0.120499
C	0.436100	2.847297	-1.296920
H	0.302313	3.353558	-2.262768
H	1.457604	2.508145	-1.125127
C	-1.712114	3.095911	-0.667346
H	-2.015646	3.629931	-1.578923
H	-2.522245	2.950102	0.046166
H	-0.018367	3.550645	0.890541
O	-1.099107	-0.523613	-1.097071
O	-1.798397	0.388593	0.954935
C	-2.013032	-1.440958	-0.513791
C	-2.786022	-0.540535	0.527997
C	-1.238168	-2.591530	0.163938
H	-1.903997	-3.377451	0.542294
H	-0.564071	-3.046529	-0.571486

H	-0.628291	-2.226860	0.995290
C	-2.884649	-2.033980	-1.628628
H	-2.262063	-2.633689	-2.304568
H	-3.667679	-2.690591	-1.228107
H	-3.360542	-1.250818	-2.225811
C	-3.957634	0.225009	-0.121938
H	-4.797524	-0.433352	-0.378206
H	-4.325846	0.977855	0.585417
H	-3.636798	0.744052	-1.030296
C	-3.318239	-1.290586	1.757467
H	-4.042202	-2.067956	1.480693
H	-2.507523	-1.758153	2.323596
H	-3.827259	-0.586815	2.428276
B	-0.711821	0.491311	-0.067734
C	0.754760	0.192942	0.654687
C	0.886229	0.430911	2.018237
C	1.947556	-0.224440	-0.041121
C	2.111529	0.298217	2.716269
H	-0.001125	0.726050	2.574416
C	3.201966	-0.357243	0.656356
C	1.937884	-0.525397	-1.437402
C	3.254381	-0.083739	2.049733
H	2.147662	0.502501	3.785310
C	4.364840	-0.760041	-0.059942
C	3.081762	-0.913558	-2.102801
H	0.989322	-0.467825	-1.959372
H	4.203796	-0.183603	2.572802
C	4.311924	-1.029691	-1.409351
H	5.303789	-0.851563	0.483627
H	3.041935	-1.138122	-3.166462
H	5.209064	-1.335773	-1.942767

47

TRANS_NapBoryl_b

Eopt -952.030922

C	-0.636694	1.934262	-0.884369
C	0.313037	3.070171	-0.622571
C	0.099291	2.607603	-2.025676
H	-0.436588	3.265349	-2.723898
H	0.895033	2.030023	-2.497871
C	-1.123614	3.212760	-0.234072
H	-1.752527	3.925239	-0.786691
H	-1.370516	3.144443	0.825509
H	1.210103	3.080974	-0.014336
O	-1.785144	-0.334776	-1.165488
O	-1.137592	0.238250	1.022471
C	-2.536755	-1.140270	-0.269317
C	-2.434114	-0.341866	1.088872
C	-1.906790	-2.546534	-0.169941
H	-2.510814	-3.231504	0.438480
H	-1.828024	-2.973028	-1.177069
H	-0.899827	-2.505778	0.255050
C	-3.960058	-1.285805	-0.824525
H	-3.932208	-1.840752	-1.770764
H	-4.612903	-1.836589	-0.135206
H	-4.413012	-0.311023	-1.026852
C	-3.491149	0.777110	1.190395
H	-4.503277	0.383852	1.349865
H	-3.244528	1.422947	2.041653
H	-3.500185	1.396554	0.288704
C	-2.517288	-1.206506	2.354426
H	-3.475004	-1.738472	2.422736
H	-1.708690	-1.942152	2.391052
H	-2.426752	-0.570520	3.244214
B	-0.707684	0.369811	-0.405049
C	0.777646	-0.323560	-0.674489
C	0.990012	-0.997746	-1.871422
C	1.913934	-0.208459	0.207110
C	2.241081	-1.550413	-2.242111
H	0.147818	-1.111700	-2.550681
C	3.192587	-0.762711	-0.159614
C	1.822604	0.448537	1.471978
C	3.327507	-1.435224	-1.404512
H	2.340795	-2.067779	-3.195086
C	4.298289	-0.627162	0.727232
C	2.913008	0.564710	2.308766
H	0.855190	0.834302	1.773091
H	4.295332	-1.851893	-1.677779
C	4.167651	0.024333	1.933485
H	5.256730	-1.050308	0.430965
H	2.811151	1.070153	3.266671
H	5.021898	0.121435	2.599749

47

TRANS_NapBoryl_c

Eopt -952.031561

C	-0.684465	2.014539	-0.517922
C	-0.419065	3.316845	0.190883
C	0.410781	2.947692	-0.993325
H	0.288064	3.521234	-1.922312
H	1.437926	2.629166	-0.814760
C	-1.762525	3.074960	-0.415139
H	-2.058548	3.665771	-1.293413
H	-2.587118	2.845334	0.259438
H	-0.132301	3.469629	1.225331
O	-1.034388	-0.531736	-1.047530
O	-1.770684	0.314583	1.021845
C	-2.340705	-1.035764	-0.815859
C	-2.511538	-0.866650	0.744563
C	-2.400842	-2.485671	-1.317339
H	-1.610980	-3.097484	-0.872279
H	-3.369194	-2.953725	-1.097922
H	-2.261261	-2.506065	-2.405638
C	-3.361658	-0.202036	-1.619256
H	-3.050811	-0.186391	-2.670987
H	-4.375736	-0.619343	-1.573130
H	-3.398264	0.832891	-1.267812
C	-3.957407	-0.659558	1.214802
H	-4.592519	-1.520216	0.968620
H	-3.977851	-0.532616	2.304628
H	-4.401378	0.235557	0.769513
C	-1.906239	-2.049751	1.530277
H	-0.885161	-2.266448	1.202587
H	-1.868298	-1.787383	2.594403

H -2.502135 -2.965830 1.430858
B -0.678013 0.465197 0.015133
C 0.811311 0.131112 0.664859
C 0.983943 0.273215 2.036613
C 1.980133 -0.240069 -0.094363
C 2.225357 0.073228 2.688911
H 0.116860 0.539274 2.637312
C 3.250077 -0.442056 0.556559
C 1.932022 -0.421700 -1.510155
C 3.343009 -0.276515 1.964884
H 2.293057 0.197374 3.768643
C 4.388343 -0.801736 -0.219601
C 3.053253 -0.768414 -2.234302
H 0.974083 -0.302121 -2.004197
H 4.303608 -0.431142 2.453046
C 4.297671 -0.960359 -1.584471
H 5.339026 -0.949293 0.290220
H 2.984567 -0.900402 -3.311902
H 5.176269 -1.234410 -2.164139
47
TRANS_NapBoryl_d
Eopt -952.031596
C -0.664693 2.041872 -0.471622
C 0.196745 3.137716 0.093542
C 0.115038 2.963453 -1.387323
H -0.403137 3.728643 -1.981708
H 0.975514 2.527718 -1.896596
C -1.270901 3.138513 0.379575
H -1.895161 3.923417 -0.070383
H -1.594773 2.838713 1.376595
H 1.045074 3.059629 0.763760
O -1.744233 -0.213122 -1.195187
O -1.151376 0.066078 1.066191
C -2.818036 -0.692157 -0.401577
C -2.139660 -0.953591 0.999779
C -3.406423 -1.940666 -1.073556
H -2.636991 -2.695048 -1.261635
H -4.199569 -2.396215 -0.466565
H -3.844852 -1.666939 -2.041599
C -3.917570 0.388597 -0.330518
H -4.189463 0.681349 -1.351864
H -4.826378 0.034406 0.172861
H -3.561026 1.282462 0.189181
C -3.073271 -0.807860 2.208346
H -3.900524 -1.528292 2.172461
H -2.512664 -0.991699 3.133661
H -3.494440 0.199763 2.271664
C -1.453591 -2.333897 1.071299
H -0.809699 -2.503486 0.203069
H -0.822248 -2.369375 1.967272
H -2.174711 -3.158703 1.132932
B -0.703479 0.411172 -0.317171
C 0.778817 -0.205323 -0.743021
C 0.958953 -0.675475 -2.038890
C 1.940195 -0.222552 0.112786
C 2.204939 -1.133405 -2.533930
H 0.095715 -0.698472 -2.700329
C 3.214479 -0.680469 -0.380410
C 1.876737 0.195164 1.477179
C 3.318488 -1.130873 -1.724318
H 2.279830 -1.485603 -3.561627
C 4.345522 -0.676787 0.484532
C 2.990696 0.185833 2.290619
H 0.910649 0.492500 1.869238
H 4.283219 -1.474117 -2.093641
C 4.242262 -0.250110 1.789840
H 5.300478 -1.022349 0.091997
H 2.909690 0.507449 3.326678
H 5.115385 -0.253165 2.438407
41
TRANS_PhBoryl_a
Eopt -798.383887
C 0.183067 2.036856 -0.358288
C 0.989980 3.072825 0.377494
C 1.277901 2.812250 -1.063543
H 1.025890 3.586608 -1.801123
H 2.185510 2.259123 -1.308586
C -0.490461 3.243032 0.263000
H -0.880602 4.058607 -0.362422
H -1.091784 3.048152 1.150829
H 1.617412 2.943200 1.252449
O -0.849416 -0.139331 -1.248184
O -0.874100 0.166135 1.091249
C -1.813334 -1.030103 -0.707407
C -2.121447 -0.396129 0.706642
C -1.212309 -2.448263 -0.594038
H -1.957278 -3.195314 -0.291515
H -0.816545 -2.743573 -1.573326
H -0.385670 -2.476972 0.121288
C -3.011640 -1.086970 -1.664675
H -2.702154 -1.534806 -2.617629
H -3.829877 -1.697171 -1.260854
H -3.398447 -0.086832 -1.880779
C -3.180413 0.723224 0.622424
H -4.188590 0.334910 0.428770
H -3.205764 1.260715 1.578076
H -2.931349 1.443087 -0.162934
C -2.559106 -1.400275 1.782396
H -3.488783 -1.915251 1.507334
H -1.787079 -2.153274 1.965324
H -2.738790 -0.874259 2.728676
B -0.044732 0.432832 -0.124609
C 1.427515 -0.295510 0.021036
C 2.161656 -0.726210 -1.101695
C 2.041801 -0.490500 1.273912
C 3.430135 -1.306624 -0.990262
H 1.716401 -0.616321 -2.089883
C 3.307902 -1.070586 1.405708
H 1.500240 -0.192052 2.170648
C 4.012389 -1.480142 0.269044
H 3.963563 -1.628894 -1.883556
H 3.745890 -1.207550 2.393566

H	4.997760	-1.931964	0.363692

41
TRANS_PhBoryl_b
Eopt -798.384657

C	0.141466	2.035314	-0.235467
C	0.858251	3.048867	0.617031
C	1.238026	2.901653	-0.819475
H	0.994789	3.715550	-1.516256
H	2.181478	2.403574	-1.047266
C	-0.619953	3.164412	0.427710
H	-1.011224	4.005669	-0.161764
H	-1.261843	2.875353	1.260055
H	1.441097	2.882491	1.516082
O	-0.820583	-0.188174	-1.230999
O	-0.847570	0.119564	1.109554
C	-2.090852	-0.598090	-0.750830
C	-1.826303	-0.857723	0.784830
C	-2.540754	-1.834027	-1.542677
H	-1.787065	-2.626238	-1.510587
H	-3.488085	-2.239834	-1.164518
H	-2.692119	-1.564168	-2.595660
C	-3.115343	0.532675	-0.989482
H	-3.084789	0.816145	-2.048610
H	-4.142894	0.229556	-0.750339
H	-2.872968	1.421501	-0.399783
C	-3.045680	-0.651380	1.693098
H	-3.864721	-1.335942	1.437379
H	-2.767365	-0.842071	2.737376
H	-3.421316	0.374332	1.633445
C	-1.248682	-2.264744	1.048935
H	-0.402473	-2.475667	0.388585
H	-0.885153	-2.308494	2.082703
H	-1.995707	-3.058121	0.919183
B	-0.031743	0.412413	-0.106953
C	1.459603	-0.284712	-0.060504
C	2.094518	-0.776129	-1.218022
C	2.186262	-0.401189	1.141083
C	3.374345	-1.341110	-1.187618
H	1.559922	-0.725988	-2.165619
C	3.465221	-0.965553	1.191947
H	1.726186	-0.053640	2.065351
C	4.068889	-1.437511	0.022031
H	3.829634	-1.711311	-2.105265
H	3.991020	-1.042490	2.142708
H	5.063441	-1.877906	0.053579

43
TRANS_tBuBoryl_c
Eopt -724.562814

C	1.291719	-1.285113	-0.050488
C	2.658715	-1.852995	-0.317521
C	2.006130	-2.116500	0.999853
H	1.674453	-3.141001	1.227106
H	2.377468	-1.583445	1.876077
C	1.515148	-2.270003	-1.183604
H	1.154700	-3.308041	-1.115593
H	1.454753	-1.859673	-2.192852
H	3.598954	-1.371712	-0.563505
O	-0.446231	0.187728	1.188072
O	-0.461657	0.236144	-1.157876
C	-1.714998	-0.300624	0.792252
C	-1.812693	0.115940	-0.741015
C	-2.779613	0.336590	1.699290
H	-3.796490	0.038170	1.413580
H	-2.617425	0.013011	2.735429
H	-2.723111	1.428758	1.683732
C	-1.776779	-1.831129	1.004899
H	-1.520496	-2.049936	2.048788
H	-2.777008	-2.239637	0.810938
H	-1.060907	-2.357970	0.370058
C	-2.519349	-0.917664	-1.637124
H	-3.565500	-1.075293	-1.342647
H	-2.517783	-0.562039	-2.675543
H	-2.007489	-1.883809	-1.620466
C	-2.520941	1.471949	-0.952055
H	-3.600948	1.416591	-0.762956
H	-2.101989	2.252964	-0.312395
H	-2.378424	1.783938	-1.994145
B	0.475103	0.167761	0.012335
C	1.498781	1.490217	0.061662
C	2.498333	1.382543	1.230910
C	2.283931	1.614312	-1.259149
C	0.710136	2.799798	0.253435
H	1.977583	1.282564	2.193236
H	3.162771	0.516072	1.126942
H	3.145958	2.273926	1.304485
H	1.598888	1.700772	-2.112881
H	2.939992	2.502329	-1.272062
H	2.923609	0.741792	-1.442628
H	1.379946	3.674511	0.326032
H	0.030476	2.983655	-0.587451
H	0.104490	2.769806	1.167405

48
INT-tropylium-trans_a
Eopt -877.201674

C	-0.087537	-0.846616	0.141919
C	1.700152	-0.570583	-0.078461
C	0.924729	-1.601185	0.859723
H	1.180050	-2.601481	0.502476
H	0.962126	-1.500726	1.941890
C	0.533937	-0.715159	-1.162480
H	0.757827	-1.615904	-1.738345
H	0.223763	0.134213	-1.766659
H	1.787063	0.428557	0.343129
O	-2.328446	-0.801307	1.035539
O	-1.709874	1.077534	-0.236332
C	-3.306178	-0.615548	0.017489
C	-3.108725	0.887850	-0.384147
C	-4.681054	-0.939753	0.596523
H	-5.475893	-0.695679	-0.119037
H	-4.746887	-2.010780	0.821953
H	-4.864635	-0.389006	1.523088
C	-3.023086	-1.575512	-1.146397

H -2.942835 -2.595427 -0.752894
H -3.826427 -1.560263 -1.892274
H -2.085085 -1.330650 -1.655132
C -3.509909 1.210561 -1.821010
H -4.565690 0.972024 -1.999827
H -3.368775 2.280649 -2.013855
H -2.900691 0.656731 -2.541334
C -3.847345 1.842575 0.565775
H -3.639573 1.603860 1.613557
H -3.500718 2.864816 0.376105
H -4.932935 1.815833 0.413049
B -1.215233 0.158999 0.801805
C -0.677585 0.872614 2.156299
H 0.203628 1.508551 1.978904
H -1.459972 1.523022 2.572544
H -0.405730 0.153357 2.942963
C 3.073006 -1.096806 -0.573308
C 3.631010 -0.228533 -1.671949
C 4.060249 -1.196494 0.561741
H 2.903986 -2.099027 -0.974338
C 4.026802 1.052711 -1.521859
H 3.670451 -0.669251 -2.666241
C 4.559416 -0.148334 1.249542
H 4.349104 -2.200598 0.866420
C 4.151754 1.789014 -0.281266
H 4.302057 1.600279 -2.422261
C 4.387902 1.257204 0.946262
H 5.175177 -0.369122 2.120411
H 4.163748 2.873743 -0.368422
H 4.574367 1.949436 1.765086
48
INT-tropylium-trans_b
Eopt -877.199320
C -0.011354 -0.029054 -0.026107
C -1.630331 0.370438 0.782921
C -1.285822 0.067827 -0.729275
H -1.773283 -0.875396 -0.984873
H -1.397861 0.835103 -1.492219
C -0.473001 -0.644789 1.204444
H -0.914479 -1.644033 1.142188
H 0.123552 -0.481990 2.100036
H -1.412147 1.400199 1.067419
O 2.018581 -0.002794 -1.285725
O 2.205489 0.792189 0.920803
C 3.073808 -0.715090 -0.646528
C 3.452951 0.224267 0.551319
C 4.195757 -0.932869 -1.658793
H 5.077634 -1.373380 -1.177554
H 3.860250 -1.620770 -2.443908
H 4.491226 0.005033 -2.136795
C 2.559689 -2.083562 -0.179537
H 2.088783 -2.593820 -1.027647
H 3.372555 -2.718612 0.191735
H 1.815623 -1.986967 0.617433
C 4.048463 -0.503561 1.753445
H 4.961212 -1.044845 1.475314
H 4.310492 0.221577 2.532987
H 3.338042 -1.215984 2.182937
C 4.410561 1.347445 0.125657
H 4.056275 1.855673 -0.776634
H 4.464817 2.087537 0.932134
H 5.423759 0.973089 -0.063434
B 1.346682 0.859801 -0.271709
C 1.012329 2.354827 -0.804194
H 0.423444 2.938436 -0.079494
H 1.943591 2.912243 -0.978959
H 0.453004 2.355789 -1.751456
C -3.045517 -0.000743 1.336350
C -4.033215 1.099481 1.037398
C -3.566228 -1.356949 0.948640
H -2.894647 -0.010316 2.420300
C -4.670827 1.321827 -0.128494
H -4.201678 1.806524 1.848099
C -4.125388 -1.708418 -0.229556
H -3.460550 -2.135959 1.701530
C -4.685604 0.481382 -1.309258
H -5.272457 2.227283 -0.196804
C -4.459143 -0.854172 -1.350987
H -4.373504 -2.761157 -0.359541
H -5.014242 0.961978 -2.228775
H -4.618154 -1.361111 -2.300774
48
INT-tropylium-trans_c
Eopt -877.201756
C -0.239754 0.441287 0.381212
C 1.486138 0.346314 -0.251386
C 0.833257 1.308389 0.840601
H 1.320742 1.075214 1.789884
H 0.737887 2.375743 0.653498
C 0.479905 -0.808094 0.202566
H 0.932219 -1.286205 1.074135
H 0.067917 -1.508113 -0.521156
H 1.329775 0.682377 -1.276849
O -2.489952 0.958486 0.866261
O -2.171662 -0.221102 -1.143762
C -3.351025 -0.178440 0.880367
C -3.455582 -0.557766 -0.638823
C -4.675567 0.226386 1.523169
H -5.411096 -0.583802 1.446658
H -4.521622 0.443573 2.586721
H -5.092345 1.121461 1.053650
C -2.717118 -1.292337 1.723630
H -2.450174 -0.885593 2.705768
H -3.409968 -2.127776 1.877771
H -1.809006 -1.688282 1.259151
C -3.722542 -2.038087 -0.897025
H -4.660668 -2.358021 -0.427038
H -3.807811 -2.216102 -1.975501
H -2.910867 -2.664931 -0.516499
C -4.518490 0.273547 -1.372942
H -4.408017 1.342153 -1.164212
H -4.396751 0.125022 -2.451858

H	-5.536233	-0.028501	-1.098621
B	-1.635183	0.895843	-0.355390
C	-1.501934	2.307641	-1.140031
H	-0.760402	2.269236	-1.953386
H	-2.462148	2.580447	-1.599909
H	-1.215137	3.138737	-0.478922
C	2.959963	0.020054	0.009605
C	3.843035	1.222166	-0.200515
C	3.461468	-1.066268	-0.905624
H	3.068320	-0.318953	1.049969
C	5.133239	1.217899	0.191875
H	3.412521	2.110800	-0.659492
C	4.653220	-1.661428	-0.695337
H	2.830489	-1.380443	-1.735618
C	5.850279	0.054959	0.675126
H	5.702474	2.143553	0.121929
C	5.635913	-1.230976	0.278933
H	4.925858	-2.515182	-1.313860
H	6.719154	0.246909	1.302424
H	6.347278	-1.983093	0.615911

48

PR-tropylium-Cis_a

Eopt -877.308731

C	0.199102	1.828218	-0.124163
C	-1.690298	0.783123	-0.039068
C	-0.874267	1.607351	0.981125
H	-1.384715	2.550165	1.223807
H	-0.569705	1.119109	1.912384
C	-0.897008	1.474347	-1.169922
H	-1.414627	2.380014	-1.516612
H	-0.610697	0.873890	-2.039290
H	-1.406980	-0.273951	0.023435
O	1.924686	0.185551	-1.174657
O	1.929120	0.324194	1.105342
C	2.805992	-0.870463	-0.717475
C	3.117012	-0.428351	0.754719
C	4.019507	-0.936923	-1.631327
H	4.744528	-1.664779	-1.249806
H	3.711731	-1.257202	-2.632428
H	4.513907	0.033748	-1.721584
C	2.012506	-2.174507	-0.801072
H	1.670063	-2.317190	-1.831271
H	2.629871	-3.034825	-0.522329
H	1.130965	-2.153132	-0.151055
C	3.287389	-1.573911	1.740048
H	4.126462	-2.212450	1.441374
H	3.502320	-1.175392	2.737283
H	2.386312	-2.189038	1.806137
C	4.299010	0.535332	0.857830
H	4.197757	1.373543	0.159678
H	4.338751	0.942842	1.873355
H	5.246915	0.026002	0.655424
B	1.353375	0.756847	-0.064230
C	0.808061	3.230094	-0.216405
H	0.017202	3.991602	-0.252472
H	1.444044	3.450138	0.651045
H	1.421452	3.343319	-1.119880
C	-3.215687	0.915530	-0.031104
C	-3.860824	0.234143	-1.215826
C	-3.834587	0.390120	1.243627
H	-3.440518	1.986688	-0.096523
C	-3.871599	-1.097850	-1.433353
H	-4.312158	0.878126	-1.969415
C	-3.838398	-0.903644	1.628040
H	-4.270331	1.124716	1.919484
C	-3.439822	-2.134389	-0.515588
H	-4.267612	-1.450337	-2.385413
C	-3.425060	-2.048398	0.839489
H	-4.213161	-1.132432	2.625410
H	-3.210576	-3.101931	-0.959488
H	-3.184681	-2.952365	1.396976

48

PR-tropylium-Cis_b

Eopt -877.309091

C	0.295085	1.347983	-0.311157
C	-1.339599	-0.046687	0.076613
C	-0.616658	1.022841	0.923075
H	-1.265265	1.876546	1.157273
H	-0.138892	0.689424	1.851824
C	-0.750181	0.606290	-1.196296
H	-1.446173	1.317075	-1.659439
H	-0.374064	-0.068781	-1.971680
H	-0.866791	-1.023225	0.233384
O	1.851363	-0.725986	-0.577727
O	2.771579	1.105838	0.429567
C	3.101168	-1.180267	-0.002014
C	3.861735	0.168867	0.243913
C	3.782191	-2.127251	-0.977347
H	4.771747	-2.413497	-0.603411
H	3.184313	-3.038377	-1.086429
H	3.899570	-1.676368	-1.966050
C	2.749440	-1.919203	1.289170
H	2.063181	-2.739314	1.053902
H	3.641538	-2.343335	1.761576
H	2.254643	-1.259455	2.010268
C	4.745713	0.183455	1.481275
H	5.524647	-0.583429	1.402416
H	5.236984	1.157945	1.573373
H	4.170771	0.004477	2.393531
C	4.654156	0.645496	-0.973172
H	4.039758	0.644605	-1.880070
H	4.996002	1.670516	-0.796091
H	5.533149	0.016244	-1.147672
B	1.656908	0.571992	-0.171036
C	0.497019	2.831998	-0.604741
H	-0.468762	3.353365	-0.654965
H	1.099661	3.315371	0.173609
H	1.008496	2.985106	-1.564204
C	-2.848560	-0.227131	0.223868
C	-3.400030	-1.207938	-0.780159
C	-3.658692	1.033726	0.065393
H	-3.048318	-0.625597	1.232489

C -4.635619 -1.739256 -0.667087
H -2.756732 -1.510249 -1.606633
C -4.964759 1.092759 0.402098
H -3.167385 1.931945 -0.305929
C -5.636148 -1.313195 0.290353
H -4.931395 -2.529555 -1.356148
C -5.783080 -0.045454 0.768327
H -5.467639 2.058872 0.376043
H -6.412489 -2.035472 0.538760
H -6.667727 0.164177 1.368057
48
PR-tropylium-Cis_c
Eopt -877.305661
C 0.016920 -1.354382 -0.210471
C 1.454511 0.153726 -0.837482
C 1.103272 -0.491804 0.520167
H 1.905333 -1.142470 0.883058
H 0.793385 0.172258 1.335249
C 0.773047 -1.044098 -1.534332
H 1.494782 -1.841020 -1.756757
H 0.184566 -0.834085 -2.433953
H 0.836954 1.050031 -0.979101
O -1.857957 0.280331 -1.003026
O -2.247051 -0.845135 0.945977
C -3.007450 0.923750 -0.399517
C -3.481354 -0.151574 0.638504
C -4.021238 1.251558 -1.484181
H -4.937820 1.655649 -1.039586
H -3.609079 2.009072 -2.159362
H -4.279955 0.370857 -2.077585
C -2.500288 2.210104 0.252628
H -2.013580 2.827318 -0.509650
H -3.322508 2.787434 0.688048
H -1.766705 2.002063 1.039231
C -4.064063 0.418587 1.922297
H -4.947748 1.028236 1.702095
H -4.371492 -0.398477 2.583659
H -3.338511 1.035719 2.458393
C -4.436222 -1.185973 0.042694
H -4.040777 -1.612758 -0.885471
H -4.570370 -2.001386 0.761028
H -5.417944 -0.748074 -0.165938
B -1.382360 -0.643251 -0.103292
C -0.045246 -2.821474 0.208003
H 0.952511 -3.278969 0.162644
H -0.418854 -2.929501 1.233472
H -0.707985 -3.399593 -0.449700
C 2.894497 0.530926 -1.234801
C 3.350629 1.810152 -0.569429
C 3.913485 -0.574105 -1.075967
H 2.836865 0.744024 -2.310047
C 3.801020 1.958306 0.691868
H 3.252274 2.713240 -1.171641
C 4.524719 -0.971490 0.058494
H 4.158384 -1.123008 -1.984697
C 4.113895 0.917101 1.655486
H 3.983925 2.975966 1.036580
C 4.442250 -0.366235 1.376023
H 5.173625 -1.844572 -0.008857
H 4.179395 1.236937 2.694176
H 4.748879 -0.998651 2.207655
48
PR-tropylium-Cis_d
Eopt -877.310189
C -0.313739 -1.306415 -0.623059
C 1.394100 -0.104097 -0.047499
C 0.703620 -1.349152 0.554079
H 1.354498 -2.231321 0.479115
H 0.316931 -1.281104 1.576066
C 0.716074 -0.433860 -1.396621
H 1.371104 -1.057471 -2.020749
H 0.338745 0.397912 -2.000227
H 0.960154 0.811741 0.379577
O -2.295462 0.302654 -1.132330
O -2.278601 -0.641558 0.947413
C -3.356639 0.958140 -0.393526
C -3.583875 -0.027188 0.805001
C -4.559662 1.138373 -1.305885
H -5.407090 1.546803 -0.743777
H -4.313581 1.842393 -2.107905
H -4.866782 0.194701 -1.763936
C -2.817176 2.321193 0.039873
H -2.506043 2.879464 -0.849196
H -3.583227 2.904945 0.560798
H -1.948012 2.220625 0.699118
C -3.963211 0.642842 2.116112
H -4.904903 1.192297 2.005242
H -4.102895 -0.116709 2.892637
H -3.191034 1.338332 2.454888
C -4.569411 -1.150580 0.483496
H -4.316553 -1.652301 -0.457063
H -4.533571 -1.895429 1.285202
H -5.594591 -0.772781 0.411710
B -1.634468 -0.526879 -0.260270
C -0.679541 -2.652070 -1.255520
H 0.227691 -3.217058 -1.508943
H -1.276810 -3.268589 -0.571078
H -1.258050 -2.518131 -2.178852
C 2.910631 -0.020314 0.001454
C 3.441552 0.113871 1.405761
C 3.454544 1.148595 -0.779363
H 3.325228 -0.945444 -0.433970
C 4.751830 -0.028765 1.696534
H 2.729126 0.306127 2.208052
C 4.768400 1.277618 -1.060324
H 2.749038 1.894253 -1.146053
C 5.812075 -0.142621 0.714989
H 5.059139 -0.035491 2.741708
C 5.819546 0.441217 -0.516319
H 5.085964 2.080979 -1.724167
H 6.731299 -0.623442 1.046581
H 6.744352 0.389610 -1.089192

48
PR-tropylium-Cis_e
Eopt -877.308663

C	-0.270082	1.300604	-0.179708
C	1.392165	-0.052865	0.143412
C	0.753644	0.588969	-1.112432
H	1.422374	1.316172	-1.590544
H	0.364830	-0.084936	-1.882736
C	0.703291	1.029439	1.003915
H	1.360503	1.891164	1.178070
H	0.275776	0.715506	1.961920
H	0.916209	-1.024713	0.330531
O	-2.357211	0.435354	1.110286
O	-2.258054	-0.097309	-1.108894
C	-3.452610	-0.484993	0.877200
C	-3.602411	-0.434031	-0.683196
C	-4.673933	-0.010190	1.648595
H	-5.540979	-0.637808	1.413400
H	-4.483475	-0.083409	2.724611
H	-4.921838	1.028723	1.416391
C	-3.000712	-1.849889	1.396410
H	-2.735194	-1.757799	2.454667
H	-3.798494	-2.594474	1.306562
H	-2.120700	-2.215625	0.856129
C	-4.011989	-1.749431	-1.326516
H	-4.987189	-2.073243	-0.945598
H	-4.097108	-1.620285	-2.410748
H	-3.281862	-2.539732	-1.134063
C	-4.514485	0.693977	-1.165155
H	-4.238392	1.655365	-0.718192
H	-4.421784	0.786387	-2.252241
H	-5.563052	0.487726	-0.926742
B	-1.634411	0.522928	-0.053967
C	-0.558079	2.771722	-0.494782
H	0.380101	3.334894	-0.592772
H	-1.111841	2.880044	-1.436579
H	-1.149404	3.245068	0.299755
C	2.903551	-0.248309	0.232798
C	3.718703	1.003705	0.034422
C	3.406843	-1.240907	-0.784941
H	3.138970	-0.641805	1.235604
C	5.037108	1.051925	0.320954
H	3.222218	1.904257	-0.323995
C	4.640593	-1.783838	-0.714954
H	2.730328	-1.541770	-1.584996
C	5.857587	-0.091881	0.664125
H	5.547935	2.012796	0.269136
C	5.680597	-1.361292	0.201015
H	4.902557	-2.581237	-1.409502
H	6.766199	0.112946	1.228670
H	6.458809	-2.089512	0.424999

48
PR-tropylium-Trans_a
Eopt -877.307163

C	0.091911	-0.399481	0.893492
C	-1.964284	-0.108907	0.225181
C	-0.875256	0.822486	0.804449
H	-0.542324	1.569080	0.072510
H	-1.098312	1.337897	1.748901
C	-0.875142	-1.169238	-0.055602
H	-0.545230	-1.146691	-1.103491
H	-1.102668	-2.207535	0.213785
H	-2.644341	-0.448543	1.013572
O	2.340258	0.889579	0.783729
O	2.225649	-1.031420	-0.449262
C	3.546186	0.864633	-0.021432
C	3.620159	-0.637392	-0.462556
C	4.718474	1.340521	0.824560
H	5.653852	1.243587	0.266667
H	4.583246	2.396557	1.082519
H	4.802343	0.771273	1.754250
C	3.320611	1.825529	-1.189799
H	3.091016	2.821655	-0.793667
H	4.211901	1.905245	-1.820579
H	2.477065	1.507769	-1.815925
C	4.189248	-0.866091	-1.852851
H	5.219387	-0.489988	-1.912192
H	4.207161	-1.941571	-2.074881
H	3.593652	-0.370398	-2.622799
C	4.339354	-1.527243	0.552839
H	3.945848	-1.384701	1.566701
H	4.190902	-2.575412	0.275434
H	5.416743	-1.328584	0.566577
B	1.562976	-0.174862	0.396673
C	0.108864	-1.010841	2.305401
H	-0.910522	-1.175520	2.678753
H	0.625918	-1.978359	2.311562
H	0.619068	-0.348521	3.016789
C	-2.772953	0.410318	-0.967196
C	-3.640069	-0.660103	-1.589527
C	-3.633054	1.601995	-0.610314
H	-2.050035	0.734904	-1.724084
C	-4.702227	-1.242394	-0.992369
H	-3.353908	-1.014969	-2.578143
C	-4.690844	1.571158	0.225261
H	-3.341519	2.564812	-1.029640
C	-5.324802	-0.849778	0.255810
H	-5.167749	-2.085167	-1.505578
C	-5.322353	0.396800	0.794670
H	-5.152914	2.525536	0.491050
H	-5.940458	-1.604771	0.741553
H	-5.933184	0.559622	1.681162

48
PR-tropylium-Trans_b
Eopt -877.303989

C	0.031187	1.557504	-0.272442
C	2.142397	1.425661	0.259736
C	0.850373	1.769358	1.037329
H	0.613981	1.018432	1.800238
H	0.795689	2.762978	1.497814
C	1.271538	0.821240	-0.866262
H	1.179766	-0.264323	-0.784512

H	1.548027	1.072174	-1.896741
H	2.619805	2.347254	-0.096797
O	-2.156485	0.794625	0.906759
O	-1.679055	-0.219332	-1.084808
C	-3.101698	-0.296875	0.782973
C	-3.028433	-0.627030	-0.748381
C	-4.465494	0.167466	1.269084
H	-5.216634	-0.612483	1.100095
H	-4.425076	0.372471	2.344187
H	-4.788571	1.079413	0.760468
C	-2.585003	-1.430691	1.669019
H	-2.486988	-1.063030	2.695757
H	-3.275802	-2.280227	1.673287
H	-1.601169	-1.782902	1.339759
C	-3.202408	-2.098618	-1.090146
H	-4.183483	-2.454702	-0.755711
H	-3.143002	-2.236175	-2.175078
H	-2.429584	-2.716812	-0.626022
C	-3.974156	0.224988	-1.594676
H	-3.866624	1.291632	-1.369006
H	-3.736167	0.076499	-2.653107
H	-5.018988	-0.060953	-1.434802
B	-1.278845	0.701544	-0.146621
C	-0.261243	2.880817	-0.992691
H	0.649313	3.487317	-1.093398
H	-0.654457	2.704712	-2.002184
H	-0.998517	3.479588	-0.442503
C	3.231526	0.607462	0.981466
C	2.762116	-0.715918	1.542908
C	4.481046	0.455842	0.143629
H	3.511967	1.223498	1.845924
C	2.608965	-1.885326	0.889288
H	2.495013	-0.707429	2.599142
C	4.704953	-0.429763	-0.846906
H	5.266013	1.185772	0.341413
C	2.964074	-2.197801	-0.484264
H	2.180006	-2.713065	1.453957
C	3.876241	-1.553701	-1.248932
H	5.631992	-0.317847	-1.408993
H	2.492794	-3.084798	-0.904473
H	4.083369	-1.964755	-2.235598

48

PR-tropylium-Trans_c

Eopt -877.304511

C	-0.033875	1.736944	-0.239684
C	-2.151154	1.472570	0.247752
C	-1.224073	0.926272	-0.861629
H	-1.077995	-0.154534	-0.787546
H	-1.487608	1.171620	-1.896671
C	-0.900133	1.918647	1.041009
H	-0.629219	1.207034	1.828235
H	-0.925538	2.926518	1.472376
H	-2.696493	2.349606	-0.123356
O	2.082903	0.565353	-1.169610
O	1.637872	0.209242	1.043212
C	2.920166	-0.548524	-0.772027
C	2.916909	-0.422870	0.790517
C	4.287627	-0.402243	-1.420189
H	4.966937	-1.182330	-1.058147
H	4.195908	-0.509528	-2.506283
H	4.733634	0.573515	-1.211205
C	2.232752	-1.818965	-1.273707
H	2.098925	-1.746453	-2.357997
H	2.834144	-2.708835	-1.060565
H	1.244636	-1.949411	-0.818461
C	2.969427	-1.747762	1.535476
H	3.886720	-2.291882	1.283430
H	2.968802	-1.565055	2.615367
H	2.110900	-2.380929	1.297270
C	3.994174	0.522325	1.321933
H	3.979304	1.484925	0.799314
H	3.810917	0.709843	2.385029
H	4.992927	0.085539	1.218154
B	1.256205	0.853625	-0.108239
C	0.215383	3.052867	-0.979786
H	-0.718911	3.614938	-1.115590
H	0.910485	3.696054	-0.424502
H	0.643710	2.874178	-1.973424
C	-3.179488	0.568007	0.955075
C	-4.381612	0.284977	0.082815
C	-2.601263	-0.692821	1.557974
H	-3.543620	1.169542	1.798380
C	-4.498591	-0.643734	-0.886505
H	-5.231097	0.951342	0.232805
C	-2.328043	-1.860614	0.942160
H	-2.353409	-0.630739	2.617007
C	-3.568668	-1.706172	-1.231845
H	-5.413698	-0.625470	-1.478230
C	-2.624068	-2.245771	-0.427041
H	-1.834452	-2.627228	1.539295
H	-3.716379	-2.163678	-2.208712
H	-2.069578	-3.102249	-0.807374

48

PR-tropylium-Trans_d

Eopt -877.309321

C	0.410860	1.606406	0.201322
C	-1.768647	1.382124	0.084310
C	-0.620709	1.324914	-0.949232
H	-0.520153	0.322229	-1.384125
H	-0.636357	2.057419	-1.764082
C	-0.721343	1.260326	1.214829
H	-0.650188	0.233591	1.591934
H	-0.825776	1.940842	2.067817
H	-2.244015	2.371384	0.088508
O	2.836605	0.960698	-0.471414
O	1.647248	-0.633380	0.652721
C	3.598414	-0.265235	-0.604280
C	2.992951	-1.157630	0.534516
C	5.077826	0.049310	-0.447793
H	5.666961	-0.874795	-0.452814
H	5.414387	0.670804	-1.284427
H	5.282180	0.588572	0.480674

C	3.322620	-0.808165	-2.006444
H	3.604674	-0.049473	-2.743802
H	3.906094	-1.713108	-2.205294
H	2.261411	-1.040560	-2.148073
C	2.920061	-2.641548	0.209603
H	3.923585	-3.042067	0.026494
H	2.487448	-3.184217	1.056849
H	2.300632	-2.835158	-0.669900
C	3.671108	-0.950466	1.888682
H	3.736414	0.111511	2.149140
H	3.082343	-1.453968	2.662438
H	4.681018	-1.373501	1.896771
B	1.651449	0.645055	0.149546
C	0.805149	3.083945	0.251836
H	-0.081026	3.733041	0.231198
H	1.362494	3.314526	1.168890
H	1.437870	3.355503	-0.601453
C	-2.841319	0.307087	-0.000337
C	-3.674179	0.412702	-1.251844
C	-3.799854	0.348953	1.161696
H	-2.346376	-0.679105	-0.000335
C	-4.519139	-0.565625	-1.639757
H	-3.561074	1.303357	-1.870262
C	-4.679448	-0.645085	1.406281
H	-3.752319	1.204660	1.835466
C	-4.867553	-1.724883	-0.842781
H	-5.006805	-0.482607	-2.610407
C	-4.940036	-1.759813	0.517541
H	-5.265575	-0.612384	2.323999
H	-5.230434	-2.596805	-1.385206
H	-5.356065	-2.657403	0.972928

48

PR-tropylium-Trans_e

Eopt -877.308896

C	0.381964	2.050335	-0.089553
C	-1.808322	1.821666	-0.274459
C	-0.626459	1.867157	-1.274263
H	-0.497443	0.952020	-1.865365
H	-0.649030	2.715647	-1.967353
C	-0.817134	2.010390	0.905435
H	-0.795743	1.186159	1.627035
H	-0.958431	2.943685	1.462034
H	-2.485381	2.675272	-0.388938
O	2.355949	0.596933	-0.932381
O	1.416715	-0.057096	1.044201
C	2.878965	-0.728788	-0.668410
C	2.577382	-0.906145	0.860521
C	4.354348	-0.765234	-1.033645
H	4.791765	-1.731379	-0.757508
H	4.470997	-0.636324	-2.114946
H	4.915852	0.027946	-0.533464
C	2.094101	-1.697269	-1.553726
H	2.206197	-1.393380	-2.599590
H	2.466801	-2.721747	-1.451710
H	1.025178	-1.690420	-1.312938
C	2.219361	-2.323928	1.278226
H	3.047442	-3.007368	1.058886
H	2.029144	-2.355012	2.356285
H	1.323879	-2.684063	0.764979
C	3.679554	-0.352296	1.762278
H	3.953072	0.670881	1.482516
H	3.319930	-0.337173	2.796328
H	4.578098	-0.976629	1.720944
B	1.402148	0.859193	0.021624
C	1.116237	3.394210	-0.121913
H	0.402656	4.221732	-0.237979
H	1.676965	3.566839	0.806185
H	1.824049	3.442928	-0.958551
C	-2.661723	0.553089	-0.289737
C	-3.698694	0.542922	0.803429
C	-1.891573	-0.734730	-0.136243
H	-3.188676	0.510502	-1.257298
C	-4.738342	-0.318499	0.799522
H	-3.608505	1.278202	1.603285
C	-2.452697	-1.935464	-0.392719
H	-0.850288	-0.695442	0.179836
C	-4.884847	-1.434227	-0.112858
H	-5.520383	-0.200508	1.548752
C	-3.861068	-2.157950	-0.648363
H	-1.819276	-2.822046	-0.375399
H	-5.896495	-1.805415	-0.270758
H	-4.120745	-3.060999	-1.199071

48

PR-tropylium-Trans_f

Eopt -877.308485

C	-0.357219	1.982702	-0.158243
C	1.840541	1.807275	-0.263426
C	0.804354	1.924376	0.886114
H	0.776663	1.067273	1.569550
H	0.900593	2.832729	1.491467
C	0.693156	1.861259	-1.303732
H	0.600684	0.962251	-1.923480
H	0.728366	2.731399	-1.969070
H	2.497371	2.682338	-0.317766
O	-2.057849	0.488762	1.107933
O	-1.729788	-0.025541	-1.093394
C	-2.741255	-0.771309	0.893890
C	-2.865880	-0.819844	-0.669312
C	-4.067970	-0.749258	1.636806
H	-4.645581	-1.653305	1.413201
H	-3.886295	-0.720796	2.716464
H	-4.668619	0.123820	1.369266
C	-1.841027	-1.868082	1.462800
H	-1.644442	-1.655609	2.518702
H	-2.318884	-2.850796	1.393130
H	-0.878478	-1.913627	0.941911
C	-2.749739	-2.210040	-1.274222
H	-3.545364	-2.860157	-0.893102
H	-2.853009	-2.149452	-2.362822
H	-1.784247	-2.670835	-1.049908
C	-4.117465	-0.118872	-1.196503
H	-4.221494	0.888399	-0.778517

H	-4.043268	-0.028608	-2.285173
H	-5.022009	-0.688873	-0.960390
B	-1.387842	0.799411	-0.050702
C	-1.109605	3.319175	-0.154879
H	-0.404102	4.159055	-0.221031
H	-1.796514	3.394600	-1.008177
H	-1.695443	3.446647	0.764093
C	2.729477	0.563070	-0.286454
C	1.986215	-0.748440	-0.241066
C	3.698193	0.533686	0.868333
H	3.314182	0.578725	-1.221087
C	2.592411	-1.926754	-0.496241
H	0.919347	-0.747033	-0.024069
C	4.755396	-0.304589	0.904221
H	3.542851	1.239964	1.684170
C	4.020308	-2.111380	-0.658568
H	1.978695	-2.824557	-0.563005
C	4.988677	-1.385482	-0.032317
H	5.484047	-0.194903	1.706680
H	4.336601	-2.988097	-1.221994
H	6.017246	-1.728339	-0.135042

48

SM-tropylium_cis_b

Eopt -877.163486

C	0.016138	-0.328421	0.528134
C	-0.717580	0.701035	-0.279205
C	-1.145327	-0.729340	-0.342064
H	-2.115020	-0.998969	0.099444
H	-0.899344	-1.294473	-1.242288
C	-0.671090	0.823365	1.210574
H	-1.603313	0.668701	1.774160
H	-0.013417	1.578391	1.641965
H	-0.318567	1.343774	-1.054400
O	2.478152	0.020849	1.318513
O	2.121738	-0.886074	-0.825523
C	3.308888	0.707070	0.410256
C	3.393797	-0.279371	-0.809999
C	4.654529	0.997251	1.076615
H	5.367207	1.434466	0.365193
H	4.515986	1.713034	1.896542
H	5.095803	0.089325	1.498056
C	2.661585	2.048955	0.028536
H	2.443696	2.608813	0.945903
H	3.319667	2.665424	-0.597064
H	1.721003	1.892030	-0.505802
C	3.643258	0.405277	-2.154368
H	4.576737	0.982916	-2.143096
H	3.724704	-0.349829	-2.946283
H	2.820806	1.077495	-2.417291
C	4.474497	-1.355831	-0.605660
H	4.386129	-1.824366	0.378962
H	4.340498	-2.136142	-1.364347
H	5.489541	-0.951105	-0.707419
B	1.559086	-0.887790	0.564782
C	1.553539	-2.380389	1.250175
H	0.923400	-3.088352	0.686041
H	2.562141	-2.818706	1.305944
H	1.156278	-2.354646	2.279167
C	-3.777435	1.599515	-0.267412
C	-3.900315	0.908213	-1.471768
C	-4.175564	1.198327	1.007155
H	-3.306420	2.576097	-0.328974
C	-4.447942	-0.352660	-1.697493
H	-3.506510	1.416018	-2.347953
C	-4.800157	0.012010	1.390660
H	-3.973263	1.904522	1.807617
C	-5.022241	-1.230173	-0.777945
H	-4.424760	-0.698588	-2.727328
C	-5.183385	-1.067056	0.596199
H	-5.016799	-0.083255	2.451488
H	-5.389575	-2.167154	-1.188003
H	-5.662334	-1.891334	1.117599

48

SM-tropylium_cis_c

Eopt -877.163483

C	0.040191	0.820460	-0.111478
C	-0.709216	0.073152	0.950023
C	-0.794216	1.564768	0.898139
H	-1.751268	2.023588	0.609008
H	-0.243331	2.131823	1.649760
C	-1.009848	-0.187968	-0.491178
H	-1.986229	0.131658	-0.881385
H	-0.639180	-1.114950	-0.929894
H	-0.304651	-0.575252	1.717750
O	2.135712	-0.181548	-1.296148
O	2.459911	0.981036	0.726188
C	2.973016	-0.961370	-0.473914
C	3.533481	0.083751	0.557226
C	4.045396	-1.629593	-1.335560
H	4.783132	-2.158197	-0.717889
H	3.579680	-2.363597	-2.005166
H	4.571132	-0.898446	-1.956584
C	2.146960	-2.065092	0.208218
H	1.617061	-2.636048	-0.563748
H	2.774639	-2.763327	0.776520
H	1.403721	-1.636283	0.885426
C	3.909934	-0.513228	1.913715
H	4.669553	-1.298954	1.809595
H	4.322483	0.269705	2.562196
H	3.036008	-0.937373	2.417508
C	4.753886	0.840637	0.003955
H	4.558448	1.227623	-1.000556
H	4.964154	1.693893	0.659766
H	5.651840	0.210871	-0.035111
B	1.621829	0.988499	-0.516848
C	1.789426	2.367117	-1.392636
H	1.459220	3.255111	-0.828344
H	2.832704	2.546187	-1.695853
H	1.186728	2.338357	-2.316384
C	-3.971518	0.304517	1.493501
C	-4.478397	1.240359	0.594425
C	-3.778387	-1.063178	1.306512

H	-3.675057	0.694402	2.463110
C	-4.935525	1.039134	-0.708514
H	-4.527838	2.263279	0.957723
C	-4.047037	-1.830335	0.174215
H	-3.355775	-1.599267	2.151547
C	-4.987117	-0.146213	-1.437116
H	-5.293939	1.926357	-1.223783
C	-4.584142	-1.422673	-1.045489
H	-3.801370	-2.886121	0.251626
H	-5.382936	-0.064878	-2.445876
H	-4.704376	-2.204036	-1.791298

48

SM-tropylium_trans_b

Eopt -877.165538

C	0.102135	0.950027	-0.261127
C	-0.985138	1.392758	0.683516
C	-0.968789	1.909320	-0.717098
H	-1.758750	1.572581	-1.403372
H	-0.660020	2.942832	-0.878298
C	-0.826667	-0.057151	0.361536
H	-1.610271	-0.542698	-0.236208
H	-0.384629	-0.698581	1.123932
H	-0.848325	1.917737	1.621508
O	2.478017	0.471804	-1.252372
O	2.169749	0.357531	1.078371
C	3.016380	-0.747935	-0.799501
C	3.245596	-0.484345	0.731885
C	4.294071	-1.058114	-1.580241
H	4.805032	-1.942132	-1.176744
H	4.047986	-1.261957	-2.629889
H	4.989326	-0.213746	-1.558592
C	2.010723	-1.886337	-1.047182
H	1.729247	-1.880363	-2.107193
H	2.432603	-2.871924	-0.811906
H	1.101162	-1.744607	-0.457142
C	3.202423	-1.743006	1.599084
H	3.961229	-2.471878	1.285749
H	3.400444	-1.480518	2.645901
H	2.220137	-2.223254	1.555052
C	4.574386	0.244316	0.998267
H	4.688321	1.113002	0.342738
H	4.578184	0.600157	2.035544
H	5.443606	-0.411229	0.859477
B	1.724607	1.125526	-0.130447
C	2.113814	2.718702	-0.070843
H	1.576878	3.260805	0.725410
H	3.189120	2.856702	0.119060
H	1.890363	3.237789	-1.017225
C	-4.137914	1.081160	0.673111
C	-4.354952	1.016802	-0.702926
C	-4.019176	0.030310	1.581795
H	-4.048064	2.079801	1.090227
C	-4.510719	-0.111002	-1.507441
H	-4.408830	1.972840	-1.216142
C	-4.084448	-1.339512	1.338359
H	-3.841023	0.318656	2.614152
C	-4.492707	-1.455256	-1.137544
H	-4.666996	0.084519	-2.565132
C	-4.299955	-2.001163	0.129152
H	-3.951289	-1.978629	2.207174
H	-4.639926	-2.169162	-1.943416
H	-4.315050	-3.086503	0.182780

48

TS1-tropylium-Cis_a

Eopt -877.154730

C	-0.378633	1.926028	0.276695
C	0.995388	1.169658	0.103310
C	0.628014	2.402591	-0.699334
H	1.239805	3.288593	-0.485397
H	0.384047	2.277041	-1.753824
C	0.505240	1.699596	1.439522
H	1.110844	2.516998	1.852527
H	0.148954	0.988887	2.183271
H	0.901173	0.152941	-0.254255
O	-2.393935	0.493003	1.094644
O	-1.730415	0.284793	-1.150590
C	-2.393005	-0.881271	0.774320
C	-2.432901	-0.887538	-0.798782
C	-3.601740	-1.550323	1.431735
H	-3.695059	-2.599528	1.123115
H	-3.488694	-1.529331	2.522693
H	-4.530793	-1.029819	1.182940
C	-1.123725	-1.545169	1.336070
H	-1.062790	-1.334989	2.410402
H	-1.135547	-2.634340	1.203682
H	-0.219322	-1.153410	0.863507
C	-1.743549	-2.094137	-1.436209
H	-2.199331	-3.036318	-1.105777
H	-1.836233	-2.039813	-2.527857
H	-0.676938	-2.118675	-1.191903
C	-3.868656	-0.804248	-1.344745
H	-4.429401	0.007527	-0.872256
H	-3.822562	-0.600393	-2.421011
H	-4.423422	-1.739248	-1.197259
B	-1.863792	1.284663	-0.049772
C	-2.863087	2.534370	-0.410830
H	-2.504889	3.111436	-1.278874
H	-3.880991	2.190090	-0.648608
H	-2.951777	3.244776	0.427601
C	3.317332	1.089655	0.286450
C	3.459608	0.207087	1.387112
C	3.668373	0.809349	-1.057957
H	3.256378	2.142443	0.541029
C	3.581392	-1.161364	1.370572
H	3.401060	0.675057	2.366314
C	3.847378	-0.416464	-1.653198
H	3.745020	1.677804	-1.707170
C	3.630037	-2.031814	0.256522
H	3.617649	-1.647163	2.342537
C	3.749273	-1.703773	-1.074765
H	4.062794	-0.400554	-2.718777
H	3.620263	-3.093183	0.491226

H 3.823067 -2.535908 -1.770360
48
TS1-tropylium-Cis_b
Eopt -877.157378
C -0.036179 1.333306 0.117134
C 0.970218 0.179054 0.567003
C 1.215020 1.077310 -0.623290
H 2.079051 1.748918 -0.498563
H 1.122745 0.661834 -1.626458
C 0.495252 1.286635 1.493118
H 1.292587 1.977012 1.801243
H -0.216090 1.048236 2.282470
H 0.560035 -0.820893 0.512527
O -2.572960 1.076168 0.667840
O -1.705273 -0.148219 -1.142027
C -3.089770 -0.235055 0.746462
C -2.885858 -0.790912 -0.710377
C -4.551066 -0.173399 1.193576
H -5.019327 -1.165739 1.165801
H -4.607858 0.196852 2.224648
H -5.133622 0.504635 0.563462
C -2.303990 -1.037885 1.796541
H -2.324644 -0.490507 2.746239
H -2.737871 -2.030951 1.967685
H -1.258911 -1.166176 1.502135
C -2.686672 -2.304815 -0.780886
H -3.544875 -2.841154 -0.356156
H -2.579116 -2.616372 -1.827133
H -1.783750 -2.613535 -0.245290
C -4.046862 -0.410091 -1.644938
H -4.278204 0.657147 -1.580879
H -3.752158 -0.630292 -2.677753
H -4.960105 -0.975904 -1.422215
B -1.579088 1.161159 -0.436509
C -1.879624 2.459692 -1.394161
H -1.178649 2.518345 -2.242708
H -2.896036 2.430047 -1.815090
H -1.788353 3.409030 -0.840889
C 3.103187 -0.469317 1.355731
C 3.249184 -1.693177 0.652491
C 3.857695 0.710752 1.153596
H 2.614466 -0.546463 2.320035
C 3.886906 -1.925021 -0.541377
H 2.751827 -2.546634 1.106554
C 4.632502 1.053408 0.067259
H 3.779097 1.456273 1.940229
C 4.586813 -1.008846 -1.362025
H 3.838244 -2.943860 -0.917456
C 4.917899 0.299486 -1.094197
H 5.087318 2.040454 0.103954
H 4.943429 -1.408719 -2.307972
H 5.504151 0.812272 -1.852333
48
TS1-tropylium-Cis_c
Eopt -877.157638
C 0.105252 1.516071 -0.309176
C -0.956286 0.748894 0.599331
C -0.633087 2.222874 0.758655
H -1.465603 2.892772 0.504535
H -0.060107 2.540319 1.629499
C -1.016162 0.772066 -0.912018
H -1.891949 1.311078 -1.307119
H -0.762261 -0.126996 -1.471364
H -0.533137 -0.039008 1.208751
O 2.058497 0.151603 -1.360336
O 2.196375 0.693697 0.923047
C 2.490290 -1.000466 -0.668339
C 3.027871 -0.423534 0.692012
C 3.549736 -1.720619 -1.503968
H 3.988517 -2.562635 -0.953284
H 3.093157 -2.118281 -2.418779
H 4.353931 -1.041476 -1.800966
C 1.306430 -1.962601 -0.470695
H 0.852978 -2.168913 -1.447340
H 1.621091 -2.918700 -0.033976
H 0.538295 -1.527524 0.174258
C 2.910668 -1.388334 1.871782
H 3.462392 -2.318323 1.683914
H 3.329663 -0.924480 2.773220
H 1.865974 -1.639292 2.079535
C 4.490779 0.040261 0.587530
H 4.645190 0.681035 -0.285465
H 4.738271 0.623309 1.482386
H 5.189268 -0.803552 0.525512
B 1.727526 1.230385 -0.388539
C 2.441430 2.644183 -0.815058
H 2.239599 3.444388 -0.084476
H 3.534576 2.544036 -0.891524
H 2.082695 3.008838 -1.791775
C -3.191594 0.445003 1.326265
C -3.911793 1.178609 0.354376
C -3.192481 -0.964129 1.490773
H -2.878816 1.011245 2.195970
C -4.482487 0.718755 -0.812267
H -3.983842 2.247716 0.534692
C -3.600922 -1.930219 0.604087
H -2.774151 -1.318089 2.429707
C -4.541220 -0.602388 -1.311739
H -4.949637 1.474176 -1.439696
C -4.151164 -1.766032 -0.688957
H -3.472657 -2.959989 0.928320
H -4.985923 -0.710300 -2.297650
H -4.325677 -2.686033 -1.241445
48
TS1-tropylium-Trans_a
Eopt -877.155804
C -0.215176 -1.058621 -0.514007
C 1.285170 -0.566139 -0.325235
C 0.896117 -2.031623 -0.414645
H 1.268415 -2.541477 -1.312189
H 0.944627 -2.639862 0.487469
C 0.392741 -0.103320 -1.464829

H	0.721571	-0.435021	-2.458257
H	0.004753	0.913232	-1.433977
H	1.477636	-0.017288	0.588328
O	-2.723034	-1.287776	0.096701
O	-1.622696	0.723695	0.618809
C	-3.497325	-0.222692	-0.412600
C	-2.992338	1.007660	0.422615
C	-4.979368	-0.544752	-0.221885
H	-5.611534	0.308728	-0.498888
H	-5.260071	-1.392301	-0.859217
H	-5.198327	-0.819059	0.814089
C	-3.229115	-0.055517	-1.917790
H	-3.396456	-1.019302	-2.413201
H	-3.897682	0.684005	-2.376097
H	-2.195720	0.246202	-2.109271
C	-3.131011	2.351291	-0.292115
H	-4.176368	2.557408	-0.555534
H	-2.783794	3.159094	0.364007
H	-2.530166	2.380612	-1.206136
C	-3.691763	1.103812	1.788697
H	-3.680912	0.140867	2.308346
H	-3.153032	1.829665	2.409378
H	-4.733103	1.437750	1.699282
B	-1.414840	-0.753209	0.579246
C	-1.043966	-1.412596	2.030900
H	-0.064945	-1.088222	2.420055
H	-1.793967	-1.132380	2.785288
H	-1.026603	-2.513654	1.996281
C	3.426578	-0.459762	-1.159364
C	3.573176	0.937105	-1.366393
C	4.140187	-1.238429	-0.210601
H	3.029524	-1.012343	-2.004284
C	4.103283	1.874356	-0.514331
H	3.158538	1.311586	-2.298787
C	4.805019	-0.815064	0.913832
H	4.104069	-2.312342	-0.374959
C	4.668928	1.687285	0.770033
H	4.065140	2.904824	-0.859088
C	4.978302	0.503913	1.398442
H	5.240656	-1.597481	1.530611
H	4.932378	2.597960	1.302054
H	5.458779	0.586050	2.370053

48

TS1-tropylium-Trans_b

Eopt -877.158831

C	0.103633	1.025715	-0.555059
C	-1.287945	1.150098	0.233559
C	-0.838591	2.129353	-0.837652
H	-1.455892	2.101050	-1.745072
H	-0.538718	3.133741	-0.541274
C	-0.859917	-0.091412	-0.517815
H	-1.487176	-0.307781	-1.396156
H	-0.558928	-0.971991	0.047247
H	-1.189092	1.303526	1.301104
O	2.668738	0.913328	-0.855349
O	1.722747	-0.099952	1.044152
C	3.153595	-0.410590	-0.779230
C	2.918723	-0.781949	0.727995
C	4.619882	-0.433551	-1.210427
H	5.066302	-1.423611	-1.051440
H	4.696857	-0.197461	-2.278942
H	5.208986	0.306798	-0.661513
C	2.353930	-1.314484	-1.732740
H	2.377279	-0.873744	-2.736539
H	2.776538	-2.325215	-1.794434
H	1.307910	-1.394968	-1.424346
C	2.730381	-2.276626	0.984958
H	3.602605	-2.852262	0.649511
H	2.602112	-2.456069	2.059635
H	1.842786	-2.660892	0.473293
C	4.051722	-0.267597	1.631527
H	4.274671	0.784266	1.427943
H	3.732533	-0.350207	2.677247
H	4.975096	-0.847662	1.509738
B	1.589353	1.094829	0.160932
C	1.782864	2.520984	0.940021
H	0.985231	2.727856	1.672251
H	2.732877	2.533261	1.494422
H	1.810412	3.376170	0.245936
C	-3.627178	1.171715	0.190264
C	-3.909318	0.678547	-1.107380
C	-3.897232	0.507758	1.416766
H	-3.525969	2.248410	0.262773
C	-4.160930	-0.619678	-1.490715
H	-3.881373	1.416810	-1.904243
C	-4.156570	-0.822699	1.631540
H	-3.848878	1.138150	2.301238
C	-4.258680	-1.781331	-0.689197
H	-4.304040	-0.778055	-2.557085
C	-4.253168	-1.869650	0.683092
H	-4.293142	-1.120086	2.668337
H	-4.394110	-2.713546	-1.231399
H	-4.384729	-2.865255	1.099646

48

TS1-tropylium-Trans_c

Eopt -877.158831

C	0.103623	1.025459	-0.555112
C	-1.288089	1.149735	0.233250
C	-0.838587	2.129065	-0.837836
H	-1.455759	2.100778	-1.745346
H	-0.538818	3.133452	-0.541358
C	-0.859894	-0.091720	-0.518123
H	-1.486957	-0.308013	-1.396632
H	-0.559004	-0.972342	0.046926
H	-1.189419	1.303122	1.300817
O	2.668774	0.913515	-0.855125
O	1.722751	-0.100210	1.044122
C	3.153819	-0.410346	-0.779167
C	2.918887	-0.781941	0.727990
C	4.620134	-0.433021	-1.210279
H	5.066638	-1.423093	-1.051598
H	4.697135	-0.196570	-2.278711

H	5.209148	0.307202	-0.661100
C	2.354350	-1.314213	-1.732865
H	2.377764	-0.873356	-2.736611
H	2.777062	-2.324894	-1.794639
H	1.308307	-1.394834	-1.424584
C	2.730811	-2.276679	0.984789
H	3.603153	-2.852123	0.649318
H	2.602542	-2.456244	2.059447
H	1.843298	-2.661070	0.473076
C	4.051715	-0.267470	1.631673
H	4.274505	0.784447	1.428199
H	3.732441	-0.350225	2.677355
H	4.975197	-0.847370	1.509917
B	1.589246	1.094680	0.161066
C	1.782397	2.520744	0.940427
H	0.984541	2.727393	1.672478
H	2.732254	2.533064	1.495088
H	1.810005	3.376044	0.246485
C	-3.627321	1.171790	0.189633
C	-3.909400	0.678105	-1.107831
C	-3.897485	0.508364	1.416400
H	-3.526089	2.248513	0.261704
C	-4.160975	-0.620284	-1.490635
H	-3.881427	1.416053	-1.904982
C	-4.156830	-0.821995	1.631738
H	-3.849215	1.139183	2.300568
C	-4.258723	-1.781593	-0.688613
H	-4.304013	-0.779127	-2.556945
C	-4.253303	-1.869343	0.683715
H	-4.293500	-1.118941	2.668648
H	-4.394092	-2.714043	-1.230425
H	-4.384890	-2.864778	1.100668

48

TS2-tropylium-Trans_a

Eopt -877.201173

C	-0.146739	0.347738	0.346055
C	1.840286	0.313577	0.320611
C	0.782661	1.362025	-0.182562
H	0.870044	2.253277	0.449949
H	0.683302	1.630472	-1.234337
C	0.776310	-0.162887	1.375639
H	0.864109	0.488917	2.252895
H	0.672938	-1.208008	1.667351
H	2.020246	-0.454937	-0.431102
O	-2.197911	0.484643	-1.170307
O	-2.188671	-1.117049	0.554473
C	-3.323572	0.723805	-0.338056
C	-3.522036	-0.665154	0.362369
C	-4.497215	1.173269	-1.200980
H	-5.418239	1.240817	-0.609183
H	-4.292925	2.164406	-1.623189
H	-4.665946	0.482255	-2.031618
C	-2.984921	1.834151	0.668662
H	-2.631875	2.715349	0.121131
H	-3.856357	2.127003	1.265462
H	-2.194717	1.526983	1.366346
C	-4.232636	-0.594003	1.711174
H	-5.229639	-0.147580	1.609178
H	-4.355885	-1.603771	2.120414
H	-3.660164	-0.006812	2.435278
C	-4.254187	-1.665057	-0.542978
H	-3.803920	-1.695316	-1.540596
H	-4.171924	-2.665181	-0.102303
H	-5.319134	-1.424730	-0.647317
B	-1.323954	-0.436149	-0.431593
C	-0.498839	-1.445491	-1.435983
H	0.138906	-2.167665	-0.908374
H	-1.264112	-2.016250	-1.981226
H	0.120612	-0.925029	-2.179321
C	3.159660	0.878024	0.878816
C	3.979101	-0.187334	1.565240
C	3.984870	1.542289	-0.196102
H	2.888142	1.635239	1.621248
C	4.543899	-1.245589	0.946894
H	4.080332	-0.106953	2.646054
C	4.551019	0.903164	-1.241196
H	4.089644	2.624009	-0.134724
C	4.626550	-1.480209	-0.480561
H	5.011982	-2.002919	1.574635
C	4.629679	-0.528554	-1.449726
H	5.023793	1.515626	-2.008060
H	4.812239	-2.508193	-0.786458
H	4.817645	-0.853615	-2.471395

48

TS2-tropylium-Trans_b

Eopt -877.199752

C	-0.047823	-0.380487	0.264388
C	1.808351	-1.055197	0.240072
C	1.137118	0.235125	-0.349843
H	1.515649	1.091489	0.219146
H	1.110346	0.441886	-1.419483
C	0.689678	-1.042381	1.348509
H	1.028857	-0.355460	2.134245
H	0.272675	-1.958424	1.767303
H	1.679777	-1.903864	-0.434743
O	-1.994490	0.294453	-1.235033
O	-2.441617	-1.058330	0.638669
C	-2.922421	0.978823	-0.406713
C	-3.546717	-0.189947	0.432072
C	-3.915470	1.731521	-1.285161
H	-4.723165	2.166701	-0.684073
H	-3.405770	2.548939	-1.809037
H	-4.356617	1.072729	-2.038376
C	-2.170373	1.985495	0.477882
H	-1.560409	2.634615	-0.160450
H	-2.855361	2.616796	1.055492
H	-1.502816	1.484970	1.191596
C	-4.115016	0.238800	1.782198
H	-4.901193	0.993962	1.657884
H	-4.556160	-0.626022	2.291882
H	-3.336905	0.649612	2.432342
C	-4.626436	-0.946582	-0.353642

H	-4.268489	-1.217848	-1.352224
H	-4.870534	-1.870522	0.182971
H	-5.547202	-0.360257	-0.459550
B	-1.448195	-0.807919	-0.429530
C	-1.072643	-2.115646	-1.345456
H	-0.683237	-2.961779	-0.762210
H	-2.007260	-2.446698	-1.820798
H	-0.352584	-1.898042	-2.146874
C	3.279227	-1.034154	0.739108
C	4.247217	-1.241746	-0.400532
C	3.646873	0.155934	1.584299
H	3.347558	-1.918829	1.381552
C	4.646770	-0.322618	-1.300931
H	4.618510	-2.258533	-0.519582
C	3.931203	1.403206	1.150089
H	3.661725	-0.011635	2.659920
C	4.375473	1.102042	-1.297610
H	5.271671	-0.672871	-2.121624
C	4.074323	1.862777	-0.217426
H	4.093314	2.163848	1.913013
H	4.534048	1.616967	-2.243328
H	4.007424	2.939105	-0.364242

48
TS2-tropylium-Trans_c
Eopt -877.199738

C	0.039959	-0.330455	0.313590
C	-1.820151	-0.988699	0.382804
C	-0.708847	-0.805450	1.483834
H	-1.047321	0.000628	2.147037
H	-0.299770	-1.645389	2.045706
C	-1.136138	0.186975	-0.400375
H	-1.514919	1.124184	0.022450
H	-1.102316	0.220702	-1.489035
H	-1.693283	-1.935387	-0.146228
O	2.458115	-1.005278	0.768297
O	1.966193	0.137287	-1.229849
C	3.222192	0.191161	0.743624
C	3.255388	0.535448	-0.786116
C	4.589138	-0.075551	1.363908
H	5.251996	0.790197	1.244765
H	4.480475	-0.273063	2.437045
H	5.067814	-0.946890	0.908290
C	2.501695	1.273548	1.562732
H	2.295429	0.882692	2.565520
H	3.107091	2.181469	1.665574
H	1.546185	1.564481	1.106962
C	3.462730	2.016162	-1.092801
H	4.406965	2.379107	-0.667995
H	3.500102	2.169897	-2.177826
H	2.645172	2.625446	-0.696265
C	4.309767	-0.289155	-1.537109
H	4.224027	-1.352970	-1.292525
H	4.145720	-0.173564	-2.614561
H	5.330831	0.040629	-1.310340
B	1.442338	-0.876292	-0.286921
C	1.066535	-2.321218	-0.965385
H	0.331146	-2.243073	-1.778191
H	1.996096	-2.717540	-1.399039
H	0.695628	-3.063591	-0.244527
C	-3.293964	-0.877650	0.860752
C	-3.654505	0.432596	1.508107
C	-4.256692	-1.251895	-0.240197
H	-3.375650	-1.651037	1.632308
C	-3.925216	1.599447	0.883414
H	-3.675575	0.434859	2.596660
C	-4.642693	-0.480575	-1.275164
H	-4.635456	-2.272193	-0.203329
C	-4.057532	1.842151	-0.539803
H	-4.084508	2.470744	1.517779
C	-4.359626	0.925417	-1.490773
H	-5.265405	-0.949242	-2.036285
H	-3.980554	2.882140	-0.851207
H	-4.508327	1.288607	-2.505982

48
TS2-tropylium-Trans_d
Eopt -877.201633

C	0.291059	-0.003992	-0.016364
C	-1.617853	-0.159514	0.454662
C	-0.683521	1.079443	0.177962
H	-1.035228	1.548754	-0.747010
H	-0.457241	1.820914	0.943995
C	-0.678611	-1.004112	-0.487907
H	-1.029348	-0.852479	-1.514430
H	-0.449515	-2.051716	-0.293358
H	-1.545646	-0.491524	1.492582
O	2.497429	0.973418	0.804004
O	2.475704	-1.238738	-0.000293
C	3.400446	0.891112	-0.288987
C	3.718993	-0.643367	-0.344518
C	4.606640	1.780466	-0.008831
H	5.383873	1.641767	-0.770213
H	4.304673	2.834490	-0.021834
H	5.037498	1.566052	0.973189
C	2.700298	1.381317	-1.565885
H	2.290674	2.381643	-1.386016
H	3.389887	1.440138	-2.415746
H	1.872419	0.722006	-1.858954
C	4.158900	-1.145208	-1.717006
H	5.062305	-0.623746	-2.057021
H	4.387051	-2.216467	-1.665919
H	3.373229	-1.005358	-2.465368
C	4.764780	-1.050180	0.702507
H	4.507183	-0.653853	1.690152
H	4.788836	-2.143786	0.770395
H	5.770710	-0.702691	0.437807
B	1.686726	-0.252012	0.769970
C	1.238687	-0.742761	2.269057
H	0.653531	-1.672861	2.268026
H	2.166384	-0.938618	2.825754
H	0.670900	0.013376	2.829418
C	-3.072271	-0.021192	0.015006
C	-3.823487	0.983579	0.849085

C	-3.820219	-1.325170	0.113423
H	-3.088652	0.313522	-1.033994
C	-5.044825	1.426623	0.486604
H	-3.348829	1.368678	1.750689
C	-5.040696	-1.480533	-0.439478
H	-3.343332	-2.159303	0.626550
C	-5.834680	0.886058	-0.601668
H	-5.499370	2.233012	1.060299
C	-5.832986	-0.412314	-1.015146
H	-5.492030	-2.471631	-0.438319
H	-6.587706	1.546051	-1.029280

48
TS-rotation-tropylium_a
Eopt -877.193714

C	0.017105	-1.537465	-0.633236
C	-1.587449	-0.769618	-0.470030
C	-0.551674	-0.715625	-1.681004
H	-1.034194	-1.202160	-2.531110
H	-0.052889	0.214704	-1.925706
C	-1.141483	-2.287754	-0.195638
H	-1.687875	-2.899368	-0.916512
H	-1.138323	-2.689126	0.813814
H	-1.358542	-0.076613	0.336848
O	1.189596	0.289195	0.787039
O	2.489417	-1.113786	-0.558340
C	2.242954	1.082460	0.255910
C	3.290196	0.020158	-0.256142
C	2.769458	1.994941	1.365690
H	3.650685	2.557943	1.033875
H	1.993201	2.717631	1.644680
H	3.034532	1.426284	2.261104
C	1.702297	1.978289	-0.867480
H	0.800944	2.489398	-0.509810
H	2.434002	2.740059	-1.162495
H	1.439787	1.410230	-1.762761
C	4.047088	0.439511	-1.515368
H	4.599852	1.372781	-1.351841
H	4.771439	-0.338169	-1.784889
H	3.373356	0.577727	-2.365536
C	4.325196	-0.358263	0.816102
H	3.857174	-0.609860	1.771000
H	4.880913	-1.237630	0.471231
H	5.042325	0.452728	0.990523
B	1.324943	-1.110567	0.343980
C	1.389169	-2.158229	1.592079
H	1.407896	-3.211027	1.267206
H	2.289403	-1.997003	2.199602
H	0.529871	-2.046110	2.271955
C	-3.069689	-0.646472	-0.922388
C	-4.012785	-0.975192	0.205764
C	-3.355219	0.734607	-1.454138
H	-3.223107	-1.368170	-1.727548
C	-4.147732	-0.253069	1.337759
H	-4.589357	-1.892522	0.103855
C	-3.329480	1.867245	-0.721171
H	-3.550176	0.811727	-2.522022
C	-3.554553	1.035375	1.627915
H	-4.781508	-0.665605	2.121581
C	-3.190867	1.974269	0.715956
H	-3.440330	2.810647	-1.253992
H	-3.496778	1.309184	2.679548
H	-2.864319	2.940877	1.094451

48
TS-rotation-tropylium_b
Eopt -877.191457

C	0.070937	1.286419	-0.487890
C	1.500127	0.876693	0.555525
C	0.977001	0.179636	-0.758863
H	1.732058	0.336575	-1.531194
H	0.585243	-0.830233	-0.725984
C	1.026177	2.283761	-0.060103
H	1.824320	2.571965	-0.750109
H	0.684216	3.091790	0.581803
H	0.945479	0.580235	1.445517
O	-1.682150	0.184067	1.067120
O	-2.254305	0.622811	-1.159306
C	-2.472674	-0.907954	0.613918
C	-3.176349	-0.345446	-0.678718
C	-3.440322	-1.303507	1.730932
H	-4.145574	-2.072195	1.391299
H	-2.876520	-1.714326	2.577149
H	-4.008853	-0.442881	2.093494
C	-1.573636	-2.117716	0.322333
H	-0.909130	-2.281276	1.178552
H	-2.163680	-3.029172	0.168031
H	-0.956288	-1.970472	-0.566513
C	-3.413921	-1.386781	-1.770935
H	-4.039775	-2.209194	-1.403157
H	-3.933473	-0.920597	-2.616439
H	-2.474656	-1.803568	-2.144869
C	-4.519182	0.337465	-0.371317
H	-4.440121	1.048220	0.455086
H	-4.843751	0.888832	-1.261049
H	-5.296708	-0.393660	-0.119726
B	-1.537828	1.189390	-0.003130
C	-2.013638	2.677635	0.463766
H	-1.824400	3.445666	-0.303471
H	-3.089850	2.702648	0.679604
H	-1.502390	3.008199	1.381820
C	3.033501	0.832269	0.885697
C	3.363716	-0.448289	1.609954
C	3.964891	1.097567	-0.262740
H	3.158219	1.649152	1.602460
C	3.544548	-1.665101	1.060353
H	3.408561	-0.370696	2.694895
C	4.318452	0.233028	-1.238898
H	4.380939	2.102118	-0.311268
C	3.623984	-2.002032	-0.346430
H	3.672041	-2.501998	1.745673
C	3.979358	-1.169932	-1.355871
H	4.947461	0.625703	-2.036835

H	3.486544	-3.053714	-0.590399
H	4.103036	-1.605373	-2.345497

48
TS-rotation-tropylium_c
Eopt -877.191113

C	0.128852	0.483679	-0.738172
C	1.423320	-0.513023	0.061751
C	0.497728	-0.838064	-1.197392
H	1.176273	-0.911077	-2.052249
H	-0.239156	-1.632590	-1.152182
C	1.447935	1.001711	-0.406934
H	2.180516	1.063119	-1.214105
H	1.518378	1.811042	0.314833
H	0.917836	-0.667880	1.014687
O	-1.712365	-0.010797	1.021112
O	-2.339253	0.790464	-1.086314
C	-2.912336	-0.681299	0.656281
C	-3.497347	0.208479	-0.504748
C	-3.812993	-0.772390	1.889478
H	-4.796251	-1.186010	1.633071
H	-3.352925	-1.434454	2.632917
H	-3.955951	0.206090	2.355764
C	-2.591236	-2.111897	0.202505
H	-1.950175	-2.587451	0.953763
H	-3.500868	-2.715491	0.098281
H	-2.069207	-2.132489	-0.756776
C	-4.250913	-0.573307	-1.579493
H	-5.094691	-1.125394	-1.147541
H	-4.650924	0.121658	-2.327259
H	-3.598848	-1.282080	-2.097394
C	-4.424076	1.321003	0.012536
H	-3.976718	1.878483	0.839151
H	-4.616217	2.025460	-0.804703
H	-5.386693	0.920523	0.352188
B	-1.325061	0.950643	-0.029515
C	-1.133037	2.465498	0.542777
H	-0.753421	3.163938	-0.220520
H	-2.082986	2.878662	0.906613
H	-0.429775	2.502779	1.389560
C	2.771682	-1.316126	0.076689
C	3.653664	-1.144981	-1.127127
C	3.505880	-1.075049	1.371033
H	2.434954	-2.357034	0.087170
C	4.483508	-0.111462	-1.388842
H	3.598536	-1.939688	-1.868787
C	4.270738	-0.010797	1.683578
H	3.350997	-1.829910	2.140096
C	4.803894	1.015880	-0.538147
H	4.990444	-0.122811	-2.352889
C	4.696636	1.065507	0.812136
H	4.641018	0.047126	2.706242
H	5.248734	1.873931	-1.038231
H	5.063961	1.960813	1.310041

48
TS-rotation-tropylium_d
Eopt -877.193514

C	-0.178673	1.012070	-0.867464
C	1.306187	0.408995	-0.060464
C	0.482366	-0.206337	-1.282948
H	1.146896	-0.184452	-2.148136
H	-0.055731	-1.139294	-1.162451
C	0.944230	1.882988	-0.598801
H	1.665898	2.093108	-1.389918
H	0.794513	2.706173	0.094305
H	0.892688	0.168340	0.918347
O	-1.748984	0.101000	0.989625
O	-2.643670	0.630987	-1.104556
C	-2.756477	-0.864290	0.712429
C	-3.586647	-0.215062	-0.461469
C	-3.563925	-1.103315	1.990091
H	-4.416704	-1.767634	1.803112
H	-2.925280	-1.579220	2.743945
H	-3.936717	-0.165581	2.410835
C	-2.102887	-2.195993	0.317686
H	-1.339945	-2.450644	1.062383
H	-2.836066	-3.010934	0.288058
H	-1.623227	-2.150371	-0.662666
C	-4.124715	-1.217732	-1.480880
H	-4.782175	-1.953981	-1.002460
H	-4.709581	-0.688750	-2.242507
H	-3.317084	-1.750488	-1.990452
C	-4.768288	0.629960	0.042240
H	-4.469361	1.334298	0.822441
H	-5.168065	1.208385	-0.798350
H	-5.574630	0.002062	0.439431
B	-1.677975	1.096176	-0.095789
C	-1.885318	2.624109	0.434221
H	-1.742036	3.375315	-0.359019
H	-2.897864	2.771647	0.831974
H	-1.189878	2.880006	1.249087
C	2.809212	0.110358	-0.134860
C	3.093006	-1.344439	0.134417
C	3.586788	0.911250	0.876256
H	3.172493	0.361470	-1.141221
C	4.312184	-1.870272	-0.101121
H	2.285146	-1.973148	0.505287
C	4.933382	0.967125	0.832187
H	3.038429	1.464275	1.637008
C	5.493089	-1.106298	-0.450403
H	4.444115	-2.946209	0.002041
C	5.770465	0.160991	-0.033663
H	5.448962	1.643652	1.512019
H	6.280771	-1.643418	-0.975991
H	6.761656	0.553960	-0.253444

48
TS-rotation-tropylium_e
Eopt -877.191082

C	-0.184218	-1.260929	0.335601
C	1.316713	-0.425851	-0.239942
C	0.526178	-0.280875	1.135229
H	1.180561	-0.639440	1.930938
H	0.014609	0.643695	1.376811

C	0.923441	-1.980484	-0.258911
H	1.641442	-2.483161	0.392022
H	0.749470	-2.498234	-1.198644
H	0.862454	0.147626	-1.046832
O	-1.793630	0.267305	-0.999578
O	-2.635397	-0.991126	0.783320
C	-2.766116	1.072465	-0.343775
C	-3.578480	0.041420	0.529464
C	-3.602401	1.786226	-1.407882
H	-4.433678	2.339794	-0.953951
H	-2.973900	2.504980	-1.947307
H	-4.008704	1.082311	-2.139081
C	-2.067443	2.148361	0.499328
H	-1.316549	2.652188	-0.120238
H	-2.777855	2.903534	0.856407
H	-1.565231	1.730388	1.374585
C	-4.072035	0.598182	1.863763
H	-4.723560	1.467504	1.712196
H	-4.651641	-0.169089	2.390415
H	-3.241965	0.893110	2.511653
C	-4.787306	-0.545808	-0.217287
H	-4.521052	-0.906739	-1.213809
H	-5.174747	-1.395218	0.356579
H	-5.592817	0.190342	-0.324627
B	-1.707868	-1.057546	-0.358085
C	-1.953351	-2.283645	-1.403514
H	-1.801519	-3.273550	-0.943901
H	-2.977958	-2.270141	-1.797641
H	-1.282512	-2.227383	-2.275226
C	2.830037	-0.138332	-0.207594
C	3.642732	-0.840942	0.841881
C	3.080195	1.337796	-0.033370
H	3.210237	-0.441250	-1.194395
C	4.989939	-0.867642	0.769036
H	3.147566	-1.347193	1.668326
C	4.278594	1.880400	-0.330382
H	2.262027	1.966479	0.314313
C	5.788283	-0.105530	-0.168585
H	5.536626	-1.476916	1.487035
C	5.470348	1.125668	-0.659166
H	4.382909	2.963740	-0.298815
H	6.784257	-0.488769	-0.383670
H	6.232434	1.646801	-1.236019

48

TS-rotation-tropylium_f

Eopt -877.190928

C	-0.184591	0.977732	-0.711539
H	-6.584885	-0.705288	-1.746037
C	1.312240	0.272316	0.041008
C	0.474085	-0.207940	-1.223663
H	1.124633	-0.106779	-2.094566
H	-0.070053	-1.145260	-1.195697
C	0.962544	1.788016	-0.349334
H	1.667560	2.082748	-1.128241
H	0.825545	2.544566	0.418817
H	0.870085	-0.038873	0.986457
O	-1.822197	-0.027326	1.029033
O	-2.652426	0.683617	-1.039040
C	-2.837170	-0.948942	0.647389
C	-3.628533	-0.187118	-0.483530
C	-3.677393	-1.283269	1.881583
H	-4.538554	-1.909613	1.617712
H	-3.065653	-1.838488	2.602769
H	-4.041101	-0.379223	2.377282
C	-2.195881	-2.251978	0.151018
H	-1.452061	-2.583929	0.884363
H	-2.940979	-3.048382	0.036316
H	-1.696524	-2.128235	-0.812469
C	-4.170809	-1.090118	-1.590105
H	-4.852860	-1.846976	-1.183691
H	-4.730440	-0.487264	-2.314974
H	-3.366352	-1.598656	-2.128584
C	-4.798328	0.644583	0.067667
H	-4.498532	1.269634	0.912461
H	-5.163711	1.304704	-0.727146
H	-5.629637	0.007530	0.392265
B	-1.700335	1.044234	0.024150
C	-1.880824	2.534473	0.660391
H	-1.694720	3.336577	-0.072029
H	-2.899878	2.682466	1.040883
H	-1.201430	2.710491	1.509127
C	2.806704	-0.103103	0.032789
C	3.534416	0.575150	1.165295
C	3.581176	0.192149	-1.219943
H	2.835341	-1.189695	0.203098
C	4.740056	0.136743	1.580966
H	3.055401	1.417301	1.661878
C	4.800492	-0.348547	-1.423851
H	3.154086	0.837458	-1.985749
C	5.532243	-0.871994	0.907701
H	5.178172	0.583246	2.472193
C	5.558823	-1.089723	-0.437234
H	5.280379	-0.197416	-2.389470
H	6.263962	-1.401445	1.515497
H	6.310494	-1.778577	-0.818826

4. REFERENCE

[1] A. F. Burchat, J. M. Chong, N. Nielsen, *J. Organomet. Chem.* **1997**, *542*, 281-283.

[2] J. Štambaský, A. V. Malkov, R. Kočovský, *J. Org. Chem.* **2008**, *73*, 9148-9150.

[3] C. R. Davis, I. K. Luvaga, J. M. Ready, *J. Am. Chem. Soc.* **2021**, *143*, 4921-4927.

[4] S. H. Bennett, A. Fawcett, E. Denton, T. Biberger, V. Fasano, N. Winter, V. K. Aggarwal, *J. Am. Chem. Soc.*, **2020**, *142*, 16766−16775.

[5] Bruker, *SAINT+ v8.39.0 Integration Engine, Data Reduction Software, Bruker Analytical X-ray Instruments Inc., Madison, WI, USA*, 2018.

[6] Bruker, *SADABS 2018, Bruker AXS area detector scaling and absorption correction, Bruker Analytical X-ray Instruments Inc., Madison, Wisconsin, USA,* **2018**.

[7] G. M. Sheldrick, *Acta Crystallographica a-Foundation and Advances*, **2015**, *71*, 3-8.

[8] G. M. Sheldrick, *Acta Crystallogr., Sect. A: Found. Crystallogr.*, **2008**, *64*, 112-122.

[9] G. M. Sheldrick, *Acta Crystallogr. C*, **2015**, *71*, 3-8.

[10] O. V. Dolomanov, L. J. Bourhis, R. J. Gildea, J. A. K. Howard and H. Puschmann, *J. Appl. Crystallogr.*, **2009**, *42*, 339-341.

[11] a) C. Lee, W. Yang, R. G. Parr, *Phys. Rev. B*, **1988**, *37*, 785–789; b) A. D. Becke, *J. Chem. Phys.*, **1993**, *98*, 5648–5652.

[12] P. J. Hay, W. R. Wadt, *J. Chem. Phys.*, **1985**, *82*, 270–283.

[13] a) W. J. Hehre, R. Ditchfield, J. A. Pople, *J. Chem. Phys.* **1972**, *56*, 2257–2261; b) P. C. Hariharan, J. A. Pople, *Theor. Chim. Acta.* **1973**, *28*, 213–222; c) R. Krishnan, J. S. Binkley, R. Seeger, J. A. Pople, *J. Chem. Phys.*, **1980**, *72*, 650–654; d) A. D. McLean, G. S. Chandler, *J. Chem. Phys.* **1980**, *72*, 5639–5648; e) M. M. Francl, W. J. Pietro, W. J. Hehre, J. S. Binkley, M. S. Gordon, D. J. DeFrees, J. A., Pople, *J. Chem. Phys.*, *1982*, *77*, 3654–3665.

[14] Y. Zhao, D. G. Truhlar, *Theor. Chem. Acc.* **2008**, *120*, 215–241.

[15] L. E. Roy, P. J. Hay, R. L. Martin, *J. Chem. Theory Comput.*, **2008**, *4*, 1029–1031.

[16] A. V. Marenich, C. J. Cramer, D. G. Truhlar, *J. Phys. Chem. B*, **2009**, *113*, 6378–6396.

[17] C. R. Davis, Y. Fu, P. Liu, J. M. Ready, *J. Am. Chem. Soc.*, **2022**, *144*, 16118–16130.

[18] J.-D. Chai, M. Head-Gordon, *Phys. Chem. Chem. Phys.*, **2008**, *10*, 6615–5520.

[19] a) F. Weigend, R. Ahlrichs. *Phys. Chem. Chem. Phys.* **2005**, *7*, 3297–3305; b) F. Weigend *Phys. Chem. Chem. Phys.* **2006**, *8*, 1057–1065.

[20] K. Fukui, *Acc. Chem. Res.* **1981**, *14*, 363−368.

[21] Gaussian 16, Revision C.01, M. J. Frisch, G. W. Trucks, H. B. Schlegel, G. E. Scuseria, M. A. Robb, J. R. Cheeseman, G. Scalmani, V. Barone, G. A. Petersson, H. Nakatsuji, X. Li, M. Caricato, A. V. Marenich, J. Bloino, B. G. Janesko, R. Gomperts, B. Mennucci, H. P. Hratchian, J. V. Ortiz, A. F. Izmaylov, J. L. Sonnenberg, D. Williams-Young, F. Ding, F. Lipparini, F. Egidi, J. Goings, B. Peng, A. Petrone, T. Henderson, D. Ranasinghe, V. G. Zakrzewski, J. Gao, N. Rega, G. Zheng, W. Liang, M. Hada, M. Ehara, K. Toyota, R. Fukuda, J. Hasegawa, M. Ishida, T. Nakajima, Y. Honda, O. Kitao, H. Nakai, T. Vreven, K. Throssell, J. A. Jr. Montgomery, J. E. Peralta, F. Ogliaro, M. J. Bearpark, J. J. Heyd, E. N. Brothers, K. N. Kudin, V. N. Staroverov, T. A. Keith, R. Kobayashi, J. Normand, K. Raghavachari, A. P. Rendell, J. C. Burant, S. S. Iyengar, J. Tomasi, M. Cossi, J. M. Millam, M. Klene, C. Adamo, R. Cammi, J. W. Ochterski, R. L. Martin, K. Morokuma, O. Farkas, J. B. Foresman, D. J. Fox, Gaussian, Inc., Wallingford CT, **2016**.

[22] AQME, v1.4, J. V. Alegre-Requena, S. Sowndarya, T. Alturaifi, R. Perez-Soto, R. S. Paton, WIREs Comput. Mol. Sci. **2023**, DOI: 10.1002/wcms.1663 17590884

[23] The PyMOL Molecular Graphics System, version 2.0.7, Schrodinger, LLC.

[24] S. Grimme, *Chem. Eur. J.* **2012**, *18*, 9955−9964.

[25] G. Luchini, J. V. Alegre-Requena, I. Funes-Ardoiz, R. S. Paton, *F1000Research*, **2020**, *9*, 291.

[26] V. S. Bryantsev, M. S. Diallo, W. A. Goddard III, *J. Phys. Chem. B*, **2008**, *112*, 9709–9719.

[27] J. Contreras-Garcia, E. R. Johnson, S. Keinan, R. Chaudret, J.-P. Piquemal, D. N. Beratan, W. Yang, *J. Chem. Theory Comput.*, **2011**, *7*, 625–632.

5. SPECTRA

5.1 NMR Spectra

Compound 5

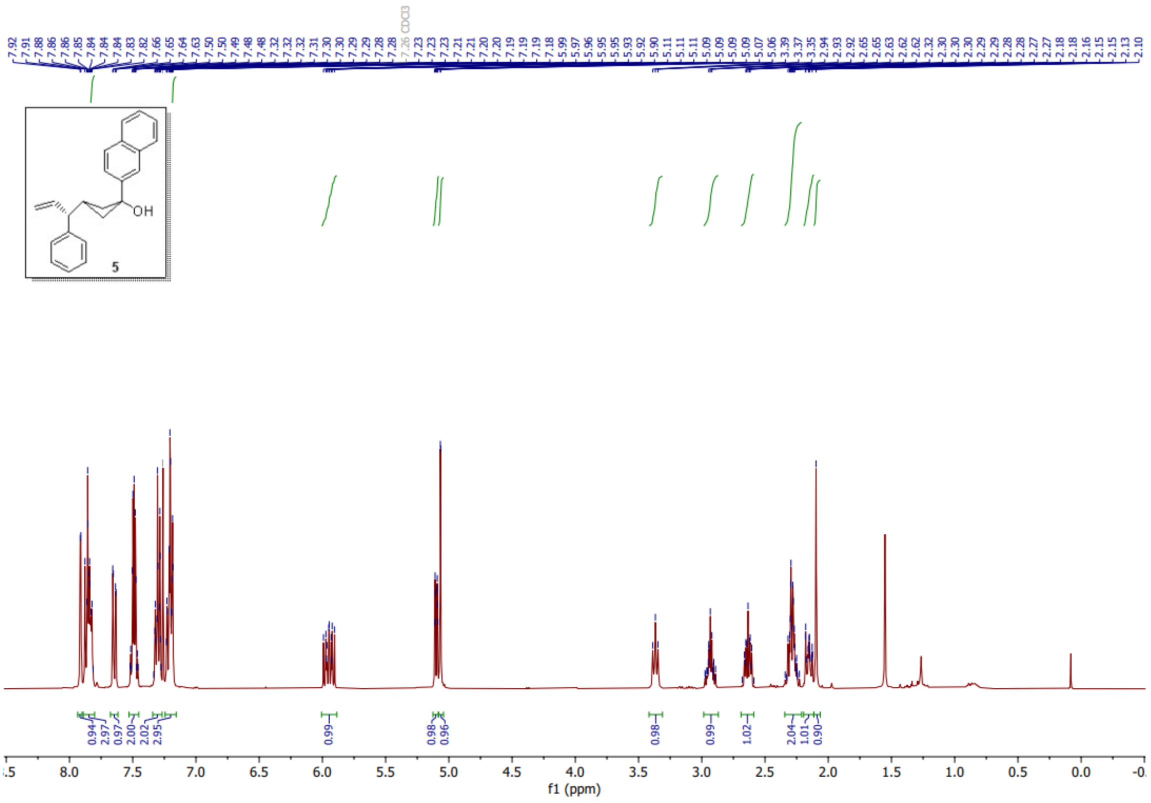

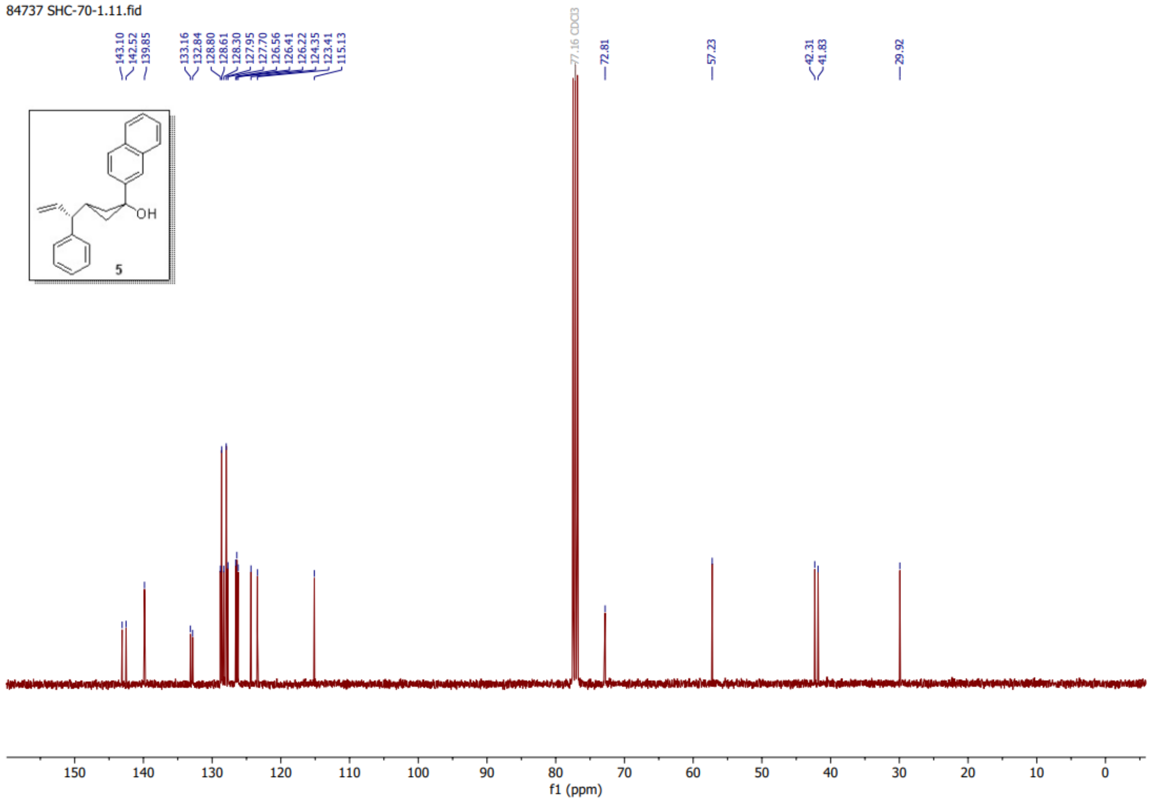

Compound 6

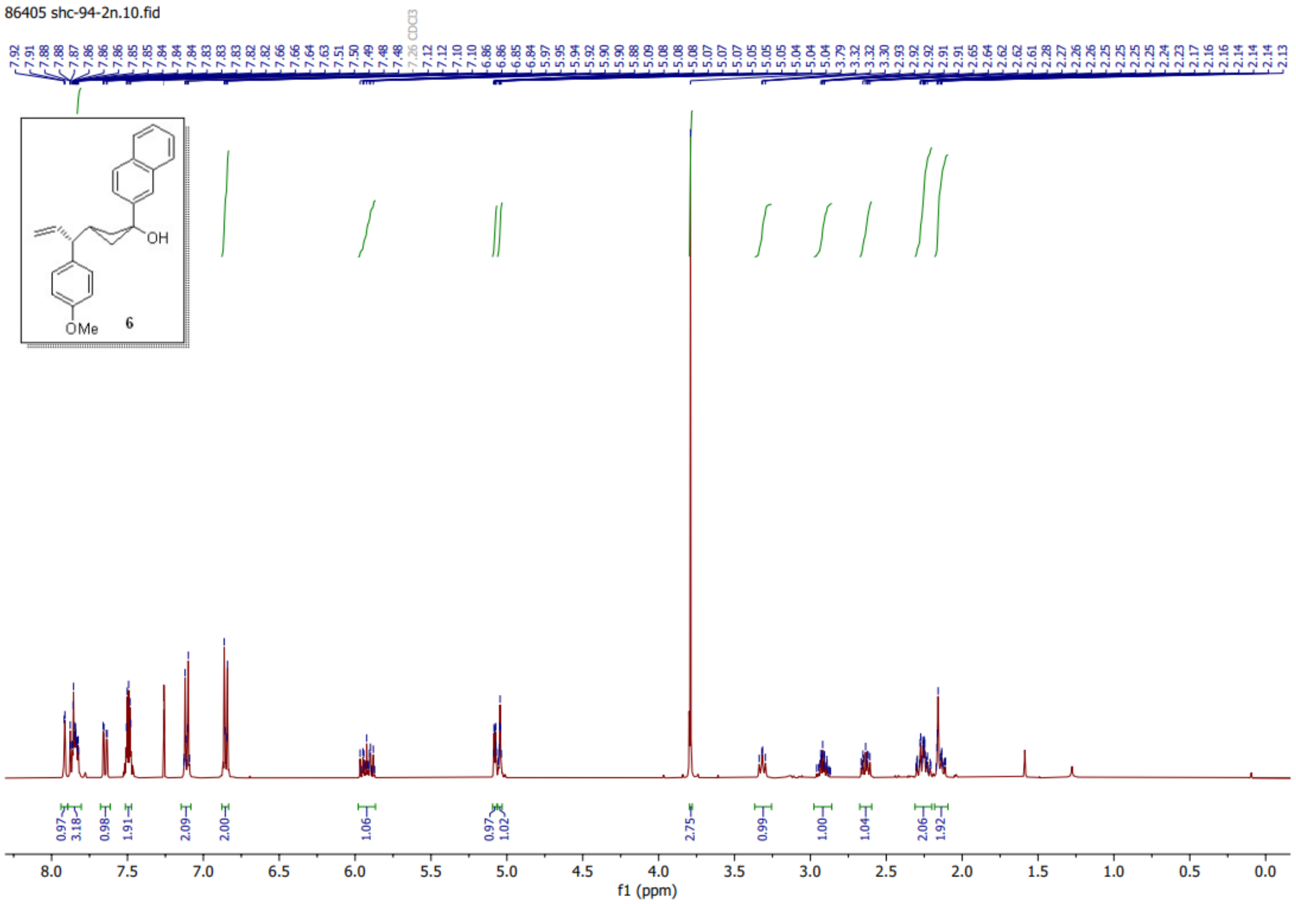

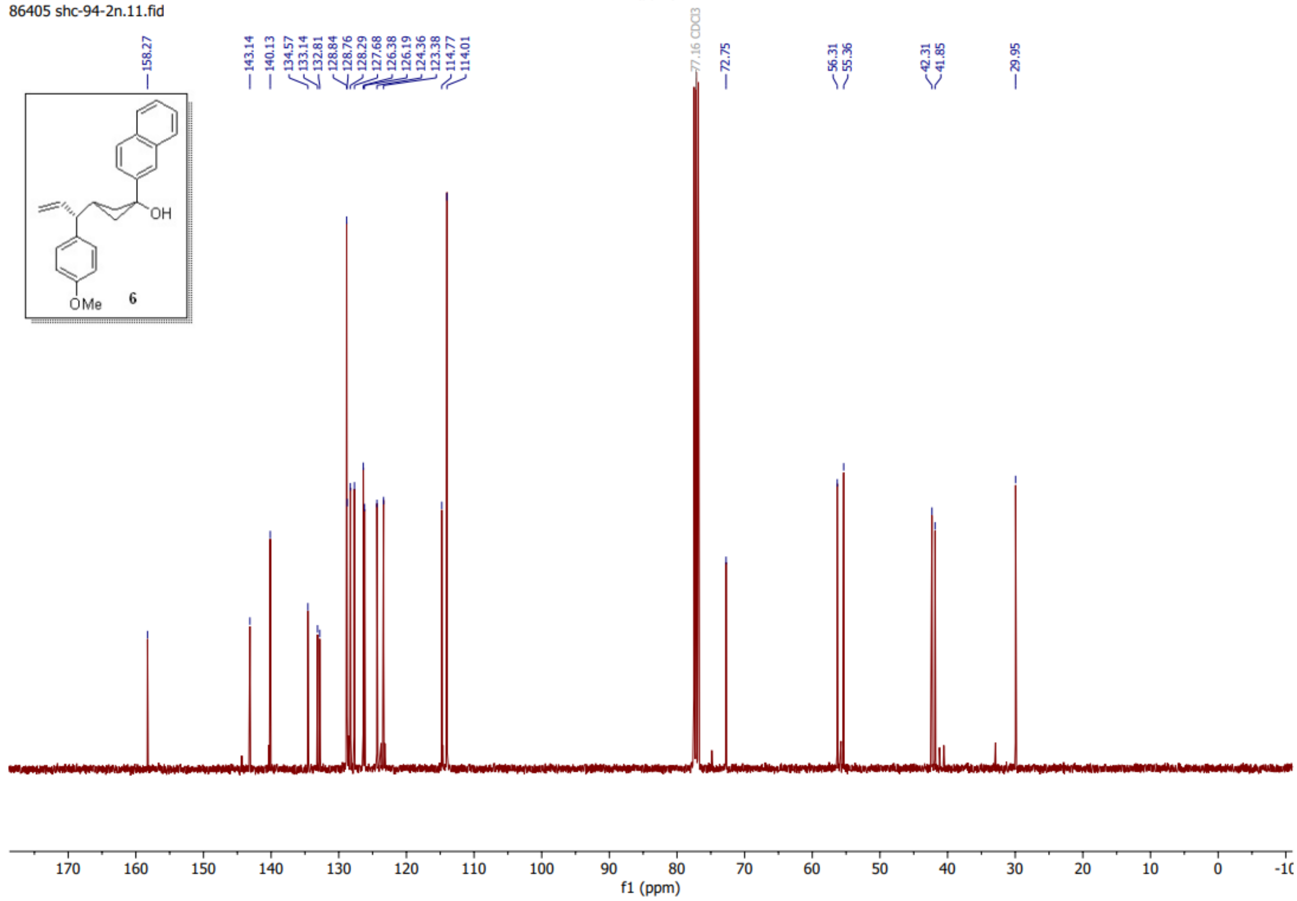

Compound 7

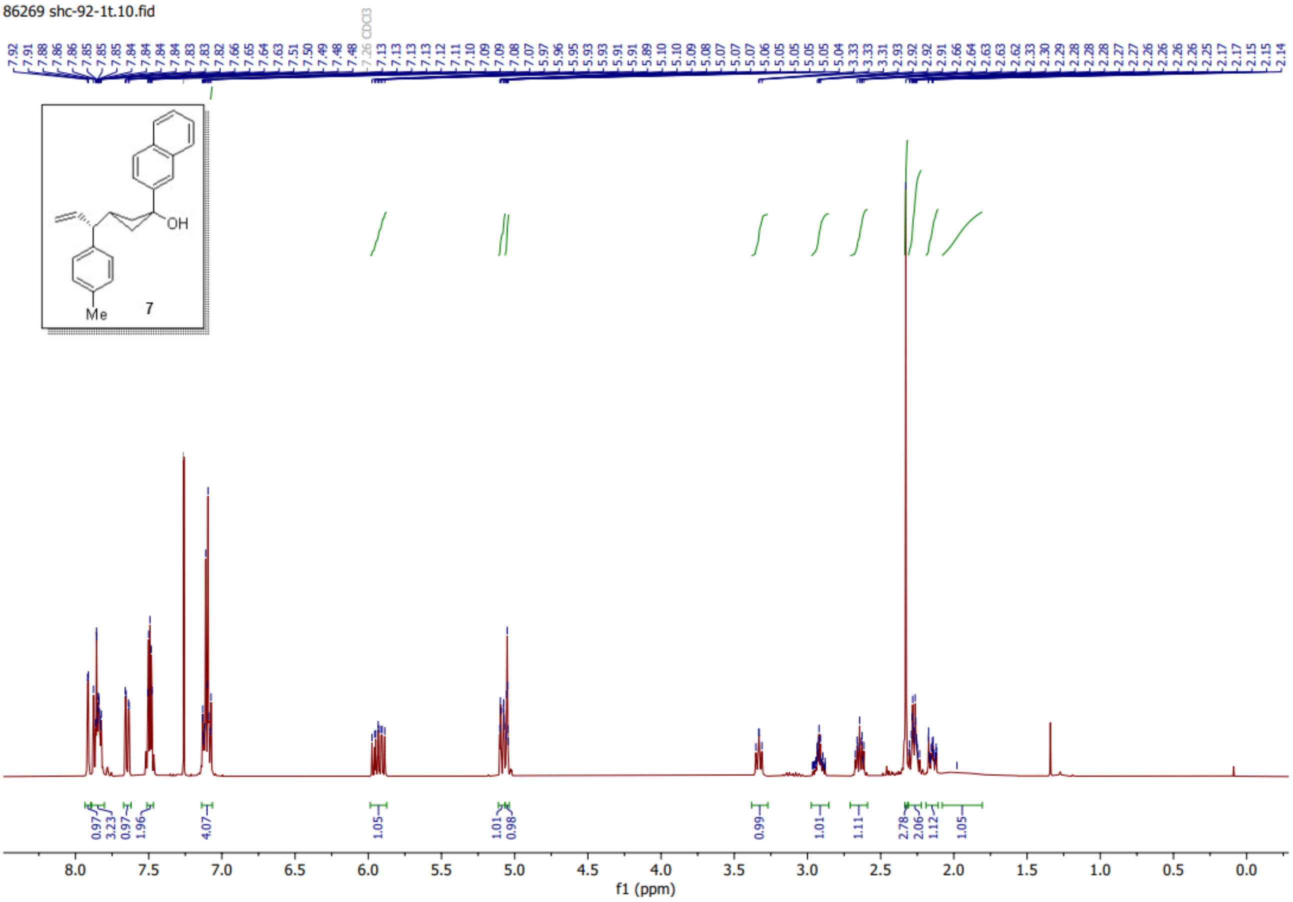
86269 shc-92-1t.10.fid
OH
Me
7
f1 (ppm)

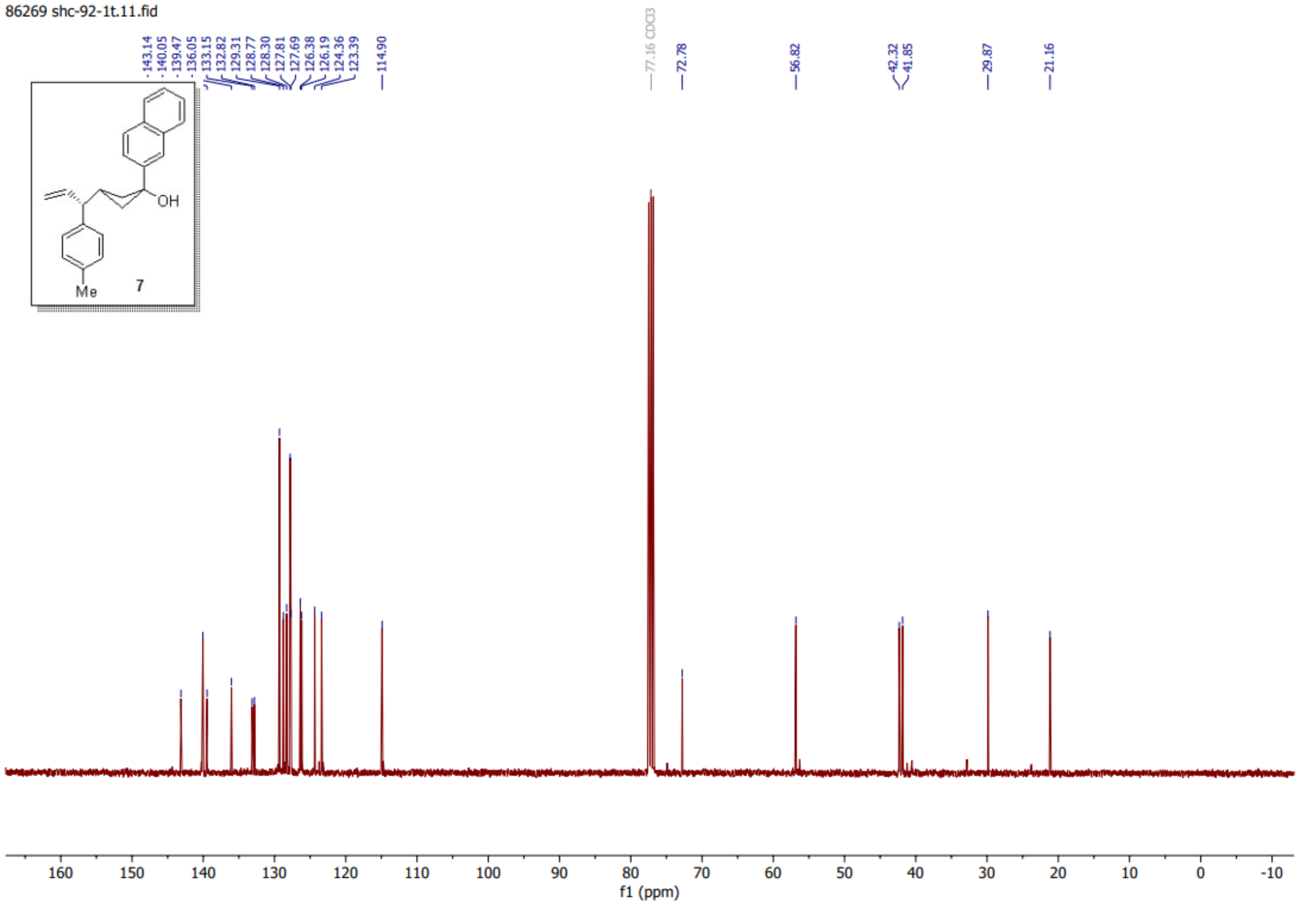
86269 shc-92-1t.11.fid
143.14
140.05
139.47
136.05
133.15
132.82
129.31
128.77
128.30
127.81
127.69
126.38
126.19
124.36
123.39
114.90
77.16 CDCl3
72.78
56.82
42.32
41.85
29.87
21.16
OH
Me
7
f1 (ppm)

Compound 8

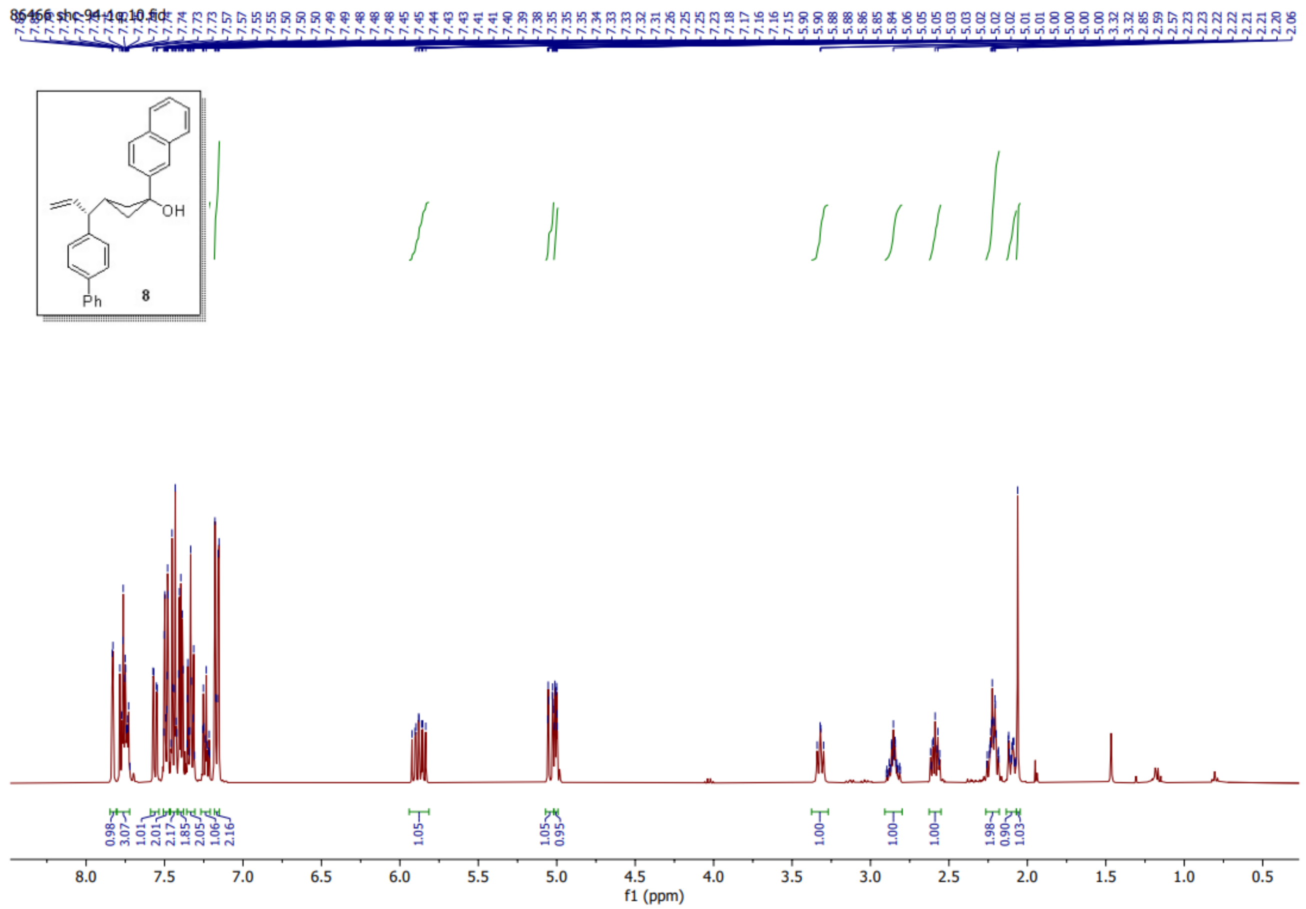
86466 shc-94-1q.10.fid
OH
Ph
8
0.98
3.07
1.01
2.01
2.17
1.85
2.05
1.06
2.16
1.05
1.05
0.95
1.00
1.00
1.00
1.98
0.90
1.03
8.0
7.5
7.0
6.5
6.0
5.5
5.0
4.5
4.0
3.5
3.0
2.5
2.0
1.5
1.0
0.5
f1 (ppm)

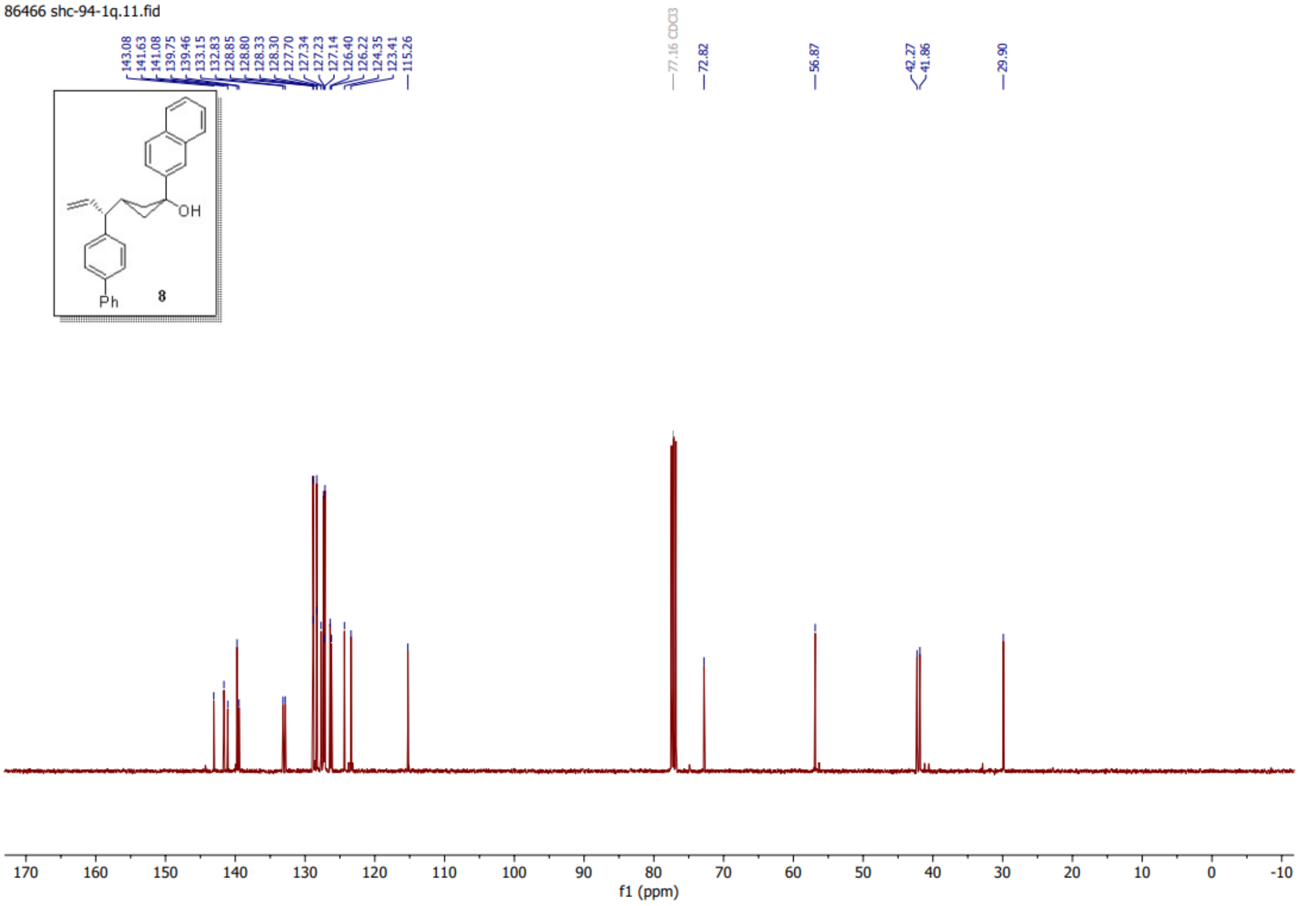
86466 shc-94-1q.11.fid
143.08
141.63
141.08
139.75
139.46
133.15
132.83
128.85
128.80
128.33
128.30
127.70
127.34
127.23
127.14
126.40
126.22
124.35
123.41
115.26
77.16 CDCl3
72.82
56.87
42.27
41.86
29.90
OH
Ph
8
170
160
150
140
130
120
110
100
90
80
70
60
50
40
30
20
10
0
-10
f1 (ppm)

Compound 9

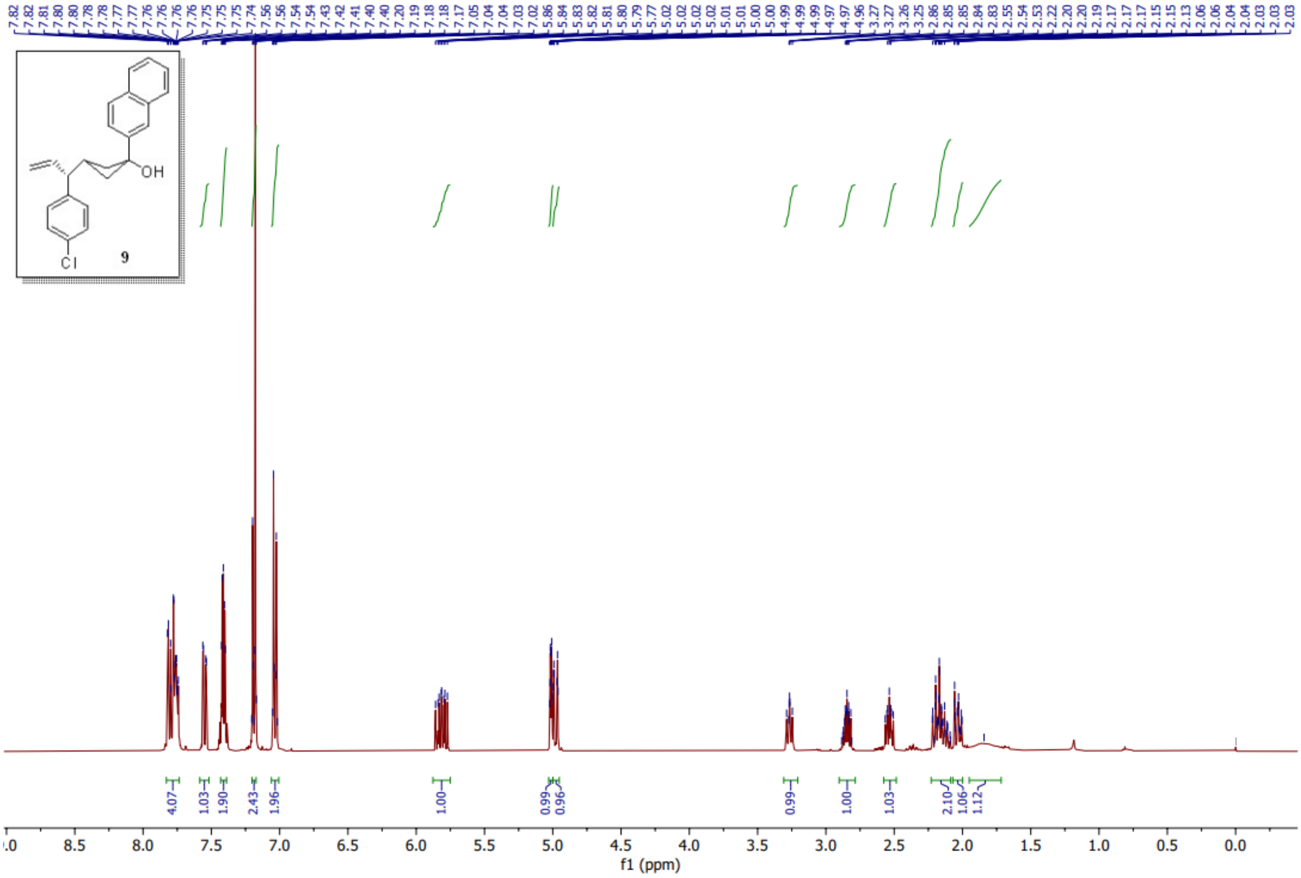

86270 shc-92-2t.11.fid

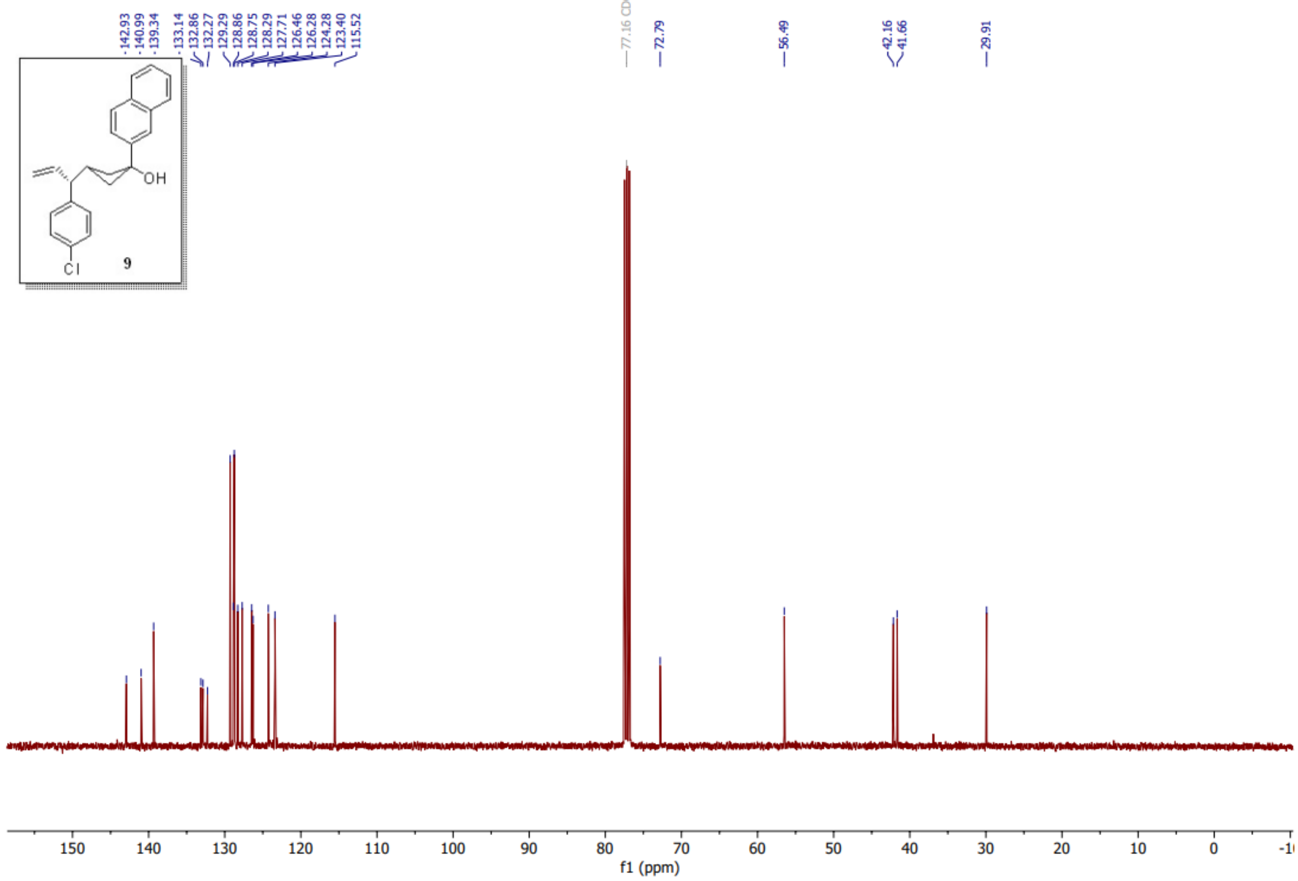

Compound 10

86303 shc-93-3.12.fid

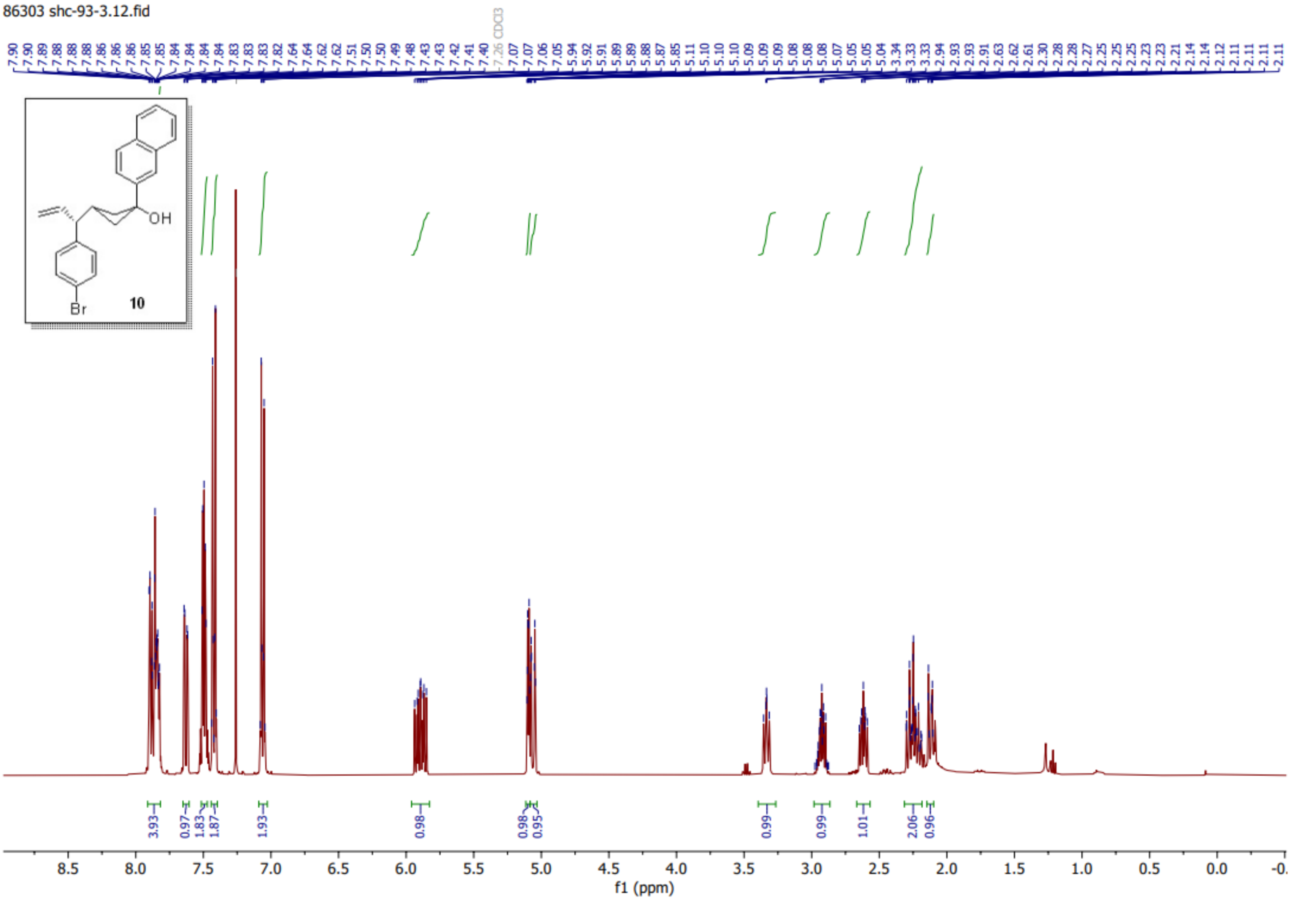

86303 shc-93-3.11.fid

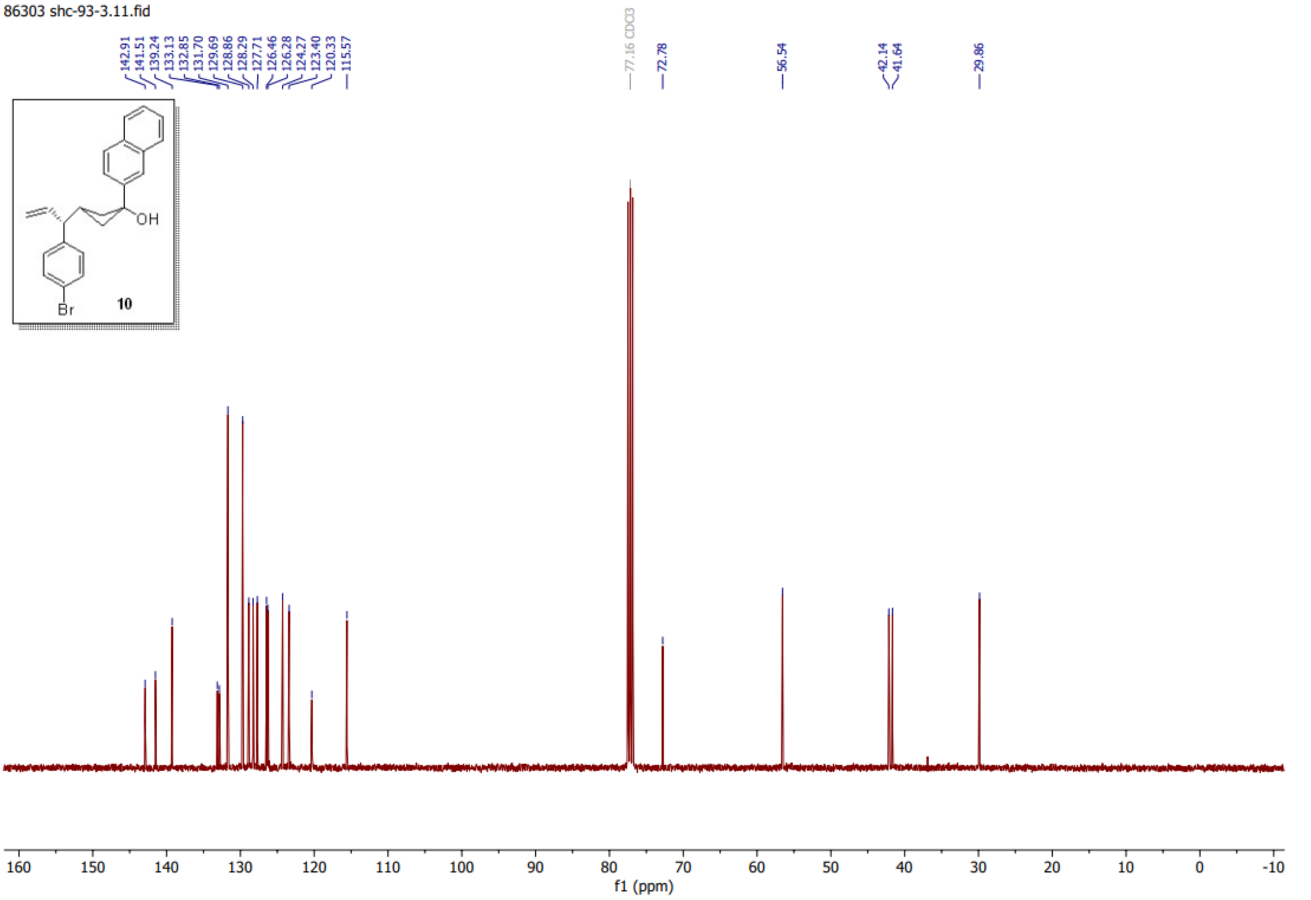

Compound 11

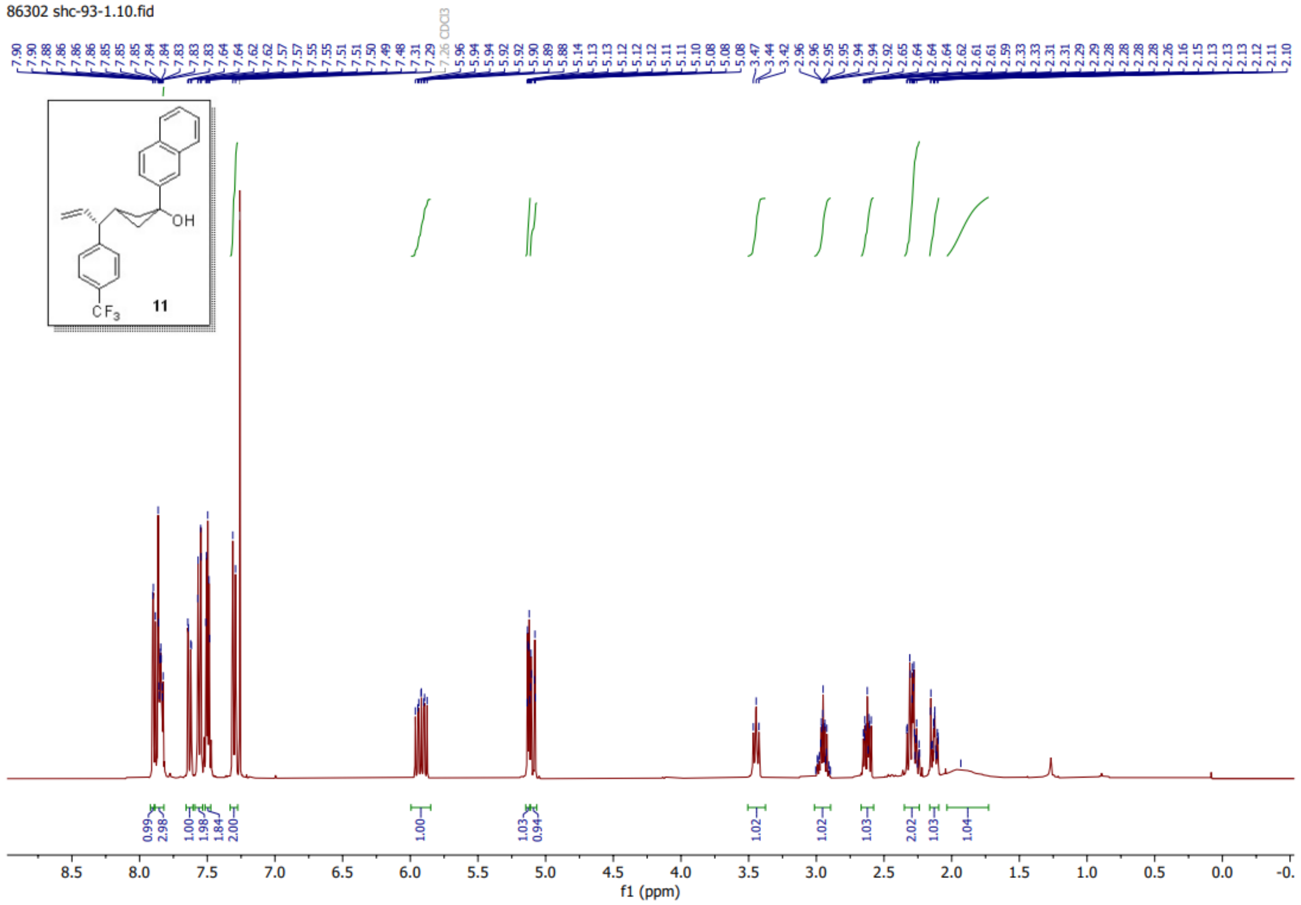
86302 shc-93-1.10.fid
OH
CF3
11
f1 (ppm)

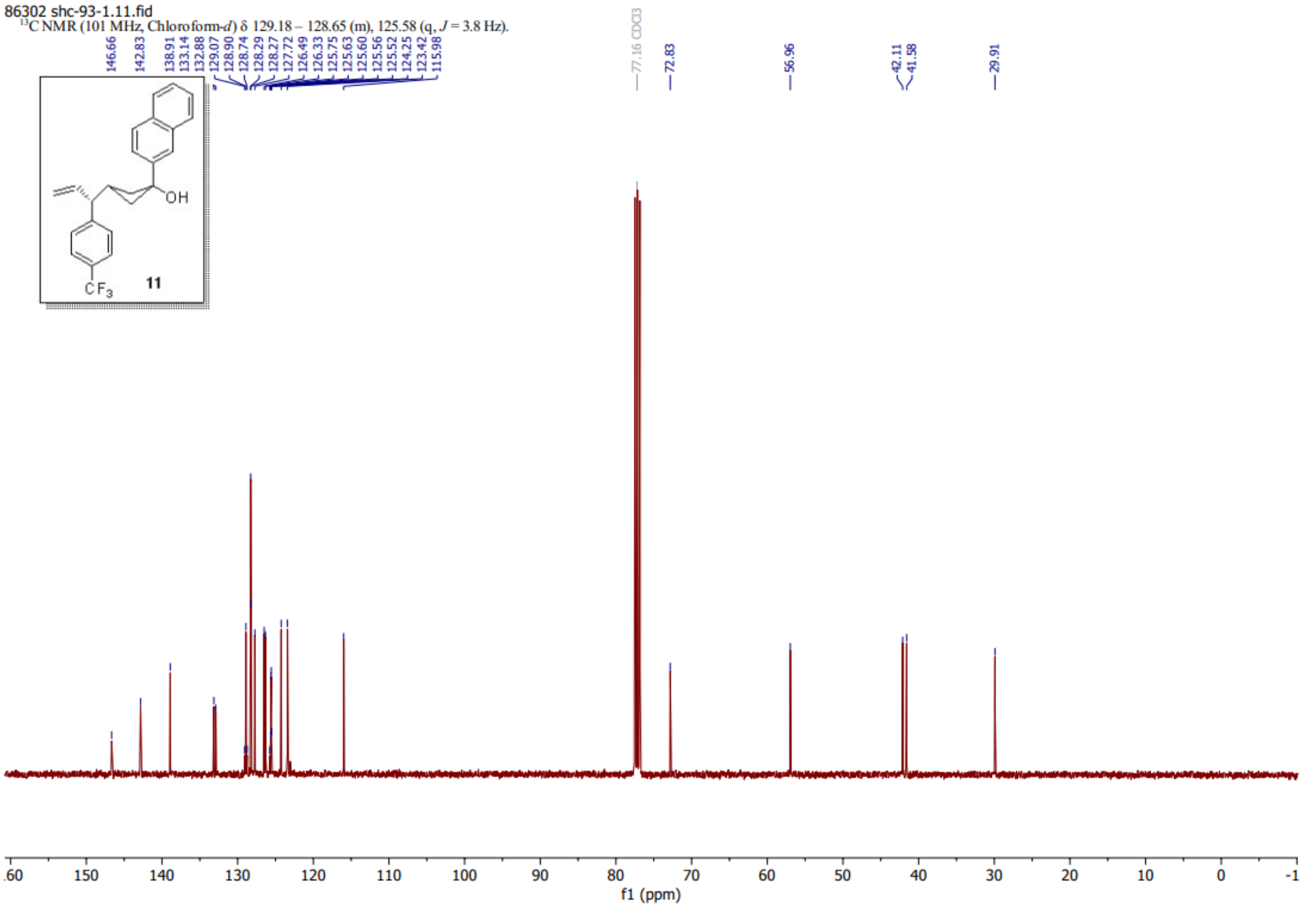
86302 shc-93-1.11.fid
13C NMR (101 MHz, Chloroform-d) δ 129.18 – 128.65 (m), 125.58 (q, J = 3.8 Hz).
OH
CF3
11
f1 (ppm)

Compound 12

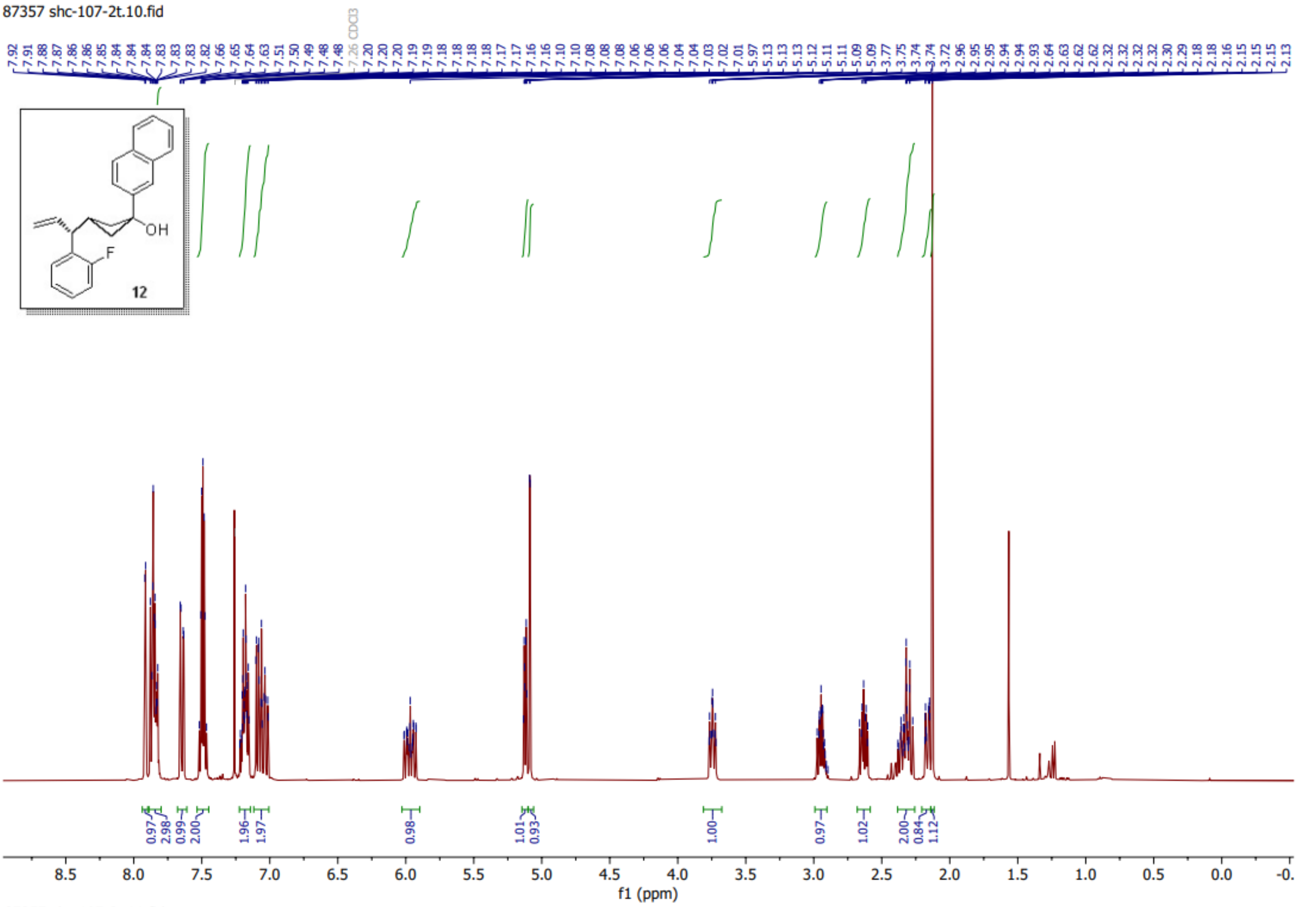
87357 shc-107-2t.10.fid
7.26 CDCl3
OH
F
12
0.97
2.98
0.99
2.00
1.96
1.97
0.98
1.01
0.93
1.00
0.97
1.02
2.00
0.84
1.12
f1 (ppm)

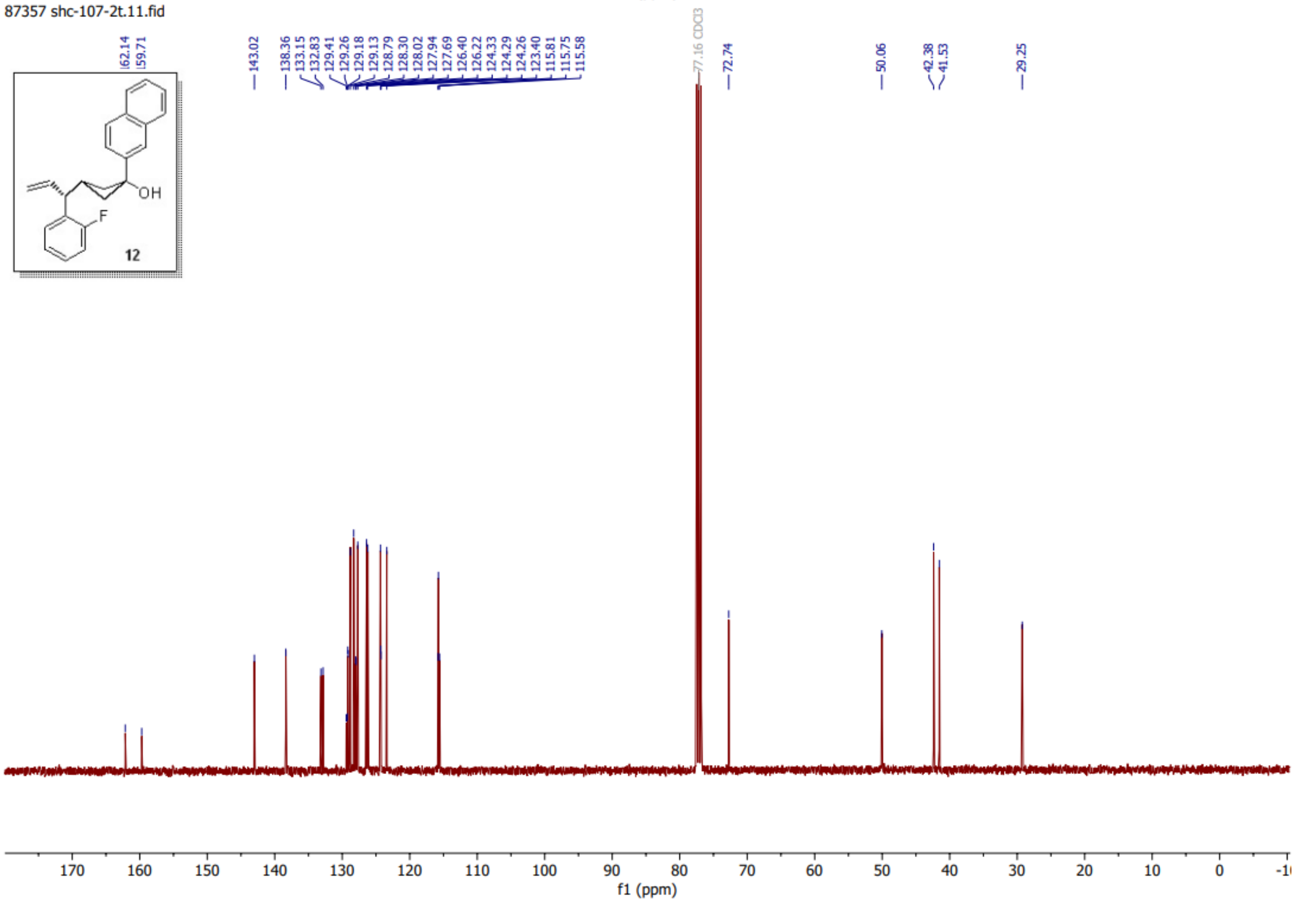
87357 shc-107-2t.11.fid
162.14
159.71
143.02
138.36
133.15
132.83
129.41
129.26
129.18
129.13
128.79
128.30
128.02
127.94
127.69
126.40
126.22
124.33
124.29
124.26
123.40
115.81
115.75
115.58
77.16 CDCl3
72.74
50.06
42.38
41.53
29.25
OH
F
12
f1 (ppm)

Compound 13

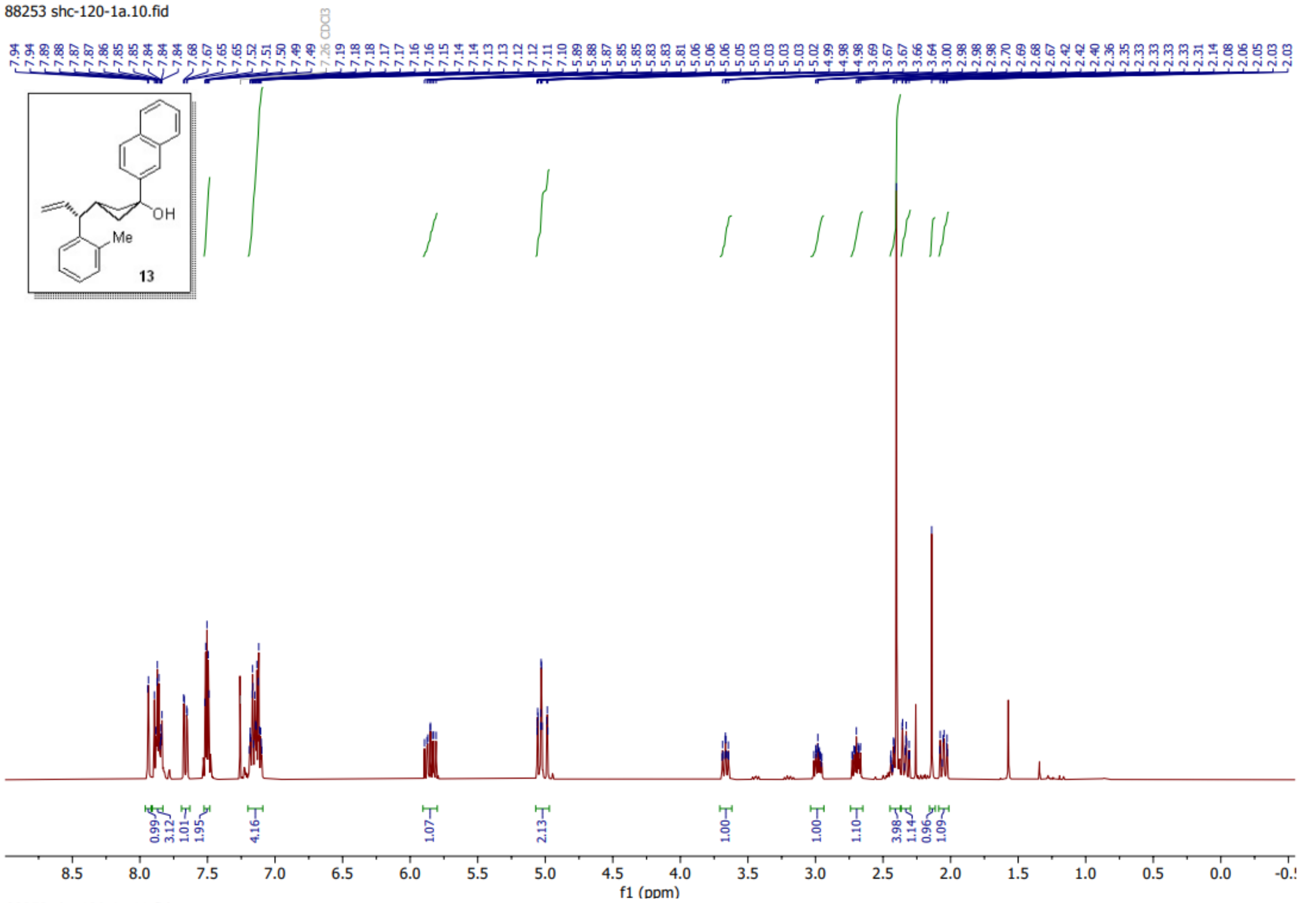

88253 shc-120-1a.10.fid
13
f1 (ppm)

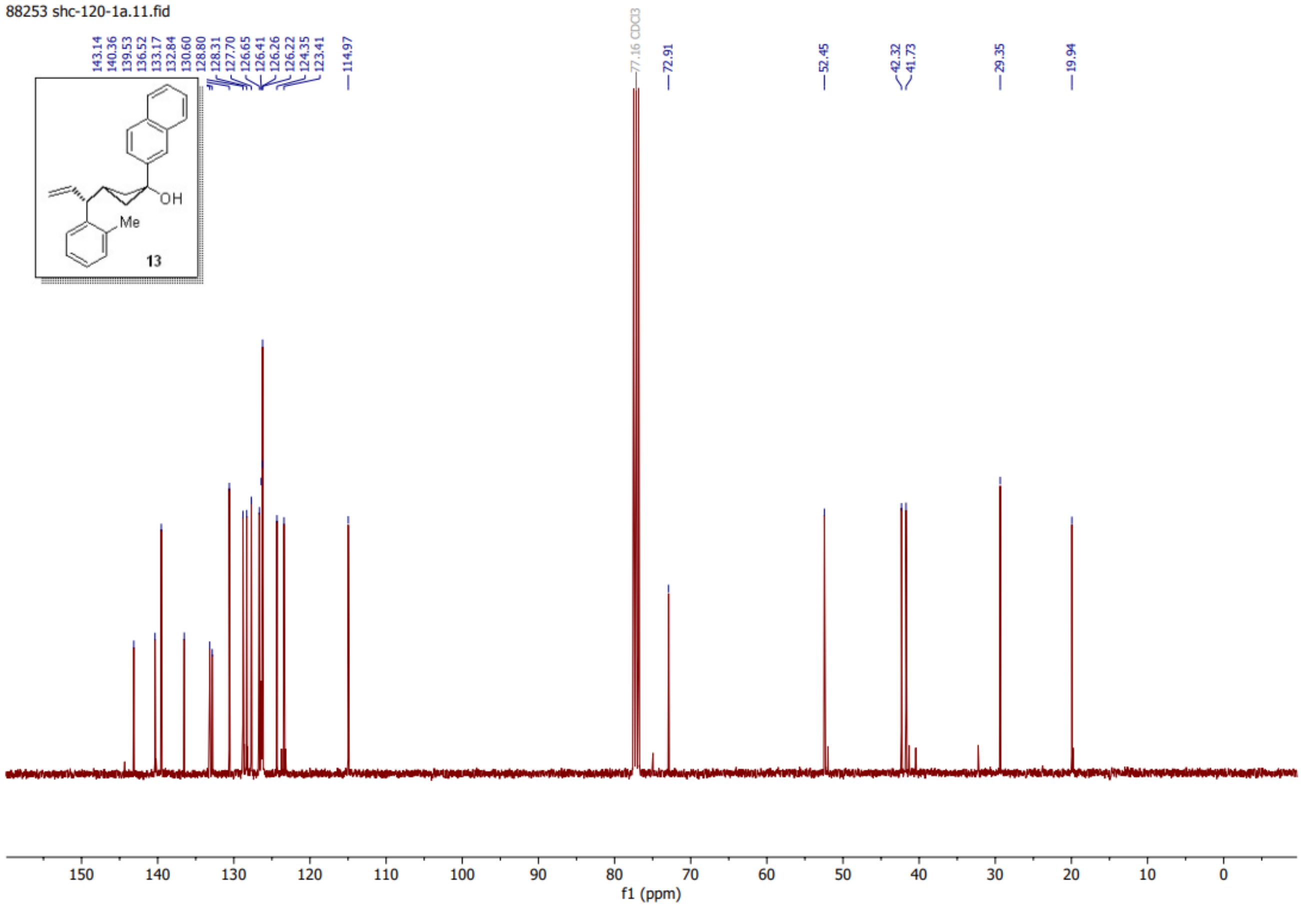

88253 shc-120-1a.11.fid
143.14
140.36
139.53
136.52
133.17
132.84
130.60
128.80
128.31
127.70
126.65
126.41
126.26
126.22
124.35
123.41
114.97
77.16 CDCl3
72.91
52.45
42.32
41.73
29.35
19.94
13
f1 (ppm)

Compound 14

87359 shc-112-1t.10.fid

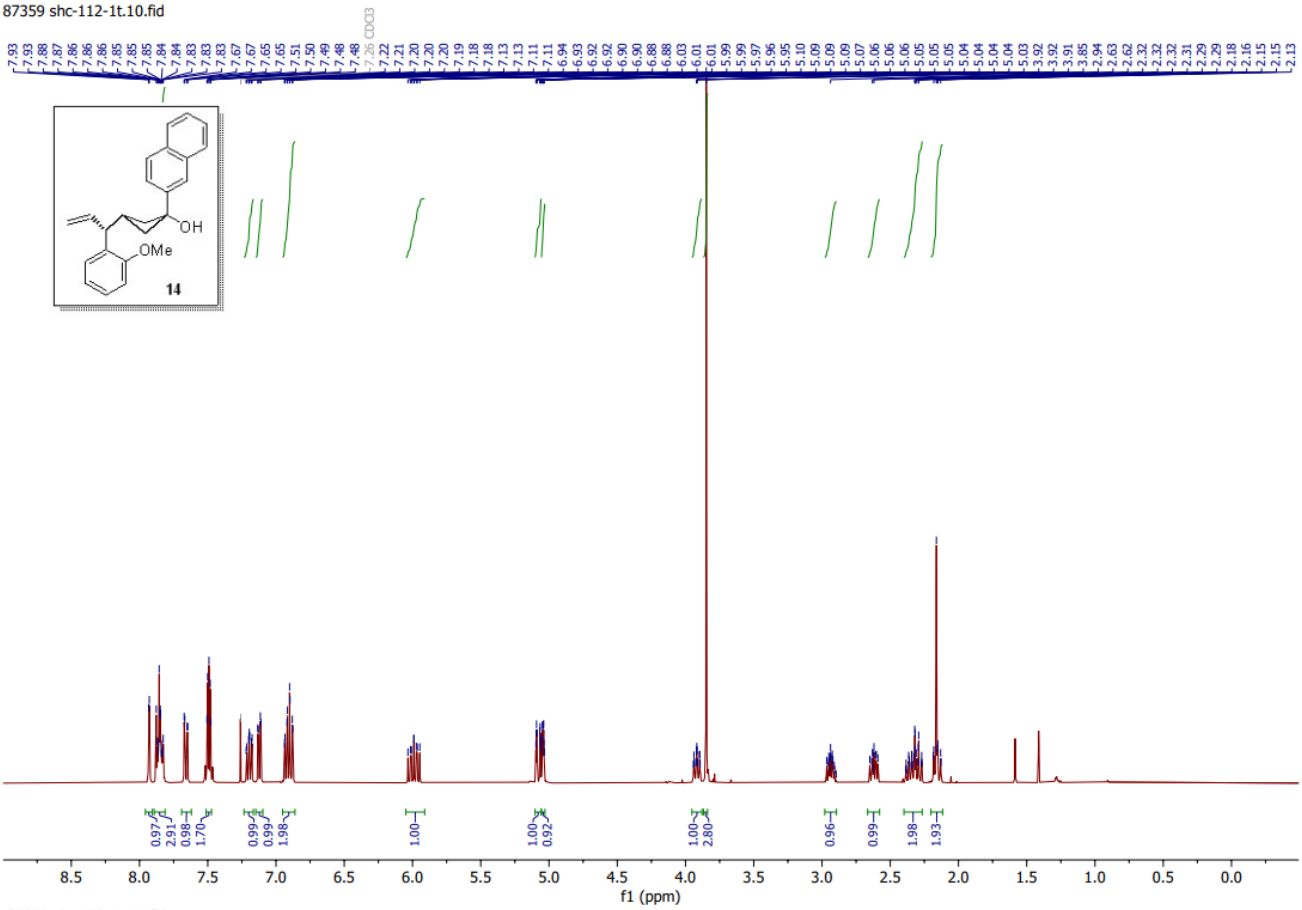

87359 shc-112-1t.11.fid

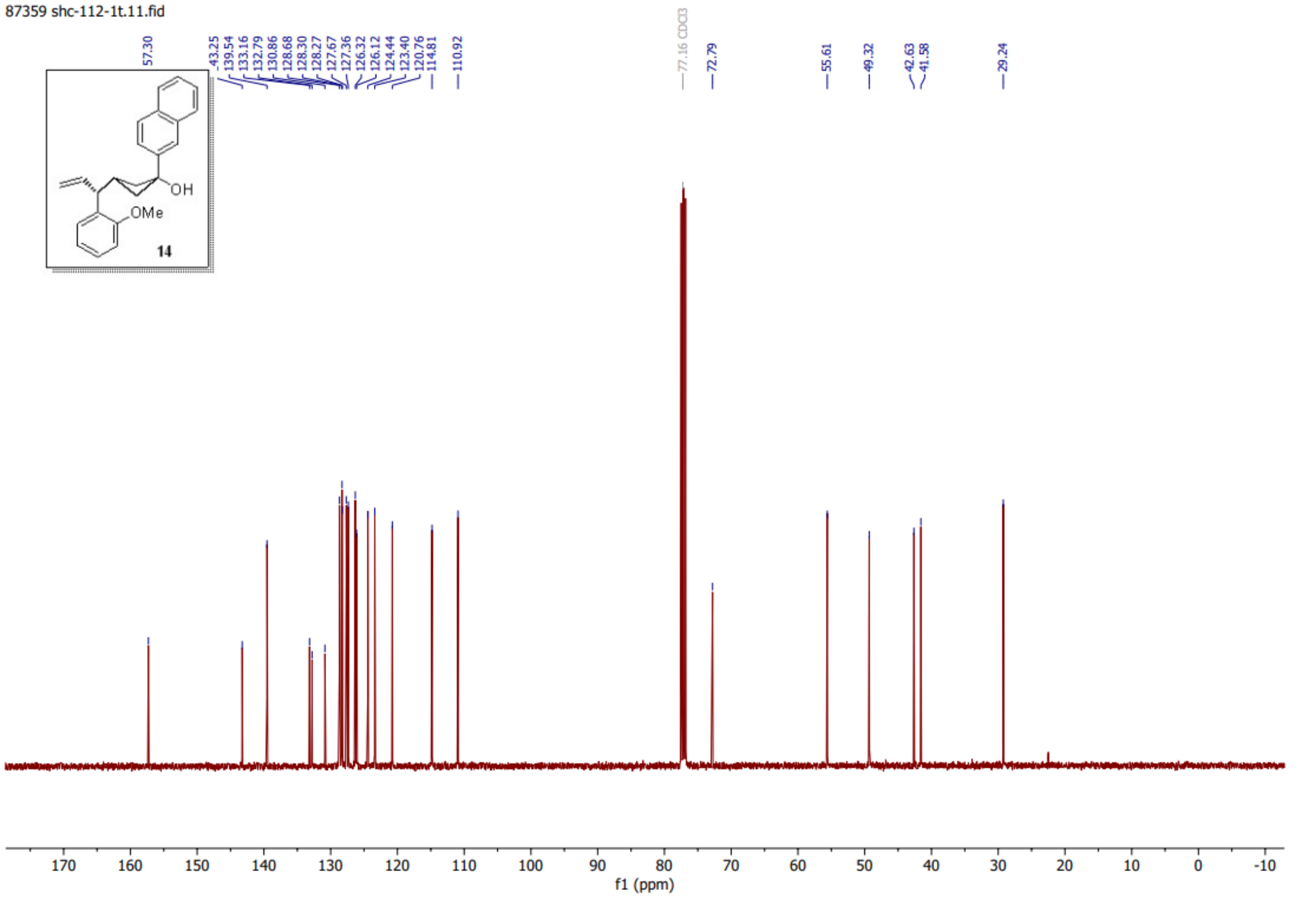

Compound 15

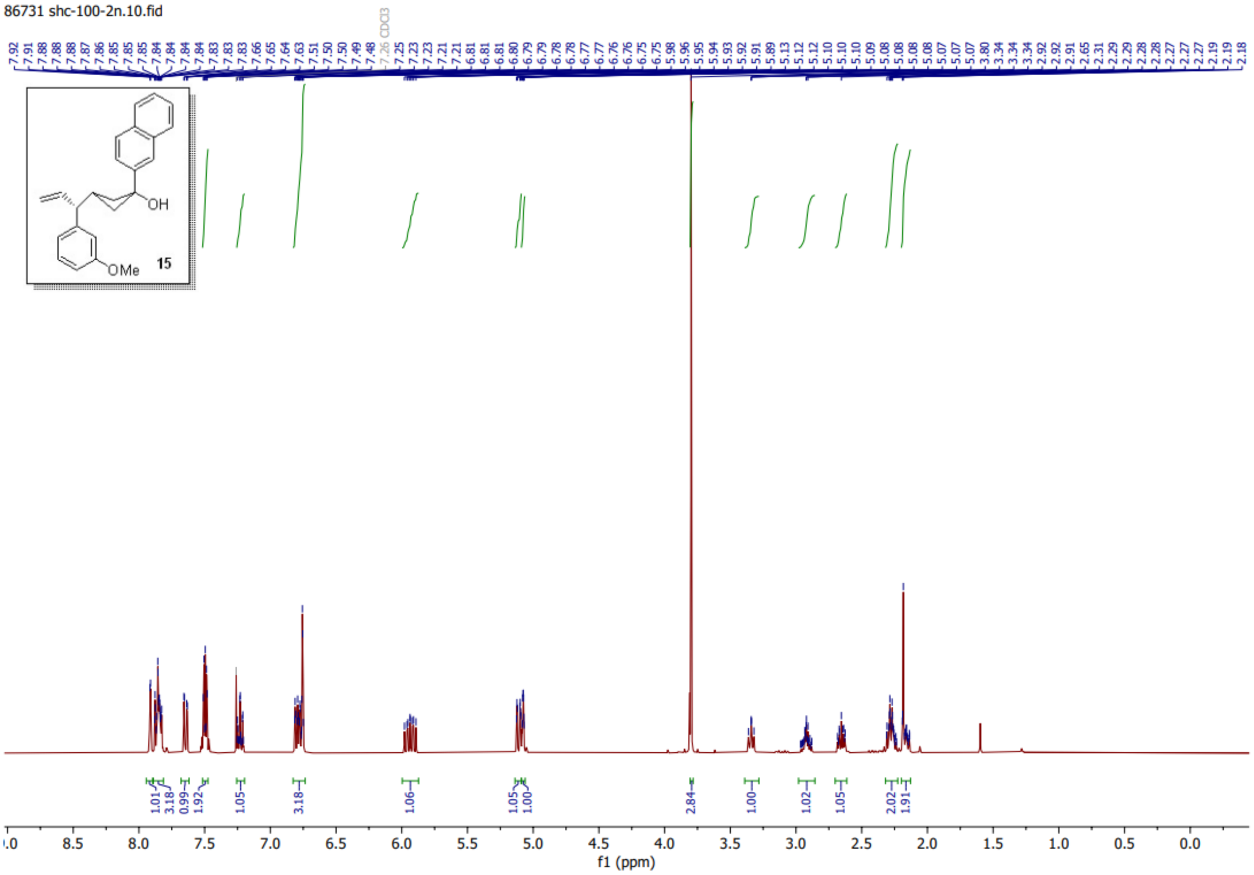
86731 shc-100-2n.10.fid
7.92 7.91 7.88 7.88 7.88 7.87 7.86 7.85 7.85 7.85 7.84 7.84 7.84 7.84 7.83 7.83 7.83 7.66 7.65 7.64 7.63 7.51 7.50 7.50 7.49 7.48 7.26 CDCl3 7.25 7.23 7.23 7.21 7.21 6.81 6.81 6.81 6.80 6.79 6.79 6.78 6.78 6.77 6.77 6.76 6.76 6.75 6.75 5.98 5.96 5.95 5.94 5.93 5.92 5.91 5.89 5.13 5.12 5.12 5.10 5.10 5.09 5.08 5.08 5.08 5.08 5.07 5.07 5.07 3.80 3.34 3.34 3.34 2.92 2.92 2.91 2.65 2.31 2.29 2.29 2.28 2.27 2.27 2.27 2.19 2.18
OH
OMe
15
1.01 3.18 0.99 1.92 1.05 3.18 1.06 1.05 1.00 2.84 1.00 1.02 1.05 2.02 1.91
f1 (ppm)

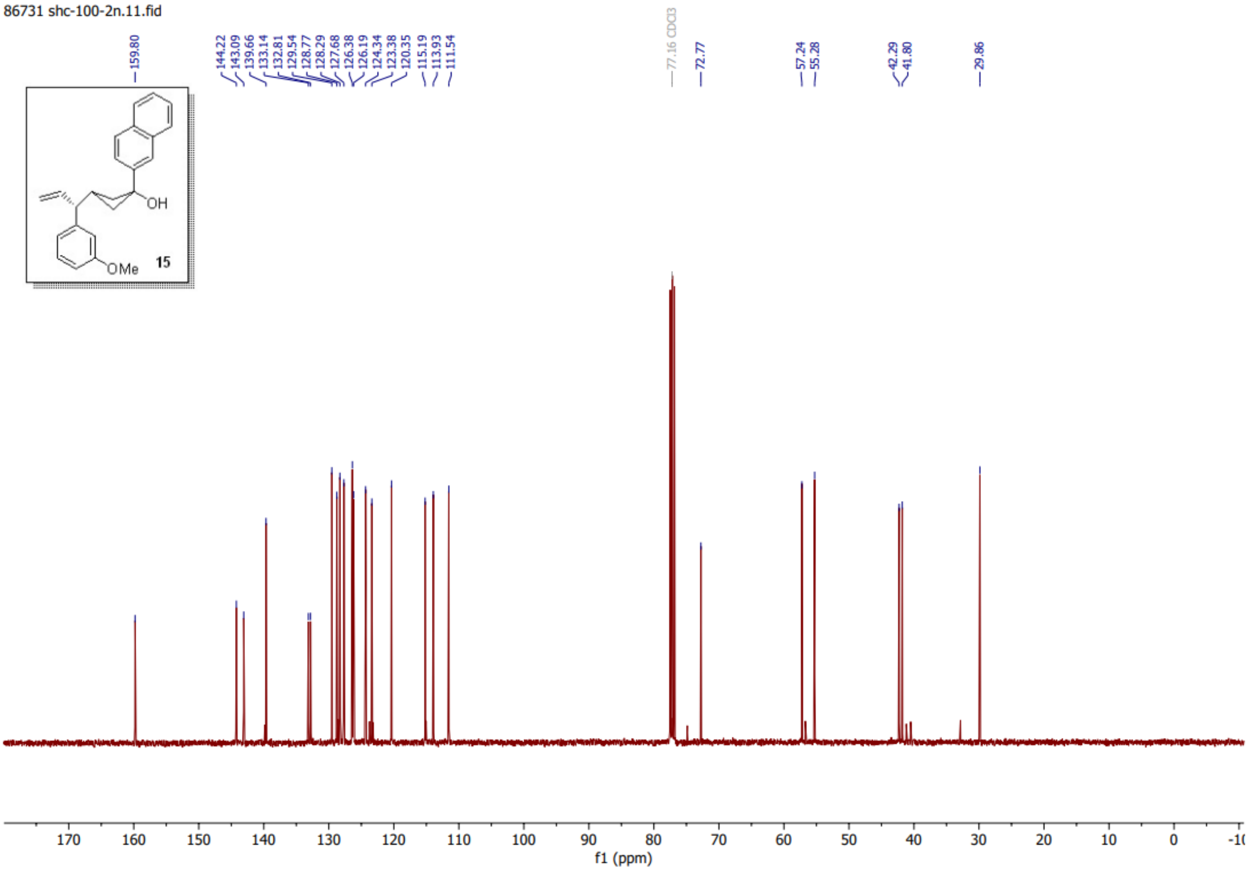
86731 shc-100-2n.11.fid
159.80
144.22 143.09 139.66 133.14 132.81 129.54 128.77 128.29 127.68 126.38 126.19 124.34 123.38 120.35 115.19 113.93 111.54
77.16 CDCl3
72.77
57.24 55.28
42.29 41.80
29.86
OH
OMe
15
f1 (ppm)

Compound 16

86840 shc-106-2.10.fid

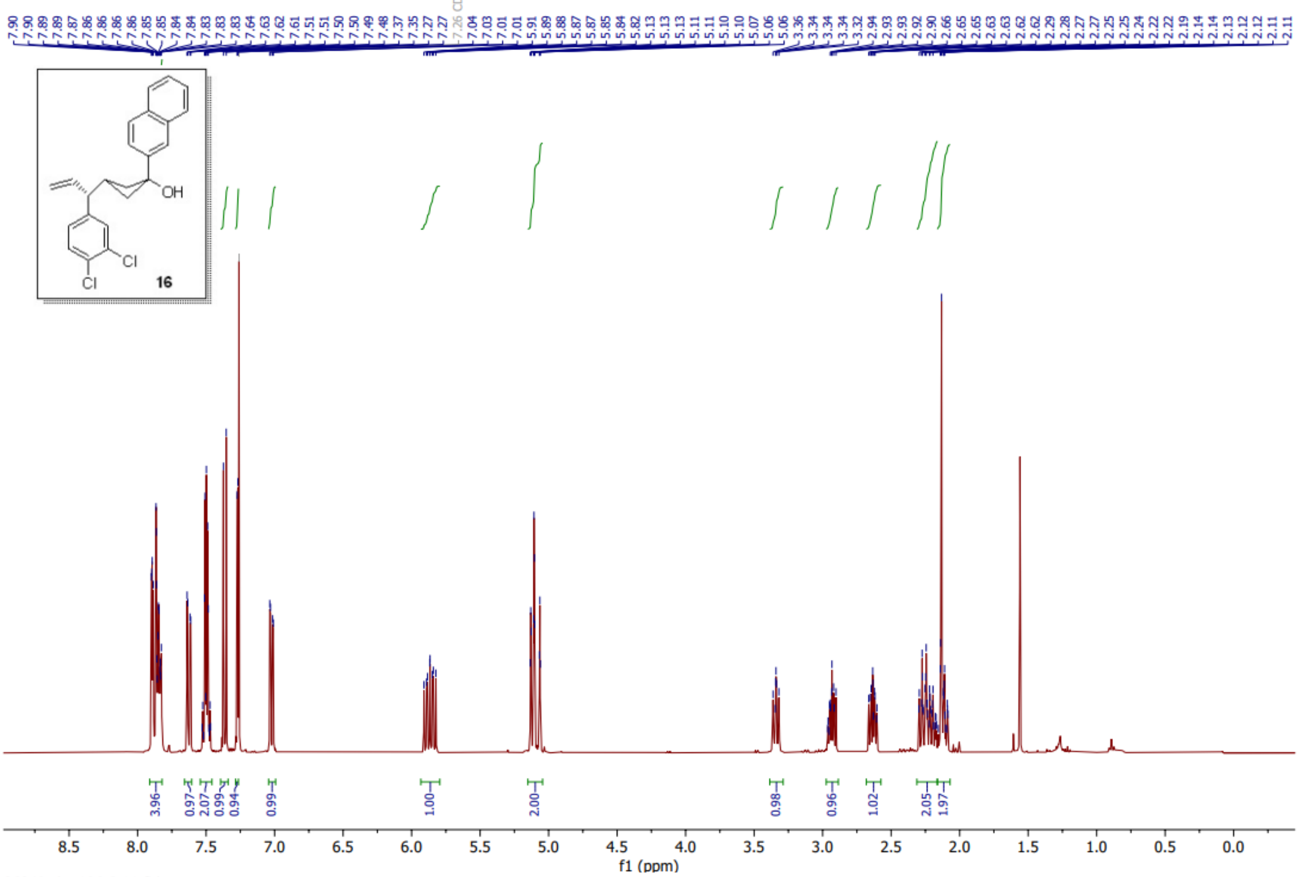

86840 shc-106-2.11.fid

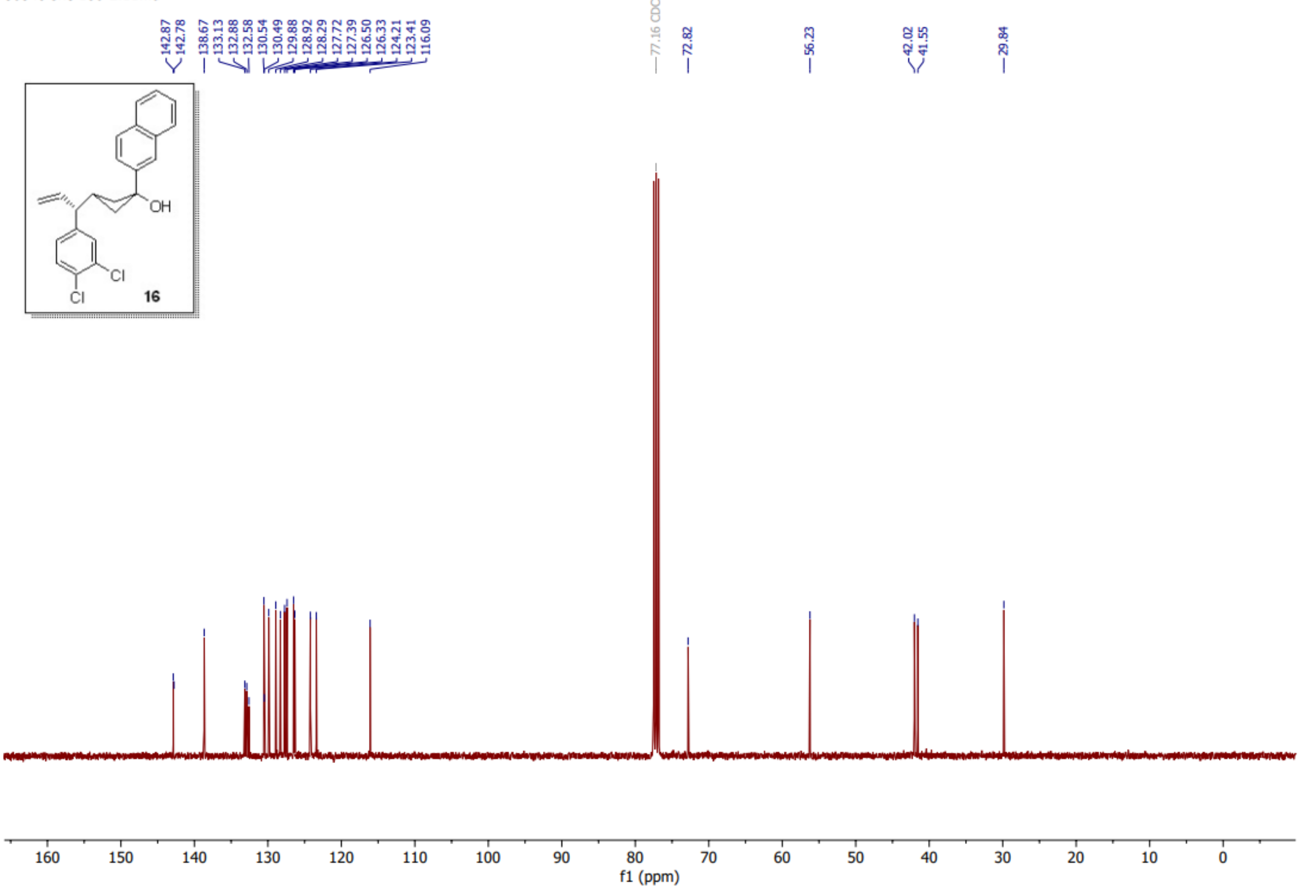

Compound 17

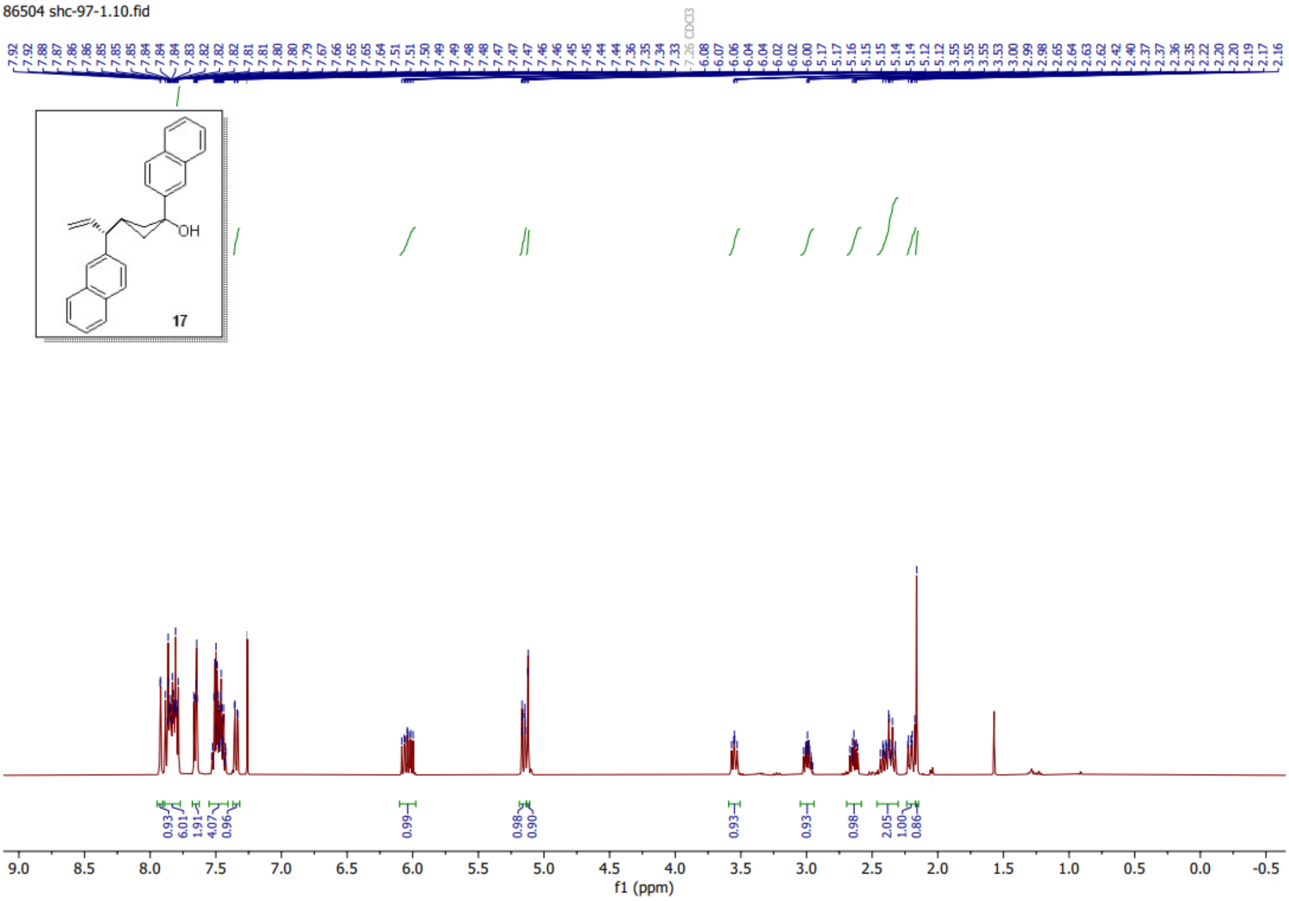
86504 shc-97-1.10.fid
OH
17
f1 (ppm)

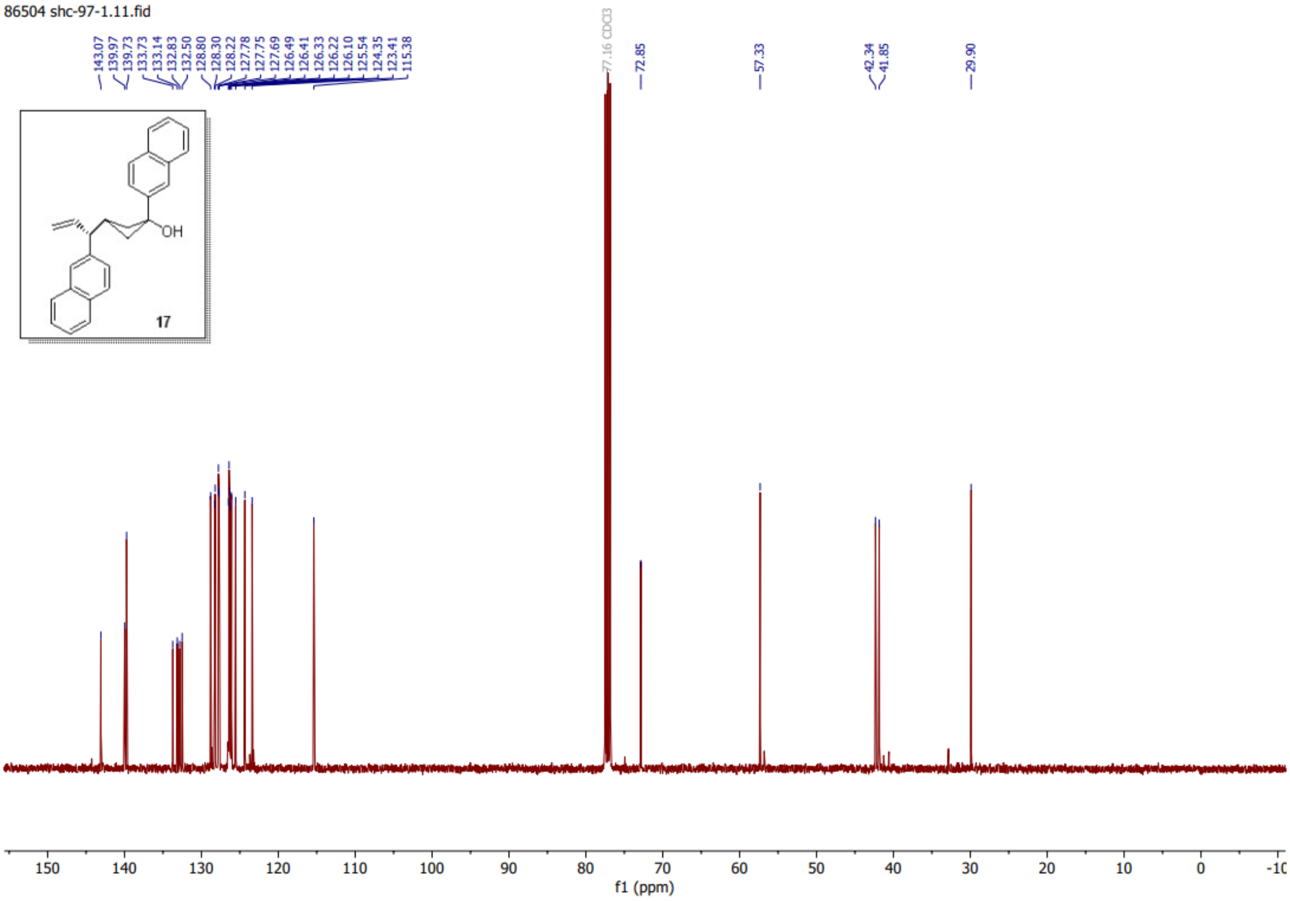
86504 shc-97-1.11.fid
OH
17
f1 (ppm)

Compound 18

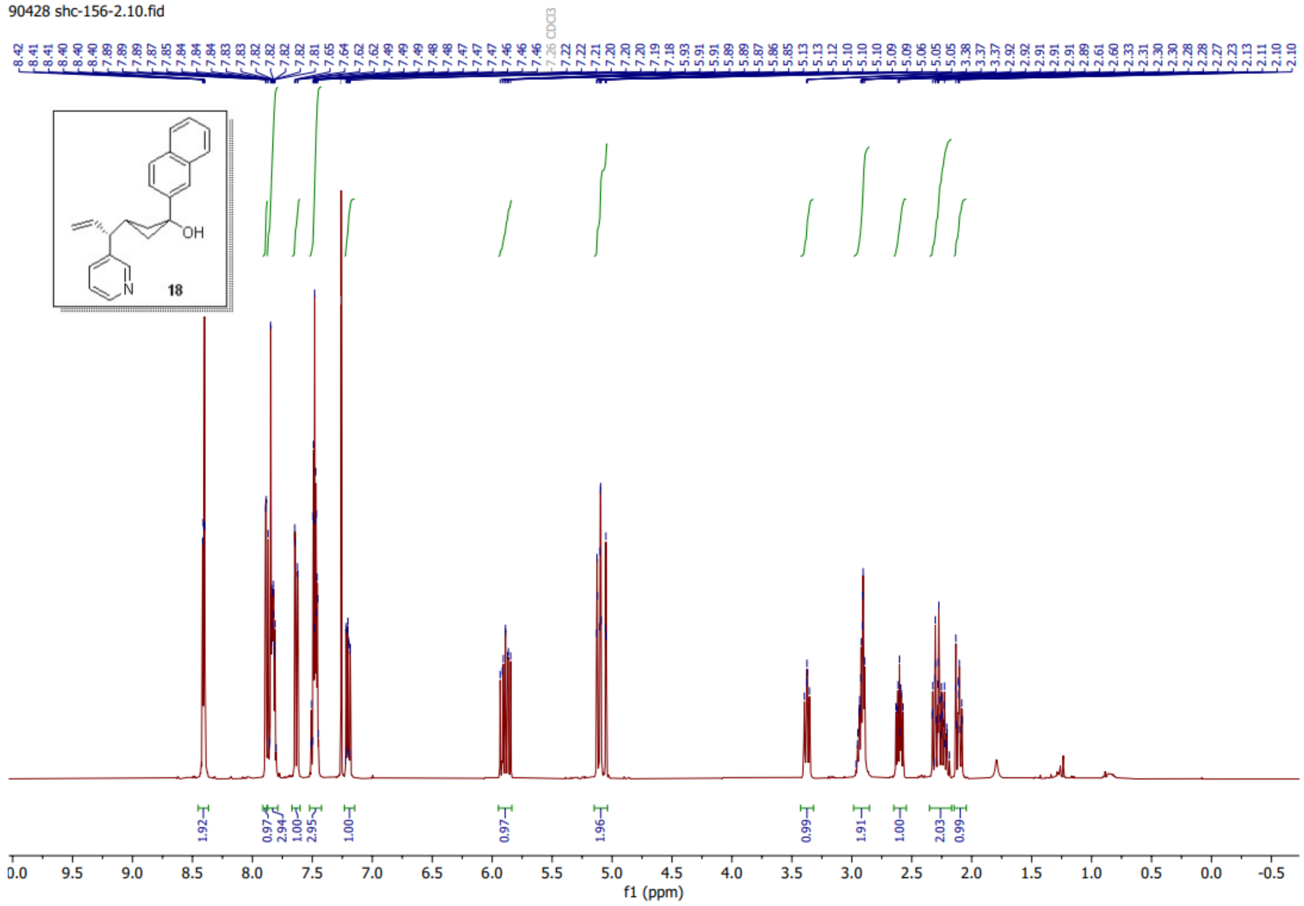

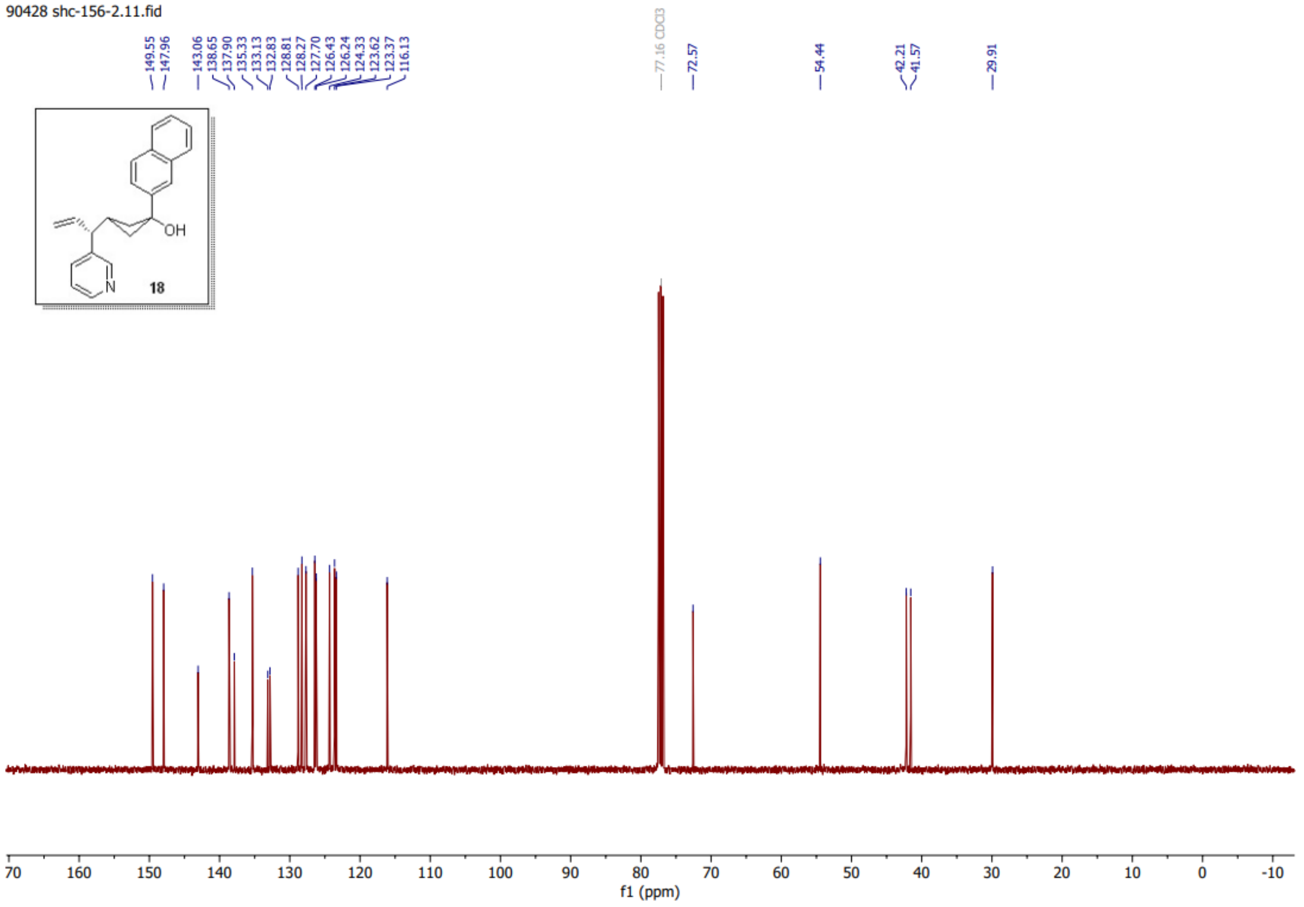

Compound 19

87453 shc-108-3q.10.fid

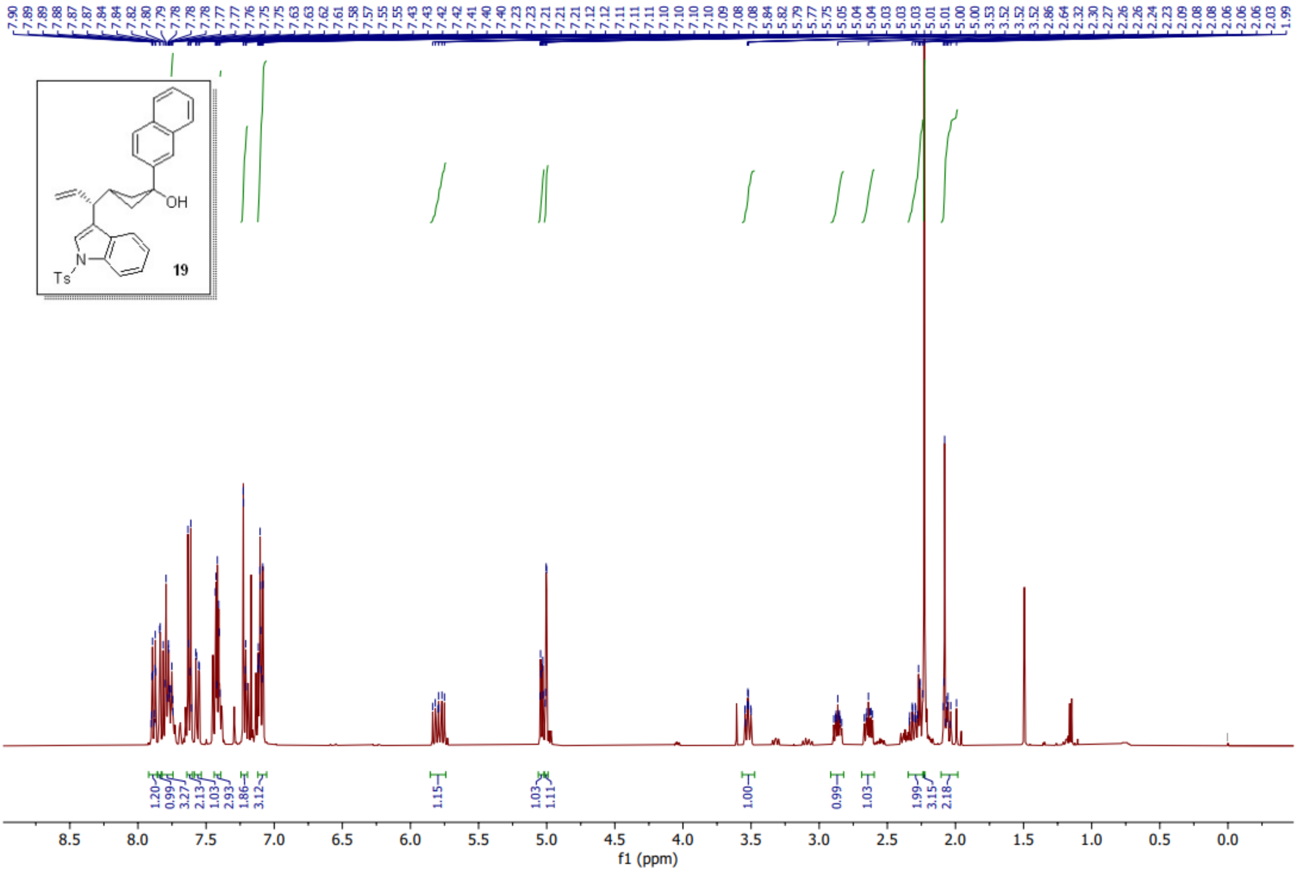

87453 shc-108-3q.11.fid

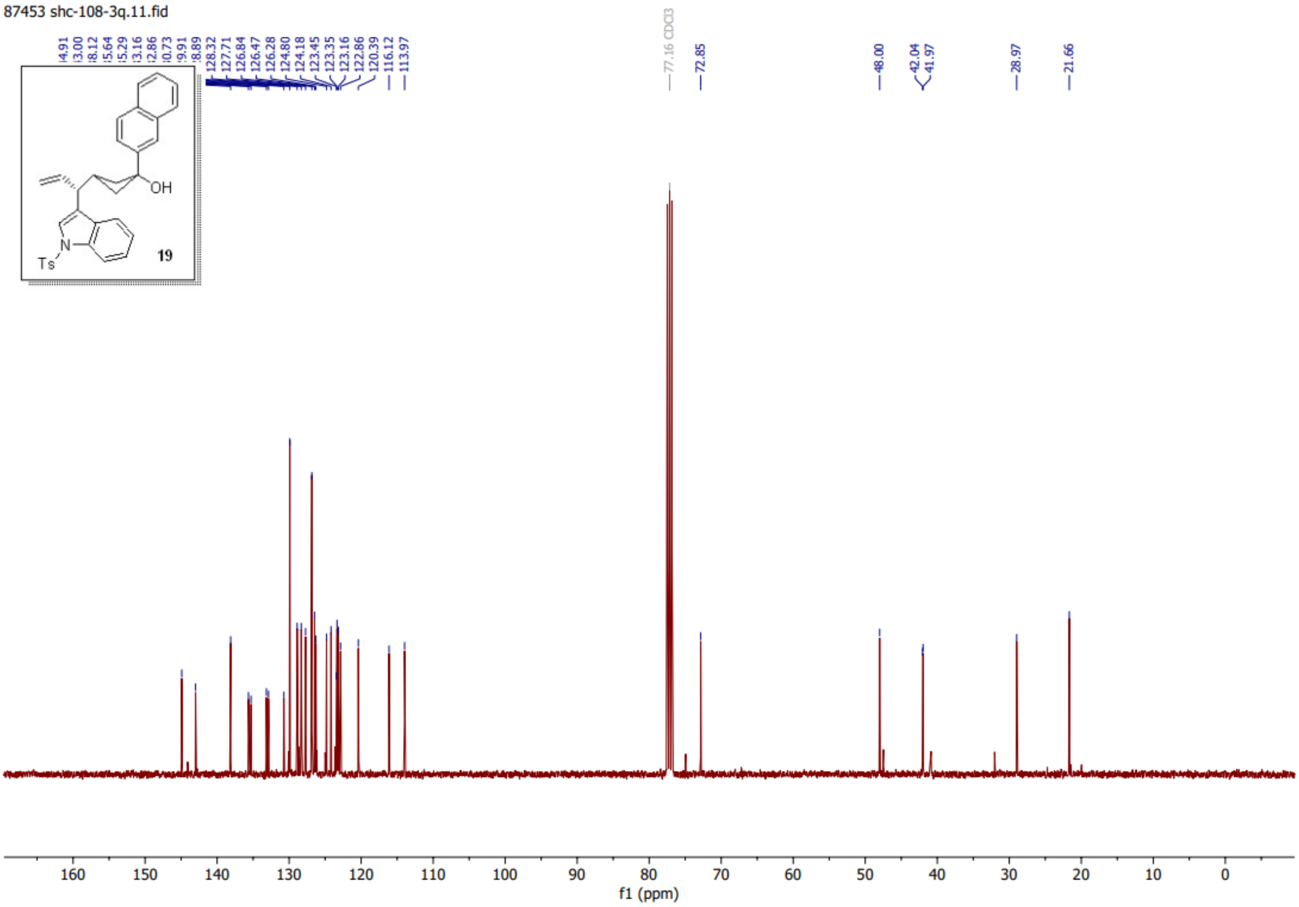

Compound 20

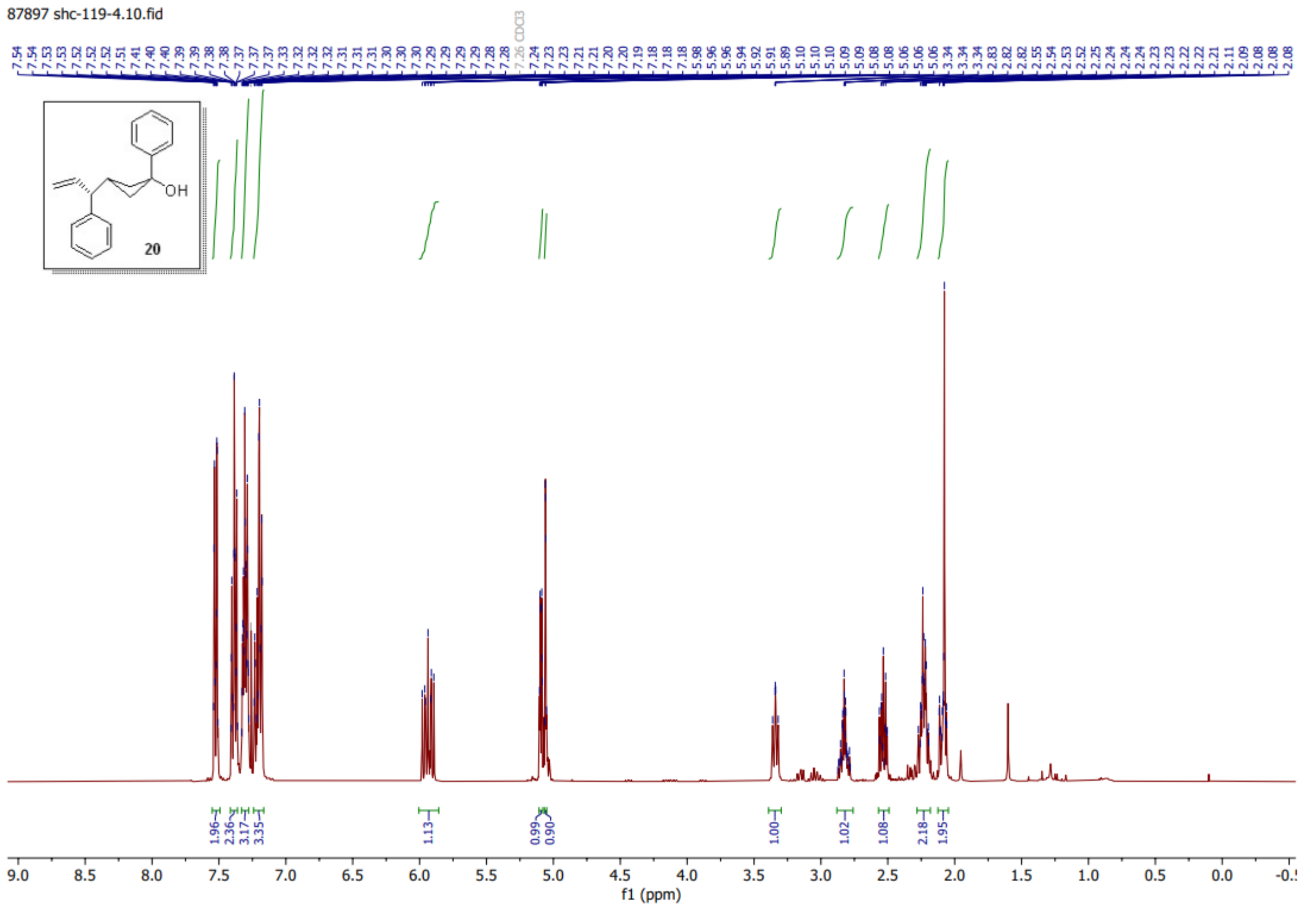
87897 shc-119-4.10.fid
20
OH
f1 (ppm)

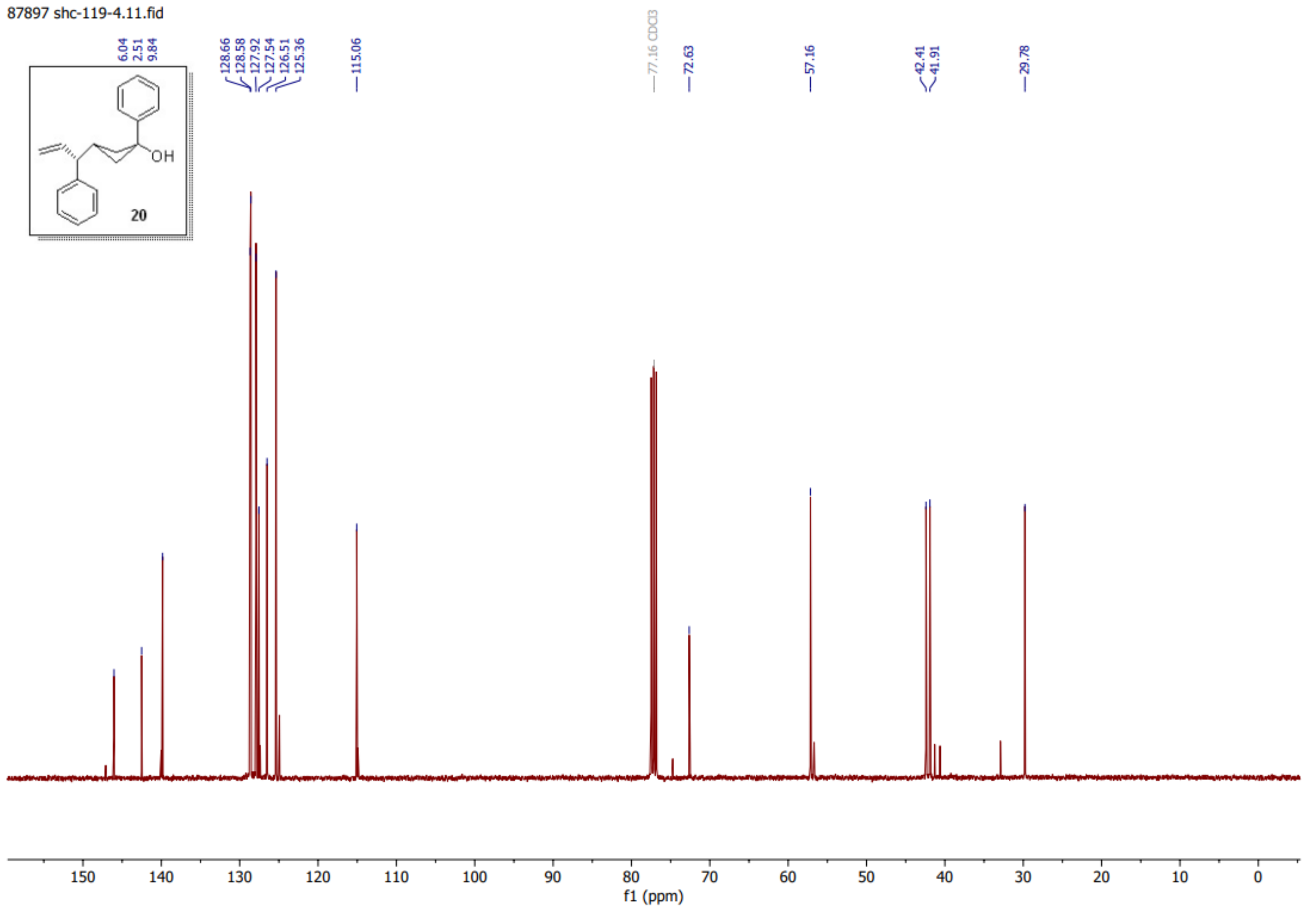
87897 shc-119-4.11.fid
20
OH
f1 (ppm)

Compound 21

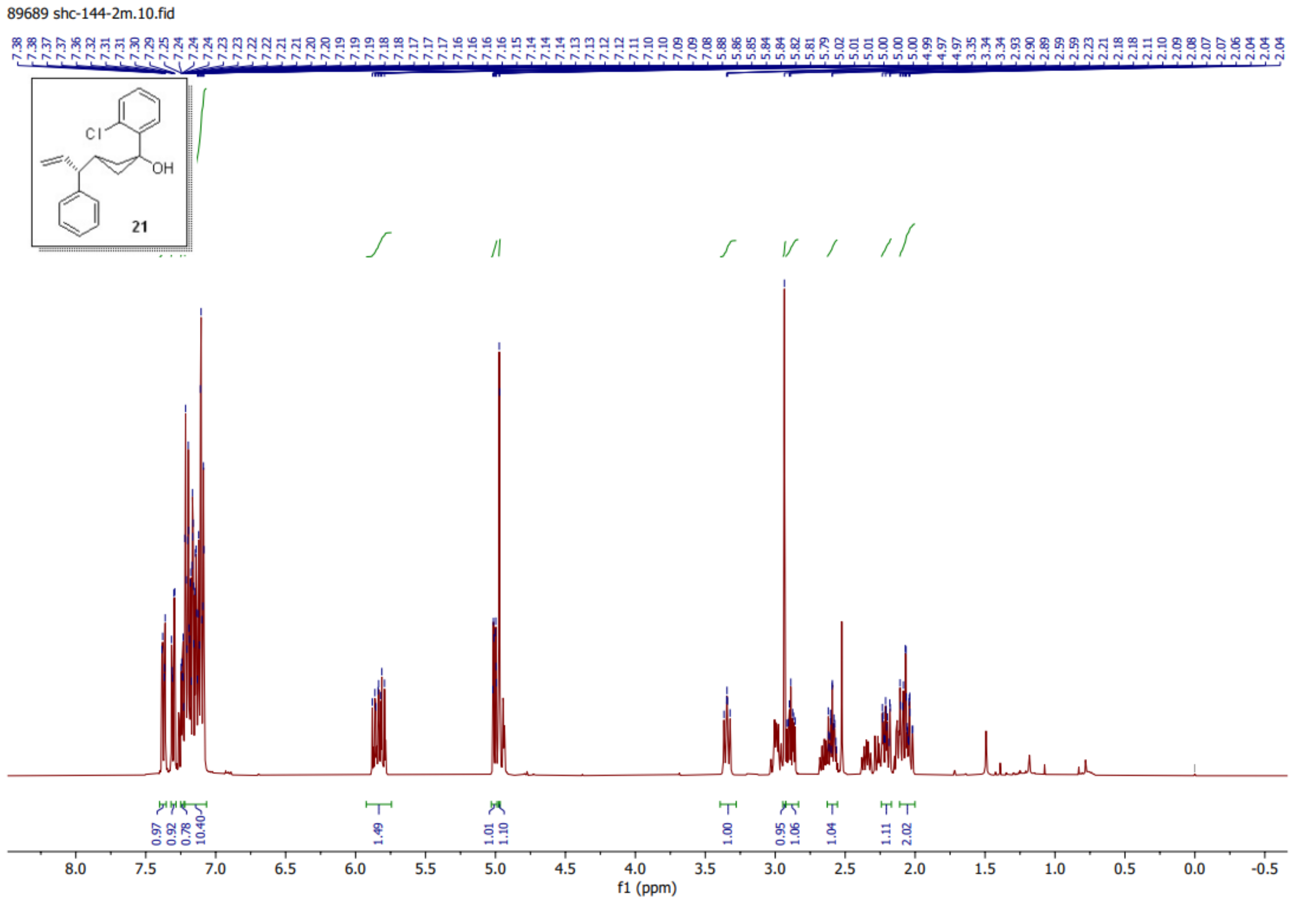
89689 shc-144-2m.10.fid
21
f1 (ppm)

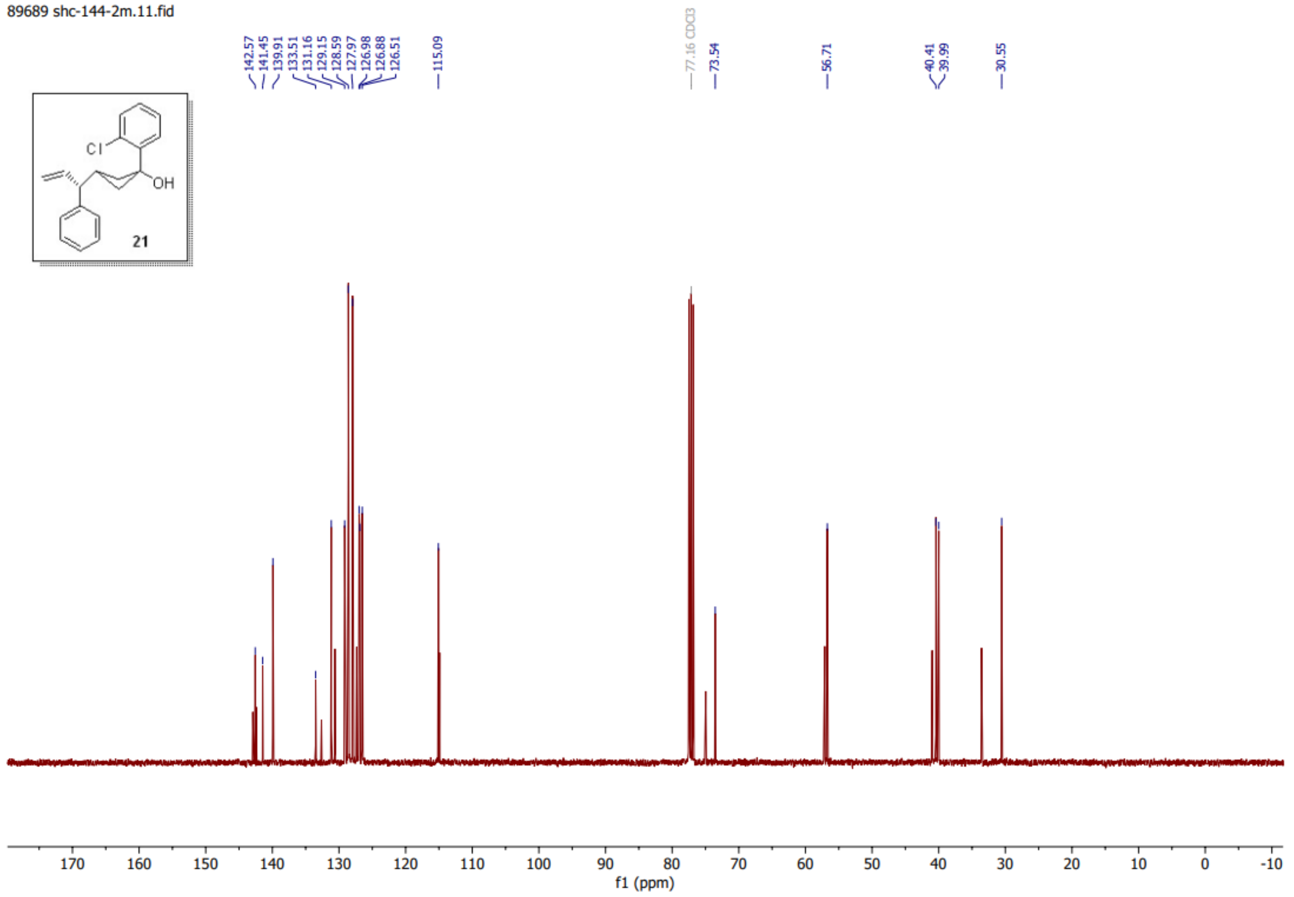
89689 shc-144-2m.11.fid
142.57
141.45
139.91
133.51
131.16
129.15
128.59
127.97
126.98
126.88
126.51
115.09
77.16 CDCl3
73.54
56.71
40.41
39.99
30.55
21
f1 (ppm)

Compound 22

91108 shc-171-2.10.fid

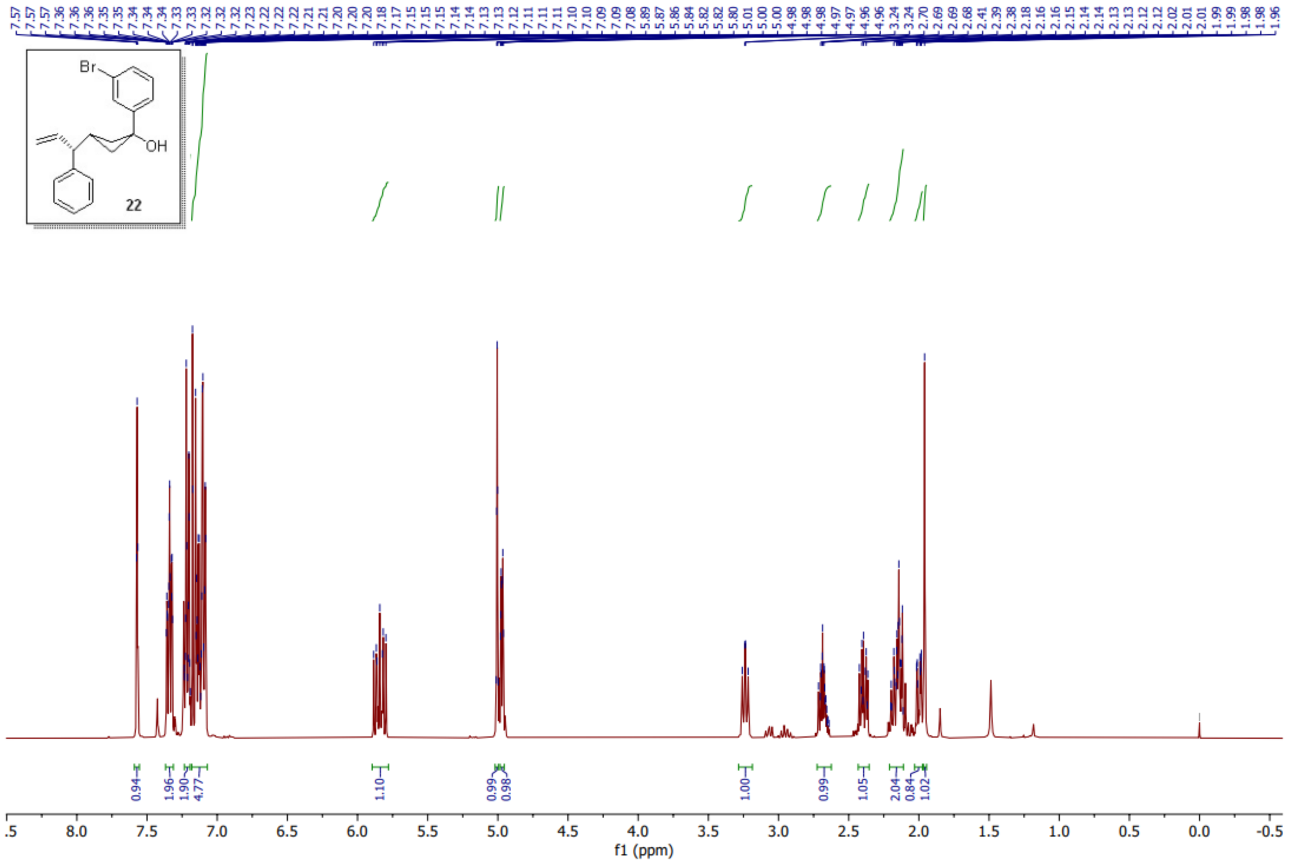

91108 shc-171-2.11.fid

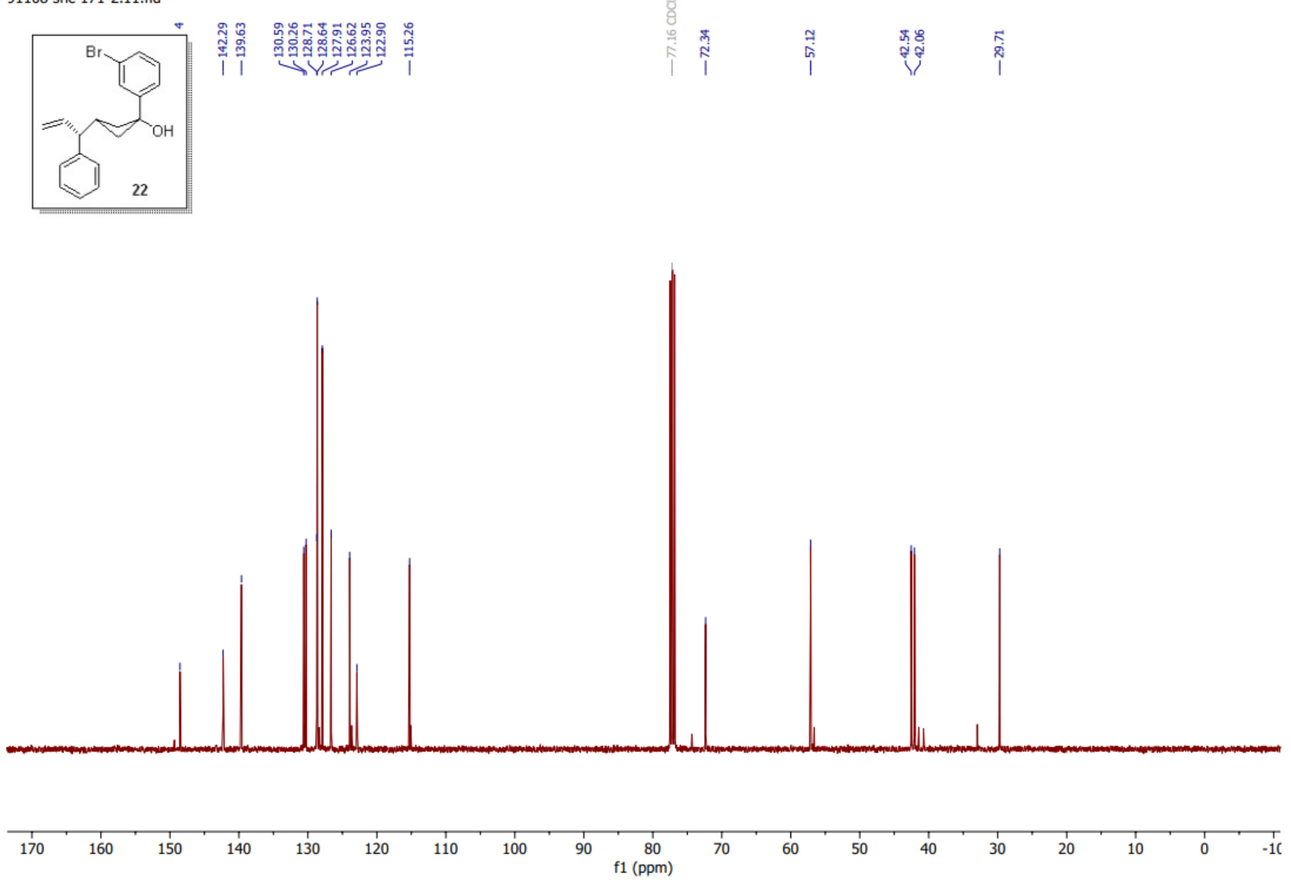

Compound 23

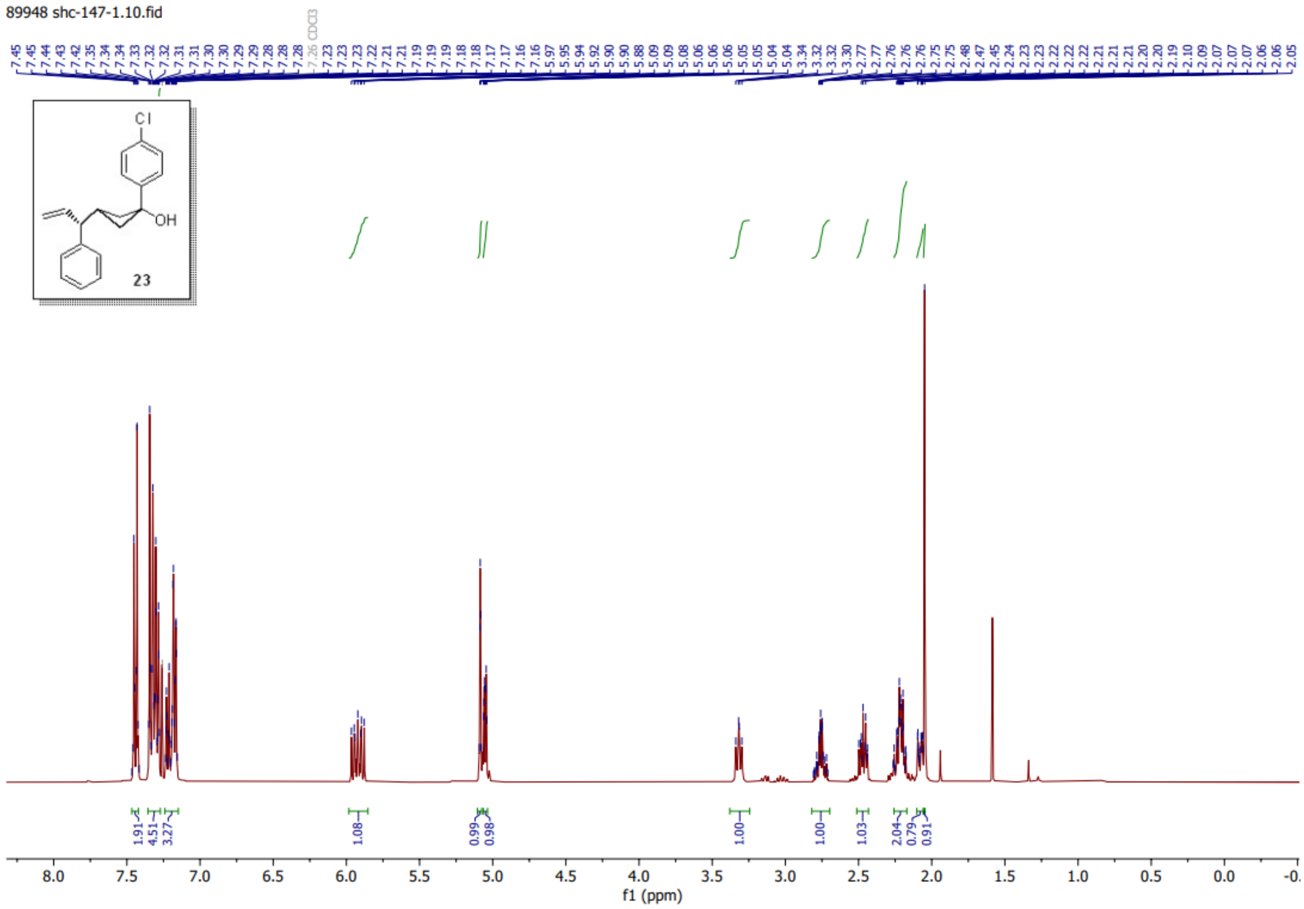
89948 shc-147-1.10.fid
23
f1 (ppm)

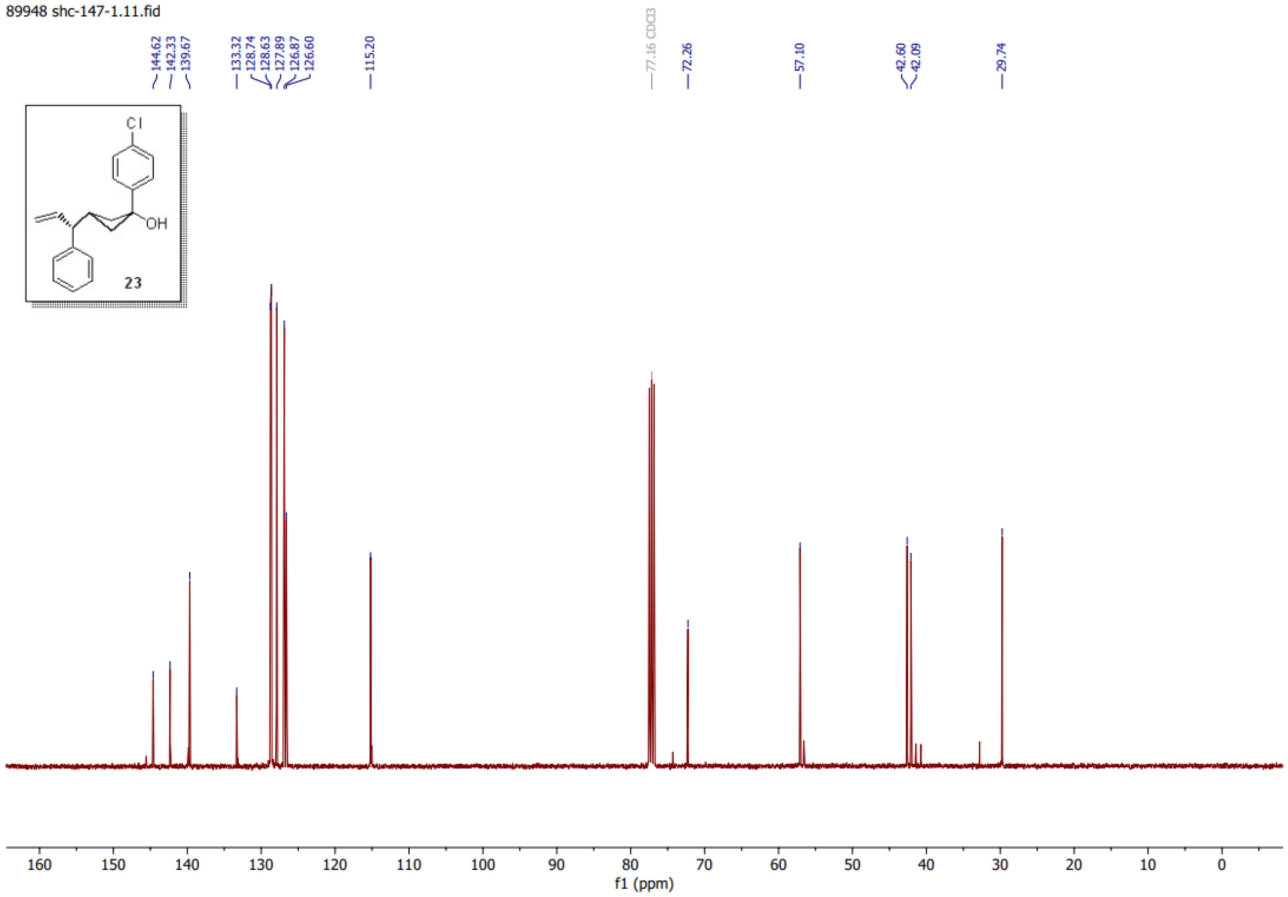
89948 shc-147-1.11.fid
144.62
142.33
139.67
133.32
128.74
128.63
127.89
126.87
126.60
115.20
77.16 CDCl3
72.26
57.10
42.60
42.09
29.74
23
f1 (ppm)

Compound 24

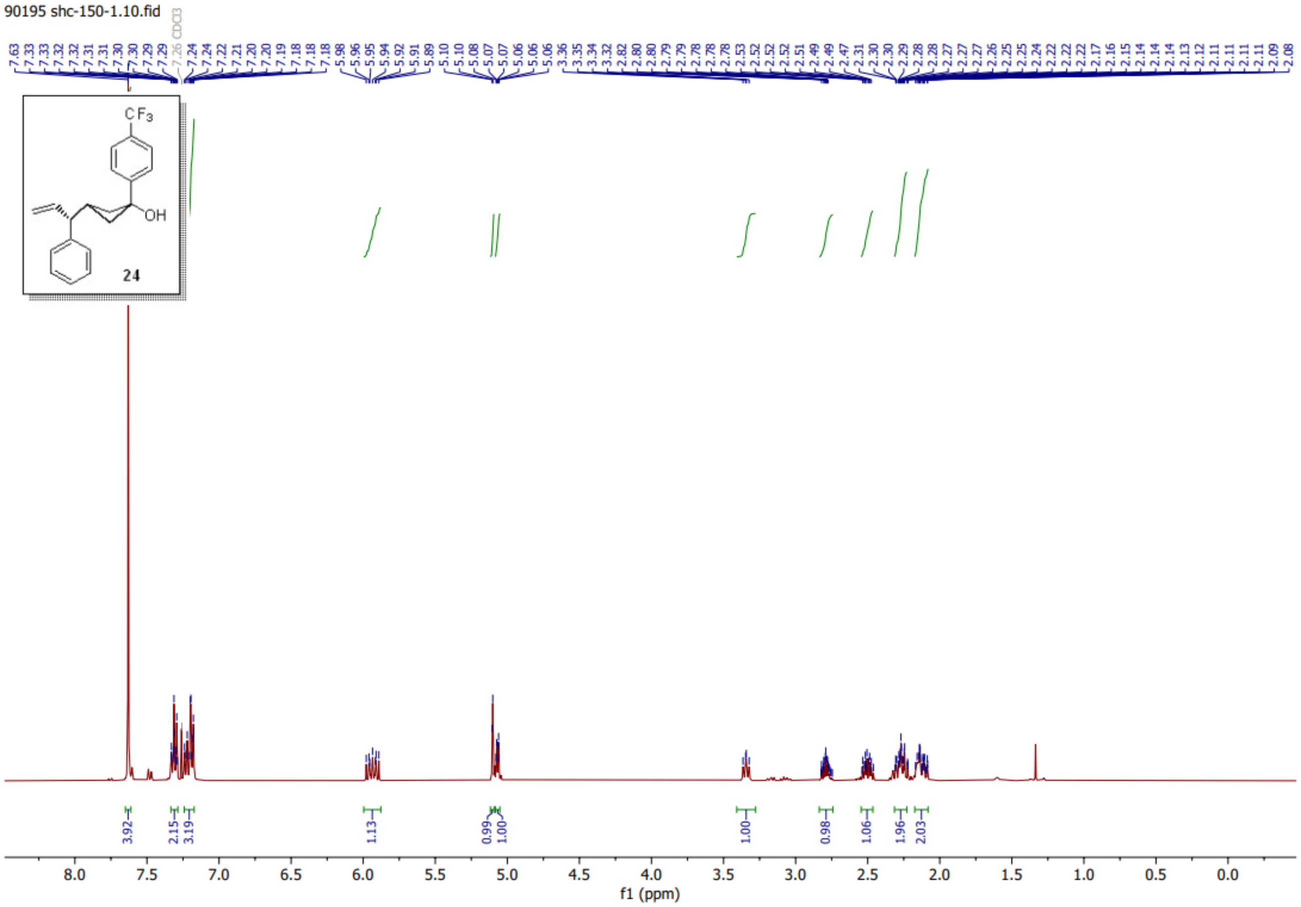
90195 shc-150-1.10.fid
24
f1 (ppm)

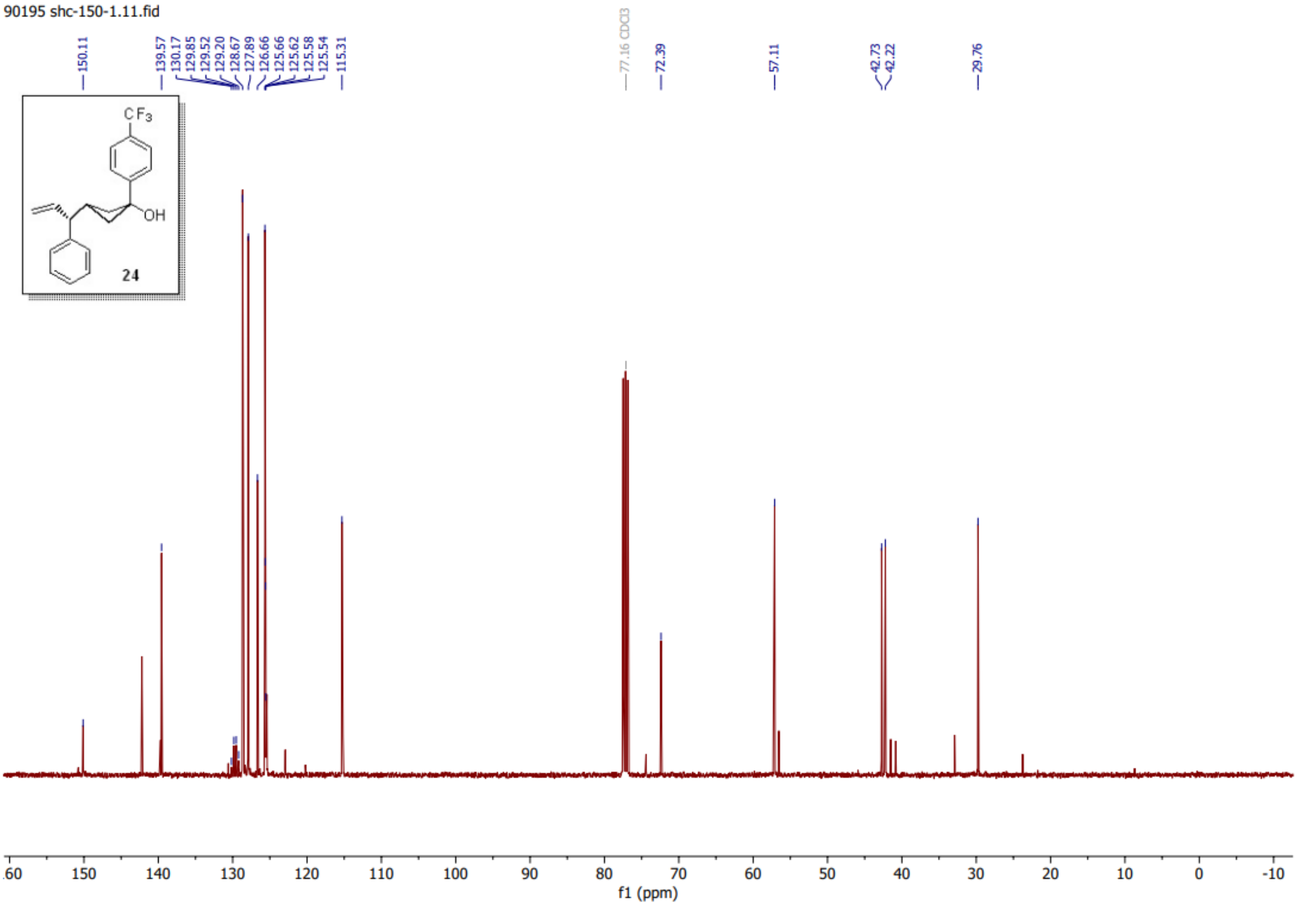
90195 shc-150-1.11.fid
150.11
139.57
130.17
129.85
129.52
129.20
128.67
127.89
126.66
125.66
125.62
125.58
125.54
115.31
77.16 CDCl3
72.39
57.11
42.73
42.22
29.76
24
f1 (ppm)

Compound 25

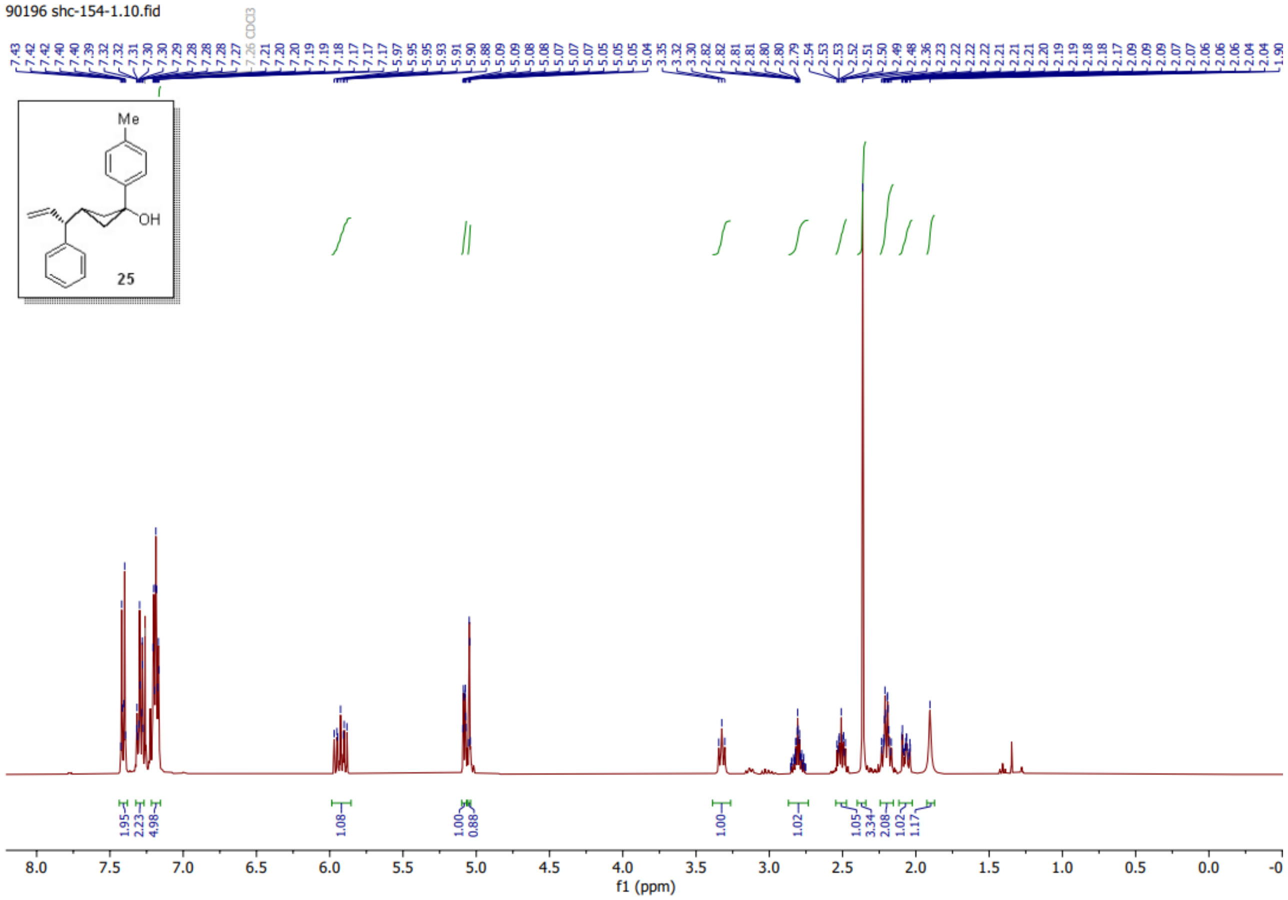
90196 shc-154-1.10.fid
Me
OH
25
1.95
2.23
4.98
1.08
1.00
0.88
1.00
1.02
1.05
3.34
2.08
1.02
1.17
f1 (ppm)

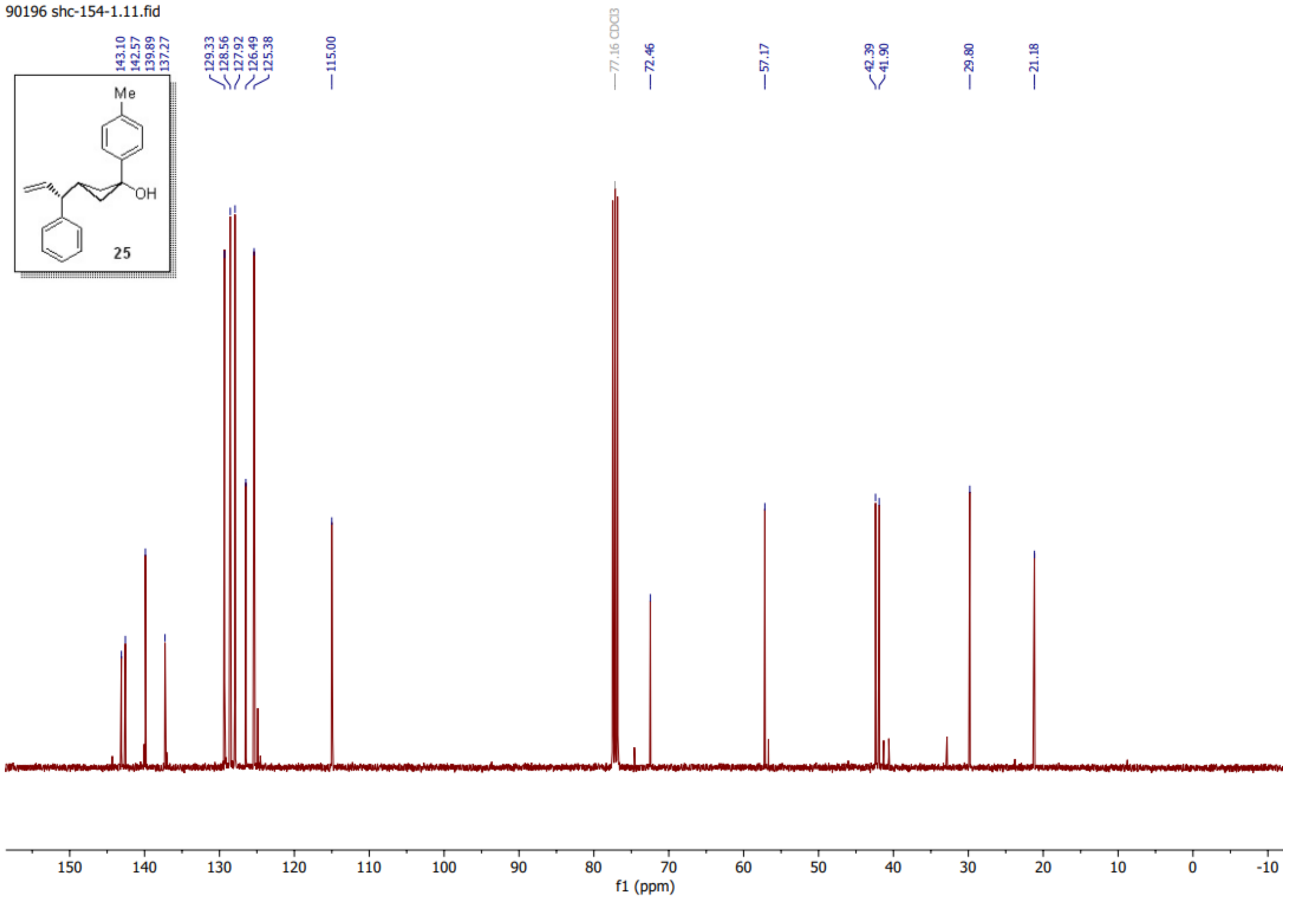
90196 shc-154-1.11.fid
143.10
142.57
139.89
137.27
129.33
128.56
127.92
126.49
125.38
115.00
77.16 CDCl3
72.46
57.17
42.39
41.90
29.80
21.18
Me
OH
25
f1 (ppm)

Compound 26

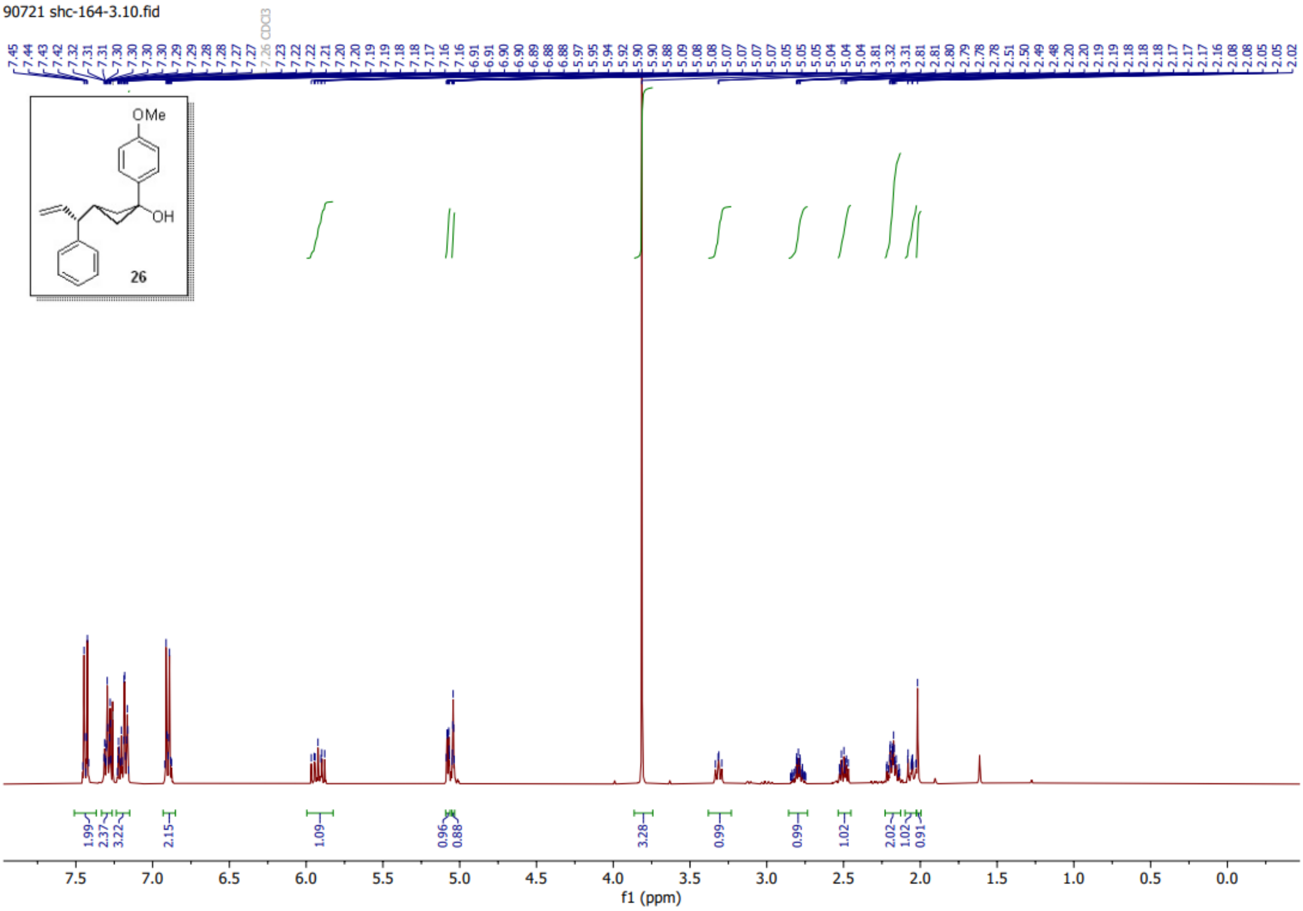
90721 shc-164-3.10.fid
OMe
OH
26
f1 (ppm)

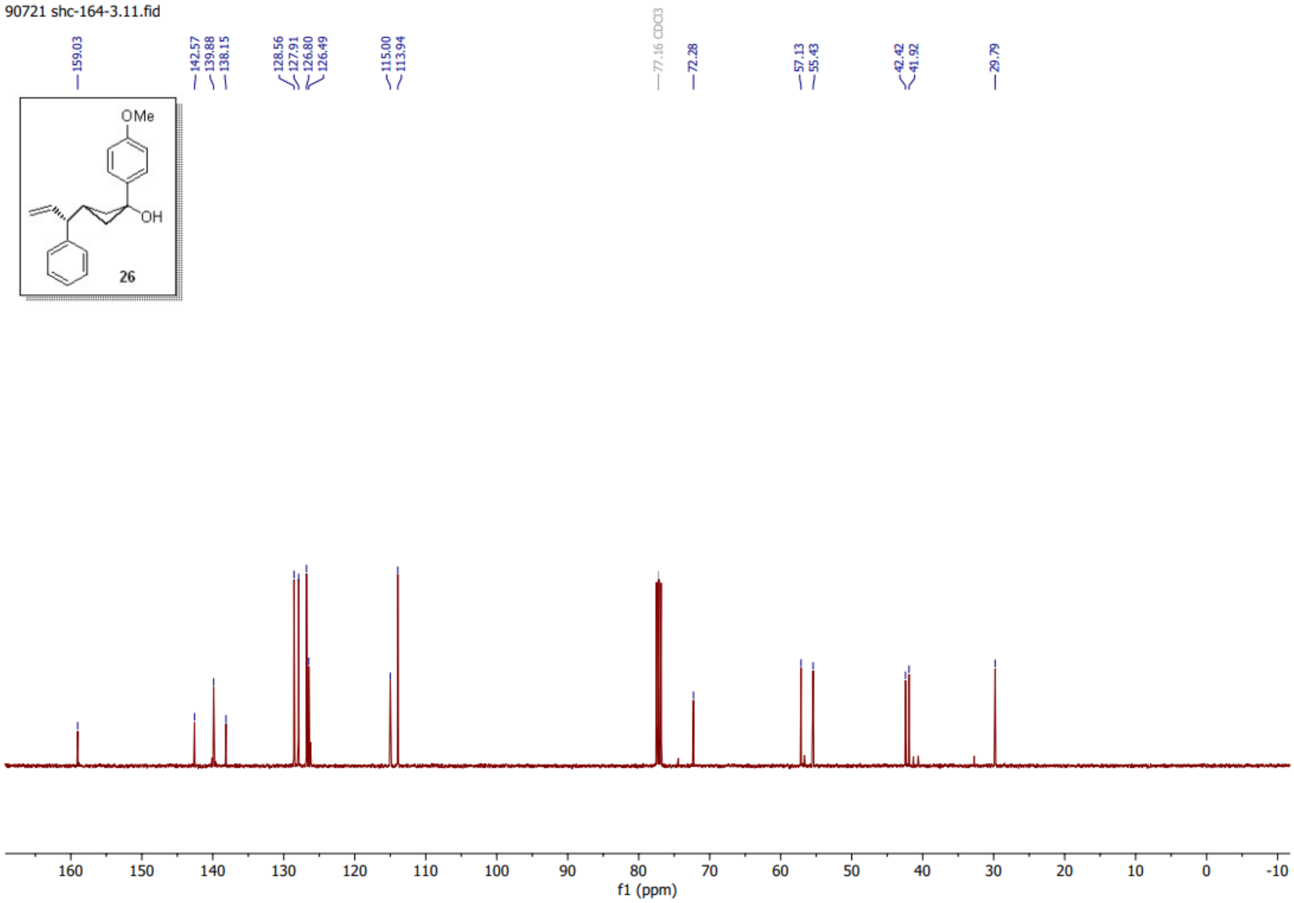
90721 shc-164-3.11.fid
159.03
142.57
139.88
138.15
128.56
127.91
126.80
126.49
115.00
113.94
77.16 CDCl3
72.28
57.13
55.43
42.42
41.92
29.79
OMe
OH
26
f1 (ppm)

Compound 27

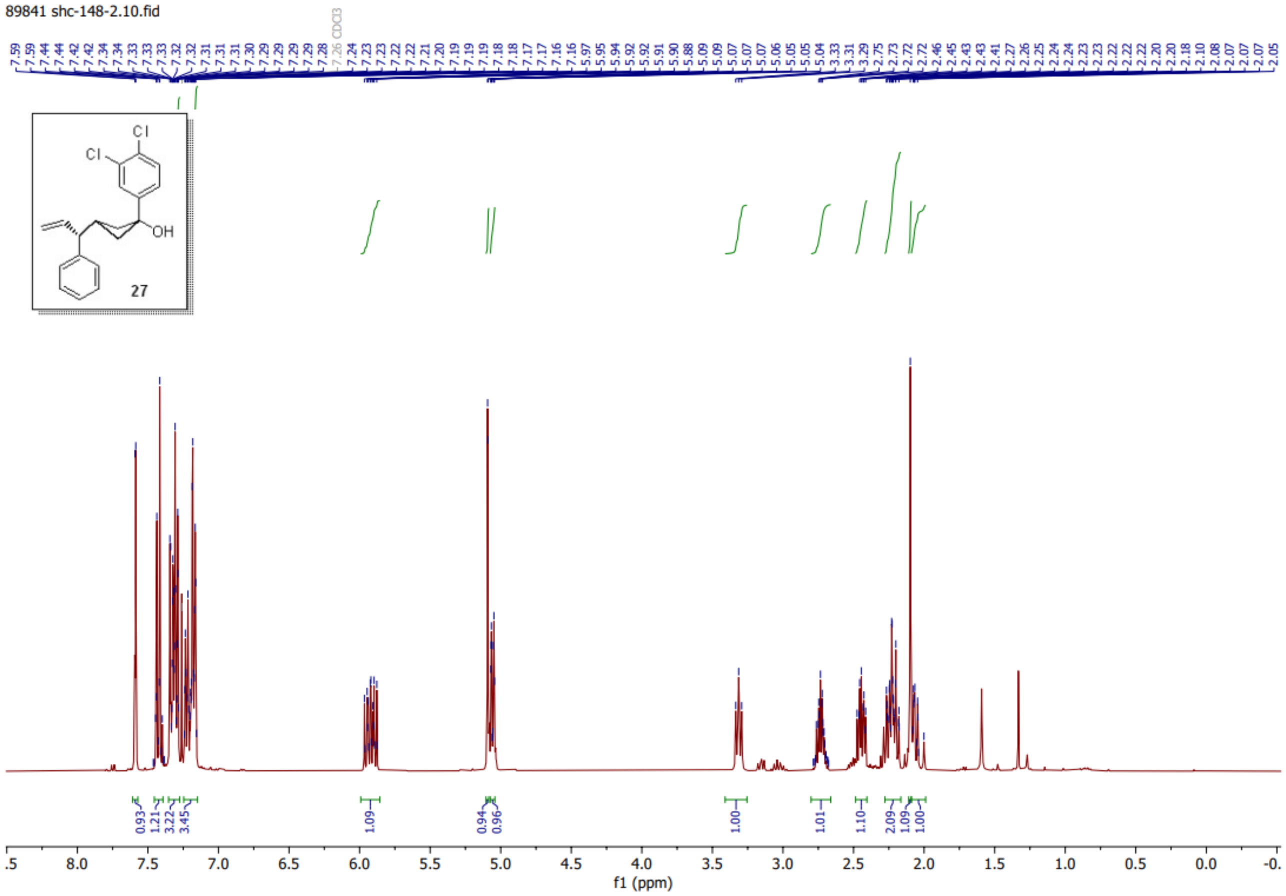
89841 shc-148-2.10.fid
7.59 7.59 7.44 7.44 7.42 7.42 7.34 7.34 7.33 7.33 7.33 7.32 7.32 7.31 7.31 7.30 7.29 7.29 7.29 7.28 7.26 CDCl3 7.24 7.23 7.23 7.22 7.22 7.21 7.20 7.19 7.19 7.19 7.18 7.17 7.17 7.16 7.16 5.97 5.95 5.94 5.92 5.92 5.91 5.90 5.88 5.09 5.09 5.07 5.07 5.06 5.05 5.05 5.04 3.33 3.31 3.29 2.75 2.73 2.72 2.72 2.46 2.45 2.43 2.43 2.41 2.27 2.26 2.25 2.24 2.24 2.23 2.23 2.22 2.22 2.22 2.20 2.18 2.10 2.08 2.07 2.07 2.07 2.05
Cl
Cl
OH
27
0.93 1.21 3.22 3.45 1.09 0.94 0.96 1.00 1.01 1.10 2.09 1.09 1.00
f1 (ppm)

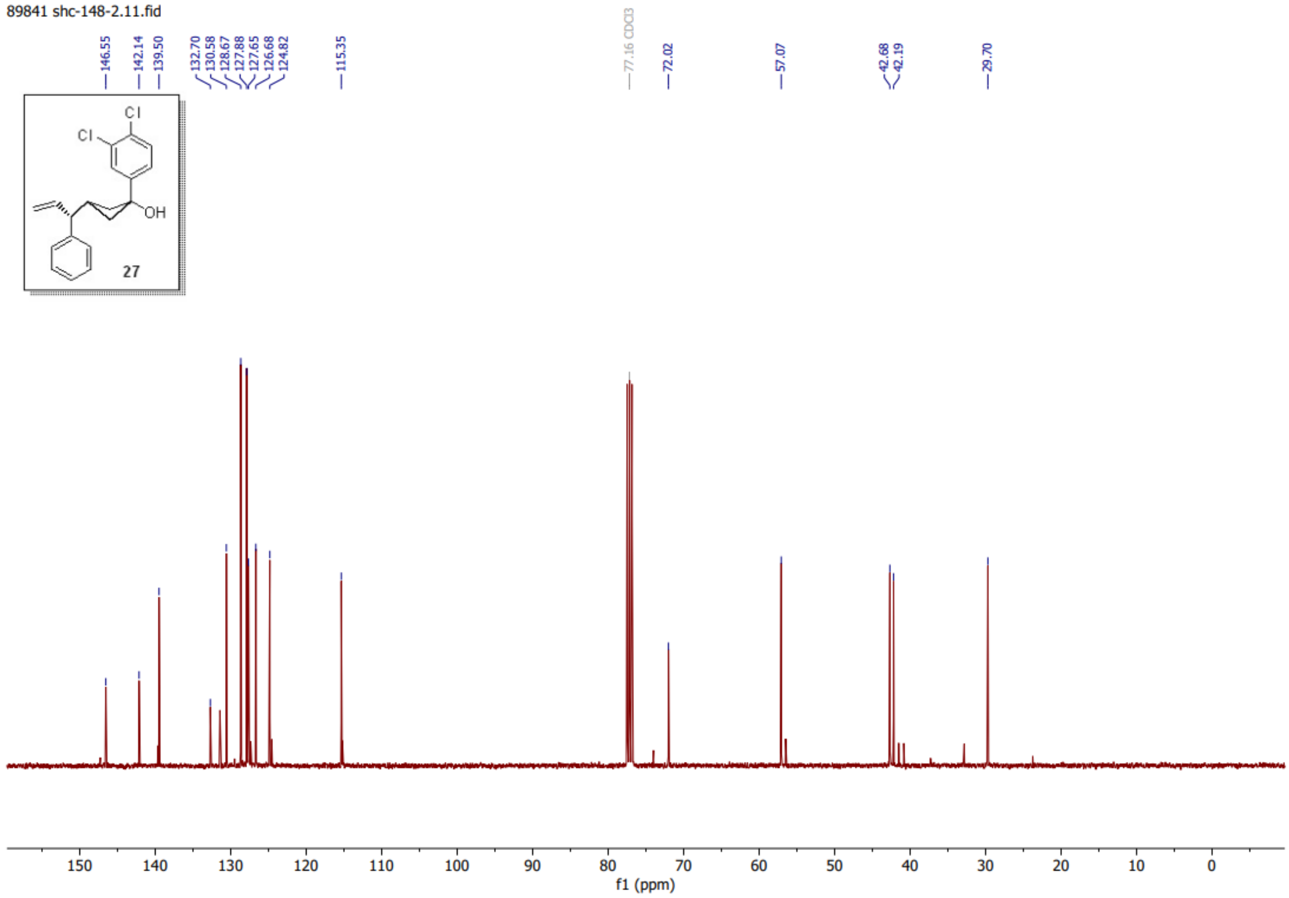
89841 shc-148-2.11.fid
146.55 142.14 139.50 132.70 130.58 128.67 127.88 127.65 126.68 124.82 115.35 77.16 CDCl3 72.02 57.07 42.68 42.19 29.70
Cl
Cl
OH
27
f1 (ppm)

Compound 28

87896 shc-114-2nn.10.fid

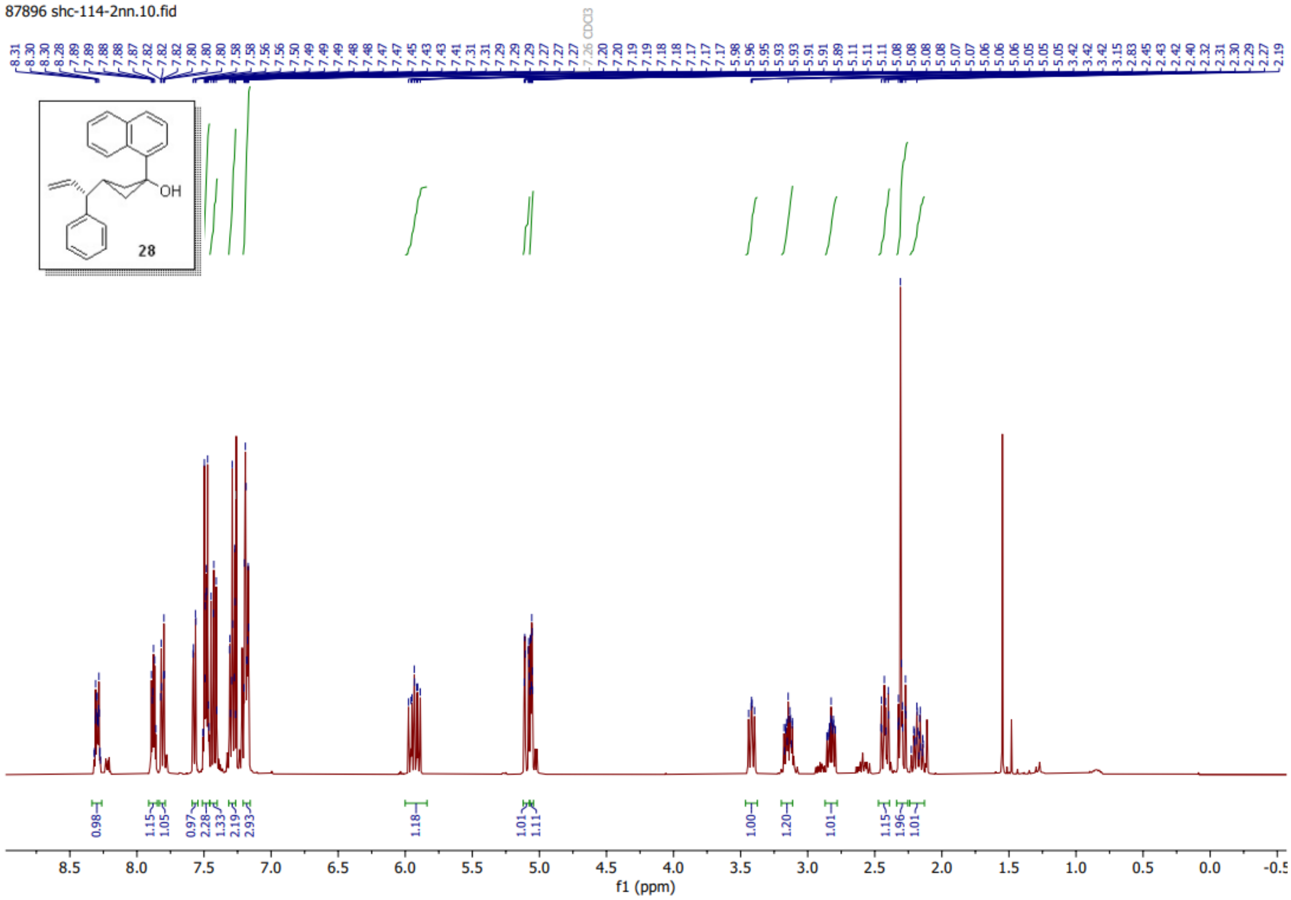

87896 shc-114-2nn.11.fid

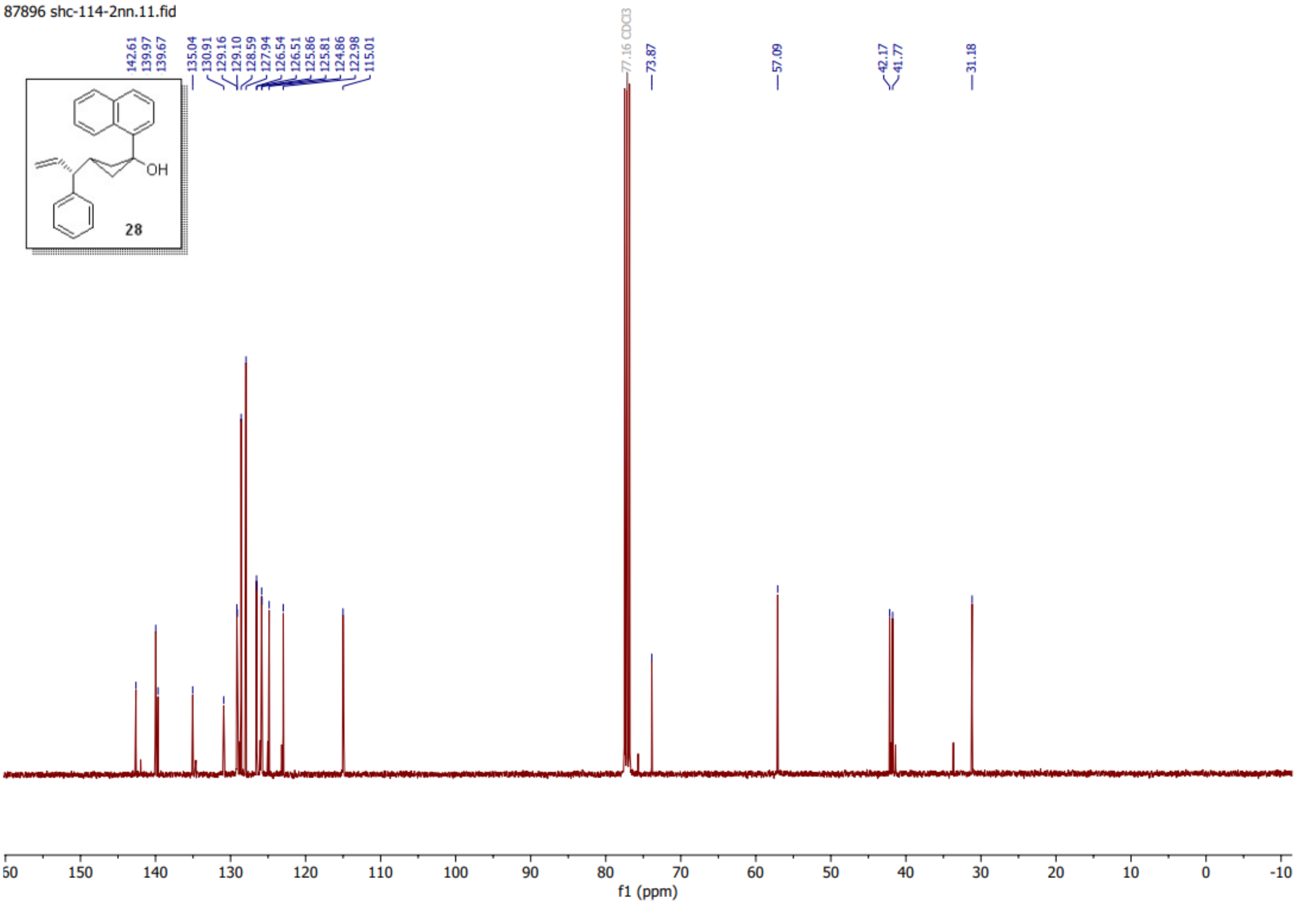

Compound 29

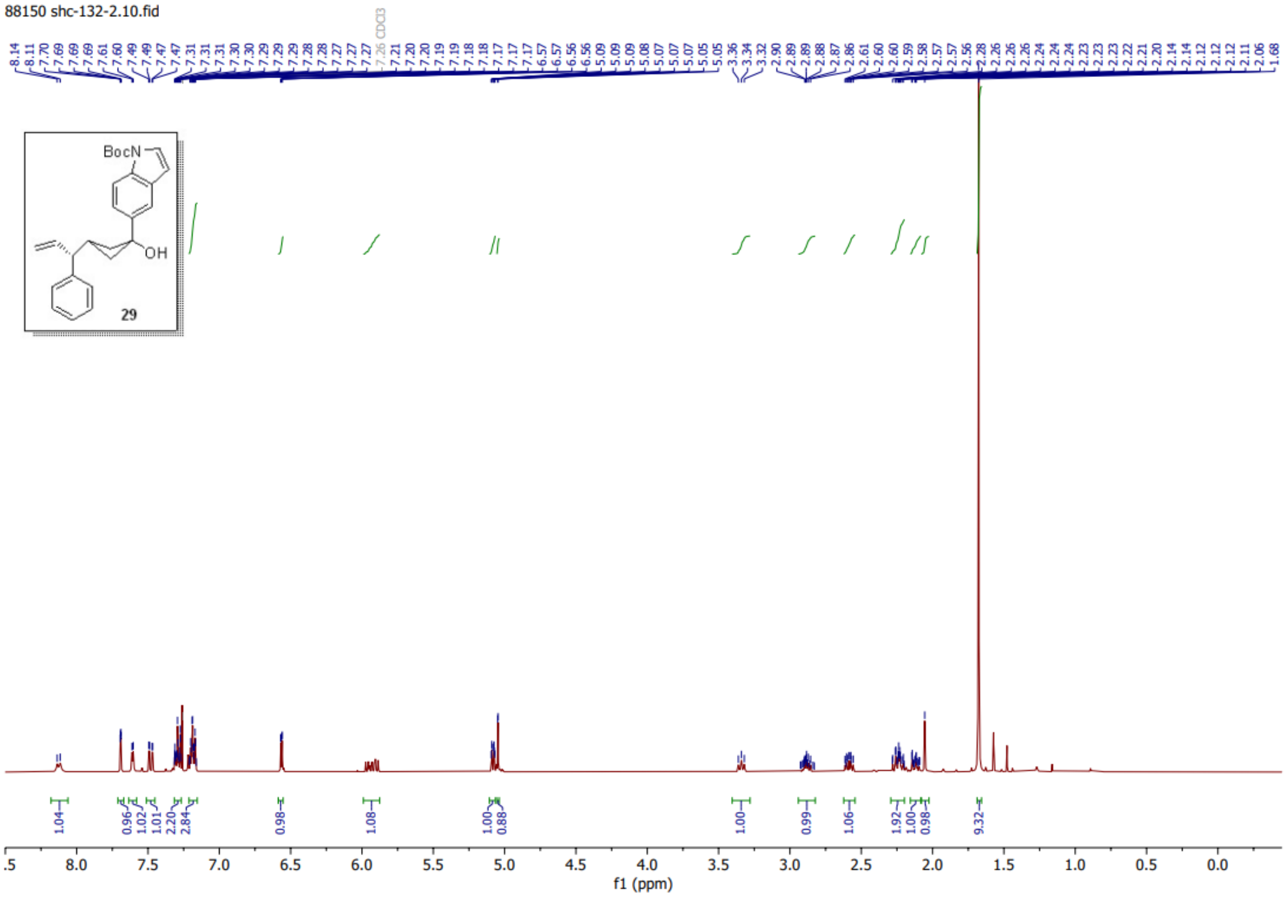
88150 shc-132-2.10.fid
29
f1 (ppm)

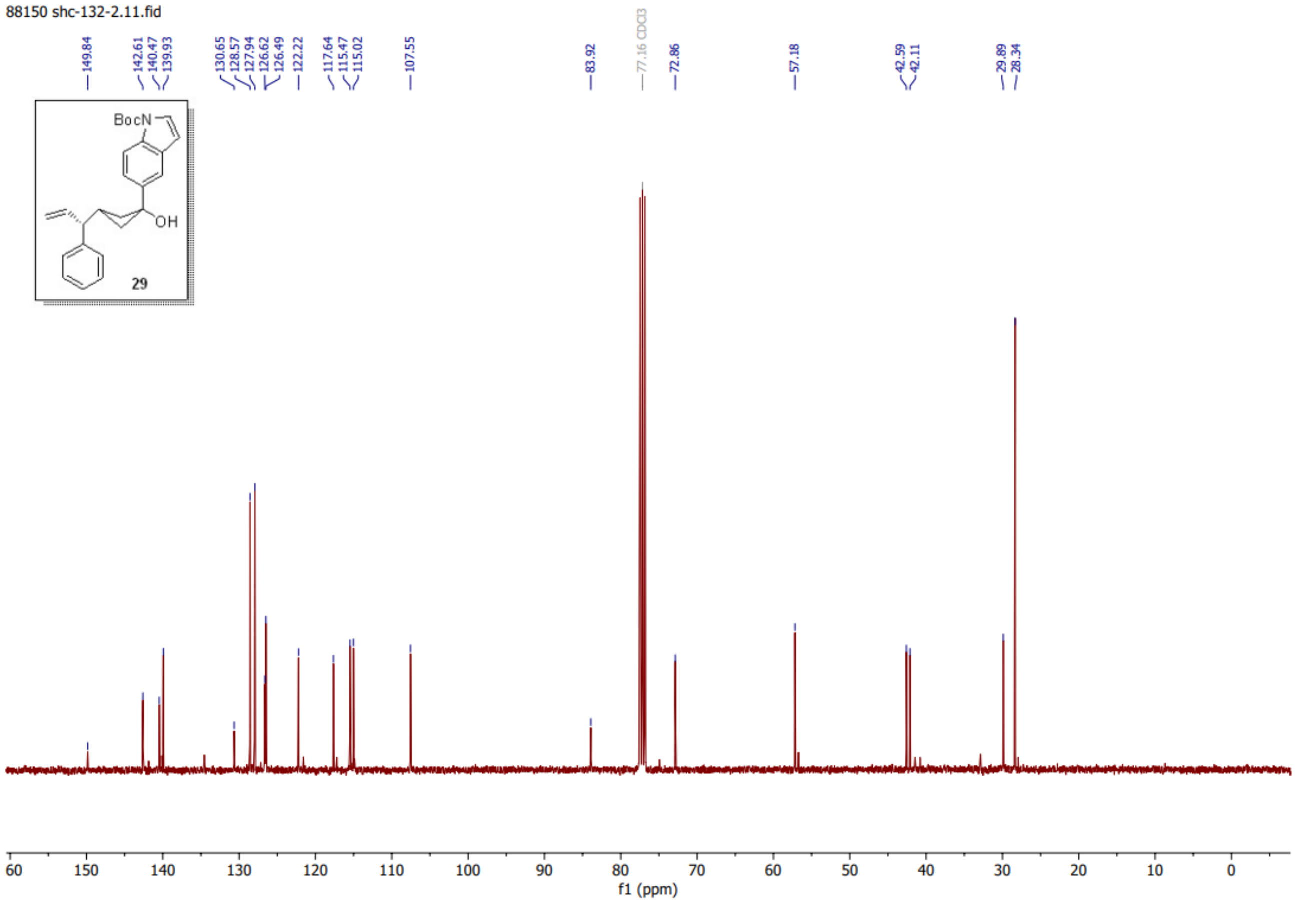
88150 shc-132-2.11.fid
149.84
142.61
140.47
139.93
130.65
128.57
127.94
126.62
126.49
122.22
117.64
115.47
115.02
107.55
83.92
77.16 CDCl3
72.86
57.18
42.59
42.11
29.89
28.34
29
f1 (ppm)

Compound 30

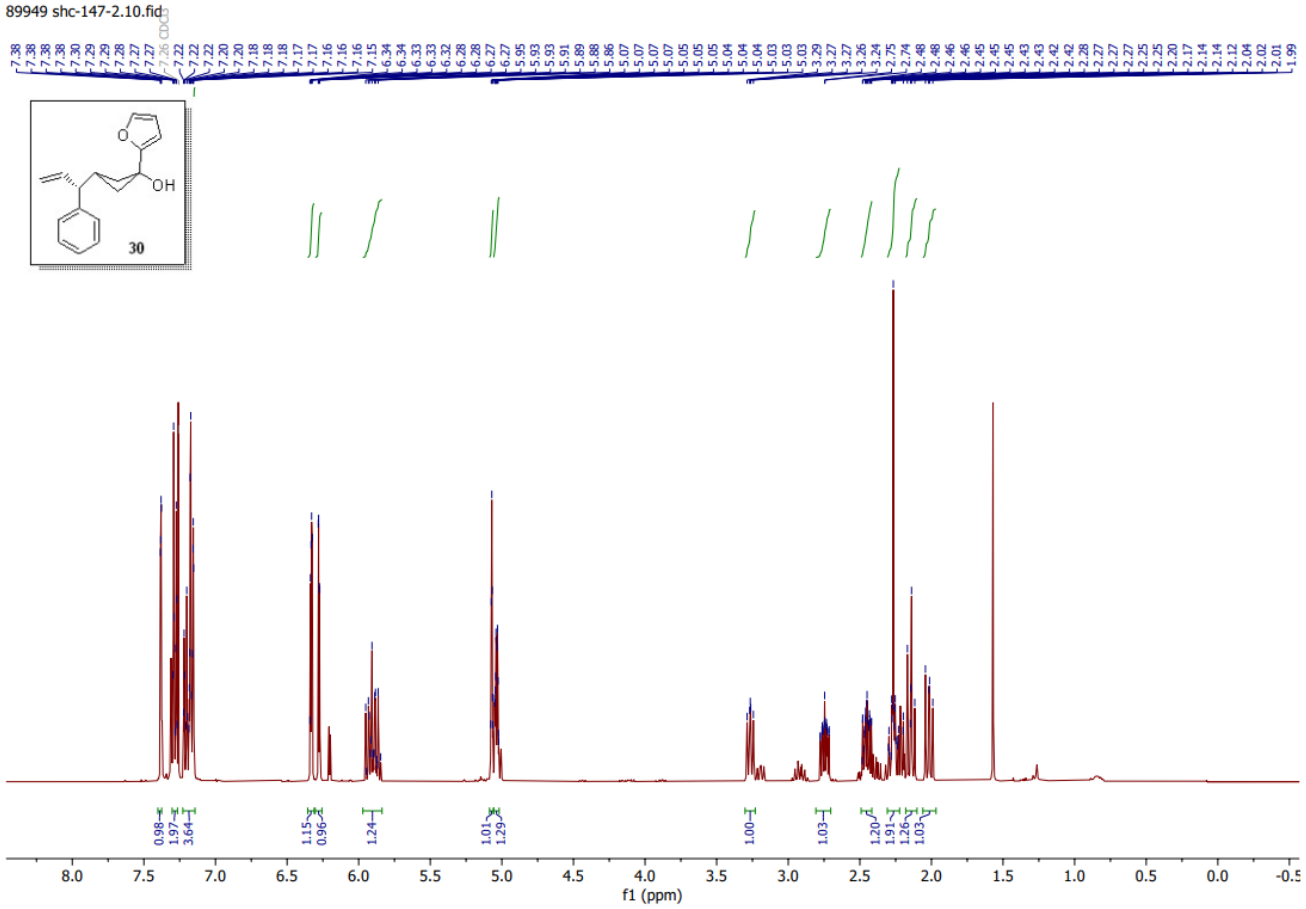
89949 shc-147-2.10.fid
30
OH
O
f1 (ppm)

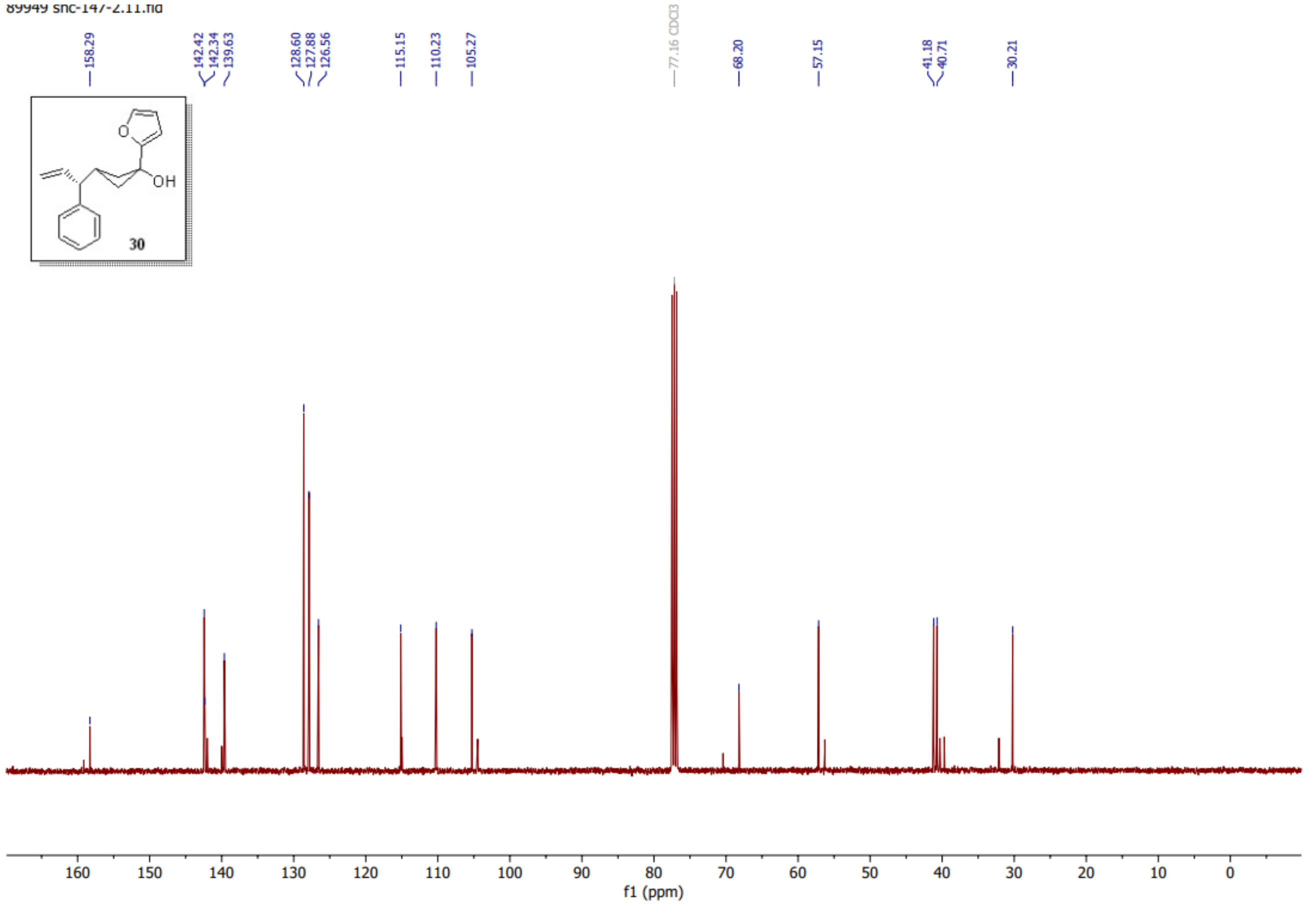
89949 shc-147-2.11.fid
158.29
142.42
142.34
139.63
128.60
127.88
126.56
115.15
110.23
105.27
77.16 CDCl3
68.20
57.15
41.18
40.71
30.21
30
OH
O
f1 (ppm)

Compound 31

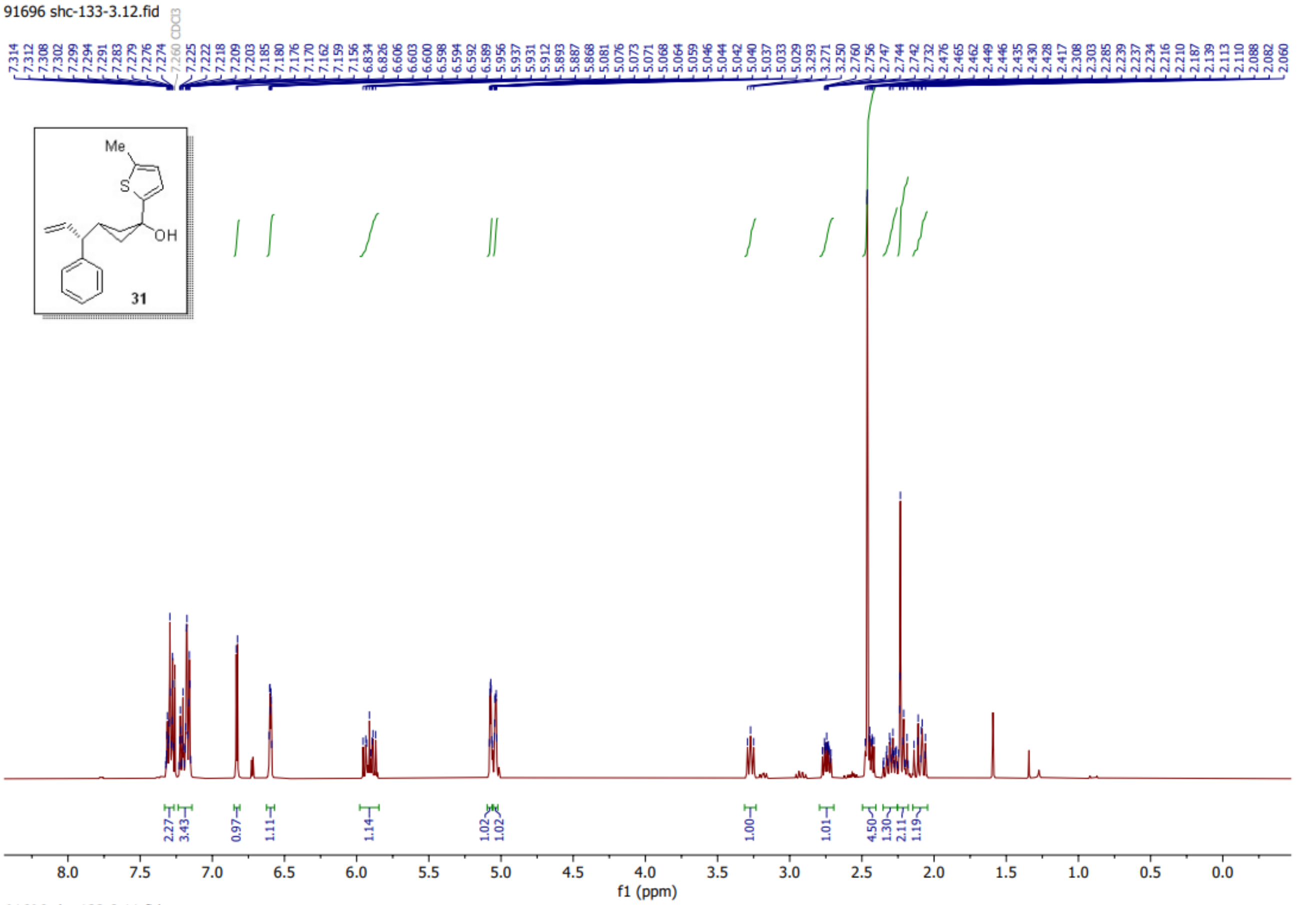
91696 shc-133-3.12.fid
Me
S
OH
31
f1 (ppm)

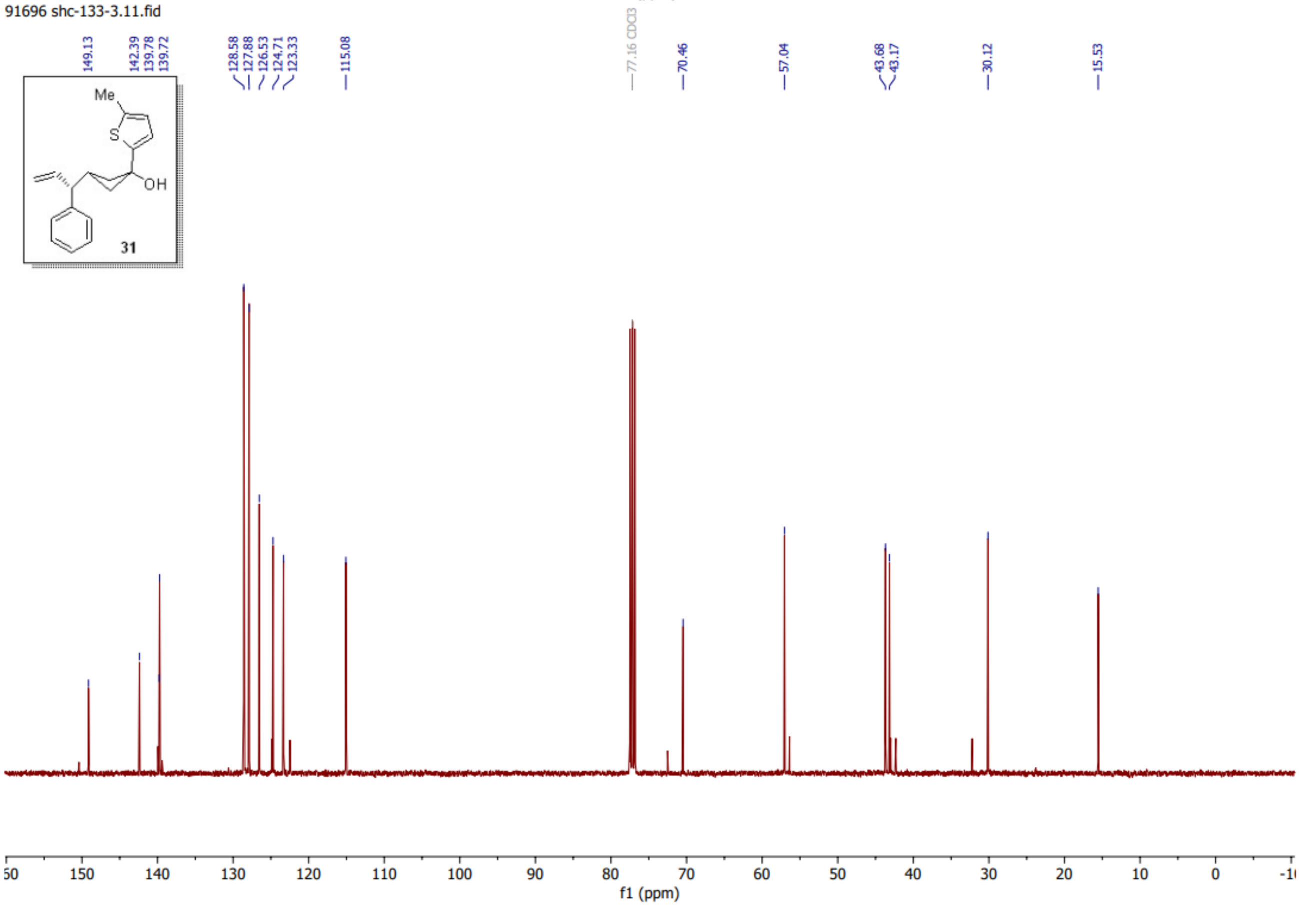
91696 shc-133-3.11.fid
149.13
142.39
139.78
139.72
128.58
127.88
126.53
124.71
123.33
115.08
77.16 CDCl3
70.46
57.04
43.68
43.17
30.12
15.53
Me
S
OH
31
f1 (ppm)

Compound 32

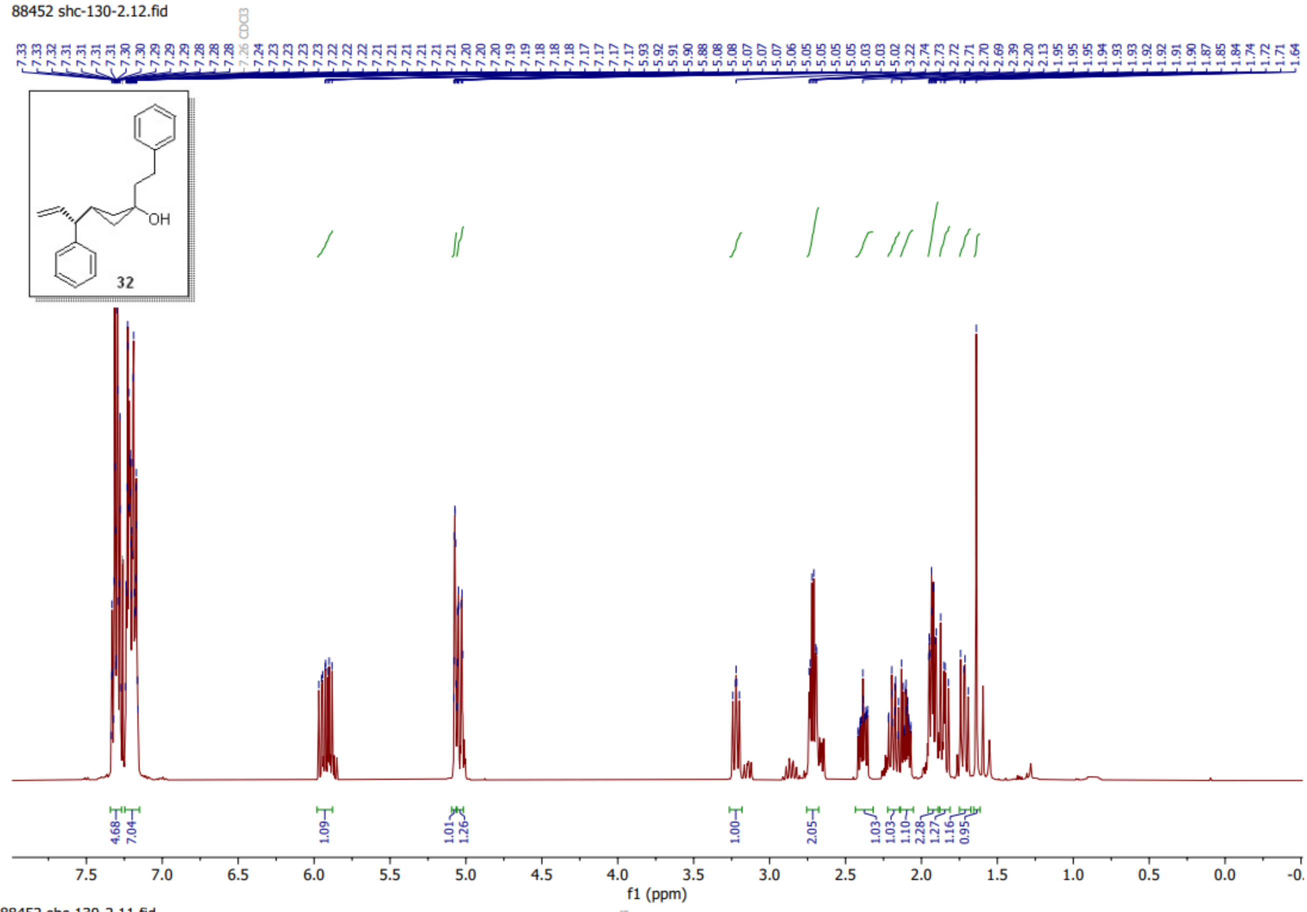
88452 shc-130-2.12.fid
7.33 7.33 7.32 7.31 7.31 7.31 7.31 7.30 7.30 7.29 7.29 7.29 7.28 7.28 7.28 7.26 CDCl3 7.24 7.23 7.23 7.23 7.23 7.22 7.22 7.22 7.21 7.21 7.21 7.21 7.21 7.21 7.20 7.20 7.20 7.19 7.19 7.18 7.18 7.18 7.17 7.17 7.17 5.93 5.92 5.91 5.90 5.88 5.08 5.08 5.07 5.07 5.07 5.06 5.05 5.05 5.05 5.05 5.03 5.03 5.02 3.22 2.74 2.73 2.72 2.71 2.70 2.69 2.39 2.20 2.13 1.95 1.95 1.95 1.94 1.93 1.93 1.92 1.92 1.91 1.90 1.87 1.85 1.84 1.74 1.72 1.71 1.64
OH
32
4.68 7.04 1.09 1.01 1.26 1.00 2.05 1.03 1.03 1.10 2.28 1.27 1.16 0.95
7.5 7.0 6.5 6.0 5.5 5.0 4.5 4.0 3.5 3.0 2.5 2.0 1.5 1.0 0.5 0.0
f1 (ppm)

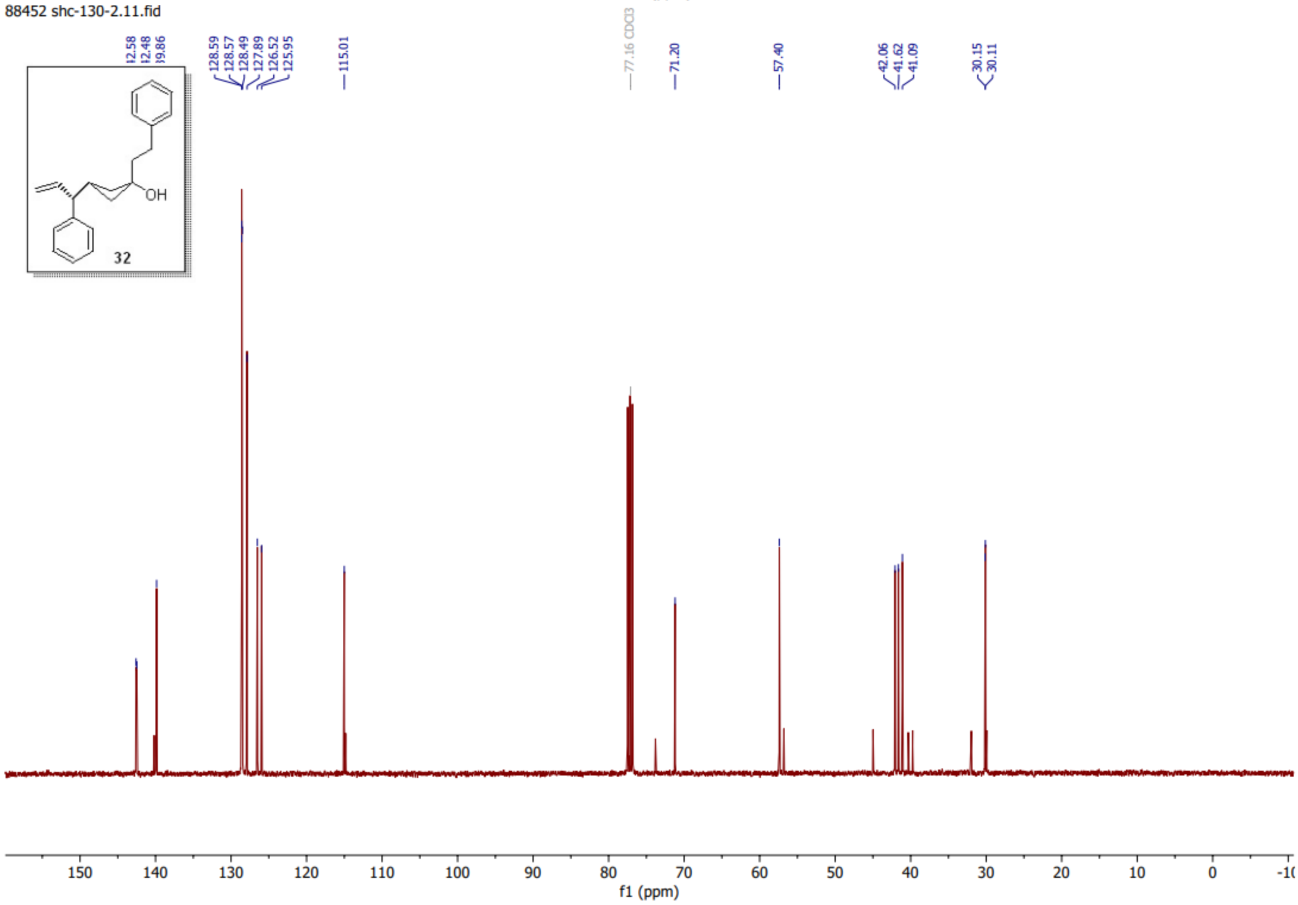
88452 shc-130-2.11.fid
12.58 12.48 19.86
128.59 128.57 128.49 127.89 126.52 125.95
115.01
77.16 CDCl3
71.20
57.40
42.06 41.62 41.09
30.15 30.11
OH
32
150 140 130 120 110 100 90 80 70 60 50 40 30 20 10 0
f1 (ppm)

Compound 33

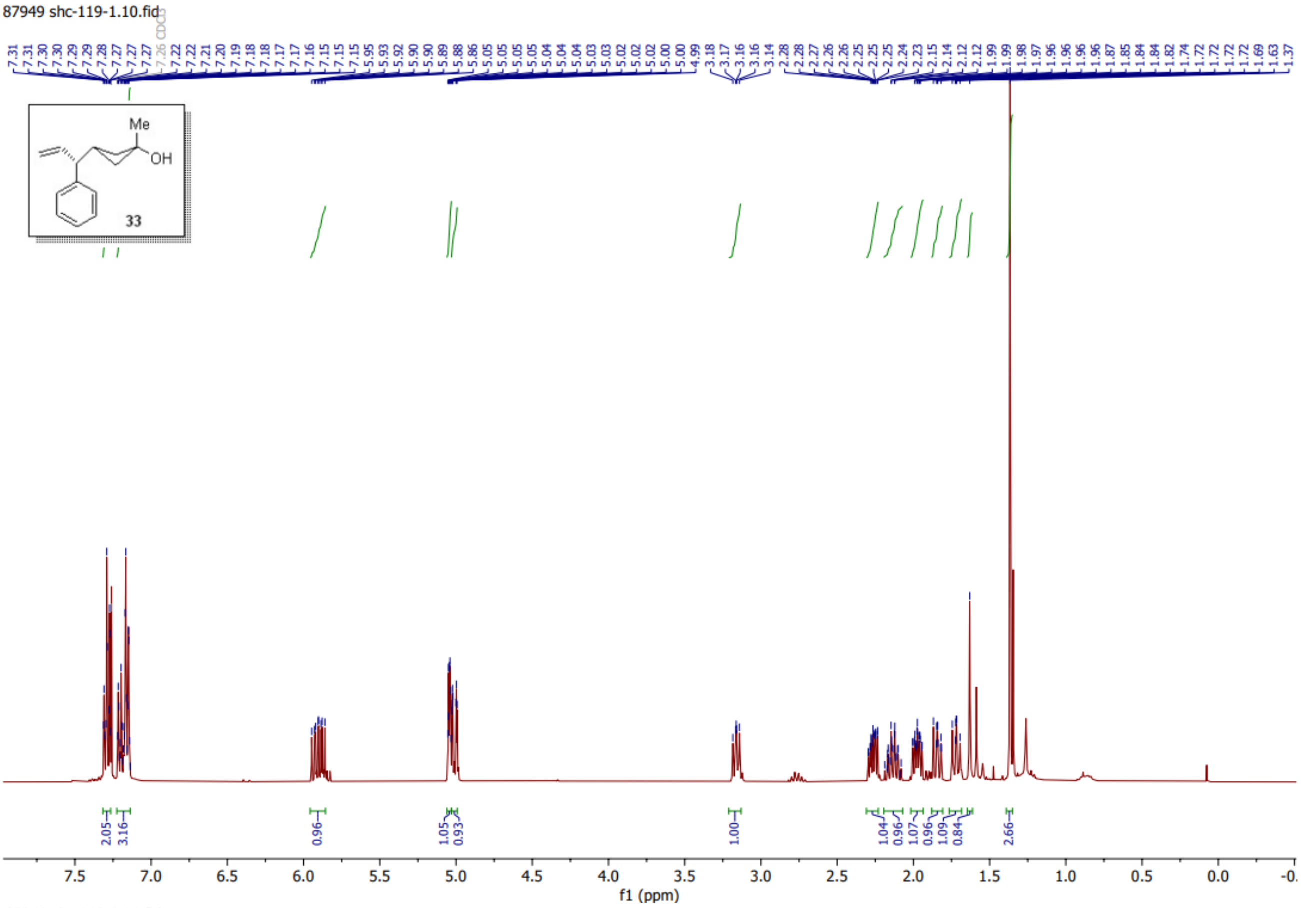
87949 shc-119-1.10.fid
Me
OH
33
f1 (ppm)

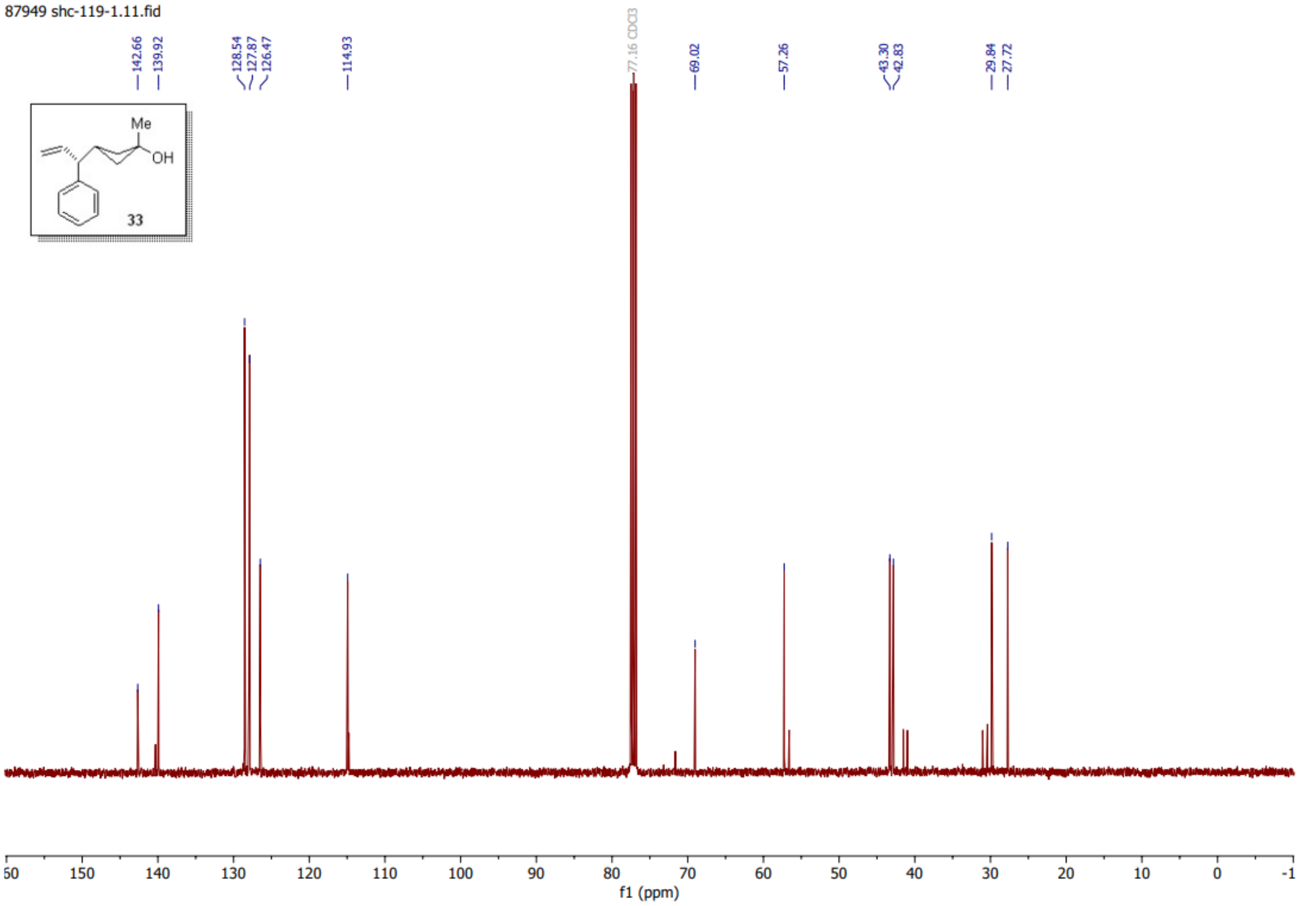
87949 shc-119-1.11.fid
142.66
139.92
128.54
127.87
126.47
114.93
77.16 CDCl3
69.02
57.26
43.30
42.83
29.84
27.72
Me
OH
33
f1 (ppm)

Compound 34

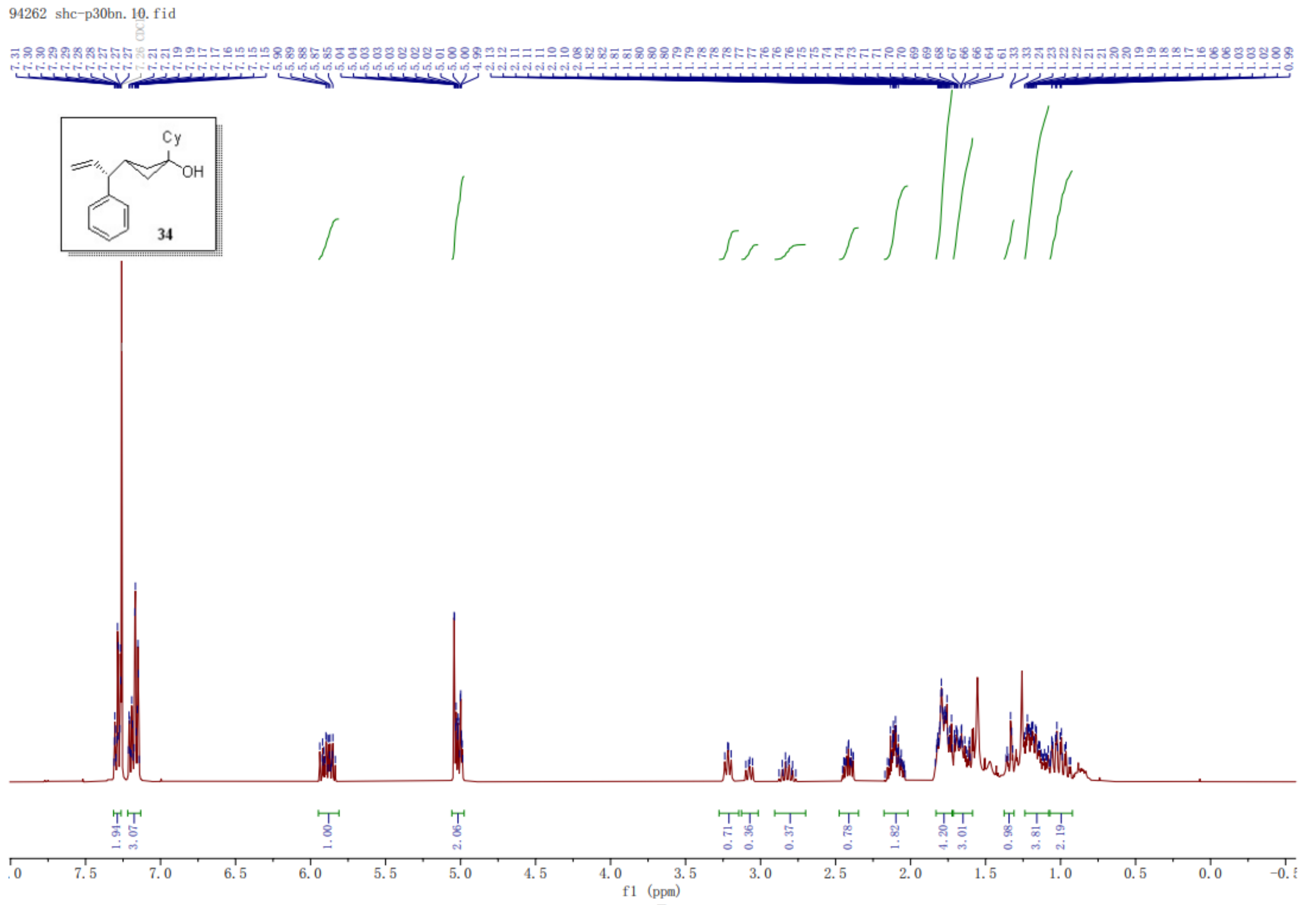
94262 shc-p30bn.10.fid
34
Cy
OH
f1 (ppm)

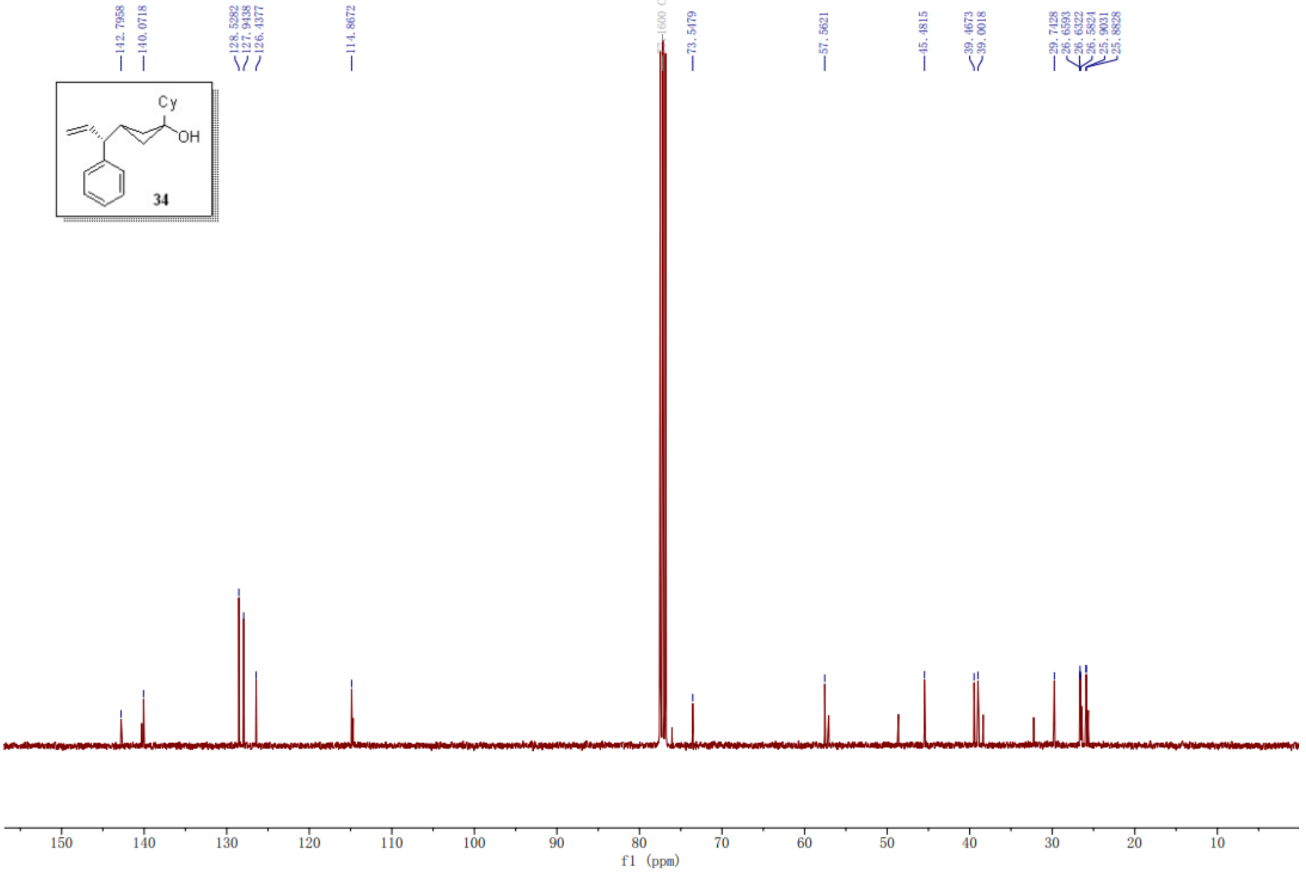
142.7958
140.0718
128.5282
127.9438
126.4377
114.8672
73.5479
57.5621
45.4815
39.4673
39.0018
29.7428
26.6593
26.6322
26.5824
25.9031
25.8828
34
Cy
OH
f1 (ppm)

Compound 35

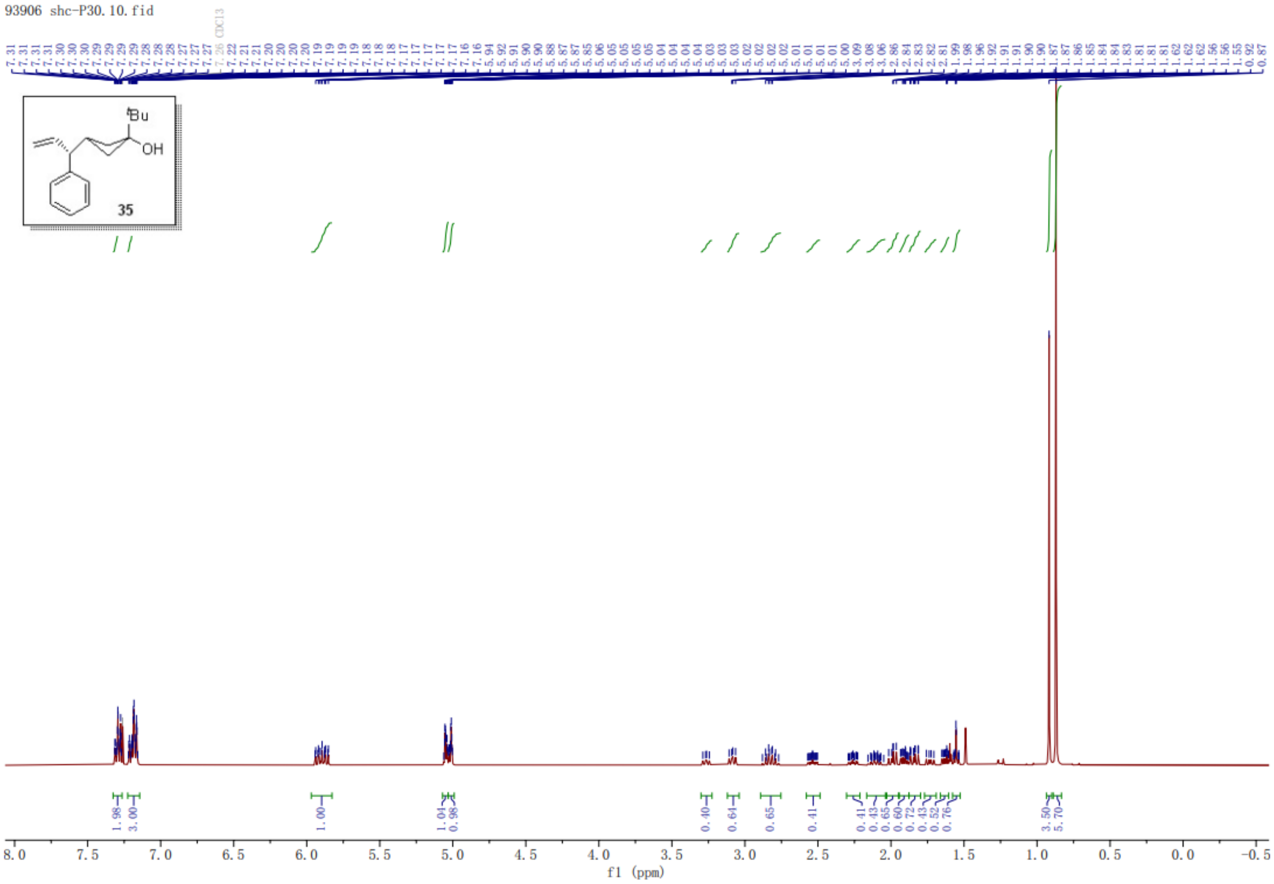

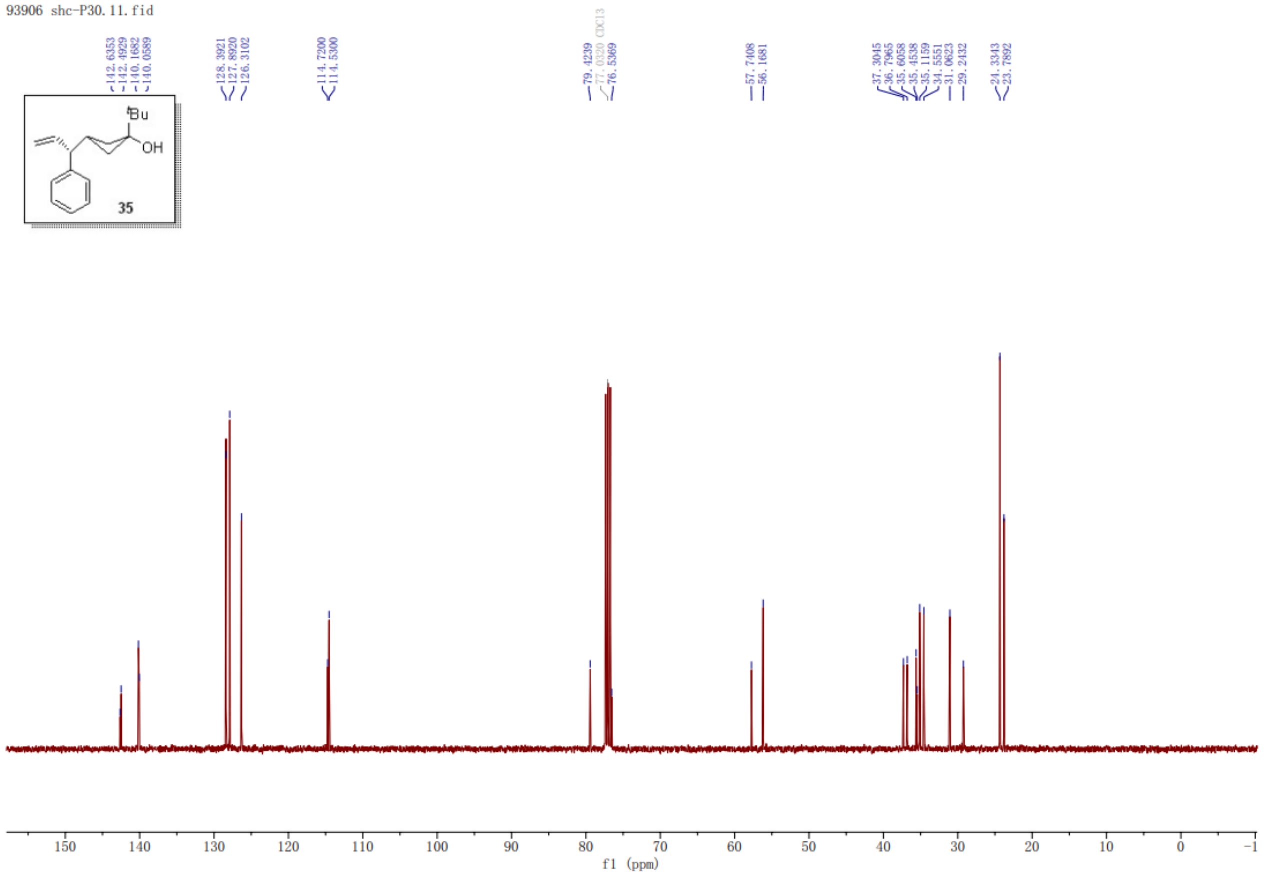

Compound 36

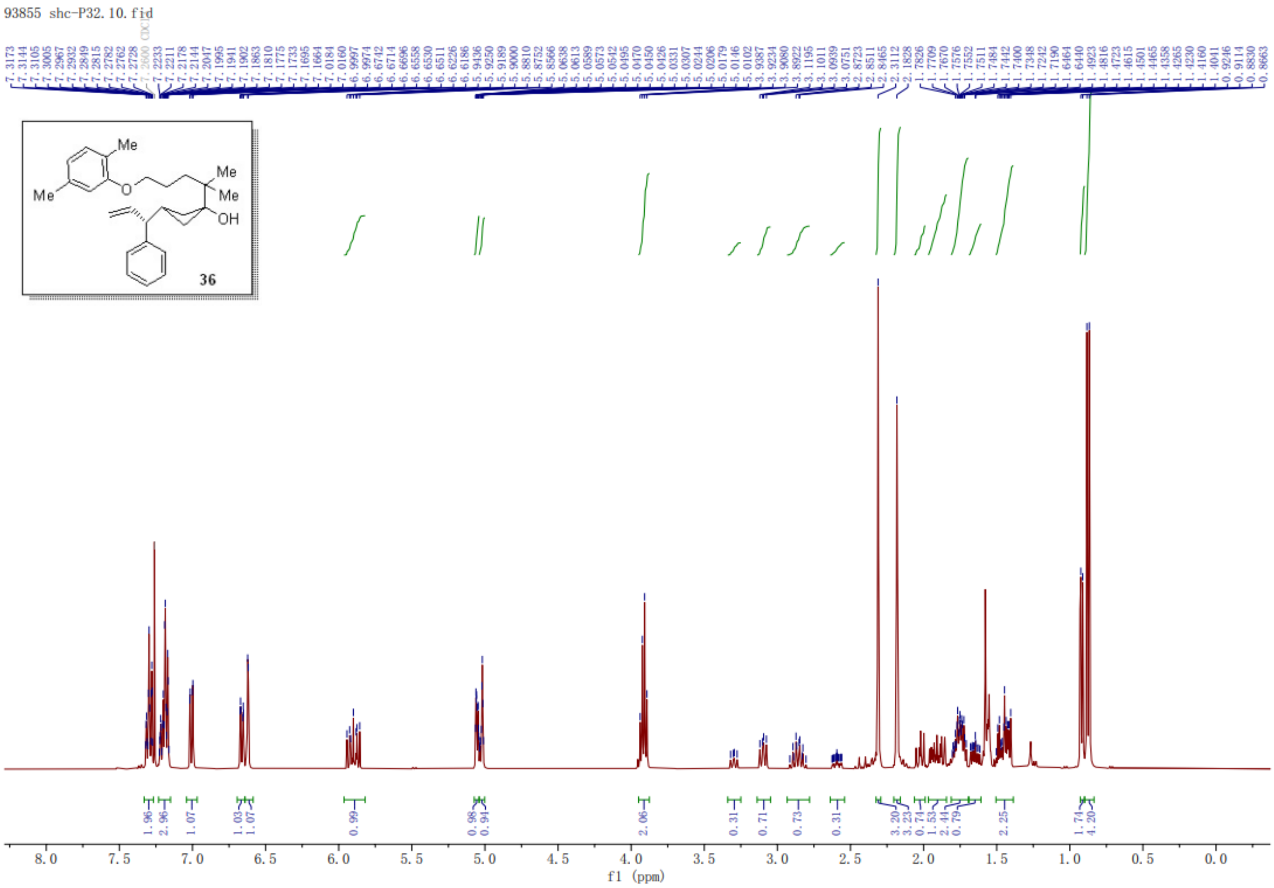

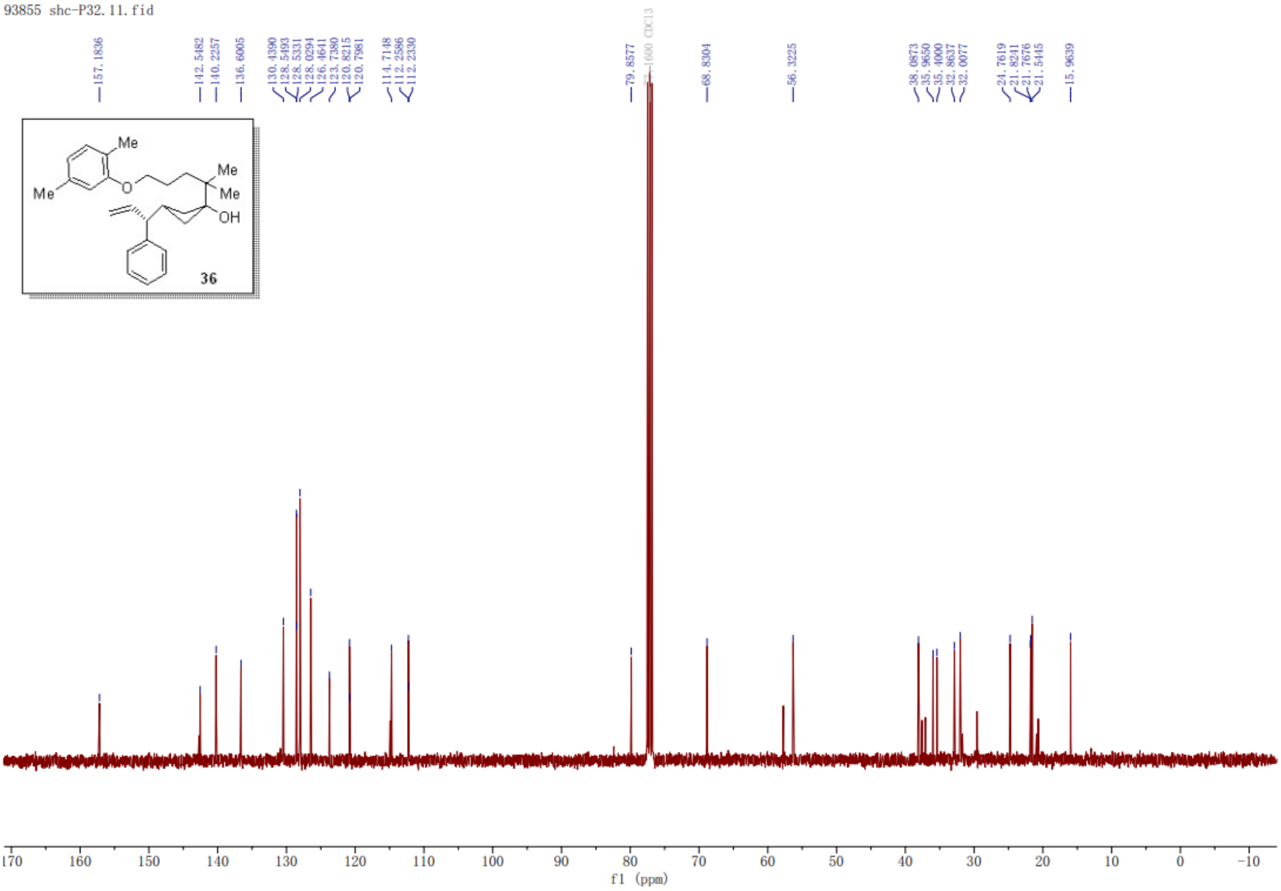

Compound 37

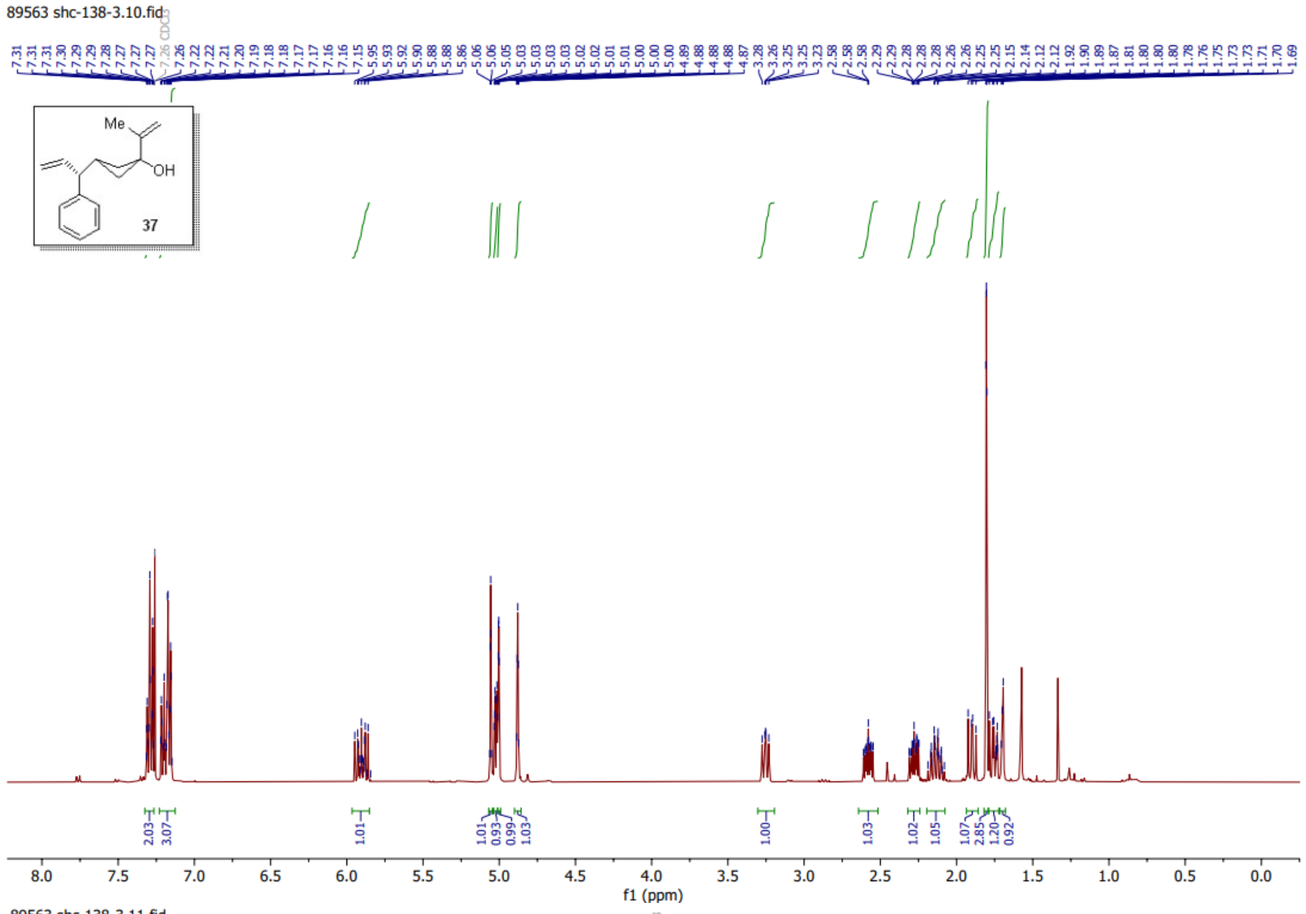
89563 shc-138-3.10.fid
Me
OH
37
f1 (ppm)

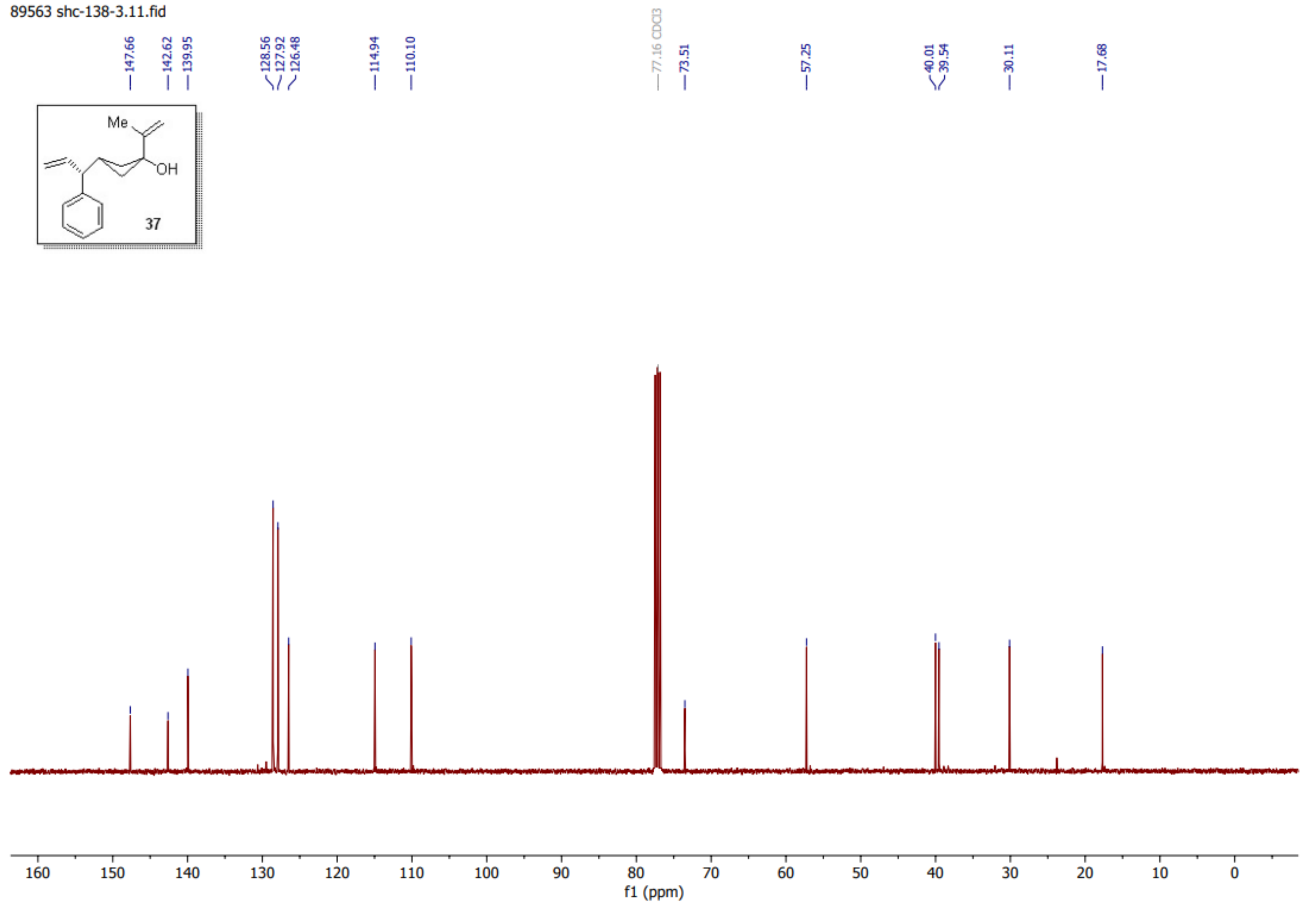
89563 shc-138-3.11.fid
147.66
142.62
139.95
128.56
127.92
126.48
114.94
110.10
77.16 CDCl3
73.51
57.25
40.01
39.54
30.11
17.68
Me
OH
37
f1 (ppm)

Compound 38

89845 shc-138-1m.11.fid

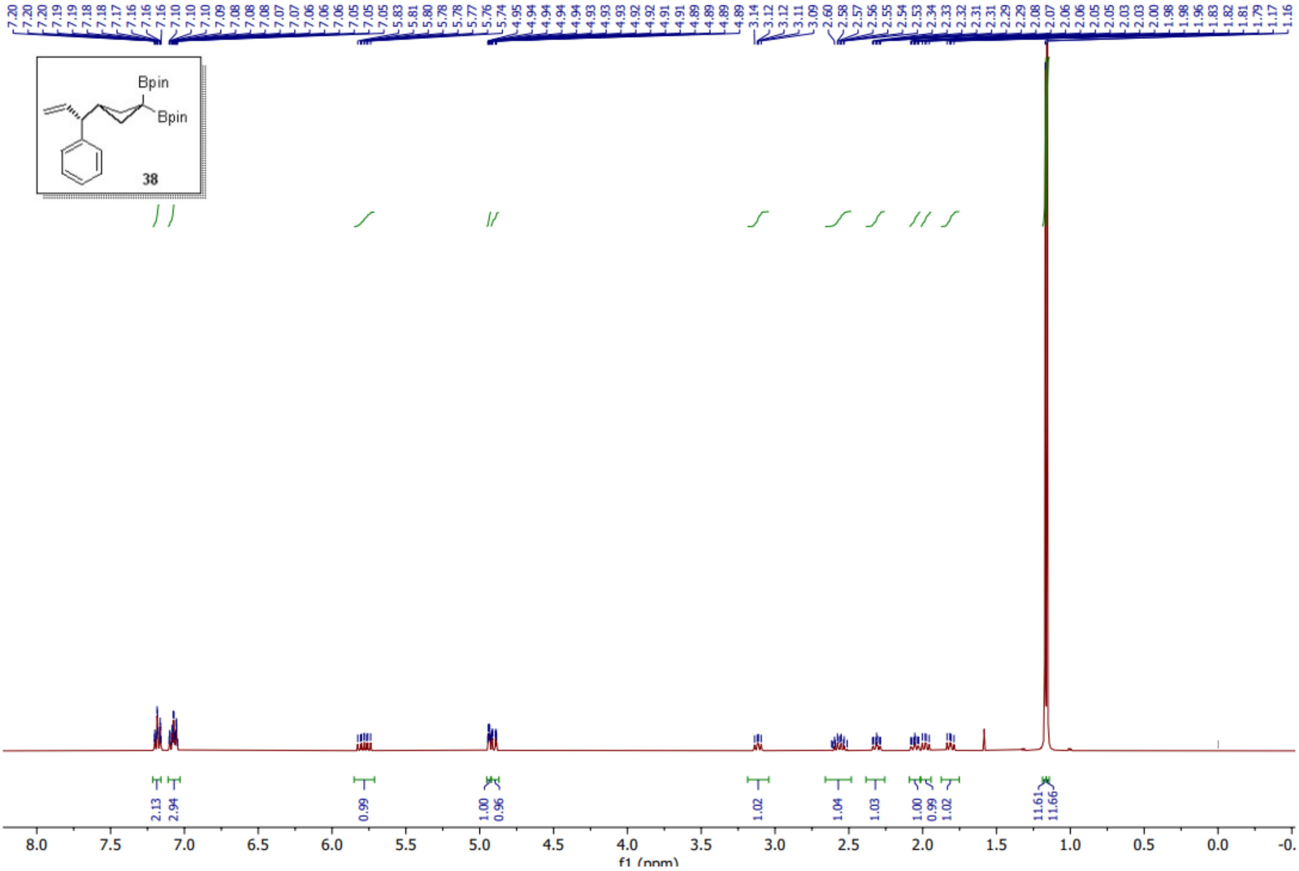

89845 shc-138-1m.12.fid

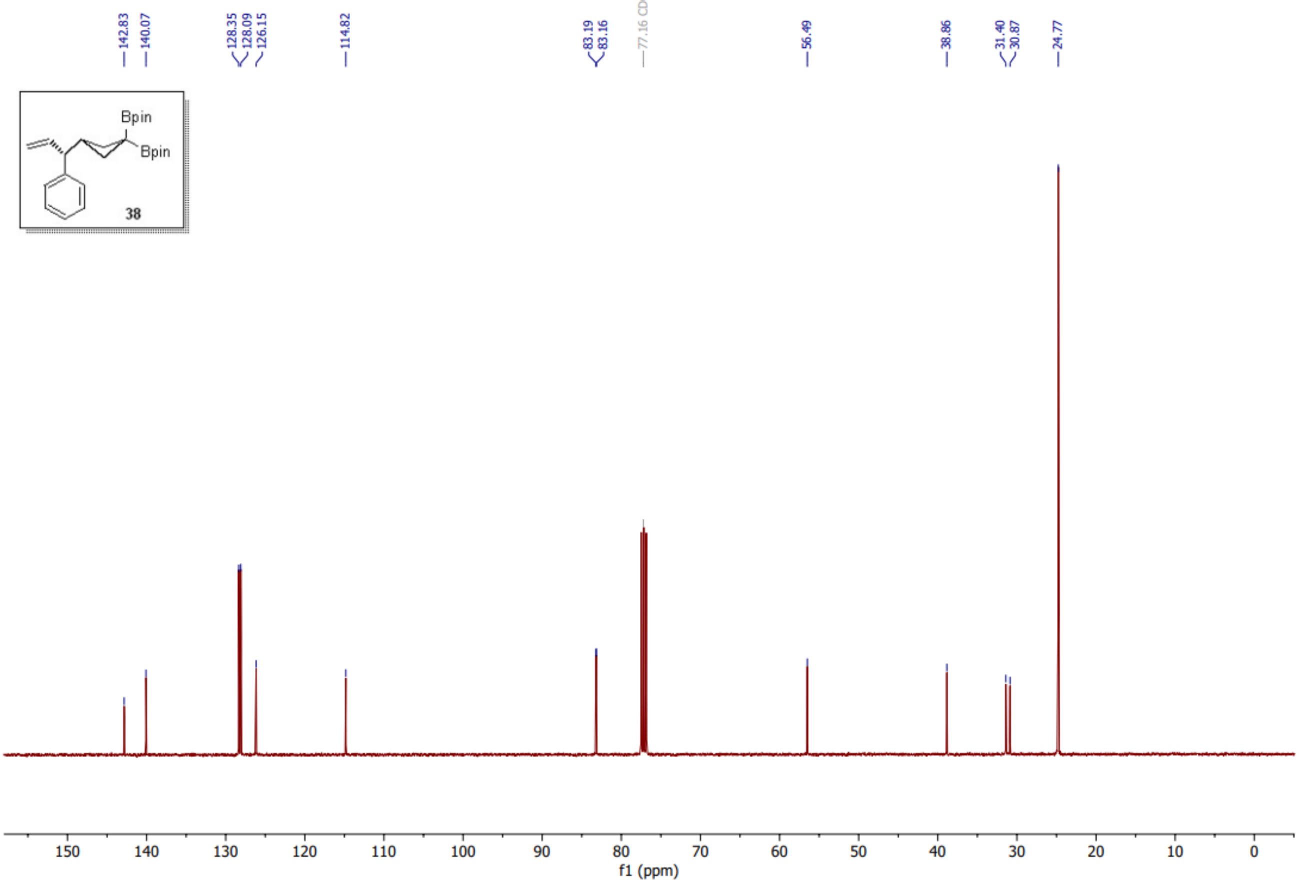

Compound 39

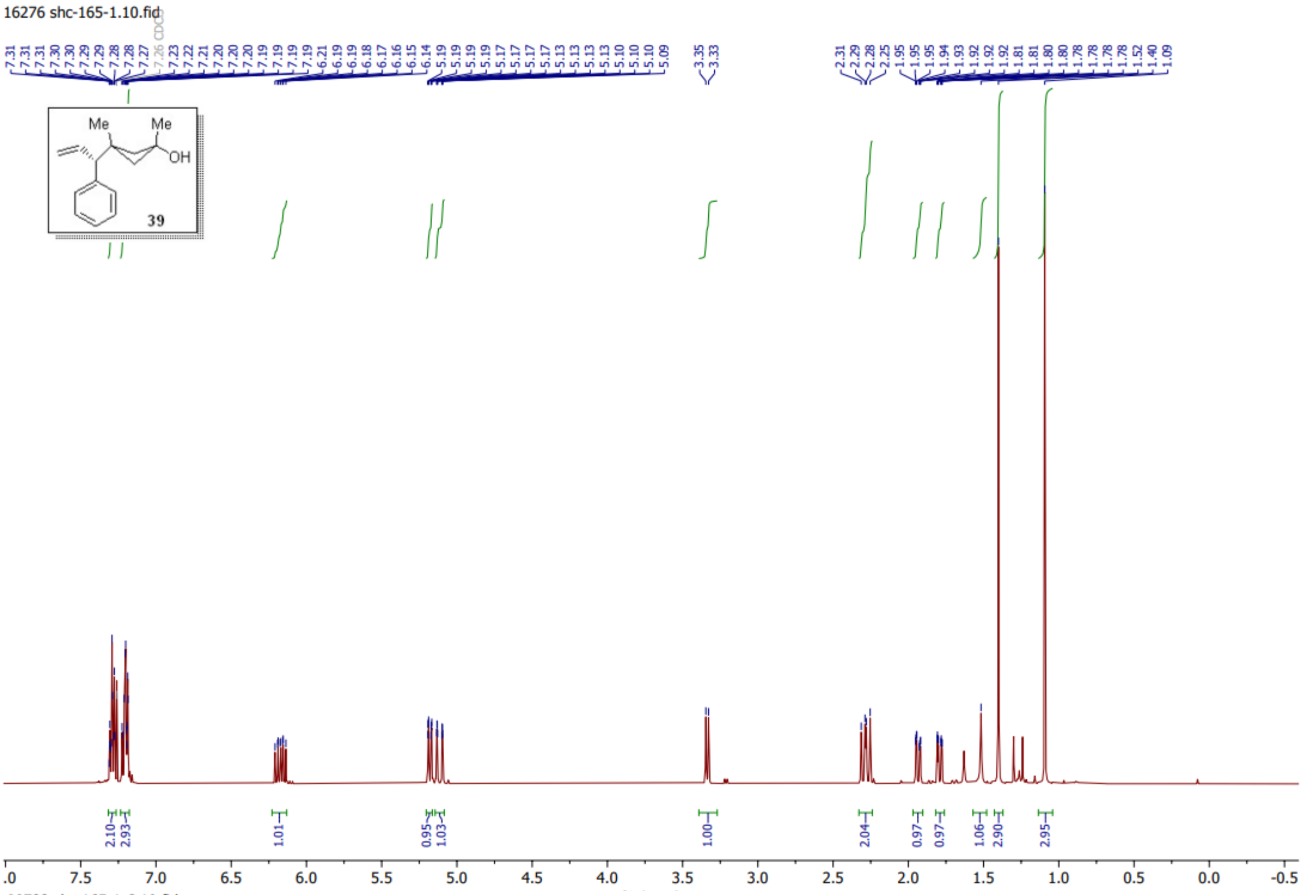
16276 shc-165-1.10.fid
7.31 7.31 7.31 7.30 7.30 7.29 7.29 7.28 7.28 7.27 7.26 CDCl3 7.23 7.22 7.21 7.21 7.20 7.20 7.20 7.19 7.19 7.19 7.19 6.21 6.19 6.19 6.18 6.17 6.16 6.15 6.14 5.19 5.19 5.19 5.19 5.17 5.17 5.17 5.17 5.13 5.13 5.13 5.13 5.10 5.10 5.10 5.09
3.35 3.33
2.31 2.29 2.28 2.25 1.95 1.95 1.95 1.94 1.93 1.92 1.92 1.92 1.81 1.81 1.80 1.80 1.78 1.78 1.78 1.78 1.52 1.40 1.09
Me
Me
OH
39
2.10 2.93 1.01 0.95 1.03 1.00 2.04 0.97 0.97 1.06 2.90 2.95

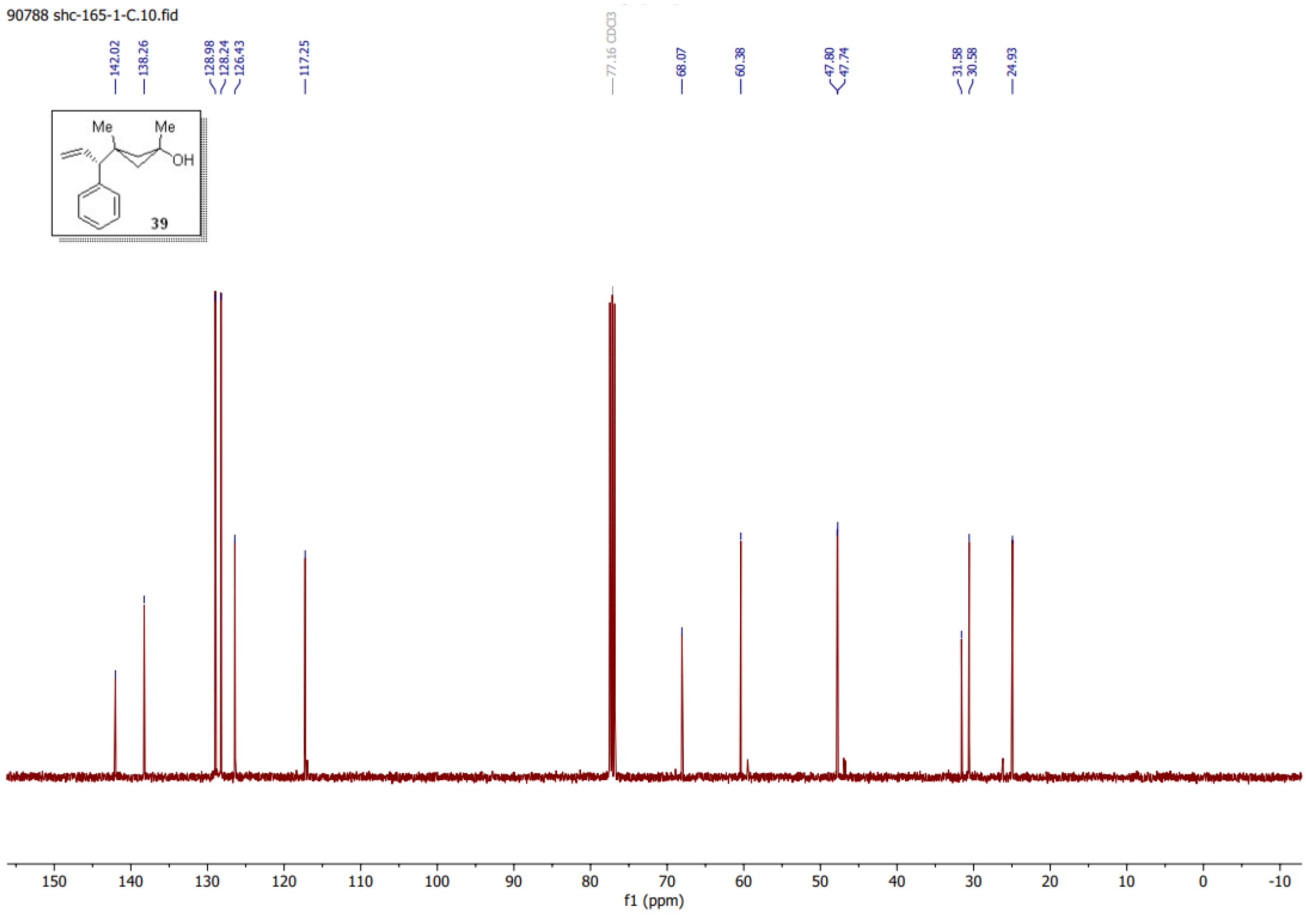
90788 shc-165-1-C.10.fid
142.02
138.26
128.98 128.24 126.43
117.25
77.16 CDCl3
68.07
60.38
47.80 47.74
31.58 30.58
24.93
Me
Me
OH
39
f1 (ppm)

Compound 40

90720 shc-164-1.10.fid

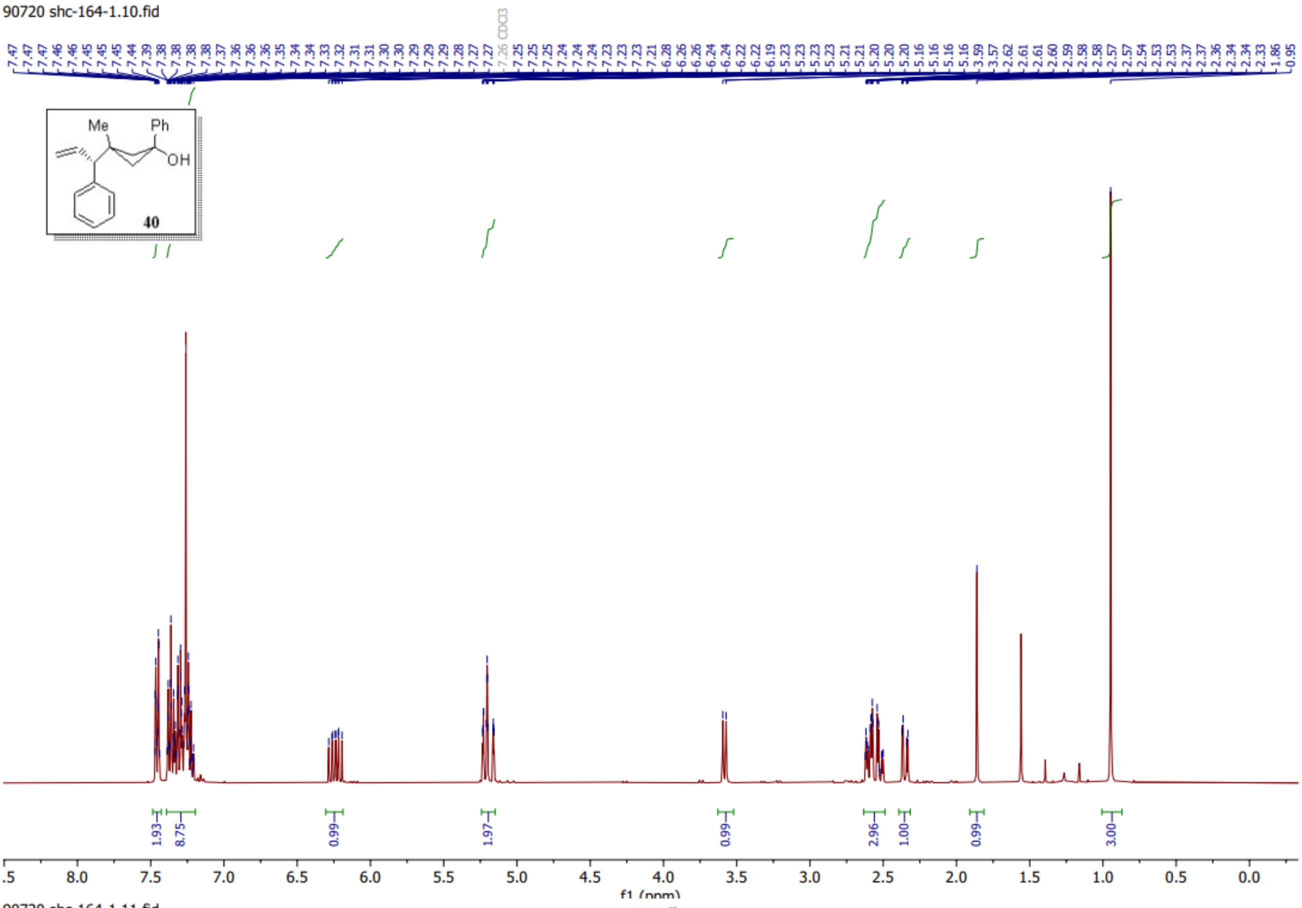

90720 shc-164-1.11.fid

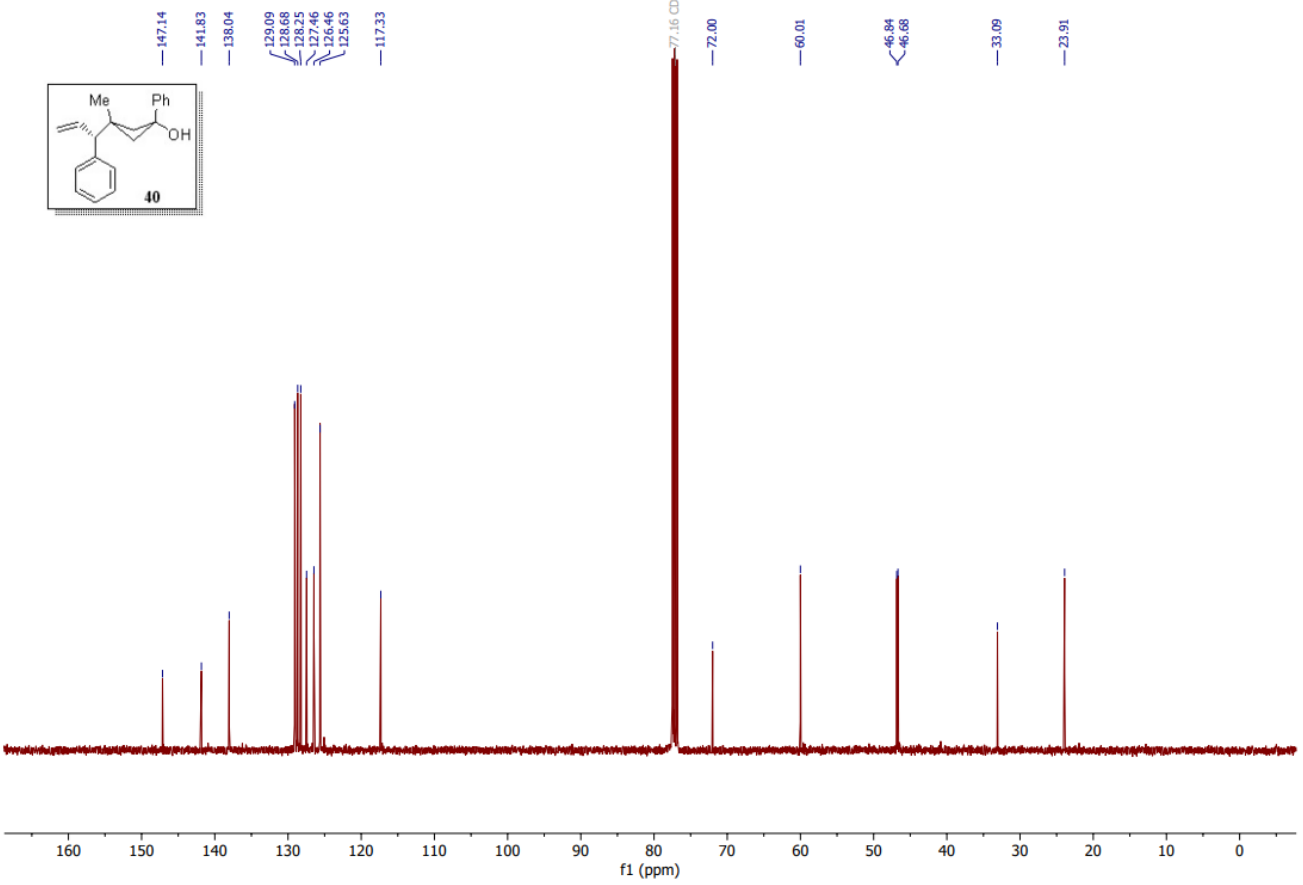

Compound 41

16435 shc-193-p.10.fid

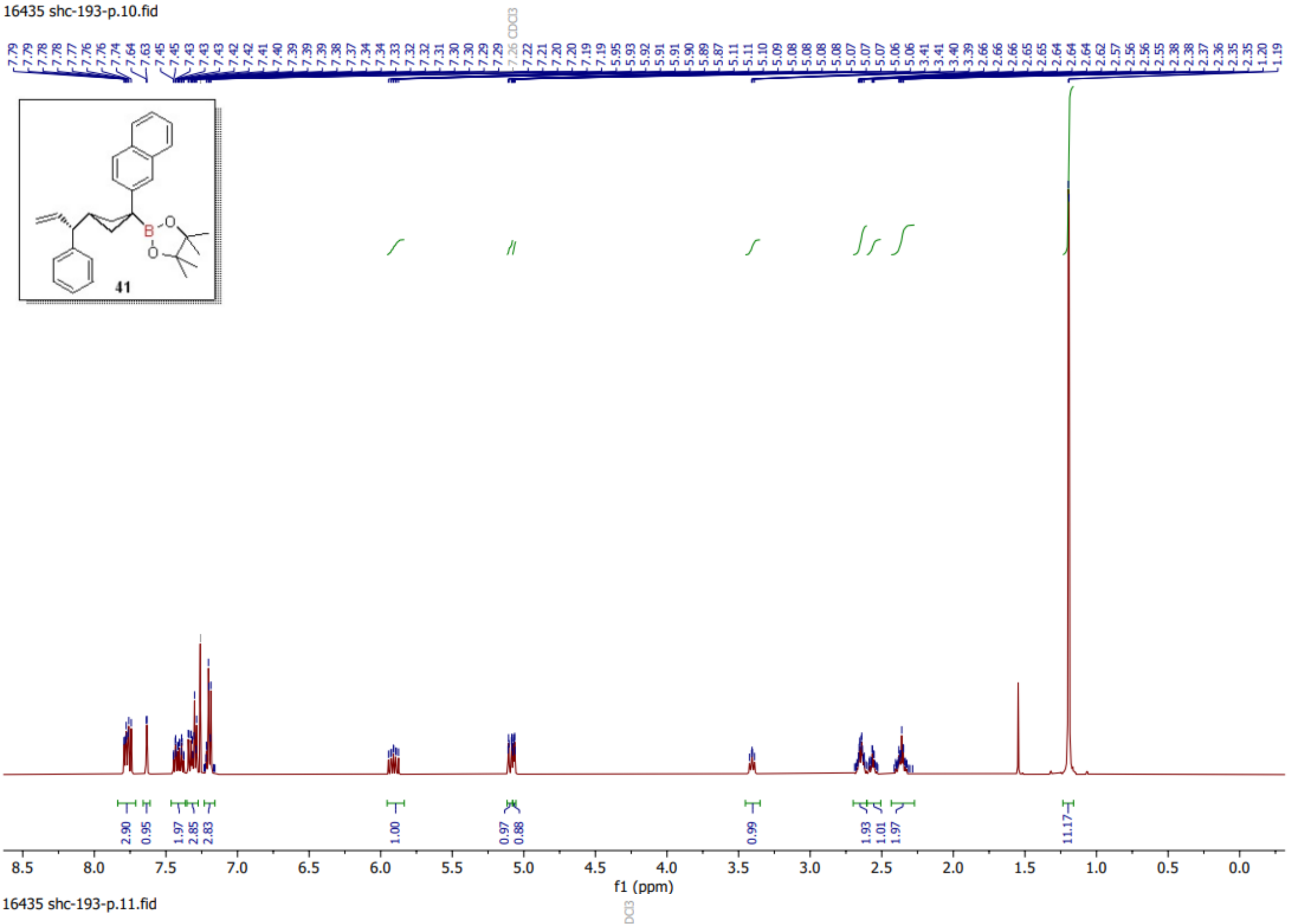

16435 shc-193-p.11.fid

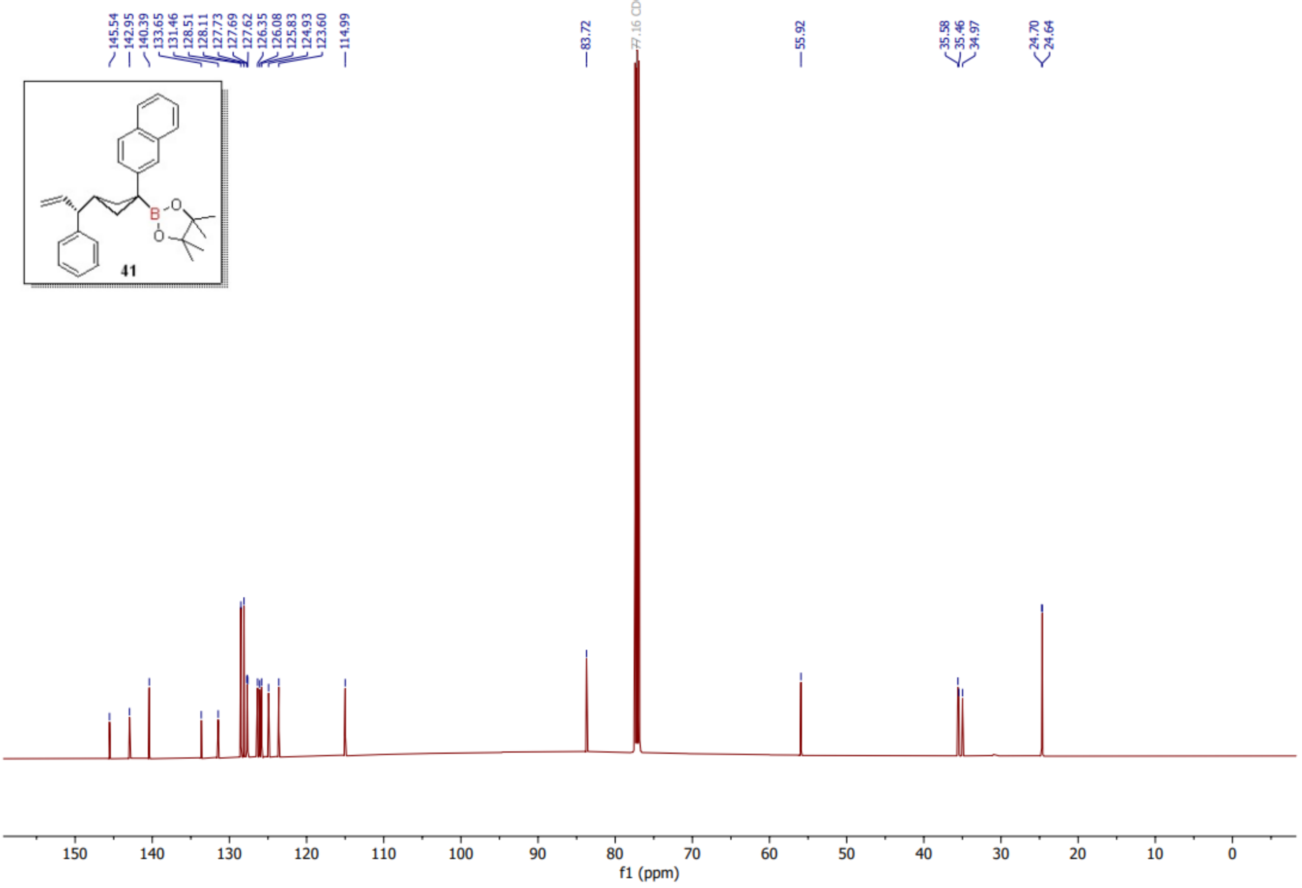

Compound 42

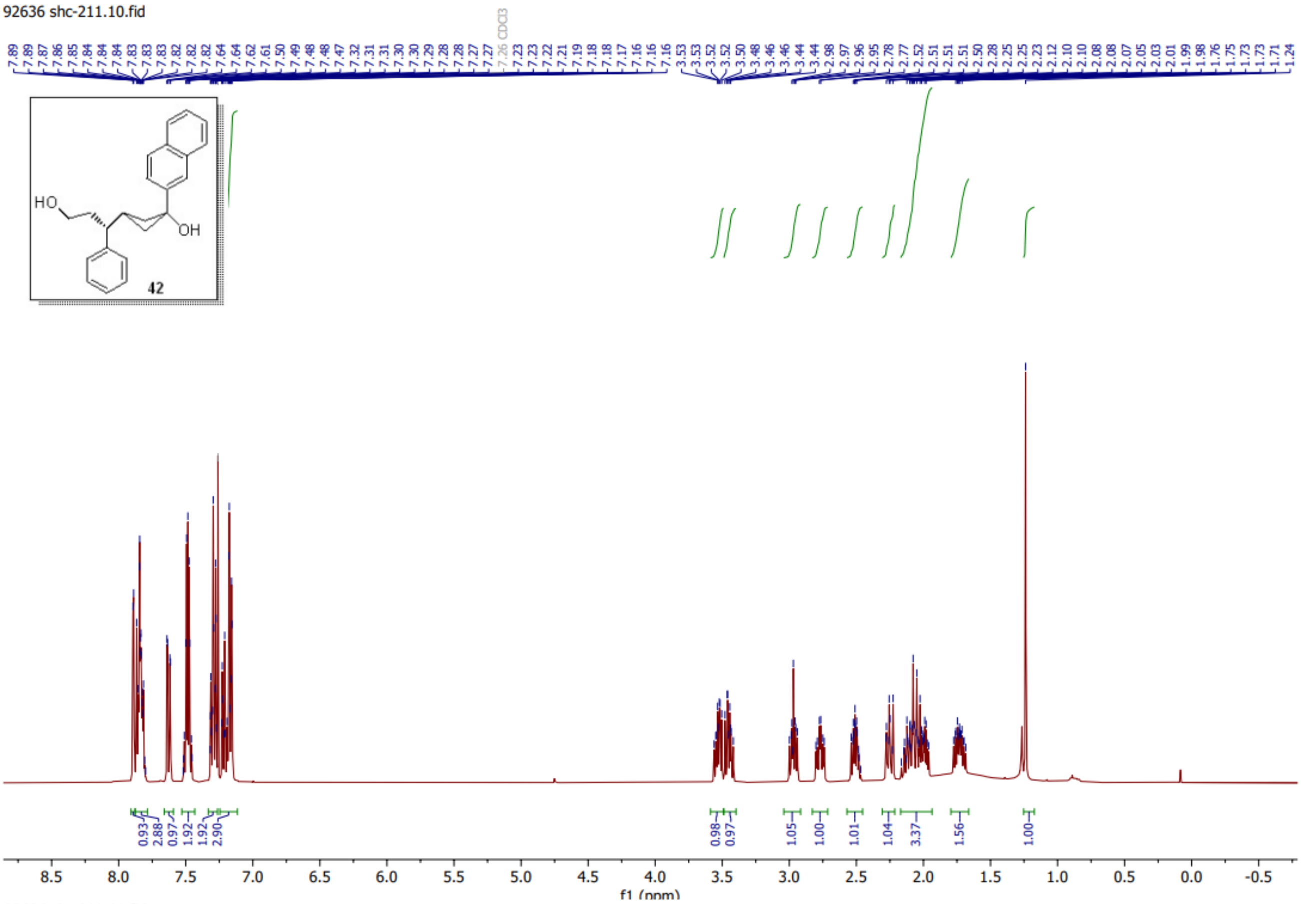

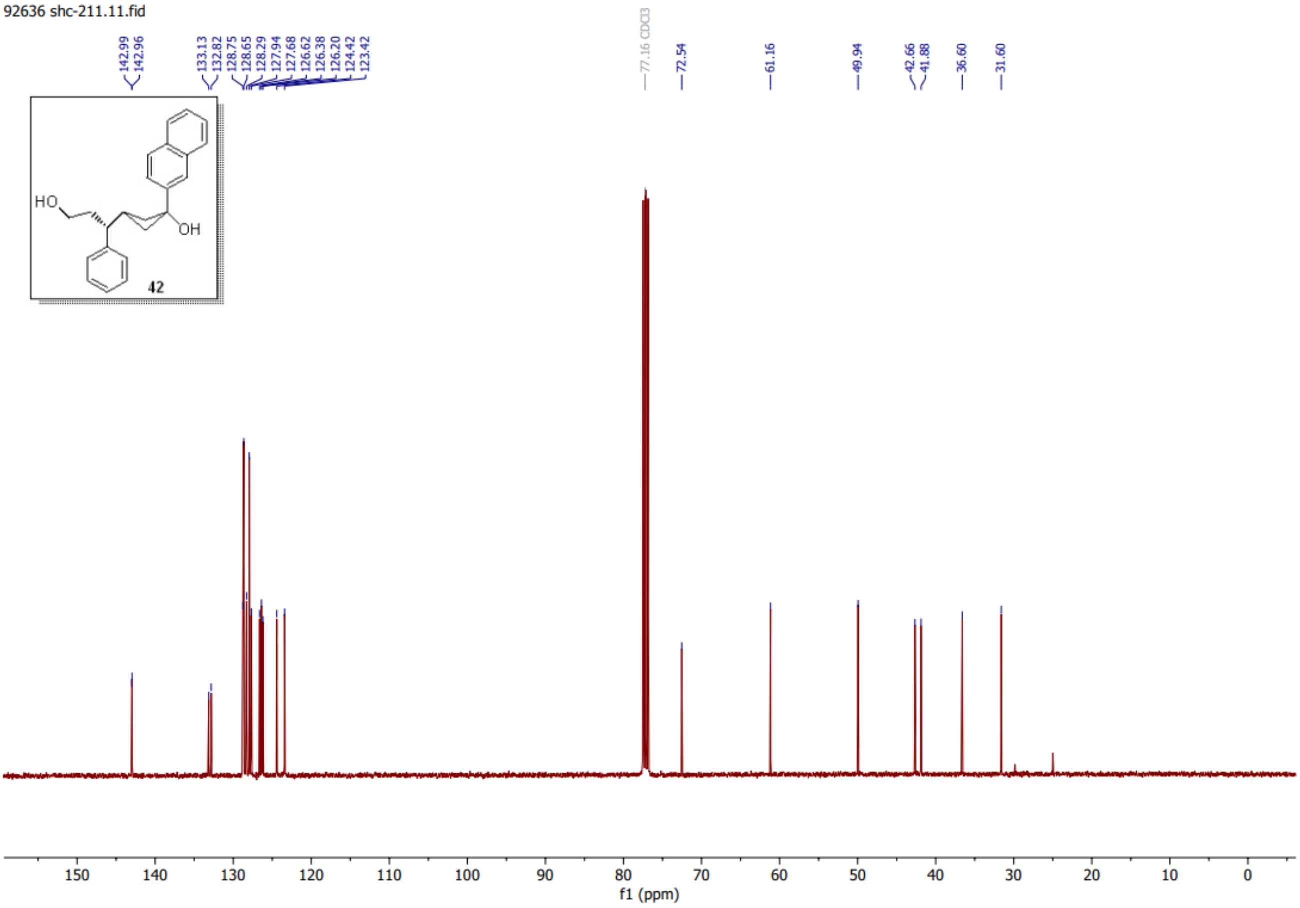

Compound 43

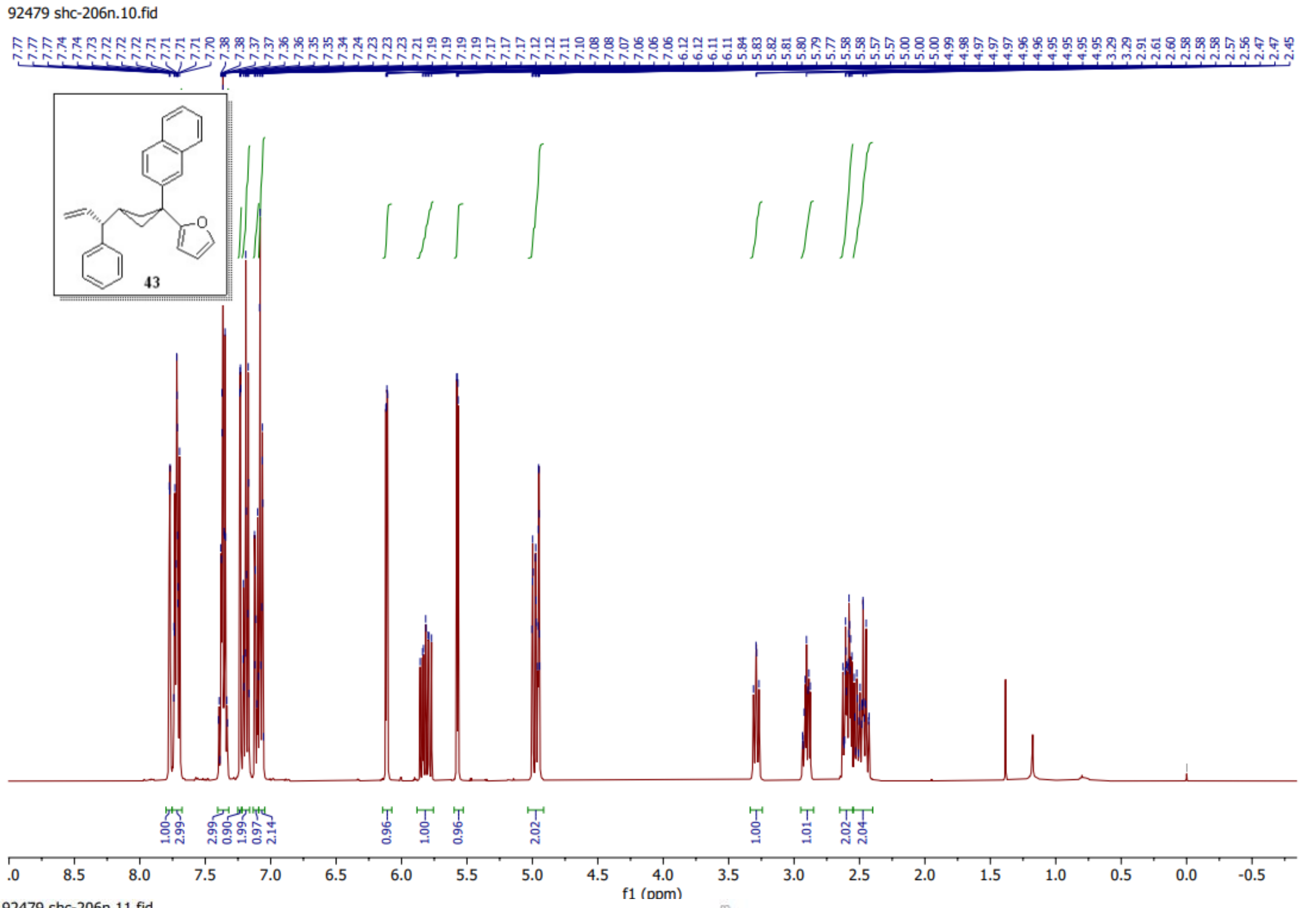

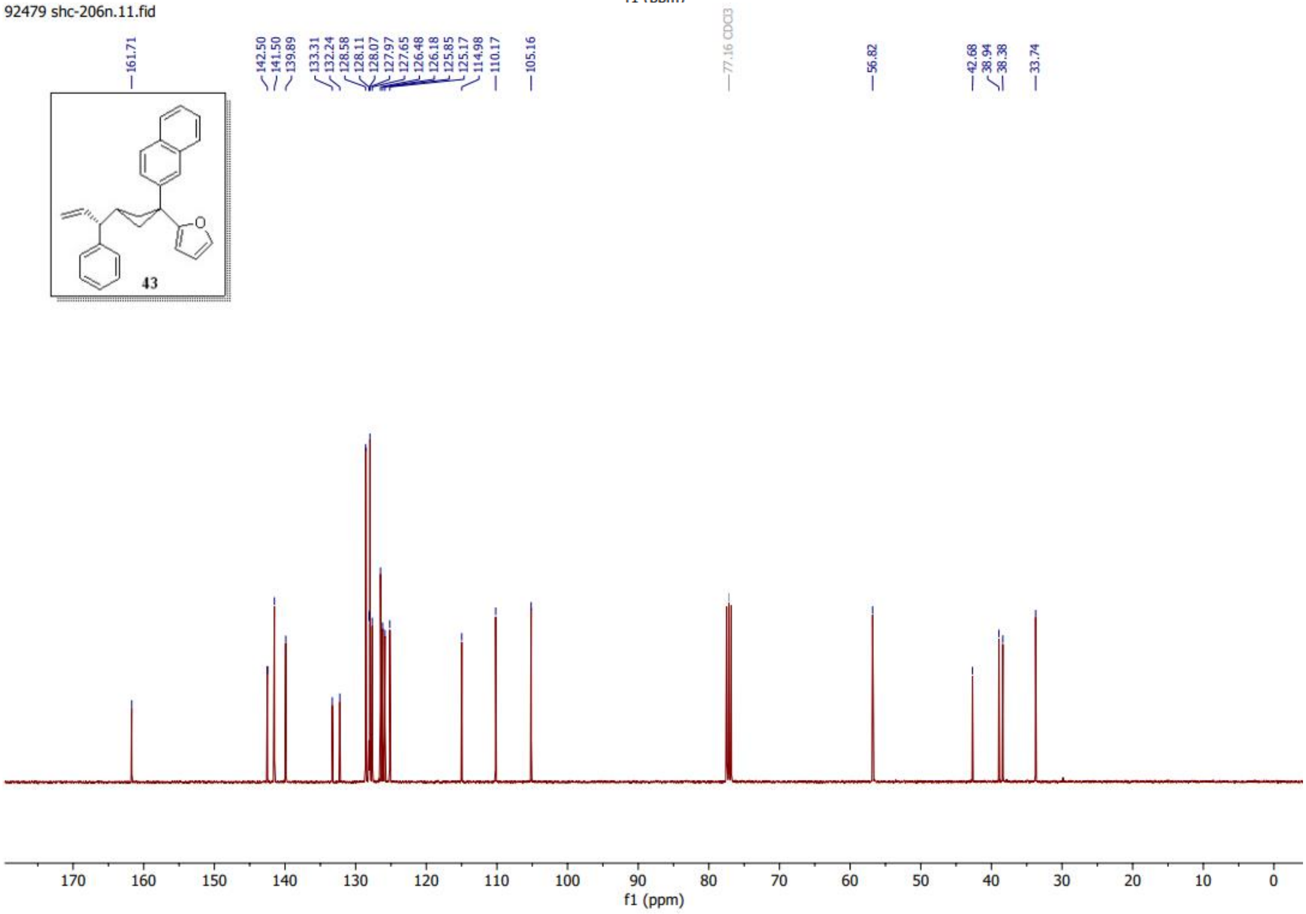

Compound 44

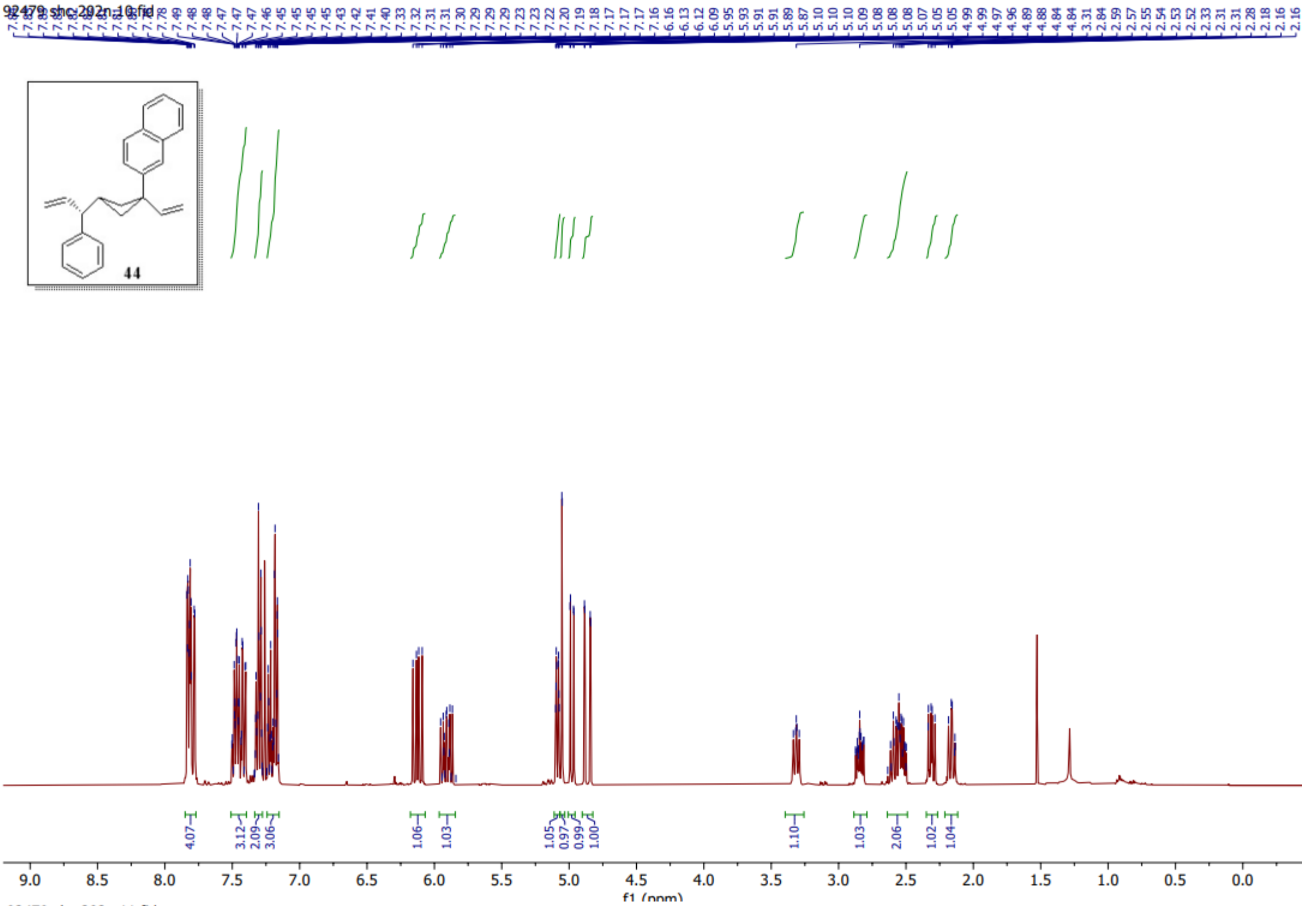
92479 shc-202n.10.fid
44
4.07
3.12
2.09
3.06
1.06
1.03
1.05
0.97
0.99
1.00
1.10
1.03
2.06
1.02
1.04
f1 (ppm)

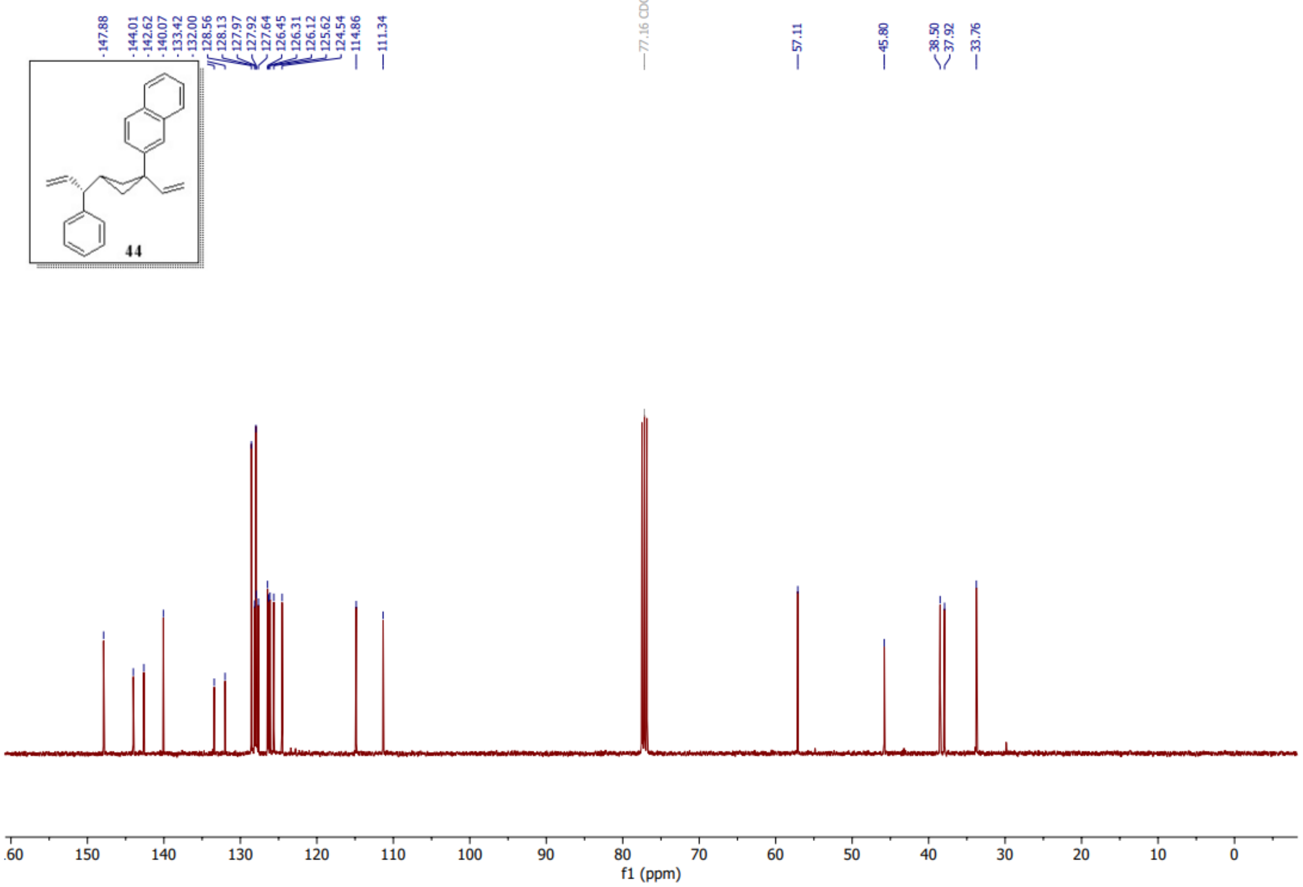
92479 shc-202n.11.fid
147.88
144.01
142.62
140.07
133.42
132.00
128.56
128.13
127.97
127.92
127.64
126.45
126.31
126.12
125.62
124.54
114.86
111.34
77.16 CDCl3
57.11
45.80
38.50
37.92
33.76
44
f1 (ppm)

Compound S3

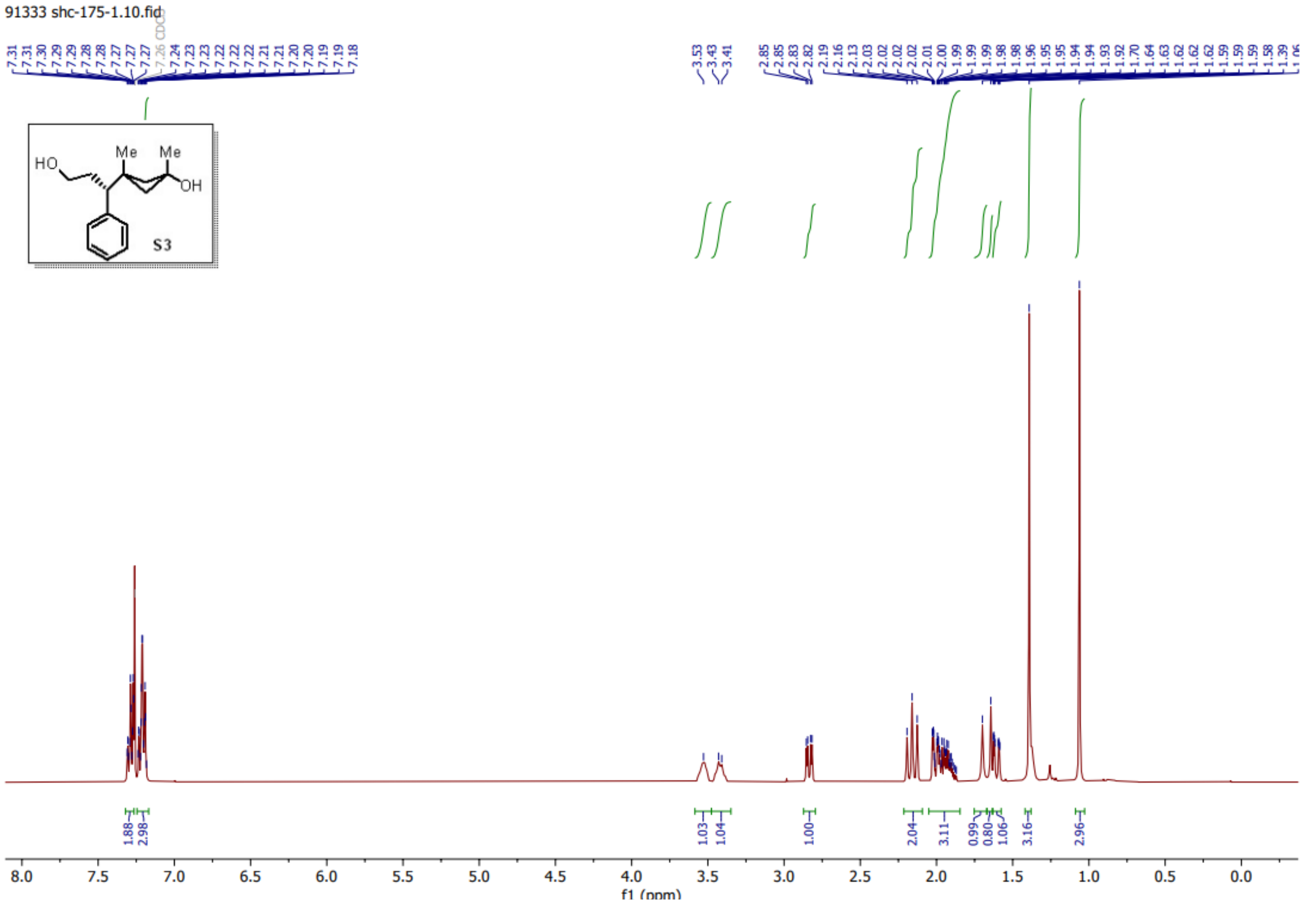
91333 shc-175-1.10.fid
HO
Me
Me
OH
S3
f1 (ppm)

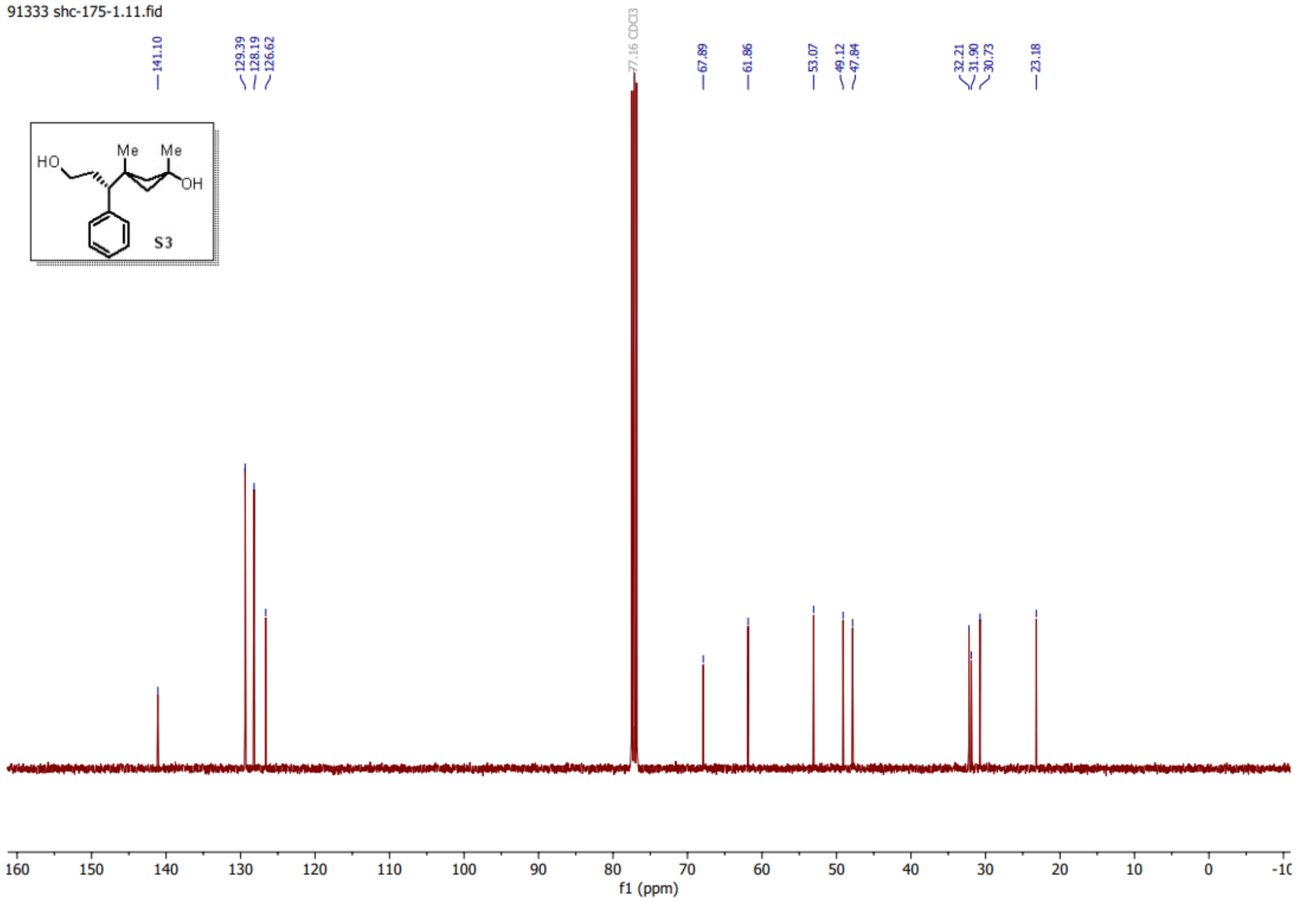
91333 shc-175-1.11.fid
141.10
129.39
128.19
126.62
77.16 CDCl3
67.89
61.86
53.07
49.12
47.84
32.21
31.90
30.73
23.18
HO
Me
Me
OH
S3
f1 (ppm)

Compound S4

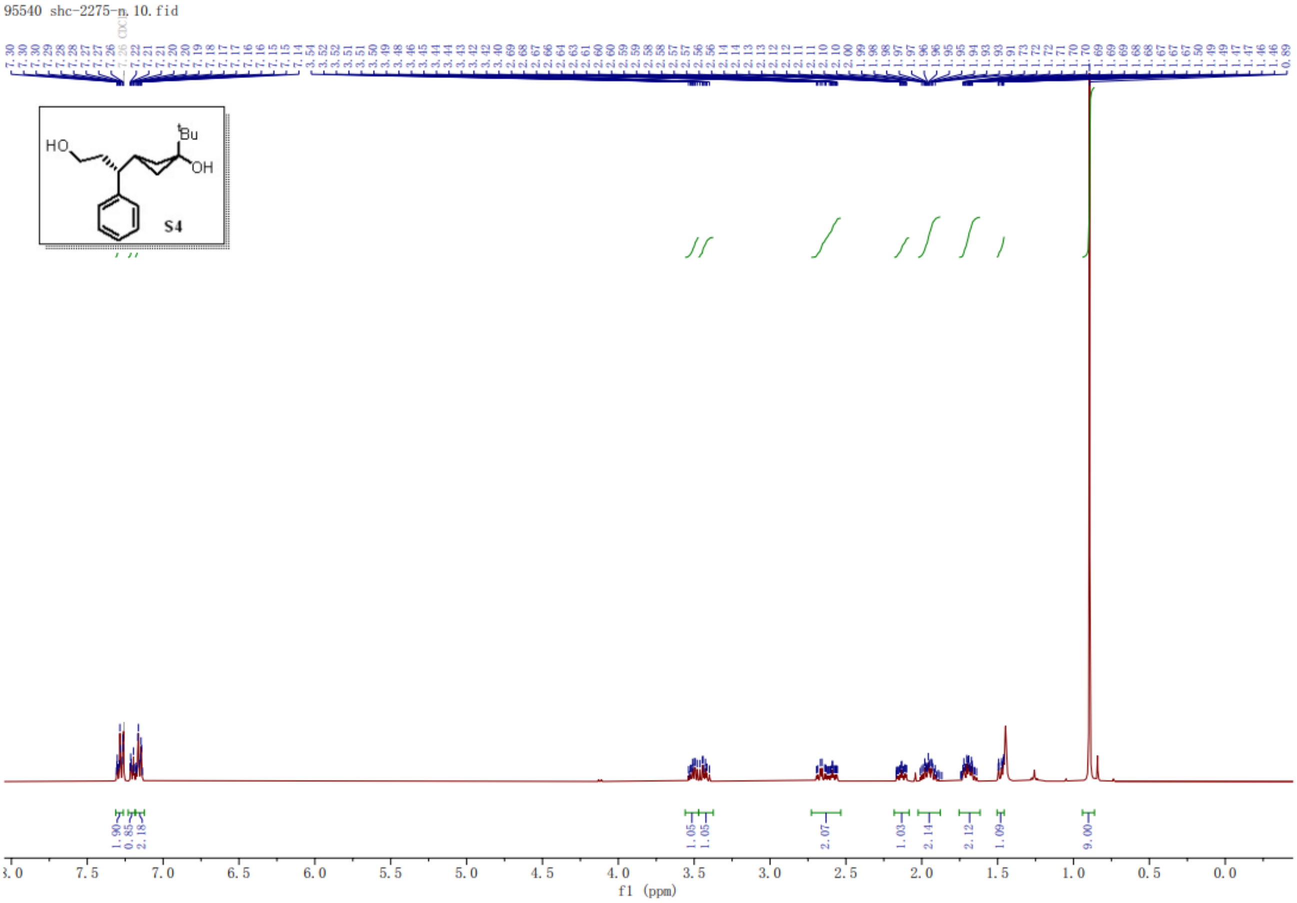
95540 shc-2275-n.10.fid
HO
tBu
OH
S4
f1 (ppm)

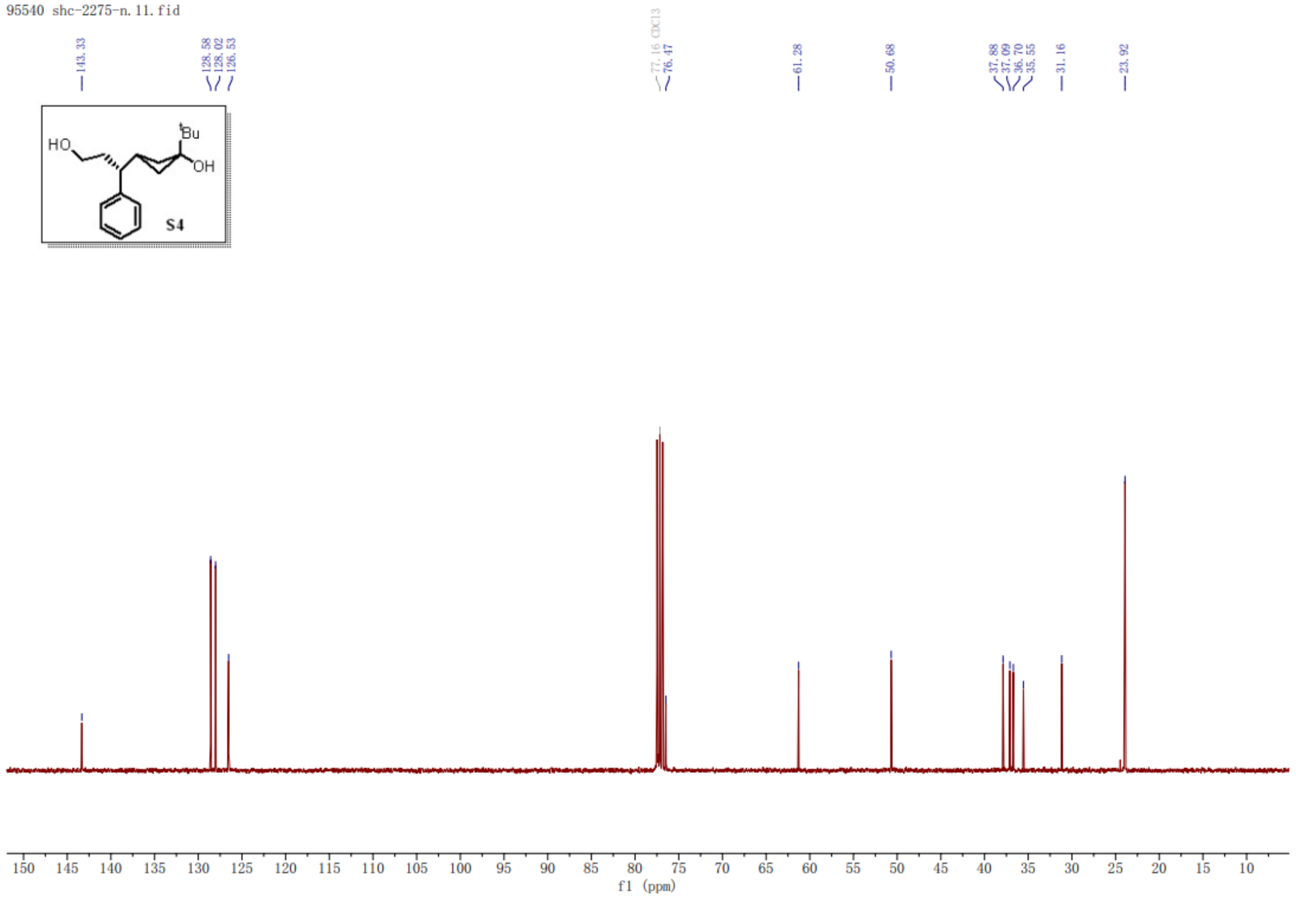
95540 shc-2275-n.11.fid
143.33
128.58
128.02
126.53
77.16 CDCl3
76.47
61.28
50.68
37.88
37.09
36.70
35.55
31.16
23.92
HO
tBu
OH
S4
f1 (ppm)

Compound S5

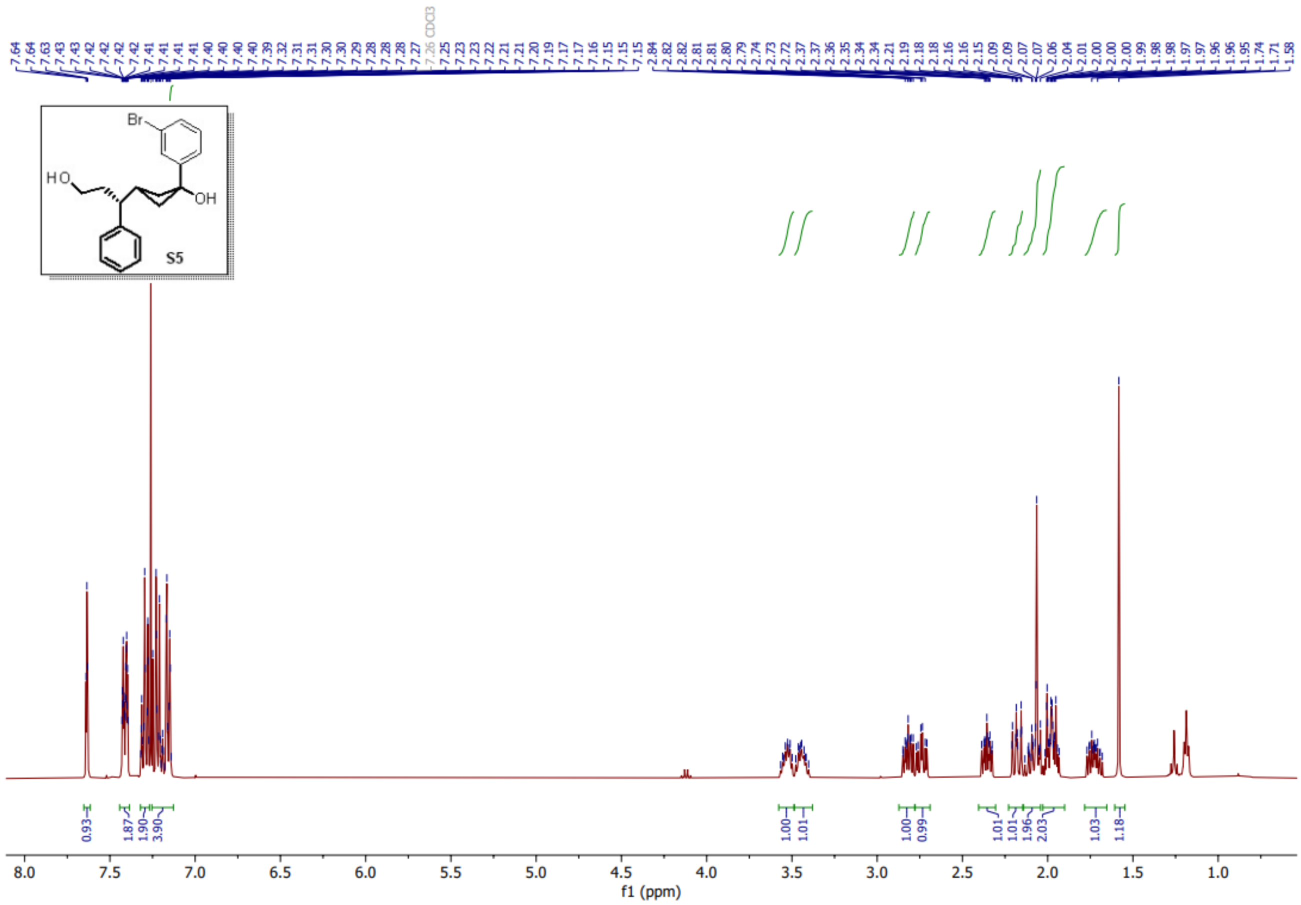

91336 shc-180-2.11.fid

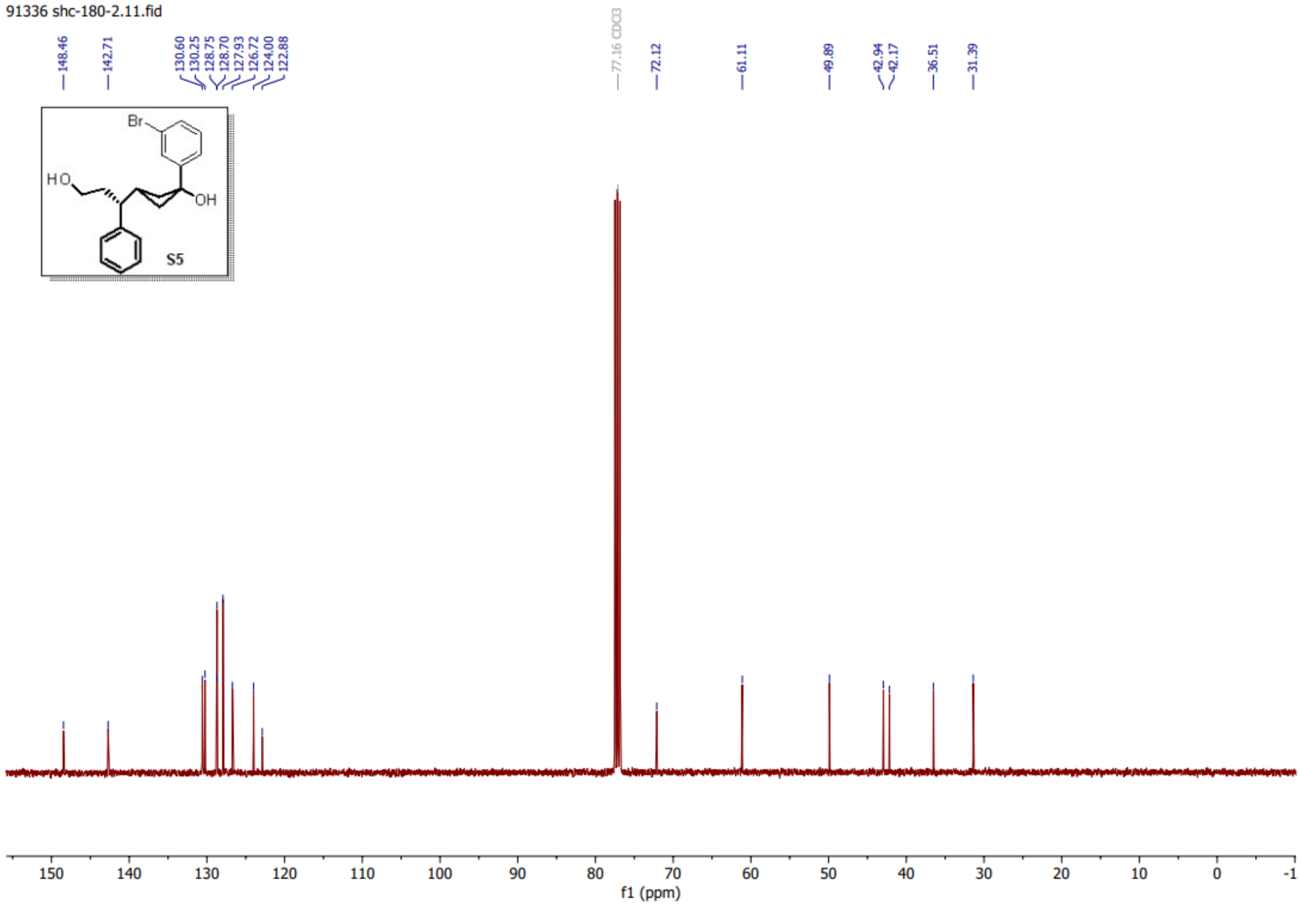

5.2 HPLC Spectra

Compound 5

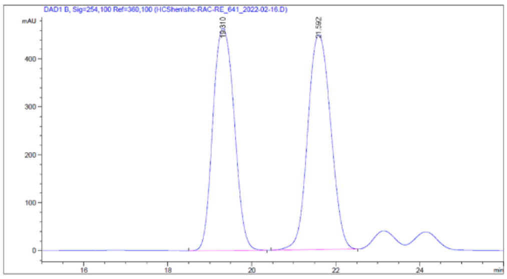

Peak #	RetTime [min]	Type	Width [min]	Area [mAU*s]	Height [mAU]	Area %
1	19.310	BB	0.4306	1.63962e4	463.60202	49.0278
2	21.592	BB	0.4598	1.70464e4	448.07132	50.9722

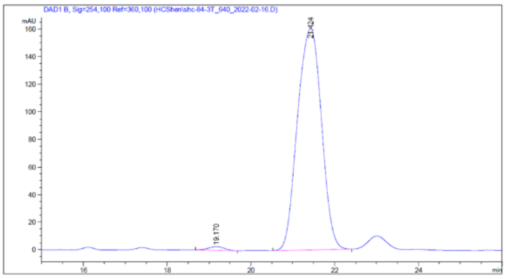

Peak #	RetTime [min]	Type	Width [min]	Area [mAU*s]	Height [mAU]	Area %
1	19.170	BB	0.3027	68.31631	2.66346	1.0577
2	21.424	BB	0.5489	6390.70459	160.72681	98.9423

Compound 6

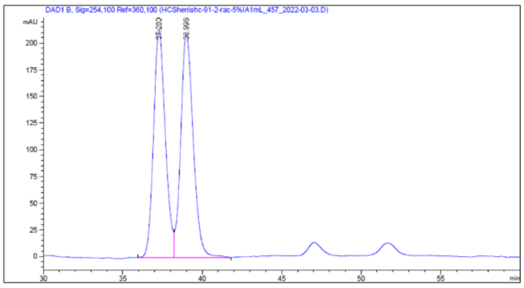

Peak #	RetTime [min]	Type	Width [min]	Area [mAU*s]	Height [mAU]	Area %
1	37.280	BV	0.7153	1.10151e4	214.05949	49.0368
2	38.998	VB	0.7519	1.14478e4	209.35629	50.9632

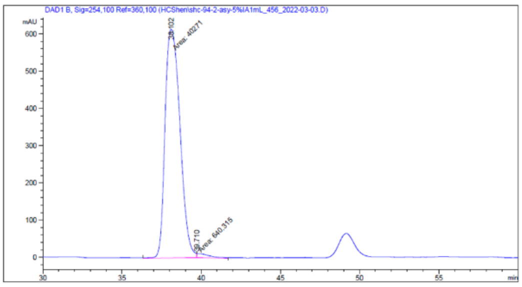

Peak #	RetTime [min]	Type	Width [min]	Area [mAU*s]	Height [mAU]	Area %
1	38.102	MM	1.0919	4.02710e4	614.68109	98.4349
2	39.710	MM	0.6553	640.31537	11.40905	1.5651

Compound 7

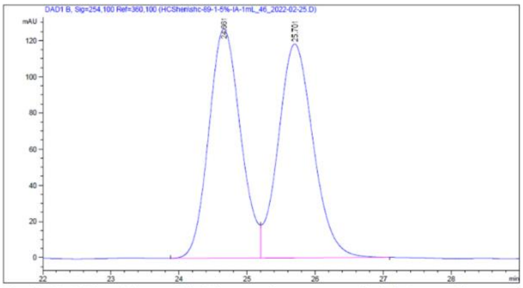

Peak #	RetTime [min]	Type	Width [min]	Area [mAU*s]	Height [mAU]	Area %
1	24.661	BV	0.4890	4105.99707	126.72458	49.2540
2	25.701	VB	0.5211	4230.38037	118.19627	50.7460

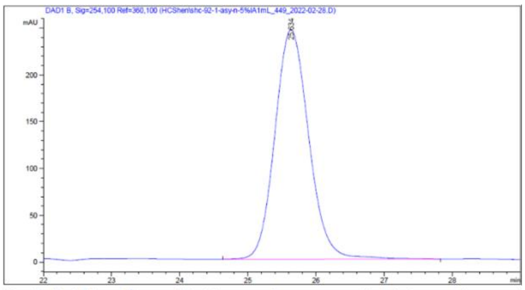

Peak #	RetTime [min]	Type	Width [min]	Area [mAU*s]	Height [mAU]	Area %
1	25.634	BB	0.5193	8478.03223	244.94186	100.0000

Compound 8

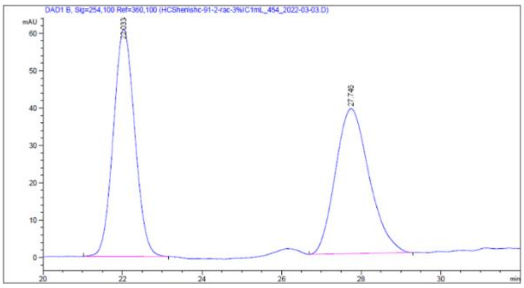

Peak #	RetTime [min]	Type	Width [min]	Area [mAU*s]	Height [mAU]	Area %
1	22.033	BB	0.5419	2270.92090	60.78239	49.8588
2	27.748	BB	0.6921	2283.78027	38.80461	50.1412

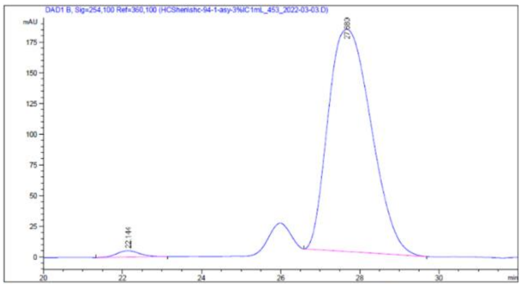

Peak #	RetTime [min]	Type	Width [min]	Area [mAU*s]	Height [mAU]	Area %
1	22.144	BB	0.4807	211.81494	5.17293	1.5213
2	27.680	BB	0.8985	1.37118e4	181.06375	98.4787

Compound 9

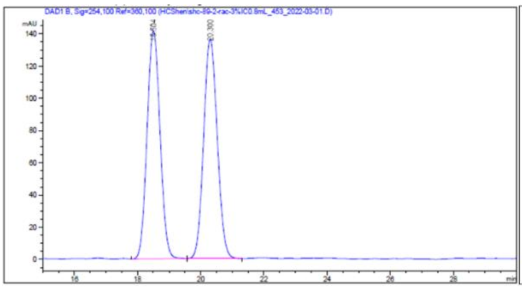

Peak #	RetTime [min]	Type	Width [min]	Area [mAU*s]	Height [mAU]	Area %
1	18.504	BB	0.4427	4027.41797	141.43639	49.7399
2	20.300	BB	0.4563	4069.53442	136.30307	50.2601

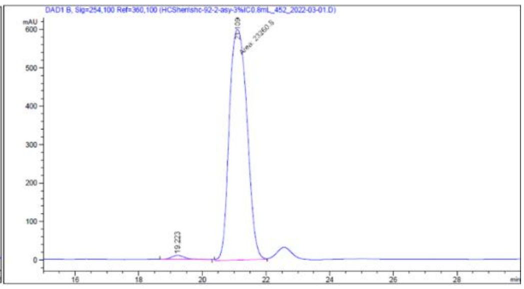

Peak #	RetTime [min]	Type	Width [min]	Area [mAU*s]	Height [mAU]	Area %
1	19.223	BB	0.3608	316.61487	10.83725	1.3429
2	21.109	MM	0.6456	2.32608e4	600.48718	98.6571

Compound 10

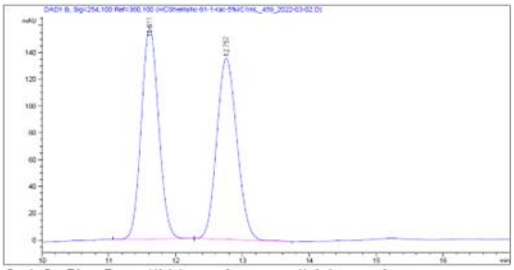

Peak #	RetTime [min]	Type	Width [min]	Area [mAU*s]	Height [mAU]	Area %
1	11.611	BB	0.2819	2836.26245	157.65063	50.0013
2	12.757	BB	0.3256	2836.11157	135.02512	49.9987

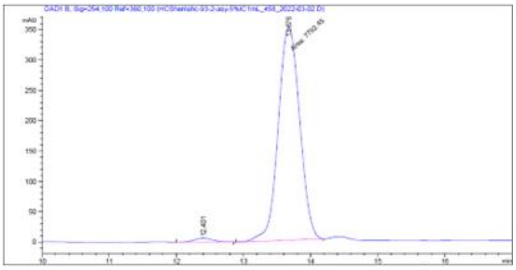

Peak #	RetTime [min]	Type	Width [min]	Area [mAU*s]	Height [mAU]	Area %
1	12.401	BB	0.2313	120.87782	6.19046	1.5275
2	13.675	MM	0.3700	7792.45313	351.04822	98.4725

Compound 11

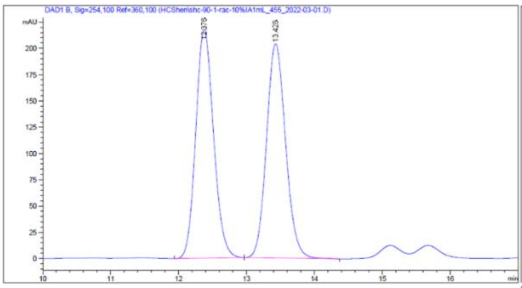

Peak #	RetTime [min]	Type	Width [min]	Area [mAU*s]	Height [mAU]	Area %
1	12.376	BB	0.2812	3968.49463	217.63937	49.8888
2	13.428	BB	0.3048	3986.19189	203.72865	50.1112

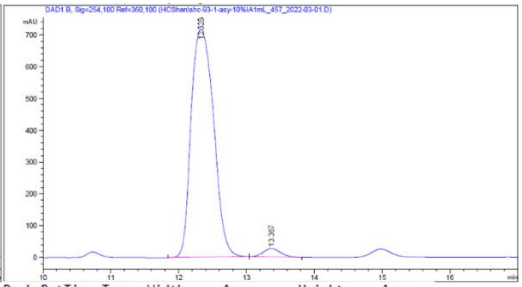

Peak #	RetTime [min]	Type	Width [min]	Area [mAU*s]	Height [mAU]	Area %
1	12.328	BB	0.3392	1.65108e4	716.93134	97.2707
2	13.367	BB	0.2680	463.27966	26.36577	2.7293

Compound 12

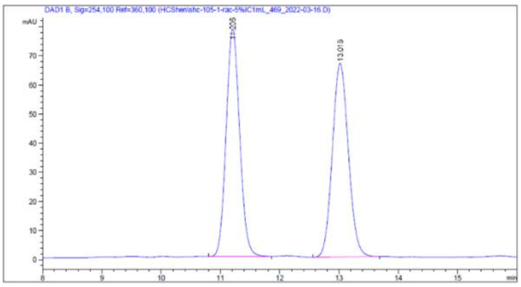

Peak #	RetTime [min]	Type	Width [min]	Area [mAU*s]	Height [mAU]	Area %
1	11.206	BB	0.2473	1235.62683	78.01012	49.7987
2	13.018	BB	0.2924	1245.61572	66.53074	50.2013

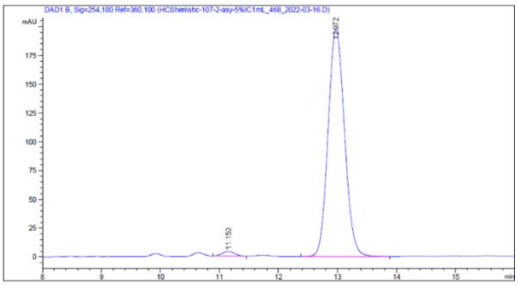

Peak #	RetTime [min]	Type	Width [min]	Area [mAU*s]	Height [mAU]	Area %
1	11.150	BB	0.1936	58.93278	4.01770	1.5189
2	12.972	BB	0.3021	3821.04321	197.59338	98.4811

Compound 13

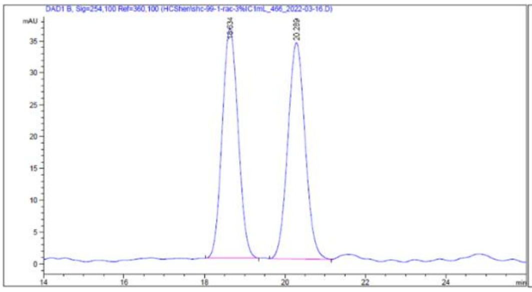

Peak #	RetTime [min]	Type	Width [min]	Area [mAU*s]	Height [mAU]	Area %
1	18.634	BB	0.3949	992.10291	36.00758	49.4833
2	20.289	BB	0.4277	1012.82190	33.93541	50.5167

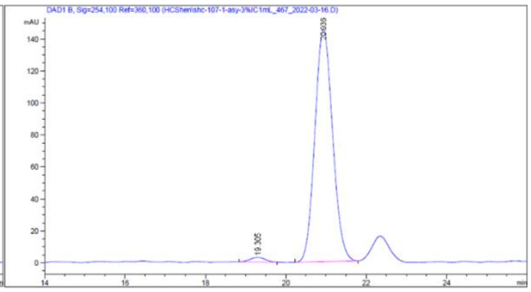

Peak #	RetTime [min]	Type	Width [min]	Area [mAU*s]	Height [mAU]	Area %
1	19.305	BB	0.3006	72.80354	2.84913	1.6067
2	20.936	BB	0.4717	4458.54834	145.03069	98.3933

Compound 14

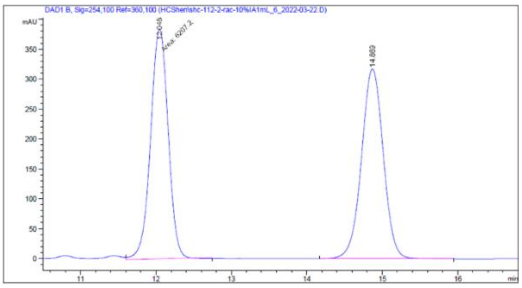

Peak #	RetTime [min]	Type	Width [min]	Area [mAU*s]	Height [mAU]	Area %
1	12.048	MM	0.2698	6207.19922	383.42764	49.3826
2	14.869	BB	0.3111	6362.41455	317.14163	50.6174

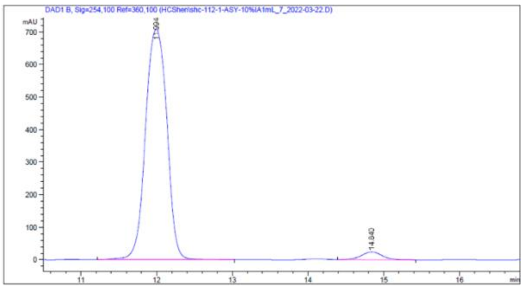

Peak #	RetTime [min]	Type	Width [min]	Area [mAU*s]	Height [mAU]	Area %
1	11.994	BB	0.3219	1.39015e4	708.51379	96.8490
2	14.840	BB	0.2739	452.29465	23.94093	3.1510

Compound 15

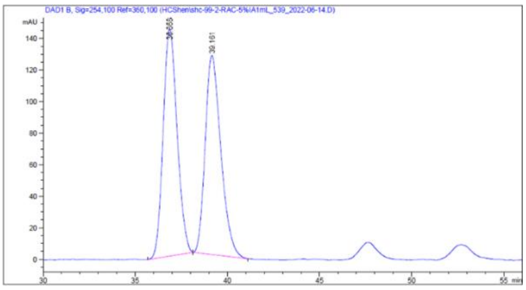

Peak #	RetTime [min]	Type	Width [min]	Area [mAU*s]	Height [mAU]	Area %
1	36.868	BB	0.7336	7578.35254	143.73895	50.3633
2	39.161	BB	0.8036	7469.02002	126.35586	49.6367

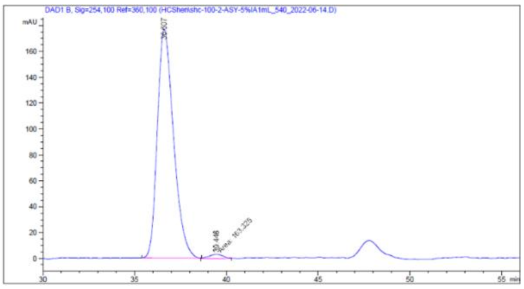

Peak #	RetTime [min]	Type	Width [min]	Area [mAU*s]	Height [mAU]	Area %
1	36.607	BB	0.8158	1.08471e4	176.79236	98.5166
2	39.446	MM	0.8353	163.32463	3.25871	1.4834

Compound 16

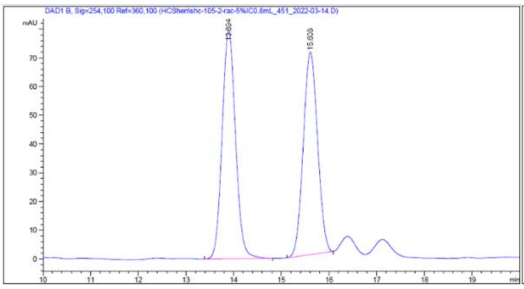

Peak #	RetTime [min]	Type	Width [min]	Area [mAU*s]	Height [mAU]	Area %
1	13.894	BB	0.2972	1552.17285	79.51478	51.3978
2	15.608	BB	0.3138	1467.74805	70.52084	48.6022

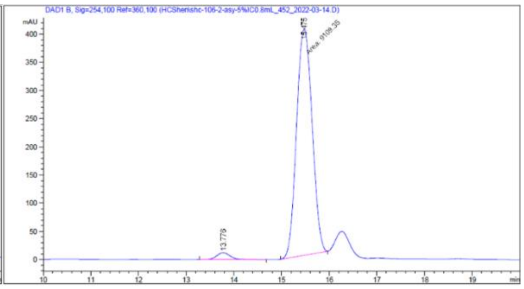

Peak #	RetTime [min]	Type	Width [min]	Area [mAU*s]	Height [mAU]	Area %
1	13.776	BB	0.2743	239.84187	12.09671	2.5654
2	15.475	MM	0.3787	9109.35059	400.87036	97.4346

Compound 17

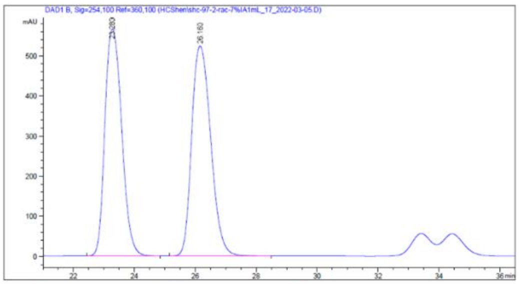

Peak #	RetTime [min]	Type	Width [min]	Area [mAU*s]	Height [mAU]	Area %
1	23.280	BB	0.4568	2.16321e4	564.88116	48.7993
2	26.160	BB	0.5103	2.26967e4	523.02344	51.2007

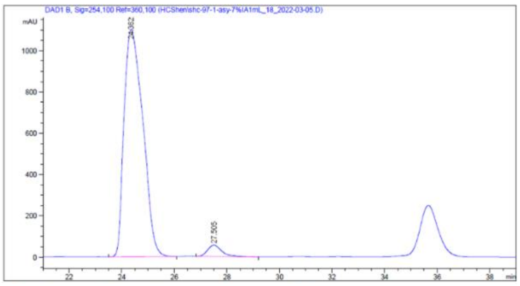

Peak #	RetTime [min]	Type	Width [min]	Area [mAU*s]	Height [mAU]	Area %
1	24.362	BB	0.5901	5.47371e4	1102.79224	96.3127
2	27.505	BB	0.5296	2095.56738	54.87165	3.6873

Compound 18

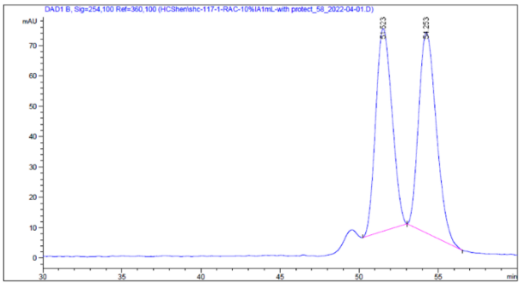

Peak #	RetTime [min]	Type	Width [min]	Area [mAU*s]	Height [mAU]	Area %
1	51.523	BB	0.8422	4778.36865	66.63052	48.1924
2	54.253	BB	0.9110	5136.81885	66.00024	51.8076

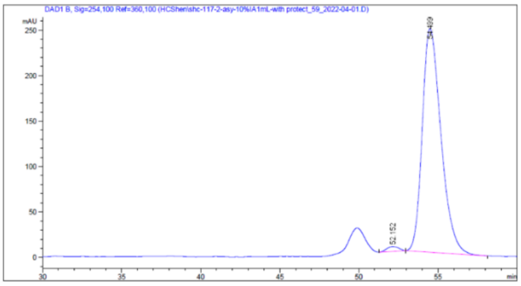

Peak #	RetTime [min]	Type	Width [min]	Area [mAU*s]	Height [mAU]	Area %
1	52.152	BB	0.6294	282.92825	5.26947	1.3801
2	54.499	BB	0.9703	2.02174e4	245.87523	98.6199

Compound 19

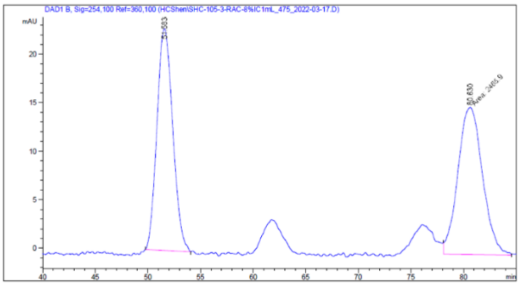

Peak #	RetTime [min]	Type	Width [min]	Area [mAU*s]	Height [mAU]	Area %
1	51.583	BB	1.2225	2390.09644	22.87565	49.2195
2	80.630	MM	2.7108	2465.90161	15.16074	50.7805

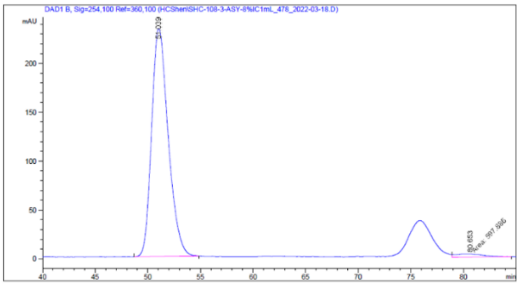

Peak #	RetTime [min]	Type	Width [min]	Area [mAU*s]	Height [mAU]	Area %
1	51.039	BB	1.2934	2.55104e4	233.07855	97.7100
2	80.653	MM	3.0988	597.88568	3.21565	2.2900

Compound 20

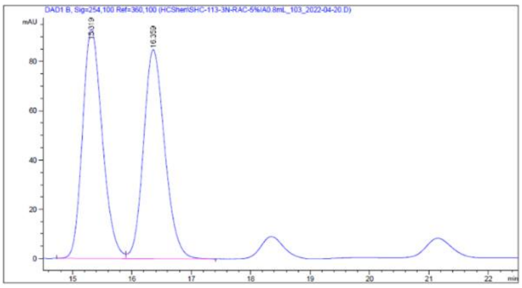

Peak #	RetTime [min]	Type	Width [min]	Area [mAU*s]	Height [mAU]	Area %
1	15.319	BV	0.3518	2149.99829	92.95711	50.5459
2	16.359	VB	0.3694	2103.55981	84.81085	49.4541

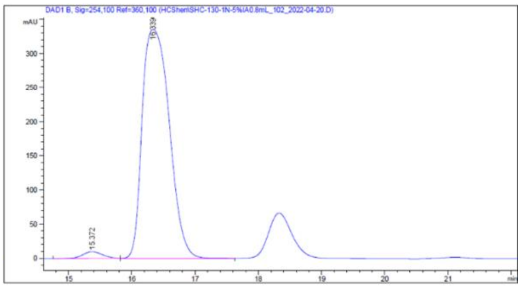

Peak #	RetTime [min]	Type	Width [min]	Area [mAU*s]	Height [mAU]	Area %
1	15.372	BB	0.2717	214.43486	9.95968	2.1067
2	16.339	BB	0.3520	9964.06543	335.02734	97.8933

Compound 21

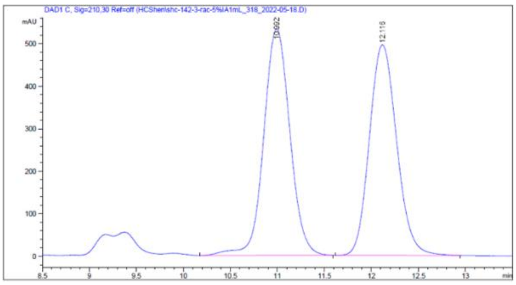

Peak #	RetTime [min]	Type	Width [min]	Area [mAU*s]	Height [mAU]	Area %
1	10.992	VV R	0.2726	1.03421e4	532.58496	50.8698
2	12.116	VV R	0.2626	9988.43457	495.65665	49.1302

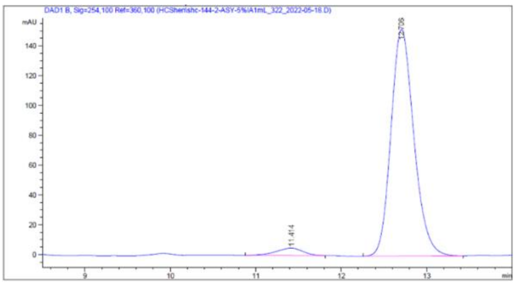

Peak #	RetTime [min]	Type	Width [min]	Area [mAU*s]	Height [mAU]	Area %
1	11.414	BB	0.2598	105.75220	4.88109	3.6053
2	12.706	BB	0.2841	2827.47534	151.93550	96.3947

Compound S5 for 22

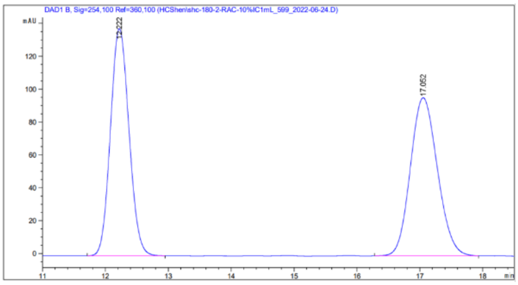

Peak #	RetTime [min]	Type	Width [min]	Area [mAU*s]	Height [mAU]	Area %
1	12.222	BB	0.3169	2827.51929	138.06334	49.6221
2	17.052	BB	0.4379	2870.58740	96.11781	50.3779

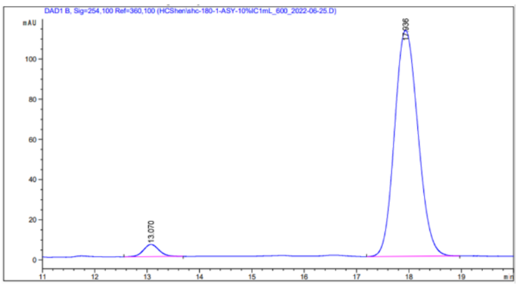

Peak #	RetTime [min]	Type	Width [min]	Area [mAU*s]	Height [mAU]	Area %
1	13.070	BB	0.2510	126.35912	6.07867	3.4932
2	17.936	BB	0.4629	3490.88599	112.52641	96.5068

Compound 23

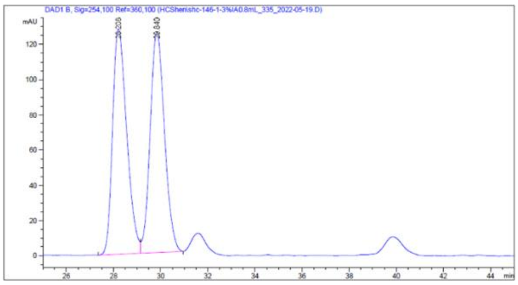

Peak #	RetTime [min]	Type	Width [min]	Area [mAU*s]	Height [mAU]	Area %
1	28.208	BV	0.5931	5331.43750	127.96606	50.1242
2	29.840	VB	0.5538	5305.01563	125.33792	49.8758

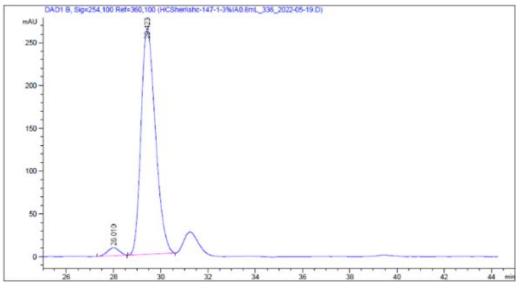

Peak #	RetTime [min]	Type	Width [min]	Area [mAU*s]	Height [mAU]	Area %
1	28.010	BB	0.3983	320.41327	9.42054	2.7925
2	29.423	BB	0.6053	1.11538e4	263.24930	97.2075

Compound 24

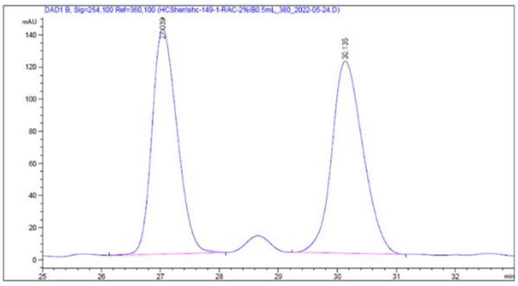

Peak #	RetTime [min]	Type	Width [min]	Area [mAU*s]	Height [mAU]	Area %
1	27.039	BB	0.4382	4130.61670	140.01152	48.4483
2	30.135	BB	0.5330	4395.20850	119.65981	51.5517

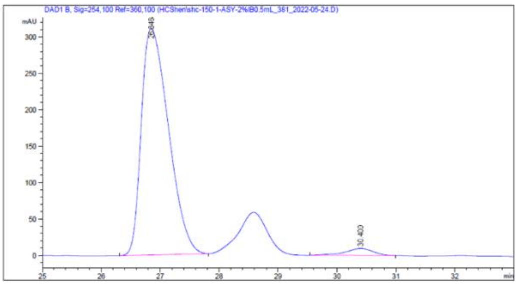

Peak #	RetTime [min]	Type	Width [min]	Area [mAU*s]	Height [mAU]	Area %
1	26.848	BB	0.4534	9954.00586	310.55759	96.9808
2	30.400	BB	0.3828	309.89014	9.48076	3.0192

Compound 25

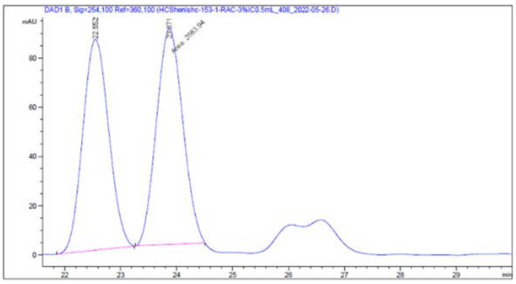

Peak #	RetTime [min]	Type	Width [min]	Area [mAU*s]	Height [mAU]	Area %
1	22.552	BB	0.4861	2754.98779	85.56284	49.0305
2	23.871	MM	0.5460	2863.93896	87.42087	50.9695

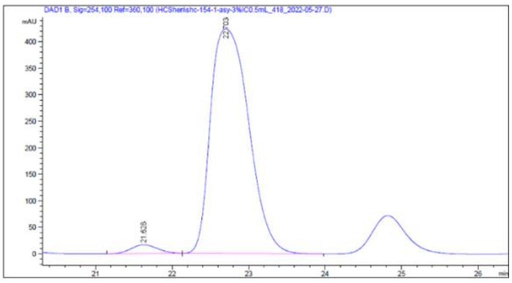

Peak #	RetTime [min]	Type	Width [min]	Area [mAU*s]	Height [mAU]	Area %
1	21.628	BB	0.3113	397.01822	16.61236	2.6292
2	22.703	BB	0.4128	1.47034e4	423.23077	97.3708

Compound 26

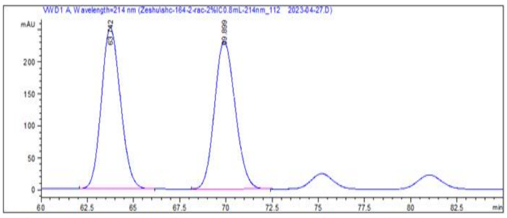

Peak #	RetTime [min]	Type	Width [min]	Area [mAU*s]	Height [mAU]	Area %
1	63.742	BV R	0.8839	1.80810e4	251.59697	50.0948
2	69.899	BV R	0.9501	1.80125e4	230.44576	49.9052

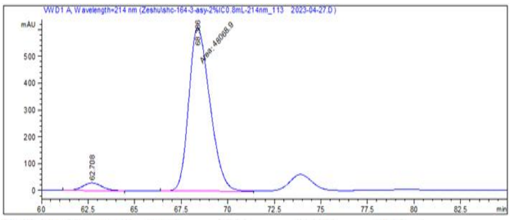

Peak #	RetTime [min]	Type	Width [min]	Area [mAU*s]	Height [mAU]	Area %
1	62.708	BB	0.8223	1974.19592	28.15923	3.9450
2	68.386	MM	1.3158	4.80689e4	608.87329	96.0550

Compound 27

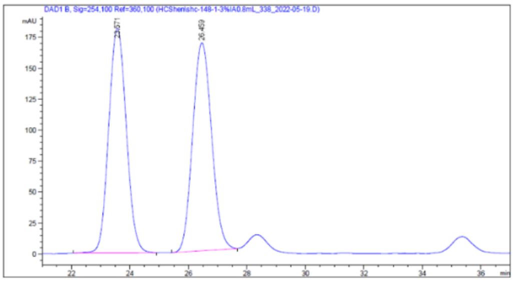

Peak #	RetTime [min]	Type	Width [min]	Area [mAU*s]	Height [mAU]	Area %
1	23.571	BB	0.5006	7585.13721	181.29274	50.0671
2	26.459	BB	0.5307	7564.80420	168.02957	49.9329

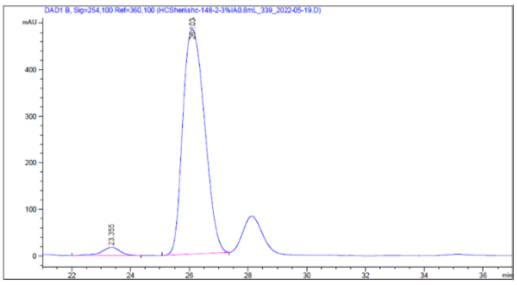

Peak #	RetTime [min]	Type	Width [min]	Area [mAU*s]	Height [mAU]	Area %
1	23.355	BB	0.5085	755.04041	17.42467	2.9388
2	26.103	BB	0.6040	2.49370e4	482.87842	97.0612

Compound 28

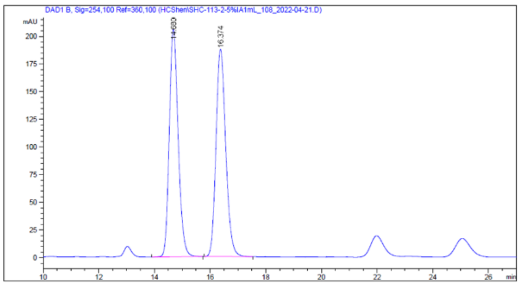

Peak #	RetTime [min]	Type	Width [min]	Area [mAU*s]	Height [mAU]	Area %
1	14.680	BB	0.3377	4548.08984	205.87820	49.9876
2	16.374	BB	0.3748	4550.35254	187.36493	50.0124

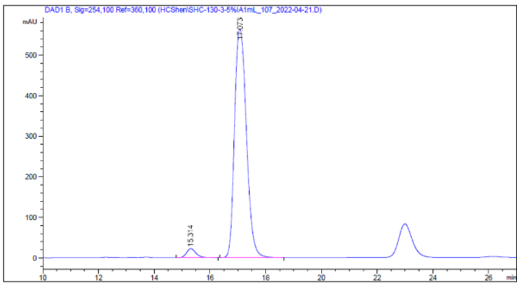

Peak #	RetTime [min]	Type	Width [min]	Area [mAU*s]	Height [mAU]	Area %
1	15.314	BB	0.3296	522.11053	22.22619	3.0398
2	17.073	BB	0.4158	1.66537e4	562.64795	96.9602

Compound 29

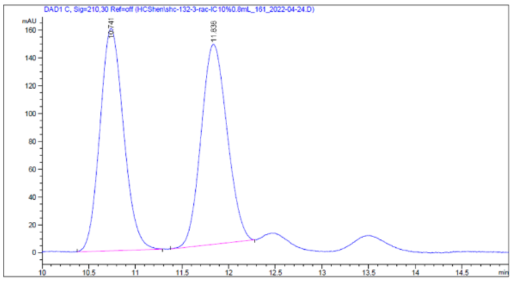

Peak #	RetTime [min]	Type	Width [min]	Area [mAU*s]	Height [mAU]	Area %
1	10.741	BV R	0.2271	2821.06250	160.05315	50.2919
2	11.838	VV R	0.2293	2788.30981	144.05669	49.7081

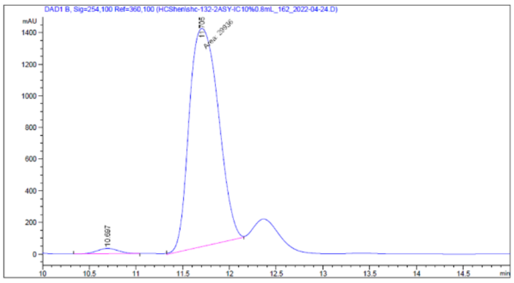

Peak #	RetTime [min]	Type	Width [min]	Area [mAU*s]	Height [mAU]	Area %
1	10.697	BB	0.2389	522.11481	33.02598	1.7142
2	11.705	MM	0.2554	2.99360e4	1379.25757	98.2858

Compound 30

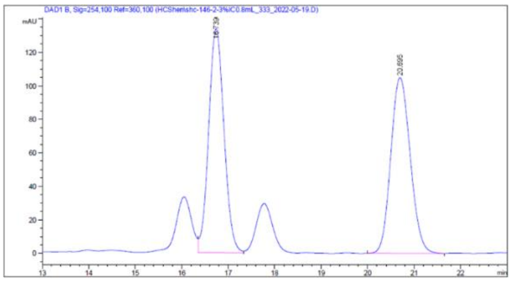

Peak #	RetTime [min]	Type	Width [min]	Area [mAU*s]	Height [mAU]	Area %
1	16.739	VV	0.3545	3064.75684	133.69008	50.1772
2	20.695	BB	0.4389	3043.11450	104.91634	49.8228

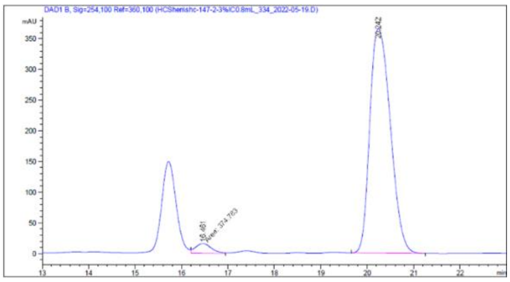

Peak #	RetTime [min]	Type	Width [min]	Area [mAU*s]	Height [mAU]	Area %
1	16.461	MM	0.3932	374.78281	15.88612	3.2238
2	20.242	BB	0.3697	1.12509e4	365.44540	96.7762

Compound 31

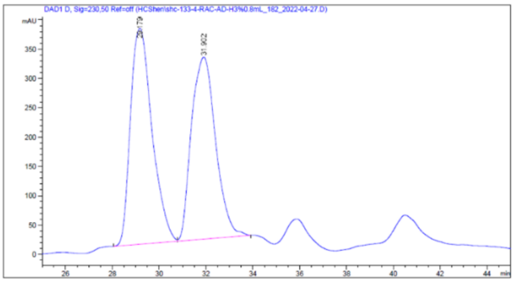

Peak #	RetTime [min]	Type	Width [min]	Area [mAU*s]	Height [mAU]	Area %
1	29.179	BV	0.7740	2.35524e4	368.08917	51.4013
2	31.902	VB	0.8941	2.22682e4	310.33441	48.5987

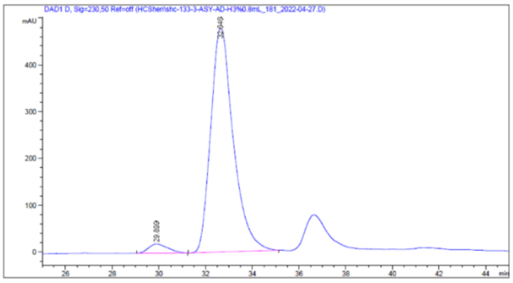

Peak #	RetTime [min]	Type	Width [min]	Area [mAU*s]	Height [mAU]	Area %
1	29.899	BB	0.6704	1121.74048	19.62365	3.3791
2	32.646	BB	0.7957	3.20750e4	477.82303	96.6209

Compound 32

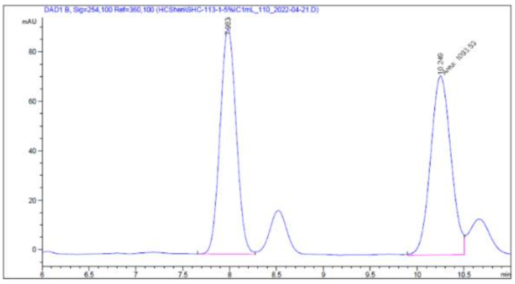

Peak #	RetTime [min]	Type	Width [min]	Area [mAU*s]	Height [mAU]	Area %
1	7.983	BV	0.1869	1091.80859	91.02465	49.9607
2	10.249	MM	0.2523	1093.52502	72.24755	50.0393

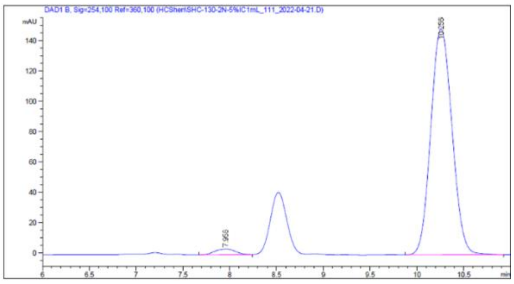

Peak #	RetTime [min]	Type	Width [min]	Area [mAU*s]	Height [mAU]	Area %
1	7.958	BB	0.1779	55.09359	3.68570	2.2950
2	10.256	BB	0.2455	2345.53271	148.73322	97.7050

Compound 33

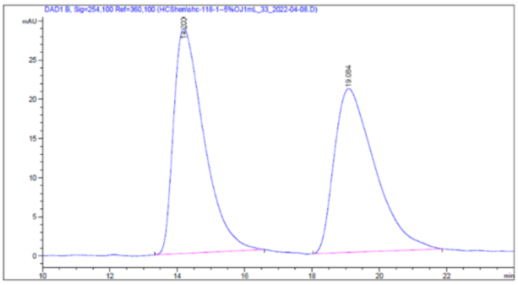

Peak #	RetTime [min]	Type	Width [min]	Area [mAU*s]	Height [mAU]	Area %
1	14.200	BB	0.7325	1795.31165	28.69302	50.6526
2	19.084	BB	0.9796	1749.05212	20.90558	49.3474

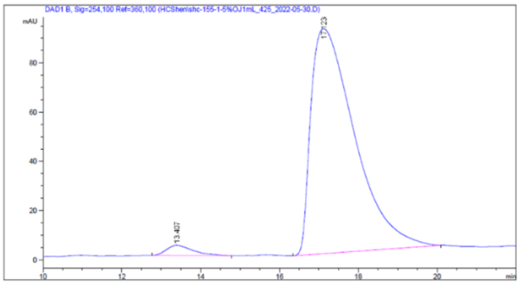

Peak #	RetTime [min]	Type	Width [min]	Area [mAU*s]	Height [mAU]	Area %
1	13.407	BB	0.5488	192.70482	4.12296	2.6587
2	17.123	BB	0.9039	7055.43262	91.37247	97.3413

Compound 34

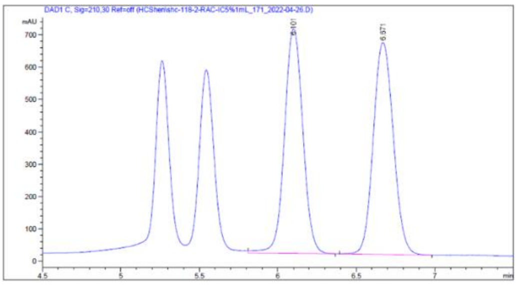

Peak #	RetTime [min]	Type	Width [min]	Area [mAU*s]	Height [mAU]	Area %
1	6.101	VB	0.1266	5656.92578	694.14777	49.3153
2	6.671	VV R	0.1395	5814.00391	655.12659	50.6847

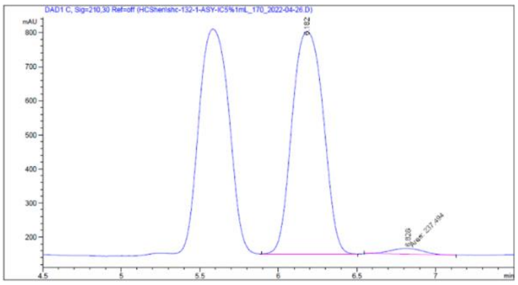

Peak #	RetTime [min]	Type	Width [min]	Area [mAU*s]	Height [mAU]	Area %
1	6.182	BB	0.2268	9033.05762	653.42535	97.4382
2	6.828	MM	0.2317	237.49385	17.08699	2.5618

Compound S4 for 35

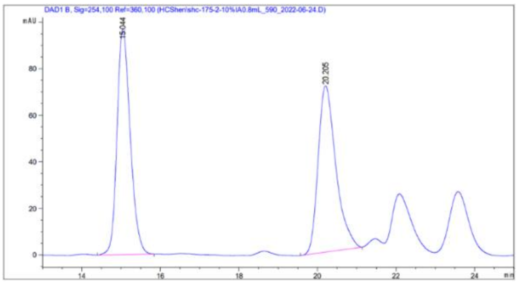

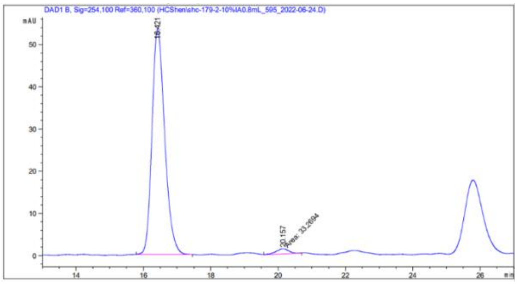

Peak #	RetTime [min]	Type	Width [min]	Area [mAU*s]	Height [mAU]	Area %
1	15.044	BB	0.3558	2260.62451	97.01794	50.1771
2	20.205	BB	0.4565	2244.66235	71.44575	49.8229

Peak #	RetTime [min]	Type	Width [min]	Area [mAU*s]	Height [mAU]	Area %
1	16.421	BB	0.3734	1399.68274	53.47049	97.6783
2	20.157	MM	0.4484	33.26939	1.23647	2.3217

Compound 36

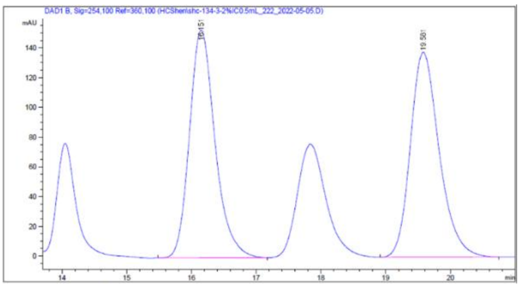

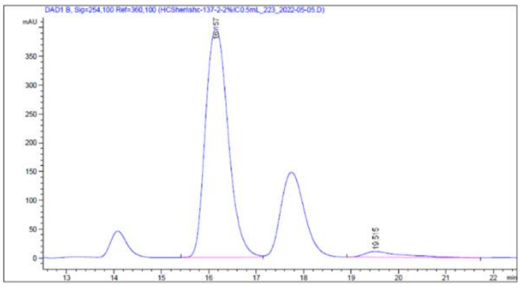

Peak #	RetTime [min]	Type	Width [min]	Area [mAU*s]	Height [mAU]	Area %
1	16.151	BB	0.3742	4193.98926	153.05721	49.5497
2	19.581	BB	0.4103	4270.22217	137.88162	50.4503

Peak #	RetTime [min]	Type	Width [min]	Area [mAU*s]	Height [mAU]	Area %
1	16.157	BV	0.4051	1.32044e4	396.47189	95.9934
2	19.515	BB	0.6404	551.12433	10.07957	4.0066

Compound 37

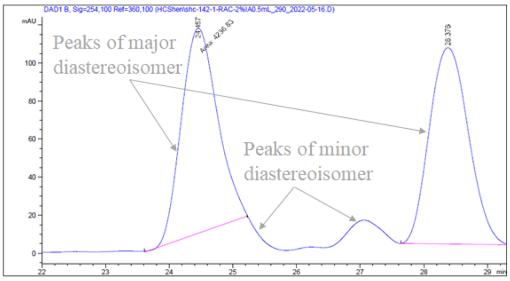

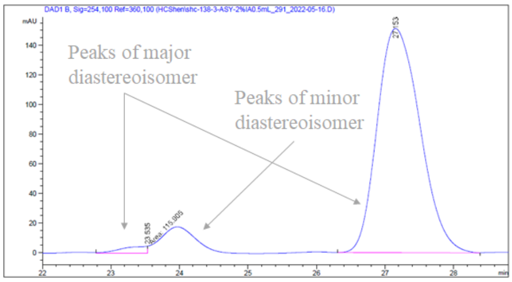

Peak #	RetTime [min]	Type	Width [min]	Area [mAU*s]	Height [mAU]	Area %
1	24.457	MM	0.6559	4236.82666	107.66248	49.0018
2	28.378	BB	0.5204	4409.43750	103.22233	50.9982

Peak #	RetTime [min]	Type	Width [min]	Area [mAU*s]	Height [mAU]	Area %
1	23.535	MM	0.4182	115.90493	4.61877	1.7354
2	27.153	BB	0.5174	6562.94434	151.18600	98.2646

Compound 38

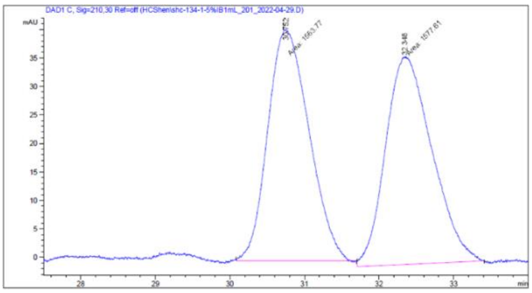

Peak #	RetTime [min]	Type	Width [min]	Area [mAU*s]	Height [mAU]	Area %
1	30.752	MM	0.6419	1563.76758	40.60304	49.7797
2	32.348	MM	0.7212	1577.60779	36.45615	50.2203

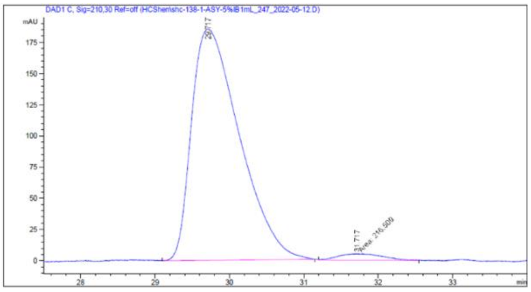

Peak #	RetTime [min]	Type	Width [min]	Area [mAU*s]	Height [mAU]	Area %
1	29.717	VV R	0.5272	8304.74023	185.18381	97.4592
2	31.717	MM	0.6947	216.50920	5.19410	2.5408

Compound S3 for 39

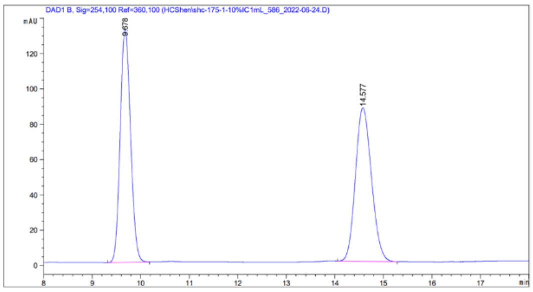

Peak #	RetTime [min]	Type	Width [min]	Area [mAU*s]	Height [mAU]	Area %
1	9.678	BB	0.2329	1952.88647	131.80045	49.4738
2	14.577	BB	0.3513	1994.42676	86.89904	50.5262

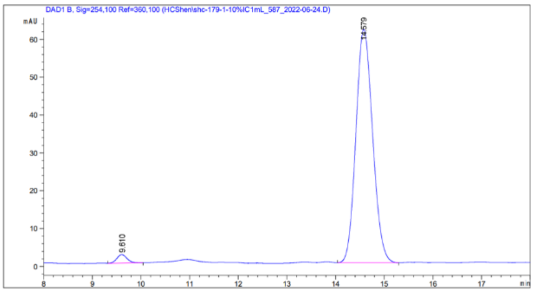

Peak #	RetTime [min]	Type	Width [min]	Area [mAU*s]	Height [mAU]	Area %
1	9.610	BB	0.1751	32.84783	2.25984	2.1824
2	14.579	BB	0.3590	1472.27051	61.66578	97.8176

Compound 40

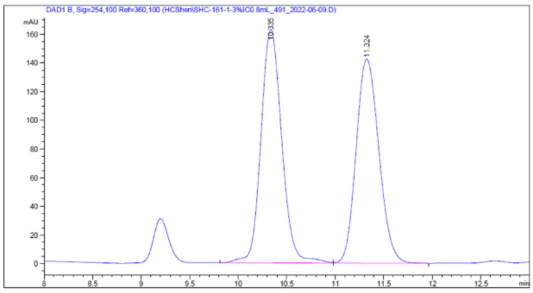

Peak #	RetTime [min]	Type	Width [min]	Area [mAU*s]	Height [mAU]	Area %
1	10.335	BV	0.2310	2427.61182	162.77521	51.1386
2	11.324	VB	0.2554	2319.51367	142.49115	48.8614

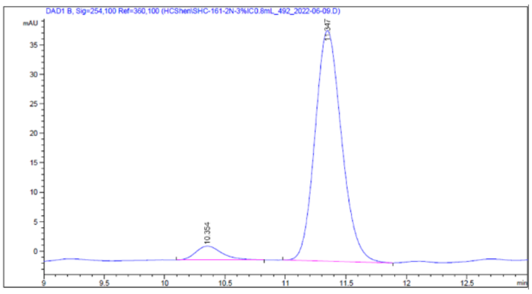

Peak #	RetTime [min]	Type	Width [min]	Area [mAU*s]	Height [mAU]	Area %
1	10.354	BB	0.1804	35.30305	2.35528	5.5874
2	11.347	BB	0.2315	596.52472	39.08929	94.4126
